# A hidden proteome encoded by circRNAs in human placentas: Implications for uncovering preeclampsia pathogenesis

**DOI:** 10.1002/ctm2.1759

**Published:** 2024-07-12

**Authors:** Huanqiang Zhao, Yu Xiong, Zixiang Zhou, Qixin Xu, Yang Zi, Xiujie Zheng, Shiguo Chen, Xirong Xiao, Lili Gong, Huangfang Xu, Lidong Liu, Huiqing Lu, Yutong Cui, Shuyi Shao, Jin Zhang, Jing Ma, Qiongjie Zhou, Duan Ma, Xiaotian Li

**Affiliations:** ^1^ The Shanghai Key Laboratory of Female Reproductive Endocrine‐Related Diseases Obstetrics and Gynecology Hospital, Fudan University Shanghai China; ^2^ Institute of Maternal and Child Medicine Shenzhen Maternity and Child Healthcare Hospital Shenzhen Guangdong Province China; ^3^ Key Laboratory of Metabolism and Molecular Medicine, Ministry of Education, Department of Biochemistry and Molecular Biology, School of Basic Medical Sciences Fudan University Shanghai China

**Keywords:** circRNA‐encoded protein, circular RNA, placenta, preeclampsia

## Abstract

**Background:**

**C**ircRNA‐**e** ncoded **p** roteins (CEPs) are emerging as new players in health and disease, and function as baits for the common partners of their cognate **l** inear‐spliced RNA **e** ncoded **p** roteins (LEPs). However, their prevalence across human tissues and biological roles remain largely unexplored. The placenta is an ideal model for identifying CEPs due to its considerable protein diversity that is required to sustain fetal development during pregnancy. The aim of this study was to evaluate circRNA translation in the human placenta, and the potential roles of the CEPs in placental development and dysfunction.

**Methods:**

Multiomics approaches, including RNA sequencing, ribosome profiling, and LC‐MS/MS analysis, were utilised to identify novel translational events of circRNAs in human placentas. Bioinformatics methods and the protein bait hypothesis were employed to evaluate the roles of these newly discovered CEPs in placentation and associated disorders. The pathogenic role of a recently identified CEP circPRKCB119aa in preeclampsia was investigated through qRT‐PCR, Western blotting, immunofluorescence imaging and phenotypic analyses.

**Results:**

We found that 528 placental circRNAs bound to ribosomes with active translational elongation, and 139 were translated to proteins. The CEPs showed considerable structural homology with their cognate LEPs, but are more stable, hydrophobic and have a lower molecular‐weight than the latter, all of which are conducive to their function as baits. On this basis, CEPs are deduced to be closely involved in placental function. Furthermore, we focused on a novel CEP circPRKCB119aa, and illuminated its pathogenic role in preeclampsia; it enhanced trophoblast autophagy by acting as a bait to inhibit phosphorylation of the cognate linear isoform PKCβ.

**Conclusions:**

We discovered a hidden circRNA‐encoded proteome in the human placenta, which offers new insights into the mechanisms underlying placental development, as well as placental disorders such as preeclampsia.

**Key points**

- A hidden circRNA‐encoded proteome in the human placenta was extensively identified and systematically characterised.
- The circRNA‐encoded proteins (CEPs) are potentially related to placental development and associated disorders.
- A novel conserved CEP circPRKCB119aa enhanced trophoblast autophagy by inhibiting phosphorylation of its cognate linear‐spliced isoform protein kinase C (PKC) β in preeclampsia.

## Graphical abstract

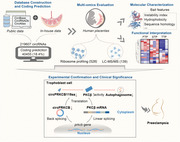

## 1 INTRODUCTION

Circular RNAs (circRNAs) have long been regarded as endogenous noncoding transcripts with covalently closed loop structures. Traditionally, this RNA category was thought to function as sponges for proteins, microRNAs (miRNAs), or other RNA species, or to serve in a scaffolding role that facilitates the formation of functional complexes.^1^, ^2^ However, recent evidence indicates that circRNAs can also serve as translational templates and encode small functional peptides,^3^, ^4^, ^5^, ^6^, ^7^, ^8^ which are appropriately called **c** ircRNA‐**e** ncoded **p** roteins (**CEPs**). This considerably broadens the complexity of the mammalian proteomes, necessitating a reassessment of the established assumptions regarding the biological roles of circRNAs based solely on the RNA structure.

CircRNAs are limited to cap‐independent translation as they lack a 5′ cap. Internal ribosomal entry sites (IRES) and N^6^‐methyladenosine (m^6^A)‐mediated initiation are the two widely accepted mechanisms of cap‐independent translation.^9^, ^10^ Recent studies show that a single m^6^A motif or IRES (or IRES‐like) is sufficient to drive translation, and these elements are surprisingly enriched in circRNA sequence, highlighting the possibility of a pervasive translation of circRNAs.^9^, ^11^ However, apart from the identification of 40 translated circRNAs in the human heart,^12^ little has been published on the global identification of CEPs so far.

Recent advances in omics technology and theory have made it possible to comprehensively identify CEPs, and uncover their roles in physiology and pathophysiology. Ribosome profiling and LC‐MS/MS are the two powerful tools that can robustly profile noncanonical translation.^12^, ^13^, ^14^ In terms of functional mechanism, a compelling body of evidence suggests that the unique characteristics of CEPs, such as low molecular weight and high‐level homology to the cognate isoforms, allow them to competitively bind to common partners of cognate **l** inear‐spliced RNA **e** ncoded **p** roteins (**LEPs**), thereby inhibiting the latter's functions.^3^, ^15^, ^16^, ^17^ This phenomenon is known as the ‘bait effect’.^18^ Despite these advances, functional impacts of only a handful of tumourigenicity‐related CEPs have been reported so far,^13^, ^14^, ^19^ and limited information is available regarding their potential role in human development and diseases.

The placenta, an essential link between the developing embryo and mother, provides an ideal model for identifying CEPs due to its considerable protein diversity that is required to maintain pregnancy.^20^ During its transient existence, the placenta performs the functions that are postnatally executed by various neonate organs, including the lungs, liver, kidneys and endocrine glands.^21^ Defects in placentation often result in gestational complications including preeclampsia, a hypertensive condition of unknown mechanistic basis that affects 4%–8% of all pregnancies.^22^ Recently, a large repertoire of circRNAs with unknown biological functions have been identified that are dysregulated in placental disorders.^23^, ^24^, ^25^ This circRNA pool can be used to identify novel CEPs and provide new insights into the molecular mechanisms of placental dysfunction.

**FIGURE 1:**
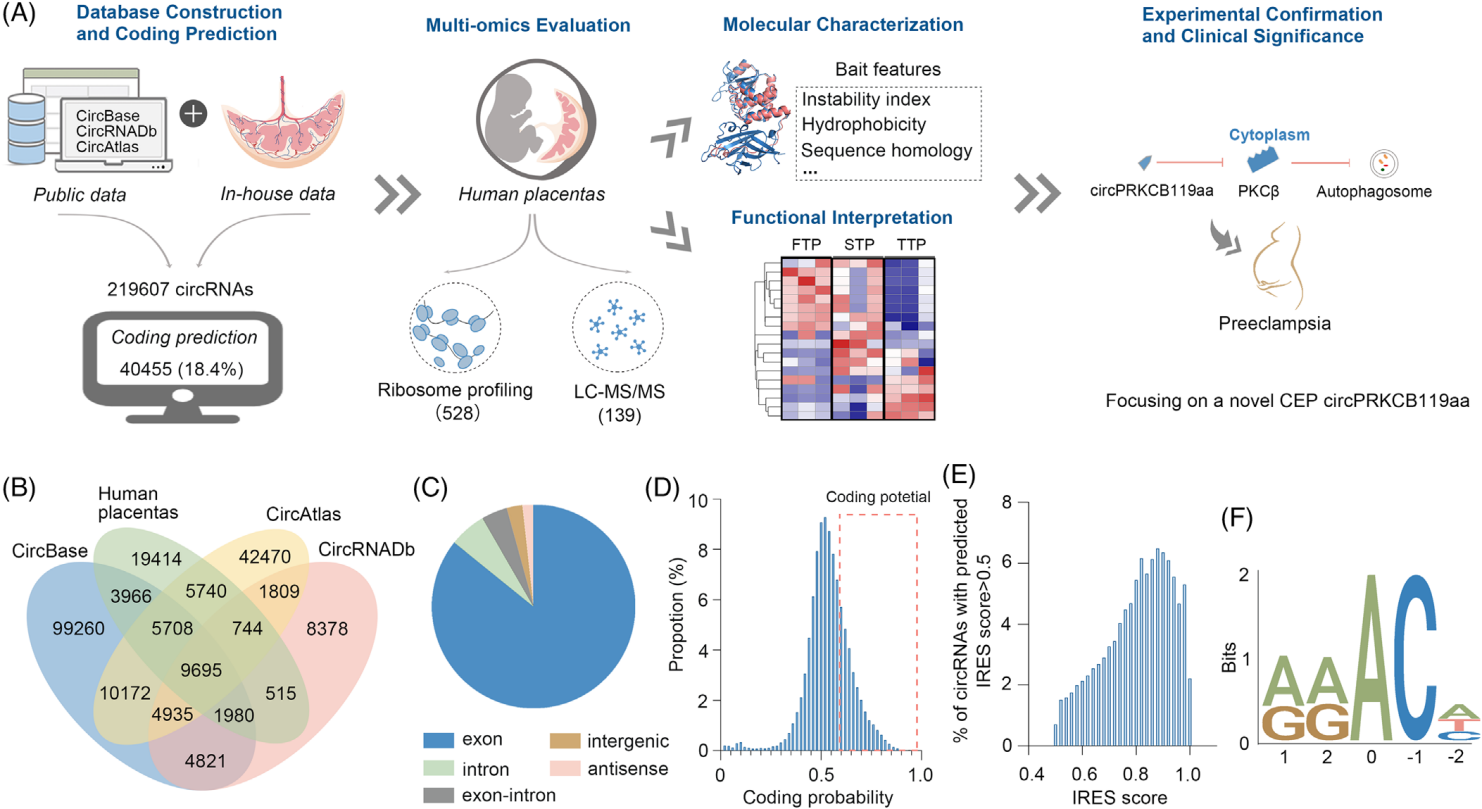
Endogenous circRNAs have translational potential. (A) Workflow of this study. The numbers inside parentheses within the second panel represent the count of coding circRNAs identified by the indicated methods. (B) Venn plot showing overlapping circRNAs obtained from sequencing results and published repositories. (C) Percentage of 219 607 circRNA derivation from DNA sequences. (D) Distribution of coding probability of 158 747 circRNAs with predicted translation capacity. The box indicates the defined range (coding probability > 60%) of coding potential. (E) Distribution of predicted IRES scores in circRNAs. Only circRNAs with an IRES sequence predicted to have a score > 0.5 are shown. (F) RRACH motif analysis of circRNAs. See also Figure S1.

In this study, we identified hundreds of CEPs in the human placenta by in silico analysis and multiomics approaches. These CEPs are likely involved in placental function on the basis of their bait features. Furthermore, we experimentally established a pathological role of the novel CEP circPRKCB119aa in preeclampsia (Figure 1A).

## 2 MATERIALS AND METHODS

### 2.1 Placental tissue collection

Three placentas from the first trimester (6–7 weeks), second trimester (18–20 weeks) and third trimester (38–40 weeks), respectively (Table S1), were collected for CEP identification. Specifically, placental villous tissues from the first trimester were obtained from healthy women with ultrasound‐dated viable singleton pregnancies who underwent elective social termination of pregnancy between 6–7 weeks of gestation. Normal second trimester placentas were obtained from healthy women undergoing medical termination of pregnancy for psychosocial reasons. Term placentas were collected from three women with uncomplicated pregnancies delivered via caesarean section. Gestational age was determined by ultrasound measurement of the embryo's crown‐rump length at early pregnancy. No pregnancy complication was diagnosed for all participants before termination. The CEP expression in each sample from the three trimesters was individually evaluated using ribosome profiling and LC‐MS/MS.

Additional placental tissues were collected from pregnant women presenting with preeclampsia (*n* = 30) and from normotensive controls (*n* = 30) to compare the levels of *circPRKCB* and its translational products between groups. The clinical characteristics between groups have been described in our previous study,^26^ and are provided in the Supplementary Materials (Table S2).

Preeclampsia was defined as blood pressures ≥140/90 mmHg on two or more consecutive measurements after 20 weeks of gestation, with or without maternal proteinuria (≥300 mg/24 h). Severe preeclampsia was diagnosed in the presence of significantly higher blood pressure levels (≥160 mmHg systolic or ≥110 mmHg diastolic) concomitant with signs of organ dysfunction. Patients with chronic hypertension, chronic kidney disease or any known fetal anomalies were excluded. Written informed consent was obtained from all patients, and the study was approved by the Ethics Committee of Obstetrics and Gynecology Hospital of Fudan University (Ethics approval number: 2017−57).

All samples were collected following standard procedures. Briefly, placental samples from the second trimester and term were obtained from the placental disc located 3–5 cm from the umbilical cord insertion site, measuring approximately 1 cm^3^ in size. After removing the maternal and fetal surfaces, the samples underwent a thorough wash with PBS. Subsequently, the samples were dissected into smaller pieces, ensuring the careful exclusion of large vessels during the preparation process. Within 30 min of collection, the samples were allocated to appropriate storage containers based on their intended purposes. For protein examination, samples were immediately snap‐frozen in liquid nitrogen and stored at −80°C. For RNA preservation, samples were immersed in RNAlater, initially stored at 4°C overnight, and subsequently transferred to the −80°C freezer. For immunohistochemical experiments, samples were fixed in tubes containing polyformaldehyde. The collection methodology for placental villous tissues followed a similar approach as described for term placenta, with the exception that it only required thorough blood cleansing before being allocated to designated tubes for their intended purposes.

### 2.2 RNA library construction and sequencing

Total RNA was extracted from placental samples using TRIZOL. The concentration and quality of the RNA were assessed using a NanoDrop ND‐1000 spectrophotometer, while RNA integrity was evaluated using an Agilent 2100 instrument with a RIN value exceeding 7.0. The enrichment of circRNAs involved initial depletion of ribosomal RNAs, followed by the elimination of linear RNAs through treatment with RNase R. The circRNAs were fragmented, reverse‐transcribed into cDNAs, and further synthesised into U‐labelled second‐stranded DNAs. Subsequently, the cDNA blunt ends were ligated with A‐tails and indexed adapters. Following size selection of the library using AMPureXP beads (Beckman), the ligated cDNA products underwent incubation with a heat‐labile UDG enzyme to remove the second‐strand cDNA. The first‐strand cDNA was PCR amplified to generate the library, which was sequenced on an Illumina Hiseq 4000 platform (LC Bio, Hangzhou) in a 150 bp paired‐end sequencing mode. The sequence data has been deposited in Gene Expression Omnibus (GEO: GSE196032).

### 2.3 CircRNA detection and full‐length circRNA assembly

For raw data obtained from placental sequencing and downloaded from CircAtlas,^27^ full‐length circRNA assembly was performed as following. The low quality reads and those with adaptor contamination or undetermined bases were removed using Cutadapt,^28^ and the sequence quality was estimated by FastQC. The reads were then mapped to the human genome using Bowtie2^29^ and TopHat2,^30^ and the reads spanning circRNA back‐splicing junctions in the unmapped reads were identified by Tophat‐Fusion.^31^ The CIRCexplorer2^32^ and CIRI^33^ pipelines were used with default parameters for de novo circRNA assembly, and back splicing reads were detected in unmapped reads by Tophat‐Fusion.^31^

### 2.4 Ribosome profiling

Ribosome profiling was conducted following previously established protocols^34^ with minor adaptations. Briefly, placenta tissues representing the first, second and third trimesters were promptly frozen in liquid nitrogen and subsequently pulverised using a mortar and pestle. The resulting powder was homogenised in 400 µL of lysis buffer for 10 min on ice, and then subjected to multiple passages through a syringe equipped with a 26‐G needle. The homogenised lysate was subjected to centrifugation at 20 000 × *g* for 10 min to eliminate debris, and the resulting supernatant was harvested. In the generation of ribosome footprints (RFs), the supernatant was treated with 10 µL of RNase I and 6 µL of DNase I (NEB) for 45 min. The solution was then applied to size exclusion columns that had been preequilibrated with 3 mL of polysome buffer, and the eluate was obtained through centrifugation at 600 g for 4 min. Following this, the eluate was supplemented with 10 µL of 10% (wt/vol) SDS, and the RFs were extracted utilising the RNA Clean and Concentrator‐25 kit. The removal of ribosomal RNAs (rRNAs) was carried out following established procedures.^35^ Subsequently, the RFs were purified employing magnetic beads. The construction of the Ribo‐seq library using the purified RFs was achieved utilising the NEBNext® Small RNA Library Prep Set for Illumina®. Finally, sequencing was conducted on the Illumina HiSeqTM X10 platform.

### 2.5 CEP prediction

CEP prediction was performed as previously described with some modifications.^5^ In brief, we first generated a comprehensive circRNA sequence compendium by integrating publicly available data sources^27^, ^36^, ^37^ with our own sequencing data from human placenta, resulting in a final total of 219 607 circRNAs obtained. Then, each circRNA sequence was replicated four times and subjected to ORF prediction using the ORF finder tool, with ATG and other alternative initiation codons utilised as start codons. All putative ORFs spanning the circRNA junction were retained. In cases where there were inclusion relationships between ORFs, preference was given to the longest one. The ORFs were converted into amino acid sequences for MS searches, and to prevent false‐positive results, we also generated a simulated pool of random amino acid sequences with similar lengths.

### 2.6 Protein extraction and trypsin digestion

Placental tissue aliquots (500 µg) were frozen in liquid nitrogen and pulverised using a mortar and pestle. The powdered tissue was then lysed with a 10‐fold volume of lysis buffer (8 M Urea, 1% SDS) containing protease inhibitors (Thermo Scientific) on ice for 30 min. Subsequently, the supernatant was collected as the whole tissue extract after centrifugation at 16 000 × *g* for 30 min. The protein content was determined using the BCA Protein Assay Kit. Each sample containing 100 µg of protein was reduced with 10 mM Tris (2‐carboxyethyl) phosphine (TCEP) at 37°C for 60 min, followed by alkylation with 40 mM iodoacetamide at room temperature in the dark for an additional 40 min. The proteins were then precipitated at −20°C for 4 h by adding precooled acetone and subsequent centrifugation at 10 000 × *g* for 20 min. The resulting pellet was dissolved in 100 µL of 100 mM tetraethylammonium bromide (TEAB) and was proteolysed with 1 µg of trypsin per 25 µg of protein, followed by overnight incubation at 37°C.

Furthermore, the resultant peptide mixtures were acidified by the addition of trifluoroacetic acid (TFA) to achieve a final concentration of 2%, and the supernatants were subsequently transferred to reversed‐phase C 18 Sep‐Pak cartridges for desalting and concentration purposes. Preparations of the Sep‐Pak cartridges involved initial washing steps with methanol and 0.1% TFA prior to the introduction of the peptide mixtures. Subsequently, elution of the peptides from the Sep‐Pak cartridges was carried out using 40% acetonitrile (600 µL) and 60% acetonitrile (600 µL), followed by drying in a SpeedVac centrifuge.

The peptide mixture was reconstituted in buffer A and subjected to high pH separation utilising an Aquity UPLC System coupled with a reverse‐phase column. The high pH separation was conducted through a linear gradient elution. The gradient program spanned over 60 min with the following conditions: 1–4% B in 2 min; 4%–35% B in 43 min; 35%–55% B in 5 min; 55%–95% B in 3 min; 95% B for 2 min; 95%–1% B in 1 min; and 1% B for 4 min. The eluate was fractionated every 1.5 min, yielding 30 fractions from 3 to 48 min. A concatenation strategy was employed to combine these 30 fractions into 15 fractions. Finally, the 15 fractions were dried using vacuum centrifugation for subsequent proteome analysis.

### 2.7 Liquid chromatography tandem mass spectrometry (LC‐MS/MS)

Peptides were subjected to fractionation using the High pH Reversed‐Phase Peptide Fractionation Kit. The peptide mixture, after drying, was reconstituted and acidified with a 0.1% TFA solution before being loaded onto the high‐pH reversed‐phase fractionation spin column that had been preequilibrated. Subsequent elution of the bound peptides into 15 distinct fractions was carried out employing a step gradient of increasing concentrations of acetonitrile in a volatile high‐pH elution solution, with each fraction then subjected to concentration via vacuum centrifugation. LC‐MS/MS analysis of the fractions was executed utilising an Orbitrap Fusion mass spectrometer coupled with an Easy nLC1000 for a duration of 120 min. The mass spectrometer was operated in positive ion mode to acquire MS data in a data‐dependent manner within a 3‐s top speed, with the survey scan covering the 350 to 1550 *m*/*z* range for HCD fragmentation analysis.

### 2.8 MS data search

We searched placenta proteomic data obtained from three first‐, second‐ and third‐trimester placenta tissues, respectively, using Mascot algorithm in Proteome Discoverer 1.3 platform and MaxQuant software (Thermo Fisher Scientific). Two independent searches were performed, one to identify canonical protein sequence in the UniProt database,^38^ and the other to detect the putative protein sequence translated from circRNAs. The circRNAs‐derived peptides were defined based on the following criteria: (i) the sequences cannot be retrieved from UniProt database, and (ii) the derived protein sequence spans the back‐splice junction. The searching parameters were set based on previous studies.^12^, ^39^ The statistical filtering of the peptide level was set to 5% FDR, and the protein FDR was excluded, since only a limited number of unique peptides can be theoretically identified from proteins with short sequences, which can overestimate the protein FDR in large proteomics datasets.^12^, ^40^, ^41^ To avoid the noise that may be interpreted as evidence for circRNAs translation,^42^ we constructed and searched a simulated pool of random amino acid sequences, which shares similarity of peptide size with those derived from ribosome‐associated circRNAs.

### 2.9 Parallel reaction monitoring (PRM)

The peptide validation were performed using parallel reaction monitoring (PRM), a targeted quantification method employing Orbitrap MS technology. The labelled peptide DSPSAPVNVTVR (the last V in the sequence is heavy isotope labelling) were utilised as spiked‐in standards and mixed with equal amount of tryptic digested lysates. The mixed peptide segments were subjected to chromatography for fraction elution, with various elution gradients set at 3%, 6%, 9%, 12%, 15%, 18%, 21%, 25% and 35%B. Subsequently, the samples were amalgamated into three fractions through cross‐merging and vacuum‐dried for future applications. The PRM analysis was performed on Q ExactiveTM HF‐X mass spectrometer (Thermo Fisher Scientific), equipped with a Nanospray Flex™(ESI) ion source, a spray voltage of 2.1 kV, and an ion transport capillary temperature of 320°C. During PRM MS data acquisition, the full scan resolution was set at 60 000 (at 200 *m*/*z*), with a C‐trap capacity of 3×10^6^, maximum ion injection time of 20 ms, and a normalised collision energy of 27%. Subsequently, the PRM data were processed using the Skyline software, and the peak area was normalised using an internal standard peptide. The raw MS data for PRM is publicly accessible in Placental Microprotein Bank (PMB, http://microprotein.cn/).

### 2.10 Calculating physicochemical values of circRNA‐encoded and canonical proteins

The amino acid count, theoretical isoelectric point (pI), instability index and grand average of hydropathicity (GRAVY) values for putative CEPs and the canonical proteins annotated in UniProt database were determined using the ExPASy website.^43^

### 2.11 Pairwise protein structure alignment

To compare the 3D structures of CEPs and their cognate LEPs, the sequences of the former were loaded onto the I‐TASSER workspace to create the predicted tertiary structures based on homology‐modelling methods. The tertiary structures of the cognate proteins were retrieved from Protein Data Bank (PDB) or predicted by AlphaFold Protein Structure Database,^44^ and then aligned with that of the CEPs using the protein structure alignment tool. PyMol (https://pymol.org/2/) was used to display the consistent helices and sheets between molecules. The alignment of superimposed proteins was evaluated by calculating the RMSD (Root Mean Square Deviation). A larger RMSD indicated greater distance between the paired structures.

### 2.12 Prediction of cellular localisation of CEPs

SignalP‐5.0^45^ was run with default parameters in a ‘Eukarya’ mode to predict the presence of signal peptides and the location of their cleavage sites in the putative CEPs. TMHMM‐2.0^46^ was used to predict transmembrane helices on the same set of proteins with default parameters.

DeepLoc‐1.0^47^ provides differentiation among 10 specific subcellular localisations. The prediction methodology relies on a neural networks algorithm that has been trained on proteins in Uniprot database with experimentally validated subcellular localisation information, using only the sequence data. In this investigation, the DeepLoc‐1.0 tool was employed to forecast the subcellular localisation of the CEPs, with the exclusion of plant‐specific chloroplasts.

### 2.13 Calculation of pathway enrichment score

Gene set variation analysis (GSVA) was performed using the R/Bioconductor package GSVA to calculate the pathway enrichment score of each sample. The hallmark gene sets and KEGG subset of canonical pathways gene sets used in this analysis were obtained from the MSigDB database v7.5.1.

### 2.14 Plasmids

To verify *circPRKCB* translation in trophoblasts, we cloned different *circPRKCB* isoforms into expression vectors. Sequences for the wild‐type *circPRKCB*, FLAG‐tagged isoform and isoform with the mutated ORF initiation codon were chemically synthesised, and cloned into the MluI/SalI sites of the pLV‐circRNA‐Hygro plasmid (HarO life, Shanghai, China). The linearised ORF expanding in *circPRKCB* was cloned into the pGMLV‐CMV‐MCS‐PGK vector (Genomeditech) at the XhoI and BamHI sites. In addition, the potential wild‐type and mutant *circPRKCB* IRES were cloned into the pcircIRES‐dReporter plasmid (HarO life, Shanghai, China) reconstructed from the psicheck2 vector. All constructs were Sanger sequenced and the cloned sequences were aligned against the human reference genome.

### 2.15 Lentiviral construction and transfection

Cells overexpressing specific genes were established by lentivirus transductions. The lentiviral plasmids were co‐transfected into 293T cells using Lipofectamine 3000 according to the manufacturers’ protocols. The lentiviral particles were transduced into the respective cell lines using polybrene (12 µg/mL, Hanbio Co. LTD, Shanghai, China) overnight. Subsequently, the transduced cells were treated with puromycin (10 µg/mL, Yeasen Biotech., Shanghai, China) or hygromycin B (100 µg/mL, Roche, Basel, Switzerland) as needed for selection.

### 2.16 Cell culture

In this investigation, the cell lines HTR‐8/SVneo, JEG‐3, HUVEC and 293T were utilised. The JEG‐3 and 293T cell lines were procured from the Type Culture Collection of the Chinese Academy of Sciences, whereas the HTR‐8/SVneo and HUVEC cell lines originated from the American Type Culture Collection. The JEG‐3, HUVEC and 293T cell lines were routinely maintained in Dulbecco's modified Eagle's medium (DMEM, Biological Industries), while the HTR‐8/SVneo cells were cultured in RPMI 1640 medium (Biological Industries). Cell preservation was conducted using cell‐saving solutions (New Cell & Molecular Biotech) at −80°C.

### 2.17 RNase‐R treatment and qRT‐PCR

The RNase‐R treatment involved adding 2U of RNase‐R (Epicenter Biotechnologies) per microgram of RNA, followed by incubation at 37°C for 30 min. RNA, with or without RNase‐R treatment, was reverse‐transcribed into cDNA. Subsequently, qRT‐PCR was performed on the Step One Plus Real‐Time PCR System (Applied Biosystems). The relative expression levels of the target genes were evaluated using the 2^−∆Ct^ method and normalised to the expression levels of β‐actin. Primer sequences utilised can be found in Table S3.

### 2.18 Nuclear mass separation assay

The nuclear and cytoplasmic fractions of trophoblast were extracted using a PARIS™ Kit (Life Technologies). Briefly, the nucleus and cytoplasm were lysed using cell fractionation buffer or/and cell disruption buffer to separate their contents. RNA isolation from the nucleus and cytoplasm was performed using a lysis/binding solution. The subsequent steps for qRT‐PCR analysis are similar as described above.

### 2.19 Antibodies

Anti‐β‐actin (60008‐1‐Ig, 1:2000 dilution for Western blotting unless otherwise indicated) and anti‐FLAG tag (20543‐1‐AP, 1:1000) antibodies were purchased from Proteintech Group (Wuhan, China). Antibody against LC3 (L8918, 1:1000 for Western blotting and 1:50 for immunofluorescence) was purchased from Sigma‐Aldrich. Antibodies targeting PKCβ1 (ab195039, 1:1000), PKCβ2 (ab32026, 1:1000), phospho‐PKCβ1^Thr642^ (ab75657, 1:1000), PKCα (ab11723, 1:1000) and PKCγ (ab108961, 1:1000) were purchased from Abcam. Anti‐p62 antibody (sc‐28359, 1:1000) was purchased from Santa Cruz, and anti‐phospho‐PKCβII^Ser660^ (9371, 1:1000) from Cell Signaling Technology. The polyclonal antibody against circPRKCB119aa (1:1000 for Western blotting and 1:50 for immunohistochemical staining) was obtained by injecting rabbit with the specific polypeptide (TSRPTSTGTSSLC) and purified by antigen affinity chromatography.

### 2.20 Immunoprecipitation (IP) assay

To investigate the specific interaction between circPRKCB119aa and the generated antibody, we performed immunoprecipitation assays in HTR‐8/SVneo cells transfected with either circular or linear forms of *circPRKCB* ORF. IP assays were conducted according to established protocols.^48^ A single confluent 10 cm dish of HTR‐8/SVneo cells was lysed using ice‐cold IP buffer supplemented with a protease inhibitor. The cell lysates were subsequently centrifuged at 13 000 × *g* for 15 min, followed by overnight incubation with antibodies and Protein G‐agarose (Roche) at 4°C. Normal IgG (Santa Cruz) was used as a negative control in the experiments. The immunocomplexes were washed with cold IP buffer, collected and subsequently subjected to immunoblotting using circPRKCB119aa antibody and horseradish peroxidase (HRP)‐linked secondary antibody.

### 2.21 Cell migration and invasion assay

In vitro migration and invasion of trophoblast cells were assessed by the transwell assay. Briefly, the cell culture inserts (Corning, NY, USA) of pore size 8 µm were placed in 24‐well plates for the migration assay and additionally coated with Matrigel (Corning) for the invasion assay. Cells were plated in the upper chambers in serum‐free media at a density ranging from 1 to 3×10^5^ cells per well, with the lower chambers containing complete media. After a 24‐h incubation period for HTR‐8/SVneo cells or a 48‐h incubation period for JEG‐3 cells, the cells remaining on the upper surface were removed using a cotton swab. The invaded or migrated cells were fixed, stained, and quantified in three randomly selected fields.

### 2.22 Cell viability assays

For the CCK‐8 assay, cells of each group were seeded at a density of 3–5×10^3^/100 µL/well in 96‐well plates (Hangzhou Xinyou Biotechnology). At each stipulated time point, 110 µL sterile CCK‐8 solution was added to each well, and the cells were incubated for 3 h. The optical density (OD) was measured using a microplate reader at 450 nm.

In the colony formation assay, cells were seeded in 6‐well plates at a density of 500 cells per well and cultured for a period of 10 days. Subsequently, the formed colonies were fixed with 4% paraformaldehyde for 30 min, stained with 0.5% crystal violet for 15 min, and quantified using ImageJ software.

### 2.23 Immunofluorescence

The presence and number of LC3 puncta were determined by the immunofluorescence assay. The suitably transduced cells were grown on coverslips for 24 h and then fixed with 4% paraformaldehyde. The fixed cells were permeabilised with PBST buffer for 10 min, and then blocked with 5% bovine serum albumin (BSA) in PBST. After incubating overnight with anti‐LC3 antibody at 4°C, the cells were probed with fluorescently‐labelled secondary antibodies for 1 h at room temperature in the dark. The cells were then counterstained with DAPI, and viewed under a fluorescence microscope (Thermo Fisher Scientific).

### 2.24 Autophagosome quantification

The quantification of autophagosomes was performed following previously reported methods with slight modifications.^49^ In brief, using a combination of automated and manual parameter adjustments in ImageJ software, we labelled and counted the LC3 puncta. For autophagosome clusters, we estimated the LC3 puncta by dividing the total area by the area occupied by a single autophagosome. Given the tendency of JEG‐3 cells to aggregate, autophagosomes were counted per cell by dividing the total count by the number of nuclei. Three clusters of JEG‐3 cells or twenty individual HTR‐8/SVneo cells per group were randomly selected for analysis. The quantification was performed in triplicate by different researchers.

### 2.25 Animal models of preeclampsia

We obtained adult male and female C57BL/6J mice from Jiesijie Laboratory in Shanghai, in accordance with the animal care and use guidelines established by Fudan University and approved by its Ethics Committee. The mice were kept in a controlled environment with regulated temperature and humidity, and were given unrestricted access to standard chow and water.

Before the commencement of the experiments, a 1‐week acclimatisation period was provided for all animals. Pregnancy was ascertained by the presence of a copulatory plug on the subsequent morning, referred to as embryonic day E0.5. NG‐Nitro arginine methyl ester (L‐NAME, 50 mg/kg/day, Sigma‐Aldrich) or PBS was administered daily via subcutaneous injection from E7.5 to E14.5 of pregnancy, which aligns with the early second to third trimester in human gestation. To assess the manifestation of preeclampsia symptoms in the animals, blood pressure was monitored utilising the Visitech System BP2000 (Apex), while 24‐h urine samples were collected in metabolic cages and quantified using a Bradford Protein Assay Kit.

### 2.26 Polymerase chain reaction (PCR)

The PCR reaction mixture was prepared in a final volume of 50 µL, comprising 25 µL of 2×Hieff® PCR Master Mix (Yeasen Biotech), 19 µL of nuclease‐free water, 2 µL each of 1 µM forward and reverse primers and 2 µL of cDNA or gDNA serving as the template. Subsequently, the PCR reaction mixture was briefly centrifuged and thoroughly mixed. The PCR amplification was conducted using the Eppendorf Mastercycler.

### 2.27 Statistical analysis

All statistical analyses were performed with SPSS 20.0 (IBM, IL, USA). The normality of the distribution was assessed, and the cumulative distributions of two data sets were compared using the Kolmogorov–Smirnov test. Continuous variables between the two groups were compared utilising either a two‐tailed unpaired Student's *t*‐test or the Mann–Whitney *U* test, depending on the normality or nonnormality of the distribution, respectively. For normally distributed variables among multiple groups, one‐way analysis of variance (ANOVA) followed by Tukey's post hoc test was applied. In instances where the data did not follow a normal distribution, the Kruskal–Wallis test with Bonferroni correction was employed. The instability index between two categories of proteins was compared using the chi‐square test.

Binary logistic regression analysis was employed to explore the correlation between placental *circPRKCB* levels and the risk of preeclampsia by calculating both unadjusted and adjusted odds ratios (ORs). Pregestational body mass index (BMI), maternal age and gestational age were selected as potential confounders based on their established associations with preeclampsia or if they influenced the relationship between circPRKCB levels and preeclampsia by over 10%.^50^

## 3 RESULTS

### 3.1 Endogenous circRNAs have translational potential

As the first step to assess the coding potential of endogenous circRNAs, we generated a global compendium by combining public and newly sequencing data (Figure 1A). We mined the CircBase^36^ and CircRNADb^37^ databases and filtered the low‐quality sequence less than 10 nt in length. We also assembled de novo circRNA sequences from raw data in CircAtlas^27^ using the CIRCexplorer2^32^ and CIRI^33^ pipelines (see Section 2). This resulted in the identification of a total of 200 193 publicly available full‐length circRNAs (Figures 1A and B and S1A), excluding those specific to the placenta. Furthermore, to capture the unique placental circRNA profile, three trimesters of placental RNA were sequenced. The obtained reads were mapped to the human genome using Bowtie2^29^ and TopHat2,^30^ and the unmapped reads were subjected to Tophat‐Fusion^31^ to identify the circRNA back‐splicing junctions. A candidate circRNA was determined if it was supported by at least two independent back‐spliced reads and assembled by CIRCexplorer2^32^ and/or CIRI^33^ pipelines. A total of 47 762 circRNAs were identified (Figures 1B and S1A), with 19 414 not present in public databases and presumed to be placenta‐specific (Figure S1B), though further experimental validation is still required. Altogether, the integrated data contained 219 607 circRNAs after elimination of redundancy (Figure 1A and B), surpassing the counts reported in previous studies. This dataset was then utilised for further analysis.

To characterise these integrated circular transcripts, we first associated the circRNAs with the coding region of genes. As many as 85.8% of these circRNAs are derived from coding genes (Figure 1C), and 68.2% of the coding genes annotated in Ensembl database can be transcribed to at least one circRNA (Figure S1C). These statistics are consistent with a previous report on CircAtlas.^27^ In addition, the circRNA‐coding genes generate a greater diversity of circular transcripts compared to linear mRNAs (Figure S1D), and there is a correlation between the number of circRNAs and mRNAs derived from the same gene (Pearson's *R* = 0.217, *p* < .001, Figure S1E). This correlation can be attributed to common regulatory factors for alternative splicing that result in the circular and linear transcripts.

To evaluate the coding potential of the circRNAs, we predicted the translation‐associated parameters, including ORF, IRES and m^6^A‐modified sites, using bioinformatics tools. The presence of an ORF, usually spanning the splicing site, is a prerequisite for circRNA translation. Among the 219 607 circRNAs, 158 747 (72.3%) were predicted to have a coding probability by the Coding Potential Assessment Tool (CPAT),^51^ and 40 455 (18.4%) showed greater than 60%, which were considered as coding potential in this study (Figure 1A; see Figure 1D for the distribution of coding probability and Figure S1F and G for the distribution of Fickett and hexamer scores of potentially coding circRNAs). Since cap‐independent translation drivers^9^, ^13^ mediate circRNA translation, we then predicted whether IRES and m^6^A are widespread in the circular transcripts. IRES was predicted by IRESfinder, which is based on a logit model with 19 carefully selected framed *k*‐mer features.^52^ As shown in Figure 1E, more than 60% of the circRNAs had an IRES sequence with a predicted score > 0.5, of which approximately 20% scored more than 0.9. The m^6^A modification usually occurs in a consensus motif of RRACH, which can initiate circRNA translation.^9^ The motif (Figure 1F) exhibited an average frequency of 46 occurrences per circRNA sequence, suggesting the prevalence of m6A‐mediated translation, which requires experimental validation. Taken together, these data suggested that a substantial proportion of endogenous circRNAs have translation potential.

### 3.2 Landscape of translating circRNAs in the human placenta

**FIGURE 2:**
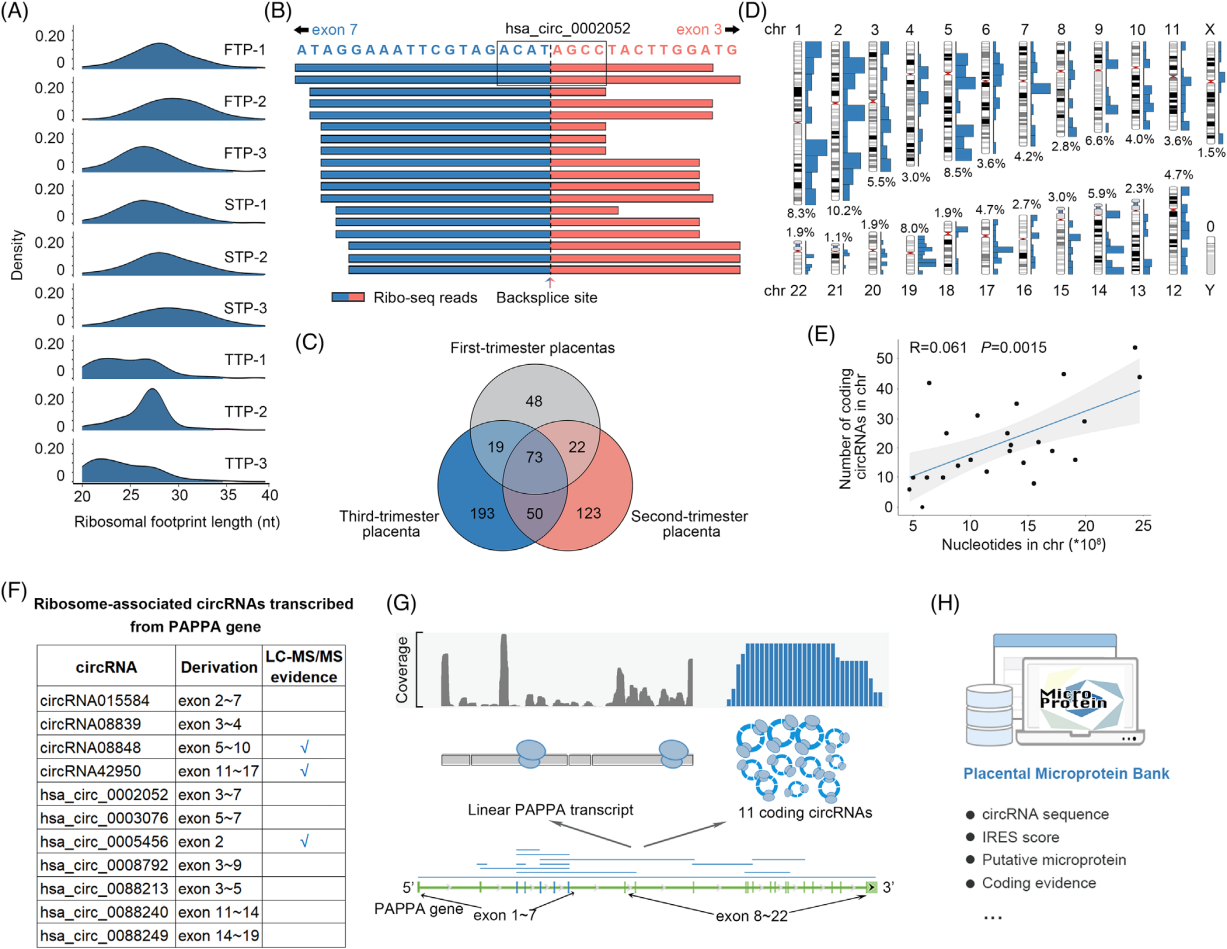
Landscape of translating circRNAs in human placenta. (A) Size distribution of ribosome footprint (RF) lengths across all placental samples. FTP, first‐trimester placenta; STP, second‐trimester placenta; TTP, third‐trimester placenta. (B) A typical example of eligible ribosome‐associated circRNA (hsa_circ_0002052). The box indicates the minimum requirements (i.e., 2 unique Ribo‐seq reads with 4‐nt overhang) for defining a ribosome‐associated circRNA. (C) Venn diagram showing the number of ribosome‐associated circRNAs identified in the first‐, second‐ and third‐trimester placentas. (D) Chromosomal coverage of the 528 placental coding circRNAs. Blue bars indicate the number of coding circRNAs in a chromosomal region. The percentages of coding circRNAs originating from each chromosome are indicated. (E) Correlation of the number of coding RNAs and nucleotides in chromosomes. The 95% confidence interval for the linear regression is represented by the grey area. (F) List of ribosome‐associated circRNAs transcribed by PAPPA gene. (G) Illustrative diagram depicting PAPPA gene transcription into linear and circular transcripts. (H) Overview of Placental Microprotein Bank (PMB). The PMB integrates the circRNAs with their coding potential, translation driver prediction, putative derived peptides and known coding evidence. See also Figure S2.

To determine the existence and abundance of translating circRNAs in the human placenta, we performed ribosome sequencing (Ribo‐seq) on the placental tissues from three different trimesters (Figure 1A). This resulted in approximately 20 million reads per sample after filtering for rRNAs, tRNAs, miRNAs, snRNAs and snoRNAs (Table S4). Ribo‐seq reads showed the expected size distribution that predominantly ranged from 25–35 nt (Figure 2A), and displayed the characteristic of 3‐nt codon periodicity (Figure S2A). In addition, these reads were largely restricted to the CDS when analysed for their distribution in the linear human genome (Figures S2B and S2C). These results suggested that most, if not all, Ribo‐seq reads detected in the experiment originate from bona fide ribosome footprints (RFs).

To identify coding circRNAs, we filtered the mapped Ribo‐seq reads to human reference genome. Only the unmapped reads that aligned to circRNA junctions (25 bp upstream and downstream of the back spliced site) were used for subsequent analyses. The ribosome‐associated circRNAs were defined as those with back‐splice junction covered by at least 2 unique Ribo‐seq reads with a minimum 4‐nt overhang. The typical ribosome‐associated circRNA is exemplified by hsa_circ_0002052; its junction was covered by 18 eligible Ribo‐seq reads (Figure 2B). Accordingly, Ribo‐seq analysis identified a total of 2634 reads that mapped to 476 back‐splice junctions of 528 distinct circRNAs (Table S5). The discrepancy in the numbers of junctions and circRNAs can be attributed to the occurrence of multiple translating circRNAs that share the same junction. We identified 162, 268, and 335 translating circRNAs in first‐, second‐, and third‐trimester placentas, respectively (Figure 2C). The translating circRNAs were distributed evenly across in all chromosomes with the exception of chromosome Y (Figure 2D), indicating their genome‐wide transcription pattern. Moreover, the amount of coding RNAs was proportional to the number of nucleotides in a given chromosome (Figure 2E). Interestingly, some genes expressed two or more coding circRNAs with various back‐splice junctions. For example, the PAPPA gene that encodes a syncytiotrophoblast‐derived metalloproteinase^53^ can be transcribed into 11 coding circular transcripts including the dominant ribosome‐dense circRNA (hsa_circ_0002052), whereas only one coding linear transcript is recorded in the Ensembl database (Figure 2F and G).

The CEPs were further verified by mass spectrometry. A major limitation in the identification of circRNA‐encoded proteomes is the lack of a predefined sequence for targeted peptides. To overcome this, we generated a database of putative CEPs that are currently not annotated in protein repositories. To ensure that the detected peptides originated exclusively from circRNAs, only those translated from the sequences spanning the hallmark circRNA regions (i.e., the back‐splice junctions) were taken into account (Figure S2D). Accordingly, 519 of the 528 ribosome‐associated circRNAs were predicted to produce at least one peptide longer than 10 amino acids (Table S6). As a result, the analysis ultimately yielded a total of 913 putative peptides.

To extensively identify peptides derived from translated circRNAs, the digested peptides from three trimesters of placental tissues were fractionated by a high pH reversed‐phase method before LC‐MS/MS, and the raw MS files were processed with MaxQuant and Mascot algorithms. After computational quality control, a total of 151 unique peptides corresponding to 139 ribosome‐associated circRNAs were identified (Table S7). To avoid the false‐positive detection of MS searches, we also constructed and searched a simulated pool of random amino acid sequences, and consequently obtained a negative result. Following this, we performed a Parallel Reaction Monitoring (PRM) assay, which is a highly sensitive and accurate targeted MS approach to detect the exact fragmentation patterns, to validate the our results and add the robustness. In 3 term placentas, we positively identify 32 out of 151 (21.2%) selected microproteins (Table S7).

Leveraging the multiomics data, we have established the Placental Microprotein Bank (**PMB**, http://microprotein.cn/), an accessible database of placental coding circRNAs and their encoded proteins. The PMB integrates the circRNAs with their coding potential, translation driver prediction, putative derived peptides and known coding evidence (Figure 2H), which can facilitate the discovery of novel circRNA‐derived proteins and disease markers. Collectively, our findings have revealed a hitherto unknown circRNA‐encoded proteome in human placenta.

### 3.3 The unique physicochemical properties confer baiting capabilities on CEPs

**FIGURE 3:**
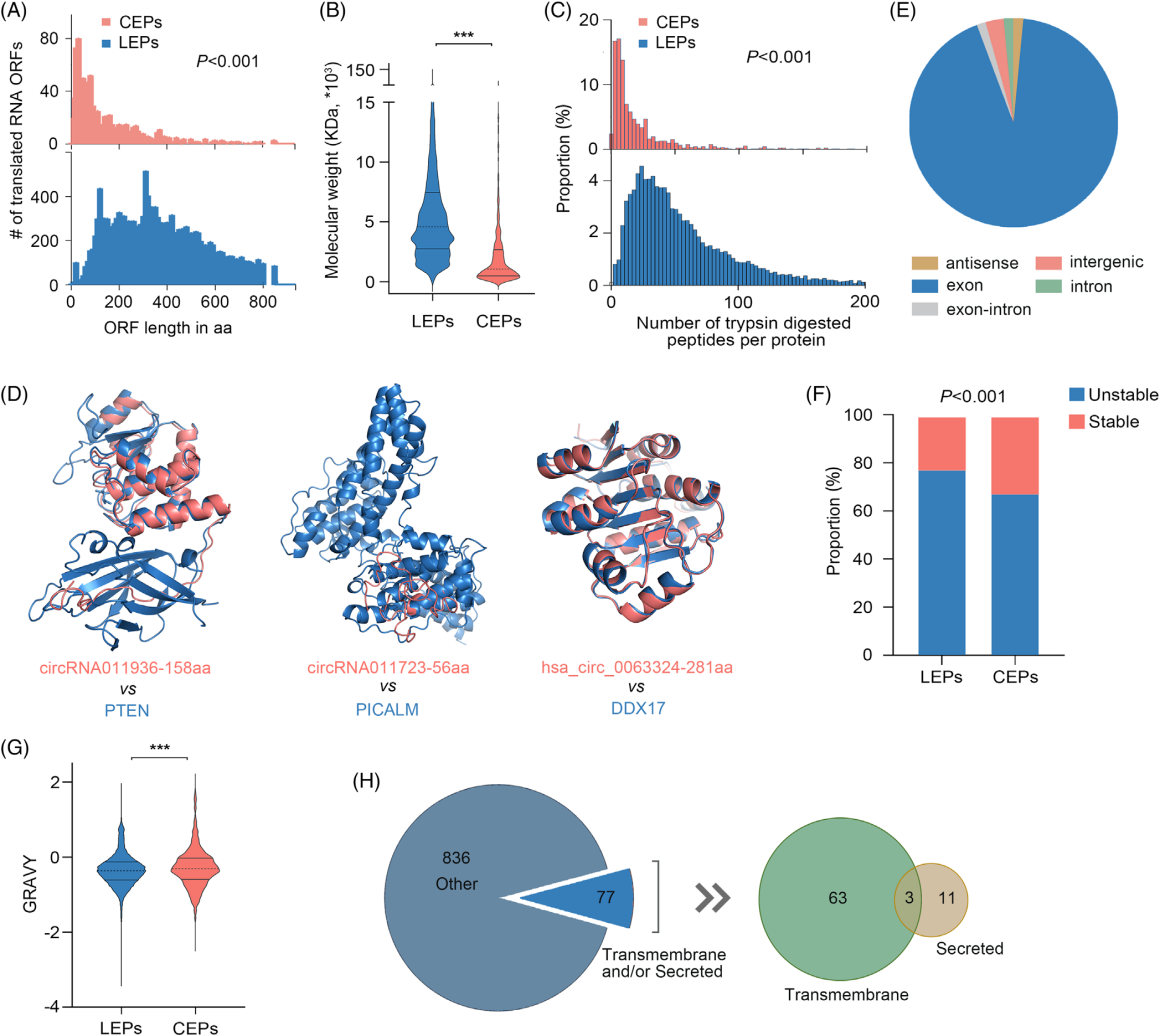
Analysis of the physicochemical properties and subcellular localisations of 913 putative CEPs. (A) The distribution of amino acid sequence lengths in LEPs annotated in the UniProt database and CEPs (*p *< .001, Kolmogorov–Smirnov test). (B) Comparison of the molecular weights of LEPs annotated in the UniProt database and CEPs (***p < .001, Mann–Whitney *U* test). (C) Distribution of the number of theoretical trypsin‐digested peptides per protein (*p *< .001, Kolmogorov–Smirnov test). (D) Three examples of highly superposed structures between CEPs (marked in red) and their cognate linear spliced isoforms (marked in blue). This significant alignment of three‐dimensional structures enables CEP to act as baits, competitively binding to the partners of cognate functional proteins, thereby indirectly inhibiting the function of the latter. PTEN, phosphatase and tensin homolog; PICALM, phosphatidylinositol binding clathrin assembly protein; DDX17, DEAD‐box helicase 17. (E) The proportion of origination of the newly identified protein‐coding circRNAs. (F) The proportion of stable and unstable proteins as per the instability index. The proteins are categorised as stable or unstable based on their instability index, with values below 40 and above 40 respectively (*p *< .001, chi‐square test). (G) Calculated grand average of hydropathicity index (GRAVY) of LEPs and CEPs. Positive values indicate hydrophobic and negative values indicate hydrophilic (***p < .001, Mann–Whitney *U* test). (H) Prediction of signal peptides and transmembrane helices in CEPs using SignalP 5.0 and TMHMM Server 2.0 respectively. See also Figures S3 and S4.

The CEPs act as baits that indirectly putting brakes on their cognate LEPs’ function by competitively binding to their common partners.^18^ To confirm that the newly recognised CEPs are likewise endowed with bait features, we analysed the physicochemical properties of the 913 putative CEPs by in silico methods. First, compared to the canonical proteins annotated in the UniProt database, the circRNA‐encoded counterparts are shorter (Figures 3A and S3A) and have a lower molecular weight (Figure 3B). This results in a reduction of trypsin‐cleaved peptides per CEP (Figure 3C), which makes their detection challenging. This difficulty arises from the fundamental role of trypsin cleavage as an essential step in protein digestion for shotgun proteomics. Second, a high homology between CEPs and their cognate LEPs was observed, as evidenced by the superposition of both primary (identity 58% ± 28%, Figure S3B) and three‐dimensional structures (see Figure 3D for examples), presumably because most protein‐coding circRNAs originate from the canonical coding exons (Figure 3E). These two characteristics of CEPs constitute the fundamental prerequisites that ensure bait‐mediated regulation to occur.^18^

Beyond that, these placental CEPs are also seemingly more stable than LEPs annotated in UniProt database (Figure 3F), which is further conducive to their function as baits, and can be partially explained by the fact that a greater percentage of CEPs are hydrophobic (Figure 3G). This is however inconsistent with the hypothesis put forth by Fan et al. that the peptides encoded by sequences across the back‐splicing junction result in unstable proteins.^54^ The theoretical isoelectric point (pI) of CEPs are comparable to that of LEPs (Figure S3C), possibly due to the considerable overlap of their amino acid sequences. In contrast, microproteins translated from long noncoding RNAs have significantly higher pI than classical proteins.^55^

The CEPs were then characterised in terms of subcellular localisation. We found that although the CEPs are ubiquitous, a large proportion (93%) was predicted to be located within the mitochondrion, extracellular space, cytoplasm and nucleus (Figure S3D). Our focus was primarily on the novel proteins involved in the maternal‐fetal crosstalk, since they may play key roles in maintaining pregnancy. The interplay is dependent on direct cell–cell contact by transmembrane molecules or indirect interactions via secreted molecules. As shown in Figure 3H, 77 of the 913 putative CEPs (8.43%) were predicted to have a transmembrane and/or secretory domain. Specifically, 63 proteins were predicted to be transmembrane, 11 secreted, and 3 both transmembrane and secreted (typical examples are listed in Figure S4A and B). The three dual‐feature proteins may be either secreted or localised in various subcellular compartments originating from the endoplasmic reticulum. Together, these data suggest that the unique characteristics may provide favourable properties for CEPs to potentially act as baits across diverse subcellular localisations.

### 3.4 The CEPs may be implicated in placental development and associated disorders

**FIGURE 4:**
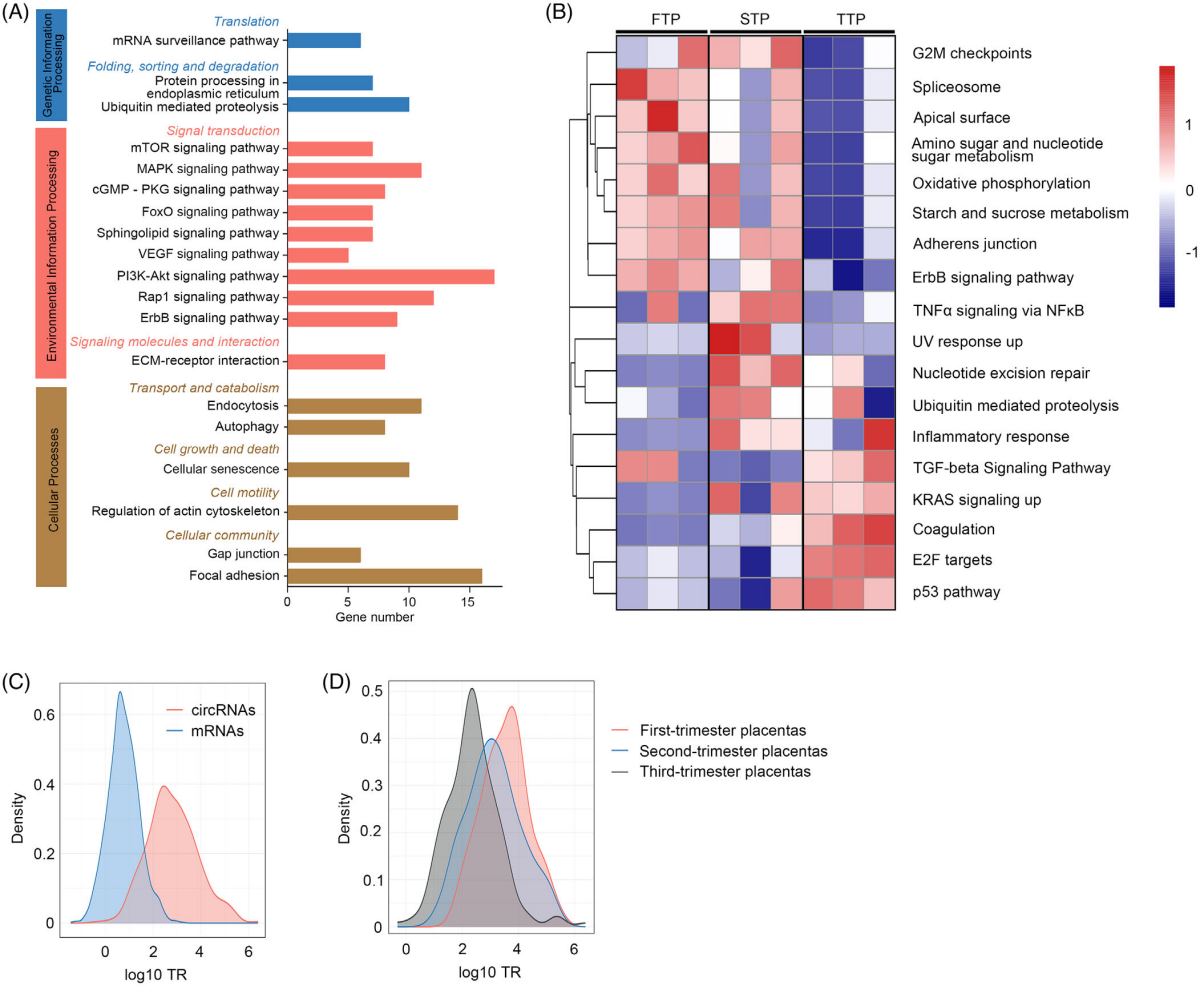
The CEPs may be implicated in placental development and associated disorders. (A) Top pathways identified from KEGG analysis of genes coding CEPs. (B) Heat map showing normalised pathway enrichment scores of CEPs from three trimesters of placental tissues. FTP, first‐trimester placenta; STP, second‐trimester placenta; TTP, third‐trimester placenta. (C) Distribution of the translation ratio (TR) of the coding circRNAs and mRNAs in human placentas. (D) Distribution of TR values of coding circRNAs in first‐, second‐ and third‐trimester placentas. See also Figure S5.

To investigate the biological roles of CEPs in placental function, we next performed the gene ontology and pathway analysis for genes of coding CEPs based on the bait principle. Enriched signatures were observed for processes involved in various developmental processes and responses, including biological regulation, metabolic processes, developmental processes and growth (Figure S5A). The pathway enrichment analysis revealed that mTOR, MAPK, VEGF, PI3K‐Akt, Rap1, autophagy and other pathways are overrepresented in CEPs (Figure 4A and Table S8). Some of these enriched biological processes and pathways have turned out to be causally associated with the known mechanism of placentation, as well as pathogenesis of placental diseases such as preeclampsia.^26^, ^56^ This underscores the importance of CEPs in terms of their biological significance.

To longitudinally examine the alterations in the CEPs‐associated pathways during placental development, we estimated the enrichment scores of pathway gene sets in each sample based on GSVA (see Section 2). Eighteen CEPs‐associated pathways were altered during pregnancy (Figure 4B). In the first‐trimester placentas, CEPs mediated energy and proliferation related pathways were exclusively upregulated, while placentas of the second‐trimester were characterised by elevated levels of inflammation‐related pathway, such as TNFα signalling via NFκB. In the third‐trimester placentas, CEPs‐mediated apoptosis‐related pathways, such as the p53 pathway, were predominantly enriched. The changes in CEPs‐mediated pathways along with the progression of pregnancy suggest the involvement of these novel proteins in placental development. Moreover, we screened for the differentially expressed coding circRNAs based on the ribosomal back‐splice reads using the DEseq2 package in R language and found seven junction‐unique coding circRNAs that were differentially expressed in the three trimesters (Figure S5B).

We also evaluated the relevant translation ratio (TR) that is defined as the ratio of the abundance of translating RNA to the total RNA. TR is a measure of translational efficiency and its alteration is associated with different cellular phenotypes.^55^, ^57^ Intriguingly, the TR of CEPs was significantly higher when compared to that of canonical proteins (Kolmogorov–Smirnov test, Figure 4C), and declined with the increase in gestational age (Kolmogorov–Smirnov test, Figure 4D). This indicated translating RNA may play an indispensable role in the development of the placenta. Overall, our analysis implied that CEPs have potential widespread and dynamic effects on placental development and disorders.

### 3.5 The *circPRKCB*‐encoded protein circPRKCB119aa functions as a bait to inhibit the cognate isoforms

**FIGURE 5:**
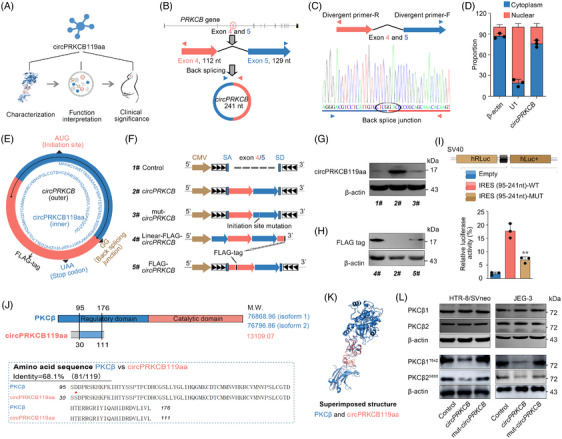
The circPRKCB‐encoded protein circPRKCB119aa functions as a bait to inhibit the cognate isoforms. (A) Workflow for exploring the biological plausibility of CEPs at the experimental level, illustrated using circPRKCB119aa as an example. (B) The annotated genomic region of *circPRKCB*. (C) Sanger‐sequencing validation of the specific back‐splice junction of *circPRKCB*. (D) The subcellular localisation of *circPRKCB* determined in HTR‐8/SVneo cells using a nuclear mass separation assay. (E) Diagram of the translation‐related elements in the *circPRKCB* sequence. The putative ORF from AUG (red) to UAA (blue), the IRES (double‐headed solid line), the back‐splicing junction (orange), and FLAG‐tag insertion site are indicated. The corresponding protein sequence is shown within the inner circle. (F) The schematic structures of various vectors for confirming the existence of circPRKCB119aa. The vectors (1#, 2#, 3# and 5#) expressing diverse *circPRKCB* isoforms were constructed using side flanking repeat sequences with splicing acceptor (SA) and donor (SD) that induce circular transcript formation. In vector 5#, the FLAG‐tag was inserted 49 nt downstream of the back‐splice site to avoid destroying the putative IRES (see also E). The linearised FLAG‐ORF was cloned into vector as a positive control (4#). Western blot analysis of circPRKCB119aa (G), and its FLAG‐tagged isoforms (H) in HTR‐8/svneo cells transduced with indicated lentiviral vectors. Lentiviral vectors are denoted by numbers 1#–5# and are annotated in (F). (I) Dual‐luciferase assay for evaluating the *circPRKCB* IRES activity. The upper panel shows schematic diagram of the luciferase bicistronic constructs. The wild‐type or mutated IRES was inserted between hRluc and hLuc reporter genes with independent initiation and stop codons. The lower panel shows the relative luciferase activity (hLuc/hRluc) in HTR‐8/svneo cells expressing the indicated constructs. **p < .01 vs. wild‐type IRES, one‐way ANOVA followed by Tukey's post hoc test. (J) Sequence alignment between PKCβ and circPRKCB119aa, revealing overlapping primary structures between residues 30–111 of circPRKCB119aa and the regulatory region of PKCβ spanning residues 95 to 176. M.W., molecular weight. (K) The superimposed structures (cross‐eyed stereo view) of circPRKCB119aa (red) and PKCβ (blue). (L) Western blot analysis of PKCβ isoforms in HTR‐8/svneo and JEG‐3 cell lines transduced with the indicated lentiviral vectors. See also Figures S6 and S7.

Having identified and characterised this novel set of proteins, we next explore their biological role at the experimental level with circPRKCB119aa as an example (Figure 5A). CircPRKCB119aa is translated from *CircPRKCB* (also called hsa_circ_0000682 and hsa_circ_15387 in other databases, Figure S6A).^36^, ^37^, ^58^ It was selected because its cognate linearly‐spliced isoform, protein kinase Cβ (PKCβ), has potential implications in the regulation of autophagy in preeclampsia, as recently reported.^26^ Based on the protein bait hypothesis,^18^ we surmised a reciprocal interplay between *circPRKCB‐*encoded protein and PKCβ. To test this hypothesis, we initially validated the circular nature of *circPRKCB* using the RNase R degradation assay (Figure S6B). Additionally, we verified that *circPRKCB* originates from the *PRKCB* gene through back‐splicing rather than DNA rearrangement, achieved by conducting PCR with divergent primer pairs flanking the back‐splice (Figures 5B and S6C) and Sanger sequencing (Figure 5C). *CircPRKCB* was predominantly located in the trophoblast cytoplasm, as determined by a nuclear mass separation assay in HTR‐8/SVneo cells (Figure 5D). This finding suggests that *circPRKCB* may have functional dependence on cytoplasmic elements.

Second, we demonstrated the translational capacity of *circPRKCB* by molecular approaches. A 360‐nt ORF spanning the back‐splice junction was identified, which potentially encodes a 119‐aa peptide that we termed circPRKCB119aa (Figure 5E). Based on the analysis of *circPRKCB* across distinct species, including macaca, mouse, dog, and rat, this putative protein shows a relatively high degree of conservation (Figure S6D and E). To determine its endogenous translation, we constructed vectors expressing wild‐type *circPRKCB* and initiation codon‐mutated isoform (mut*‐circPRKCB*) (Figure 5F), and generated a specific antibody against a unique sequence in circPRKCB119aa to detect the putative products in two trophoblast cell lines (HTR‐8/SVneo and JEG‐3). Both the wild‐type and mutated forms of *circPRKCB* were successfully overexpressed as confirmed by qRT‐PCR (Figure S6F), and the specific interaction between circPRKCB119aa and generated antibody was evaluated through IP assays (Figure S6G). Overexpression of *circPRKCB* upregulated circPRKCB119aa (Figures 5G and S6H), whereas mut*‐circPRKCB* failed (Figure 5G). Similar results were obtained when the *circPRKCB* sequence was tagged with FLAG (Figures 5H and S6I). Moreover, a 122 nt putative IRES was predicted in *circPRKCB* by IRESfinder^52^ and its translation‐driven capacity was validated by the dual‐luciferase assay (Figure 5I). These results suggest that *circPRKCB* can be translated in an IRES‐dependent manner.

Third, we examined whether circPRKCB119aa is equipped with bait features. *CircPRKCB* is transcribed from the fourth and fifth exons of *PRKCB* gene (Figure 5B), and the derived protein is shorter and has lower molecular weight than its cognate LEP PKCβ (Figure 5J). In addition, considerable degree of homology was also observed, as reflected by the primary (Figure 5J) and three‐dimensional (Figure 5K) structure overlaps between residues 30−111 of circPRKCB119aa and the regulatory region of PKCβ at residues 95–176. Therefore, these features indicate that circPRKCB119aa is eligible for action as a bait, supporting its potential involvement in PKCβ inhibition.

Given that PKCβ is activated through phosphorylation,^59^, ^60^ we investigated the impact of circPRKCB119aa on both PKCβ and the levels of phosphorylated PKCβ. PKCβ has two isoforms, namely PKCβ1 and PKCβ2. We initially confirmed the specificity of their respective antibodies to ensure there was no cross‐reactivity, even though the antibody datasheets had already indicated no cross‐reactivity between the two isoforms. Our findings revealed that both antibodies were able to detect the overexpression of their respective isoforms, whereas the corresponding antibodies were not (Figure S7A). Consistent with the bait principle, circPRKCB119aa significantly downregulated PKCβ1^thr‐642^ and PKCβ2^ser‐660^ (Figures 5L and S7B), while it had no impact on the mRNA and protein levels of both PKCβ variants (PKCβ1 and PKCβ2) (Figures 5L and S7C).

As PKCα and γ are classical PKC isoforms, similar to PKCβ, and circPRKCB119aa shares 91% and 81% homology with PKCα and γ, respectively, we further investigated the impact of circPRKCB119aa on the phosphorylation of PKCα and γ. The results demonstrated that circPRKCB119aa reduced the phosphorylation of PKCα and γ without affecting their protein levels (Figure S7D). Additionally, we examined the effect of circPRKCB119aa on the phosphorylation of Akt at Ser‐473 due to previous reports indicating that PKCβ can promote Akt phosphorylation. The overexpression of circPRKCB119aa was found to inhibit Akt phosphorylation at Ser‐473 (Figure S7E). These data suggested that the *circPRKCB‐*encoded protein circPRKCB119aa may function as a bait to inhibit the cognate isoforms.

### 3.6 CircPRKCB119aa enhances trophoblast autophagy

**FIGURE 6:**
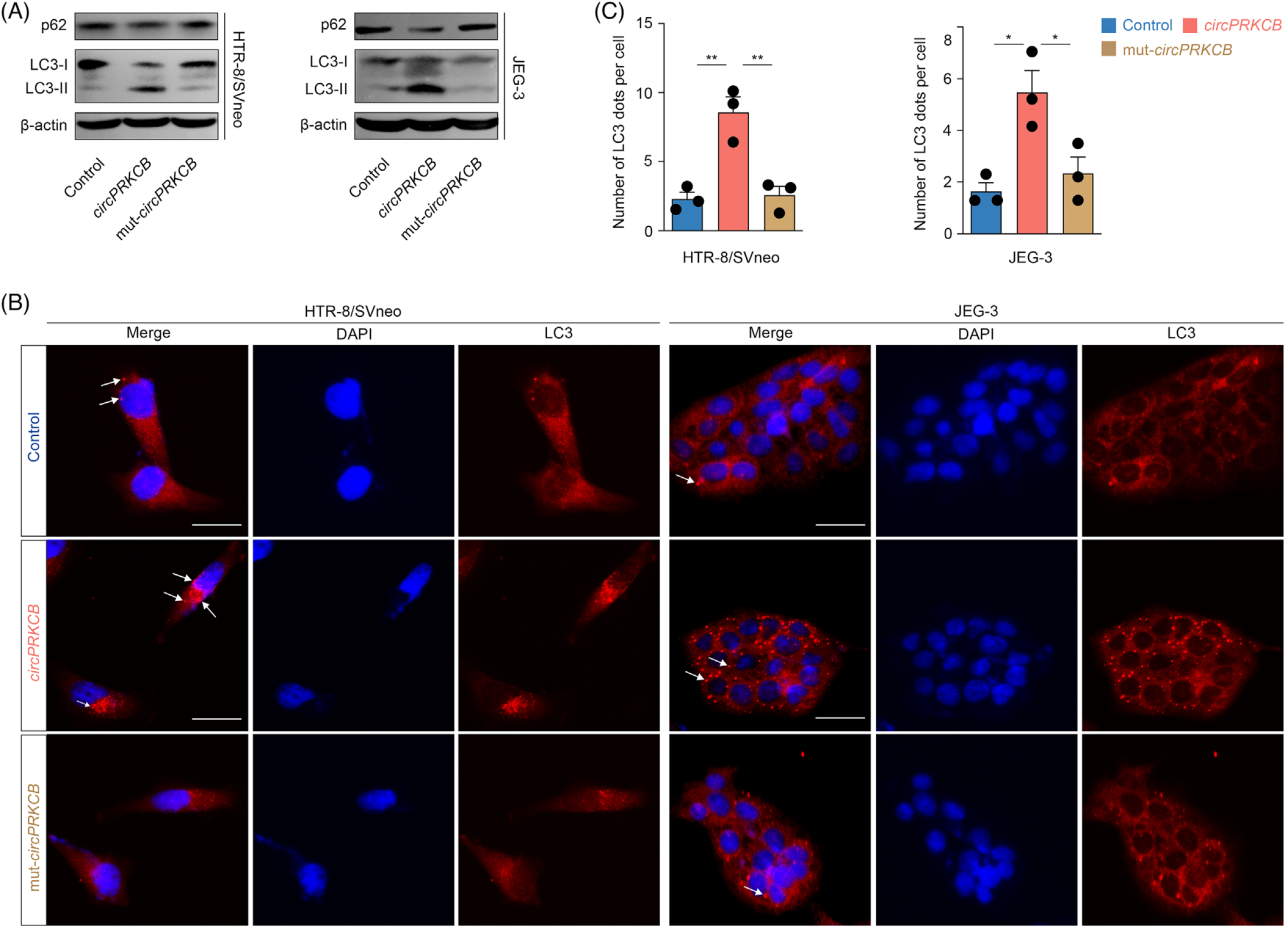
CircPRKCB119aa enhances trophoblast autophagy. (A) Western blot analysis of autophagic markers in HTR‐8/svneo and JEG‐3 cell lines transduced with the indicated lentiviral vectors. (B) Effects of circPRKCB119aa on autophagosome formation in HTR‐8/svneo and JEG‐3 cells. The cells were transduced with circPRKCB, mut‐*circPRKCB* and control lentiviral vectors, and representative immunofluorescence images are shown. The arrow indicates the autophagosome. Scale bars: 25 µm for HTR‐8/svneo and 50 µm for JEG‐3 cells. (C) The quantification of LC3 puncta in B. See also Figure S8.

Dysfunctional autophagy perturbs trophoblast homeostasis in the placentas of women with preeclampsia.^26^, ^61^ Since PKCβ inhibits placental autophagy in women with preeclampsia, we hypothesised that circPRKCB119aa has an antagonistic effect based on its bait features. During autophagy, the soluble form of LC3 (LC3‐I) is conjugated to phosphatidylethanolamine to form a nonsoluble and autophagic vesicle‐associated form (LC3‐II),^62^ while p62 is considered to be a substrate in autophagic degradation.^63^ Overexpression of *circPRKCB* significantly enhanced trophoblast autophagy, as indicated by the elevated LC3‐II/β‐actin ratio, promoted conversion of LC3‐I to LC3‐II, and downregulated p62, while mut‐*circPRKCB* with the disability of translation did not result in significant alterations in the levels of autophagy markers (Figures 6A and S8A). Consistent with this, immunofluorescence assay and transmission electron microscopy revealed a substantial increase in the autophagosomes in trophoblasts overexpressing *circPRKCB* but not mut*‐circPRKCB* (Figures 6B and C and S8B). These data demonstrate that circPRKCB119aa, rather than its transcript *circPRKCB*, enhances trophoblast autophagy.

To further ascertain whether autophagosome accumulation was due to enhanced autophagosome formation or blocked degradation, we examined the effects of circPRKCB119aa on autophagic flux using tandem fluorescent probe mRFP‐GFP‐LC3^64^ in two trophoblast cell lines of HTR‐8/SVneo and JEG‐3. In cells transfected with mRFP‐GFP‐LC3, the autophagosomes will be labelled with both GFP and RFP, whereas the acidic autolysosomes are only labelled with RFP due to GFP quenching in low pH.^64^ CircPRKCB119aa markedly promoted autophagosome formation but had a limited effect on the autolysosomes (Figure S8C). Blocking the fusion of autophagosomes and lysosomes with Bafilomycin A1 augmented autophagosome formation in all groups, whereas more autophagosomes were observed in cells overexpressing *circPRKCB* compared to the control and mut*‐circPRKCB* groups (Figure S8C), indicating that circPRKCB119aa induces autophagic flux by enhancing autophagosome formation. Taken together, circPRKCB119aa is an autophagy‐activating regulator that functions independently of its transcript structure.

### 3.7 CircPRKCB119aa demonstrates potential clinical significance in preeclampsia

**FIGURE 7:**
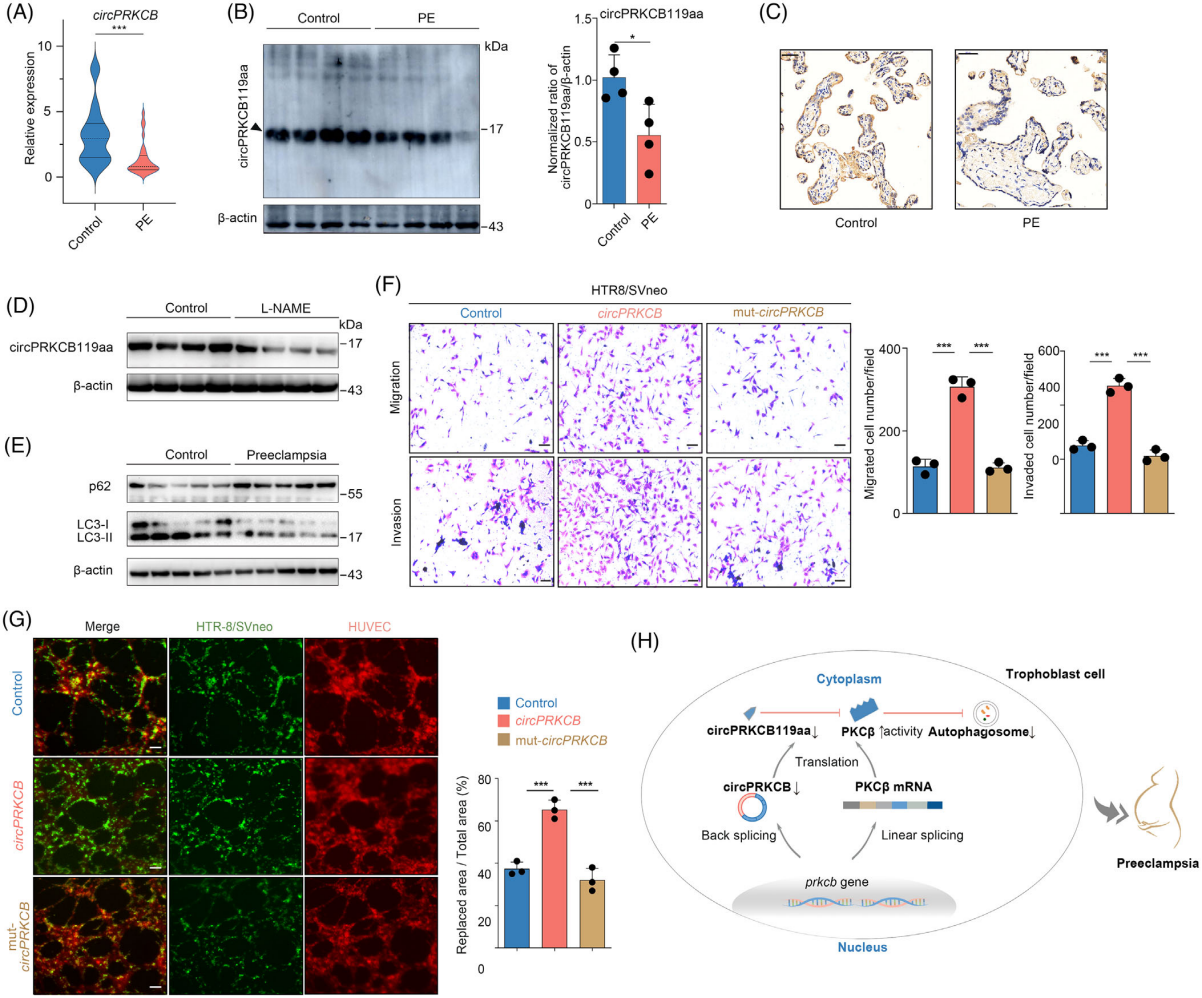
CircPRKCB119aa demonstrates potential clinical significance in preeclampsia. (A) Placental *circPRKCB* expression measured by qRT‐PCR in normotensive controls and preeclampsia patients. *n* = 30 samples per group. (B) Western blot analysis of circPRKCB119aa in placentas from normotensive and preeclampsia pregnancies. *n* = 4 randomly selected samples per group. Left panel, representative Western blot bands. Right panel, quantitative results determined by band densitometry relative to the control. (C) Representative immunohistochemistry images showing circPRKCB119aa expression and localisation in placentas from normotensive and preeclamptic women. Scale bars, 50 µm. (D) Western blot analysis of circPRKCB119aa in placentas from a preeclamptic mouse model treated with L‐NAME and control subjects. Four randomly selected samples were analysed per group. (E) Western blot analysis of autophagic markers in placentas from normotensive and preeclamptic pregnancies. Five randomly selected samples were analysed per group. (F) Effect of circPRKCB119aa on migration and invasion of HTR8/SVneo cells. The cells were transduced with circPRKCB, mut‐circPRKCB and control lentiviral vectors, and representative images are presented. The results are quantified in the right graph. Scale bars: 100 µm. (G) Impact of circPRKCB119aa on HTR‐8/SVneo cells replacing HUVECs in tube‐like structures for simulating vascular remodelling during gestation (right), and the quantification of replaced area proportion (left). Representative images are presented. Scale bars: 200 µm. (H) **Working model of circPRKCB119aa in preeclampsia**. The circular RNA‐encoded protein circPRKCB119aa acts as a bait to impede the phosphorylation of the corresponding linear‐splicing isoform, PKCβ, consequently enhancing cellular autophagy. In the context of preeclampsia, the downregulation of circPRKCB119aa ultimately leads to a decrease in trophoblast cell autophagy. Data are presented as the bar charts (B, F and G, mean ± SD) and violin plots (A, the median (dotted line), upper and lower quartiles (solid lines)); two‐tailed *p* values were calculated by one‐way ANOVA followed by Tukey's post hoc test (F and G) or Mann–Whitney *U* test (A and B). *p  <  .05, **p  <  .01 and ***p  <  .001. PE, preeclampsia. See also Figure S9.

To explore the potential clinical implications of circPRKCB119aa, we first detected its expression in placental tissues from normotensive and preeclamptic pregnant women at both mRNA and protein levels. The clinical characteristics of subjects have been described in our previous study,^26^ and are provided in Table S2. Briefly, the maternal age and pregestational BMI were comparable between the two groups, while the gestational age at delivery, highest blood pressure and newborn birth weight were significantly different. Both *circPRKCB* (*n* = 30 samples per group, Figure 7A) and circPRKCB119aa (*n* = 4 randomly selected samples per group, Figure 7B and C) were significantly downregulated in the preeclamptic placentas. After adjusting for gestational age, maternal age and BMI, *circPRKCB* mRNA expression remained significantly lower in the preeclampsia group (Table S9). We also observed downregulation of circPRKCB119aa in a preeclamptic mouse model with L‐NAME treatment (Figure 7D).

We subsequently analysed changes in placental autophagy using the markers p62 and LC3 and detected a decrease in autophagy in preeclampsia placentas with low expression of circPRKCB119aa (Figure 7E). This finding supports our hypothesis that the reduced placental expression of circPRKCB119aa may lead to decreased autophagy, potentially contributing to preeclampsia.

We also evaluated the effects of circPRKCB119aa on trophoblast phenotype, which is typically abnormal in preeclampsia. Overexpression of *circPRKCB* rather than mut*‐circPRKCB* resulted in a dramatic increase in trophoblast migration and invasion (Figures 7F and S9A and B), as well as in vascular remodelling as indicated by HTR‐8/SVneo cells replacing human umbilical vascular endothelial cells (HUVECs) in tube‐like structures (Figure 7G). However, neither *circPRKCB* nor mut‐*circPRKCB* affected cellular viability (Figure S9C) or the colony forming capacity (Figure S9D). Together, these data suggest that circPRKCB119aa is clinically significant and underscores the potential importance of CEPs in the development of preeclampsia.

## 4 DISCUSSION

### 4.1 Key findings

Studies increasingly point towards a ‘hidden’ proteome encoded by circRNAs that may play a role in human health and diseases.^13^, ^14^ However, only a few CEPs have been identified and functionally linked with cancer.^4^, ^13^, ^14^ In this study, we provided both in silico and experimental evidence for a hidden circRNA‐encoded proteome in the human placenta. We identified 528 ribosome‐associated circRNAs and confirmed translation of CEPs from 139 circRNAs. The CEPs can be physicochemically distinct from the canonical proteins, and regulate placentation and placental dysfunction based on their bait features. Furthermore, we corroborated the biological plausibility of CEPs at the experimental level with circPRKCB119aa as an example. CircPRKCB119aa is a conserved preeclampsia‐related microprotein, which enhanced trophoblast autophagy by inhibiting phosphorylation of its cognate linear‐spliced isoform PKCβ (Figure 7H). To the best of our knowledge, this is the first comprehensive analysis of placental CEPs, which supplement the classical human proteome. Our study provides novel insights into the mechanisms underlying placentation and placental diseases such as preeclampsia.

### 4.2 Comparison and interpretation

It is now evident that circRNAs are much more than ‘noncoding’ transcripts and are also translated. This class of proteins, referred to as CEPs, has been shown to be ubiquitous in human tissues such as the heart,^12^ and they are involved in numerous biological processes.^13^ However, systematic analysis has overlooked this class of proteins due to several reasons. These include technical challenges in detecting them, particularly their relatively lower abundance throughout the proteome, difficulty in attributing a peptide to a circRNA rather than an mRNA origin, and the algorithm's focus on evolutionary conservation and minimum ORF length. In this study, we obtained the basic coding data from both translation and protein levels through ribosome profiling and LC‐MS/MS. To distinguish the inherent noise from the true translational signals,^42^ we used a subsequent set of CEP‐special analysis strategy, including searching a random peptide library as the negative control and restricting the effective ribosome footprints on back‐splicing junctions, in order to identify hundreds of reliable translational events of placental circRNAs. This lays a foundation for their function interpretation, and can prove to be a valuable repository of factors underlying mammalian physiology and disease.

Given their short length and limited domains, CEPs are also referred to as microproteins^12^, ^65^ and are functionally distinct from the proteins coded by the cognate linear mRNA.^3^, ^16^, ^66^ We recently proposed the protein bait hypothesis to explain the functional mechanism of CEPs in various biological and pathophysiological events.^18^ The interplay between CEPs and LEPs constitutes a feedback loop, ensuring maintenance of homeostasis in biological process. The CEPs detected in the present study also showed the characteristic features of baits. Furthermore, a novel CEP circPRKCB119aa was identified that inhibits phosphorylation of its cognate linear‐spliced isoform PKCβ through bait function. These data, therefore, provided additional support for the theory.

Mitochondria are vital organelles in eukaryotic cells that play essential roles in bioenergetics, metabolism and signalling. These functions necessitate intricate protein networks involved in respiration, metabolite transport, protein quality control and the regulation of membrane architecture.^67^ Our findings indicate a significant localisation of CEPs in the mitochondria, suggesting their potential involvement in mitochondrial functions. This aligns with previous studies that have demonstrated the localisation of a considerable number of microproteins, which are encoded by noncoding RNAs, in mitochondria.^12^

The placenta undergoes remarkable changes during gestation to adapt to the metabolic and nutritional requirements of the growing fetus. The proteome of the placenta is properly reprogrammed, which is observed as spatiotemporal alterations in protein composition and levels.^68^ We found that the CEPs‐associated pathways underwent longitudinal changes during placental development: from proliferation in the first trimester to inflammation in the second trimester, and apoptosis in the third trimester. This is partially consistent with the phenotypic transformation of the placenta during pregnancy, and suggested that our comprehensive map of placental CEPs revealed new players in placentation and pregnancy complications.

CEPs are involved in processes such as myogenesis^4^ and cancer.^14^ However, little is known regarding their roles in nononcogenic disease to date. Herein, we present experimental evidence, for the first time, supporting the role of CEPs in pregnancy complications, exemplified by circPRKCB119aa. Through a variety of molecular techniques, we confirmed the existence of circPRKCB119aa and elucidated a potential mechanism wherein elevated levels of circPRKCB119aa competitively inhibit PKCβ kinase binding, consequently leading to PKCβ inactivation and subsequent modulation of PKCβ‐mediated autophagy. Additionally, we explored the potential clinical implications of this pathway within the context of preeclampsia. These findings not only validate the in silico analysis of the newly discovered CEPs but also expand the clinical relevance of CEPs beyond oncogenic diseases.

### 4.3 Limitations of this study

This study has some limitations that ought to be considered. First, although multiomics approaches have provided the supporting evidence for existence of CEPs, false positives are unavoidable. Therefore, the results need to be further substantiated with antibody‐based detection and molecular cloning tools. Second, bioinformatic analysis cannot identify the causal effects. A major future task will be to establish clear causal relationships between CEPs, except for circPRKCB119aa, and placentation, and to determine whether these links are misaligned during pregnancy complications, which is ongoing in our laboratory. Third, evaluating the upstream determinants of CEPs, especially the opposite regulators of the cognate isoforms, is vital to decode CEP‐dependent maintenance of biological homeostasis. Fourth, it should be noted that the sample size in this study is relatively small, further investigations based on large‐scale proteomic analysis are necessary to gain a better understanding of the CEP‐mediated network and explore its potential roles in placental development. Finally, the differences of CEP expression observed in the different stages of the placenta may be attributed to the varying abundance of cell types among the placentas. Therefore, single‐cell level detection of CEP protein or RNA, as well as cell‐specific functional studies, can provide further insights into the role of CEP in placental development or related diseases.

## 5 CONCLUSIONS

We comprehensively characterised the landscape of translating circRNAs and CEPs in human placenta and associated them with placentation and pregnancy complications such as preeclampsia. Our findings suggest a widespread translation of circRNAs, and provide paradigmatic guidance on further CEP research in other human tissues. Importantly, the pro‐autophagic role of circPRKCB119aa rationalises targeting this new category of proteins as an alternative or add‐on strategy for the treatment of preeclampsia.

## AUTHOR CONTRIBUTIONS

Xiaotian Li, Duan Ma and Qiongjie Zhou designed the project. Huanqiang Zhao and Yu Xiong performed the experiments. Huanqiang Zhao, Zixiang Zhou, Yu Xiong, Xirong Xiao, Lili Gong, Huangfang Xu, Lidong Liu, Huiqing Lu, Yutong Cui, Shuyi Shao, Jin Zhang and Jing Ma processed the data. Huanqiang Zhao wrote the manuscript. Huanqiang Zhao, Zixiang Zhou, Qixin Xu, Yang Zi, Xiujie Zheng and Shiguo Chen revised the manuscript. All the authors contributed to and approved the final version of this manuscript.

## CONFLICT OF INTEREST STATEMENT

The authors declare no competing interests.

## ETHICS STATEMENT

The study was approved by the Ethics Committee of Obstetrics and Gynecology Hospital of Fudan University (Ethics approval number: 2017−57).

## Supporting information

Supporting Information

Supporting Information

Supporting Information

Supporting Information

Supporting Information

Supporting Information

Supporting Information

Supporting Information

Supporting Information

Supporting Information

## Data Availability

The raw data of circRNA sequencing have been deposited in Gene Expression Omnibus (GSE196032). The raw data of LC‐MS/MS and ribosome profiling sequencing were deposited in PMB database (http://microprotein.cn/index.aspx). The datasets used in this study are available from CircBase (http://www.circbase.org/), CircRNADb (http://202.195.183.4:8000/circrnadb/circRNADb.php) and CircAtlas (http://circatlas.biols.ac.cn/). The data supporting the findings of this study are available from the corresponding authors upon reasonable request.
