# Supplementary figures and images for: A hidden proteome encoded by circRNAs in human placentas: Implications for uncovering preeclampsia pathogenesis
Source: Clin Transl Med. 2024 Jul 12;14(7):e1759. doi: 10.1002/ctm2.1759 (PMC11245404; doi:10.1002/ctm2.1759)

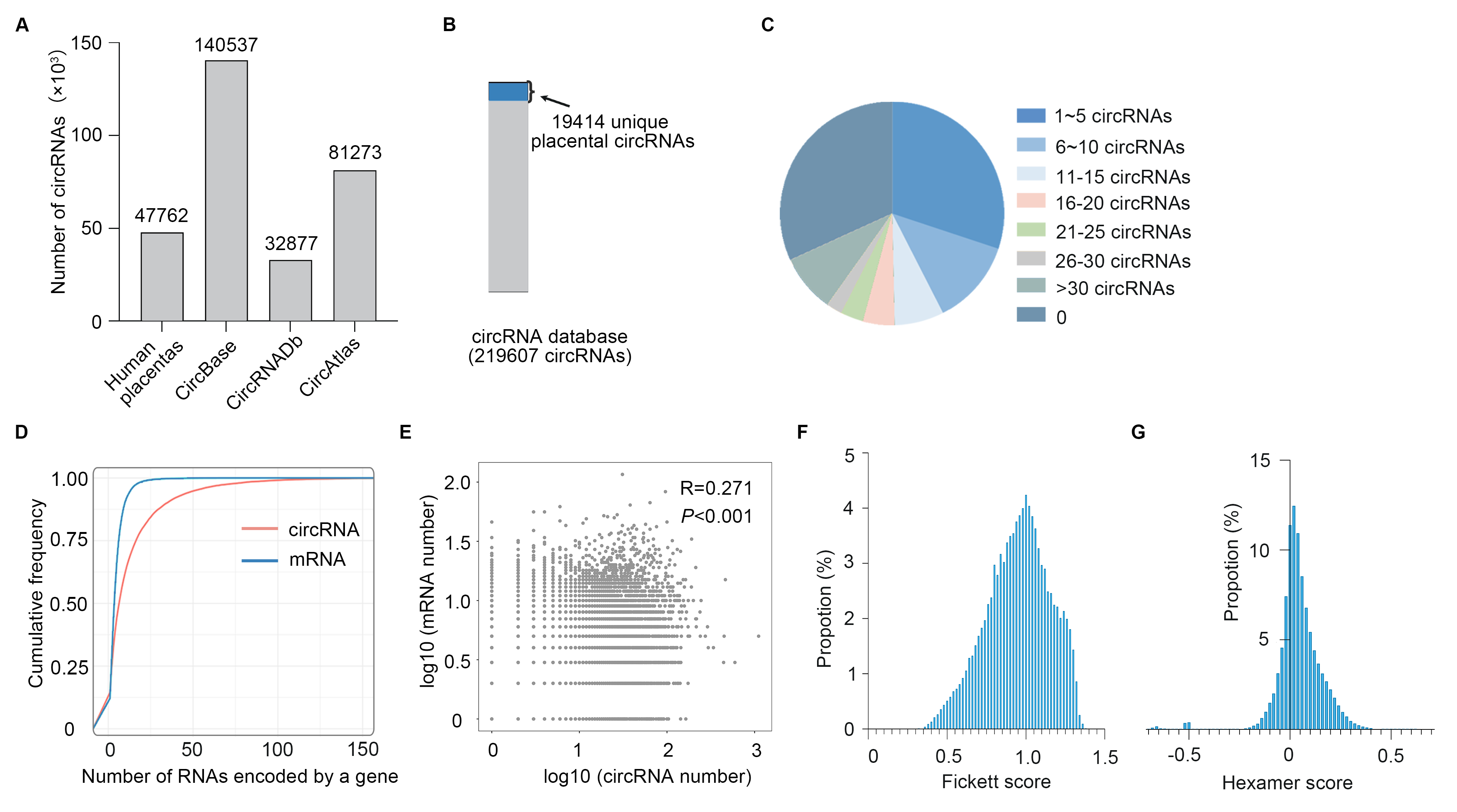

Supplement: Supplementary file 1 — Supporting Information [file CTM2-14-e1759-s008.tif]

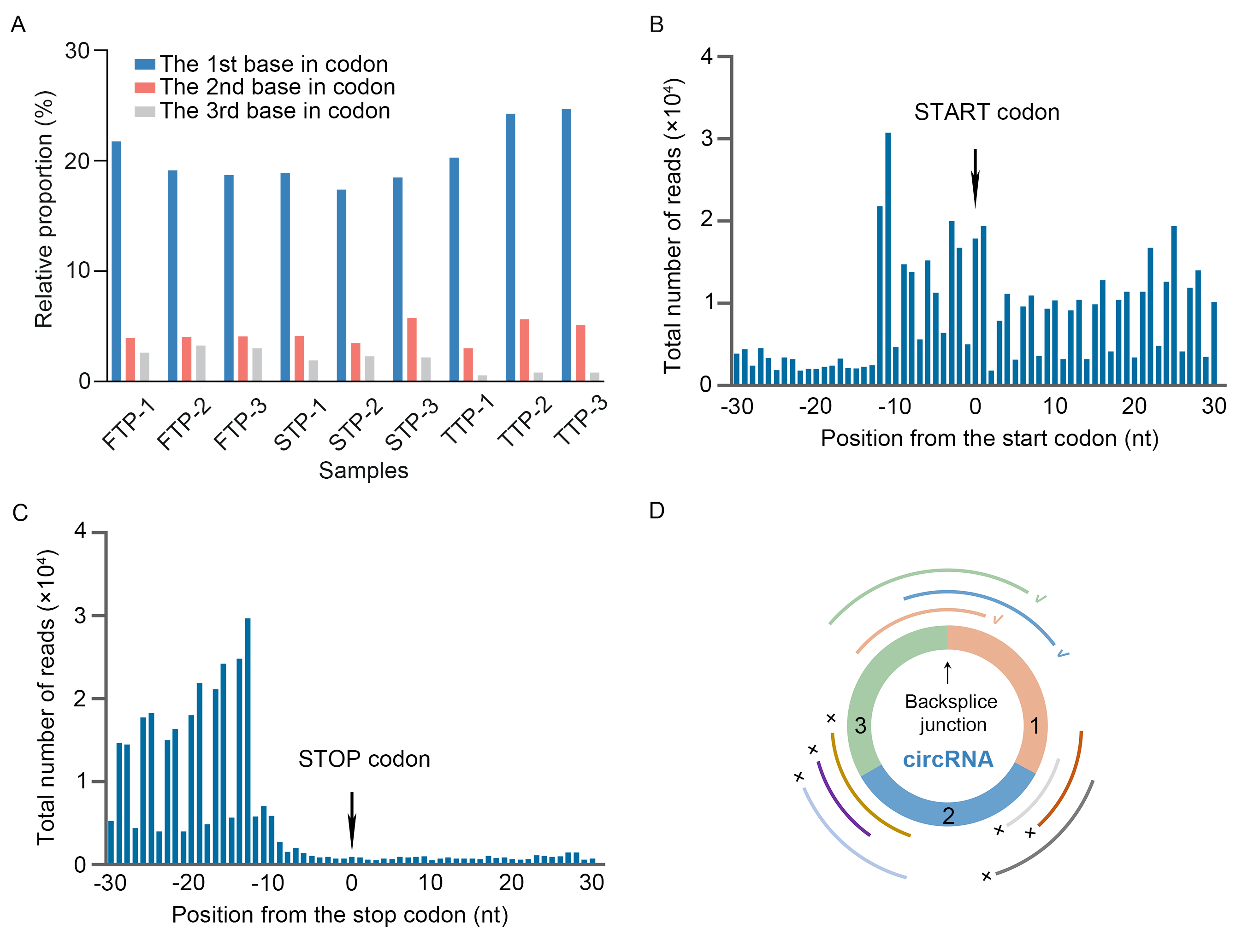

Supplement: Supplementary file 2 — Supporting Information [file CTM2-14-e1759-s003.tif]

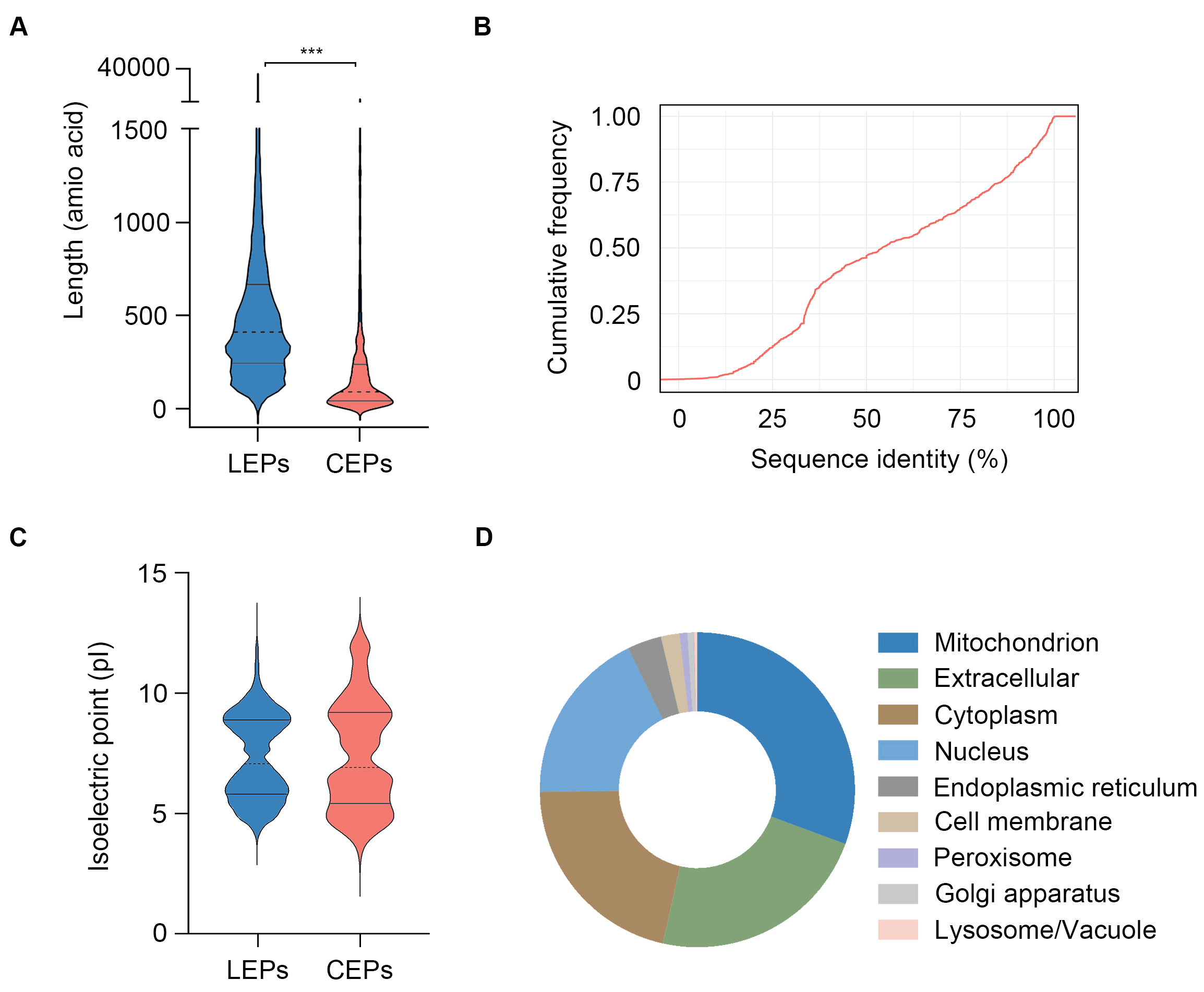

Supplement: Supplementary file 3 — Supporting Information [file CTM2-14-e1759-s007.tif]

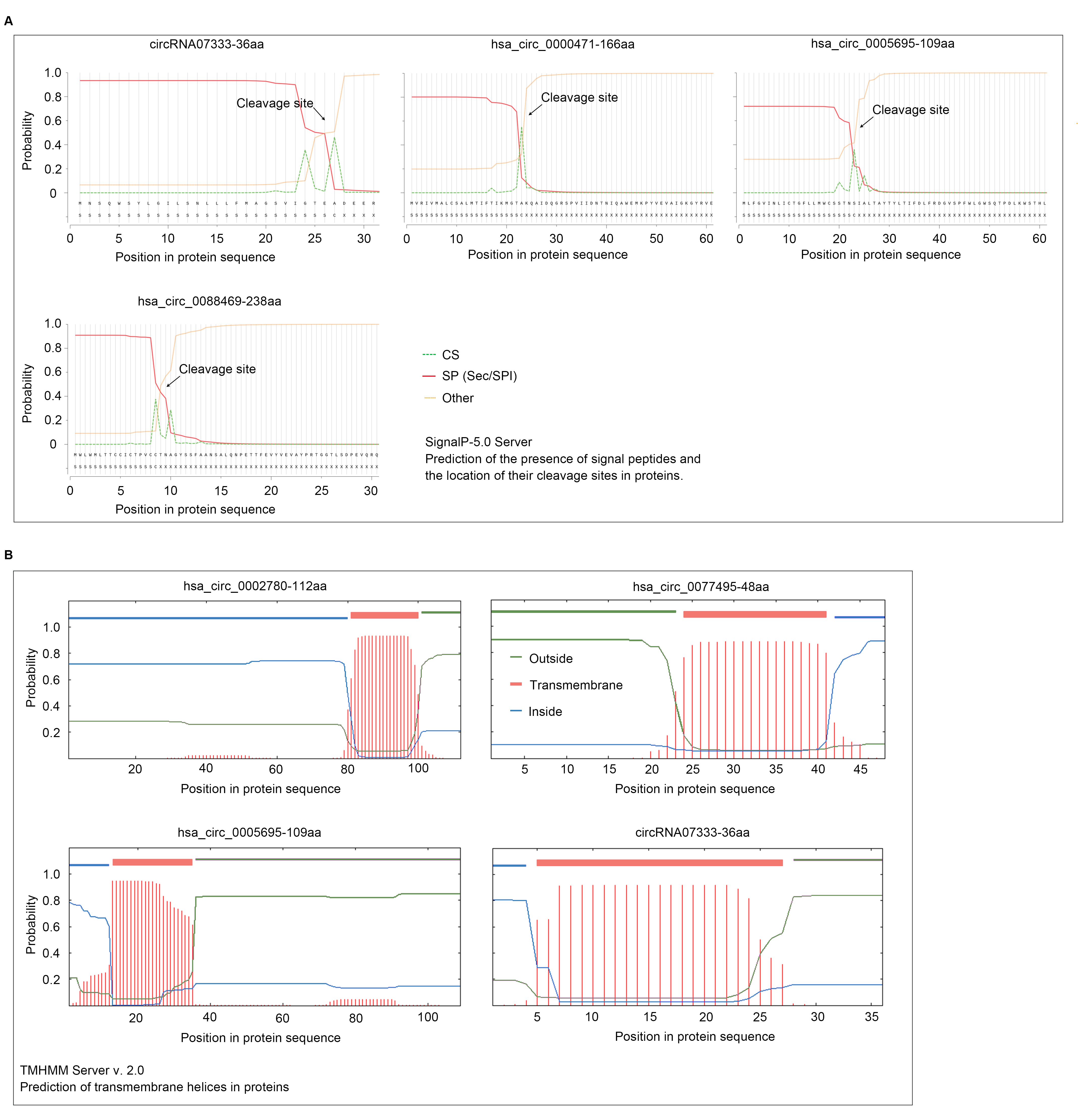

Supplement: Supplementary file 4 — Supporting Information [file CTM2-14-e1759-s006.tif]

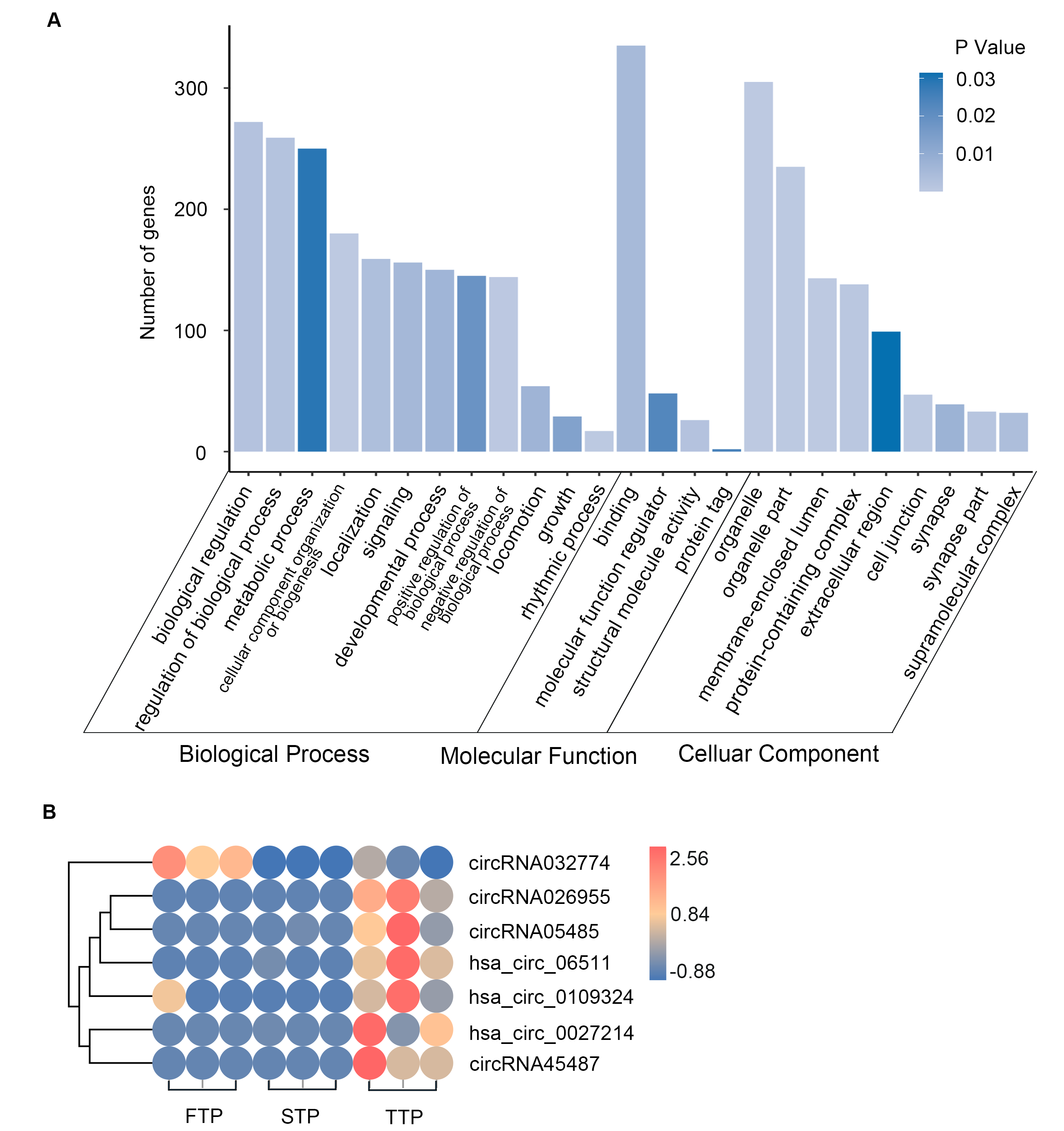

Supplement: Supplementary file 5 — Supporting Information [file CTM2-14-e1759-s001.tif]

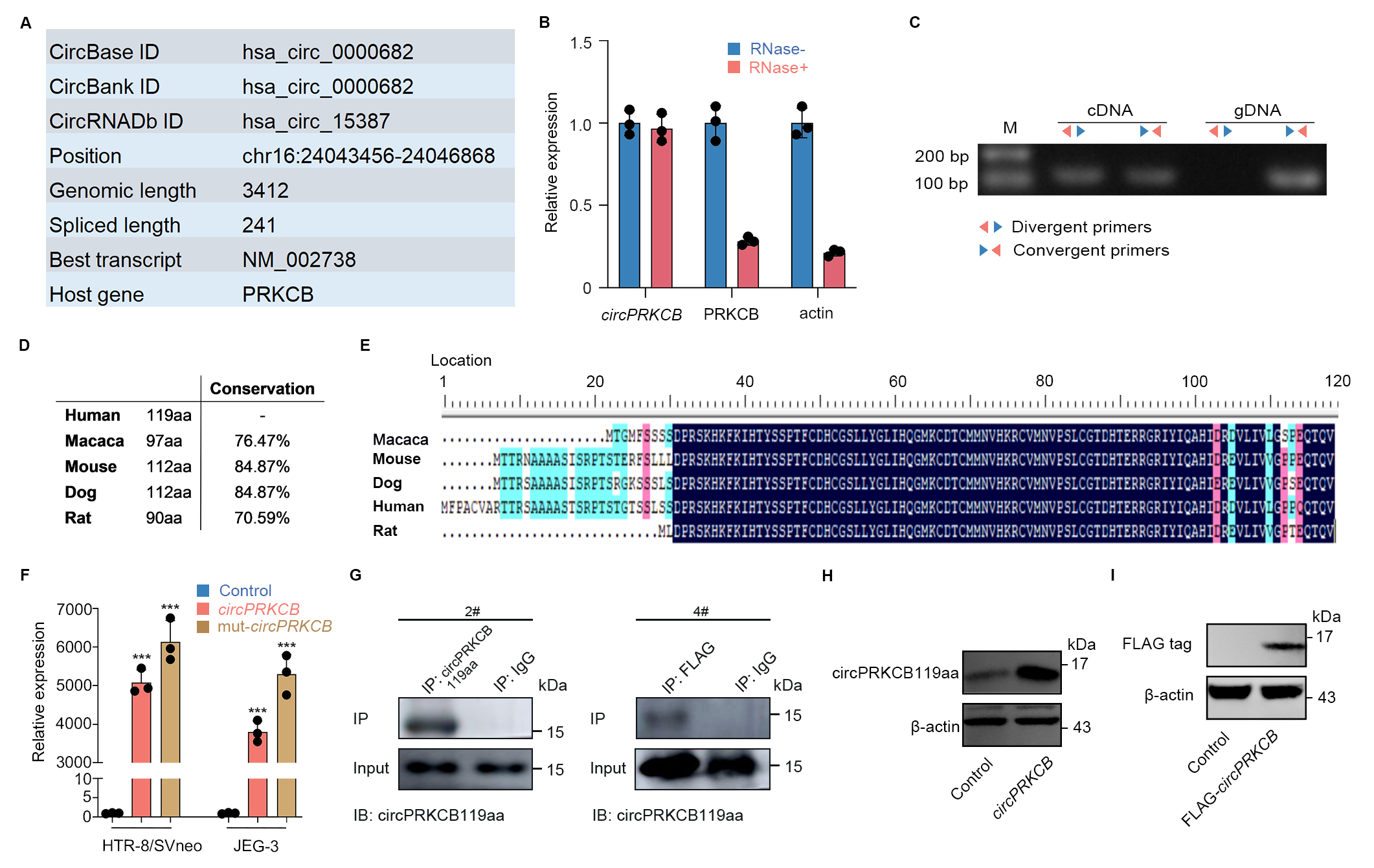

Supplement: Supplementary file 6 — Supporting Information [file CTM2-14-e1759-s009.tif]

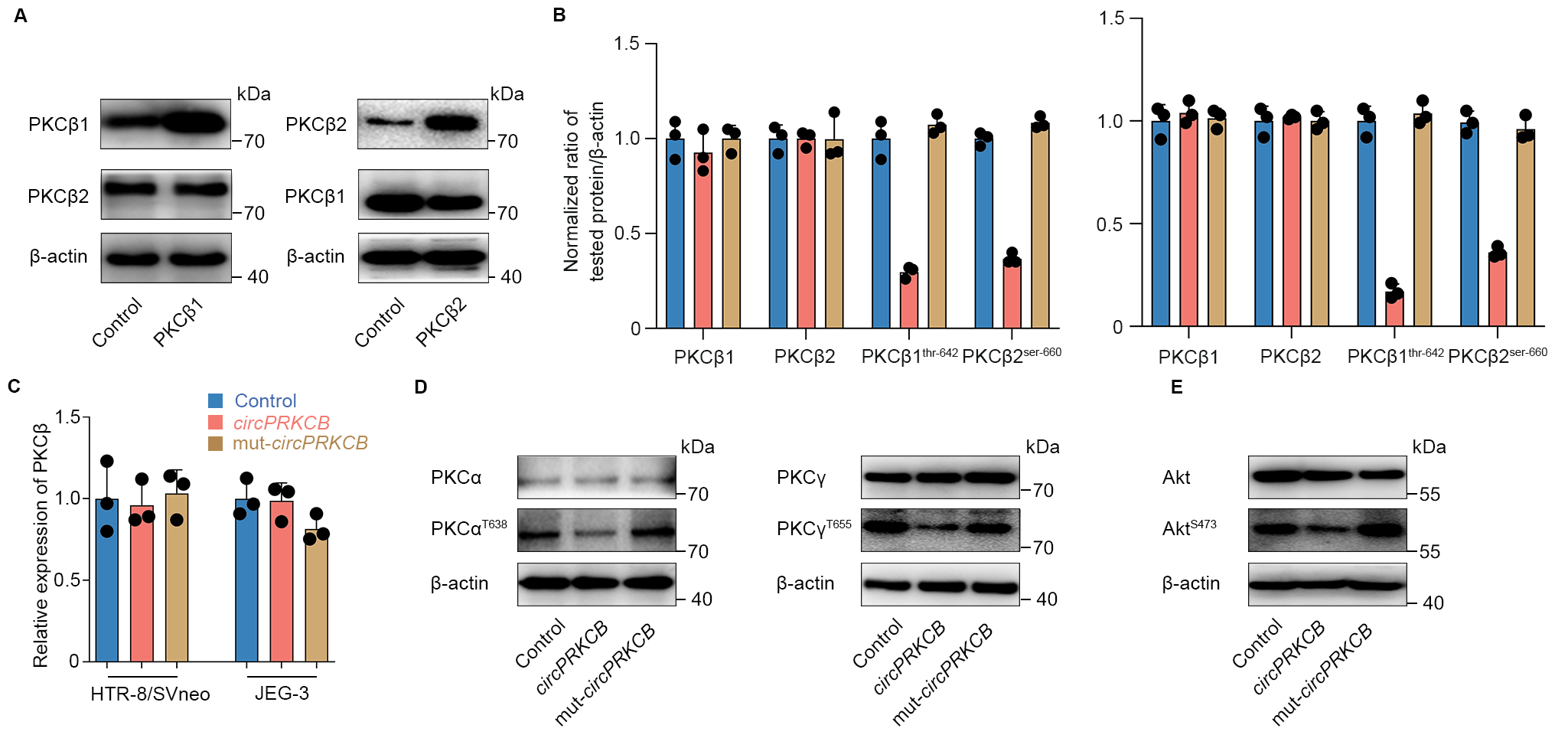

Supplement: Supplementary file 7 — Supporting Information [file CTM2-14-e1759-s010.tif]

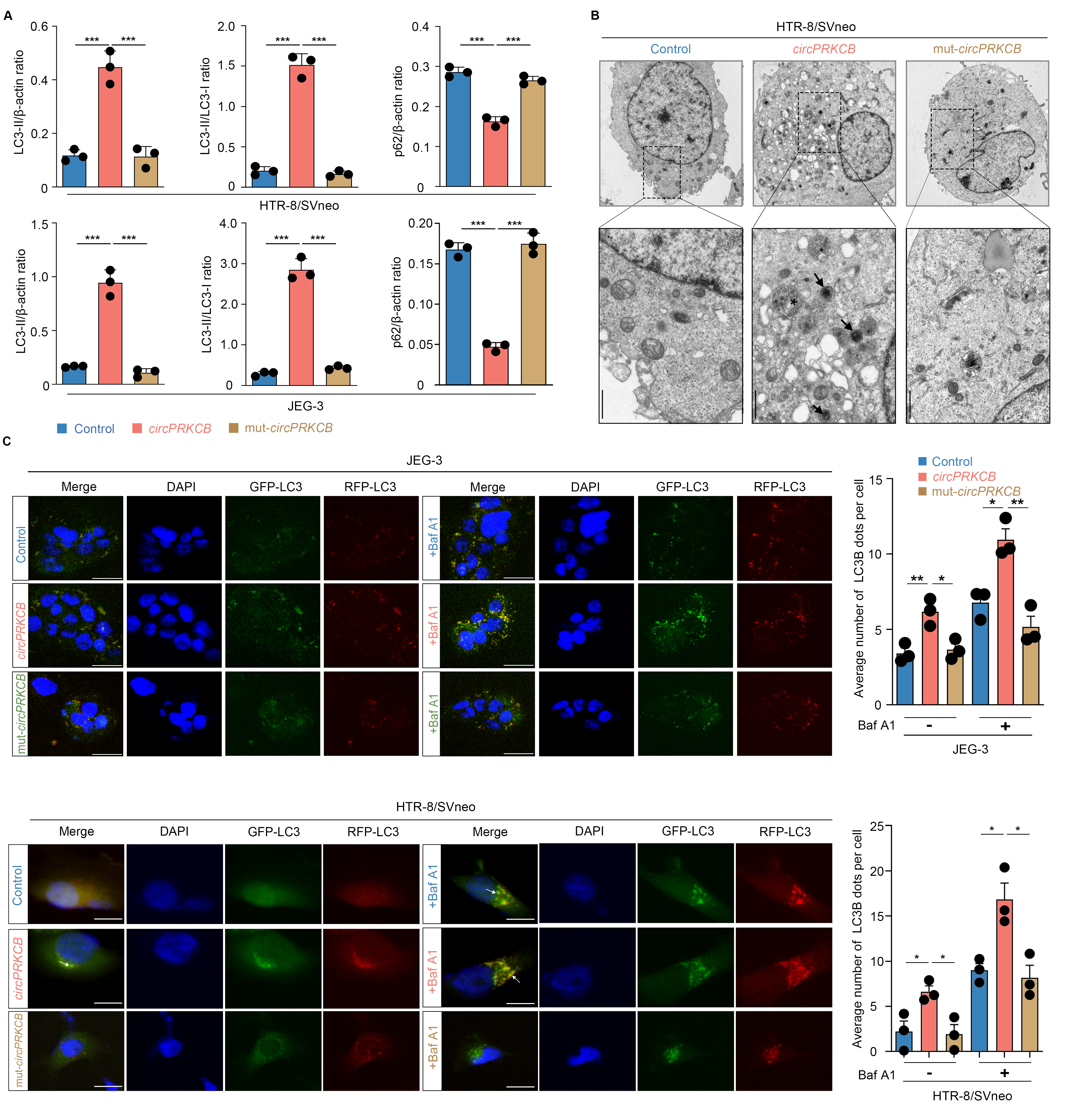

Supplement: Supplementary file 8 — Supporting Information [file CTM2-14-e1759-s002.tif]

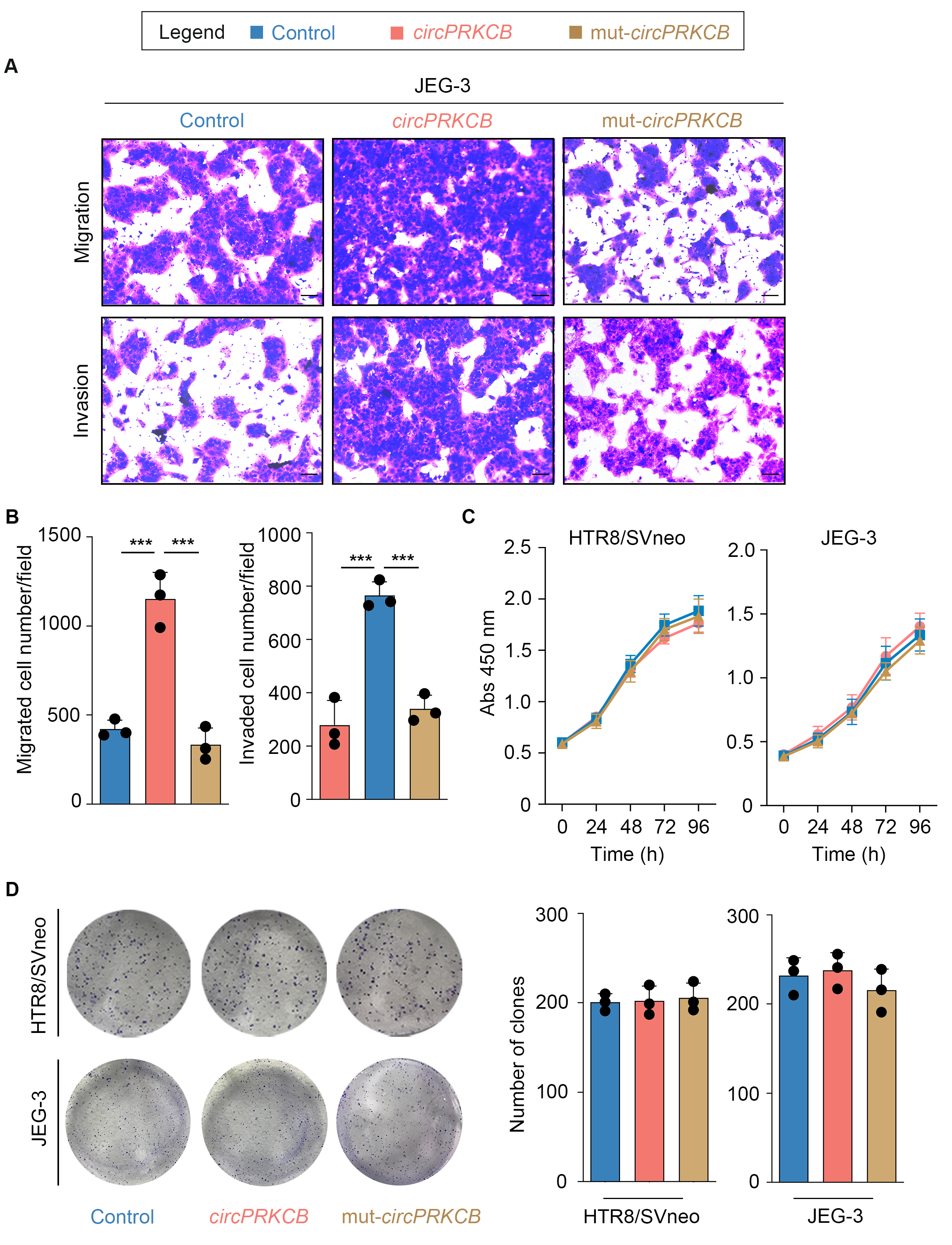

Supplement: Supplementary file 9 — Supporting Information [file CTM2-14-e1759-s005.tif]
