# Supplementary material for: A hidden proteome encoded by circRNAs in human placentas: Implications for uncovering preeclampsia pathogenesis
Source: Clin Transl Med. 2024 Jul 12;14(7):e1759. doi: 10.1002/ctm2.1759 (PMC11245404; doi:10.1002/ctm2.1759)
Supplement: Supplementary file 10 — Supporting Information [file CTM2-14-e1759-s004.docx]

| **Supplementary Table 1. The clinical characteristics of the study population for CEP identification.** | | | | | | | | | | | | | | |
| --- | --- | --- | --- | --- | --- | --- | --- | --- | --- | --- | --- | --- | --- | --- |
| **Sample Number** | **General Sample Id** | **Tissue Source** | **Period** | **Maternal Age (years)** | **Gestational Age （weeks）** | **Maternal BMI (Pregestational)** | **Maternal Ethnicity** | | **Previous Miscarriage** | **previous hypertensive pregnancy** | **HELLP diagnosis** | **IUGR diagnosis** | **PE diagnosis** | **Chorioamnionitis diagnosis** |
| 1 | FTP-1 | Obstetrics and Gynecology Hospital of Fudan University, Shanghai, China | First trimester | 26 | 7.0 | 19.6 | | Asian | Yes | NO | **/** | **/** | **/** | / |
| 2 | FTP-2 | Obstetrics and Gynecology Hospital of Fudan University, Shanghai, China | First trimester | 23 | 6.6 | 22.1 | | Asian | NO | NO | / | / | / | / |
| 3 | FTP-3 | Obstetrics and Gynecology Hospital of Fudan University, Shanghai, China | First trimester | 28 | 6.1 | 23.4 | | Asian | NO | NO | / | / | / | / |
| 4 | STP-1 | Obstetrics and Gynecology Hospital of Fudan University, Shanghai, China | Second trimester | 22 | 18.9 | 20.0 | | Asian | NO | NO | **/** | NO | **/** | NO |
| 5 | STP-2 | Obstetrics and Gynecology Hospital of Fudan University, Shanghai, China | Second trimester | 30 | 20.0 | 23.6 | | Asian | Yes | NO | NO | NO | NO | NO |
| 6 | STP-3 | Obstetrics and Gynecology Hospital of Fudan University, Shanghai, China | Second trimester | 28 | 18.6 | 18.4 | | Asian | No | NO | / | NO | / | NO |
| 7 | TTP-1 | Obstetrics and Gynecology Hospital of Fudan University, Shanghai, China | Third trimester | 32 | 39.4 | 23.1 | | Asian | NO | NO | NO | NO | NO | NO |
| 8 | TTP-2 | Obstetrics and Gynecology Hospital of Fudan University, Shanghai, China | Third trimester | 31 | 38.6 | 19.2 | | Asian | NO | NO | NO | NO | NO | NO |
| 9 | TTP-3 | Obstetrics and Gynecology Hospital of Fudan University, Shanghai, China | Third trimester | 27 | 39.6 | 22.6 | | Asian | Yes | NO | NO | NO | NO | NO |
| FTP, first-trimester placenta; STP, second-trimester placenta; TTP, third-trimester placenta. | | | | | |  | |  |  |  |  |  |  |  |
| “/” means not applicable and indicates that the diagnosis is inappropriate for the given gestational age. | | | | | | | | | | | | | | |
| HELLP, Hemolysis, Elevated Liver enzymes and Low Platelets | | | | | |  | |  |  |  |  |  |  |  |
| IUGR, Intrauterine growth restriction | | | | | |  | |  |  |  |  |  |  |  |
| PE, Preeclampsia | | | | | |  | |  |  |  |  |  |  |  |

| **Supplementary Table 2. Clinical characteristics of the study samples for comparing the levels of circPRKCB and its translational products between groups.** | | | |
| --- | --- | --- | --- |
| **Variables** | **Control (n=30)** | **Preeclampsia (n=30)** | ***p* value** |
| Maternal age (years) | 28.6 ± 3.2 | 29.9 ± 3.3 | >0.05 |
| Gestational age at delivery (weeks) | 38.8 ± 0.6 | 36.3 ± 2.1 | <0.001 |
| Pregestational BMI (kg/m^2^) | 21.7 ± 2.7 | 21.9 ± 3.6 | 0.823 |
| Highest SBP (mm Hg) | 110.8 ± 11.0 | 154.5 ± 12.1 | <0.001 |
| Highest DBP (mm Hg) | 71.1 ± 8.7 | 98.7 ± 6.6 | <0.001 |
| Proteinuria (g/24h) | NA | 2.4 ± 2.0 | NA |
| Birth weight (g) | 3313.0 ± 325.6 | 2526.9 ± 615.9 | <0.001 |
| Infant gender (F, %) | 43.3 | 46.7 | >0.05 |
| Values shown are means ± SD. BMI, body mass index; NA, not applicable; SBP, systolic blood pressure; DBP, diastolic blood pressure | | | |

| **Supplementary Table 3. Primers used in this study** | | |
| --- | --- | --- |
| **Primer name** | **Sequence** | **Product (bp)** |
| U1-F^#^ | GACGGGAAAAGATTGAGCGG | 112 |
| U1-R^#^ | GCCACGAAGAGAGTCTTGAAGG |  |
| actin-F^#^ | AGAGCTACGAGCTGCCTGAC | 184 |
| actin-R^#^ | AGCACTGTGTTGGCGTACAG |  |
| circPRKCB-F^#^ | CTCATTGTCCTCGGACCCCC | 154 |
| circPRKCB-R^#^ | GCAGCGCTTGTGCACATTCAT |  |
| circPRKCB-F (Divergent primer) ^$^ | CATCTACATCCAGGCCCACATC | 100 |
| circPRKCB-R (Divergent primer) ^$^ | ACACAGGCTGGGAACATTCATC |  |
| circPRKCB-F (Convergent primer) ^$^ | CGCTGCGTGATGAATGTTCC | 101 |
| circPRKCB-R (Convergent primer) ^$^ | ACAATGAGGACGTCCCTGTC |  |
| circPRKCB-F ^&^ | CGCTGCGTGATGAATGTTCC | 216 |
| circPRKCB-R ^&^ | TCACATTTCATCCCCTGGTGG |  |
| # used only for qRT-PCR; $ used for PCR; &, used for PCR and the subsequent Sanger sequencing. | | |

| **Supplementary Table 4. Ribo-seq reads mapped to ribosomal RNAs, tRNAs, miRNAs, snRNAs and snoRNAs** | | | | | | | |
| --- | --- | --- | --- | --- | --- | --- | --- |
| **Sample** | **All Reads Num** | **rRNA** | **tRNA** | **snoRNA** | **snRNA** | **miRNA** | **other sRNA** |
| FTP-1 | 71348590 | 39285207 | 944545 | 48719 | 1514197 | 2741870 | 1944500 |
| FTP-2 | 57056857 | 27589905 | 849585 | 40146 | 928352 | 849237 | 1657198 |
| FTP-3 | 50402853 | 24968252 | 453222 | 43698 | 1440018 | 2090809 | 1592286 |
| STP-1 | 45040606 | 23619643 | 2106298 | 11887 | 875983 | 78177 | 2467225 |
| STP-2 | 52246771 | 19867412 | 3709000 | 12340 | 1232061 | 52105 | 1463987 |
| STP-3 | 52671299 | 24603069 | 2272911 | 13007 | 1000371 | 81256 | 2644434 |
| TTP-1 | 64997440 | 30783314 | 2532553 | 15051 | 871044 | 709760 | 3295107 |
| TTP-2 | 52763571 | 29731595 | 3063401 | 25449 | 982755 | 437448 | 2537748 |
| TTP-3 | 45351183 | 20995309 | 2245018 | 10916 | 2077509 | 140863 | 4694979 |

| **Supplementary Table 5. Ribo-Seq identified 528 translating circRNAs** | |
| --- | --- |
| **circRNA** | **Sequence** |
| circRNA010050 | GATGAAACAAGTGAAGATGCTAACTGTCTTGCTTTGAGTGGACATGATAAAACAGAAGCAAAGGTATACTAATTTAGCCTTTAGAATACATACACAATTAAGAGTACAGAAGATGTAATAGAAATGTCTCTAAAATATTGTATTTTTGTGCTATAGGTTTTAGATTTTTTTAGTATTGTTTATTACTGTTAATTATGTAATGCTGAACACTGAAGGGAATATTGTGATGAGTGAAACACATTCTGTGCCTTTAAAGGAGCTTATAATTAAGTAGACAAGATAAGTCATGAATGTAATTAATTACATTATCAAAGAAAAAGTAATTGAGGGCTGGGGGCCACGGGGCAAGAAAAGTAAGATGTTATTTCTGACTGCAGAAATAGCAGTACATGTCTTCATTGAAGAAAGAAGTAGCATTTGAAGTGGTCCTCAAAGGATACATAGGATTTTTGATAGTCCGGGCTGAGATCATGATCTGTCTCTTTGGGTTGAGAATTTGTTATGATTATAGAAGCAGTTTTTGGCGTAAGGTTTTGGCTAGAACCTGGGGTACATGGGGCTTGAATGAGGTGTAGATGGGGTGGAAGAAATTTTTTTTTCCCCAAAATGTAAAAGACTCATTTTTTTTCCCTAGAAATTGAAATCTATTGAAGGCTTTTAACTTTCTTTCCTTGTGGAAAAATTGGAAATTCTCCCCCTTCTCCCAAATTATCCAGATTTCTATGATGGATATTTTAACAAAACTCTCTTAGGTTTTGTGTGTGCCCTCATAATGCCCTTGCTTTATAATTTTGATTCACTTTGTAAACCAAGAAAAAAGTATGTGTATCATTATGTAATCTACAAAATTATTAATGGCTTCATAGAATGCTGTCTTAAGGCTATGATTATTTTTATCCAACCAATTTCCTATCCATAGCAATTATTTCCATTTAAAAAAGAAAGTTGTGCTTTCCTGCTGTAATATTGGTTACAGCTAAATACCATATCTGCCATTCATTCATGTTTATTTACTTAGCATAAATTAATGGAAGAAGAATTATTGAGGAAAATAGTATGCTTTATCTTAAAGTTTGGTATACATCACCAAGTTGTCCTCCAGGAAGGCTGCATGTCTTCCATTCCTACTTCTTACATCTTATTTACCTTTTTTAATTTCATAGGAGAAAACAATATTTCGTTTTTTTTTTTTTTGGTATAGTTTCATTGAAATTGAAGTTTTTATATTTTTCTTTTCTTCTTTGTCCTTTTTTTGGTTTTTTTTGAGATGGAGTCTTGCTCTGTCGCCCAGGCTGGAGTGCAGTGGGACAATCTCGGCTCACTGCAACCTCTGCCTCCTAGGTTCAAGCAATTCTTCTGCCTCAGCCTCCCGAGTAGTGGGGATTACAGGCACCCGCCACCATGCCTGGCTAATTTTGTATTTTTAGTAGAGATGGGGTTTCACCATGTTGGCCAGGCTGGTCTTGAACTCCTGATGTCATGATCTCCCCACCTCGGCCTCCCAAAGTGCTAGGATTACAGGTGTGAGCCACCACGTCTGGCCTATATTTTTATTTTCATTTATTTGTGTAGATATTATTTTCTTTTGTGTACTGCTTTTAAAAAGTTTAGAGTGATGGCATGACATACTTTACCCTGTGTTTTCTGATGAGTAGAATCAGTGCACTGATTACTAGAGAGGGGTGAAGAGACCATAAGGAGGGAGAAATCTTTTTTAGACATATTTTCTACTTTTAATTAAAGGTGTGAGGTTATTTTATTTAGTAGTATTGAATAAGTTTTTTTTTTTTTTTTTCCTCCCACAAAGCTAAGAACTGTCCTTGGGTTTATGCTCTGTTATTGTCTGGGCAACATTTCATGATGTGCCTATGTGTGTTTTTAATTATTGTTTGCTTAAAATGTCCCAAGTTGGAGCTGGTTGGAAAGTCATGAGCTAGTTTGACCCATGCCTTATAAGATTTAAGTAGGCAAATAGAAATTTATTAATATCTGAAATGGTGAAATAACCTACAACATCTACTGACTAGATGTTTTTGTAATGTAGTCACATCTTTCTCTTGATCCCTCCTGTCTTCCAGTTTGTCTTCTTTATTAGCCACCAAACCCAGTTATGTTCTTTTATTCTTTTTTAAGTCTCTTTTCCCCCTTCCCCATCCTCTTTGTCTCACAGGAACAACTTGATACAGAGACAAGTACAACTCAATCAGAAACTATTCAGACAGCGGCTTCTCTGTTGGCCTCTCAGGTACTAAGTGCAAAAAGCAAGGAGAATTTTGTAAATGTCCTTAAGATTAGTGTGGTATATTGAATTAAAGTGAGAGTTCATAGAAGACTATTAAAATTAATCTTTCTGAAAGTAATTACTTGCTAGCCTATAAACGTATTTATTGTAGTTTTAAGGCAGGGCCCTGGATTTGACGGAACCGTTCTCTGGGACTGTTTCGATGTTAACCCATCCCCTTATATTAATATATTATTTGATACTGCAAATCATTTAAATTCTCACTAGTCATTTACACAGGGTTCATATGTAGGTTTTATTTGTAGGTCAAAAGTGAAATTAAAACTAGGAATGAACTAAAAAACATTCTTCGTGGATTTCTTTTGCAAAGGCTAATATATTTTTATTTTAAAAATGTATTTCTGCCTTATTTTTCCACTACTGATTTCAGTTAGTTATTTAATATAGCCCATAGCTCTTCAGAATTATGTTTTCATTTGTCCTAGCAATTCTACTTGTGACAGACCCTCACATACAGAATACAGTAGGCATCTGGAATCTTTTTTCATTGATTTGTGAACCTTCATTTTGTTGAATAATTTTTTTCTTGAATCCTTTCACATGAATGAACCCTAACATCTAGCTCAAATTCTGACACAAAGTAGATATATTCTTTAATAGATGATAAAACTGGTTAATGTCTACAAAGTAACTGAGATAACAACTGCTGCTGTACTGATATGATACTATGTACTATGATGATATTTTGCTGCTCCTGTGCAGCTCCGAGTTATTTTAACGTGATCATTTAAACATGTGCTTTTGCTAGATGTTAACTTTTATTGACTCTAAAGATATATAAATATTATGTTTAGTTTCTGAAATACCTTTTCCATAATCTAATAAAACTTTAGGGGGAGTAACTAACATCAGGAAGTATGCTTAATATTGATACGTGGACAAACTTGTTTATCAGTCTTAAACTGTCCTGTGATTGTGATTGCATGTTTTTTGGTTTGCCACATACTCTCGGGACTTATTTTCATAGTTTTGTCTTACTGAATTTATCCCTATAGTAATTAGTACCCATGGGTATTTGCCTTTTAGATATTTTTATGAGTGAGTAGAATTTTCTTTCTAATGAAAGGATTCATAGAGCCATAGAGAGAAAAATCACCCAGTTATGTAAGAAGAATGATTATGTTGATTTAAAAAAAAATTCATTCCTATCTTTGGTAATGTTAGGGGCGTACTTATTTCTGGGGAGGTGATAATATTGTGGCTATACCTAATAAAGAGTTCTTATTGTTTTGAGATAAATCTGGAAATATTTATAGATGAAATGATAAGATCTCTAGGATTTGCTTCAGAATAACACAAGGTGAATTGAAAGTGGGAGGACAGTGGCTTTGATTTGATAATTGATGAAGCTGGGTAATGAATACATGGGGATGCATTATACAGTGCTCTCTACTTTGTGTATGTTTGGAATTTTCCATAATAAAACTTTTAAAGATCCATCTCTGTATTTTTTTAGTGTTAAAGAACGCTTGTGATTTAATCTAATTAATATGAAAATAATCTACCTAAAATTTAGGATGATTAAATTATTGCTTAGCTGTATATATTTGTTTAATTTACAGTGTCAGAAAATAATAAATTTACTGCTGCTTCTGTATATAAAATGAGCTTTGAGTATTCTCCCACTTGGGGGTGCTCCAATACTAGTTAATATTACAGCTATTAGTGTTGTAATAAGTGAATACAGTTTAGCAGTAACTTAAGCATTATATGATAGGTGATTCTTAGAGGAACAATTTTAAAAACTATTTGAAATTACTTAGGAGTTTTTAAAATATTATTAAAATATACCTATTTGAAAATTCCTTTGTTGAACACATTATTTGTTGAACAAATAATGTGATAGGCATATTTACATTCCATTTTGACAACATTATAGAGTTTTTAAATTTGTAATACTTTTCTTCTTAGAAGGTTTCTTAGGAAGTAAAGCAATGGAAAATACATCTCATTGTTAAATTATGGTGCTTTGAACAATGTTGTGTTCTTTGCTTTCATTCTCAGAAAACATCCAGTACAGACCTTTCTGATATCCCTGCTCTCCCTGCAAATCCTATTCCTGTTATCAAGAATTCAATAAAACTGAGATTGAATCGGTAAAAACAACCTCAGGGGTCCATAAACAATATCTGCCAACTCAACCTGTTGTCTTCAAATGCTAAAAAAGGAGAATGGAGGGTACAAGACTAGACATGACTGAAATGGATTTGGGTTTTTTGGTGACCTCCCTTACTGGGCTAATCAGCACTTGATCGGAAGTCCAG |
| circRNA010064 | AGGGCCAGAAAGGATGGGCTTTCCCTTTTCCTCGTGGCAGAAACTTCCCTGACTTCATAGCTGGGTTCCCCCTATCCACAGTTTACAGTACAACTATCTCCTCCAAGTTGGCCAGCATTGCCTCTGATACGTGAACAAGAAGTATTTGGAGGTAAGGTCACCCTTCCTCTGGTTTTATTTTTTTAAAGTTTGGCTTTCAAAACCATTGCTCCTCTGTAGAATCCAAGATTATATAAGATTACATTAAGTCTTTTCTTTCTGTTTGCATTCTTTCTATAACAGGGTTAATAAATACAAATCAACACATATACACATGAAGAGAAAATGGATGTAATAACCAAGGACTGCCTGAT |
| circRNA011723 | TGATGTAAATTGGAGTCAACCAGGTGAAAAGAAGTTAACTGGGGGATCTAACTGGCAACCAAAGGTTGCACCAACAACCGCTTGGAATGCTGCAACAATGGCACCCCCTGTAATGGCCTATCCTGCTACTACACCAACAGGCATGATAGGATATGGAATT |
| circRNA011936 | GTTTTTGGATTCAAAGCATAAAAACCATTACAAGATATACAATCTTTGTGCTGAAAGACATTATGACACCGCCAAATTTAATTGCAGAGTTGCACAATATCCTTTTGAAGACCATAACCCACCACAGCTAGAACTTATCAAACCCTTTTGTGAAGATCTTGACCAATGGCTAAGTGAAGATGACAATCATGTTGCAGCAATTCACTGTAAAGCTGGAAAGGGACGAACTGGTGTAATGATATGTGCATATTTATTACATCGGGGCAAATTTTTAAAGGCACAAGAGGCCCTAGATTTCTATGGGGAAGTAAGGACCAGAGACAAAAAGGGAGTAACTATTCCCAGTCAGAGGCGCTATGTGTATTATTATAGCTACCTGTTAAAGAATCATCTGGATTATAGACCAGTGGCACTGTTGTTTCACAAGATGATGTTTGAAACTATTCCAATGTTCAGTGGCGGAACTTGCA |
| circRNA012383 | GGTATTCTGGAACAAGATGCGTTCCACAGATCTCTCTTGGCCTGCTGCCTTGAGGTCGTCACTTTTTCTTATAAGCCTCCTGGGAATTTTCCATTTATTACTGAAATATTTGATGTGCCTCTTTATCATTTTTATAAGGTGATAGAAGTATTCATTAGAGCAGAAGATGGCCTTTGTAGAGAGGTGGTAAAACACCTTAATCAGATTGAAGAACAGATCTTAGATCATTTGGCATGGAAACCAGAGTCTCCACTCTGGGAAAAAATTAGAGACAATGAAAACAGAGTTCCTACATGTGAAGAGGTCATGCCACCTCAGAACCTGGAAAGGGCAGATGAAATTTGCATTGCTGGCTCCCCTTTGACTCCCAGAAGGGTGACTGAAGTTCGTGCTGATACTGGAGGACTTGGAAGGA |
| circRNA012651 | GAGTCATTGCAGTTTTCAGTAGAGGTGTACTTCTGAGAAGTGGCTTCTTGGGTCTTCATGCAGCCATGGATCTGGATAAACCATCTGTTTGGGGCTCATTAAAACAGCGGACCAGGCCATTGTTGATCAACTTGAGCAAGAAGAAGGTGAAAAAGAACCCAAGTAAGCCCCCAGATCTACGGGCAAGGCATCACTTGGACCGCCGTCTCAGCCTCTCTGTGCCTGATCTCCTGGAGGCTGAGGCCTTGGCCCCAGAGGGCCGGCCTTACTCCGGGCCACAGTCTTCCTACACCTCGGTGCCCAGCAGTCTGTCCACTGCAGGGATCTTTCCCAAGAGCAGCAGTAGCTCCTTGAAACAGTCTGAAGAAGAATTGGATTGGAGCCAGGAAGAAGCCAGTCACCTCCATGTGGTGGAAACAGACTCAGAGGAGGCCTATGCCTCTCCTGCTGAGCGGAGACGGGTGTCCAGCAACGGCATCTTTGATCTTCAGAAAACTTCCCTTGGAGGGGATGCACCAGAAGAGCCAGAGAAGCTATGTGGAAGCAGTGACCTGAATGCTTCTATGACATCTCAACATTTTGAAGAACAATCTGTACCGGGGGAAGCCAGTGATGGCTTGAGTAACCTCCCCAGCCCTTTTGCGTACCTCCTCACCATACACCTGAAGGAAGGCCGGAACCTGGTTGTCCGAGATCGCTGTG |
| circRNA012781 | GAGAAAATGTCCACCTTGGTTCTGGTGATGGGCAGTCAAAAGATTCTGGGCCCCTTCCTCAAGTGGAAAAGAAGCTCAAGTGTACAGTTGAAGGTTGTGACCGGACATTTGTATGGCCAGCTCACTTTAAATACCACCTCAAGACTCATCGAAATGACCGCTCCTTCATCTGTCCTGCAGAAGGTTGTGGGAAAAGCTTCTATGTGCTGCAGAGGCTGAAGGTGCACATGAGGACCCACAATGGAGAGAAGCCCTTTATGTGCCATGAGTCTGGCTGTGGTAAGCAGTTTACTACAGCTGGAAACCTGAAGAACCACCGGCGCATCCACACAG |
| circRNA012959 | TGAAGCTGCCCAAGCCCTACATCACCATCAACAACTCAAAACCCAGGGAGAATAAGGATGTCTTAGCCTTCACCTGTGAACCTAAGAGTGAGAACTACACCTACATTTGGTGGCTAAATGGTCAGAGCCTCCCGGTCAGTCCCAGGGTAAAGCGACCCATTGAAAACAGGATCCTCATTCTACCCAGTGTCACGAGAAATGAAACAGGACCCTATGAATGTGAAATACGGGACCGAGATGGTGGCATGCGCAGTGACCCAGTCACCCTGAATGTCCTCTATGGTCCAGACCTCCCCAGCATTTACCCTTCATTCACCTATTACCGTTCAGGAGAAAACCTCTACTTGTCCTGCTTCGCGGAATCTAACCCACCGGCAGAGTATTTTTGGACAATTAATGGGAAGTTTCAGCAATCAGGACAAAAGCTCTCTATCCCCCAAATTACTACAAAGCATAGAGGGCTCTATACTTGCTCTGTTCGTAACTCAGCTACTGGCAAGGAAAGCTCCAAATCCATGACAGTCGAAGTCTCTG |
| circRNA014889 | GTTGACTTTGGCAGAATATCATGAACAGGAAGAAATTTTCAAACTTAGACTAGGACATCTCAAAAAGAGATAACAGTATAGCAACAACTCTGCCACTGAAATCCTGTTCTCTGACCGATATTGGCACCTGCAAAGAGAAACAACCAGTAACAGGCAGCAGCAGCATCAGTATTAATCTTCCATGATGAAATCTTTACAGGTCAAGAACAAGTACACAGCTCTTTTCTCACTCCTTCACAGTGGACCATGCAACTAGTTGAAATGGAAGACAATGGATTGTCTACAAGCCTTTTGAACAGTGGAGAATGCAGGGCGTTGGCTTTAG |
| circRNA015581 | CGGGGATCTGATGAGCTTCTTTCTTCTGGCATCATTAACGGACCTTTTACCATGAATAGTTCTACTCCTTCTACAGCTAATGGGAATGACAGCAAGAAATTTAAACGAGATAGACCTCCCTGTTCGCCTTCCCGTGTTCTCCATCTTCGAAAAATTCCATGTGATGTCACCGAAGCAGAGATCATATCATTAGGTCTACCATTTGGCAAAGTAACTAATCTTTTGATGTTGAAAGGAAAAAGCCAGGCTTTCTTAGAAATGGCTTCTGAGGAAGCTGCCGTTACTATGGTGAATTATTACACTCCTATTACTCCTCACCTTCGAAGCCAGCCTGTTTATATTCAGTATTCCAATCACAGAGAACTTAAGACTGACAATCTACCTAATCAAGCTCGAGCCCAAGCTGCACTGCAGGCTGTCAGTGCCGTCCAATCAGGAAGCCTGGCCCTTTCTGGAGGTCCTTCCAATGAAGGCACAGTCCTACCTGGGCAGAGCCCTGTGCTTCGAATAATTATTGAAAACCTCTTTTACCCTGTTACCCTGGAAGTTCTTCATCAGATATTTTCTAAATTTGGCACAGTCTTGAAGATTATCACCTTTACAAAGAATAATCAGTTTCAAGCCTTGCTTCAGTATGCTGACCCAGTAAATGCACATTATGCCAAAATGGCTCTGGATGGCCAGAATATCTATAATGCATGCTGCACTCTGCGCATTGACTTCTCCAAGCTCACCAGCCTTAATGTGAAATATAATAATGACAAAAGCAGAGACTTCACTCGCTTAGACCTTCCTACTGGTGATGGCCAGCCATCCCTTGAACCCCCTATGGCTGCTGCTTTTG |
| circRNA015584 | GGCTGTATGACAAATGTTCTTATATCTCACGTGACCGAGGATGGGTCGTGGGCATTCACACCATCAGTGACCAAGACAACAAAGACCCACGCTACTTTTTCTCCTTGAAGACAGACCGAGCCCGGCAAGTGACCACCATCAATGCCCACCGCAGCTACCTCCCAGGCCAGTGGGTATACCTAGCTGCCACCTATGATGGGCAGTTCATGAAGCTCTATGTGAATGGTGCCCAGGTGGCCACCTCTGGGGAACAAGTGGGTGGCATATTCAGCCCACTGACCCAGAAGTGCAAAGTGCTCATGTTAGGGGGCAGTGCCCTGAATCACAACTACCGGGGCTACATCGAGCACTTCAGTCTGTGGAAGGTGGCCAGGACTCAGCGGGAGATACTGTCTGACATGGAAACCCATGGCGCCCACACTGCTCTACCTCAGCTCCTCCTCCAGGAGAACTGGGACAATGTGAAGCATGCCTGGTCCCCCATGAAGGATGGCAGCAGCCCCAAAGTGGAATTCAGCAATGCCCACGGCTTTCTGCTGGACACGAGTCTGGAGCCTCCTCTGTGCGGACAGACATTGTGTGACAACACAGAGGTCATTGCCAGCTACAATCAGCTCTCAAGTTTCCGCCAGCCCAAGGTGGTGCGCTACCGCGTGGTCAACCTCTATGAAGATGATCATAAGAACCCGACGGTGACGCGCGAGCAGGTGGACTTCCAGCACCATCAGCTGGCTGAGGCCTTCAAGCAATACAACATCTCCTGGGAGCTGGACGTGCTGGAGGTGAGCAACTCCTCCCTTCGCCGCCGCCTCATCCTGGCCAACTGTGACATCAGCAAGATTGGGGATGAGAACTGTGACCCCGAGTGCAACCACACGCTGACGGGCCACGACGGCGGGGATTGCCGCCACCTGCGCCACCCTGCCTTCGTGAAGAAGCAGCACAACGGGGTGTGTGACATGGACTGCAACTATGAACGGTTCAACTTTGATGGTGGAGAGTGCTGTGACCCTGAAATCACCAATGTCACTCAGACTTGCTTTGACCCCGACTCTCCACACAGAGCCTACTTGGATGTTAATGAGCTGAAGAACATTCTTAAATTGGATGGATCAACACATCTCAATATTTTCTTTGCAAAATCCTCAGAGGAGGAGTTGGCAGGAGTAGCAACTTGGCCATGGGACAAGGAGGCCCTGATGCACTTAGGTGGCATTGTCTTGAACCCATCTTTCTATGGCATGCCTGGGCACACCCACACCATGATCCATGAGATTGGTCACAGCCTGGGCCTCTATCACGTCTTCCGAGGCATCTCAGAAATCCAGTCCTGCAGTGACCCCTGCATGGAGACAGAGCCCTCCTTCGAGACTGGAGACCTCTGCAATGATACCAACCCAGCCCCTAAACACAAGTCCTGTGGTGACCCAGGGCCAGGAAATGACACCTGTGGCTTTCATAGCTTCTTCAACACTCCTTACAACAACTTCATGAGCTATGCAG |
| circRNA01630 | AGCAACTTATGACTCTCATCTCTGCTGCACGAGAATATGAGATAGAGTTCATCTATGCGATCTCACCTGGATTGGATATCACTTTTTCTAACCCCAAGGAAGTATCCACATTGAAACGTAAATTGGACCAGGTTTCTCAGTTTGGGTGCAGATCATTTGCTTTGCTTTTTGATGATATAGACCATAATATGTGTGCAGCAGACAAAGAGGTATTCAGTTCTTTTGCTCATGCCCAAGTCTCCATCACAAATGAAATCTATCAGTACCTAGGAGAGCCAGAAACTTTCCTCTTCTGTCCCACAGAATACTGTGGCACTTTCTGTTATCCAAATGTGTCTCAGTCTCCATATTTAAGGACTGTGGGTGAAAAGCTTCTACCTGGAATTGAAGTGCTTTGGACAGGTCCCAAAGTTGTTTCTAAAGAAATTCCAGTAGAGTCCATCGAAGAGGTTTCTAAGATTATTAAGAGAGCTCCAGTAATCTGGGATAACATTCATGCTAATGATTATGATCAGAAGAGACTGTTTCTGGGCCCGTACAAAGGAAGATCCACAGAACTCATCCCACGGTTAAAAGGAGTCCTCACTAATCCAAATTGTGAATTTGAAGCCAACTACGTTGCTATCCACACCCTTGCCACCTGGTACAAATCAAACATGAATGGAGTGAGAAAAGATGTAGTGATGAGTAGGCAAGTTGCACACAGTGGAGCTAAAGCAAGTGTAGTTGATGGGACTCCTTTAGTTGCAGCACCCTCTTTAAATGCCACAACCGTAGTAACAACAGTTTATCAGGAGCCCATTATGAGCCAGGGAGCAGCCTTGAGTGGTGAGCCTACTACTCTGACCAAGGAAGAAGAAAAGAAACAGCCTGATGAAGAACCCATGGACATGGTGGTGGAAAAACAAGAAGAAACGGACCACAAGAATGACAATCAAATACTGAGTGAAATTGTTGAAGCGAAAATGGCAGAGGAATTGAAACCAATGGACACTGATAAAGAGAGCATAGCTGAATCAAAATCCCCAGAGATGTCCATGCAAGAAGATTGTATTAGTGACATTGCCCCCATGCAAACTGATGAACAGACAAACAAGGAGCAGTTTGTGCCAGGTCCAAATGAAAAGCCTTTGTACACTGCGGAACCAGTGACCCTGGAGGATTTGCAGTTACTTGCTGATCTATTCTACCTTCCTTACGAGCATGGACCCAAAGGAGCACAGATGTTACGGGAATTTCAATGGCTTCGAGCAAATAGTAGTGTTGTCAGTGTCAATTGCAAAGGAAAAGACTCTGAAAAAATTGAAGAATGGCGGTCACGAGCAGCCAAGTTTGAAGAGATGTGTGGACTAGTGATGGGAATGTTCACTCGGCTCTCCAATTGTGCCAACAGGACAATTCTTTATGACATGTACTCCTATGTTTGGGATATCAAGAGTATAATGTCTATGGTGAAGTCTTTTGTACAGTGGTTAGGGTGTCGTAGTCATTCTTCAGCACAATTCTTAATTGGAGACCAAGAACCCTGGGCCTTTAGAGGTGGTCTAGCAGGAGAGTTCCAGCGTTTGCTGCCAATTGATGGGGCAAATGATCTCTTTTTTCAGCCACCTCCACTGACTCCTACCTCCAAAGTTTATACTATCAGACCTTATTTTCCTAAGGATGAG |
| circRNA016607 | GTTCTCTCTGGAAGTTATGTTGAATCAACACTGAAGGGACAAAGGGGAAGCAGGGAGACCAATTAGGAGGCTGCTGTAGTCATCCAGGTAAGAGATGATGGTGGCTGGGACCAAGGTGGAATTGGTGATGGAATTCCTACCTGTCCCTTCCAGGCTGTGCAATGGGACAACTTTAAGAGCCAGAGTCTCCAGCTGTCACTACCCTGTCACCATCCCTACTATCATCTCTGGACCCCATATTTCACCTGTGCTCACCCTGACACCACTTCTGCCCTTGTGTCTCCTCACAGCAAGTGTGGGTGCTACTTCCCCTGAGATGCCATGAGTTTCTTTTTCCTTATTGCCAGGATTGCCCTGAGCCATCACAGGCCCTTGATTTTTACTATCTAAGCTGTATTTCCTCTGCCCATCCATTAGGAAGAAGATTGCTGTCTGGTTTGAACTGGAATTTCTCGCTATGTCCCACTGGAG |
| circRNA01724 | GGGACTTATTGTGTTTGAAAATGAAAGCTATGTCTTAGAACCAATGAAAAGTGCAACCAACAGATACAAACTCTTCCCAGCGAAGAAGCTGAAAAGCGTCCGGGGATCATGTGGATCACATCACAACACACCAAACCTCGCTGCAAAGAATGTGTTTCCACCACCCTCTCAGACATGGGCAAGAAGGCATAAAAGAGAGACCCTCAAGGCAACTAAGTATGTGGAGCTGGTGATCGTGGCAGACAACCGAGAGTTTCAGAGGCAAGGAAAAGATCTGGAAAAAGTTAAGCAGCGATTAATAGAGATTGCTAATCACGTTGACAAGTTTTACAGACCACTGAACATTCGGATCGTGTTGGTAGGCGTGGAAGTGTGGAATGACATGGACAAATGCTCTGTAAGTCAGGACCCATTCACCAGCCTCCATGAATTTCTGGACTGGAGGAAGATGAAGCTTCTACCTCGCAAATCCCATGACAATGCGCAGCTTGTCAGTGGGGTTTATTTCCAAGGGACCACCATCGGCATGGCCCCAATCATGAGCATGTGCACGGCAGACCAGTCTGGGGGAATTGTCATGGACCATTCAGACAATCCCCTTGGTGCAGCCGTGACCCTGGCACATGAGCTGGGCCACAATTTCGGGATGAATCATGACACACTGGACAGGGGCTGTAGCTGTCAAATGGCGGTTGAGAAAGGAGGCTGCATCATGAACGCTTCCACCGG |
| circRNA017254 | TGTCACTGGTGAGTCGAGGAAAAACTCCTTTCCCAAGTGTAACGTTTTAATTAAAAGAAAGTAAAAGGTTTAATCAATAAAAAAGACTATGAGAATATTCATCTCTTGAGAGTCCAAAATTAACGTATTTATTGTTCAAAAGAGACGGGGTCTCGCTATGTTGCCCAGGCTGGAGTGCAGTGGCTATTCACAGGCGCGATCCCACTACTGATCAGCACGGGAGTTTTGACCTGCTCCGTTTCCGACCTGGGCCGGTTCACCCCTCCTTAGGCAACCTGGTGGTCCCCCGCTCCCGGGAG |
| circRNA017314 | GCCTCAGACGATACAGCAGACTCTCAAGAGGACACTGCAGTATTATGAACATCAAGTTATTGGTTACAGGGATGCAGAAAAGAATTTCCACAATATCTCTAACAGATGCTCCTATGCAGACCACTCCAACAAAGAAGAAATTGAAGATGTCTCAGGAATTCTTCAGTGTACTGCTAATATACTC |
| circRNA018689 | GACCTGTGTTTCTTGGTCACCGGGATGAGCTTGAGGGAACATACAAGATTTACTGCCAGAATCATGATGAGGCCATTGCGCTGCTTGAAATCTACGAGAAGGATGAGAAGATCCAGAAGCATCTTCAGGACTCCTTGGCAGATCTGAAGAGCCTATACAACGAATGGGGATGCACAAATTATATTAACCTGGGCTCCTTCCTCATCAAACCAGTACAGAGAGTAATGCGTTACCCGCTGTTGCTAATGGAGTTGCTGAATTCCACCCCAGAATCCCACCCAGATAAAGTGCCTTTAACCAATGCAGTCCTTGCGGTCAAGGAAATCAACGTTAACATTAATGAATATAAACGGCGAAAGGACCTGGTCCTCAAGTACCGTAAGGGTGATGAAGATAGCCTTATGGAGAAAATTTCCAAACTGAACATCCACTCCATCATCAAGAAATCCAACCGAGTTAGCAGTCACCTGAAGCATCTCACTGGCTTTGCTCCTCAGATAAAAGATGAAGTATTTGAAGAAACAGAAAAAAACTTCCGAATGCAAGAAAGATTGATTAAGTCTTTTATCCGAGACCTGTCTCTCTACCTCCAGCACATCCGG |
| circRNA018769 | GGGACTTATTGTGTTTGAAAATGAAAGCTATGTCTTAGAACCAATGAAAAGTGCAACCAACAGATACAAACTCTTCCCAGCGAAGAAGCTGAAAAGCGTCCGGGGATCATGTGGATCACATCACAACACACCAAACCTCGCTGCAAAGAATGTGTTTCCACCACCCTCTCAGACATGGGCAAGAAGGCATAAAAGAGAGACCCTCAAGGCAACTAAGTATGTGGAGCTGGTGATCGTGGCAGACAACCGAGAGTTTCAGAGGCAAGGAAAAGATCTGGAAAAAGTTAAGCAGCGATTAATAGAGATTGCTAATCACGTTGACAAGTTTTACAGACCACTGAACATTCGGATCGTGTTGGTAGGCGTGGAAGTGTGGAATGACATGGACAAATGCTCTGTAAGTCAGGACCCATTCACCAGCCTCCATGAATTTCTGGACTGGAGGAAGATGAAGCTTCTACCTCGCAAATCCCATGACAATGCGCAGCTTGTCAG |
| circRNA018853 | AATGTCACAGGTAGACTAATGGTTGGCCTACGTTGGTGGAATCACATTGATGAAGATGGAAAGAGCCATTGGGTGTTTGAATCTAGAAAGGAGTCCTCTCAAGAGAATAAAACTGTGTCAGAGGCTGAATCAAGAATCTTTTGGTTGGGACTTATTGCCTGTTCAGTACTGTGGGTGATATTTGCCTTTAGTGCACTCTTCTCCTTCACAGTAAAGTGGCTG |
| circRNA020003 | TCCGGTTCCAGTTGTTGGCAAAGGAGAGGAAGAGGAAGAGGAAGATGGCATGCGGCTTTGTCTGCCAGCCAACCCGAAAAACTGCCTTCCTCACCGCCGGGGCATCAGCATCCTGGAGAAGCTCATCAAAACATGCCCGGTGTGGCTGCAGCTGAGTCTGGGCCAGGCAGAGGTGGCCAGGATCCTGCACCGGGTGGTGGCTGGGATGTTCCTGGTTCGCCGGGACAGCAGCTCGAAGCAGCTGGTGCTCTGTGTCCACTTTCCTTCTCTGAACGAAAGCTCGGCCGAGGTGCTCGAATACACCATTAAGGAAGAAAAGTCGA |
| circRNA020175 | TGGAGACTCCCAAGCCCTCCATCTCCAGCAGCAAATTAAACCCCAGGGAGGCCATGGAGGCTGTGAGCTTAACCTGTGATCCTGAGACTCCGGACGCAAGCTACCTGTGGTGGATGAATGGTCAGAGCCTCCCTATGTCTCACAGGTTGCAGTTGTCTGAAACCAACAGGACCCTCTTTCTATTGGGTGTCACAAAGTACACTGCAGGACCCTATGAATGTGAAATACGGAACCCAGTGAGTGCCAGCCGCAGTGACCCATTCACCCTGAATCTCCTCC |
| circRNA020827 | GGATTGTGCTGCATTCTGACTTAGATTCTGCCATCAAAAACAGAATGCCCTGATCCTTAGAGGCAGAGGCAGCTCTTCAGCCTATGGGCTTAAGCTCTTGGACTCACTGAGGCAGTAAAAGAACCATATCCTGTGTTTGAATCAAACCCCGAGTTCCTGTATGTTTAGAAGGTTTGCCAGACAGGATTCCCTTTTGAAGCCCTCCTGGTTTGGAATTCCATGACTTAAAAGGATCGTCCGTGGGAGTAATAACATCAAGTCTGTTGGTAAAAACTTCGCAGTTTCTAAAAAGACCACGAAGCCCTGAGAGTAATGAAAAGGTTCCTGAAATTGAGGTCACTGTGGAAGAAGGAAACGTGTCATGTACCAGCTATCCATCCAATGACTGCCCATCAGTTCCATATTCACCCTTCACTGCCTGATCTGCAAACATAGAGGAGAATCTAATGCCTGATGAACTGTCATTGTCTCCCATCACCCCCAGATGGGACCATCTAGTTGCAGGAAAAGAAGGTCAAGACTCCCACTCATTCTACATTATGTCTTCCTGTGTTGCCCAGGCTGGAGCACAGTGGTTTGATCTTGGCTCACTGCAGTCTCAACCTCTCAGGCTCAAGCTATCCTTCTGCCTCAGACTCTGGGGTAGCTGGGACAACAGCCTCAGCCAAGATGTCTCACCCTACTCTCTATGATGCAACAAGAAGCCCCTGGGGAATGTTTCAGTCCCAGTCTGTACTATTGTCATGTGCTCATCACAGTCTGGTGGAATTTAAATGACCTTTTATCAAGATGGATAAACGCAAGTTTCCCAGTGCTGGAATACAGAAAATGGATGGACAA |
| circRNA021462 | GGCTGGTGAATTACCAGATCTCCGTCAAGTGCAGTAACCAGTTCAAGTTGGAAGTGTGTCTTTTGAAATCAGAAAACAAAGTCGTGGACAACCAGGCTGGGACCCAGGGCCAGCTGAAGGTGCTGGGTGCCAACCTCTGGTGGCCGTACCTGATGCACGAACACCCCGCCTACCTGTACTCGTGGGAGGATGGTGATTGCTCACACCAAAGCCTTGGACCCCTCCCAGCCTGTGACCTTTGTGACCAACTCCACCTACGCAGCAGACAAGGGGGCTCTGTATGTGGATGTGATCCGTGTGAACAGCTACTACTCTTGGTATCGCAACTACGGGCACCTGGAGTTGATTCAGCTGCAGCTGGCCGCCCAGTTTGAGAACTGGTGTAAGACATCACAATCCCATTATTCAGAGCGTGTATGGAGTGGAAACGCTTGTAGGGTTTCACCAGGATCCACCTTGATGTTCAGTGAAGAGTACCAGAAAAGTCTGCTAGAGCAGTACCATCTGGGTCTCAATCAAAAACGCAGAAAATACGTGGTTGGAGAGCTCATCTGGAATTTTGCCGATTTCATGACTAACCAGT |
| circRNA021573 | GGCTGGTGAATTACCAGATCTCCGTCAAGTGCAGTAACCAGTTCAAGTTGGAAGTGTGTCTTTTGAATGCAGAAAACAAAGTCGTGGACAACCAGGCTGGGACCCAGGGCCAGCTGAAGGTGCTGGGTGCCAACCTCTGGTGGCCGTACCTGATGCACGAACACCCCGCCTACCTGTACTCGTGGGAGGATGGTGATTGCTCACACCAAAGCCTTGGACCCCTCCCAGCCTGTGACCTTTGTGACCAACTCCACCTACGCAGCAGACAAGGGGGCTCTGTATGTGGATGTGATCCGTGTGAACAGCTACTACTCTTGGTATCGCAACTACGGGCACCTGGAGTTGATTCAGCTGCAGCTGGCCGCCCAGTTTGAGAATTGGTGTAAGACATCACAATCCCATTATTCAGAGCGCGTATGGAGTGGAAACGCTTGTAGGGCTTCACCAGGATCCACCTCTGATGTTCAGTGAAGAGGACCGGAAAAGTCTGCTAGAGCAGTACCATCTGGGTCTGGATCAAAAACGCAGAAAATACGTGGTTGGAGAGCTCATCTGGAATTTTGCCGATTTCATGACTAACCAGT |
| circRNA021696 | ATGTTGATGAATGCCAGACCCCAGGAATCTGCATGAATGGGCACTGCATCAACAGTGAAGGGTCCTTCCGCTGTGACTGTCCCCCAGGCCTGGCTGTGGGCATGGATGGACGTGTGTGTGTTGATACTCACATGCGCAGTACCTGCTATGGAGGAATCAAGAAAGGAGTGTGTGTGCGTCCTTTCCCCGGTGCAGTGACCAAGTCCGAATGCTGCTGTGCCAATCCAGACTATGGTTTTGGAGAACCCTGCCAGCCATGCCCTGCAAAAAATTCAGCTGAATTCCACGGCCTTTGTAGTAGTGGAGTAGGTATCACTGTGGATGGAAGAGATATCAATGAATGTGCTTTGGATCCTGATATATGTGCCAATGGGATTTGTGAAAACTTACGTGGTAGTTACCGTTGTAATTGCAACAGTGGCTATGAACCAGATGCCTCTGGAAGAAACTGTATTG |
| circRNA024119 | CATACAACTGGCAGGATCTTTGAGAGTGAAGTGAGGTATCAGTGTAACCCGGGCTATAAGTCAGTCGGAAGTCCTGTATTTGTCTGCCAAGCCAATCGCCACTGGCACAGTGAATCCCCTCTGATGTGTGTTCCTCTCGACTGTGGAAAACCTCCCCCGATCCAGAATGGCTTCATGAAAGGAGAAAACTTTGAAGTAGGGTCCAAGGTTCAGTTTTTCTGTAATGAGGGTTATGAGCTTGTTGGTGACAGTTCTTGGACATGTCAGAAATCTGGCAAATGGAATAAGAAGTCAAATCCAAAGTGCATGCCTGCCAAGTGCCCAGAGCCGCCCCTCTTGGAAAACCAGCTAGTATTAAAGGAGTTGACCACCGAGGTAGGAGTTGTGACATTTTCCTGTAAAGAAGGGCATGTCCTGCAAGGCCCCTCTGTCCTGAAATGCTTGCCATCCCAGCAATGGAATGACTCTTTCCCTGTTTGTAAGATTGTTCTTTGTACCCCACCTCCCCTAATTTCCTTTGGTGTCCCCATTCCTTCTTCTGCTCTTCATTTTGGAAGTACTGTCAAGTATTCTTGTGTAGGTGGGTTTTTCCTAAGAGGAAATTCTACCACCCTCTGCCAACCTGATGGCACCTGGAGCTCTCCACTGCCAGAATGTGTTCCAGTAGAATGTCCCCAACCTGAGGAAATCCCCAATGGAATCATTGATGTGCAAGGCCTTGCCTATCTCAGCACAGCTCTCTATACCTGCAAGCCAGGCTTTGAATTGGTGGGAAATACTACCACCCTTTGTGGAGAAAATGGTCACTGGCTTGGAGGAAAACCAACATGTAAAGCCATTGAGTGCCTGAAACCCAAGGAGATTTTGAATGGCAAATTCTCTTACACGGACCTACACTATGGACAGACCGTTACCTACTCTTGCAACCGAGGCTTTCGGCTCGAAGGTCCCAGTGCCTTGACCTGTTTAGAGACAGGTGATTGGGATGTAGATGCCCCATCTTGCAATGCCATCCACTGTGATTCCCCACAACCCATTGAAAATGGTTTTGTAGAAGGTGCAGATTACAGCTATGGTGCCATAATCATCTACAGTTGCTTCCCTGGGTTTCAGGTGGCTGGTCATGCCATGCAGACCTGTGAAGAGTCAGGATGGTCAAGTTCCATCCCAACATGTATGCCAATAGACTGTGGCCTCCCTCCTCATATAGATTTTGGAGACTGTACTAAACTCAAAGATGACCAGGGATATTTTGAGCAAGAAGACGACATGATGGAAGTTCCATATGTGACTCCTCACCCTCCTTATCATTTGGGAGCAGTGGCTAAAACCTGGGAAAATACAAAGGAGTCTCCTGCTACACATTCATCAAACTTTCTGTATGGTACCATGGTTTCATACACCTGTAATCCAGGATATGAACTTCTGGGGAACCCTGTGCTGATCTGCCAGGAAGATGGAACTTGGAATGGCAGTGCACCATCCTGCATTTCAATTGAATGTGACTTGCCTACTGCTCCTGAAAATGGCTTTTTGCGTTTTACAGAGACTAGCATGGGAAGTGCTGTGCAGTATAGCTGTAAACCTGGACACATTCTAGCAGGCTCTGACTTAAGGCTTTGTCTAGAGAATAGAAAGTGGAGTGGTGCCTCCCCACGCTGTGAAGCCATTTCATGCAAAAAGCCAAATCCAGTCATGAATGGATCCATCAAAGGAAGCAACTACACATACCTGAGCACGTTGTACTATGAGTGTGACCCCGGATATGTGCTGAATGGCACTGAGAGGAGAACATGCCAGGATGACAAAAACTGGGATGAGGATGAGCCCATTTGCATTCCTGTGGACTGCAGTTCACCCCCAGTCTCAGCCAATGGCCAGGTGAGAGGAGACGAGTACACATTCCAAAAAGAGATTGAATACACTTGCAATGAAGGGTTCTTGCTTGAGGGAGCCAGGAGTCGGGTTTGTCTTGCCAATGGAAGTTGGAGTGGAGCCACTCCCGACTGTGTGCCTGTCAGATGTGCCACCCCGCCACAACTGGCCAATGGGGTGACGGAAGGCCTGGACTATGGCTTCATGAAGGAAGTAACATTCCACTGTCACGAGGGCTACATCTTGCACGGTGCTCCAAAACTCACCTGTCAGTCAGATGGCAACTGGGATGCAGAGATTCCTCTCTGTAAACCAGTCAACTGTGGACCTCCTGAAGATCTTGCCCATGGTTTCCCTAATGGTTTTTCCTTTATTCATGGGGGCCATATACAGTATCAGTGCTTTCCTGGTTATAAGCTCCATGGAAATTCATCAAGAAGGTGCCTCTCCAATGGCTCCTGGAGTGGCAGCTCACCTTCCTGCCTGCCTTGCAGATGTTCCACACCAGTAATTGAATATGGAACTGTCAATGGGACAGATTTTGACTGTGGAAAGGCAGCCCGGATTCAGTGCTTCAAAGGCTTCAAGCTCCTAGGACTTTCTGAAATCACCTGTGAAGCCGATGGCCAGTGGAGCTCTGGGTTCCCCCACTGTGAACACACTTCTTGTGGTTCTCTTCCAATGATACCAAATGCGTTCATCAGTGAGACCAGCTCTTGGAAGGAAAATGTGATAACTTACAGCTGCAGGTCTGGATATGTCATACAAGGCAGTTCAGATCTGATTTGTACAGAGAAAGGGGTATGGAGCCAGCCTTATCCAGTCTGTGAGCCCTTGTCCTGTGGGTCCCCACCGTCTGTCGCCAATGCAGTGGCAACTGGAGAGGCACACACCTATGAAAGTGAAGTGAAACTCAGATGTCTGGAAGGTTATACGATGGATACAGATACAGATACATTCACCTGTCAGAAAGATGGTCGCTGGTTCCCTGAGAGAATCTCCTGCAGTCCTAAAAAATGTCCTCTCCCGGAAAACATAACACATATACTTGTACATGGGGACGATTTCAGTGTGAATAGGCAAGTTTCTGTGTCATGTGCAGAAGGGTATACCTTTGAGGGAGTTAACATATCAGTATGTCAGCTTGATGGAACCTGGGAGCCACCATTCTCCGATGAATCTTGCAGTCCAGTTTCTTGTGGGAAACCTGAAAGTCCAGAACATGGATTTGTGGTTGGCAGTAAATACACCTTTGAAAGCACAATTATTTATCAGTGTGAGCCTGGCTATGAACTAGAG |
| circRNA025171 | GGCTCCAGCGGGGCCCCACAGACGAGCAGGATGCCTGTCCCCATGAGTGCCAAGAACAGACCCGGAACCCTGGACAAACCCGGCAAGCAGTCCAAACTGCAGGATCCCCGCCAATATCGTCAGGTAGTTTTACCTTAAACCCACTTTTGGATGGACGCTATTTCAGTTAAGCAAGTCACTGACTTAGTTTATACCAAATATTGTGCTTTCTTTGTAAGATAACGGTTTACATAGACATCCTGGATCTGGGGGCATGAAGAAAGTCTAAATAAACCTTTGTTACACTTTTTTACCACGCTTTTGCATGCTTGCAATAAAACATCTTTTACTTTGTGACTCCAAACTCCAAATTTTAACTGTTAACACACGGTGCCAGACCAGGTGGCTTTTCTTTGGTGAATGTAGTGTTTCATCTGAACACCTCGGGAAGCAGAGACAAACCAACCTGTGGTTGAACTGCCCTTAACGTCACCACTACTAACGTCTATGTTGACTGTATTGTGTTAGAAGCACATTAACACTCCGTCACAATGCCCGACCCCCACCCCAGTAATTATCCAGACGCATGGCCCACCTGGCACACAGGAAATGGTAGAGCTGGAATGATGGGACTCCTCTCACAAATGTATTCTTCCTTTCCTCCTTTCCCGTCCATCCTTTGCTATGTACATGGGGGGTTTCTACCAGGTCCAGTAGAGCACAACAAGACTTAGCTCAGGCCTTGAACTGTGTTTGGTTGGTTTTCTTTGATTGAATTATTCTCAGAAGGGCTGTGTTGCCAGGCCCTGTGGGTTGATCATGTGACCGCCTTTCTGACAAAATGTCTCCCGCCATCTTTATTTGCAGGCTAATGGAAGTGCTAAGAAATCTG |
| circRNA026414 | CAATGACTCAGATGCATATAAAGATCAAATATCAGTACTGCCAAATGAACAAGACTTGGTGAGAGAAGAAGCCCAGAAAATGAGTAGTCTTTTACCAACTATGTGGCTTGGAGCTCAAAATGGCTGTTTGTATGTCCATTCATCTGTAGCCCAGTGGAGGAAATGTCTCCATTCCATTAAACTTAAAGATTCGATTCTCAGTATTGTACACGTGAAGGGAATCGTGTTAGTAGCCCTGGCTGACGGCACCCTTGCAATCTTTCACAGAGGAGTGGATGGGCAGTGGGATTTGTCAAACTATCACCTCTTAGACCTTGGACGGCCTCATCATTCCATCCGTTGCATGACTGTGGTACATGACAAAGTCTGGTGTGGCTATAGGAACAAAATCTATGTGGTGCAGCCAAAGGCCATGAAAATAGAGAAATCTTTTGATGCACATCCCAGGAAGGAGAGCCAAGTGCGACAGCTTGCGTGGGTGGGGGATGGCGTGTGGGTCTCCATTCGCTTGGATTCTACGCTCCGTCTCTATCATGCACACACTTATCAACATCTACAGGATGTGGACATTGAGCCTTATGTAAGCAAAATGTTAGGTACTGGAAAACTGGGCTTCTCTTTTGTGAGAATTACAGCTCTTATGGTGTCTTGTAATCGTTTGTGGGTGGGGACAGGAAATGGTGTCATTATCTCCATCCCATTGACAGAAACAAATAAAACCTCAGGTGTACCAGGAAATCGTCCTGGAAGTGTAATCCGTGTATATGGTGATGAAAACAGTGATAAAGTGACTCCAGGGACATTTATACCCTATTGTTCAATGGCACATGCACAGCTTTGCTTCCATGGGCACCGGGATGCTGTGAAATTCTTTGTGGCAGTCCCAGGTCAAGTCATCAGCCCACAAAGTAGCAGTAGTGGCACGGATCTGACGGGTGACAAAGCAGGGCCATCTGCACAGGAGCCTGGTAGTCAGACGCCCTTGAAGTCTATGCTTGTCATCAGTGGAGGAGAGGGCTACATCGACTTCCGAATGG |
| circRNA026955 | ATGTTGATGAATGCCAGGCTATCCCAGGGATATGCCAAGGAGGAAACTGTATCAATACAGTGGGCTCTTTTGAATGCAGATGCCCTGCTGGTCACAAACAGAGTGAAACTACTCAGAAATGTGAAGACATTGATGAGTGCAGCATCATTCCTGGGATATGTGAAACTGGTGAATGTTCCAACACCGTGGGAAGCTATTTTTGTGTTTGTCCACGTGGATATGTAACCTCAACAGATGGCTCTCGATGCATCGATCAGAGAACAGGCATGTGTTTCTCGGGCCTGGTGAATGGCCGCTGTGCACAAGAGCTCCCGGGGAGAATGACGAAAATGCAGTGCTGCTGTGAGCCTGGCCGCTGCTGGGGCATCGGAACCATTCCTGAAGCCTGTCCTGTCAGAGGTTCTGAGGAATATCGCAGACTTTGCATGGATGGACTTCCAATGGGAGGAATTCCAGGGAGTGCTGGTTCCAGACCTGGAGGCACTGGGGGAAATGGCTTTGCCCCAAGTGGCAATGGCAATGGCTATGGCCCAGGAGGGACAGGCTTCATCCCCATCCCTGGAGGCAATGGCTTTTCTCCTGGCGTTGGGGGAGCCGGTGTGGGGGCCGGGGGACAGGGACCTATCATCACTGGACTAACAATTCTGAACCAGACAATAGATATCTGTAAGCATCATGCTAACCTTTGTTTAAATGGACGCTGTATACCAACTGTCTCAAGCTACCGATGTGAATGCAACATGGGTTATAAGCAGGATGCAAATGGAGATTGTATAG |
| circRNA028717 | ATAGACCCAGCCCCATTTCTCTATCACAAGCCAGCTTAGCAGCCTGTGGTGGCAGTATTTCAAGGAGATCGCTGGCCTGGAGCTGTGGAGCCAGAGCTTCTGGAAGAGGGAATGTTCTTAGAACTCCATGGATTTAAAAAGAAGATTTGGCCGGGCATGGTGCCTCAGGCCTGCAATCCCAGTACTTTGGGAGGCCAAGTGCCTGGATCAAAGTGGAACAGCTGAAGCCATATCATGCTCATAAAGAGGAAATGATAAAAATTAACAAGGGTAAACGATTCCAGCAAGCGGTAGATGCTGTCGAAGAGTTCCTCAGGAGAGCCAAAGGGAAAGACCAGACGTCATCCCACAATTCTTCTGATGACAAGAATCGACGTAATTCCAGTGAGGAGAGAAGTAGGCCAAACTCAGGTGATGAGAAGCGCAAACTTAGCCTGTCTGAAGGGAAGGTGAAGAAGAACATGGGAGAAGGAAAGAAGAGGGTGTCTTCAGGCTCTTCAGAGAGAGGCTCCAAATCCCCTCTGAAAAGAGCCCAAGAGCAAAGTCCCCGGAAGCGGGGTCGGCCCCCAAAGGATGAGAAGGATCTCACCATCCCGGAGTCTAGTACCGTGAAGGGGATGATGGCCGGACCGATGGCCGCGTTTAAATGGCAGCCAACCGCAAGCGAGCCTGTTAAAGATGCAGATCCTCATTTCCATCATTTCCTGCTAAGCCAAACAGAGAAGCCAGCTGTCTGTTACCAGGCAATCACGAAGAAGTTGAAAATATGTGAAGAGGAAACTGGCTCCACCTCCATCCAGGCAGCTGACAGCACAGCCGTGAATGGCAGCATCACACCCACAGACAAAAAGATAGGATTTTTGGGCCTTGGTCTCATGGGAAGTGGAATCGTCTCCAACTTGCTAAAAATGGGTCACACAGTGACTGTCTGGAACCGCACTGCAGAGAAA |
| circRNA03251 | AATGAAAACCATGTAGCTGGTCTTATTATTCTCTAATCATGAAGACCATTATTGTTATTTGTGGACTAAGGAGCAGAAAGACTGATAGTGGACATAGGCCAGCTGATTTAATAATTTAAGAAAGAACTGTTATCCTGAGTAAGGAGCATAGAGACAAATATAAAATGAACGCACTCTACCTGATTTAATTATAGTCTTCTGGTGAAGATCTTGCTGTTATCTCTGTGAATCAGGAGAGAAGAGATTGATAAGGAATAGACGAGATTGGATTTGGTCATTTCCCCAAGGTGAAGAGCATGGCGATTTCTTCTGTGGTAAGGAGCAAT |
| circRNA032706 | TACTCATGTAATGGAGGAAAGTCTATATCTGCAGCTGGACCCAGCCATACCAGGAGTGTATTTTGGAAATCCATACCCGTTTGGGATTGATCCGATTTGGAACTTGGCTTCAAACAAACTCACATTTCTGAACTCGTATAAAATGAAGATGTCGGTGATCCTGGGAATTGTCCAGATGGTTTTCGGTGTCATCCTCAGCCTTTTCAATCACATATACTTCAGAAGAACTCTCAACATCATTCTGCAATTTATCCCTGAGATGATTTTTATCCTGTGTCTGTTTGGATACCTGGTTTTCATGATCATTTTCAAATGGTGCTGCTTTGACGTCCATGTATCTCAGCACGCCCCCAGCATCCTCATCCACTTCATCAACATGTTTCTGTTTAACTACAGTGACTCTTCCAACGCACCCCTCTACAAACATCAGCAAGAAGTCCAAAGTTTCTTTGTGGTTATGGCTTTGATTTCTGTGCCGTGGATGCTTCTGATTAAGCCGTTTATTCTTAGAGCCAGTCATCGGAAATCCCAG |
| circRNA032774 | ATTGCCCAGAATGCACGCTACAGGAAAACCCATTCTTCTCCCAGCCGGGTGCCCCAATACTTCAGTGCATGGGCTGCTGCTTCTCTAGAGCATATCCCACTCCACTAAGGTCCAAGAAGACGATGTTGGTCCAAAAGAACGTCACCTCAGAGTCCACTTGCTGTGTAGCTAAATCATATAACAGG |
| circRNA033514 | ATGGCCTGATATGAAGGAGTCACGCCTCCCGCCTCCCGGAGCTGCCCAGTGGCTGCCTTGTCCTTCAAGTGCAGGAGCTGGTTCAAATGTCAGGAATGGAAGCCACTGTGACCATCCCAATCTGGCAAAACAAGCCACATGGGGCTGCTCGAAGTGTAGTAAGAAGAATTGGGACCAACCTACCCTTGAAGCCGTGTGCCCGGGCGTCCTTTGAG |
| circRNA03380 | GCTGGACCGCTTTGAGAAGACCAATGAGATGCTGCTCAACTTCAACAACCTGTCCAGTGCCCGCCTGCAGCAGATGAGCGAACGCTTCCTGCACCACACGAGGACCCTAGTAGAGATGAAACGGGACCTGGACAGCATCTTCCGCCGTATCAGGCTTCACTGCGCACTTAAGTGTCTGGGACAATTCCAACCCTGGGATAAAATCTTGAC |
| circRNA034136 | GGACCCTCAGGGGCGGCACGTAAAAACCTATGAGGTGTCTCTCCGAGAAAAGGAATTCAATAAGGGCCCTTGGAAACAGGAAAATGTCGAAGCTGAAGCTTCCATGGTGATCGCAGTCCCAGAGCCCTTTGGGGGGGCCATCATCATTGGACAGGAGTCAATCACCTATCACAATGGTGACAAATACCTGGCTATTGCCCCTCCTATCATCAAGAATGACGACATATCAGTAAACATATAAGATATATTGATGGAGCGCCTCAGAAAATTATCTTCAAAATAAAAAAGCTCCCCATTTTCTATTAATGGGCAATAGGATCTATCCTCATTTTCATGTAATGTTATCTGAGCGATTTGTCTGCTGCAAAGCACGATTGTGTGCCACAATCGAGTGGACCCTAATGGCTCAAGATACCTGCTGGGAGACATGGAAGGCCGGCTCTTCATGCTGCTTTTGGAGAAGGAGGAACAGATGGATGGCACCGTCACTCTCAAGGATCTCCGTGTAGAACTCCTTGGAGA |
| circRNA03459 | ATGGTCCAGACCTCCCCAGAATTTACCCTTCATTCACCTATTACCGTTCAGGAGAAGTCCTCTACTTGTCCTGTTCTGCGGACTCTAACCCACCGGCACAGTATTCTTGGACAATTAATGAAAAGTTTCAGCTACCAGGACAAAAGCTCTTTATCCGCCATATTACTACAAAGCATAGCGGGCTCTATGTTTGCTCTGTTCGTAACTCAGCCACTGGCAAGGAAAGCTCCAAATCCATGACAGTCGAAGTCTCTG |
| circRNA03460 | ATGGTCCAGACCTCCCCAGAATTTACCCTTCATTCACCTATTACCGTTCAGGAGAAAACCTCGACTTGTCCTGCTTTGCGGACTCTAACCCACCGGCAGAGTATTCTTGGACAATTAATGGGAAGTTTCAGCTATCAGGACAAAAGCTCTTTATCCCCCAAATTACTACAAATCATAGCGGGCTCTATGCTTGCTCTGTTCGTAACTCAGCCACTGGCAAGGAAATCTCCAAATCCATGATAGTCAAAGTCTCTG |
| circRNA03462 | GTGGGAGATGCCAACTCTGAATGAAGGATGCCTGTGGAGGAATCAAAGGTGCCACACAGGACAATCTTCTGTTATCCACACAGCGAAGCTGCCCATGCCTTACATCACCATCAACAACTTAAACCCCAGGGAGAAGAAGGATGTGTTAGCCTTCACCTGTGAACCTAAGAGTCGGAACTACACCTACATTTGGTGGCTAAATGGTCAGAGCCTCCCGGTCAGTCCGAGGGTAAAGCGACCCATTGAAAACAGGATACTCATTCTACCCAGTGTCACGAGAAATGAAACAGGACCCTATCAATGTGAAATACGGGACCGATATGGTGGCATCCGCAGTAACCCAGTCACCCTGAATGTCCTCTATGGTCCAGACCTCCCCAGAATTTACCCTTCATTCACCTATTACCGTTCAGGAGAAAACCTCGACTTGTCCTGCTTTGCGGACTCTAACCCACCGGCAGAGTATTCTTGGACAATTAATGGGAAGTTTCAGCTATCAGGACAAAAGCTCTTTATCCCCCAAATTACTACAAATCATAGCGGGCTCTATGCTTGCTCTGTTCGTAACTCAGCCACTGGCAAGGAAATCTCCAAATCCATGATAGTCAAAGTCTCTG |
| circRNA03465 | TGGAGACTCCCAAGCCCTCCATCTCCAGCAGCAACTTAAACCCCAGGGAGGCCATGGAGACTGTGATCTTAACCTGTAATCCTGAGACTCCGGACGCAAGCTACCTGTGGTGGATGAATGGTCAGAGCCTCCCTATGACTCATAGGATGCAGCTGTCTGAAACCAACAGGACCCTCTTTCTATTTGGTGTCACAAAGTATACTGCAGGACCCTATGAATGTGAAATATGGAACTCAGGGAGTGCCAGCCGCAGTGACCCAGTCACCCTGAATCTCCTCC |
| circRNA03467 | TGGAGACTCCCAAGCCCTACATCTCCAGCAGCAACTTAAACCCCAGGGAGGCCATGGAGGCTGTGCGCTTAATCTGTGATCCTGAGACTCTGGACGCAAGCTACCTATGGTGGATGAATGGTCAGAGCCTCCCTGTGACTCACAGGTTGCAGCTGTCCAAAACCAACAGGACCCTCTATCTATTTGGTGTCACAAAGTATATTGCAGGACCCTATGAATGTGAAATACGGAACCCAGTGAGTGCCAGTCGCAGTGACCCAGTCACCCTGAATCTCCTCC |
| circRNA034835 | GGCTGAGGGAGGAAGACGGCATGGTTACATGGGACACCTAACGAGGATAGCTAACTGTATCGTGCACAGCACTGACAAGGGCCCCAACAGTGCATTAGTGCAGCAGCTTATCAAAGATCTTCCCGACGAAGTCAGGGAACGATGGGAGACGTTCTGCACAAGCTCCTTAGGAGAAACTAACAAGAGGAACACGGTAGATCTA |
| circRNA03512 | GGATACCTCAGACAATGGCAGCTTTTGGATTGAAATATTTGCTGAATGACACTGGATACACAAGCAGCAAGTCCAACACAATCACAGCAACAGACTCTCCTGCAGACCTTCCCAGGAAGCCAGAGCCACACACACCATCATGGTCCTG |
| circRNA03556 | GCCCCTTCACAGATGTAGTCACTACAAATCTTAAATTGCGAAATCCATCGGATAGAAAAGTGTGTTTCAAAGTGAAGACTACAGCACCTCGCCGGTACTGTGTGAGGCCCAACAGTGGAATTATTGACCCAGGGTCAACTGTGACTGTTTCAGTAATGCTACAGCCCTTTGACTATGATCCGAATGAAAAGAGTAAACACAAGTTTATGGTACAGACAATTTTTGCTCCACCAAACACTTCAGATATGGAAGCTGTGTGGAAAGAGGCAAAACCTGATGAATTAATGGATTCCAAATTGAGATGCGTATTTGAAATGCCCAATGAAAATGATAAATTGAATGATATGGAACCTAGCAAAGCTGTTCCACTGAATGCATCTAAGCAAGATGGACCTATGCCAAAACCACACAGTGTTTCACTTAATGATACCGAAACAAGGAAACTAATGGAAGAGTGTAAAAGACTTCAGGGAGAAATGATGAAGCTATCAGAAGAAAATCGGCACCTGAGA |
| circRNA036250 | GGCTGGTGAATTACCAGATCTCCATCAAGTGCAGTAACCAGTTCAAGTTGGAAGTGTGTCTTTTGAATGCAGAAAACAAAGTCGTGGACAACCAGGCTGGGACCCAGGGCCAGCTGAAGGTGCTGGGTGCCAACCTCTGGTGGCCGTACCTGATGCACGAACACCCCGCCTACCTGTACTCGTGGGAGGTGCGGCTGACTGCACAGAAGTCACTGGGGCCTTTGACTTCTACACACTCCCTGTGGGGCTCCGCACTGTGCCCGTCACCGAGAGCCAGATGGTGATTGCTCACACCAAAGCCTTGGACCCCTCCCAGCCTGTGACCTTTGTGACCAACGTCACCTATGCAGCAGACAAGGGGGCTCTGTATGTGGATGTGATCCGTGTGAACAGCTACTACTCTTGGTATCGCAACTACGGGCACCTGGAGTTGATTCAGCTGCAGCTGGCCACCCAGTTTGAGAATTGGTGTAAGACATCACAATCCCATTATTCAGAGCGCGTATGGAGTGGAAACGCTTGTAGGGTTTCACCAGGATCCACCTCTGATGTTCAGTGAAGAGTACCAGAAAAGTCTGCTAGAGCAGTACCATCTGGGTCTGGATCAAAAACGCAGAAAATACGTGGTAGGAAAGCTCATCTGGAATTTTGCCGATTTCATGACTAACCAGT |
| circRNA036366 | GGCTGGTGAATTACCATATCTCCGTCAAGTGCAGTAACCAGTTCAAGTTGGAAGTGTGTCTTTTGAAATCAGACAACAAAGTCGTGGACAACCAGGCTGGGACCCAGGGCCAGCTGAAGGTGCTGGGTGCCAACCTCTGGTGGCCGTACCTGATGCACGAACACCCCGCCTACCTGTACTCGTGGGAG |
| circRNA03794 | GCTAGCTTGTCACTTTCTGCAAAGGTTTCCCTCAGGGAGCCTCCTGCTGCCAGGCACCATGACAGTGAGGGGGGATGTGCTGGCCCCGGATCCAGCGTCGCCCACGACCGCAGCAGCCTCGCCCAGCGTCTCCGTGATCCCCGAGGGCAGCCCCACTGCCATGGAGCAGCCTGTGTTCCTGATGACAACTGCCGCTCAGGCCATCTCTGGCTTCTTCGTGTGGACGGCCCTGCTCATCACATGCCACCAGATCTACATGCACCTGCGCTGCTACAGCTGCCCCAACGAGCAGCGCTACATCGTGCGCATCCTCTTCATCGTGCCCATCTACGCCTTTGACTCCTGGCTCAGCCTCCTCTTCTTCACCAACGACCAGTACTACGTGTACTTCGGCACCGTCCGCGACTGCTATGAGG |
| circRNA038715 | CTGTGACTAATACTACTGTAGAGGATGAGACTGAAACAAATGAAGTTCAAGGATTTCTCTTTGGGAAACTAAAAGAAATATATTCAGATCTTAGAGATAATCTGACAGCATTCCAAAAATACCTGATTGAGAGTAACAAACAAATGATGCCTTTGAAAGTCTGGGAACTACAAGATCTTAGTTTTCAAGCAGCTTCTCAAATAATGTCCGCTCCAGTTTATGATTCCATTAAATTAATGAAAGACATTTCACAGAACTTCCCCATAAAAGCCAG |
| circRNA0412 | ACGATGTGACTCAAGCGACTCCAGAAACATTCACAGAAGATCCTAATCTGGTGAATGATCCCGCTACAGATGAAACAGTTTTGGCTGTTTTGGCTGATATTGCACCTTCCACAGATGACTTGG |
| circRNA042331 | GGATTTGAGAGCACCTCCAGAACAAGGAAAGATTTTTATTGCAAGGCGCTCTCTCTTAGATGAACTGCTTGAAGTGGACCACATCAGAACAATATATCACATGTTTATTGCCCTCCTCATTCTCTTTATCCTCAGCACACTTGTAGTAGATTACATTGATGAAGGAAGGCTGGTGCTTGAGTTCAGCCTCCTGTCTTATGCTTTTGGCAAATTTCCTACCGTTGTTTGGACCTGGTGGATCATGTTCCTGTCTACATTTTCAGTTCCCTATTTTCTGTTTCAACATTGGGCCACTGGCTATAGCAAGAGTTCTCATCCGCTGATCCGTTCTCTCTTCCATGGCTTTCTTTTCATGATCTTCCAGATTGGAGTTCTAGGTTTTGGACCAACATATGTTGTGTTAGCATATACACTGCCACCAGCTTCCCGGTTCATCATTATATTCGAGCAGATTCGTTTTGTAATGAAGGCCCACTCATTTGTCAGAGAGAACGTGCCTCGGGTACTAAATTCAGCTAAGGAGAAATCAAGCACTGTTCCAATACCTACAGTCAACCAGTATTTGTACTTCTTATTTGCTCCTACCCTTATCTACCGTGACAGCTATCCCAG |
| circRNA043194 | GTTCAGTGATCGGATTTTGTGGTTGGTTGATATTCCTAGAGAAAACATCACACAAAGCACAGATATTGCAGCTGTAGAAGAATGGTTAGTAAGAATCACTTTACATCATGGACTAAATATTTATGCTACTGAAGGAACTCTATTGGATGTTATTCGAGAACCGATTCTTCAGTGGACTCCTGGGGATGTGATTCCAGAAAGTGAAATCAGTAAATTATATCCACATGTGGTAGATCTCAAAGTGACAAAATGCCCCTGTGCCAATGATGTGGCATTACTAGGCTTCATTGTGGATACAATAGTTGATGGTGTTTACATAGGCATAACCTTTGGTGGATTCTGGCATGATTATGATACCACATGGTTTAACATGACACAGACTATCTATTCCCAACTTCAAGAAGAATATGAAGACCTTTCATTGGTGGATATGGTTTTAACGAATCATTTTTTAGTTATCCTCACCTCTTTGGGCCTTTTTGTAAGTGAAGATCTTCGTTATCCATCACGCCACAGCTTATC |
| circRNA04411 | TCCATGCTCCGAGGGAGAGGAGGTTGTTACAAACACACATTCTATGGAATGAGAGCCATCGCTGCATGGAAACCACCCCGAGCTTGGCGTGTGCTAATAAATGTGTCTTCTGTTGGCGGCACCACACCAACCCCGTGGGCACTGAGTGGCGGTGGAAGATGGACCAGCCTGAAATGATCTTGAAGGAAGCCATTGAAAACCATCAGAACATGATTAAGCAGTTTAAAG |
| circRNA04417 | TCGATGCTCCGAGGGAGAGGAGCTTGTTACAAACACACATTCTATGGAATGAGAGCCATCGCTGCATGGAAACCACCCCGAGCTTGGCGTGTGCTAATAAATGTGTCTTCTGTTGGTGGCACCACAACAACCCTGTGGGCACTGAATGGTTGTGGAAGATGGACCAGCCTGAAATGATCTTGAAGGAAGCCATTGAAAACCATCAGAACATGATTAAGCAGTTTAAAG |
| circRNA05232 | GGCTGGTGAATTACCATATCTCCGTCAAGTGCAGTAACCAGTTCAAGTTGGAAGTGTGTCTTTTGAAATCAGACAACAAAGTCGTGGACAACCAGGCTGGGACCCAGGGCCAGCTGAAGGTGCTGGGTGCCAACCTCTGGTGGCCGTACCTGATGCACGAACACCCCGCCTACCTGTACTCGTGGGAGGATGGTGATTGCTCACACCAAAGCCTTGGACCCCTCCCAGCCTGTGACCTTTGTGACCAACTCCACCTACGCAGCAGACAAGGGGGATCCACCTCTGATGTTCAGTGAAGAGTACCAGAAAAGTCTGCTAGAGCAGTACCATCTGGGTCTCAATCAAAAACGCAGAAAATACGTGGTTGGAGAGCTCATCTGGAATTTTGCCGATTTCATGACTAACCAGT |
| circRNA05233 | GGCTGGTGAATTACCATATCTCCGTCAAGTGCAGTAACCAGTTCAAGTTGGAAGTGTGTCTTTTGAAATCAGACAACAAAGTCGTGGACAACCAGGCTGGGACCCAGGGCCAGCTGAAGGTGCTGGGTGCCAACCTCTGGTGGCCGTACCTGATGCACGAACACCCCGCCTACCTGTACTCGTGGGAGGATGGTGATTGCTCACACCAAAGCCTTGGACCCCTCCCAGCCTGTGACCTTTGTGACCAACTCCACCTACGCAGCAGACAAGGGG |
| circRNA05260 | AATGGGAGTGAGGCCCTAATGATTCTAGATGACCAAACTAACAAACTGAAATCGGAAAGCAAAGATATCCTGTTAGTGGATCTAAACTCTGAAATCGACACCAATCAGAATTCTTTAAGAGAAAATCCATTCTTAACAAACGGCATCACCTCCTGTTCTCTTCCTCGACCAACGCCTCAGGCATCCTTCTTGCCTGAAAATGCCTTTTCTGCCAATCTCAACTTCTTTCCCACCCCTAATCCTGATCCTTTCCGTGACGATCCTTTCACACAGCCAGACCAATCGACACCTTCTTCGTTTGATTCTCTCAAATCTCCAGATCAGAAGAAAGAGAATTCGAGTAGCTCGTCTACTCCGCTGAGTAATGGGCCCCTGAATGGTGATGTTGACTACTTTGGTCAGCAATTTGACCAGATCTCTAACCGGACTGGCAAACAGGAAGCTCAGGCAGGCCCATGGCCCTTTTCAAGTTCGCAAACCCAGCCAGCAGTGAGAACTCAAAATGGGGTATCTGAAAGAGAACAGAACGGCTTCTCTGTCAAATCCTCCCCGAACCCTTTTGTGGGAAGCCCTCCCAAAGGACTGTCCATACAGAATGGCGTAAAGCAGGACTTGGAAAGCTCTGTCCAGTCCTCACCACATGACTCCATAGCCATTATCCCACCTCCACAAAGTACCAAACCAGGAAGAGGCAGAAGGACTGCTAAG |
| circRNA05485 | CCTAGGCAAGGTACCTCTCAAGAGGGCCTTTGATTTGATTAATTATCTTGGAAATGAGAACCATACTGCACCCATCACCGAAGCCCTGTTTCAGACAGACCTCATCTATAACCTCCTTGAAAAACTGGGATACATGGATCTGGCCTCAAGACTGGTGACTAGGGTATTTAAATTACTTCAAAACCAAATTCAACAACAAACTTGGACTGATGAGGGCACTCCATCTATGCGAGAGCTTCGGTCAGCCCTGCTAGAGTTTGCTTGCACCCACAACCTGGGGAACTGCTCTACTACTGCCATGAAACTGTTTGATGACTGGATGGCATCCAATGGAACTCAAAG |
| circRNA05938 | GTGGGTTTGGAGGATTTGGGACAACATCTACAACTGCAGGTTCTGCATTCAGCTTTTCTGCCCCAACTAACACAGGCACTACTGGACTCTTTGGTGGTACTCAGAACAAAGGTTTTGGATTTGGTACTGGTTTTGGCACAACAACGGGAACTAGTACTGGTTTAGGTACTGGTTTGGGAACTGGACTGGGATTTGGAGGATTTAATACACAGCAGCAGCAGCAAACTAGCAGTAGGTTATAGTTGCATGCCCAGTAATAAAGATGAAGATGGGCTAGTGGTTTTAGTTTTCAACAAAAAAGAAACAGAGATTCGAAGCCAACAACAACAGTTGGTAGAATCATTGCATAAAGTTTTGGGAGGAAACCAGACCCTTACTGTAAATGTAGAGGGCACTAAAACATTGCCAGATGATCA |
| circRNA06073 | TTCCAAAGAACTACTGGTTCAGATTTTAGCTCTTCCCAAGTTGTGAAAATAGTGAAGGATGCTTAGACTACTTAACATACAAACTGCTTTCTGGTTAATCATCTTTAGAAGACTGGATTTCTGGATATCTACTCCACTCCATCTCTATTGACTTTTAAAACATGATAATGCAAACCTATAACACTGGCAACCATCAGTGAACCTTTAATTTCATTGATTAATAGCGTTTGAAGCTTCCTCAGGGAATAACAATGACATCAGCAGTGGTTGACAGTGGAGGTACTATTTTGGAGCTTTCCAGCAATGGAGTAGAAAATCAAGAGGAAAGTGAAAAGGTTTCTGAATATCCAGCAGTGATTGTGGAGCCAGTTCCAAGTGCCAGATTAGAGCAGGGCTATGCAGCCCAGGTTCTGGTTTATGATGATGAGACTTATATGATGCAAGATGTGGCAGAAGAACAAGAAGTTGAGACCGAGAATGTGGAAACAGGTGGAATCTCACTCTGTCACCCAGGCTGGAGTGCGGTGGTGTGATCTCGGCTCACTGCAACCTCCGCCTCCCGGGTTCAAGTGATTCTCCTGCCTCAGCCTCCCAAGTGGCTGGGACTACAGAAATCCTCCCTGTGGCACAAAAGGACTCCTTTAATTAGAAAATATTGTTGCCACAGGGAGGTAGAAGATCGACATGAAAAACTGGGGATCTGAAAACGAGCCCCTATCCTAGGATTACCTGAAGGTGAAACAGATTGAGCCAGAAGGAAGACAATGCTG |
| circRNA06307 | CACCTGAGACTTTTACCTTTACCCAGAAAGGAATAAAAGAGGTCAGATGATGACAGCTGTGTCCTTAACAACCAGGCCCCAGGAATCAGTGGCTTTTGAGGACGTGGCTGTGTACTTCACTACGAAGGAATGGGCCATCATGGTGCCTGCCGAGAGGGCCTTGTACAGGGATGTGATGCTGGAGAACTATGAGGCTGTGGCCTTTGTAGTGCCACCCACTTCCAAACCAGCTTTGGTCTCTCATCTGGAGCAAGGGAAAGAGTCCTGTTTCACCCAGCCACAGGGAGTCCTAAGCAGGAATGACTGGAGAGCAGGCTGGATAG |
| circRNA06432 | AAAACCTCGGATCAATTCTCAGCTGGTGGCACAACAAGTGGCACAACAGTATGCCACCCCACCACCCCCTAAAAAGGAGAAGAAGGAGAAAGTTGAAAAGCAGGACAAAGAGAAACCTGAGAAAGACAAGGAAATTAGTCCTAGTGTTACCAAGAAAAATACCAACAAGAAAACCAAACCAAAGTCTGACATTCTGAAAGATCCTCCTAGTGAAGCAAACAGCATACAGTCTGCAAATGCTACAACAAAGACCAGCGAAACAAATCACACCTCAAG |
| circRNA06493 | GATGCTGACCTGTTGGATGTTGAAAGCAAACACTTTGAAGACCTGGAGTTCCAGCAGCTTGAACATGAGAGCCGTCTAGATGAAGAAAAGGAGAACTTGACTCAACAGCTCCTGCGTGAAGTTGCTGAATATCAACGGAACATCGTTTCTAGAAAGGAAAAAATTTCTGCATTGAAAAAGCAAGCCAATCACATTGTTCAGCAGGCTCAGAGAGAGCAAGATCATTTTGTGAAAGAAAAGAATAATTTAATAATGATGTTGCAAAGA |
| circRNA07333 | GCTCAGTTATTGGGACAGAGGCTGATGAAGAAAGACCTGAAGGAAGGCCCTAGTCTGTGTGGTCTCTGTTTGGAGGAGCTAAAAGGGGACTTTTTGGTCGAGAGACTCAGAGAGAATATCCCTGCCGTAAGGAGAAAACCAAAAGCCAAGGCACCACTTCCTCCAGCTGAGACCAAATATACTGATGTCTCTTCAGCTGCTGATTCTGTAGAATCCACTGCTTTCATCATGGAACAGAAAGAAAACATGATAGATAAAGACGTTGAACTCTCAGTGGTCCTACCTGGGGATATTATCAAATCTACTACTGTTCATGGCAG |
| circRNA07735 | GTAGAGTACAGAGAGATGGATGAAAGCTTGGCCAACCTCTCAGAAGATGAGTATTATTCAGAAGAAGAGAGAAATGCCAAAGCAGAGAAGGAAAAGAAGCTTCCCCCACCACCCCCTCAAGCCCCACCTGAGGAAGAAAATGAAAGTGAGCCTGAAGAACCATCGGGTGTGGAGGGCGCAGCTTTCCAGAGCCGACTTCCTCATGACCGGATGACTTCTCAAGAAGCAGCCTGTTTTCCAGATATTATCAGTGGACCACAACAGACCCAGAAGGTTTTTCTTTTCATTAGAAACCGCACA |
| circRNA08204 | TTCCAAGATGAGATGGGATTCTCCAACATGGAAGATGATGGCCCAGAAGAGGAGGAGCGTGTGGCTGAGCCTCAAGCTAACTTTAACACCCCTCAAGCTCTACGGTTTGAGGAACTACTGGCCAACCTACTAAATGAACAACATCAGATAGCGAAGGAACTATTTGAACAGCTGAAGATGAAGAAACCTTCAGCCAAACAGCAGAAGGAGGTAGAGAAGGTTAAACCCCAGTGTAAGGAAGTTCATCAGACCCTGATTCTGGACCCAGCACAAAGGAAGAGACTCCAGCAGCAGATGCAGCAGCATGTTCAGCTCTTGACACAAATCCACCTTCTTGCCACCTGCAACCCCAATCTCAATCCGGAGGCCAGTAGCACCAGGATATGTCTTAAAGAGCTGGGAACCTTTGCTCAAAGCTCCATCGCCCTTCACCATCAGTACAACCCCAAGTTTCAGACCCTGTTCCAACCCTGTAACTTGATGGGAGCTATGCAGCTGATTGAAGACTTCAGCACACATGTCAGCATTGACTGCAGCCCTCATAAAACTGTCAAGAAGACTG |
| circRNA08839 | AGCCTACTTGGATGTTAATGAGCTGAAGAACATTCTTAAATTGGATGGATCAACACATCTCAATATTTTCTTTGCAAAATCCTCAGAGGAGGAGTTGGCAGGAGTAGCAACTTGGCCATGGGACAAGGAGGCCCTGATGCACTTAGGTGGCATTGTCTTGAACCCATCTTTCTATGGCATGCCTGGGCACACCCACACCATGATCCATGAGATTGGTCACAGCCTGGGCCTCTATCACGTCTTCCGAGGCATCTCAGAAATCCAGTCCTGCAGTGACCCCTGCATGGAGACAGAGCCCTCCTTCGAGACTGGAGACCTCTGCAATGATACCAACCCAGCCCCTAAACACAAGTCCTGTGGTGACCCAGGGCCAGGAAATGACACCTGTGGCTTTCATAGCTTCTTCAACACTCCTTACAACAACTTCATGAGCTATGCAG |
| circRNA08848 | ATGACGACTGTACGGACTCCTTCACGCCCAATCAAGTCGCCAGAATGCACTGTTACCTGGACCTGGTCTACCAGGGCTGGCAGCCCTCCAGGAAACCAGCGCCTGTTGCCCTCGCCCCCCAAGTTCTGGGCCACACAACGGACTCTGTGACACTGGAGTGGTTCCCACCTATAGATGGCCATTTCTTTGAAAGAGAATTGGGATCAGCATGTCATCTTTGCCTGGAAGGGAGAATCCTGGTGCAGTATGCTTCCAACGCTTCCTCCCCAATGCCCTGCAGCCCATCAGGACACTGGAGCCCTCGTGAAGCAGAAGGTCATCCTGATGTTGAACAGCCCTGTAAGTCCAGTGTCCGCACCTGGAGCCCAAATTCAGCTGTCAACCCACACACGGTTCCTCCAGCCTGCCCTGAGCCTCAAGGCTGCTACCTCGAGCTGGAGTTCCTCTACCCCTTGGTCCCTGAGTCTCTGACCATTTGGGTGACCTTTGTCTCCACTGACTGGGACTCTAGTGGAGCTGTCAATGACATCAAACTGTTGGCTGTCAGTGGGAAGAACATCTCCCTGGGTCCTCAGAATGTCTTCTGTGATGTCCCACTGACCATCAGACTCTGGGACGTGGGCGAGGAGGTGTATGGCATCCAAATCTACACGCTGGATGAGCACCTGGAGATCGATGCTGCCATGTTGACCTCCACTGCAGACACCCCACTCTGTCTACAGTGTAAGCCCCTGAAGTATAAGGTGGTCCGGGACCCTCCTCTCCAGATGGATGTGGCCTCCATCCTACATCTCAATAGGAAATTCGTAGACATGGATCTAAATCTTGGCAGTGTGTACCAGTATTGGGTCATAACTATTTCAGGAACTGAAGAGAGTGAGCCATCACCTGCTGTCACATACATCCATGGAAGTGGGTACTGTGGCGATGGCATTATACAAAAAGACCAAGGTGAACAATGCGACGACATGAATAAGATCAATGGTGATGGCTGCTCCCTTTTCTGCCGACAAGAAGTCTCCTTCAATTGTATTGATGAACCCAGCCGGTGCTATTTCCATGATGGTGATGGGGTATGTGAGGAGTTTGAACAAAAAACCAGCATTAAGGACTGTGGTGTCTACACGCCCCAGGGATTCCTGGATCAGTGGGCATCCAATGCTTCAGTATCTCATCAAGACCAGCAATGCCCAGGCTGGGTCATCATCGGACAGCCAGCAGCATCCCAG |
| circRNA13002 | ATTTTCAGCTGAACTCTCATCTCTCAACACTGGCAAATATTCATAAGATCTACCACACCCTTAATAAGCTGGAAGTCTGCGGTCTTGCAGTTCTTCAGACTGCTTTAATAAAGTGATGCCACCAAGGAAAAAGAGAAGACCTGCCTCTGGAGATGATTTATCTGCCAAGAAAAGTAGACATGATAGCATGTATAGAAAATATGATTCGACTAGAATAAAGACTGAAGAAGAAGCCTTTTCAAGTAAAAGGTGCTTGGAATGGTTCTATGAATATGCAGGAACTGATGATGTTGTAGGCCCTGAAGGCATGGAGAAATTTTGTGAAGACATTGGTGTTGAACCAGAAAACGTGAGTCAAACTTACTGAGTTGGGTGAATCAGTTGGTTGTTTTTCATACTTAAATCTTTGTTCTTTAGCAAATAAATAAATAATTAAAAAGTAGTGGTATGTTAGTTTTTATGAAGCAGTCTAAGAAATAAGTTCTAATTCTAGTTTGACTTATAAGCAGATTCTCCATTCTTGTAAGTGATATGGTGTAACTACAGTTATTTTTTCTCTCATTTAATTTCTTGTATGTAAAAGGTACAGTAAGCCAGATGCTTACAAAATGGTGTGGCCACATGTGCCTACAATGACGGATCAACTGGAGGCCACATTGTACGCTGTGTACCTTCGTGCCCCTCAGTAGTTGTTTTAGCCTAATGTAGAGTCAATCTAGGACTTATAATTATTCATCATGATTTTGAGTAGATTGTAATCATCAAGAATTTTTCATAGATCGTTTACTTCCAATTGAATTTAGCTCAGAAGTGATTGCTTTTTTTTTTTTGAGATGGAGTCTCGCACTGTCGCCAGGCTGGAGTGCAATGGTATGATGTCGGCTCACTGCAACCTCTGCCTCCCGGGTTGAAGCGATTTCCCCTGCCTCAGCCTCCTGAGTAGCTGGGACTACAGGTAGTTATGCTTGTCCTAGCTTGGAAATTGGATGCACAAAACATGGGTTATTTTACTCTACAGGAGTGGTTAAAAGGAATGACTTCTCTCCAATGTGATACAACAGAAAAACTCAGAAATACTTTGGATTACTTAAGATCATTCTTAAATGATTCTACAAACTTTAAACTTATTTACAGATATGCGTTTGACTTTGCACGG |
| circRNA13291 | AGCATCTGCTGCACCCAAGCCTGAGCCGGTTCCTGTTCAAAAGAAAACACAAGTGACAAATAACCCTGGCACTGTGAAAATCCCACCTAAACGCCCACCAAGAAAACACATTGTGGAGCGCTATACAGAGTTTTATCATGTACCCACTCACAGTGATGCCAGCAAGAAGAGACTGATTGAGGATACTGAAGACTGGCGTCCAAGGACTGGAACAACTCAGTCTCGCTCTTTCCGAATCCTTGCCCAGATCACTGGGACTGAACATTTGAAAGAATCTGAAGCCGATAATACAAAGAAGGCAAA |
| circRNA15457 | TCCTGGAAGATACTGCTCTTTGGTGTAATAAACTTGATATGTACTGGCTTCCTGCTTATGTGGTGCAGTTCTACTAATAGTATAGCTTTAACTGCCTATACTTACCTGACCATTTTTGATCTTTTTAGTTTAATGACATGTTTAATAAGTTACTGGGTAACATTGAGGAAACCTAGCCCTGTCTATTCATTTGG |
| circRNA16161 | CTGTTTGGGGCAACTTCGTTAATATGAGCTTTCTACTCAACAGGTCTATCCAGGAAAATGGTGAACTAAAAATTGAAAGCAAGATTGAAGAGATGGTTGAACCACTAAGAGAGAAAATCAGAGATTTAGAAAAAAGCTTTACCCAGAAATACCCACCAGTAAAGTTTTTATCAGAAAAGGATCGGAAAAGAATTTTG |
| circRNA1728 | GATATTGGATTCCGACTCGACTCATTACATACCATCCTGCAACAGGAAGTCCTGTTACAAGAGGATGTGGAGCTGATTGAGCTACTTGATCCCAGTATCCTGTCTGCAGGGCAATCTCAACAACAGGAAAATGGACACCTTCCAACACTTTGCTCCCTGGCAACCCCTAATATTTGGGATCTCTCAATGCTATTTGCCTTCATTAGCTTGCTCGTTATGCTTCCCACTTGGTGGATTGTGTCTTCCTGGCTGGTATGGGGAGTGATTCTATTTGTGTATCTGGTCATAAGAGCTTTGAGATTATGGAGGACAGCCAAACTACAAGTGACCCTAAAAAAATACAGCGTTCATTTGGAAGATATGGCCACAAACAGCCGAGCTTTTACTAACCTCGTGAGAAAAGCTTTACGTCTCATTCAAGAAACCGAAGTGATTTCCAGAGGATTTACACTGGTCAGTGCTGCTTGCCCATTTAATAAAGCTGGACAGCATCCAAGTCAGCATCTCATCGGTCTTCGGAAAGCTGTCTACCGAACTCTAAGAGCCAACTTCCAAGCAGCAAGGCTAGCTACCCTATATATGCTGAAAAACTACCCCCTGAACTCTGAGAGTGACAATGTAACCAACTACATCTGTGTGGTGCCTTTTAAAGAGCTGGGCCTTGGACTTAGTGAAGAGCAGATTTCAGAAGAGGAAGCACATAACTTTACAGATGGCTTCAGCCTGCCTGCATTGAAGGTTTTGTTCCAACTCTGGGTGGCACAGAGTTCAGAGTTCTTCAGACGGTTAGCCCTATTACTTTCTACAGCCAATTCACCTCCTGGGCCCTTACTTACTCCAGCACTTCTGCCTCATCGTATCTTATCTGATGTGACTCAAGGTCTACCTCATGCTCATTCTGCCTGTTTGGAAGAGCTTAAGCGCAGCTATGAGTTCTATCGGTACTTTGAAACTCAGCACCAGTCAGTACCGCAGTGTTTATCCAAAACTCAACAGAAGTCAAGAGAACTGAATAATGTTCACACAGCAGTGCGTAGCTTGCAGCTCCATCTGAAAGCATTACTGAATGA |
| circRNA24045 | GTCTCAGCTTGACTATGAAGTTCCCAGGGAAAAGGCCTTCCAAAAGAGCAGCACCGGCTTTTCACCTGAGACTTCCTTCCTGGATAGCCAGGTGATGACTGCTCTGAAGATGGAGAGATACCTGAAGAAGATCCACTTCCTCTATCTCAATGTGGCTCCCAGCCGGTACTTTAGGCCTTACAGCCTGATGGTGGTGCCACCCGACAAGGTGAATCCCGAGCACTACATCTTCTCTCCCTTTGGGATCTTGCATGTACATCCTGTGGAAGGTAGCGAGACGATGACACTGGGTACCTGGCACCATCACTGTGTTCTCTGGCAGCAGCTCCAGTTCATTCCATTCTTTAAGTATTGCCTCTTACGCAAGTCCTTTACCTGTTGGAAGAAGAATGTGAGATTACAGGGGCTGCATCGACTCCAGAAATTCCTAGAGAATCATCTGCTCTTGGCTGTGCCCCACTTTGGAGCTGGGCTACTCCATATTAGCAGGCTTCTGCAGGAGCTACACTCTGTGTCCTGGCTACCCCAGGAACTGGATCGGTGCTATGAGCTGCTGGACCTGCAGACGGCTCTAGCCGAGGAGAAGCATAAGGCTCTACGGCTGCTCCATCGTTGCCTAAACCTCTGCACATCCATTCTTCGACTG |
| circRNA27199 | GTGGGAACATCCAGTTGCAGGAAAACAAGCTTAACACGCCCACTGATTCTACATTATGGGTCTCCCTCTGTTGTCCAAGGCTGGAGTGTAGTAGTGCTATCGCAGCTGACTGCAGCCTCAACCTTCCAGGCTGAAGCGATCCTCCCACCTCAACCTCCCACGTGGCTGAGACTACAGGTGCTTGCCACTATGCCCAACTAACATTTGGAATTTTCGTATACGTGGATTCTAGAGGGGTGACAGCGAAAC |
| circRNA28259 | GAGGCTTGGAGTACCCATAATACAGTGAGCCCACCCTCGTGGTCCCCAGACATTTCAGGAGGTCGGGAAATTTTTAAACCCAGGCAGCTTCCTGGCAGTGCCATTTGGAGCATCAAAGTGGGCCATGGGTCTGGATTTCCAAGAAAGCGGAGACCTCGAGGTGCAGGACTGTCGGGGCGAGGTGGCCGAGGCAGGTCAAAGCTGAAAAGTGGAATCGGAGCTGTTGTATTGCCTGGGGTGTCTACTGCAGATATTTCATCAAATAAGGATGATGAAGAAAACTCTATGCACACTACGGTTGTGTTGTTTTCTAGCAGTGACAAGTTCACTTTGAATCAGGATATGTGTGTAGTTTGTGGCAGTTTTGGCCAAGGAGCAGAAGGAAGACTACTTGCCTGTTCTCAGTGTGGTCAGTGTTACCATCCATACTGTGTCAGTATTAAGATCACTAAAGTGGTTCTTAGCAAAGGTTGGAGGTGTCTTGAGTGCACTGTTTGTGAGGCCTGTGGGAAGGCAACTGACCCAGGAAGACTCCTGCTGTGTGATGATTGTGACATAAGTTATCACACCTACTGCCTAGACCCTCCATTGCAGACAGTTCCCAAAGGAGGCTGGAAGTGAAGTGCAAATGGTGTGTTTGGTGCAGACACTGTGGAGCAACATCTGCAGGTCTAAGATGTGAATGGCAGAACAATTACACACAGTGCGCTCCTTGTGCAAGCTTATCTTCCTGTCCAGTCTGCTATTGAAACTATAGAGAAGAAGATCTTATTCTGCAATGTAGACAATGTGATAGATGGATGCATGCAGTTCGTCAGAACTTAAATACTGAGGAAGAAGTGGAAAATGTAGCAGACATTGGTTTTGATTGTAACATGTGCAGACCCTATATGCCTGCATCTAATGTGCCTTCCTCAGACTGCTGTGGATCTTCACTTGTAGCACAAATTGTCACAAAAGTAAAAAAGCTAGACCCACCCAAGACTTATACCCAGGATGATGTGTGTTTGATTGAATCAGGGATGACTCAGTTACAGAGCCTCACAGTTACAGTTCCAAGAAGAAAACTGTCAAAACCAAAACTGAAATTGAAGATTATAAATCAGAATAGCGTGGCCGTCCTTCAGACCCCTCCAGACATCCAATCAGAACATTCAAGGGATGGTGATATGGATGATAGTCGAG |
| circRNA30114 | GGCTGGTGAATTACCAGATCTCCGTCAAGTGCAGTAACCAGTTCAAGTTGGAAGTGTGTCTTTTGAATGCAGAAAACAAGGTCGTGGACAACCAGGCTGGGACCCAGGGCCAGCTGAAGGTGCTGGGTGCCAACCTCTGGTGGCCGTACCTGATGCACGAACACCCCGCCTACCTGTACTCGTGGGAGGTGTGGCTGACTGCACAGAAGTCACTGGGGCCTTTGACTTCTACACACTCCCTGTGGGGCTCCGCACTGTGGCCGTCACCGAGAGCCAGTTCCTCATCAGCGGGAAACCTTTCTATTTCCACGGCGTCAACAAGCATGAGGATGCGGACATCCAAGGGAAGGGCTTCGACTGTCCGCTGCTGGTGAAGGACTTCAACCTGCTTTGCTGGCTTGGCGCCAACACCTTCTGCCCCAGCCACTATCCCTACACGGAGGAGATGCTGCAGATATGTTACCGGTATGGGATTGTGGTCATCGATGAGTGTCCTGCTGTGGGCCTGATGCTGCCGCAACTCTTCAACAACGTGTCTATGCATCACCATATGTGGGTGGTGGAGGAACCGGTGCTAAGAGACAAGAACCACCCGCCATCATGATGTGGTCTGTGGCCAACGAGCCTGCGTCCTTCCTGGAATCTGCTGGCTACTCCTTCAAGATGGTGATTGCTCACACCAAAGCCTTGGACCCCTCCCAGCCTGTGACCTTTGTGACCAACTCCACCTACGCAGCAGACAAGGGG |
| circRNA32134 | GCTGACCAACTGACTGAAGAGCAGATTGCAG |
| circRNA33070 | GTGGGAACATCCAGTTGCGGGAAAACAAGCTTAACACGCCCACTGATTCTACATTATGGGTCTCCCTCTGTTGTCCAAGGCTGGAGTGTAGTAGTGCTATCGCAGCTGACTGCAGCCTCAACCTTCCAGGCTGAAGCGATCCTCCCACCTCAACCTCCCACGTGGCTGAGACTACAGGTGCTTGCCACTATGCCCAACTAACATTTGGAATTTTCGTATACGTGGATTCCAGAGGGGTGACAGCGAAAC |
| circRNA36024 | GTATCCATCACGAATTGAAAAAATTGACTATGAGGAGGGCAAGATGTTGGTCCATTTTGAGCGCTGGAGTCATCGTTATGATGAGTGGATTTACTGGGATAGCAATAGATTGCGACCCCTTGAGAGACCAGCACTAAGAAAAGAAGGGCTAAAAGATGAGGAAGATTTCTTTGATTTTAAAGCTGGAGAAGAAGTTCTGGCTCGTTGGACAGACTGTCGCTATTACCCTGCCAAGATTGAAGCAATTAACAAAGAAGGAACATTTACAGTTCAGTTTTATGATGGAGTAATTCGTTGTTTAAAAAGAATGCACATTAAAGCCATGCCCGAGGATGCTAAGGGGCAGGATTGGATAGCTTTAGTCAAAGCAGCTGCTGCAGCTGCAGCCAAGAACAAAACAGGGAGTAAACCTCGAACCAGCGCTAACAGCAATAAAGATAAGGATAAAGATGAGAGAAAGTGGTTTAAAGTACCTTCAAAGAAGGAGGAAACTTCAACTTGTATAGCCACACCAGACGTAGAGAAGAAGGAAGATCTGCCTACATCTAGTGAAACATTTGGACTTCATGTAGAGAACGTTCCAAAGATGGTCTTTCCACAGCCAGAGAGCACATTATCAAACAAGAGGAAAAATAATCAAGGCAACTCGTTTCAGGCAAAGAGAGCTCGACTTAACAAGATTACTGGTTTGTTGGCATCCAAAGCTGTTGGGGTTGATGGTGCTGAAAAAAAGGAAGACTACAATGAAACAGCTCCAATGCTGGAGCAG |
| circRNA40049 | ATTACAGGCATGAGCCACTGTGCATGGCCTAAGCCTCTGCATTTTATCCTCAGACCTTTCTCATGTTCTATTTTCTTCTACTTCATACCTTTCCCCAAGTCCAGGTAAAGTTTGCCTGGCCCAGAGTAAATCAGCACCCAAAGATGACACCAATGTGGACAGACTTAGGGGAAATCTGGTTAGAAGAAGGCCACTAAAGGCTATCAGTACTTAAGGCTGTACTTTGCTCAATTCTCTGTCCCCAAGACTTAAAACACAGCACACAATGTGGTAGAAATTTGGATAGTTAAATCAGTTATCATCAGTAATAACCTAGAAGAAAAGCAGACACAAGAACTCAAAAAGCACAGCATCTAAGCTGAAAACCTTATTTATCAACTTGTCACCAACCACCATTTGAATTTCAAATTTTTTTATTGCCGGCACGATGGCTCACACCTGTAATCCCAGCACTTTGGGAGGTGGAGGTGGGCGGATTACTTGAAGTCAGGTGTTCCAGACCAGCCTGGCCAACATGGTGAAACCCCATCTCTACTAAAAATAAAAAAAATTGGCCGGCGTGGTGGCACATGCCTGTAATCCCAGCACTTTGGGAGGCCGAAGCAGGTGGATCATGAGGTCAGGAGATCAAGACCATCCTGGCTAACATGGTGAAACCCCGTCTCTACTAAAAATACAAGAAATTAGCCAGGGATAGTGGCGGGTGCCTATAGTCCCAGCTACTTAGGAGGCTGAGGCAGGAGAATGGTGTGAACCTGGGAGGCAGAGCTTGCAGTGAGCCGAGATCGCGCCACTGCACTCCAGCCTGGGCGACAGAACGAGACTCCATCTCAAAAAAAAAAAAAAAAATACAAAAAATACAAAAAATTAGCCAAGCTAATTAGCCAAGTGGCACACGCCTGTAATCCCAACTACTTGGGAGGCTGAGGAATGAGAATCGCTTGAACCCGAGAGATGGAGGTTGTAGTGAGCCAAGATCGTGCCACTGCCCTCCAGCTTGGGCAAAGAGTGAGACTCTGTCTCTGGGGAAAAAAAATATTAATTTCTCCCTGGATAAAATTGTATCTGCTGGGGTGGAATGGAAGTAGGTGAGGTTGGGGGACCTATGAAGAAAAAAGAGCCTTTCCACTGGGCAGTGAAGTGTGTACACACACACTGGGGGCAGAGCAGTGTGAAAACATTCTGCACCAGCACACAAATGACTTTTGGCAAATCATCTTCCTGATCTGTCGGATGTTACGTCTCTGCAGAATCTGGAGAAAACCAGAAAACCCAGCTTGTTTGCCCTCATTTTGGCAGTTTAATTTAGGAATCACACTGGCTTTACATAAACTCTTTACCAAAAAAACTGTATTCTGTATTTTGAAGGCACAAGTTAACATGGGCCCAAGGGAAGGAAGCATTGTATACAATTACATAATAGCTACTCTATTACTTTAAAACCTAATGGCAGCCTCGGGCAGAAAAGTCAAAAGGGGAGAGAAACCATTTCTGTGAAATTATCTGATGCAATCATCTCTTTGGAGACATTGTCAGTTGACAATGGTTCTGCTTTTTCTCTCGAGCTCATTCAATTTCTGTTATTTACCTGAACCTGGAAGCAGAGTTTTCCTCCCATCTACCACTGGTTTAAAAAAAAAAAAAAAAAAGAGGCCGGGAGCAGTGGCTCACGCCTGTAATCCCAACACTTTGGGAGGCTGAGGCGGGTGGATCACGAGGTCAGGAGTTCAAGACCAGCCTGGCCAAGATGGTGAAACCCCCGTCTCTACTAAAGATACAAAAATTAGCCGGCTGTGATGGCGGGCGCCTGTAATCCCAGCTACTCGGGAGGCTGAGGCAGAGAATTGCTTGAATCTGGGAGGCAGAGATTGCAGTGAGCCGAGATCATGCCACTGCACTCCAGCCTGGGCAACAGAGACTCTGTCTCAAAAAAAAAAAAAAAAAAAAAAAAACAAAGGCAGGCAGGATTTCTCTAATAAAAGCTTGAAATGAAAAGCAGTAACTACTCCCAAGGCCCTGCTTATTGTAGAAAAGTCATTCAACAAATCTTTTTAGGTCTCTATTAACAATTTATTTTCTCTACCCCCATCACAAAAAAGCAGAGGAAGTAGTGTAGCAGCCCCAGCCACAGCCACAGCCACACCCCACTACTTGCTTCCTAGGTGTGTGTGGTGTGTGACTGCCTGCAAGCAACTCCAGCTCTCTGAACTTGTTTCCTCATCTGCAAACTGGAAATATTTATTTATTTGACAAGGACATTATGAGAATTTACTCTGAAAAATACATGTAGAGTACCTAGCATTCAATTAAGACTAGGTGAATGCCCTTCTCAGGAACCGTACTAGAGAAGGTGAGTTTCTGGTCCAGTGTGATGACTCACGCCTGTAATCCCAGCACTTTAGGAGGCTGAG |
| circRNA41102 | GGTCTCTCCTCAGGGATGACATCATCCGTCCACCTCCTTGTCTTCAAGGACCACCTCCTCTCCATGCTGAGCTGCTGCCAAGGGGCCTGCTGCCCATCTACACCTCACGAGGGCACTAGGAGCACGGTTTCCTGGATCCCACCAACATACAAAGCAGCCACTCACTGACCCCCAGGACCAGGATGGCAAAGGATGAAGAGGACCGGAACTGACCAGCCAGCTGTCCCTCTTACCTAAAGACTTAAACCAATGCCCTAGTGAGGGGGCATTGGGCATTAAGCCCTGACCTTTGCTATGCTCATACTTTGACTCTATGAGTACTTTCCTATAAGTCTTTGCTTGTGTTCACCTGCTAGCAAACTGGAGTGTTTCCCTCCCCAAGGGGGTGTCAGTCTTTGTCGACTGACTCTGTCATCACCCTTATGATGTCCTGAATGGAAGGATCCCTTTGGGAAATTCTCAGGAGGGGGACCTGGGCCAAGGGCTTGGCCAGCATCCTGCTGGCAACTCCAAGGCCCTGGGTGGGCTTCTGGAATGAGCATGCTACTGAATCACCAAAGGCACGCCCGACCTCTCTGAAGATCTTCCTATCCTTTTCTGGGGGAATGGGGTCGATGAGAGCAACCTCCTAGGGTTGTTGTGAGAATTAAATGAGATAAAAGAGGCCTCAGGCAGGATCTGGCATAGAGGAGGTGATCAGCAAATGTTTGTTGAAAAGGTTTGACAGGTCAGTCCCTTCCCACCCCTCTTGCTTGTCTTACTTGTCTTATTTATTCTCCAACAGCACTCCAGGCAGCCCTTGTCCACGGGCTCTCCTTGCATCAGGGCTAATCTCGGGCCTTGTCGAAGGAAGAGGCTGCAGACGTTAATGAGGTTAGCTGCTGGATTCCAGTATTCGTCGCATAAGGATCCTTCTTTGTCTGCGAAGGAAAAACACACTGATTATCATAATGAGGCACGTGGGCCGTGGCCCGGCTGGGTCGGCTGAAGAACTGCGGATGGAAGCTGCGGAAGAGGCCCTGATGGGGCCCACCATCCCGGACCCAAGTCTTCTTCCTGGCGGGCCTCTCGTCTCCTTCCTGGTTTGGGCGGAAGCCATCACCTGGATGCCTACGTGGGAAGGGACCTCGAATGTGGGACCCCAGCCCCTCTCCAGCTCGAAATC |
| circRNA422 | GTTTCCGATGGCACCTGTGTCAAGGTCTTCCAACAACTCCGGGTCTTCCAGCGACTTCAAGTCTTCCAATAATCTCAAGGTCTTCCAGATAATCCTGAGCTTCCAGAAAATCCACATCTTCCAGACAATCCATGTCTTCCGGACAATCCATGTCTTCCAAGAAGCTCCAAGTCTTCCAGTAAATCAAGTCTTCCAGCAAATCCAGTCTTCCAGCAATTACTGGTCTTCCACCAAATCCAGATCTTCCAGGAAAATCCACGTCTTCCAGGAAATCCATGTCTTCCAATAATTTCAAGGTCTTCCATCAAATACAGATCTTCCAGCTAATCCATGTCTTCCAGAAAAATCTGTGTCTTCCACCAAATCCAAGTCTTCCAGTAAATCTAGTTCTTCCAGAAAAATCTAGATCTTCCAGTCAATCAGTGTCTTCCAGAAAGAAATCCAGGTCTTCCAGTCAATCAGTGTCTTCCAGAAAGAAATCCAGGTCTTCCAGTCAGTCAGTGTCTTCCAGAAAAATCTACGTCTTCCACCAAATCCAGGTCTTCCAGTCAATCCACATCTTCCGGAAAAAATCCAGGTCTTCCAGCCAATATATGTCTTCCTGAAGATCCACGTCTTCCAGAAAATCCATGTCTTCCAGAAAATCCATGTCTTCCAGTAACCTCCCAGTCTTCCAGAAAATCCACGTCTTCCCAACAATCCAAGTCTTCCGGATAATTTGGGTCTTCCTGAAAATCTACGTCTTCCAAAAAAGCCATGTCTTCCAGAAAATCCACATCTTCCAATGGCCTCCAGGTCTTCCAGACTATCCATGTCTTCCAGAAAATCCTTGTCTTCCCTTAAATCTATAGCTTCCAAAAAATCCGGGTCTTCCAGGAAATCCGTGTCTTCCAGCAAGTCCACGTCTTCCAACAAAGCCATGTCTTCCAGACTATCCATGTCTTCCAGAAAATCCTTGTCTTCCCTCAAATCCATAGCTTCCGAAAAATCCAGGTCTTCCAGGAAATCCGTGTCTTCCAGCAAATCCACGTCTTCCAACAAAGCCATGTCTTCCATCAAATTAATGTCTTCCAGCCTACTTGTGTCTTCCAACAAAGGTACGTCTTCCAACAAAGGTACGTCTTCCAACAAAGGTATGTCTTCCAACAAAGGTACGTCTTCCAGAAAATCCACGTCTTCCAACCAAGCCATGTCTTCCAGAAAATCCACGTCTTCCAGAAAATATATGTCTTCCAACTAAGCTACGTCTTCCAACAAATCCATGTCTTCCTATATCTCCAGGTCTTCCAGCATCTCCAGGGCTTCCAGCATCTGCTCGTCTTCCAACATCTCCACGTCTTCCAGCATCTCTGTGTCTTCCAGCATCTTCATGTCTTCCAACAACTACCCAGTCTTCCATCAACTGGCTCAATATCCATGTCTTCCAACGTCTCCAGTGTGCTGATCTTCTGACATTCAGGTCTTCCAGTGTCTGCAATATCCAGG |
| circRNA42950 | GTGTGTCGAACCAAGGTGATAGATCTCAGTGAAGGCATTTCCCAGCATGCCTGGTACCCTTGCACCATCAGCTACCCATATTCCCAGCTGGCTCAGACCACTTTTTGGCTCCGGGCGTATTTTTCTCAACCAATGGTTGCCGCAGCTGTCATTGTCCACCTGGTGACGGATGGGACATATTATGGGGACCAAAAGCAGGAGACCATCAGCGTGCAGCTGCTTGATACCAAAGATCAGAGCCACGATCTAGGCCTCCATGTCCTGAGCTGCAGGAACAATCCCCTGATTATCCCTGTGGTCCATGACCTCAGCCAGCCCTTCTACCACAGCCAGGCGGTACGTGTGAGCTTCAGTTCGCCCCTGGTCGCCATCTCGGGGGTGGCCCTCCGTTCCTTCGACAACTTTGACCCCGTCACCCTGAGCAGCTGCCAGAGAGGGGAGACCTACAGCCCTGCCGAGCAGAGCTGCGTGCACTTCGCATGTGAGAAAACTGACTGTCCAGAGCTGGCTGTGGAGAATGCTTCTCTCAATTGCTCCAGCAGCGACCGCTACCACGGTGCCCAGTGTACTGTGAGCTGCCGGACAGGCTACGTGCTCCAGATACGGCGGGATGATGAGCTGATCAAGAGCCAGACGGGACCCAGCGTCACAGTGACCTGTACAGAGGGCAAGTGGAATAAGCAGGTGGCCTGTGAGCCAGTCGACTGCAGCATCCCAGATCACCATCAAGTCTATGCTGCCTCCTTCTCCTGCCCTGAGGGCACCACCTTTGGCAGTCAATGTTCCTTCCAGTGCCGTCACCCTGCACAATTGAAAGGCAACAACAGCCTCCTGACCTGCATGGAGGATGGGCTGTGGTCCTTCCCAGAGGCCCTGTGTGAGCTCATGTGCCTCGCTCCACCCCCTGTGCCCAATGCAGACCTCCAGACCGCCCGGTGCCGAGAGAATAAGCACAAGGTGGGCTCCTTCTGCAAATACAAATGCAAGCCTGGATACCATGTGCCTGGATCCTCTCGGAAGTCAAAGAAACGGGCCTTCAAGACTCAGTGTACCCAGGATGGCAGCTGGCAGGAGGGAGCTTGTGTTCCTGTGACCTGTGACCCACCTCCACCAAAATTCCATGGGCTCTACCAGTGTACTAATGGCTTCCAGTTCAACAGTGAGTGTAGGATCAAGTGTGAAGACAGTGATGCCTCCCAG |
| circRNA44372 | GGCTGGTGAATTACCAGATCTCCGTCAAGTGCAGTAACCAGTTCAAGTTGGAAGTGTGTCTTTTGAATGCAGAAAACAAAGTCGTGGACAACCAGGCTGGGACCCAGGGCCAGCTGAAGGTGCTGGGTGCCAACCTCTGGTGGCCGTACCTGATGCACGAACACCCCGCCTCCCTGTACTCGTGGGAGGATGGTGATTGCTCACACCAAAGCCTTGGACCCCTCCCAGCCTGTGACCTTTGTGACCAACTCCACCTACGCAGCAGACAAGGGG |
| circRNA45487 | GGTCCAGATAATGAAGAGCGTTTAGCAGCAACAGCAGCAGGGGAGGCCGAGGCACTCGCCAGCATGCCTGGGGAAGTGGAGGCCAGTGGTGTGGCCCCCGGGGAGCTGGACCTCTCCATGTCCGCCCAGAGCCTCGGGGAAGAGGCCACTGTGGGTCCAAGCAGTGAAGACAGTTTAACAACAGCTGCAGCTGCAACCGAAGTGTCCCTCAGTACTTTTGAGGATGAGGAAGCCAGTGGGGTCCCCACAGATGGCCTGGCTCCCCTCACAGCCACCATGGCCCCTGAGCGGGCAGTCACTTCTGGTCCTGGTGATGAAGAAGACTTGGCAGCAGCCACAACAGAGGAGCCCCTCATCACAGCTGGGGGTGAAGAGTCCGGCAGCCCTCCCCCTGATGGGCCACCGCTGCCCCTGCCCACAGTGGCTCCTGAAAGATGGATCACTCCA |
| circRNA5030 | AGCCACCCCTGAGTGACGACCTGCAGCCTTCCTCAGCTGTGTCCAGCCCCACCCAGCCAGGTCCTGTCCTGTACATGCCATCTGCCGCCGGAGACTCGGTGCCTGTGAGCCCCTCCAGCCCGCATGCCCCTGACCTCAGTGCCCTCCTCTGTAGAAACAGCAGCCTAGGCAGCCCGTCTAACCTCTGCGGCTCCCCACCGGGCTCCATCAGGAAGCCCCCAAACCTGGAGGGCATCGTCTTCCCTGGGGAGTCTGGCCTGGCCCCTGGCAGCTATAAGAAGGCTCCCGGCTTCGAGAGGGAAGACCAGGTGGGAGCCGAGTACCTGAAAAATTTCAAATGCCAGGCCAAATTAAAACCCCACTCATTAGAGCCCAGGAGTCAAGAGCAGCCTCTGCTTCAGCCCAAACAGGACATGCTGGGCATCCTCCCCGCAGGCAGCCCCCTGACCTCAAGCATCTCTTCTAGTATCACCTCCAGCCTGGCAGCTACCCCCCCTAGCCCAGTGGGCACCAGCAGCGTCCCCGGCATGAATGCAAACGCTCTGCCCTTCTACCCCACCAGCGACACGGTAGAGTCAGTCATAGAGTCTGCTTTGGATGACCTGGACCTGAATGAGTTTGGCGTGGCCGCCCTGGAGAAGACTTTCGATAACAGCACAGTGCCCCACCCAGGAAGCATCACCATCGGCGGCAGCTTGCTGCAGAGCTCTGCACCCGTGAACATCCCCGGCTCCTTGGGCAGCTCTGCCTCCTTCCACTCAGCATCCCCGTCCCCTCCCGTCAGCCTCTCCTCGCATTTCCTGCAGCAGCCCCAGGGCCACCTGAGTCAGTCGGAAAACACATTTTTGGGAACCTCAGCATCACATGGATCTTTGG |
| circRNA52499 | CAGGAGGAGGCCTCAGGGGGGCCCACAGCCCCCAAAGCTGAGAGTGGTCGAAGCGGAGGTGGGGGACTCATGGAAGAGATGAACGCCATGCTGGCCCGGAGAAGGAAAGCCACGCAAGTTGGGGAGAAAACCCCCAAGGATGAATCTGCCAAT |
| circRNA55294 | GGGCCCCAAGGACCAAAAGGGCAGAAAGGTGAGCCTTATGCACTGCCTAAAGAGGAGCGCGACAGATATCGGGGTGAACCTGGAGAGCCTGGATTGGTCGGTTTCCAGGGACCTCCCGGCCGCCCTGGGCATGTGGGACAGATGGGTCCAGTTGGAGCTCCAGGGAGACCAGGACCACCTGGACCCCCTGGACCAAAAGGACAGCAAGGCAACAGAGGACTTGGTTTCTACGGAGTTAAGGGTGAAAAGGGTGACGTAGGGCAGCCGGGACCCAACGGGATTCCATCAGACACCCTCCACCCCATCATCGCGCCCACAGGAGTCACCTTCCACCCAGATCAGTACAAGGGTGAAAAAGGCAGTGAGGGGGAACCAGGAATAAGAGGCATTTCCTTGAAGGGAGAAGAAGGAATCATGGGCTTTCCTGGACTGAGGGGTTACCCTGGCTTGAGTGGTGAAAAAGGATCACCAGGACAGAAGGGAAGCCGAGGCCTGGATGGCTATCAAGGGCCTGATGGACCCCGGGGACCCAAGGGAGAAGCCGGAGACCCAGGGCCCCCTGGACTACCTGCCTACTCCCCTCACCCTTCCCTAGCAAAAGGTGCCAGAGGTGACCCGGGATTCCCAGGGGCCCAAGGGGAGCCAGGAAGCCAGGGTGAGCCAGGAGACCCGGGCCTCCCAGGTCCCCCTGGCCTCTCCATCGGAGATGGAGATCAGAGGAGAGGCCTGCCGGGTGAGATGGGACCCAAGGGCTTCATCGGAGACCCCGGCATCCCTGCGCTCTACGGGGGCCCACCTGGACCTGATGGAAAGCGAGGGCCTCCAGGACCCCCCGGGCTCCCTGGACCACCTGGACCTGATGGCTTCCTGTTTGGGCTGAAAGGAGCAAAAGGAAGAGCAGGCTTCCCTGGGCTTCCCGGCTCCCCTGGAGCCCGCGGACCAAAGGGGTGGAAAGGTGACGCTGGGGAATGCAGATGTACAGAAGGCGACGAAGCTATCAAAGGTCTTCCGGGACTGCCAGGACCCAAGGGCTTCGCAGGCATCAACGGGGAGCCGGGGAGGAAAGGGGACAGAGGAGACCCCGGCCAACACGGCCTCCCTGGGTTCCCAGGGCTCAAGGGAGTGCCTGGCAACATTGGTGCTCCCGGACCCAAAGGAGCAAAAGGAGATTCCAGAACAATCACAACCAAAGGTGAGCGGGGACAGCCCGGCGTCCCAGGTGTGCCCGGGATGAAAGGTGACGATGGCAGCCCAGGCCGCGATGGGCTCGATGGATTCCCCGGCCTCCCAGGCCCTCCC |
| circRNA56391 | ATAAGATCATTGAGGTTGAGGAGGAACAAGAGGACCCATATCTGAATGACCGCTGCCGAGGAGGCGGGCCCTGCAAGCAGCAGTGCCGAGACACGGGTGACGAGGTGGTCTGCTCCTGCTTCGTGGGCTACCAGCTGCTGTCTGATGGTGTCTCCTGTGAAGATGTCAATGAATGCATCACGGGCAGCCACAGCTGCCGGCTTGGAGAATCCTGCATCAACACAGTGGGCTCTTTCCGCTGCCAGCGGGACAGCAGCTGCGGGACTGGCTATGAGCTCACAGAGGACAATAGCTGCAAAGATATTGACGAGTGTGAGAGTGGTATTCATAACTGCCTCCCCGATTTTATCTGTCAGAATACTCTGGGATCCTTCCGCTGCCGACCCAAGCTACAGTGCAAGAGTGGCTTTATACAAGATGCTCTAGGCAACTGTATTG |
| circRNA56890 | AGACAGAAGAGATGGGTGATGAAGAAGTTTTCTCCTGGTTGAAGTGTGCAAAGGGACAGTCCCACGAACCAGAGAATCTCATGCCCACACAAATTATTCCTGGCACAGCTCTTTACAATATTGGAGACATGGTACATGCTGCCCGGGGCAAGTGGGGAATTAAAGCAAACTGCCCTTGTATCAGTCGACAGAACAAATCTGTATTGAGACCTGCCGTCACCAATGGGATGTCACAGCTTCCTAGCATAAACCCTAGTGCCTCTTCTGGAAACGAAACTACCTTCTCTGGTGGAGGAGGACCGGCACCAGTAACAACTCCAGAGCCGGACCATGTTCCCAAAGCCGACAGCACTGACATCAGATCTGAAGAGCCTCTGAAAACAGACAGTTCGGCATCAAATAGCAATAGTGAACTGAAAGCCATCAGGCCTCCTTGCCCTGACACGGCCCCACCCTCCTCCGCCCTGCACTGGTTGGCAGATTTAGCAACTCAGAAGGCTAAAGAAGAAACAAAAGAAGCAGGGTCCCTGAGGTCGGTGCTCAATAAAGAGTCTCATTCACCCTTTGGGCTGGACTCGTTCAACTCCACTGCAAAGGTCTCTCCGCTGACTCCAAAGCTTTTTAACAGTCTGTTGCTGGGTCCCACTGCCTCCAACAACAAAACCGAAGGGTCTAGCCTTCGAGACCTCCTTCACTCCGGGCCGGGAAAACTTCCTCAAACCCCCTTGGACACAGGCATACCCTTTCCCCCGGTCTTCTCTACATCCTCAGCAGGAGTGAAGAGCAAGGCCAGCCTACCCAACTTTCTTGACCACATCATTGCCTCAGTGGTAGAAAATAAGAAAACCTCAGATGCTTCAAAGCGGGCCTGCAACTTGACTGATACCCAGAAGGAAGTGAAGGAGATGGTGATGGGGTTAAATGTGCTAGATCCCCATACTTCTCACTCCTGGCTTTGTGATGGGAGGCTTCTGTGTCTCCATGACCCCAGCAACAAAAACAATTGGAAGATCTTCCGGGAGTGTTGGAAGCAAGGTCAG |
| circRNA5758 | GTTAAAAATCTGTAAGAGCCTGATTTTAGAATTCACCAGCTCCTCAGAAGTTTGGCGAAATATGAGTTATTAAGCCTACGCTCAGATCAAGGTAGCAGCTAGACTGGTGTGACAACCTGTTTTTAATCAGTGACTCAAAGCTGTGATCACCCTGATGTCACCGAATGGCCACAGCTTGTAAAAGATCAGGAGAACCTCAGTCTGACGACATTGAAGCTAGCCGAAT |
| circRNA59699 | AATTCTCCCTCTGAAGCACAGAATTTAGATGAGAATACAACTGAGGGCTGGGAAAATCGGATAAGACTATGGACTGACCAGTATGAAGAAGCTTTCACTAATCAGTACAGTGCAGATGTACAGAACGCGCTTGAACAACACCTACATTCTAGCAAGGAATTTGTGGGCAAACCTACTATTTTAGACACTATTAATAAGACTGAATTGGCCTGTAATAACACAGTTATTGGTTCCCAAATGCAGTTACAGCTGGGAAGAGTCACTCGTGTTCAAAAGCACCGGAAGATCCTGAGGGCTGCAAGAGATTTGGCTTTGGACACTCTTATAATAGAGTATCGTGGGAAAGTCATGTTACGACAGCAATTTGAGGTCAATGGGCATTTCTTCAAAAAACCATACCCCTTTGTGCTCTTCTACTCAAAATTCAATGGTGTAGAGATGTGTGTGGATGCCCGTACTTTCGGTAATGATGCTCGGTTCATCAGAAGATCATGTACACCAAATGCAGAGGTGCGACACATGATTGCAGATGGGATGATTCACCTGTGCATCTATGCTGTGTCTGCCATCACCAAGGATGCTGAGGTCACCATAGCATTTGATTATGAGTATAGTAACTGTAATTATAAAGTGGACTGTGCCTGTCACAAGGGAAACCGGAATTGTCCTATACAAAAAAGGAATCCTAATGCTACAGAACTGCCACTCCTACCACCTCCTCCAAGCCTACCCACCATTGGAGCAGAGACTAGACGTAGAAAAGCACGACGGAAAGAGCTAGAGATGGAGCAGCAGAATGAGGCTTCAGAGGAGAATAATGACCAGCAATCACAAGAAGTTCCAGAAAAAGTAACTGTATCCAGTGATCATGAGGAAGTAGACAATCCAGAAGAAAAACCAGAAGAAGAGAAAGAAGAGGTTATAGATGACCAGGAGAACCTAGCTCATAGCAGGAGGACCAGGGAAGATAGAAAGGTAGAAGCCATCATGCATGCTTTTGAAAACTTAGAGAAAAGAAAGAAGCGGCGGGATCAGCCCTTGGAACAGAGCAACTCTGATGTAGAGATTACTACAACCACCTCAGAGACTCCTGTTGGTGAAGAGACAAAAACTGAAGCCCCTGAATCTGAAGTTAGCAACTCTGTTTCAAATGTTACCATCCCAAGCACCCCACAGAGTGTTGGTGTGAATACCCGGAGGTCTTCCCAAGCAGGGGATATTGCTGCAGAAAAACTAGTCCCCAAGCCACCTCCAGCAAAGCCTTCTAGGCCCCGGCCGAAGAGTCGAATTTCTCGGTACAGGACCAGTTCAGCCCAAAGACTAAAGCGTCAGAAGCAGGCCAATGCACAGCAGGCAGAATTGTCACAAGCTGCCTTGGAAGAGGGAGGAAGTAACAGTTTAGTAACTCCTACTGAAGCTGGAAGTCTAGACAGTTCAGGAGAAAACAGGCCATTAACAGGGTCTGACCCAACTGTGGTGTCAATTACTGGATCCCATGTCAACCGTGCTGCATCTAAATACCCCAAAACCAAAAAGTATCTAGTTACAGAATGGTTGAATGACAAAGCAGAGAAGCAAGAGTGCCCTGTTGAGTGCCCTTTACGTATCACAACGGATCCAACTGTACTGGCAACGACCCTAAACATGTTACCAGGTCTTATCCATTCCCCGTTAATTTGCACCACCCCCAAACACTACATTCGCTTTGGCTCACCCTTTATCCCTGAGAGACGTCGAAGGCCCCTTCTGCCTGATGGCACATTCAGCTCCTGTAAGAAG |
| circRNA59971 | GAGGAAATAGCCCTGTCCAGGAGTTCACTGTGCCTGGGAGCAAGTCTACAGCTACCATCAGCGGCCTTAAACCTGGAGTTGATTATACCATCACTGTGTATGCTGTCACTGGCCGTGGAGACAGCCCCGCAAGCAGCAAGCCAATTTCCATTAATTACCGAACAGAAATTGACAAACCATCCCAGATGCAAGTGACCGATGTTCAGGACAACAGCATTAGTGTCAAGTGGCTGCCTTCAAGTTCCCCTGTTACTGGTTACAGAGTAACCACCACTCCCAAAAATGGACCAGGACCAACAAAAACTAAAACTGCAGGTCCAGATCAAACAGAAATGACTATTGAAGGCTTGCAGCCCACAGTGGAGTATGTGGTTAGTGTCTATGCTCAGAATCCAAGCGGAGAGAGTCAGCCTCTGGTTCAGACTGCAGTAACCACTATTCCTGCACCAACTGACCTGAAGTTCACTCAGGTCACACCCACAAGCCTGAGCGCCCAGTGGACACCACCCAATGTTCAGCTCACTGGATATCGAGTGCGGGTGACCCCCAAGGAGAAGACCGGACCAATGAAAGAAATCAACCTTGCTCCTGACAGCTCATCCGTGGTTGTATCAGGACTTATGGTGGCCACCAAATATGAAGTGAGTGTCTATGCTCTTAAGGACACTTTGACAAGCAGACCAGCTCAGGGAGTTGTCACCACTCTGGAGAATGTCAGCCCACCAAGAAGGGCTCGTGTGACAGATGCTACTGAGACCACCATCACCATTAGCTGGAGAACCAAGACTGAGACGATCACTGGCTTCCAAGTTGATGCCGTTCCAGCCAATGGCCAGACTCCAATCCAGAGAACCATCAAGCCAGATGTCAGAAGCTACACCATCACAGGTTTACAACCAGGCACTGACTACAAGATCTACCTGTACACCTTGAATGACAATGCTCGGAGCTCCCCTGTGGTCATCGACGCCTCCACTGCCATTGATGCACCATCCAACCTGCGTTTCCTGGCCACCACACCCAATTCCTTGCTGGTATCATGGCAGCCGCCACGTGCCAGGATTACCGGCTACATCATCAAGTATGAGAAGCCTGGGTCTCCTCCCAGAGAAGTGGTCCCTCGGCCCCGCCCTGGTGTCACAGAGGCTACTATTACTGGCCTGGAACCGGGAACCGAATATACAATTTATGTCATTGCCCTGAAGAATAATCAGAAGAGCGAGCCCCTGATTGGAAGGAAAAAGACAG |
| circRNA64462 | GTTGCCAGAGGAGGCCCGCCACCGGCGTGTGTTCGAGATGGTGGAGGCACTGCAGGAGCACCCTCGAGACCCCAACCAGATCCTGATCGGCTACAGCCGAGGCCTCGTTGTCATCTGGGACCTACAGGGCAGCCGCGTGCTCTACCACTTCCTCAGCAGCCAGCAACTGGAGAACATCTGGTGGCAGCGGGACGGCCGCCTGCTCGTCAGCTGTCACTCTGACGGCAGCTACTGCCAGTGGCCCGTGTCCAGCGAAGCCCAGCAACCAGAGCCCCTCCGCAGCCTCGTGCCTTACGGTCCCTTTCCTTGCAAAGCGATTACCAGAATCCTCTGGCTGACCACTAGGCAGGGGTTGCCCTTCACCATCTTCCAGGGTGGCATGCCACGGGCCAGCTACGGGGACCGCCACTGCATCTCAGTGATCCACGATGGCCAGCAGACGGCCTTCGACTTCACCTCCCGTGTCATCGGCTTCACTGTCCTCACAGAGGCAGACCCTGCAGCCA |
| circRNA64586 | CCTTCCAGTGCCCTGCCCACAGCCACTACGAGCTCTGCGGTGACTCCTGTCCTGGGAGCTGCCCGAGCCTGTCGGCACCCGAGGGCTGTGAGTCGGCCTGCCGTGAAGGCTGTGTCTGCGATGCTGGCTTCGTGCTCAGTGGTGACACGTGTGTACCTGTGGGCCAGTGTGGCTGCCTCCACGATGACCGCTACTACCCACTGGGCCAGACCTTCTACCCTGGCCCTGGGTGTGATTCCCTTTGCCGCTGCCGGGAGGGCGGTGAGGTGTCCTGTGAGCCCTCCAGCTGCGGCCCGCATGAGACCTGCCGGCCATCCGGTGGCAGCTTGGGCTGCGTGGCCGTGGGCTCTACCACCTGCCAGGCGTCGGGAGATCCCCACTACACCACCTTCGATGGCCACCGCTTCGACTTCATGGGCACCTGCGTGTATGTGCTGGCTCAGACCTGCGGCACCCGGCCTGGCCTGCATCGGTTTGCCGTCCTGCAGGAGAACGTGGCCTGGGGTAATGGGCGAGTCAGTGTGACCAGGGTGATCACGGTCCAGGTGGCAAACTTCACCCTGCGGCTGGAGCAGAGACAGTGGAAGGTCACGGTGAACGGTGTGGACATGAAGCTGCCCGTGGTGCTGGCCAACGGCCAGATCCGTGCCTCCCAGCATGGTTCAGATGTTGTGATTGAGACCGACTTCGGCCTGCGTGTGGCCTACGACCTTGTGTACTATGTGCGGGTCACCGTCCCTGGAAACTACTACCAGCTGATGTGTGGCCTGTGTGGGAACTACAACGGCGACCCCAAGGATGACTTCCAGAAGCCCAATGGCTCGCAGGCAGGCAACGCCAATGAGTTCGGCAACTCCTGGGAGGAGGTGGTGCCCGACTCTCCCTGCCTGCCGCCGCCCACCTGCCCGCCGGGGAGCGCGGGCTGTATCCCCAGCGACAAGTGTCCTCCCGAGCTGGAGAAGAAGTATCAGAAGGAGGAGTTCTGTGGGCTCCTCTCCAGCCCCACAGGGCCACTGTCCTCCTGCCACAAGCTGGTGGATCCCCAGGGTCCCTTGAAAGATTGCATCTTTGATCTCTGCCTGGGTGGTGGGAACCTGAGCATTCTCTGCAGCAACATCCATGCCTACGTGAGTGCTTGCCAGGCGGCTGGAGGCCACGTGGAGCCCTGGAGGAATGAAACTTTCTGTCCCATGGAATGCCCTCAGAACAGTCACTACGAGCTCTGTGCGGACACCTGCTCCCTGGGCTGCTCGGCTCTCAGTGCCCCTCTGCAGTGCCCAGATGGGTGTGCTGAGGGCTGCCAGTGTGACTCCGGCTTCCTCTACAACGGCCAAGCCTGCGTGCCCATCCAGCAATGTGGCTGCTACCACAATGGTGTCTACTATGAGCCGGAGCAGACAGTCCTCATTGACAACTGTCGGCAGCAGTGCACGTGCCATGTGGGTAAAGTCGTGGTGTGCCAGGAACACAGCTGCAAGCCGGGGCAGGTGTGCCAGCCCTCCGGAGGCATCCTGAGCTGCGTCACCAAAGACCCGTGCCACGGCGTGACATGCCGGCCACAGGAGACATGCAAGGAGCAGGGTGGCCAGGGCGTGTGCCTGCCCAACTATGAGGCCACGTGCTGGCTGTGGGGCGACCCACACTACCACTCCTTCGATGGCCGGAAGTTTGACTTCCAGGGCACCTGTAACTATGTGCTGGCAACAACTGGCTGCCCGGGGGTCAGCACCCAGGGCCTGACACCCTTCACCGTCACCACCAAGAACCAGAACCGGGGCAACCCTGCTGTGTCCTACGTGAGAGTCGTCACCGTGGCTGCCCTCGGCACCAACATCTCCATCCACAAGGACGAGATCGGCAAAGTCCGGGTGAACGGTGTGCTCACAGCCTTGCCTGTCTCCGTGGCCGACGGGCGGATTTCAGTGGCCCAGGGTGCATCGAAGGCACTGCTGGTGGCTGACTTTGGACTGCAAGTCAGCTATGACTGGAACTGGCGGGTAGACGTGACGCTCCCCAGCAGCTATCATGGCGCAGTGTGCGGGCTCTGCGGTAACATGGACCGCAACCCCAACAATGACCAGGTCTTCCCTAATGGCACACTGGCTCCCTCCATACCCATCTGGGGCGGCAGCTGGCGAGCCCCAGGCTGGGACCCACTGTGTTGGGACGAATGTCGGGGGTCCTGCCCAACGTGCCCTGAGGACCGGTTGGAGCAGTACGAGGGCCCTGGCTTCTGCGGACCCCTTTCATCTGGCACAGGGGGCCCCTTCACCACCTGCCATGCTCATGTGCCACCTGAGAGCTTCTTCAAGGGCTGTGTTCTGGACGTCTGCATGGGTGGTGGGGACCGTGACATTCTTTGCAAGGCTCTGGCTTCCTACGTGGCCGCCTGCCAGGCCGCTGGGGTTGTCATCGAAGACTGGCGGGCACAGGTTGGCTGTGAGATCACCTGCCCAGAAAACAGCCACTATGAGGTCTGTGGCCCACCCTGCCCAGCCAGCTGTCCGTCCCCTGCACCCCTTACGACGCCAGCCGTATGTGAGGGCCCCTGTGTGGAGGGCTGCCAGTGCGACGCGGGTTTCGTGTTAAGTGCTGACCGCTGTGTTCCCCTCAACAACGGCTGCGGCTGCTGGGCCAATGGCACCTACCACGAGGCGGGCAGTGAGTTTTGGGCTGATGGCACCTGCTCCCAGTGGTGTCGCTGCGGGCCTGGGGGTGGCTCGCTGGTCTGCACACCTGCCAGCTGTGGGCTGGGTGAAGTGTGTGGCCTCCTGCCATCCGGCCAGCACGGCTGCCAGCCCGTCAGCACAGCTGAGTGCCAGGCGTGGGGTGACCCCCATTACGTCACTCTGGATGGGCACCGATTCGATTTCCAAGGCACCTGCGAGTACCTGCTGAGTGCACCCTGCCACGGACCACCCTTGGGGGCTGAGAACTTCACTGTCACTGTAGCCAATGAGCACCGGGGCAGCCAGGCTGTCAGCTACACCCGCAGTGTCACCCTGCAAATCTACAACCACAGCCTGACACTGAGTGCCCGCTGGCCCCGGAAGCTACAGGTCGACGGCGTGTTCGTGGCTCTGCCTTTCCAGCTGGACTCGCTCCTGCACGCACACCTGAGCGGCGCCGACGTGGTGGTGACCACAACCTCAGGGCTCTCGCTGGCTTTCGATGGGGACAGCTTCGTGCGCCTGCGCGTGCCGGCGGCGTACGCGGCCTCTCTCTGTGGCTTATGCGGGAACTACAACCAGGACCCCGCAGACGACCTCAAGGCTGTGGGCGGGAAGCCCGCTGGATGGCAGGTGGGCGGGGCCCAGGGCTGCGGGGAATGTGTGTCCAAGCCATGCCCGTCGCCGTGCACCCCAGAGCAGCAGGAGTCCTTCGGCGGCCCGGACGCCTGCGGCGTGATCTCCGCCACCGACGGCCCGCTGGCACCCTGCCACGGCCTTGTGCCGCCCGCGCAGTACTTCCAGGGCTGCTTGCTGGACGCCTGCCAAGTTCAGGGCCATCCTGGAGGCCTCTGTCCTGCAGTGGCTACCTACGTGGCAGCCTGTCAGGCCGCTGGGGCCCAGCTCGGCGAGTGGAGGCGGCCGGACTTCTGTC |
| circRNA65762 | GTTTTGAGAGATTACCTGAAGTTGCACTCAAAAGCAAGATATACTACTAACTTTGAAATGGAATAAGAACATAAGAGAGCAGCTGCCCATGCCAAATAGTTACCTCTTTTAAACTCATTCTACTGGAGCAAATG |
| circRNA67333 | TCTTGACTCGTCTGCTGAACAAATCCTCTGACCTCAGGCCGGCTGTGAACGTAGTTCCTGAGAGATAGCAAACATGCCCAACAGTGAGCCCGCATCTCTGCTGGAGCTGTTCAACAGCATCGCCACACAAGGGGAGCTCGTAAGGTCCCTCAAAGCGGGAAATGCGTCAAAGGGGAAACTGTGACACTCCCTTGGCCGAGGGGAGAGGGATTCTCAATAACTGCTTCTTGAAGAGACTTCAACCAGCGCTCCTGTTTTCATAACCCGCTTTCCTGCCCCCACTCCT |
| circRNA68796 | GTGCTGTTGATGGGTAAAAGTGGGTCTGGTAAGACCAGCATGAGGTCTATTATCTTTGCAAATTATATTGCCAGAGACACACGTCGCCTTGGCGCAACAATTGATGTAGAACATTCTCATGTTCGATTTCTGGGAAACCTGGTATTGAACCTGTGGGATTGTGGTGGGCAAGACACCTTCATGGAAAATTATTTCACTAGCCAACGGGACAACATCTTCCGAAATGTGGAGGTTCTGATTTATGTCTTTGATGTGGAGAGCCGCGAACTGGAAAAGGACATGCACTATTACCAATCATGCCTGGAGGCCATTCTGCAGAATTCTCCAGATGCCAAAATATTTTGCTTGGTACACAAAATGGATCTGGTACAGGAGGATCAACGGGACCTG |
| circRNA7025 | AGACAGGGTTTCTCTGTGTTGCCCAGGCTGGTCTCGAACTCTGAGCTCAAGCGACCTGCCCACCTCGGCCACCCAAAATGTTGGGATTACAGGGGTTGCTGCTTTTGCTCAGAGGACATCCATGACCCTAATGGTCTTTTTGTTCAAGATAAAGTGATTTTTTGCCTTTGTTGATTAACTGGACAAATTCAG |
| circRNA7089 | GTTTTGAGAGATTACCTGAAGTTGCACTCAAAAGCAAGATATACTACTAACTTTGAAATGGAATAAGGCCCTCCCTATCCTGAAGACATTAGCTGAGAGTCTGGCACCTTTTAAAGGTCTGATCATCAATTGTCTCTCTGGGCGGCCACCTATGAGACTTCATCTGTGTGCTTTATAAATAGCAGTCCAGGGCAACAGTACTTGATAAAAAGGTTATACCTTATTGGTGTGGACGTTGTCTAAATTTCGGTAGCCATGGCACAAGAATATA |
| circRNA72366 | GGGTGAGCTTATGGAACCAAGGAAGAGCTGATGAAGTTGTCAGTGCCTCTGTTGGGAGTGGGGACCTCTGGATCCCAGTGAAGAGCTTCGACTCCAAG |
| circRNA73675 | GTGGAAGAAGAGCCCGAAGAAGAACCTGAAGAGACAGCAGAAGACACAACAGAAGACACAGAGCAAGACGAAGATGAAGAAATGGATGTGGGAACAGATGAAGAAGAAGAAACAGCAAAG |
| circRNA7644 | GAGACGTTGACATTTAGGGATGTGGCCATAGAATTCTCTCTGGAGGAGTGGGAATGCCTGAACCCTGCTCAGCAGAATTTATATATGAATGTGATGTTAGAGAACTACAAAAACCTGGTCTTCTTGGGTATTGCTGTCTCTAAGCAAGACCCGATCACCAGTCTAGAGCAAGAAAAAGAGCCCTGGAATATGAAGATATGTGAGATGGTGGATGAATCCCCAG |
| ciRNA039 | CAGAGGTTGATCTTTGCCGGAAAGCAGCTGGAAGATGGGCGCACCCTGTCTGACTACAACATCCAGAAAGAGTCTACCCTGCACCTGGTGCTCCGTCTCAGAGGTGGGATGCAGATCTTCGTGAAGACCCTGACTGGTAAGACCATCACCCTCGAGGTGGAGCCCAGTGACACCATCGAGAATGTCAAGGCAAAGATCCAAGATAAGGAAGGCATTCCTCCTGATCAGCAGAGGTTGATCTTTGCCGGAAAACAGCTGGAAGATGGTCGTACCCTGTCTGACTACAACATCCAGAAAGAGTCCACCTTGCACCTGGTACTCCGTCTCAGAGGTGGGATGCAAATCTTCGTGAAGACACTCACTGGCAAGACCATCACCCTTGAGGTCGAGCCCAGTGACACTATCGAGAACGTCAAAGCAAAGATCCAAGACAAGGAAGGCATTCCTCCTGACCAGCAGAGGTTGATCTTTGCCGGAAAGCAGCTGGAAGATGGGCGCACCCTGTCTGACTACAACATCCAGAAAGAGTCTACCCTGCACCTGGTGCTCCGTCTCAGAGGTGGGATGCAGATCTTCGTGAAGACCCTGACTGGTAAGACCATCACTCTCGAAGTGGAGCCGAGTGACACCATTGAGAATGTCAAGGCAAAGATCCAAGACAAGGAAGGCATCCCTCCTGACCAGCAGAGGTTGATCTTTGCCGGAAAACAGCTGGAAGATGGTCGTACCCTGTCTGACTACAACATCCAGAAAGAGTCCACCTTGCACCTGGTGCTCCGTCTCAGAGGTGGGATGCAGATCTTCGTGAAGACCCTGACTGGTAAGACCATCACTCTCGAGGTGGAGCCGAGTGACACCATTGAGAATGTCAAGGCAAAGATCCAAGACAAGGAAGGCATCCCTCCTGACCAG |
| ciRNA04125 | ATCACTTTGGGAAGCAGTGACATCCAACCTGTATCAAGTCTTCTAATCTGTGAACATGGTATGCCTCTTCATTTATTTAGGTCTTTCTAGATTCCTTTTAAAAGCATTTTGACATTTCTAGCATAAATATTTTGCACGTTTTGCTAATTTTATACCTATGTACTTCGTTGTTTTGGTGTTTTATAAATAGCAGTTTTAAAATTTCAGTTTCTAATCTTTTGTTGCTAGCACATAAATACACAATTCTATGTTGCAACCTTTACAAACTTATTTATAGTTTTAAATATTTTTCTTTCTGCAGATATCTTTTTTTCTTTTTCTTGAGACTGGCTCTCACTTTGTTCCCCAGGCTGGAGTGCAGTGTCGTGATCACAGCCCACTGCAGCCTCGACCTCCCGGGCTGAAGGGATCCTCGCACCTCAGCCTCCTGAGTAGCTGAGACTATAGGTGTGCACCACCTCACCCAGCCAATTTTTTGTATCGTTGGTAGAGACAGGGTTTCACCATGTTACCCAGATTGGATCTTGAATTCCCAGGCTCAAGGGATCCACCCACCTTGGCCTCCCAAAGTGCTGGGATTATAGGCGTGAGCCACTGCACCCAGCCTCTTTCTGAAGATTTCTTGAGATTTTTCTTTTTCTTTTTCATTTCCTTTCCTTTTTTTTTTTGACAGGGTCTTGCTTAGTCACCCAGGCTGGAGTGCCATAGTGCAAACACAGCTCACTGCAGCCTTGACCTCCCAGGTTCAAGTCATCCTCCCACCTCAGCCTCCCAAATAGCTGGGACTACAG |
| ciRNA0538 | AGGCAGGATCTTGCTCAGTCACCCAGAATGGAGTGCAGGGTGCAAACACTCACTGTAGCCTCAACCTCCTGGGCTCAAGTAATCTTCCCATCTCAGCCTCCCAAGTAGCTGGGACTACAAGCACACACCACCACACCTGGCTAATTATTTTTTGTAGAGATGAGGATTCACCATGTTGTCCAG |
| ciRNA0714 | GTCCGTGTAGAGCACAGGAGAAGCCAGCAGCTGTGACACATTTGTGATCTCACACCAAGCCTGGGAGGAGTTTGGACAGACCAGAGACAGCACATATCCTGGAAAAAAGGAAAGAAGAGAATGTCAGGAATGAAAGGGAGAGGTATTAATGCTTACCGGCTTTCTGCAGGAAACAGAGCCTTGAGTGGATGGTACTGAAACATTTTACTACATGTAGGGAAGTCGCTAACAGGTGGCCCACAGACCTATAGACACTGTCAATTTGGCTTGCACAGATTTTTAAAAATGATTTTTAAGTGTTTAGCTGTCATCATTTAAAAATGAGCTATTTCACATGAAAGCCTGGATTCCCCCTATAAATTCGATCTGGGAACACTTATGTCTTTGTTCTGGTGAATCAGCAACTTGATGGAGCAGCGAATAGCTGTTCAGACCGGCTTGTGCTCAACAGTTCAACACAGCCCATCACATCCACTTCACTCTGCATTATCAGCCTGGCTACTGGAAAGCAGGGCTTGTGGGGCCTGAATGTAGGGAGAAAGCATAGCAACCTCAGTCTAGAAATCACATTGCAAGAAATCTGTTCGCTGGGCATGGTAACTCACACCTGTAATCCCAGCATTTTGGGAGGCCAAGGTGGGATGATTGCTTGATCCCAGGAGTTTGAGACCAGCCTGGGCAACATAGTGAAACCCCATCTACATAAAAAAAATTGAAAAATTAACCGGGCGTGGTGGTGCACGTCTGTGGTCCAAGCTACTCGGGAGGCTGAGGAGGGAGGATCGCTTGAGCCCAGGAGGATGAAGCTGCAGCGAGCTGCGATCACACCACTGCACTCCAGCCTAGGTAACAGCAAGACCCTGTCTCGAAAAAGAAAAAAAGAAAAAAGAAATTGGCTGAGCATTATGGCTGTAATCCCAGCACTTTGGGTGGCCAAGGCGGGTGGATCACTTGAGATCAGGAGTTCGAGACCAGCCTGGCCAACATGGTGAAACCCCATCTCTACTAAAAATACAAAAATTAGCGGGGCATGGGCATGGTGGTGCATGCCTATAGTCCCAGCTACTCTGGAGGTGGAGGCAGGAGAATCACTTGAACCCAGGAGGCGGCTGCAGTGAGCTGAGATCGCGCCACTGCACTCCAGCCTGGGTGACAGAACGAGACTATCTCCAAAAAAAAAAAAAAAAAAAGAAGAAATCTGTCTAACTCATTTGGTGGGGTATGAGTCATTTGGTGGGTATGAAGAGTTATATATTTCAAAAAACGTTTTAAAAGTGGGTTTAGTTTTCTAGTTTAGTTGATTTTTCAGGTCTGTTAATCCACTTTGTCCTAAAGAAAATGGAATCTAATAAAAAGAATTTAAATATCATTAGGCTTTTTAAAAAATGAGATTCAAAGAAGCATGTAAATCCCCTGAACCAAGTCCACTATTATAAATGTCTTGGAATTTTGAGGCTAAACACTAAAATACAAAATGAAAACGTTTTACACTATGTAAGAAACTTTTTATAATCTGTTCTCCACTTGAAAAGACACCAACAAGGTGACATTGTGACAAGATGTTCTGTCTTCCCATCTTTCCTTTAAAAATGGCAAATTCTCCCATTATGAATTACCCTAAACAAAAGAGGGTAAAGGAAAATTAATCAGAAACAATTTTGTAATTCAATTTTTAGAAAAGTTTTACATTTCAAAAATCTTAAGAAGGCATATAAGCAACAAGAGGCAGTTTGATCAAATGATGTCTTAGCAACAAGACTTCCATAAGTGGACCTATGTACCCCTGGCAAAGCTTCATGAAGCCACCTGAATTTTACCACATGCAAAGTTAAGAAACACCCATGAAGTTCAGGTCTGGAAAATAATAAAATAATGGAATTCAAGGTTTTCATGACTATTATTTCATTTATCTCTGTAATTTGAAAATGATCCAACTTTGTTTCAGAAATCCAGAATAGTTGGACATGGTGGCTCACCCTTGTAATCCCAGCACTTAGGGAGGCAGAGGCTAGAGGTTTGCTTGAGCCCCGGAGTTCAAGACCAGCCTGGGCAATATAGCGAGATCTCCACAAAAACAAAAACAAAAACAAAACAAAACAAAGAAATAGAAATCCAGAATAAACTCAAAGTCAAAACTAAATATCCTAACTGCTGCAAATTTTCATTTCTTTTTTTTCTGAGACAGGGTCTCACTCTGTCACCCAGGTTGTAGTGCAGTGGTGTCATCACGGCTCACTGCAACCTTGACCTCCTGGGTTTAAATGATCCTCCCATCTCAGCCTCCCAAGTAGCTGGAACCACAGGCATGCACCACCATGCCCAACTAATTTTTTTAGTTTTTTTTTGTAGAGATGGGCTCTCACTACGTTGGCCAGGCTGATGCTGCAAATTTTCAAGCAAGCTAGCCCTTAGCAATCTTGTTCCACACGATGTTTATCTCATCTAAGCCCCAAGAAGGGAAAGTGATTAAGTATATTTTTGTAAGAATTTTGATTACATTCATTCCTAAGAAAACATCGGACCTGACTTACCAGTGAATTCCATGGCTGCATTCATGTCATTTACTATGCAGTTAACATAACCACTCCAAAACTTTGTCTCTGAAATCAGTGTGGGTAGATCTCATTCCAGTGAAACAATATTCCTTCTAAGCTGTTGATACAGCAAAATGTTTCTGACATTGTAGGGGGTGTGGGAGCAGAGGGTCATAAGGGAGATCAAATGATCACTGTTGCATTTTGCAAGTTCTCATTGCAACTGGCAGGCATGAATGCTCCAAGTTTTATGGCAGGAAAAATAATAAAAATCTTGTCTGGTTACTGACCTTAGGACTTAAATTGTTTTATGCTTTGGGAACATGTTAGGGAGAGAGCCTACAGCAGATATGACTGAAAGAGGAAAGAAAGGATGGGTTAGTGGATGTGGAGTGGCCTACGGGAGAAGGGAAGAGATGGGTAATGGATGGTGTGTGGGCAGGGAATTTGGAGTGTTTTAAAGGAATCAAAGACATCTCAAGTCACAATTGGCTTATCAAGATTTAAGTAAGCACAGTAATTTTCCAGGGAGTTTAATCCAGACTTTATTCCACAGAAAGAGAACATCTACTCCATTCATTATGCTTCATTTTTAACAGACACTATCAATAGTCCTCTAAAGTGACTGTACCAAAGTACACTCTTGCTAGCCACGAACATGTACTTCTTCTTTGATGTTATTTTTCTAAATAGAGAATAATGAATATTTAGGAAATATATATTATAAATATATATATATATACACACCAATGTATCAACCAGCCAGTTTAAGAAATAAAGCATCACCCCTCAGAGTTAGTAACTATCCTGATTTTCTCTTTAACATTCTTTTGCTTATAAAAAAGATTTTCTCCATATATCTTGAAAATGCTTTTGCCTCTTTTTGCACCTATGAAAGTGTAATGATAAAGAATTTGTTCTTAGGTGACTTGTTTTTGAGATTCATCAAAACATGATGCATGAGGCTGCAGGATTTGTTTTTTCCCCACTGTTCTAGAGAATTCCACTGTATAACTATCACACAACTAATTTTTCTGTTTTATAGTTGATTGATATTTAAGTAATTTACAGGTCCCACAAACAGTACTTCTAATGTTCTTATATTATGTTGAACTATATGAAATTGATGATAACTGAACAATTTGAACCCTATGACAAGTAGTTTCATATGTTTCAACCTAATATTTATCTTCTATGTACAGGTGTAATAGTTTACATACACACACCTTTAACATTTAAAAATGAAGATCTACTATCTATAATCATGGGCAAGTTGAGTAATGGCTCTTGGTTTCAGTTTCCGCATCTGTAAAATGAGGGACTTTGACTCAATGATTCTAAAGTTTGATCATAACCTATGTTTGTTCTCAGAATTACTTACAATACTGGTGCCTCATAAATGTTGCTGATGGACCAGTTCTAAACTCATAGGCACTGATAGAGGAGAGATATTTTAAAGAAACTGATGCCAATATATTAAGTGACACTGGCCAAAGTTATGTAACTGCTAAAACCAGGGCCAAGTAACCCAATTCCCAGCCAGCATTCTATTTCAGAAAAATAAAAACATAAAGTACAAGAAAAAGCCAGTTGAAATTTATTATTCATGTTTATATAAGGATGAAATGCTTTAAATAATTTGTCAAATATTTGCCTATAGAGAATAATAATATAAATTTAAATAATAATTTAAATTATCCAGTTTATTACATGGAATAGAAATCTGGTTTTAAGAAAGTCAATAATTGTATAAAACTACCTTTTTAATCACCGCAAGATATTTCCAGTTGGGGGTCTCACCATCTATTCTACTGTGATAGTTTGGATTATTATTCATTCAGTATTCAGCCCCTTACCTTTCCCAATGTGGGCAAAGGTGCTTCACCTGATGTTGGGCTGGAAGTAAGTTGGTGGCCAATGGGAAGTCAGCAGAAGTGATATGAAGCTAGATTTCAAAGATGCTTATGTGGTTGGGTTTACTCATTTGCATCTCTGTAATTCCCTGAGGAGCTGCTGTCCCTTCAGCCTCTGCACCCAAATGAAGACACATGGAGCAGATCTGAGCTCATCCCACATGAAGGAGCCAAACCCAGGTGGACATTAGCCTGAAGCAGAGACATATAG |
| ciRNA0799 | GTAAGTACTTAAGGATGAGGAGGGTAGAGCAGGGCATGGAGCGAGCTGGGATTGAGGGTTTCACAGTGTGGGGAGTAGATGGAAAGAGCAGCTGTGTTGGTGGACATGGGTTAGGTGAAGCCAGGGAGGGAAGAGAAGGCCTTATAATATTAGTCATGGGCCTAACCCTACTTCTGGCATCAAGATTAATTCTGTTCTCATCCCCACCCGGCTT |
| ciRNA2501 | TAGAGACGGGGTTTCTCCATGTTGGTTAGGCTGGTCTCGAACTCCCGACCTCAAGTGATCCGCCTGCCTTGGCCTCCCAAAGTGCTGGAATTACAGGCATGAGCCACCATGCCCGGCCCACTCCCAGTTTTGAAAGTTCTATTATGAGTAAAATGCTATCAGACAGCATCGCATGCTACAGAGAAATCTTTGTGAAAGGAAGAGTCAATGGATGTGGCAAACTTCATTGTTGTCTTAGTTTAAGAAATTGCCATAGCCATCACAACCTTCAGTAGCCACTTCTCTCATCAGTCAGCAGCCGTCATCGAGGCAAGACTCTCCACCAGCACAAAGATTACAACTCAACTGAAGGCTCAGATGATTATTAGCATTTTATAGCAATGATGTATTTTAAAATTAAGGTATATACATTGTCTTTTTAAATATAACTTTATTGCACACTTAACATAATTCAATATAGTATAAACATAACTTATATATGCCTTGGGAAATCAAAAAATTTGTGTGACTTGTTTTGTTGTGGTGGTTTAGAACCAAACCCACAATATCTCCCAGATATGTCTGTATATTTGTGTGATTATATGAGATTTGGACCAATTCCTAATTTTAGAATAATATATTTCCCCTGTATGCCACTAAAGAATTAGTACTGTTATTGTTAGACTAGAACCTAACATTGCCTATGGGTCTCATAACAATGAATACTTCACTATTTGTTGAATTAGAATGTATATTGACATATTAACAATTAGTAGAAAGGAGCTTTATTCAAAGAATATGAAGAAGCCCAAGTTTAACTTGAATATTTAGGAATACATAAAGGTAGGATTAAATGTTTCTTCTTAATTTATTATAGCTTCTATATGTTTGTTAATATGTATATGTATATGCTTAAAAATATACTTTAAGTAAATTAAAGTAATTGTGATATACTTTTAATAGTGGAACTTGGAAAATTATTTTTAATAGTTGATCTGATTCTAAGTTAGAAGAGTAAATAATTGATTCATAGGAATTCTTATTTAAACAAAATTTGTGTATTTCTCAGTAATTGCCTTTTAAAATTTTCCTTTTATAGGTAGACTGGCGTCGTTCCTTCATCACCACTGATGTTAATCCTTACTATGATTCATTTGTCAGATGGCAATTTTTAACATTAAGAGAAAGAAACAAAATTAAATTTGGGAAGCG |
| ciRNA6406 | ATGCAGTCTCGTTCCATTGTCCAGGTCAGAATGCAGTGGCGCAATCTCGGCTCACTGTCACCTCCGCCTCCCGGGTTCAAGCGATTGTCCTGCCTCAGCCTCCCGAGTAGCTGAGATTACAGACATGTACTGCCACGCCTGGCTAATTTTTGCATTTTTAGTAGAGATGGGGTTTCTCCATGTTGGCCAGGCTGGTCTTGAACTCCTGACCTCAAATGATCTACCTGCCTTAGCCTCCCAAAGTGCCGGGATTACAGGTGTAAGCCACCATGCCCGGCCAGTTTGATTTTTTATTGTGGTAAAATACATACAAAATCTATTATTTCAGCCATTTTCAAAGGAAAAATTCAGTGGTGTTAAGTGCATCCACCACATTGTACAGCCATGACCCCCATCCATCTCCAGAACGCTTTCATACTGTCCTGCAAATATGCAGCACCTTGCTACACTCCAGGTTGTTTGTCCCACAACAGAGCTGGGCTGAATTATTAATGTGACTTTGTTCAACAACGGACTAAAGAGGGAGAAGCCCATGAACTGCGTGAGGAGTGCATGACAGGTGCTCGTGGGATAACATGGCTCGGCGCCCTCCAGCTGCTGCTACCGCTGCCTGTCCTGCTGGGCGGCCACCTCCTCCCAGGGAAGAAGAGCACTCACAACTGCTGCTGATCTCCTTCCAGGGCTTCCGCTGGGACTAGGATCAGGATGTGGACACCCCCAACCTGGACCGTCTGGCCGGGGAGGGCGTCAAGGCCAAGTACCTCATGCCGCCCCTTGTCACAATGACCTCCCCATCCCACTTCACTGCCATCCCAG |
| ciRNA6456 | ATTACAGGCATGAGCCACCGCACCCGCCCATTCTCTTGTTAAATCAGAGATTAAACTTTAACATAATGGACACTCTAGAATTCTAGAAGTAGGATGGTGAAAATGCAGTGGGATGAGTACCTGTCCCACCTGTGGGCACCTGTGGGCCTGGATAAGTGAGAAATCCCACCTAAGAAGAGGAGTAGAGGGGGCCACTGGAAAGTCCCAGCCAACAAGACAACAATGTTAACAATTGAACTCAAGCACTCTCCATGGCCTGTTGAATGTTTTCTGTGGAAACTGGTCCAGATGCACATGCACACAACACCTGATAGAAACTGGGAGGTATTTAAGCCCCTTAGTGCAGAGTCTGCAATCACAAGTCTCAGTGGTAATCGAAAGGTGATCAAGAAAAGCCTTTGCTGGCCTTCCTGGACTTGCCTCCTTGCTTGCAGAAACCTAAAGTGCCTGTCAGTCCTTCCTCTATATGATCTGGCGAAACTTCCTATCTTCGTCCTTCCTTTCTTTCTCTATAGCTCCCTGTGACCTTTAGGATAAGTCTTTAAAAGTATTAAAACATCCTACATTGTAGCCAAGCTCACCATCTATTGGTAATAGATTTCTTTGCAAATGGGCCCTTTCACACATTAGCTAGGACGCAAAGTCCACTGCCCACCAGCTCTTTAATCCTCTCTACCTTGGTATACTGCATTTCTTGCAATTTGAATTTATATAACTGTAACTTCTATTTCAGGACACTAAAAAATGCACCTGCTATTTGCCTGAGGGACCAGAGACACGTGGAAAGTTTAACAGTAGCAGCCCACAACATGTACCACTGGCAGCTGGACTTTCACTGAATTTCTACACTGCAACCAAAGGTCAAGACAATGGTGAATCATGGAAAGACTTCAACTTTGAAAGCCTTACAAACAAAAGAGAGAGGATGCATTTGTTCAAAATACTGGCTTTTCTTTTCAGCAATTTTTCCATTTTACACTAGGAGCTTCTTTTCAAACGCTGCACAAAACAGTTACAAACCAACAGCTATGAATTCCCTTGCTTTAATATCCATTTCCCTTTGGCCATCTCATGTGAATCAAATTTCCAACAGCAAAAACAGAGTGATGTTTGCCTTTCTGAATTCCAGTGTACTGCTTTGGCATACCGAGGTTCCATCGCCTTATGACCCACAAAGATTAATGATCAGAAAATCAGAGTAAAGTTTTAAAATACTCCCATCTTGTGGCAGTTTTGAAATATGGCAACCTGAATATGACAGTCCCGACTTATCAGTCCTTATTGTTCATTTGTCCCTCTGCTGTAGATAAGGTTCCAGTAAATTCAACACACCTGGATGGAGAAAGTTCTAAAACCATCAAGGAAGTGGCAAACTTGTAAACTAAAATTGACAAAACATTAGGCTGGGTGCAGTGGCTCTTGTCCAAGCACTTTAGGAGGCTGAG |
| ciRNA6687 | GCCACCATGAGACCTCAACCCCTGCAGGACCCAGCCAAGCTGACCTTCACACTCCCCACACAGAGGATGGAGGTCCTTCT |
| ciRNA731 | ATCACTTTGGGAAGCAGTGACATCCAACCTGTATCAAGTCTTCTAATCTGTGAACATGGTATGCCTCTTCATTTATTTAGGTCTTTCTAGATTCCTTTTAAAAGCATTTTGACATTTCTAGCATAAATATTTTGCACGTTTTGCTAATTTTATACCTATGTACTTCGTTGTTTTGGTGTTTTATAAATAGCAGTTTTAAAATTTCAGTTTCTAATCTTTTGTTGCTAGCACATAAATACACAATTCTATGTTGCAACCTTTACAAACTTATTTATAGTTTTAAATATTTTTCTTTCTGCAGATATCTTTTTTTCTTTTTCTTGAGACTGGCTCTCACTTTGTTCCCCAGGCTGGAGTGCAGTGTCGTGATCACAGCCCACTGCAGCCTCGACCTCCCGGGCTGAAGGGATCCTCGCACCTCAGCCTCCTGAGTAGCTGAGACTATAGGTGTGCACCACCTCACCCAGCCAATTTTTTGTATCGTTGGTAGAGACAGGGTTTCACCATGTTACCCAGATTGGATCTTGAATTCCCAGGCTCAAGGGATCCACCCACCTTGGCCTCCCAAAGTGCTGGGATTATAGGCGTGAGCCACTGCACCCAGCCTCTTTCTGAAGATTTCTTGAGATTTTTCTTTTTCTTTTTCATTTCCTTTCCTTTTTTTTTTTGACAGGGTCTTGCTTAGTCACCCAGGCTGGAGTGCCATAGTGCAAACACAGCTCACTGCAGCCTTGACCTCCCAGGTTCAAGTCATCCTCCCACCTCAGCCTCCCAAATAGCTGGGACTACAGGTGTGCCACTACACTTGGCTAATTTTATTTTATATTTTGTAGAGATGGGGTCTTGCTTTGTTGATAGGGCTGGTCTCGAACTCCTGGGCTCAAGCGATCCTCCTGCCTCAGCTTCCCAAAGTGCTGGGATTATAGGCATGAATCACCACACCCAGCCCTTGGGATTTTCTTTTTTTCTTTTCTTTTCTTTTTTTTTTTTTTTGAGACGTAGTCTCCCTCTGTCGCCCAGGCTGGAGTGCAGTGGTGCGATCTCAGCTCACCGCAAGCTCTGCTTCCCGGGTTCATGCCATTCTTCTGCCTCAGCCTCCCGAGTAGGTGGGACTACAGGTGCCCGCCACCATGCCCAGCTAATTTTTTGTATTTTTAGTAGAGATGGGTTTCGCTGTGGTCTCAATCTCCTGACCTCGTGATCGGCCCGCCTTGGCCCTCCAAAGTGCTGGGATTACAGGCTTGAGCCACTGCGTCTAGCCCAACCTGTGGGATTTTCTATGGAGACAATCAGGAGTTTTGGGTAGGGACAATTTTGATTCTTCCTCTTCTATCTGTATGCTCTTTATTTCTTTTCTTGCCTTATTGCCCTACTTTGGTCTTCCAGTACAATGTTGAACAGGAGCGATGAGAATGAACATCCTTATCTTGTACCTTAACTTGGGTGAACACATTCAGTCTTGAAGTATGACTTTAGCTCTGGATTTTTGGAGATGTCCTTTATCAGGTTGAGGAAGTTCCCCTTTATTCCTATTTTGAAAAGAGTTTTTATTTAGCATGGATGTTGAATCTTCTCAAATGCTTTTTCTGCATATGATACATTGATACGTTGATATGATCATATTGATATATTGATTATTTTAAGAAATGGATACATTGATCAATGTGTTGGTATGTTTTTCTTCTGTTAATAACATACACTGCATTGATTGATTTCAGATATTGAACCAGCCTTGCATTGCTGATATAAAACTGCCTGGTTGTGATTTATTACTGTTTTATATATTGGTGCATTCTGTTTGCTACTTTTTTTGAGTTTTTTTGGTCTATGTTCATAGAGACTATTGGTCTGTGTAGACTTCTGTTTTTGTACTACCATAGTCTAGTTTTGATGTAAAGGTAATGTTGGCTTTATACAACAATTTTGGAAGTGTTCATTTCCCTATTTTCTGGAAACAATTGTGTAGAATTGGTGTTTTTTCTGTTTTAAATCTTTGGTAAGAATTATCTGGAGAAATTATCTAGGCCTTGAGATTTCCTCTTGAAAGGTTTTTTAACAACAAATTCAATTTCGTTAATAGTATGGGACTATTCCCATTCTCTGTTTTACCTTGTGTGAGTTTTGGTAGTTTGTGGTTTTTCAGAAATTGGTTCATTTCATCTACATTGTTAAATTTATGATATAGACTTGTTCATAATATCCCCTTATTATCCTTCTAACATTTGTAGAATTTGTAGTGATATCACGTATTTCATTCCCAATATTGATAATTTATGTCTCGTTTCCTTTCTTTTCTCTCTTTTTTTGTTATTTGGCTAGAGGTTTGGGAATTTTACACATCTTTTCAAAGAATCAGCTTTTGATTTAATTGATTTCTCTATTGTTTTCAGTTTCATTGGTTGCTGCTCTTACCTTTGTTACTTCCTTCTTTGTGCTTACCTTGGGTTGATTTTACTCTTTTTATAGTTTCTTAAAACAGAAATTTAGGGCCAGGCGCAGTGGCTCATGCCTGTAATCCCAGCACTTTGGGAGGCCCAGGCGGGCGGATCATGAGGTCAGGAGATCATGACCATCCTGGCTAACATGGTGAAACCCTGTCTCTACTAAAAATACAAAAAATTAGCCGGGCATGGTGGCTGGCTCCTGTAGTTCCAGCTACTCGAGAGGCTGAGGCAGGAGAATGGTGTGAACCCGGGAGGCGGAGCTTGTAGTGAGCCAAGATCCTGCCACTGCACTCCAGCCTGGGCGACAGAGCGAGACTCCGTCTCAAAAAAACAAAACAAAACAAAAAACAGAAATTTAGGTTATCGATTAGAAAACTTTCGTCGTCCTCCTCCTCCTCCTCTTCTTCTTCCTCCTCTTTCTTCTTCACTACTTCTTCTCCTTCTCTTTCTCTTTTTCCTTCTCTTTCTCTTTCTCCTCCTTCTCCTCTCCTCCTCCTCCAACTCCTCCTCTTCCTTTCTTCTTTCTTCTTCTCCTGAGACAGGGTCTCACTCTATCACCTAGGCTGGAGTACCATGACAGGATCCTAGCTCACTGCATCCTCAAACTCATGGGCTCAAGTGATTCTCCTGCTTTAGTCTCCCGAGTAGCTGGGACTACAG |
| hsa_circ_0000439 | AGGTCGAAACAGTAACAAAGGACTGCCTCAGTCTACGATTTCTTTTGATGGAATCTATGCAAATATGAGGATGGTTCATATACTTACATCAGTTGTT |
| hsa_circ_0000471 | AATTCTGCTTGGTCAGAATCGTGATGGCATTGTGTTCAGCACTGATGACTATTTTCACCATCAAGATGGGTACAGCAAAACAAGCTATCGATCAGGGAAGATCTCCAGTTATAATAGATAACACTAATATACAAGCTTGGGAAATGAAGCCATATGTGGAAGTGGCCATAGGAAAAGGATACAGAGTAGAGTTTCATGAACCTGAAACTTGGTGGAAATTTGATCCTGAAGAATTAGAAAAGAGGAATAAACATGGTGTGTCTCGAAAGAAGATTGCTCAGATGTTGGATCGTTATGAATATCAAATGTCCATTTCTATTGTAATGAATTCAGTGGAACCATCACACAAAAGCACACAAAGACCTCCTCCTCCACAGGGGAGACAGAG |
| hsa_circ_0000511 | TTCCCAGAGAACGGGGCTCCGCGCGAGGTCAGACTGGGCAGGAGATGCCGTGGACCCCGCCCTTCGGGGAGGGGCCCGGCGGATGCCTCCTTTGCCGGAGCTTGGAACAGACTCACGGCCAGCGAAGTGAGTTCAATGGCTGAGGTGAG |
| hsa_circ_0000513 | GTGAGTTCCCAGAGAACGGGGCTCCGCGCGAGGTCAGACTGGGCAGGAGATGCCGTGGACCCCGCCCTTCGGGGAGGGGCCCGGCGGATGCCTCCTTTGCCGGAGCTTGGAACAGACTCACGGCCAGCGAAGTGAGTTCAATGGCTGAG |
| hsa_circ_0000520 | GGAAGCTCATCAGTGGGGCCACGAGCTGAGTGCGTCCTGTCACTCCACTCCCATGTCCCTTGGGAAGGTCTGAGACTAGGGCCAGAGGCGGCCCTAACAGGGCTCTCCCTGAGCTTCGGGGAG |
| hsa_circ_0000586 | GTTAAAAATCTGTAAGAGCCTGATTTTAGAATTCACCAGCTCCTCAGAAGTTTGGCGAAATATGAGTTATTAAGCCTACGCTCAGATCAAGGTAGCAGCTAGACTGGTGTGACAACCTGTTTTTAATCAGTGACTCAAAGCTGTGATCACCCTGATGTCACCGAATGGCCACAGCTTGTAAAAGAGAGTTACAGTGGAGGTAAAAGGAGTGGCTTGCAGGATGGAGAAGCTGCACCAGTGTTATTGGAAATCAGGAGAACCTCAGTCTGACGACATTGAAGCTAGCCGAAT |
| hsa_circ_0000591 | GATGGGAAGGCCATTGTGACTATGTGGTGATTACAGTTGTCTTACTACTGAGTTTCCTACTGAAATCATGGAGGAGAAACAGCAGATTATATTGGCTAATCAAGATGGTGGAACAGTGGCAGGAGCAGCACCTACCTTCTTTGTCATCTTAAAGCAGCCAGGAAATGGCAAAACTGATCAAGGAATTTTGGTTACTAATCAGGATGCCTGTGCTTTGGCTAGTAGTGTGTCATCACCAGTAAAATCTAAAGGGAAGATTTGCCTTCCAGCTGATTGTACTGTGGGTGGAATCACTGTTACCCTCGATAACAATAGTATGTGGAATGAGTTCTATCATCGAAGCACAGAGATGATTCTGACCAAGCAAGGAAGACGCATGTTTCCTTACTGTCGTTATTGGATAACAGGTTTAGATTCAAATTTGAAGTATATTCTTGTCATGGATATATCTCCTGTGGATAACCATCGTTATAAGTGGAATGGTCGTTGGTGGGAACCTAGTGGGAAGGCTGAACCTCATGTTTTGGGGAGGGTTTTCATTCATCCAGAATCTCCTTCCACAGGTCATTATTGGATGCATCAACCAGTATCTTTCTATAAACTCAAACTTACCAACAATACACTGGACCAAGAAGGGCATATCATCTTGCACTCTATGCATCGTTACCTGCCGAGGCTTCATTTGGTGCCTGCAGAAAAGGCTGTGGAGGTGATACAATTAAATGGCCCTGGTGTCCACACTTTTACCTTCCCACAGACTGAATTCTTTGCAGTAACAGCTTATCAGAACATTCAGATTACTCAGCTGAAAATAGATTACAATCCATTTGCCAAAGGCTTTCGGGATGATGGGCTGAATAATAAGCCCCAGAGAGATGGAAAACAAAAGAACAGCTCTGACCAAGAAGGGAATAATATTTCCAGTTCTTCTGGTCATCGGGTCCGTCTTACAGAAGGTCAGGGGTCAGAGATACAACCAGGTGATTTGGATCCTTTGTCAAGGGGTCATGAAACATCAGGCAAGGGTTTGGAGAAGACTTCCCTTAATATAAAACGAGACTTTCTTGGTTTCATGGATACTGATTCAGCACTTAGTGAAGTTCCTCAATTGAAGCAAGAGATTTCTGAATG |
| hsa_circ_0000620 | ATATCCTTGGAACAGAAGATCTTATTGTGGAAGTGACTTCCAATGATGCTGTGAGATTTTATCCCTGGACCATTGATAATAAATACTATTCAGCAGACATCAATCTATGTGTGGTGCCAAACAAATTTCTTGTTACTGCAGAGATTGCAGAATCTGTCCAAGCATTTGTGGTTTACTTTGACAGCACACAAAAATCGGGCCTTGATAGTGTCTCCTCATGGCTTCCACTGGCAAAAGCATGGTTACCTGAGGTGATGATCTTGGTCTGCGATAGAGTGTCTGAAGATGGTATAAACCGACAAAAAGCTCAAGAATGGTGCATCAAACATGGCTTTGAATTGGTAGAACTTAGTCCAGAGGAGTTGCCTGAGGAGGATGATGACTTCCCAGAATCTACAGGAGTAAAGCGAATTGTCCAAGCCCTGAATGCCAATGTGTGGTCCAATGTAGTGATGAAGAATG |
| hsa_circ_0000643 | GTGTGCAACTGAGGAACATGGCTCAAGAAACTAATCACAGCCAAGTGCCTATGCTTTGTTCCACTGGCTGTGGATTTTATGGAAACCCTCGTACAAATGGCATGTGTTCAGTATGCTATAAAGAACATCTTCAAAGACAGAATAGTAGTAATGGTAGAATAAGCCCACCTGCAACCTCTGTCAGTAGTCTGTCTGAATCTTTACCAGTTCAATGCACAGATGGCAGTGTGCCAGAAGCCCAGTCAGCATTAGACTCTACATCTTCATCTATGCAGCCCAGCCCTGTATCAAATCAGTCACTTTTATCAGAATCTGTAGCATCTTCTCAATTGGACAGTACATCTGTGGACAAAGCAGTACCTGAAACAGAAGATGTGCAGG |
| hsa_circ_0000711 | ATCTTGAGCCAGATGATTGTGCATCCATTTACATCTTTAATGTAGATCCACCTCCATCTACTTTAACCACACCACTTTGCTTACCACATCATGGATTACCGTCTCACTCTTCTGTTTTGTCACCATCGTTTCAGCTCCAAAGTCACAAAAACTATGAAGGAACTTGTGAGATTCCTGAATCTAAATATAGCCCATTAGGTGGTCCCAAACCCTTTGAGTGCCCAAGTATTCAAATTACATCTATCTCTCCTAACTGTCATCAAGAATTAGATGCACATGAAGATGACCTACAGATAAATGACCCAGAACGGGAATTTTTGGAAAGGCCTTCTAGAGATCATCTCTATCTTCCTCTTGAGCCATCCTACCGGGAGTCTTCTCTTAGTCCTAGTCCTGCCAGCAGCATCTCTTCTAGGAGTTGGTTCTCTGATGCATCTTCTTGTGAATCGCTTTCACATATTTATGATGATGTGGACTCAGAGTTGAATGAAGCTGCAGCCCGATTTACCCTTGGATCCCCTCTGACTTCTCCTGGTGGCTCTCCAGGGGGCTGCCCTGGAGAAGAAACTTGGCATCAACAGTATGGACTTGGACACTCATTATCACCCAGGCAATCTCCTTGCCACTCTCCTAGATCCAGTGTCACTGATGAGAATTGGCTGAGCCCCAGGCCAGCCTCAGGACCCTCATCAAGGCCCACATCCCCCTGTGGGAAACGGAGGCACTCCAGTGCTGAAGTTTGTTATGCTGGGTCCCTTTCACCCCATCACTCACCTGTTCCTTCACCTGGTCACTCCCCCAGGGGAAGTGTGACAGAAGATACGTGGCTCAATGCTTCTGTCCATGGTGGGTCAGGCCTTGGCCCTGCAGTTTTTCCATTTCAGTACTGTGTAGAGACTGACATCCCTCTCAAAACAAGGAAAACTTCTGAAGATCAAGCTGCCATACTACCAGGAAAATTAGAGCTGTGTTCAGATGACCAAGGGAGTTTATCACCAGCCCGGGAGACTTCAATAGATGATGGCCTTGGATCTCAGTATCCTTTAAAGAAAGATTCATGTGGTGATCAGTTTCTTTCAGTTCCTTCACCCTTTACCTGGAGCAAACCAAAGCCTGGCCACACCCCTATATTTCGCACATCTTCATTACCTCCACTAGACTGGCCTTTACCAGCTCATTTTGGACAATGTGAACTGAAAATAGAAGTGCAACCTAAAACTCATCATCGAGCCCATTATGAAACTGAAGGTAGCCGAGGGGCAGTAAAAGCATCTACTGGGGGACATCCTGTTGTGAAG |
| hsa_circ_0000825 | GATGAGTTAGATGAACTCCGTGCTGAGATGGAAGAGATGAGAGACAGTTATTTAGAGGAAGATGTTTACCAGCTGCAGGAACTTCGGCGAGAACTGGACCGCGCTAATAAAAACTGCCGAATCCTGCAGTACCGTCTTCGGAAAGCCGAGCAGAAAAGCCTGAAAGTGGCTGAGACGGGTCAGGTGGATGGTGAGCTTATTCGAAGCCTGGAGCAGGACTTGAAGGTAGCCAAAGATGTATCTGTCAGATTGCACCACGAACTTAAGACGGTGGAGGAAAAGCGCGCTAAAGCTGAGGATGAAAACGAAACTCTCCGACAGCAGATGATTGAAGTGGAAATATCCAAACAGGCCCTCCAGAATGAGCTGGAGAGACTGAAAGAG |
| hsa_circ_0000826 | ATCCAGGATGAGAAGACTGATAAAAGAAGAAGCTAGCTGAACAGCTGTAAAATGCCCAAATCTGGGTTCACAAAACCAATTCAGAGTGAAAATTCTGACAGTGACAGCAATATGGTAGAGAAACCATATGGAAGAAAGAGTAAAGACAAGATTGCATCCTACAGCAAAACTCCAAAAATTGAACGAAGTGATGTGAGCAAGGAGATGAAAGAGAAATCATCCATGAAACGTAAACTTCCTTTTACTATTAGCCCATCAAGAAATGAAGAACGAGATTCAGACACAGATTCAGATCCAGGACATACAAGTGAAAATTGGGGGGAGAGACTTATATCTTCTTACAGGACATACTCAGAGAAAGAAGGTCCAGAAAAGAAGAAGACAAAAAAGGAAGCTGGAAATAAGAAATCCACACCAGTTAGCATTCTTTTTGGTTATCCACTCTCTGAGCGAAAACAGATGGCACTTCTTATGCAGATGACAGCAAGAGACAACAGTCCAGATTCCACACCAAATCATCCATCACAAACAACGCCTGCCCAAAAGAAAACTCCCAGTTCTTCATCTCGACAGAAAGATAAAGTTAATAAAAGAAATGAACGTGGTGAAACTCCTTTACACATGGCTGCTATTCGAGGAGATGTGAAACAAGTTAAAGAATTAATAAGTTTAGGGGCAAATGTGAATGTGAAAGATTTTGCAGGTTGGACACCACTGCATGAAGCTTGCAATGTTGGATATTACGATGTTGCTAAGATACTTATAGCAGCTGGAGCAGATGTTAACACACAAGGATTAGATGATGACACTCCACTCCATGATTCTGCTAGTAGTGGGCACAGAGATATAGTAAAGCTGTTACTTCGTCACGGTGGAAATCCATTTCAAGCTAATAAACATGGGGAGCGTCCAGTGGATGTAGCAGAAACAGAGGAGTTGGAGTTGCTACTAAAAAGAGAGGTGCCTTTATCTGATGATGATGAAAGTTACACAG |
| hsa_circ_0000907 | CTCCAGGACACGCGCGCCCCGAGCCTGGGAGGCATGCTGAAGCCAGGCGGCCGGCAGGATGAGTGTGAAAGAGGCAGGCAGCTCGGGCCGCCGGGAGCAGGCGGCCTACCACCTGCACATCTACCCCCAGCTGTCCACCACCGAGAGCCAGGCCTCGTGCCGCGTGACTGCCACCAAGGACAGCACCACCTCGGACGTCATCAAGGACGCCATTGCCAGCCTGCGGCTGGACGGCACCAAATGTTATGTGCTGGTGGAGGTCAAAGAGTCGGGAGGCGAGGAATGGGTGCTGGACGCCAACGACTCGCCTGTGCACCGGGTGCTGCTATGGCCCCGGCGGGCACAGGACGAGCACCCTCAGGAGGATGGCTACTACTTCCTGCTGCAGGAGCGCAACGCAGATGGAACCATCAAGTACGTGCATATGCAGCTGGTGGCGCAGGCCACAGCCACCCGGCGCCTAGTGGAGCGTGGCCTCCTGCCACGGCAGCAGGCGGACTTTGATGACCTGTGTAACCTCCCCGAGCTAACCGAGGGCAACCTCCTGAAGAACCTCAAGCACCGCTTCCTGCAACAAAAGATCTACACGTACGCGGGGAGCATCCTGGTGGCCATCAACCCCTTTAAGTTCCTGCCCATCTACAACCCCAAGTACGTGAAGATGTATGAGAACCAGCAGCTGGGCAAGCTGGAGCCACACGTCTTCGCGCTGGCCGACGTGGCCTACTACACCATGCTCAGGAAGCGCGTGAACCAGTGCATCGTGATCTCGGGTGAGAGCGGCTCCGGCAAGACCCAGAGCACCAACTTCCTCATCCACTGCCTCACCGCCCTCAGCCAGAAGGGCTACGCCAGCGGCGTCGAGAGGACCATCCTGGGTGCTGGCCCTGTGCTGGAG |
| hsa_circ_0000963 | GGATACCTCAGACAATGGCAGCTTTTGGATTGAAATATTTGCTGAATGACACTGGATGTAAGTTTGTCTTTTATTATCACAGGGTGGGCACAAGAGTTTCCCTCAGCACCATCTGCCTTCTCAATGGATTCCAAGCCATTAAGCTCAACCCCAGTATATGCAGGTGGATGGAGATCAAGATTAGATCCCCAAGGTTTATTGACTTCTGTTGTCTCCTCTGCTGGGCCCCCCATGTCTTGATGAATGCATCTGTTCTTCTATTAGTGAATGGCCCACTGAATAGCAAAAACAGTAGTGCAAAAAACAATTATGGATACTGTTCTTACAAAGCATCAAAGAGATTTAGCTCATTACATGCAGTCTTATATTTTTCCCCTGATTTTATGAGTTTGGGCTTCATGGTCTGGGCCAGTGGCTCCATGGTCTTCTTCCTCTACAGACACAAGCAGCAAGTCCAACACAATCACAGCAACAGACTCTCCTGCAGACCTTCCCAGGAAGCCAGAGCCACACACACCATCATGGTCCTG |
| hsa_circ_0001006 | TTGTTGACCTCCTGTACTGGAGAGACATTAAGAAGACTGGAGTGGTGTTTGGTGCCAGCCTATTCCTGCTGCTTTCATTGACAGTATTCAGCATTGTGAGCGTAACAGCCTACATTGCCTTGGCCCTGCTCTCTGTGACCATCAGCTTTAGGATATACAAGGGTGTGATCCAAGCTATCCAGAAATCAGATGAAGGCCACCCATTCAGGGCATATCTGGAATCTGAAGTTGCTATATCTGAGGAGTTGGTTCAGAAGTACAGTAATTCTGCTCTTGGTCATGTGAACTGCACGATAAAGGAACTCAGGCGCCTCTTCTTAGTTGATGATTTAGTTGATTCTCTGAAG |
| hsa_circ_0001009 | GGAAGAGACTTCCACCTTAGGATAGTGTTGCCTGAAGATTTACAACTGAAGAATGCAAGATTATTATGTAGTTGGCAGCTGAGAACAATACTTAGTGGATACCATCGAATAGTACAACAGAGAATGCAGCACTCTCCTGATCTAATGAGCTTTATGATGGAGTTGAAGATGCTTTTGGAAGTTGCCTTAAAGAATAGACAAGAGCTGTATGCACTACCTCCTCCTCCCCAGTTCTACTCAAGCCTTATTGAAGAGATAGGAACTCTTGGTTGGGATAA |
| hsa_circ_0001060 | CTGTTTGGGGCAACTTCGTTAATATGAGGTCTATCCAGGAAAATGGTGAACTAAAAATTGAAAGCAAGATTGAAGAGATGGTTGAACCACTAAGAGAGAAAATCAGAGATTTAGAAAAAAGCTTTACCCAGAAATACCCACCAGTAAAGTTTTTATCAGAAAAGGATCGGAAAAGAATTTTG |
| hsa_circ_0001092 | CTCCATAATGGGAGAAGTAAAGAACAAAGACTTAAGGAACAGCTTGGCGCTCAACAAGAACCAGTGAAGAAATCCATTCAGGAATCAGAAGCTTTTTTGCCTCAGAGCATACCTGAAGAGAGATACAAGATGAAGAGCAAGCCCCTAGGAATCTGCCTGATAATCGATTGCATTGGCAATGAGACAG |
| hsa_circ_0001122 | TGGAAGAACTCAGAAACAACTAGTAGATTATTTCAAAGACAGTGGAATGAAGAGAATTCCATATGAAAGAAGGCACAGCAAGACCCACACGTCCGTTCGAGCTCTGACTGCAGACCTACCAAAACAG |
| hsa_circ_0001163 | ATCGAAGCAAAGCTGAGATGGATTTGAAGGAGCTGAGCGAGTCGGTCCAGCAACAGTCCACCCCTGTTCCTCTCATCTCTCCCAAGCGCCAGATTCGTAGCAGGTTCCAGCTGAATCTTGACAAGACCATAGAGAGTTGCAAAGCACAATTAGGCATAAATGAAATCTCGGAAGATGTCTATACGGCCGTAGAGCACAGCGATTCGGAGGATTCTGAGAAGTCAGATAGTAGCGATAGTGAGTATATCAGTGATGATGAGCAGAAGTCTAAGAACGAGCCAGAAGACACAGAGGACAAAGAAGGTTGTCAGATGGACAAAGAGCCATCTGCTGTTAAAAAAAAGCCCAAGCCTACAAACCCAGTGGAGATTAAAGAGGAGCTGAAAAGCACGTCACCAGCCAGCGAGAAGGCAGACCCTGGAGCAGTCAAGGACAAGGCCAGCCCTGAGCCTGAGAAGGACTTTTCCGAAAAGGCAAAACCTTCACCTCACCCCATAAAGGATAAACTGAAGGGAAAAGATGAGACGGATTCCCCAACAGTCCATTTGGGCCTGGACTCTGATTCAGAGAGCGAACTTGTCATAGATTTAGGAGAAGACCATTCTGGGCGGGAGGGTCGAAAAAATAAGAAGGAACCCAAAGAACCATCTCCCAAACAGGATG |
| hsa_circ_0001206 | GTATCCAAGCCCACCAATGGGATCTGTCTCAGCACCCAACCTGCCTACAGCAGAAGATAACCTGGAATATGTACGGACTCTGTATGATTTTCCTGGGAATGATGCCGAAGACCTGCCCTTTAAAAAGGGTGAGATCCTAGTGATAATAGAGAAGCCTGAAGAACAGTGGTGGAGTGCCCGGAACAAGGATGGCCGGGTTGGGATGATTCCTGTCCCTTATGTCGAAAAGCTTGTGAGATCCTCACCACACGGAAAGCATGGAAATAGGAATTCCAACAGTTATGGGATCCCAGAACCTGCTCATGCATACGCTCAACCTCAGACCACAACTCCTCTACCTGCAGTTTCCGGTTCTCCTGGGGCAGCAATCACCCCTTTGCCATCCACACAGAATGGACCTGTCTTTGCGAAAGCAATCCAGAAAAGAGTACCCTGTGCTTATGACAAGACTGCCTTGGCATTAGAG |
| hsa_circ_0001211 | CTCTGCTCTACAAGCCTGTGGACCGTGTGACGAGGAGCACGCTGGTCCTCCATGACTTGCTGAAGCACACTCCTGCCAGCCACCCTGACCACCCCTTGCTGCAGGACGCCCTCCGCATCTCACAGAACTTCCTGTCCAGCATCAATGAGGAGATCACACCCCGACGGCAGTCCATGACGGTGAAGAAGGGAGAG |
| hsa_circ_0001263 | GTGCTAGTGCTTGGAAACAAGAGAGATCTTCCTAATGCCTTGGATGAGAAACAGCTAATTGAAAAAATGAATCTGTCTGCTATTCAGGATAGAGAAATTTGCTGCTATTCAATTTCTTGCAAAGAAAAGGATAATATAG |
| hsa_circ_0001360 | ATCAACCTCTCCACTTCTCCTACACCTGCACAGTTAATAAGCCGTTCCCAGGCTTCCAGTTCTACCAGCGGCAGTATTACCCAACAGACTATGTTACTAGGGAGTACTTCCCCTACCCTAACGGCAAGCCAAGCTCAAATGTATCTCCGAGCTCAAATGCTGATTTTCACACCCGCTACCACTGTGGCTGCTGTACAGTCTGACATTCCTGTTGTCTCGTCGTCATCGTCATCTTCCTGTCAGTCTGCAGCTACTCAG |
| hsa_circ_0001400 | CGAATGCTGATGTCTTAAAGGCGATGGTAGCAGATAACAGCCTGTATGATCCTGAAAGCCCCGTGACCCCCAGCACACCAGGGAGCCCGCCAGTGAGTCCTGGGCCTTTGTCACCAGGGGGGACGCCAGGGAAGCACGTCTGTGGCCATCATCTGCATACGGTGGGCGGTGTTGTCGAGAGGGATGTGTGTCATCGGTGTAGGCACAAGCGGTGGCACTTTATAAAGCCCACTAACAAGTCCAGAGAGAGCAGACCACGGCGCCAAGGCGAGGTCACGGTCCTTTCTGTTGGCAGATTTAGAGTTACAAAAGTGGAGCACAAGTCAAACCAGAAGGAACGGAGAAGCCTGATGTCTGTTAGTGGGGCTGAAACCGTCAATGGGGAGGTGCCGGCAACACCTGTGAAGAGAGAACGCAGTGGCACAGAGTAGCAG |
| hsa_circ_0001411 | AAAGTGACTGAGACCGAAGATGATAGTGATAGTGACAGCGATGATGATGAAGATGATGTTCATGTCACTATAGGAGACATTAAAACGGGAGCACCACAGTATGGGAGTTATGGTACAGCACCTGTAAATCTTAACATCAAGACAGGGGGAAGAGTTTATGGAACTACAG |
| hsa_circ_0001436 | GTGTTGACAGCTCTTCTACCACAAGCAGTGCTTCTCCAATGCCCAACAGTTATGATGCCCTGGAAGGAGGCAGTTACCCAGATATGCTTTCTTCATCAGCAAGCAGTCCTGCTCCTGATCCCGCCCCTGAACCTGATCCTGCTTCTGCTCCAGCTCCAGCTTCAGCTCCAGCTCCTGTCGTCCCTCAGCCTTCAAAAATGGCTAAGCCTTTTGGCTATGGCTATCCAACACTTCAGCCTGGTTATCAGAATGCTACAGCACCACTTATTTCTGGAGTACAGCCCAGTAACCCGGTATATTCTGGATTCCAGCAGTATCCTCAA |
| hsa_circ_0001546 | CTGATGTTCTAATCATGTCAGATAAAGATGATATTGAGACTCCACTGCTAACTGAAGCAGCCCCCATCCTTGAAGATGGAAACTGTGAGCCAGCCAAGAATTCTGAGTCTGTTGACCAAGGTGCCAAACCAGAGAGTAAATCAGAACCTGTAGTTTCCACTCGGAAAAGACCAGAGACCAAACCTTCCAGTGACCTTGAGACTTCAAAAGTTCTCCCTATTCAGGATAATGTTTCCAAAGATGTACCCCAGACCAGATGGGGTTATTGGGGGAGCTGGGGCAAGTCCATACTCTCCTCAGCCTCGGCTACAGTAGCTACAGTAGGACAAGGCATTTCAAATGTCATCGAGAAGGCAGAGACTTCCCTTGGAATCCCTGGTCCCAGTGAAATTTCAACTGAAGTCAAGTATGTAGCAG |
| hsa_circ_0001573 | GTTGCTCCGACTGTGTGTTCCAGGAGTGGTGGCTCTGAGGTGTGACCCTGCCCACGTTTGGGCCCAGCCAGGTTCAGCCCCCCAATAAGGAGGGCAGCTTGATAACACAAAGAAAACAGTGTCAACGAGTACTACCAAGAGAAGAAGACAAGAGGTCAACACAGACTCATTTTCTACTCCGTGTGAATGATAGCTACAGCAGGGGAAAGTTTCATAGTCTATCAGTGGGTCAGAAAATGGAGTTTTATAGCAGAGGCTTCTTAGAAGCTTAAACCCCTGTCCCAATGACGTCAAGTTCGCCCGCTGGCTTGGAAGGTTCAGACCTATCTTCCATCAACACCATGATGTCGGCGGTCATGAGTGTAGGGAAGGTCACAGAGAATGGCGGGAGCCCCCAGGGGATCAAGTCCCCCTCGAAGCCTCCAGGACCAAATCGGATTGGCAGAAGGAACCAGGAAACGAAAGAGGAGAAGTCTTCCTATAACTGCCCCCTGTGTGAGAAGATTTGCACTACCCAGCACCAGCTGACCATGCACATTCGCCAGCACAACACAGACACTGGAGGAGCCGACCACTCATGCAGCATCTGCGGAAAGTCACTGAGCTCGGCCAGCTCCCTCGATCGCCACATGCTGGTGCACTCTGGCGAGAGGCCTTACAAGTGCACTGTGTGTGGCCAGTCATTTACCACCAATGGGAACATGCACAG |
| hsa_circ_0001610 | CTTGGATTCCTTAGCACCACCACAGCTCAGCCAGAACAGAAGGCCTCGAATCTCATTGGCACATACCGCCATGTTGACCGTGCCACCGGCCAGGTGCTAACCTGTGACAAGTGTCCAGCAGGAACCTATGTCTCTGAGCATTGTACCAACACAAGCCTGCGCGTCTGCAGCAGTTGCCCTGTGGGGACCTTTACCAGGCATGAGAATGGCATAGAGAAATGCCATGACTGTAGTCAGCCATGCCCATGGCCAATGATTGAGAAATTACCTTGTGCTGCCTTGACTGACCGAGAATGCACTTGCCCACCTGGCATGTTCCAGTCTAACGCTACCTGTGCCCCCCATACGGTGTGTCCTGTGGGTTGGGGTGTGCGGAAGAAAGGGACAGAGACTGAGGATGTGCGGTGTAAGCAGTGTGCTCGGGGTACCTTCTCAGATGTGCCTTCTAGTGTGATGAAATGCAAAGCATACACAGACTGTCTGAGTCAGAACCTGGTGGTGATCAAGCCGGGGACCAAGGAGACAGACAACGTCTGTGGCACACTCCCGTCCTTCTCCAGCTCCACCTCACCTTCCCCTGGCACAGCCATCTTTCCACGCCCTGAGCACATGGAAACCCATGAAGTCCCTTCCTCCACTTATGTTCCCAAAGGCATGAACTCAACAGAATCCAACTCTTCTGCCTCTGTTAGACCAAAGGTACTGAGTAGCATCCAGGAAGGGACAGTCCCTGACAACACAAGCTCAGCAAGGGGGAAGGAAGACGTGAACAAGACCCTCCCAAACCTTCAGGTAGTCAACCACCAGCAAGGCCCCCACCACAGACACATCCTGAAGCTGCTGCCGTCCATGGAGGCCACTGGGGGCGAGAAGTCCAGCACGCCCATCAAGGGCCCCAAGAGGGGACATCCTAGACAGAACCTACACAAGCATTTTGACATCAATGAGCATTTGCCCTGGATGATTGTGCTTTTCCTGCTGCTGGTGCTTGTGGTGATTGTGGTGTGCAGTATCCGGAAAAGCTCGAGGACTCTGAAAAAGGGGCCCCGGCAGGATCCCAGTGCCATTGTGGAAAAGGCAGGGCTGAAGAAATCCATGACTCCAACCCAGAACCGGGAGAAATGGATCTACTACTGCAATGGCCATG |
| hsa_circ_0001674 | GAACGATTTGTAACCCTGGACCGCGGAAGTCTATGTCTAAGCTGCTTTACATCCGCCTGGCGCTGTTTTTTCCAGAGATGGTCTGGGCCTCTCTGGGGGCTGCCTGGGTGGCAGATGGTGTTCAGTGCGACAGGACAGTTGTAAACGGCATCATCGCAACCGTCGTGGTCAGTTGGATCATCATCGCTGCCACAGTGGTTTCCATTATCATTGTCTTTGACCCTCTTGGGGGGAAAATGGCTCCATATTCCTCTGCCGGCCCCAGCCACCTGGATAGTCATGATTCAAGCCAGTTACTTAATGGCCTCAAGACAGCAGCTACAAGCGTGTGGGAAACCAGAATCAAGCTCTTGTGCTGTTGCATTGGGAAAGACGACCATACTCGGGTTGCTTTTTCGAGTACGGCAGAGCTTTTCTCAACCTACTTTTCAGACACAGATCTGGTGCCCAGCGACATTGCGGCGGGCCTCGCCCTGCTTCATCAGCAACAGGACAATATCAGGAACAACCAAGAGCCTGCCCAGGTGGTCTGCCATGCCCCAGGGAGCTCCCAG |
| hsa_circ_0001709 | TCCACCTCTGGAGGTATGAAGGAGGCTATCCAGCCCTCACAGAAGTCATGAATAAACTCAGAGAAAATAAGGAATTTTTGGAATTTCGTAAGGCAAGAAGTGACATGCTTCTCTCCAGGAAGAATCAGCTCCTGTTGGAGTTCAGTTTCTGGAATGAGCCTGTGCCAAGATCCGGACCTAATATATATGAACTCAGGTCTTACCAACTCCGA |
| hsa_circ_0001810 | GCAAGATCACGTCTCTGGATACCAGCACCATGAGAGCAGCCATGAAACCAGGCTGGGAGGACCTGGTAAGAAGGTGTATTCAGAAGTTCCATGCGCAGCATGAAGGAGAATCTGTGTCCTATGCTAAGAGGCATCATCATGAAGTACTGAGACAAGGATTGGCATTCAGTCAAATCTATCGTTTTTCCTTGTCTGATGGCACTCTTGTTGCTGCACAAACGAAGAGCAAACTCATCCGTTCTCAGACTACTAATGAACCTCAACTTGTAATATCTTTACATATGCTTCACAG |
| hsa_circ_0001846 | CTTTCTGAAACAAAGGTCTTCACTGCCTCATCTGCTCCAGCAGAGAATCACATCTTACCTGGGCAAAGCATTGATCTGGTAGCCTTGCTCCAGAAGCCTGTTCCTCACAGTCAAGCCTCAGAAGCCAACTCCTTTGAAACTTCCCAACAGCAGGGCTTTGGCCAAGCCCTTGTCTTCACAAATTCGCAACACAACAATCAGATGGCACCAGGGACTGGCAGCTCCACTGCCGTCAACTCCTGTTCTCCTCAGAGCCTGTCATCCGTCCTTGGCTCAGGATTTGGAGAGCTTGCACCACCAAAAATGGCAAACATCACCAGCTCCCAGATTTTGGACCAGTTGAAAGCTCCGAGTTTGGGCCAGTTTACCACCACCCCAAGTACACAGCAGAATAGTACAAGTCACCCTACAACTACTACTTCTTGGGACCTCAAGCCCCCAACATCCCAGTCCTCAGTCCTCAGTCATCTTGACTTCAAATCTCAACCTGAGCCATCCCCAGTTCTTAGCCAGTTGAGCCAGCGACAACAGCACCAGAGCCAGGCAGTCACTGTTCCTCCTCCTGGTTTGGAGTCCTTTCCTTCCCAGGCAAAACTTCGAGAATCAACACCTGGAGACAGTCCCTCCACTGTGAACAAGCTTTTGCAGCTTCCCAGCACGACCATTGAAAATATCTCTGTGTCTGTCCACCAGCCACAGCCCAAACACATCAAACTTGCTAAGCGGCGGATACCCCCAGCTTCTAAG |
| hsa_circ_0001849 | AACTGGCATCAAATACTCACAACATAGCTCAGGATCTGTCAAACAAAAGTTCTTATGGACTCAAAGGGGCTTGGAAGAATTCTGTGGAAGAGTGGACAACAGAAGACTGGACTGAAGAT |
| hsa_circ_0001851 | GATTTGGACGTGGCAGAGGGAGAGGGGCAGGAAGGTTCTCAACCCAAGGCATGGGGACATTTAATCCTGCAGACTATTCAGATTCTACATCTACAGATGTGTGTGGGACAAAGCTAGTAGTTTGGGAAGCTGCTCAGAATGGTGCAGATGAGGGAACTG |
| hsa_circ_0001900 | ATAACATCCCTGAGGACCTCAGAGACCCTTTCTACGTTGACCAGTATGAGCAGGAGCACATTAAGCCGCCTGTTATCAAGCTTCTCCTGTCCAGCGAGCTGTACTGCCGTGTCTGCAGCCTCATCCTGAAAGGGGACCAGGTGGCCGCCTTACAGGGACACCAGTCTGTCATCCAGGCCCTGTCCCGGAAAGGGATCTATGTGATGGAGAGTGATGACACCCCCGTGACAGAGTCCGACCTCAGTCGCGCACCCATAAAAATGAGTGCCCACATGGCAATGGTGGATGCCCTGATGATGGCCTACACTGTGGAGATGATCAGCATCGAGAAGGTGGTGGCCAGTGTCAAGCGCTTCTCAACGTTCAGTGCCTCGAAAGAACTTCCGTACGACCTCGAGGATGCCATGGTGTTCTGGATCAACAAG |
| hsa_circ_0002052 | AGCCTACTTGGATGTTAATGAGCTGAAGAACATTCTTAAATTGGATGGATCAACACATCTCAATATTTTCTTTGCAAAATCCTCAGAGGAGGAGTTGGCAGGAGTAGCAACTTGGCCATGGGACAAGGAGGCCCTGATGCACTTAGGTGGCATTGTCTTGAACCCATCTTTCTATGGCATGCCTGGGCACACCCACACCATGATCCATGAGATTGGTCACAGCCTGGGCCTCTATCACGTCTTCCGAGGCATCTCAGAAATCCAGTCCTGCAGTGACCCCTGCATGGAGACAGAGCCCTCCTTCGAGACTGGAGACCTCTGCAATGATACCAACCCAGCCCCTAAACACAAGTCCTGTGGTGACCCAGGGCCAGGAAATGACACCTGTGGCTTTCATAGCTTCTTCAACACTCCTTACAACAACTTCATGAGCTATGCAGATGACGACTGTACGGACTCCTTCACGCCCAATCAAGTCGCCAGAATGCACTGTTACCTGGACCTGGTCTACCAGGGCTGGCAGCCCTCCAGGAAACCAGCGCCTGTTGCCCTCGCCCCCCAAGTTCTGGGCCACACAACGGACTCTGTGACACTGGAGTGGTTCCCACCTATAGATGGCCATTTCTTTGAAAGAGAATTGGGATCAGCATGTCATCTTTGCCTGGAAGGGAGAATCCTGGTGCAGTATGCTTCCAACGCTTCCTCCCCAATGCCCTGCAGCCCATCAGGACACTGGAGCCCTCGTGAAGCAGAAGGTCATCCTGATGTTGAACAGCCCTGTAAGTCCAGTGTCCGCACCTGGAGCCCAAATTCAGCTGTCAACCCACACACGGTTCCTCCAGCCTGCCCTGAGCCTCAAGGCTGCTACCTCGAGCTGGAGTTCCTCTACCCCTTGGTCCCTGAGTCTCTGACCATTTGGGTGACCTTTGTCTCCACTGACTGGGACTCTAGTGGAGCTGTCAATGACATCAAACTGTTGGCTGTCAGTGGGAAGAACATCTCCCTGGGTCCTCAGAATGTCTTCTGTGATGTCCCACTGACCATCAGACTCTGGGACGTGGGCGAGGAGGTGTATGGCATCCAAATCTACACGCTGGATGAGCACCTGGAGATCGATGCTGCCATGTTGACCTCCACTGCAGACACCCCACTCTGTCTACAGTGTAAGCCCCTGAAGTATAAGGTGGTCCGGGACCCTCCTCTCCAGATGGATGTGGCCTCCATCCTACATCTCAATAGGAAATTCGTAGACAT |
| hsa_circ_0002254 | GGCAGCGGTCTTAATTCTTTTTATGATCAACGAGAATACATAGGGAGAAGTGTTCATTATTGGAAGAAAGTTTTGCCATTGTTGAAGATAATAAAAAAGAAGAACAGTATTCCTGAACCTATTGATCCTCTGTTTAAACATTTTCATAGTGTAGACATTCAGGCATCAGAAATTGTTGAATATGAAGAAGACGCACACATAACTTTTGCTATATTGGATGCAGTAAATGGAAATATAGAAGATGCTGTGACTGCTTTTGAATCTATAAAAAGTGTTGTTTCTTATTGGAATCTTGCACTG |
| hsa_circ_0002257 | AGTTTGGCTGAATTGGAAGTGTTATGTACTCATCTCTACATAGGGACTGATCTTACACAAAGAATAGAGGCTGAGAAAGCACTCTTGGAACTTATTGACAGTCCAGAATGTCTCAGCAAGTGTCAACTTTTATTAGAACAAGGAACAACATCCTATGCTCAGCTCCTTGCAGCAACATGTCTTTCAAAACTTGTCAGCCGAGTCAGTCCTTTACCTGTTGAGCAGAGGATGGACATCA |
| hsa_circ_0002346 | ATGAGAACTGGACTGATGACCAACTGCTTGGTTTTAAACCATGCAATGAAAACCTTATTGCTGGCTGCAATATAATCAATGGGAAATGTGAATGTAACACCATTCGAACCTGCAGCAATCCCTTTGAGTTTCCAAGTCAGGATATGTGCCTTTCAGCTTTAAAGAGAATTGAAGAAGAGAAGCCAGATTGCTCCAAGGCCCGCTGTGAAGTCCAGTTCTCTCCACGTTGTCCTGAAGATTCTGTTCTGATCGAGGGTTATGCTCCTCCTGGGGAGTGCTGTCCCTTACCCAGCCGCTGCGTGTGCAACCCCGCAGGCTGTCTGCGCAAAGTCTGCCAGCCGGGAAACCTGAACATACTAGTGTCAAAAGCCTCAGGGAAGCCGGGAGAGTGCTGTGACCTCTATGAGTGCAAACCAGTTTTCGGCGTGGACTGCAGGACTGTGGAATGCCCTCCTGTTCAGCAGACCGCGTGTCCCCCGGACAGCTATGAAACTCAAGTCAGACTAACTGCAGATGGTTGCTGTACTTTGCCAACAAG |
| hsa_circ_0002534 | GACTCAGTGGCCTTTGAAGATGTGGCTGTGAACTTCACACAAGAGGAGTGGGCTTTGCTGGGTCCATCACAGAAGAGTCTCTACAGAAATGTCATGCAGGAAACCATTAGGAACCTGGACTGTATAGAAATGAAATGGGAGGACCAGAACATTGGAGATCAGTGCCAAAATGCCAAGAGAAATCTAAG |
| hsa_circ_0002600 | GCTGGACCGCTTTGAGAAGACCAATGAGATGCTGCTCAACTTCAACAACCTGTCCAGTGCCCGCCTGCAGCAGATGAGCGAACGCTTCCTGCACCACACGAGGACCCTAGTAGAGATGAAACGGGACCTGGACAGCATCTTCCGCCGTATCAGGTGGGTGCTCAGTGCCACCCCAGCCCAGCTGACCTCTGCCTCCACCTCCCCATCTGAGCCTCAGTTTCCTTCACATACCAGGGTAAAGGGAGATTTCTGTTTGTTTTACAAGGCTTCACTGCGCACTTAAGTGTCTGGGACAATTCCAACCCTGGGATAAAATCTTGAC |
| hsa_circ_0002649 | AATGGGAGTGAGGCCCTAATGATTCTAGATGACCAAACTAACAAACTGAAATCGGGTGTTGACCAGATGGATTTGTTTGGGGACATGTCTACACCTCCTGACCTAAATAGTCCAACAGAAAGCAAAGATATCCTGTTAGTGGATCTAAACTCTGAAATCGACACCAATCAGAATTCTTTAAGAGAAAATCCATTCTTAACAAACGGCATCACCTCCTGTTCTCTTCCTCGACCAACGCCTCAGGCATCCTTCTTGCCTGAAAATGCCTTTTCTGCCAATCTCAACTTCTTTCCCACCCCTAATCCTGATCCTTTCCGTGACGATCCTTTCACACAGCCAGACCAATCGACACCTTCTTCGTTTGATTCTCTCAAATCTCCAGATCAGAAGAAAGAGAATTCGAGTAGCTCGTCTACTCCGCTGAGTAATGGGCCCCTGAATGGTGATGTTGACTACTTTGGTCAGCAATTTGACCAGATCTCTAACCGGACTGGCAAACAGGAAGCTCAGGCAGGCCCATGGCCCTTTTCAAGTTCGCAAACCCAGCCAGCAGTGAGAACTCAAAATGGGGTATCTGAAAGAGAACAGAACGGCTTCTCTGTCAAATCCTCCCCGAACCCTTTTGTGGGAAGCCCTCCCAAAGGACTGTCCATACAGAATGGCGTAAAGCAGGACTTGGAAAGCTCTGTCCAGTCCTCACCACATGACTCCATAGCCATTATCCCACCTCCACAAAGTACCAAACCAGGAAGAGGCAGAAGGACTGCTAAG |
| hsa_circ_0002703 | CTCATCAGTGGGGCCACGAGCTGAGTGCGTCCTGTCACTCCACTCCCATGTCCCTTGGGAAGGTCTGAGACTAGGGCCAGAGGCGGCCCTAACAGGGCTCTCCCTGAGCTTCGGGGAG |
| hsa_circ_0002706 | GGCAGCGGTCTTAATTCTTTTTATGGTCAACTAGAATACATAGGGAGAAGTGTTCATTATTGGAAGAAAGTTTTGCCATTGTTGAAGATAATAAAGAAGAACAGTATTCCTGAACCTATTGATCCTCTGTTTAAACATTTTCATAGTGTAGACATTCAGGCATCAGAAATTGTTGAATATGAAGAAGACGCACACATAACTTTTGCTATATTGGATGCAGTAAATGGAAATATAGAAGATGCTGTGACTGCTTTTGAATCTATAAAAAGTGTTGTTTCTTATTGGAATCTTGCACTG |
| hsa_circ_0002780 | ATCGCCTTTGTTTTGCCATTCTCTACAGCAGACCAAAGAGTGCATCAAATGTACATTATTTCAGCATAGATAATGAACTTGAATATGAGAACTTCTACGCAGATTTTGGACCACTCAATCTGGCAATGGTTTACAGATATTGTTGCAAGATCAATAAGAAATTAAAGTCCATTACAATGTTAAGGAAGAAAATTGTTCATTTTACTGGCTCTGATCAGAGAAAACAAGCAAATGCTGCCTTCCTTGTTGGATGCTACATG |
| hsa_circ_0002810 | GGGGCTTGGAGTACCCATAATACAGTGAGCCCACCTTCCTGGTCCCCAGACATTTCAGAAGGTCGGGAAATTTTTAAACCCAGGCAGCTTCCTGGCAGTGCCATTTGGAGCATCAAAGTGGGCCGTGGGTCTGGATTTCCAGGAAAGCGGAGACCTCGAGGTGCAGGACTGTCGGGGCGAGGTGGCCGAGGCAGGTCAAAGCTGAAAAGTGGAATCGGAGCTGTTGTATTACCTGGGGTGTCTACTGCAGATATTTCATCAAATAAGGATGATGAAGAAAACTCTATGCACAATACAGTTGTGTTGTTTTCTAGCAGTGACAAGTTCACTTTGAATCAGGATATGTGTGTAGTTTGTGGCAGTTTTGGCCAAGGAGCAGAAGGAAGATTACTTGCCTGTTCTCAGTGTGGTCAGTGTTACCATCCATACTGTGTCAGTATTAAGATCACTAAAGTGGTTCTTAGCAAAGGTTGGAGGTGTCTTGAGTGCACTGTGTGTGAGGCCTGTGGGAAGGCAACTGACCCAGGAAGACTCCTGCTGTGTGATGACTGTGACATAAGTTATCACACCTACTGCCTAGACCCTCCATTGCAGACAGTTCCCAAAGGAGGCTGGAAGTGCAAATGGTGTGTTTGGTGCAGACACTGTGGAGCAACATCTGCAGGTCTAAGATGTGAATGGCAGAACAATTACACACAGTGCGCTCCTTGTGCAAGCTTATCTTCCTGTCCAGTCTGCTATCGAAACTATAGAGAAGAAGATCTTATTCTGCAATGTAGACAATGTGATAGATGGATGCATGCAGTTTGTCAGAACTTAAATACTGAGGAAGAAGTGGAAAATGTAGCAGACATTGGTTTTGATTGTAGCATGTGCAGACCCTATATGCCTGCGTCTAATGTGCCTTCCTCAGACTGCTGTGAATCTTCACTTGTAGCACAAATTGTCACAAAAGTAAAAGAGCTAGACCCACCCAAGACTTATACCCAGGATGGTGTGTGTTTGACTGAATCAGGGATGACTCAGTTACAGAGCCTCACAGTTACAGTTCCAAGAAGAAAACGGTCAAAACCAAAATTGAAATTGAAGATTATAAATCAGAATAGCGTGGCCGTCCTTCAGACCCCTCCAGACATCCAATCAGAGCATTCAAGGGATGGTGAAATGGATGATAGTCGAG |
| hsa_circ_0003028 | CATGTAGAGCGCATGAAGTACAGGACAATAAAGCTTCCTACACATATCACCAGGAGGATCTCTTTGAAAGATTCACTGCAGGACTACCAGAGAGAATAATTTGTCTGAAGCATCATGTGTTGAAACAACAGAAGTCTATTCACCTGTGCACTAACTAGAAACAGAGTTACAATGTTTTCAATTCTTTGAGCTCCAGGACTCCAGGGAAGTGAGTTGAAAATCTGAAAATGCGGCCATGGACTGGTTCCTGGCGTTGGATTATGCTCATTCTTTTTGCCTGGGGGACCTTGCTGTTTTATATAGGTGGTCACTTGGTACGAGATAATGACCATCCTGATCACTCTAGCCGAGAACTGTCCAAGATTCTGGCAAAGCTTGAACGCTTAAAACAACAGAATGAAGACTTGAGGCGAATGGCCGAATCTCTCCG |
| hsa_circ_0003034 | GGCAGGGGTCTTAATTCTTCTTATGATCAACAAGAATACATAGGGAGAAGTGTTCATTATTGGAAGAAAGTTTTGCCATTGTTGAAGATAATAAAGAAGAACAGTATTCCTGAACCTATTGATCCTCTGTTTAAACATTTTCATAGTGTAGACATTCAGGCATCAGAAATTGTTGAGTATGAAGAAGATGCACACATAACTTTTGCTATATTGGATGCAGTACATGGAAATATAGAAGATGCTGTGACTGCTTTTGAATCTATAAAAAGTGTTGTTTCTTATTGGAATCTTGCACTG |
| hsa_circ_0003053 | GCTGGAGCATTGGATTTGCTAAAGGAGCTTAAGAATATTCCTATGACCCTGGAATTACTGCAGTCCACAAGAATCGGAATGTCAGTTAATGCTATTCGCAAGCAGAGTACAGATGAGGAAGTTACATCTTTGGCAAAGTCTCTCATCAAATCCTGGAAAAAATTATTAG |
| hsa_circ_0003076 | ATGACGACTGTACGGACTCCTTCACGCCCAATCAAGTCGCCAGAATGCACTGTTACCTGGACCTGGTCTACCAGGGCTGGCAGCCCTCCAGGAAACCAGCGCCTGTTGCCCTCGCCCCCCAAGTTCTGGGCCACACAACGGACTCTGTGACACTGGAGTGGTTCCCACCTATAGATGGCCATTTCTTTGAAAGAGAATTGGGATCAGCATGTCATCTTTGCCTGGAAGGGAGAATCCTGGTGCAGTATGCTTCCAACGCTTCCTCCCCAATGCCCTGCAGCCCATCAGGACACTGGAGCCCTCGTGAAGCAGAAGGTCATCCTGATGTTGAACAGCCCTGTAAGTCCAGTGTCCGCACCTGGAGCCCAAATTCAGCTGTCAACCCACACACGGTTCCTCCAGCCTGCCCTGAGCCTCAAGGCTGCTACCTCGAGCTGGAGTTCCTCTACCCCTTGGTCCCTGAGTCTCTGACCATTTGGGTGACCTTTGTCTCCACTGACTGGGACTCTAGTGGAGCTGTCAATGACATCAAACTGTTGGCTGTCAGTGGGAAGAACATCTCCCTGGGTCCTCAGAATGTCTTCTGTGATGTCCCACTGACCATCAGACTCTGGGACGTGGGCGAGGAGGTGTATGGCATCCAAATCTACACGCTGGATGAGCACCTGGAGATCGATGCTGCCATGTTGACCTCCACTGCAGACACCCCACTCTGTCTACAGTGTAAGCCCCTGAAGTATAAGGTGGTCCGGGACCCTCCTCTCCAGATGGATGTGGCCTCCATCCTACATCTCAATAGGAAATTCGTAGACAT |
| hsa_circ_0003123 | TCGATGCTCCGAGGGAGAGGAGCTTGTTACAAACACACATTCTATGGAATTGAGAGCCATCGCTGCATGGAAACCACCCCGAGCTTGGCGTGTGCTAATAAATGTGTCTTCTGTTGGTGGCACCACAACAACCCTGTGGGCACTGAATGGCGGTAGAAGATGGACCAGCCTGAAATGATCTTGAAGGAAGCCATTGAAAACCATCAGAACATGATTAAGCAGTTTAAAG |
| hsa_circ_0003147 | GAGCTCAACAGGACCTGTCGGGCCATGCAGCAGCGCATCGTGGAGCTCATCTCCCGCGTGTCCAATGAGGAGGTCACCGAGGAGCTGCTGCATGTGAACGATGACCTCAACAACGTCTTCCTTCGATACGAGAGGTTCGAACGATACAGGTCTGGCCGATCCGTTCAAAATGCCAGTAATGGAGTACTGAATGAAGTAACCGAAGACAACTTAATAGACCTGGGGCCAGGGTCTCCAGCCGTGGTGAGCCCAATGGTGGGGAACACAGCGCCCCCATCTTCCCTCTCCTCCCAGCTTGCAGGCTTAG |
| hsa_circ_0003151 | ACTCCTGCCCCTGCCACGAGAACACACCCTTTCCAGTTTGCTGGAGAAATATAAGAGAAGTCCAGTGAAAGAGAGCTGAGTTTCCCCAATGATGCCATCAAGATGAACTAACTACAGTTAACCAGCCAGCTGATAGCAGATGCATGAGTGAGCCCAGCTGAGAGCAGGTTGTGCAAGCATGGCACGTGCATCTGTTTGGCTTGTGGTGAGGCCTCAGGAAGCTTACAGTCATGGTGGAAGGCAAAGAGGGAGCCAGTACATCACGTGAACTCGTAGAGTGAGAACTCATTCACTACCATGAAGACAACACCAAGCCATTCCTGAG |
| hsa_circ_0003210 | AAGAAGTTAGCGGTATTCAGCAAGATGCAGGACTCTCTGGAAGTCACCCTTCCCAGCAAACAAGAGGAGGAGGATGAGGAGGAGGAGGAGGAGGAGAAAGACCAGCCTGCCGAGATGGAGTACCTTAACTCTCGCTGTGTCCTTTTCACTTATTTCCAGGGAGACATTGGGTCAGTAGTGGATGAACACTTCTCAAGAGCTTTGGGCCAAGCCATCACCCTCCATCCAGAATCTGCCATTTCAAAAAGCAAGATGGGGCTAACCCCCCTATGGCGAGACAGCTCAGCTCTCTCAAGCCAGCGGAATAGTTTCCCAACTTCCTTTTGGACCAGCTCTTACCAGCCCCCACCTGCACCTTGTTTGGGGGGAGTTCATCCTGACTTCCAGGTCACTGGACCCCCTGGCACCTTTTCTGCAGCTGATCCCAGTCCTTGGCCGGGACACAACCTGCATCAGACTGGCCCAGCCCCTCCCCCTGCTGTGTCTGAGTCCTGGCCTTATCCTTTGACATCTCAGGTGAGCCCATCCTACAGCCATATGCATGACGTGTACATGCGGCACCACCACCCTCATGCCCACATGCACCACCGCCACCGCCACCATCATCACCATCACCACCCTCCTGCTGGCTCTGCCCTGGATCCATCCTATGGGCCTCTGCTGATGCCTTCAGTGCATGCGGCCAGGATTCCTGCTCCCCAGTGTGACATCACAAAGACAGAACCAACTACAGTCACCTCTGCTACCTCAGCATGGGCTGGAGCCTTTCATGGAACAGTAGACATAGTGCCCAGCGTGGGATTCGATACAG |
| hsa_circ_0003337 | ATTTCTCGAATGCTGGCAGCCAAAACCGTTTTGGCTATCCGTTATGATGCTTTTGGTGAGGATTCAAGTTCTGCAATGGGAGTTGAGAACAGAGCCAAATTAGAGGCCAGGTTGAGAACTTTGGAAGACAGAGGGATAAGAAAAATAAGTGGAACAGGAAAAGCATTAGCAAAAACAGAAAAATATGAACACAAAAG |
| hsa_circ_0003347 | AACGCTGCGATGACTGGGGACTAGACACCATGAGGCAAATCCAAGTGTTTGAAGATGAGCCAGCTCGCATCAAGTGCCCACTCTTTGAACACTTCTTGAAATTCAACTACAGCACAGCCCATTCAGCTGGCCTTACTCTGATCTGGTATTGGACTAGGCAGGACCGGGACCTTGAGGAGCCAATTAACTTCCGCCTCCCCGAGAACCGCATTAGTAAGGAGAAAGATGTGCTGTGGTTCCGGCCCACTCTCCTCAATGACACTGGCAACTATACCTGCATGTTAAGGAACACTACATATTGCAGCAAAGTTGCATTTCCCTTGGAAGTTGTTCAAAAAGACAGCTGTTTCAATTCCCCCATGAAACTCCCAGTGCATAAACTGTATATAGAATATGGCATTCAGAGGATCACTTGTCCAAATGTAGATGGATATTTTCCTTCCAGTGTCAAACCGACTATCACTTGGTATATG |
| hsa_circ_0003401 | ACTCAGATTGATGGACAACTTTTCTTAATTAAGCACCTTTTGATACTTCGTGAACAAATTGCTCCATTTCACACTGAATTCACCATTAAGGAAATTTCCCTGGACCTCAAGAAAACTAGAGATGCAGCATTTAAAATCCTGAACCCTATGACTGTCCCAAGATTTTTTAGGCTGAATAGCAACAATGCCTTGATAGAGTTCTTGTTGGAGGGTACTCCTGAGATAAGAGAACATTATCTTGACTCTAAAAAAGACGTAGACCGTCATCTGAAATCGGCCTGTGAGCAGTTTATTCAGCAGCAGACCAAGCTGTTTGTAGAACAGCTGGAGGAGTTCATGACAAAG |
| hsa_circ_0003426 | AACTCAACAGTGACAATTGAGGAATTCCACTGTAAGCTCCAAGAAGCCACAAACTTTCCCCTTCGTCCTTTTGTGATTCCATTTCTCAAGGCCAACCTGCCCCTGCTGCAGCGGGAACTGCTGCACTGCGCTCGGGCGGCCAAGCAGACCCCATCCCAGTACCTGGCTCAGCACGAACACCTTCTGCTCAACACAAGCATTGCATCGCCTGCTGACTCGTCAGAGTTGCTCATGGAGGTGCACGGAAATGGGAAGAGGCCCAGTCCAGAGAG |
| hsa_circ_0003432 | GTGCTGCAGAGCTTCAAGATAATGGATTACAGCCTCTTGATGTCAATCCATAATATAGATCATGCACAACGAGAGCCCTTAAGCAGTGAAACACAGTACTCAGTTGATACTCGAAGACCGGCCCCCCAAAAGGCTCTGTATTCCACAGCCATGGAATCCATCCAGGGAGAGGCTCGACGGGGTGGTACCATGGAGACTGATGACCA |
| hsa_circ_0003436 | TGCTGGACCCAAAGGAGACAACATTTATGAATGGAGGTCAACTATATTGGGACCCCCAGGATCTGTCTATGAAGGAGGGGTGTTCTTTCTTGACATTACCTTTTCACCAGACTATCCGTTTAAACCCCCTAAGGTTACCTTCCGAACAAGAATCTATCACTGTAATATTAACAGCCAAGGTGTGATCTGTCTGGACATCTTAAAGGACAACTGGAGTCCGGCTTTAACTATTTCTAAAGTTCTCCTCTCCATCTGCTCACTTCTTACAGATTGCAACCCTG |
| hsa_circ_0003451 | GTTCTTTGCAGATGTAGTTCCAGCAGTCAGGAAGTGGAGAGAGGCCGGAATGAAGGTGTACATCTATTCCTCAGGGAGTGTGGAGGCACAGAAACTGTTATTCGGGCATTCTACGGAGGGAGATATTCTTGAGCTTGTTGATGGTCACTTTGATACCAAGATTGGACACAAAGTAGAGAGTGAAAGTTACCGAAAGATTGCAGACAGCATTGGGTGCTCAACCAACAACATTTTGTTTCTGACAGATGTTACTCGAG |
| hsa_circ_0003458 | CGGGGATCTGATGAGCTTCTTTCTTCTGGCATCATTAACGGACCTTTTACCATGAATAGTTCTACTCCTTCTACAGGTGTGTATGCTAATGGGAATGACAGCAAGAAATTTAAACGAGATAGACCTCCCTGTTCGCCTTCCCGTGTTCTCCATCTTCGAAAAATTCCATGTGATGTCACCGAAGCAGAGATCATATCATTAGGTCTACCATTTGGCAAAGTAACTAATCTTTTGATGTTGAAAGGAAAAAGCCAGGCTTTCTTAGAAATGGCTTCTGAGGAAGCTGCCGTTACTATGGTGAATTATTACACTCCTATTACTCCTCACCTTCGAAGCCAGCCTGTTTATATTCAGTATTCCAATCACAGAGAACTTAAGACTGACAATCTACCTAATCAAGCTCGAGCCCAAGCTGCACTGCAGGCTGTCAGTGCCGTCCAATCAGGAAGCCTGGCCCTTTCTGGAGGTCCTTCCAATGAAGGCACAGTCCTACCTGGGCAGAGCCCTGTGCTTCGAATAATTATTGAAAACCTCTTTTACCCTGTTACCCTGGAAGTTCTTCATCAGATATTTTCTAAATTTGGCACAGTCTTGAAGATTATCACCTTTACAAAGAATAATCAGTTTCAAGCCTTGCTTCAGTATGCTGACCCAGTAAATGCACATTATGCCAAAATGGCTCTGGATGGCCAGAATATCTATAATGCATGCTGCACTCTGCGCATTGACTTCTCCAAGCTCACCAGCCTTAATGTGAAATATAATAATGACAAAAGCAGAGACTTCACTCGCTTAGACCTTCCTACTGGTGATGGCCAGCCATCCCTTGAACCCCCTATGGCTGCTGCTTTTG |
| hsa_circ_0003478 | GCCTCTCTCTGGAACGCCTACCCAACTCCATCGCCTCCCGCTTCCGCCTGACAGAGAGGGAGGAGGAAGTGATCACCTGTTTTGAGAGGGCCTCCTGGATCGCTCAGGTGTTCCTGCAGGAATTGGAGAAGACCACAAATAACAGCACGTCGAGGCATCTGAAAGGCTGTCACCCGCTTGACTATGAGCTCACCTACTTCCTGGAAGCTGCCCTCCAGAGCGCCTATGTGAAAAACCTGAAGAAGGG |
| hsa_circ_0003496 | CATTGATCTGGTAGCCTTGCTCCAGAAGCCTGTTCCTCACAGTCAAGCCTCAGAAGCCAACTCCTTTGAAACTTCCCAACAGCAGGGCTTTGGCCAAGCCCTTGTCTTCACAAATTCGCAACACAACAATCAGATGGCACCAGGGACTGGCAGCTCCACTGCCGTCAACTCCTGTTCTCCTCAGAGCCTGTCATCCGTCCTTGGCTCAGGATTTGGAGAGCTTGCACCACCAAAAATGGCAAACATCACCAGCTCCCAGATTTTGGACCAGTTGAAAGCTCCGAGTTTGGGCCAGTTTACCACCACCCCAAGTACACAGCAGAATAGTACAAGTCACCCTACAACTACTACTTCTTGGGACCTCAAGCCCCCAACATCCCAGTCCTCAGTCCTCAGTCATCTTG |
| hsa_circ_0003536 | GTATCTCTCTGAATTCCTTTACGCATGGTTGATGTCAACATTGAGTCGTGCCGATGGCTCTCAAATGGCAGAGGAAAGGATAATGGAAGAGCAGCAGAAAGGCCGTAGTAGTAAAAAAACAAAGAAAAAAAAGAAAGTTCGCCCATTGAGCCGAGAGATCACAATGAGCCAAGCATATCAGAACATGTGTGCTGGAATGTTTAAAACCATGGTAGCATTTGACATGGACGGCAAAGTACGTAAACCGAAGTTTGAGCTTGATAGTGAACAAGTTCGGTATGAACACAGGTTTGCTCCATTCAACAGTGTGATGACCCCGCCGCCAGTGCACTACTTACAGTTCAAGGAAATGTCTGACCTCAATAAATATAGCCCTCCTCCTCAGTCTCCTGAACTGTATGTGGCAGCTAGTAAGCACTTTCAACAGGCAAAAATGATATTGGAAAATATTCCTAACCCGGACCATGAG |
| hsa_circ_0003593 | GATGAAGATGAGCTGGATTCCCACACCATGGTGAAGACTAGTGTGGAGAGTGTGGGCACCATGCGGGCCACAAGCACGATGAGTGAAGGGGCCCAGACCATGATTGAACATAATAGCACGATGTTGGAATCCGACTTGGGGACCATGGTGATAAACAGTGAGGATGAGGAAGAAGAAGATGGAACTATGAAAAGAAATGCAACCTCACCACAAGTACAAAGACCATCTTTCATGGACTACTTTGATAAGCAAGACTTCAAGAATAAGAGTCACGAAAACTGTAATCAGAACATGCATGAACCCTTCCCTATGTCCAAAAACGTTTTTCCTGATAACTGGAAAGTTCCTCAAGATGGAGACTTTGACTTT |
| hsa_circ_0003713 | GACTGTGAATTGGAGAATCAAGAGGCACATGAGCAAGATGGAAATGATGAACTAAAGGACTCTGAAGAATTTGGTGAAAATGAAGAAGAAAATGTGCATTCCAAGGAGTTACTCTCTGCAGAAGAAAACAAGAGAGCTCATGAATTAATAGAGGCAGAAGGAATAGAAGATATAGAAAAAGAGGACATCGAAAGTCAGGAAATTGAAGCTCAAGAAGGTGAAGATGATACCTTTCTAACAGCCCAA |
| hsa_circ_0003718 | ATAGATGCTGTGAAATTCATGCTAAAAAACCACACGAGTGAACACTTCCCTTTTCTTGGCATCAGTGACAATCATAGTCTCAGCGACTTCAGGTGTCGAACAACCTTCTACACAGCGCTCACTCGCCTTCTGATGGTAGATCTGGGTGAAGATGAGGATGAATTTGAGAATTTCATGCTGCCTCTTACAGTTGCTTTTGAAACAGTATTACAAATATTCAACAACAACTTTAAACAAGAAGATGTAAAGCGTATGTTGATCGGGCTGGCAAGAGATCTTCGAGGGATTGCCTTTGCACTGAACACAAAGACCAGCTACACCATGCTGTTTGACTGGAT |
| hsa_circ_0003806 | ACAGCTCAGCTCTCTCAAGCCAGCGGAATAGTTTCCCAACTTCCTTTTGGACCAGCTCTTACCAGCCCCCACCTGCACCTTGTTTGGGGGGAGTTCATCCTGACTTCCAGGTCACTGGACCCCCTGGCACCTTTTCTGCAGCTGATCCCAGTCCTTGGCCGGGACACAACCTGCATCAGACTGGCCCAGCCCCTCCCCCTGCTGTGTCTGAGTCCTGGCCTTATCCTTTGACATCTCAGGTGAGCCCATCCTACAGCCATATGCATGACGTGTACATGCGGCACCACCACCCTCATGCCCACATGCACCACCGCCACCGCCACCATCATCACCATCACCACCCTCCTGCTGGCTCTGCCCTGGATCCATCCTATGGGCCTCTGCTGATGCCTTCAGTGCATGCGGCCAGGATTCCTGCTCCCCAGTGTGACATCACAAAGACAGAACCAACTACAGTCACCTCTGCTACCTCAGCATGGGCTGGAGCCTTTCATGGAACAGTAGACATAGTGCCCAGCGTGGGATTCGATACAG |
| hsa_circ_0003812 | ACTCAAATGACTCCAGAAAGCTACACTTCCTGTTGTGAGTATATGATATCCATTTCCCTACATAGCCACTAACATCAGGTTTTTACAATTTTATTTATTTCTTGCTACTTTAAGAAATTTTTGTGGTGAAATACATATAATAGAAGTTGACTATCTGAATCATTTTTAAGTATACATTCAGTAGTGTTAAGTATGTCGCCATTGTTGTACAACCAATCTCCAGAACTTTTTCATCTTGCAAAACAAACTCTGTACCCATTAAATAACATTAAACATTCCATTCCCTCCAGCCTCAGCAACCCCATTCTACTTTCTGTTTCTGTGAGTTTGACTATTCCAAGCACTTCATATCAGTTAAATCATGAAGTATTTGTCTGTCTGTGACTGGCTTATTTCTCTGAGCACAGTGTCCTCGAGATGCGTCTATGTTGTAGCATATGTCAGAATTTCCTTCCTTTTTAAAAGATCCAAATAATATTCTTATTTTATATCTTTTTTTTATCCATTCATCCATTAGTGGACACTTGGGTTGCTTTTGGCTATTGTAAATAATGGTGCTATGTACAAATATCTATATTATTGTATTTACAAGTATAATGCTGTAATGTACACACATCTTTTTGAGATCCTACCTTCAGTTCTTTTGAGTATATAGCCAGAAGTGGTATTACTAAATCTTACGATATTTCTATTTTTAATTTATTGAGGAACCACTGTAGTTTTTCATAGCAACTGCACCATTTTACGTTCTCACCAAGAGTGCACAAGGGTTCCGAGGTTCCCACATCCTCCCCAACACTTGTTATTTTCTGCTTTTTTTAGATTGCAGCCATCATAGTGGGTGTGAGGTGACATTTCATTGTGGTTTTGATTTGCATTTCCCTAATGAGGAGTGATGCTGAGCATCTTTTCATATGCTTACTGGTCATTTGTATGTTGTCTTTGGAAAAATGTCTATTCAAGTCCTTTGACTATTTTAAAAATTGGGTTATTAGAGTTATCGTTGTTGTTGACTTGTAGGAGTTTCTTTCTATATTCTGGATATTAATCCCCTATCAGATATATGATTTGCAAATATCTTCTCTTATTCCATAAGGTTACTTTTTCACTTTGTTGATTGTGTTCTTTGATGTATAGAAGTTTTTAGTTTTGAAATAGTCTAATTTATCTGTTTTTACTTTTGTGGTCTGTGCTTTTGGTGTCATATCCAAGAAATCCTTGCCAAATCCAACGTTATAAGGTACTTTTAAGGTATTTTAGTTGTCTTAGTCTATATTTCTGTACTCACCTTTCTTTATCCACTCATCAGTTGATGGGCATGTAGGTTGGTTCCATATCTTTGCAATTCTGAATTGTGCTATGATCAGGTGTCTTTTTAGTATAATGATTTACTCTCCTTTGGGTAGATACCCAGTAGTGGGATTGCTGGATCGAATGGTTTTTATAATTTTCTATTTTACCACAGTTTCTCTCTGCATTTTTCCTCTTTGACCACTAACCATGTGAAATTCTCATATTGACCTTTATAATGATCATGAACTCTTAGTATCATTGGGAAGGCCACATTTGCCACTTATGATTGTAAACCTTATCCTCCATTTTTCCTGTTATTGTTGGTGCAAAAAGCACCTATTATACCAGGACTTTAAAAATCAGTCTGATAAGTCTTTGATAAGTCTAATAATAATAACTGATAAGTCCATTGAATTTGCTTCTGATTACTTTTTCTTTAGTAGCTAAACATGTATGTACTCCTATGATTACAATGAACACTCCTCTCCATTTAAATTAATTATTTACATTGATGAAATAGCAAAATGTTAATGACTAAATACTGTCTTGGTTTTTTCGTTCCAGGTCAGTCAATATTAACTTCTTATAATTTTCTTTTTTTTCTTTATGTGTGTGTGTGTGTGTATTTTTTTTTTTTTAATTTCAATGGCTTTTGGGGTACAAATGGCTTTTGGTCATATAGATGAATTCTACAGTAGTGAAGTCTGAGATTTTACTGCACCGGTCACCTGAGTAGTGTACATTGTACCCAATATGTGGTTTTTTATACCTTGCCCCCCTCTTACCCTCCCCACTTTGAGTCTCTAGTGTCCATTATGTCACTCTGTATACCTTTTTGTACCCATAAGTTAGCTCTCACTTATAAGTGAGAACACACAGTATTTGGTTTTCCATTCCTGAGTTGCTTCACTTAGAATAATATCCTCCAGCTCCATCCAAAATTGCTGCAAAAAAAAAAAAAACCACAAACATTATTTTGTTCTTTTTTATTGCTAAGTCATATTCCATGGTGTAGAGATACCACATTTTATTTATCCACTCACTGGTTGATGGGTTGGTTCCACATCTTTGCAATTGTGACTTGTACTGCCATCAAGTGTCTTTCTGGTATAATGACTTCTTTTCCTTTGGGTAGATACCCAGGAGTGGGATTGCTAGATCAAATGGTTCTTAACATTTTCTCTCTGGATCTATTTCTGGAAATTTTAGGCTCCAGTTTTTGTTGTTGTTGTTAATAAAATGCAATGGAATGTAATGATCATCACTTTTCATTATGCTTTAAAATCTGGTAAATGGAGGCTAGAACACTCCTGTAAGGCAAGAATATTCTCTCTGTTGGAACTCAAATACACAGAACTGGGTAAATCTCAATCTTAATCTTTGATTCAGGACACAACATGGCTCTCTTTTACTTGCTTTCTTTAATTGTTTTTTAATAATGTGGTAAGCATTTCTGAATCTCCTATCCAATACAAAAACTAGGACAATACAGACAGTAACTCCTATGGTTACAATGAACACTCCTCTCCACTTAAATTAATTATTTACACTGATGAAATTGAAATAGCAAAATTTTAATGACTAAATACTGTCTTTGATTTTTTGTTCCAGGTCTGTCAATATTAACTTCTTATAATTTTCTTTTTTTTTCTTTATGTGTGTGTGTGTGTGTGTATATATATATATTTAATTTCAATGGCTTTTGGGGTACAAATGGCTTTTGGTCATATATATGAGTTCTACAGTAGTGAAGTCTGAGATTTTACTACACCTTCCACTTATGTGGTCCCACACCACCCGCCTCCCCTGCCGCCTCCTGCCACCCCCTAGGCCAAGGTAATAATCATCCTGAATCCTGGGTTTATCTCTCACTTGCTTTCTTTTCATATAATTTTGCAAAAGAATCTGATCTAAATGTGTTTTTCAGAGTATATATTTATATTTTAGCTGTTCTTAGAGAAAATTTATTATTTTGCATGTAATCTTATGGAACATTCTCATTTAATACCATGGTAAGATTCAGCCCTTGCCCAGGGGATAGTTCATTTAGTTTGTTTACTGGATAGAGCTCATCATGTGACTATACCTCAGTTAGTTTATCAGTTCTCCCATCCATGGTGACTAGGTTGCCTCTCAGCCTCTCAACAACACTGTTTCTCAGTGTCCTTGTAGAAGTGATATGTGGGTGTTTTCTCCTTACACAGAGTTGAAAGGTGACGACAACAACGTTGGCACTACCAATCCCCCACCCTCCAGAGGGGTAACCAGTGTTACCAGTTTGCTGTGTTTCCTGCTACACCTCGCCTTATTCACTTCCATTTGTATCTGAAAAACGTGTTGCATGGTTTCTTTTCTATAGAAGTGGTAAAATGCTATTGTGTCCTGTACATTATTGATTACTTTTTTTCATTTAACAGTAGGGAGATGCCTGGGAGTACACAGAGAACTGCCCTCATTGTTTTCAACTTCTGCACTGTATGTCTGTGAGTTTAGCCATTCTGCTGTTAATGGAAATTTACAGTATTCTAATCTTTTGATATTACAAACAGTTCTGTGCGATCATCGTCATACACAACCCCTTGTGCACAATGCATGAGTGTTTCTCAGGGTAGGTACCAAGAAGTGAAATTCCTGGGTCATAGGGCGTGAGTCCGACATTTTTCTCCATTCTGCCCTGTTGCCCTCCAGAGTGGGTGTCCAGCTTTGCATACCTAAGTATGAGAGTATCTGTTGTTCATATCCTCTACGACGCTCCATATATGAAACTTAAGTTTCTGCTAGTTGCCATCTTTGATCTATCATGTATGCAGTGACCTACTAAGACTGTAATTGGTACAGTAGATTCTTGTCATCTGTGTGTGAATTTAGCATTCATGGGCTTAATGCTGACAAGGCCCCCAGGGTCCAAGACATATAATCATGTATAATTTTGTCAAGGTATAATTTTTTAAATTGCTTTTGTCATGTGTCTGCTGGTGATGCCCAACCCAGTGCTCTGCACCCAGGTCACACTGTGGCTTTGTCCTCTGCTTATGCCTGCATTGCAGCAACTGTCCTGAAGAGACCAAAATTATGCAGATTTAGGTAAGTCCATGGCTAATGTTATTATATTATGTGCTATTGTAATGGATGGGGCTGTGGAGTGTATGAATTTATAAATCACTGGTCTTGTAATTAAAATTCAAACACTATAGAAAAAGGCCATGTAGAAGATAAAAGTTCCTCTATAATCCCGGACCCCTAAGATAACTACTAATGACAACTTCATTTATATTCCTTCAGACATTTTCTGGCTGTGGATGTACTAAAATGTATCCTATTATTCTCTGCCCTAAAATGGAATCATACAAGGTGTACTGTTATTTTTATGGCTCTATAACATGTCATATTGTACGTGTTGGTATGGTCATTTTAACCATTTTTCTAGTGATGGCTTTGAGGTTATTTGCAGTTTCCTAGCCATCTCAAAGTGTGCTGCGGGGATCTCTTTTGCATCCCTCTGGGTGCAGAGCTGAGGCACCCAGAGGCAGTGTCCAGAGGAGGCAGCATCTGTAGGTGTCTTCACCTGCTCTGGCTCTTGGCACATCTGGTTGGTGACACTGTTTTGTGAGATGGGTTGAAAGCACGTGCTGCCAAAATAGAATAATGTTGGTCCTCTCCTCATGTGCCGTGGAACTGGGGTAAAACTGCGTAGTGGCTGCAGCTGCCTGTCCATACCGGAATCGAGTATAACACGGTGCCTGGCTTAGCACAAAACAGTAGTGGGTCCTGCAGGCCCCAGAGTCTAATTCCTGGTATTCTTTCCCCTACACAGATTAAATAAACCAAAAACAAACTATTCTAGGAAAGCGTCTGTGACATTTGTAAAAAGTGGTATTTAATGATCTTTTATTCACTTGTCTGTTTAGTTTGTTGAAATCTTAAGTGGCATCCTGGTCTGGGAAGGAGTGCTGTCTGCGCCTGCCCTCCGCTGGGCACAGCGTGGCTGCTTCAGGGGCTAAGCACACACTTTCTGTCTTCTAAAGGGCCGCCACATGCCAGGAGCTCAGGTGTGAGCCCGGCTCTGGCTCTTACCTCATAGGGTCACTCATAGGGGCACAGGGAGCAGAACATTGTACACAGCGAGGCACCACCCGGCTTGGCATCTGCCTCGGTGGACTTACTACCTCTAGAAGGAAATACCTGAGTTCCTCTGGCCTCAGCTCCTAGAGTGACTGGTGTGCTGTCCCTGTTACTCTTCTGTCAAGGTGACAACTGTGTGACCCATCATCTGTGTGTCAAAGCAAGGCCCTGCCTGGGCCTCTGCTCCTGTGCTGACCCCAAAGGCAAATGCTTTGCTAGTTTCCTTCCAGTTAATTTCACCTATGAATAGATGTGTGAAAACTGTTCAAAGCCATACCTGCACATGTTTGAACTTCAAACCCTGTGGGTGATTCAGTGGCATCTTTCTCTAACCCCCAGCCTCCCTTCCCACAGAGGCCACCGTCATGGCCAGTTGCTGCAGTTTCTTTCCAGAGAACCTGTGTATGTGTAAAGCTGTACAGGCGTGGGTACACCACACAGCCTGTCTTGCACTGTGGACTGTTGAGTTACTAGTACATCTAGGTAAGCACCGCATATCTGTATTCATGTCTGCCTTGGTCTTTTCAACATCTGTGTGGTAGCCGTGTTTGAATTACCCATTCCCTTTTTGGGGAACCATTAAGTTGTTTCAGCAATTTTTACTGTAGATAAGGCTATACCGCATATCTGTGTACATGGGTTTTTATGTACATGGGCAAGTATATCTGTGAGAGAAAAGTTTCCTCAGGAGGAATTCTGGGCACAGCATGTGTAAATTTCTAAATATGATGGACACCCCCAGCTTCCACCTCAAGGAGGTTGGTCCCATTGACATTTCCCCACACCTTCACCCAGGCTGTGCCCTTAAACTTGGTTATTTGTCAATGTGAGAAGTGGAAAATAGTATTTAATTGTAGTTTGGATTTGTATTTCTATTGGGTTGTATACTTACTGATTAATAATAAGAGCTCTTTACATATTAAGGAAATTAACCCTTTTCAAATACATTCCTATTTCTCACTAATCTTTAAGTTTTATTGTAATATTTTGCTCTTTAGTTTATATATATATGTATATATATATATATGTATATATATATATATACATATATATATACATATATATATACTAATTTTCTTTTATGGTTCCTGGATTTTGTGAGTAGTTTGAAAAGGCTAATCCAGCTGAAGATTTTGTTGTTGTTGTTAAACCCCATGTTTTCTCCTAACTCTTTTTATTTTTATTTTGGAGGACTCTATCTAGACTTAATTTTAGCATAACAAGTGACAGGGTTAGTTAGCCTGTTGTCCTTACACCATTTTCTGGCTAATACAGCTATTAACTATTGATCTGTCTATTCACGTGCCAGTTCCTAATGGTTTTACATAGTGTAATCTGCACTTCAAAATAGCGAAGGGAAGCCCTACCTCATTATTCTACTTTTCCAGAATTCTCCTGGCTATTCCAGGCTGCATGTTTACCTTAACCTTCCCTGTGATGTCTTCATGCCGTTGTCTTCTTATGCAAGAATAAG |
| hsa_circ_0004029 | CTGTTTGGGGCAACTTCGTTAATATGAGGTCTATCCAGGAAAATGGTGAACTAAAAATTGAAAGCAAGATTGAAGAGATGGTTGAACCACTAAGAGAGAAAATCAGAGATTTAGAAAAAAGCTTTACCCAGAAATACCCACCAGTAAAGTTTTTATCAGAAAAGGATCGGAAAAGAATTTTGATAACAGGAGGCGCAGGGTTCGTGGGCTCCCATCTAACTGACAAACTCATGATGGACGGCCACGAGGTGACCGTGGTGGACAATTTCTTCACGGGCAGGAAGAGAAACGTGGAGCACTGGATCGGACATGAGAACTTCGAGTTGATTAACCACGACGTGGTGGAGCCCCTCTACATCGAGG |
| hsa_circ_0004058 | ACACTCCTAGAATATGCAGAGAAATGGAAAACTTCAGAAGATCCTTTACCTTTATTGGAGGTATACACAGTGGCTATCCAAAGTTATGTTAAAGCCCGACCTTATCTTACCTCTGAATGTGAAAATGTAGCCTTGGTTCTGGAACGCTTGGCATTAAGCTGTGTTGAACTTTTACTGTGTCTGCCTGTTGAGTTATCAGATAAACAGTGGGAACAATTTCAGACACTGGTGCAGGTAGCTCATGAAAAGCTGATGGAGAATGGCAGCTGTGAATTGCATTTTTTAGCTACTCTAGCTCAAGAGACTGGGGTGTGGAAAAACCCGGTACTGTGCACTATTCTTTCCCAGGAACCATTGGATAAGGATAAAG |
| hsa_circ_0004087 | GTTGAAAGGATTGTAGACAAGAGGAAGAACAAGAAAGGAAAATGGGAGTATCTTATCCGATGGAAAGGCTACGGGAGCACCGAGGACACGTGGGAGCCGGAGCACCACCTCTTGCACTGTGAGGAGTTTATTGATGAATTCAATGGGTTGCACATGTCCAAGGACAAGAGGATCAAGTCAGGGAAGCAGTCCAGTACCTCCAAGCTGCTGCGTGACAGTCGAGGCCCGTCGGTTGAGAAACTGTCCCACAGACCTTCAGATCCTGGAAAGAGCAAGGGGACCTCCCATAAACGGAAGCGAATTAACCCTCCCCTGGCCAAGCCAAAAAAAGGGTATTCAGGCAAGCCCTCTTCAGGAGGTGACAGGGCCACCAAGACGGTGTCTTACAGGACTACCCCCAGTGGTTTGCAAATAATGCCCCTGAAAAAGTCTCAGAACGGGATGGAAAATGGGGACGCCGGCTCTGAGAAGGATGAGAGGCACTTTGGAAATGGGTCCCATCAGCCTGGCTTGGATTTGAATGATCATGTTGGAGAGCAAGATATGGGTGAATGTGACGTGAATCACGCTACACTGGCGGAGAACGGGCTCG |
| hsa_circ_0004127 | TTGGTGTAATACGGTGCCCAGTATGCCGCCAAGAATGCAGACAGATAGACCTTGTGGATAATTATTTTGTGAAAGACACATCTGAAGCTCCTAGCAGTTCTGATGAAAAATCAGAACAGGTATGTACTAGTTGTGAAGACAATGCAAGTGCAGTTGGCTTTTGTGTAGAATGTGGAGAGTGGCTATGTAAGACATGTATCGAAGCACATCAAAGAGTAAAATTTACTAAAGATCACTTGATCAGGAAGAAAGAAGATGTCTCAGAGTCTGTTGGAGCATCTGGTCAACGCCCTGTTTTCTGCCCTGTACACAAACAAGAACAGTTGAAACTTTTCTGTGAAACATGTGATAGATTGACATGTAGAGACTGTCAGCTATTGGAACACAAAGAACATAG |
| hsa_circ_0004270 | CATTGATGTTGATGGGACAATGACAGTGGACTGGAATGAATGGAGAGACTACTTCTTATTTAATCCTGTTACAGACATTGAGGAAATTATCCGTTTCTGGAAACATTCTACAGGAATTGACATAGGGGATAGCTTAACTATTCCAGATGAATTCACGGAAGACGAAAAAAAATCCGGACAATGGTGGAGGCAGCTTTTGGCAGGAGGCATTGCTGGTGCTGTCTCTCGAACAAGCACTGCCCCTTTGGACCGTCTGAAAATCATGATGCAGGTTCACGGTTCAAAATCAGACAAAATGAACATATTTGGTGGCTTTCGACAGATGGTAAAAGAAGGAGGTATCCGCTCGCTTTGGAGGGGAAATGGTACAAACGTCATCAAAATTGCTCCTGAGACAGCTGTTAAATTCTGGGCATATGAACAGTACAAGAAGTTACTTACTGAAGAAGGACAAAAAATAGGAACATTTGAGAGATTTATTTCTGGTTCCATGGCTGGAGCAACTGCACAGACTTTTATATATCCAATGGAG |
| hsa_circ_0004302 | GTCACTCTGTGAGACTGCTGGGTCAGAAAAAGGATAATGGAAGGCGGCTTGGGGGAGCACGATTGGATTTGCCGAAGATTAGGAAGAATCCACTGATAGAAATCATTTCCATCAATACCGGGTGTCTCAATGCTTGTACCTACTGCAAAACTAAACACGCCAGAGGAAATTTGGCCAGTTATCCAATTGATGAACTAGTAGATAGAGCCAAACAATCTTTTCAAGAGGGTGTTTGTGAGATATGGTTGACCAGTGAAGACACGGGGGCTTATGGCAGAGATATTGGCACCAATCTCCCCACACTCCTGTGGAAACTGGTTGAAGTGATTCCTGAGGGAGCAATGCTGAGGCTTGGCATGACAAATCCGCCCTATATTTTAGAGCATCTGGAGGAAATGGCAAAAATCCTTAATCACCCCAGAGTCTACGCTTTTCTGCACATACCAGTCCAGTCTGCCTCCGACAGCGTACTCATGGAAATGAAAAGAGAATACTGTGTGGCTGACTTCAAAAGAGTAGTGGATTTTCTGAAAGAGAA |
| hsa_circ_0004356 | CTCATCCCACTGAAGAGCTATGGCACTTCCAATTCCTGAGCCTTTGTGAGGTTCTGCGTGTCAGTAAGCTTGCTTCCGGGCATCACCTCCGAAAACACTTGGGTTTCAGTTTTCTCTGTGAGGCTTCTTAAGGAGTGGAGGAAAGTGGATGTTTTCAAGATAACGCAGCTAACATTCAAAGAGGTTAAGTGAATTGTCCAAAGTCACACAGCAAGCACTGGAG |
| hsa_circ_0004459 | TGATACGGGAACAACACCTCCAGAGAGTGGTATTTTTGGATTTATGATAAACTTCTCTGCATTTCTTGGTGCAGCCACGATGTATACAAGATACAAAATAGTACAGAAGCAAAATCAAACCTGCTATTTCAGCACTCCTGTTTTTAACTTGGTGTCTTTAGTGCTTGGATTGGTGGGATGTTTCGGAATGGGCATTGTCGCCAATTTTCAG |
| hsa_circ_0004542 | GGCAGTGGTCTTAATTCTTTTTATGATCAACGAGAATACATAGGGAGAAGTGTTCATTATTGGAAGAAAGTTTTGCCATTGTTGAAGATAATAAAAAAGAAGAACAGTATTCCTGAACCTATTGATCCTCTGTTTAAACATTTTCATAGTGTAGACATTCAGGCATCAGAAATTGTTGAATATGAAGAAGACGCACACGTAACTTTTGCTATATTGGATGCAGTAAATGGAAATATAGAAGATGCTATGACTGCTTTTGAATCTATAAAAAGTGTTGTTTCTTATTGGAATCTTGCACTG |
| hsa_circ_0004628 | GATTTCGGCTGAATGCGTCTTCTTGGCCCATGTTCCTTTTGAAGACGCTAAATGGAGCAGAGATGGCTCCCATCAGGATTTTCCACAAGGAGCCACCATCGCCTTCCCACAACTTCTTCAAAATGGGAATGAAGCTAGAAGCTGTGGACAGGAAGAACCCTCATTTCATTTGCCCAGCCACTATTGGGGAGGTTCGGGGCTCAGAGGTGCTTGTCACTTTTGATGGGTGGCGAGGGGCCTTTGACTACTGGTGCCGCTTCGACTCCCGAGACATCTTCCCTGTGGGCTGGTGTTCCTTGACTGGAGACAACCTGCAGCCTCCTGGCACCAAAG |
| hsa_circ_0004771 | GAAGTGTTTGGATTGTGAGCTATTTCAGAACTGTTCTCAGGACTCATTATTTTAACATTTGGGAGAAACACAGCCAGAAGATGCACACTTGACTGAAGGAGGACAGGGAATCTGAAGACTCCGGATGACATCAGAGCTACTTTTCAACAGCCTTCTCAATTTTCTTTCTCAGAAAGCAGAGGCTCAGAGCTTGGAGACAGACG |
| hsa_circ_0004816 | CTGAGATCTGTATCTGTGGACCTGAATGTTGATCCCTCGCTTCAGATTGACATACCTGATGCGCTCAGTGAGAGAGACAAAGTCAAATTTACAGTGCACACAAAGACCACACTGCCCACGTTTCAGAGCCCAGAGTTTTCTGTTACAAGGCAACATGAAGACTTTGTGTGGCTACATGACACTCTTATTGAAACAACAGACTATGCTGGGCTTATT |
| hsa_circ_0005019 | AACATGGTCCAAGACAATTCCTGGGAAAGTTCAGTTCTTCTCAAGTGAGGGTTCTGACACATCTGTACCAATTCCAGTAGTGCCACTACGGGGTGTGGACGACTCCTACCCGCCCCAGAAGAAGTCCTTCATGATGCTCAAGTACATGCACGACCACTACTTGGACAAGTATGAATGGTTTATGAGAGCAGATGATGACGTGTACATCAAAGGAGACCGTCTGGAGAACTTCCTGAGGAGTTTGAACAGCAGCGAGCCCCTCTTTCTTGGGCAGACAGGCCTGGGCACCACGGAAGAAATGGGAAAACTGGCCCTGGAGCCTGGTGAGAACTTCTGCATGGGGGGGCCTGGCGTGATCATGAGCCGGGAGGTGCTTCGGAGAATGGTGCCGCACATTGGCAAGTGTCTCCGGGAGATGTACACCACCCATGAGGACGTGGAGGTGGGAAGGTGTGTCCGGAGGTTTGCAGGGGTGCAGTGTGTCTGGTCTTATGAG |
| hsa_circ_0005038 | AGGTACTTGGAAGGCTCCATCGCCCCTGAAGACAAAGGAAGCTGCTCATTGCTGCCTGGGCCTCCATTTGCCAGAGAGTTACACATCGAAATTGTGTCTTCTCCCCACTACAGCACTAATGGAAATTATGACGGTGTTCTTTACCGGCACTTTCAGATACCCAGGGTAGTCCAGGAAGGGGATGTTCTATGTGTGCCAACAATTGGGCAAGTAGAGATCCTGGAAGGAAGTCCAGAGAAACTGCCCAG |
| hsa_circ_0005101 | GCAGGATGTAGCCAATCAAATGTGCACCAAGACCAAGGAGGAGTGTGAGAAGCACTATATGAAGCATTTCATCAATAACCCTCTGTTTGCATCTACCCTGCTGAACCTGAAACAAGCAGAGGAAGCAAAAACTGCTGACACAGCCATTCCATTTCACTCTACAGATGACCCTCCCCGACCTACCTTTGACTCCTTGCTTTCTCGGGACATGGCCGGGTACATGCCAGCTCGAGCAGATTTCATTGAG |
| hsa_circ_0005129 | TTGGATTGGATGCTGCTGGCAAGACAACCATTCTGTATAAACTGAAGTTAGGGGAGATAGTCACCACCATTCCTACCATTGGTTTTAATGTGGAAACAGTAGAATATAAGAACATTTGTTTCACAGTATGGGATGTTGGTGGTCAAGATAGAATTAGGCCTCTCTGGAAGCATTACTTCCAGAATACCCAG |
| hsa_circ_0005134 | ATCACTAAAGTGGTTCTTAGCAAAGGTTGGAGGTGTCTTGAGTGCACTGTGTGTGAGGCCTGTGGGAAGGCAACTGACCCAGGAAGACTCCTGCTGTGTGATGACTGTGACATAAGTTATCACACCTACTGCCTAGACCCTCCATTGCAGACAGTTCCCAAAGGAGGCTGGAAGTGCAAATGGTGTGTTTGGTGCAGACACTGTGGAGCAACATCTGCAGGTCTAAGATGTGAATGGCAGAACAATTACACACAGTGCGCTCCTTGTGCAAGCTTATCTTCCTGTCCAGTCTGCTATCGAAACTATAGAGAAGAAGATCTTATTCTGCAATGTAGACAATGTGATAGATGGATGCATGCAGTTTGTCAGAACTTAAATACTGAGGAAGAAGTGGAAAATGTAGCAGACATTGGTTTTGATTGTAGCATGTGCAGACCCTATATGCCTGCGTCTAATGTGCCTTCCTCAGACTGCTGTGAATCTTCACTTGTAGCACAAATTGTCACAAAAGTAAAAGAGCTAGACCCACCCAAGACTTATACCCAGGATGGTGTGTGTTTGACTGAATCAGGGATGACTCAGTTACAGAGCCTCACAGTTACAGTTCCAAGAAGAAAACGGTCAAAACCAAAATTGAAATTGAAGATTATAAATCAGAATAGCGTGGCCGTCCTTCAGACCCCTCCAGACATCCAATCAGAGCATTCAAGGGATGGTGAAATGGATGATAGTCGAG |
| hsa_circ_0005148 | AACCTAAAGGCAGACCCAGAAGAGCTTTTTACAAAACTAGAGAAAATTGGGAAGGGCTCCTTTGGAGAGGTGTTCAAAGGCATTGACAATCGGACTCAGAAAGTGGTTGCCATAAAGATCATTGATCTGGAAGAAGCTGAAGATGAGATAGAGGACATTCAACAAGAAATCACAGTGCTGAGTCAGTGTGACAGTCCATATGTAACCAAATATTATGGATCCTATCTGAAGGATACAAAATTATGGATAATAATGGAATATCTTGGTGGAGGCTCCGCACTAGATCTATTAGAACCTGGCCCATTAGATGAAACCCAGATCGCTACTATATTAAGAGAAATACTGAAAGGACTCGATTATCTCCATTCGGAGAAGAAAATCCACAGAGACATTAAAGCGGCCAACGTCCTGCTGTCTGAGCATGGCGAGGTGAAGCTGGCGGACTTTGGCGTGGCTGGCCAGCTGACAGACACCCAGATCAAAAGGAACACCTTCGTGGGCACCCCATTCTGGATGGCACCCGAGGTCATCAAACAGTCGGCCTATGACTCGAAG |
| hsa_circ_0005243 | GCTAGCTTGTCACTTTCTGCAAAGGTTTCCCTCAGGGAGCCTCCTGCTGCCAGGCACCATGACAGTGAGGGGGGATGTGCTGGCCCCGGATCCAGCGTCGCCCACGACCGCAGCAGCCTCGCCCAGCGTCTCCGTGATCCCCGAGGGCAGCCCCACTGCCATGGAGCAGCCTGTGTTCCTGATGACAACTGCCGCTCAGGCCATCTCTGGCTTCTTCGTGTGGACGGCCCTGCTCATCACATGCCACCAGCCCAGGGCTCGGCTGGGCCAAGTGTGATGTCATGAGAGCTGGGACGAGACACCCAGGGGCTGCCTGGACTGAGGACTGTGGTAGAGCAGCTCTTGGCATTTATCTACATGCACCTGCGCTGCTACAGCTGCCCCAACGAGCAGCGCTACATCGTGCGCATCCTCTTCATCGTGCCCATCTACGCCTTTGACTCCTGGCTCAGCCTCCTCTTCTTCACCAACGACCAGTACTACGTGTACTTCGGCACCGTCCGCGACTGCTATGAGG |
| hsa_circ_0005270 | GGTCTCTCCTCAGGGATGACATCATCCGTCCACCTCCTTGTCTTCAAGGACCACCTCCTCTCCATGCTGAGCTGCTGCCAAGGGGCCTGCTGCCCATCTACACCTCACGAGGGCACTAGGAGCACGGTTTCCTGGATCCCACCAACATACAAAGCAGCCACTCACTGACCCCCAGGACCAGGATGGCAAAGGATGAAGAGGACCGGAACTGACCAGCCAGCTGTCCCTCTTACCTAAAGACTTAAACCAATGCCCTAGTGAGGGGGCATTGGGCATTAAGCCCTGACCTTTGCTATGCTCATACTTTGACTCTATGAGTACTTTCCTATAAGTCTTTGCTTGTGTTCACCTGCTAGCAAACTGGAGTGTTTCCCTCCCCAAGGGGGTGTCAGTCTTTGTCGACTGACTCTGTCATCACCCTTATGATGTCCTGAATGGAAGGATCCCTTTGGGAAATTCTCAGGAGGGGGACCTGGGCCAAGGGCTTGGCCAGCATCCTGCTGGCAACTCCAAGGCCCTGGGTGGGCTTCTGGAATGAGCATGCTACTGAATCACCAAAGGCACGCCCGACCTCTCTGAAGATCTTCCTATCCTTTTCTGGGGGAATGGGGTCGATGAGAGCAACCTCCTAGGGTTGTTGTGAGAATTAAATGAGATAAAAGAGGCCTCAGGCAGGATCTGGCATAGAGGAGGTGATCAGCAAATGTTTGTTGAAAAGGTTTGACAGGTCAGTCCCTTCCCACCCCTCTTGCTTGTCTTACTTGTCTTATTTATTCTCCAACAGCACTCCAGGCAGCCCTTGTCCACGGGCTCTCCTTGCATCAGCCAAGCTTCTTGAAAGGCCTGTCTACACTTGCTGTCTTCCTTCCTCACCTCCAATTTCCTCTTCAACCCACTGCTTCCTGACTCGCTCTACTCCGTGGAAGCACGCTCACAAAGGGCTAATCTCGGGCCTTGTCGAAGGAAGAGGCTGCAGACGTTAATGAGGTTAGCTGCTGGATTCCAGTATTCGTCGCATAAGGATCCTTCTTTGTCTGCGAAGGAAAAACACACTGATTATCATAATGAGTTCCTGACCTGGCCATCCCGGGGTGCCCTTGACCAGCCCCGTGTCTCCTCAGGGTGTCCCAGCACCAGCCTGGCACAGAGTGGGGCTCAGTTAGAGTATGTGGGATGTTGGTTTCGCCAGGTGAGCACGTGGGCCGTGGCCCGGCTGGGTCGGCTGAAGAACTGCGGATGGAAGCTGCGGAAGAGGCCCTGATGGGGCCCACCATCCCGGACCCAAGTCTTCTTCCTGGCGGGCCTCTCGTCTCCTTCCTGGTTTGGGCGGAAGCCATCACCTGGATGCCTACGTGGGAAGGGACCTCGAATGTGGGACCCCAGCCCCTCTCCAGCTCGAAATC |
| hsa_circ_0005442 | ATGAGAACTGGACTGATGACCAACTGCTTGGTTTTAAACCATGCAATGAAAACCTTATTGCTGGCTGCAATATAATCAATGGGAAATGTGAATGTAACACCATTCGAACCTGCAGCAATCCCTTTGAGTTTCCAAGTCAGGATATGTGCCTTTCAGCTTTAAAGAGAATTGAAGAAGAGAAGCCAGATTGCTCCAAGGCCCGCTGTGAAGTCCAGTTCTCTCCACGTTGTCCTGAAGATTCTGTTCTGATCGAGGGTTATGCTCCTCCTGGGGAGTGCTGTCCCTTACCCAGCCGCTGCGTGTGCAACCCCGCAGGCTGTCTGCGCAAAGTCTGCCAGCCGGGAAACCTGAACATACTAGTGTCAAAAGCCTCAGGGAAGCCGGGAGAGTGCTGTGACCTCTATGAGTGCAAACCAGTTTTCGGCGTGGACTGCAGGACTGTGGAATGCCCTCCTGTTCAGCAGACCGCGTGTCCCCCGGACAGCTATGAAACTCAAGTCAGACTAACTGCAGATGGTTGCTGTACTTTGCCAACAAGATGCGAGTGTCTCTCTGGCTTATGTGGTTTCCCCGTGTGTGAGGTGGGATCCACTCCCCGCATAGTCTCTCGTGGCGATGGGACACCTGGAAAGTGCTGTGATGTCTTTGAATGTGTTAATGATACAAAGCCAGCCTGCGTATTTAACAATGTGGAATATTATGATGGAGACATGTTTCGAATGGACAACTGTCGGTTCTGTCGATGCCAAGGGGGCGTTGCCATCTGCTTCACCGCCCAGTGTGGTGAGATAAACTGCGAGAGGTACTACGTGCCCGAAGGAGAGTGCTGCCCAGTGTGTGAAGATCCAGTGTATCCTTTTAATAATCCCGCTGGCTGCTATGCCAATGGCCTGATCCTTGCCCACGGAGACCGGTGGCGGGAAGACGACTGCACATTCTGCCAGTGCGTCAACGGTGAACGCCACTGCGTTGCGACCGTCTGCGGACAGACCTGCACAAACCCTGTGAAAGTGCCTGGGGAGTGTTGCCCTGTGTGCGAAGAACCAACCATCATCACAGTTGATCCACCTGCATGTGGGGAGTTATCAAACTGCACTCTGACAGGGAAGGACTGCATTAATGGTTTCAAACGCGATCACAATGGTTGTCGGACCTGTCAGTGCATAAACACCGAGGAACTATGTTCAGAACGTAAACAAGGCTGCACCTTGAACTGTCCCTTCGGTTTCCTTACTGATGCCCAAAACTGTGAGATCTGTGAGTGCCGCCCAAGGCCCAAGAAGTGCAGACCCATAATCTGTGACAAGTATTGTCCACTTGGATTGCTGAAGAATAAGCACGGCTGTGACATCTGTCGCTGTAAGAAATGTCCAGAGCTCTCATGCAGTAAGATCTGCCCCTTGGGTTTCCAGCAGGACAGTCACGGCTGTCTTATCTGCAAGTGCAGAGAGGCCTCTGCTTCAGCTGGGCCACCCATCCTGTCGGGCACTTGTCTCACCGTGGATGGTCATCATCATAAAAATGAGGAGAGCTGGCACGATGGGTGCCGGGAATGCTACTGTCTCAATGGACGGGAAATGTGTGCCCTGATCACCTGCCCGGTGCCTGCCTGTGGCAACCCCACCATTCACCCTGGACAGTGCTGCCCATCATGTGCAGATGACTTTGTGGTGCAGAAGCCAGAGCTCAGTACTCCCTCCATTTGCCACGCCCCTGGAGGAGAATACTTTGTGGAAGGAGAAACGTGGAACATTGACTCCTGTACTCAGTGCACCTGCCACAGCGGACGGGTGCTGTGTGAGACAGAGGTGTGCCCACCGCTGCTCTGCCAGAACCCCTCACGCACCCAGGATTCCTGCTGCCCACAGTGTACAGATCAACCTTTTCGGCCTTCCTTGTCCCGCAATAACAGCGTACCTAATTACTGCAAAAATGATGAAGGGGATATATTCCTGGCAGCTGAGTCCTGGAAGCCTGACGTTTGTACCAGCTGCATCTGCATTGATAGCGTAATTAGCTGTTTCTCTGAGTCCTGCCCTTCTGTATCCTGTGAAAGACCTGTCTTGAGAAAAGGCCAGTGTTGTCCCTACTGCATAG |
| hsa_circ_0005456 | GGCTGTATGACAAATGTTCTTATATCTCACGTGACCGAGGATGGGTCGTGGGCATTCACACCATCAGTGACCAAGACAACAAAGACCCACGCTACTTTTTCTCCTTGAAGACAGACCGAGCCCGGCAAGTGACCACCATCAATGCCCACCGCAGCTACCTCCCAGGCCAGTGGGTATACCTAGCTGCCACCTATGATGGGCAGTTCATGAAGCTCTATGTGAATGGTGCCCAGGTGGCCACCTCTGGGGAACAAGTGGGTGGCATATTCAGCCCACTGACCCAGAAGTGCAAAGTGCTCATGTTAGGGGGCAGTGCCCTGAATCACAACTACCGGGGCTACATCGAGCACTTCAGTCTGTGGAAGGTGGCCAGGACTCAGCGGGAGATACTGTCTGACATGGAAACCCATGGCGCCCACACTGCTCTACCTCAGCTCCTCCTCCAGGAGAACTGGGACAATGTGAAGCATGCCTGGTCCCCCATGAAGGATGGCAGCAGCCCCAAAGTGGAATTCAGCAATGCCCACGGCTTTCTGCTGGACACGAGTCTGGAGCCTCCTCTGTGCGGACAGACATTGTGTGACAACACAGAGGTCATTGCCAGCTACAATCAGCTCTCAAGTTTCCGCCAGCCCAAGGTGGTGCGCTACCGCGTGGTCAACCTCTATGAAGATGATCATAAGAACCCGACGGTGACGCGCGAGCAGGTGGACTTCCAGCACCATCAGCTGGCTGAGGCCTTCAAGCAATACAACATCTCCTGGGAGCTGGACGTGCTGGAGGTGAGCAACTCCTCCCTTCGCCGCCGCCTCATCCTGGCCAACTGTGACATCAGCAAGATTGGGGATGAGAACTGTGACCCCGAGTGCAACCACACGCTGACGGGCCACGACGGCGGGGATTGCCGCCACCTGCGCCACCCTGCCTTCGTGAAGAAGCAGCACAACGGGGTGTGTGACATGGACTGCAACTATGAACGGTTCAACTTTGATGGTGGAGAGTGCTGTGACCCTGAAATCACCAATGTCACTCAGACTTGCTTTGACCCCGACTCTCCACACAG |
| hsa_circ_0005519 | GTCAAAAGAAATAGACTGGCAACCTTATTTTACTACACGCATTGTAGATGACTTTGGCACACACTTACGAGTATTCAGAAAGGCTCAACAGAAAATAACAGAGAAAGATGATCAAGTGAAAGGTACAGCAGAAGATCTTGTAGATACCTTCTTTGAAGTTGAAGTTGAAATGGAGAAGGAGGTTTGCCGTGATCTAGTGTGCACTTCCCCCAAAGATGAAGAAGGATTCCTAAGGGATTTGTGTGAGGTCTTACTATATTTATTGCTACCTCCTGGAGATTTCCAGAACAAGATCATGCGATACTTTGTCAGGGAAATCCTTGCACGAGGAATTCTTCTTCCATTAATAAATCAACTCAGTGATCCTGATTATATTAATCAGTATGTCATATGGATG |
| hsa_circ_0005585 | GCAATGAATCGCTCCCTGGCTAATGTGATTCTTGGAGGCTATGGCACCACTTCAACAGCTGGTGGAAAACCCATGGAAATTTCTGGCACACATACGGAAATCAACCTTGACAATGCAATTGACATGATTCGAGAAGCTAATAGCATTATTATTACACCAGGCTATGGTCTCTGTGCAGCCAAAGCTCAATACCCCATTGCTGATTTGGTAAAGATGCTCACTGAGCAAGGCAAAAAAGTCAGGTTTGGAATTCACCCAGTTGCAGGCCGAATGCCTGGTCAGCTTAATGTGCTGCTGGCTGAGGCTGGTGTGCCATATGACATTGTGTTGGAAATGGATGAGATCAACCATGATTTTCCAGATACTGATTTGGTCCTTGTAATTGGAGCTAATGACACTGTTAATTCAGCAGCTCAAGAAGATCCCAACTCTATTATTGCAGGCATGCCAGTCCTTGAGGTCTGGAAATCAAAGCAGGTGATTGTTATGAAGAGGTCTTTGGGTGTTGGCTATGCTGCAGTGGACAATCCAATCTTCTACAAACCTAACACGGCCATGCTTCTAGGTGATGCCAAGAAAACATGTGACGCGCTCCAGGCGAAAGTTAGAGAATCCTATCAGAA |
| hsa_circ_0005600 | GCTGCTGAGAACTAGCCCTAGACCTCTGCGTGAGGGTTCTTCTGCCGAAGACATCACCAGTGTGTGGAGCCTGCCACACCCACCCGCTGCCAAACCACGGCCTTTACCTGTGTCTTCCGGTGTTTCCCGTGCGACCCATCCTGTGGGAGTGCCTCGTGGGCTGCCCCAGAGTTCACCCCACACTCAGCAGCACCAATGGTGAAGATGACAAGATCGAAGACTTTCCAGGCATATCTGCCCTCCTGCCACCGGACCTACAGCTGCATTCACTGCAGAGCTCACTTGGCCAATCATGATGAACTAATTTCCAAG |
| hsa_circ_0005605 | CTTGGAAGTCTTTTTGCATGTTACTGTTCCACTGAAGTCACTCAGGCAATATGGGATGGATATCTACAACAAGCAGATCCATTTTTTATTTATTTCTTAATGTTAATTATCCTTGTTAATGCAAAAGAAGTTATTTTAACACAAGAGTCAGACAGCAAAGAAGAAGTTATCAAGTTCTTGGAAAATACTCCATCCAGTCTGAATATAGAAGATATAGAAGACCTTTTCTCTCTGGCTCAGTATTATTGCAGCAAAACACCGGCTTCTTTTAGGAAGGATAATCACCATCTCTTTGGTAGTACTTTGTTGGGAATTAAGGATGATGATGCAGATCTGAGTCAGGCTCTTTGTCTGGCCATCTCCGTGTCAGAGATCCTTCAAGCGAATCAGCTACAAGGG |
| hsa_circ_0005633 | CCTTCACTTAACTTAAGGGACCTTGGATTATCTGAACTAAAAATTGGACAGATTGATCAGCTGGTAGAAAATCTACTTCCTGGATTTTGTAAAGGCAAAAACATTTCTTCCCATTGGCATACATCCCATGTCTCTGCACAATCCTTCTTTGAAAATAAATATGGTAACTTAGATATATTTAGTACATTACGTTCCTCTTGCTTGTATCGACATCATTCAAGAGCTCTTCAAAGCATTTGTTCAGATCTTCAGTACTGGCCAG |
| hsa_circ_0005692 | GTTCCTGCGTGAAGACCAGCTGGGAGCCCACTGCCTGCTGCCACCTCCAACTCCGGCCCCCTCACCATGCACTCCCTGGACGAGCCGCTCGACCTGAAGCTGAGTATCACCAAGCTCCGGGCGGCAAGAGAGAAGCGGGAGAGGACGCTGGGTGTGGTCCGGCCCCGTGCTCTGCACAGGGAGCTGGGCCTGGTGGATGACAGCCCCACACCTGGCTCTCCAGGCTCCCCGCCCTCAGGCTTCCTGCTGAACTCCAAGTTCCCCGAGAAGGTGGAGGGACGCTTTTCAGCAGCCCCTCTCGTGGACCTCAGCCTGTCACCACCATCTGGGCTGGACTCCCCCAATGGCAGCAGCTCGCTGTCCCCCGAGCGCCAGGGCAACGGGGACCTGCCTCCAGTGCCCAGTGCCTCG |
| hsa_circ_0005695 | TCCTGGAAGATACTGCTCTTTGGTGTAATAAACTTGATATGTACTGGCTTCCTGCTTATGTGGTGCAGTTCTACTAATAGTATAGCTTTAACTGCCTATACTTACCTGACCATTTTTGATCTTTTTAGAGACGGGGTTTCACCATTTTGGCTGGGCTGGTCTCAAACTCCTGACCTCAAGTGGTCCACCCACCTCGGCCTACCAAAGTGCTGGGATAACAGGCGTGAGCTACCGTGCCTGAGCAATAGTTTAATGACATGTTTAATAAGTTACTGGGTAACATTGAGGAAACCTAGCCCTGTCTATTCATTTGG |
| hsa_circ_0005768 | CATTAGTGCCAGCAGTAGTTCCCAAGGGCTGTCTCAGCCATCCACCCAGACGACTCAGTATCTGAGAGCAGACACGCCCAACAATGCAACTCCTATCACCAGCTCCTTAGGTTATCCTACCTTGCGGATAGAGAAGAACGACTTGAGAAGTGTCACTCTTTTGGAGGCCAAAGGCAAGGTGAAGGATATAGCAATATCCAGAGAGAGGATAACTCTAAAAGATGTACTCCAAGAAGGTACTTTTGGGCGTATTTTCCATGGGATTTTAATAGATGAAAAAGATCCAAATAAAGAAAAACAAGCATTTGTCAAAACAGTTAAAG |
| hsa_circ_0005806 | CTCTTCACGGCCGCCCCCCAAGGCCCCGGCCCCTCCCGTGGCTCAGCCTCCCCCCTCATCATCCTCTTCGTCCTCCTCCTCCTCATCTGCCTCCTCCTCGTCCGCGCAGCTCACCCACCGGCCCCCGACGCCCTCACTGCCCCTGCCTTTGTCCACCCACAGCTTTCCCCCTCCCGGGCTGCGGCCCCCCCCACCACCCCACCACCCCTCCTTGTTCTCCCCTGGCCCCACCCTGCCCCCACCCCCACCCCTGCTGCAGGTGCCAGGGCACCCTGGGGCCTCAGCCGCTAACGCCCTTTCTGAGCAGGACCTGATCGGCCAGGACCTGAACTCTCGCTACCTGAATGCCCAGGGTGGCCCTGAGGTGGTGGGGGCAGGGGGCTCGGCCCGGCCCCTGGCCTTCCAGTTCCACCAGCACAACCACCAGCACCAGCACACCCACCAGCACACCCACCAGCACTTCACCCCTTATCCCCCGGGCCTGCTGCCACCCCACGGCCCCCACATGTTTGAGAAATATCCAGGAAAGATGGAAGGCCTTTTCCGACATAATCCGTACACGGCCTTCCCTCCCGCAGTGCCCGGGCTGCCTCCGGGCCTCCCGCCGGCCGTCTCCTTTGGCTCCCTGCAGGGGGCCTTCCAGCCCAAGAGCACGAACCCTGAGCTGCCACCACGACTGGGGCCGGTGCCGAGCGGGCTCTCCCAGAAGGGGACACAGATCCCCGACCATTTCCGGCCACCTTTGAGG |
| hsa_circ_0006087 | ATACATATGAACCAGATGGTTACAACCCAGAAGCTCCTAGTATTACTAGTTCTGGTAGATCTCAGTACAGACAGTTCTTTTCAAGAACTCAGACACAGCGTCCCAATCTGATTGGCCTAACATCTGGAGATATGGATGTAAATCCAAGAGCTGCTAACATTGTGATCCAGACTGAACCACCAGTTCCTGTTTCGATTAATAGCAACATAACCAGAGTAGTTCTTGAACCAGATAGTCGAAAAAGAGCTATGAGTGGTTTGGAAGGGCCACTCACAAAGAAACCTTGGCTGGGAAA |
| hsa_circ_0006097 | GTGTATGGTTGAAGCCTGGAATTTTTTGCGGCAACATTGCAATAGGTTGAATATAGAGGAGTTACTGAAGCACATGTATGAAGTCTGTCAGGAAATGGGCTTGATGGAAGATTTACTGAAGTTACCATTTACAGACACTGAGCAGGAATGTTTAGTGAAATTTTTGCAGTCCAGTGCCAGCGTTCAGAATCATGAATTCCTTTTAGTGCACCATTTGCAGCGTGCCAATTATGTGCCTGCCTTGAAGCTGAACCAAACTCTGAAGATTAATGTTATGAATGATCGTGATCCTCGTTTGCGGGAGAGATCACTGGCTCGAAATTCTATATTAGACCAGTATGGAAAAATCCTTCCTAGAGTCCATCGAAAATTAGCCATTGAACGAGCTAAGCCTTATCATCTGTCAACATCATCAGTTTTTCGATTAG |
| hsa_circ_0006110 | TGGTTTTTAGATCGTATGGCTGATGACGACTGGTGGCCAATGCAGATACTAATTAAGTGCCCTAATCAAATTGTGAGACAGATGTTTCAGCGTTTGTGTATCCATGTGATTCAGAGGCTGAGACCTGTGCATGCTCATCTCTATTTGCAGCCAGGAATGGAAGATGG |
| hsa_circ_0006127 | GACTCCCTGGAGGAAAAGCGGAAGCGGCAGCGGTCTGAACGCCTGGAACGGATTTTCCAACTTAGTGAGGCTCATGGGGCCCTGGCACCTGTGTATGGGACTGAAGTCCTGGATTTCTGTACCCTGCCCCAACCTGTTGCCAGCCCCATCGGCCCTCGTTCTCCTGGCCCCAGCCACCCCACCTTTTGGACTTATACCGAGGCTGCCCACCGGGCTGTACTGTTTCCCCAGCAGCGACTAGACCAGCTGTCAGAAATCATTGAGAGGTTCATCTTTGTCATGCCTCCTGTGGAGGCACCTCCCCCTTCCCTGCATGCCTGCCACCCACCTCCTTGGCTGGCCCCACGTCAGGCAGCCTTCCAGGAGCAATTGGCCTCTGAGCTCTGGCCCCGGGCTCGTCCTTTGCACCGTATTGTGTGTAACATGCGCACCCAGTTCCCTGACTTAAGACTCATCCAGTATGATTGCG |
| hsa_circ_0006156 | GTGATTGAAGATAGTACTGGAGTCCGCCGGGTGGTGGTCACACCCCAGTCTCCTGAGTGTTATCCCCCAAGCTACCCCTCAGCCATGTCTCCAACCCATCATCTCCCTCCCTATCTGACTCACCATCCACATTTTATTCATAACTCACACACGGCTTACTACCCACCTGTTACCGGACCTGGAGATATGCCGCCTCAGTTTTTTCCCCAGCATCATCTTCCCCACACAATATATGGTGAGCAAGAAATTATACCATTTTATGGAATGTCAACCTACATCACCCGAGAAGACCAGTACAGCAAGCCTCCGCACAAAAAACTGAAAGACCGCCAGATCGATCGCCAGAACCGCCTCAACAGCCCTCCTTCTTCTATCTACAAAAGCAGCTGCACAACAGTATACAATGGCTATGGGAAGGGCCATAGTGGTGGAAGTGGCGGAGGCGGCAGCGGTAGTGGTCCCGGAATTAAGAAAACAGAGCGACGAGCAAGAAGCAGCCCAAAGTCGAATGATTCAGACTTGCAAG |
| hsa_circ_0006166 | GGCAGGGGTCTTAATTCTTCTTATGATCAACAAGAATACATAGGGAGAAGTGTTCATTATTGGAAGAAAGTTTTGCCATTGTTGAAGATAATAAAGAAGAACAGTATTCCTGAACCTATTGATCCTCTGTTTAAACATTTTCATAGTGTAGACATTCAGGTAACAGAGTTCCTTTATGAATTTATTGGAGATGGGAATTTCCAGTTTATAAACAAAGACGTGGAGCTATAAACTGCTTAAATTAATTGCCTTGTTATTTAACGGTAATCTTGTTTTCTAAATTCACTAGCTCTCACAGATAAGATATGACAGAGAAGAGTAATGAGATGTTTGTCTCTTAAGATCATATAAAATCTTTGGAAAATCATTTGGGTTTTATATTCTGAGTATAAACAATTTGACTAAAAACTATTCTGTGTGTTTAGGCATCAGAAATTGTTGAGTATGAAGAAGATGCACACATAACTTTTGCTATATTGGATGCAGTACATGGAAATATAGAAGATGCTGTGACTGCTTTTGAATCTATAAAAAGTGTTGTTTCTTATTGGAATCTTGCACTG |
| hsa_circ_0006459 | CTATCTTAATGACTTGGACCGCGTAGCTGACCCTGCCTACCTGCCTACGCAACAAGATGTGCTTAGAGTTCGAGTCCCCACCACAGGGATCATCGAATACCCCTTTGACTTACAAAGTGTCATTTTCAGAATGGTCGATGTAGGGGGCCAAAGGTCAGAGAGAAGAAAATGGATACACTGCTTTGAAAATGTCACCTCTATCATGTTTCTAGTAGCGCTTAGTGAATATGATCAAGTTCTCGTGGAGTCAGACAATGAG |
| hsa_circ_0006566 | TTGCTGAACCTGTTTGCATGAGTTGCTCCTGACGGCCCTTTAGGATACTTCCATGTCTGTAGGGTCTAGAGTGACATCTCCTCCCCTCCCCTGACGAGATCTGCCCTCCTTGGCACTGTGCTTCCCCAGAGGGGTGGCCTCGCTGTTCCCATGGACATGGCCCAG |
| hsa_circ_0006707 | TTGGTGTGGATGACTACAGCTCAGAGTCTGATGTGATTATTATACCTTCAGCCCTGGACTTTGTCTCACAAGATGAAATGTTGACGCCCCTGGGGAGATTGGACAAGTATGCTGCAAGTGAGAACATATTTAACAGACAAATGGTGGCCCGGAGTTTGCTCGATACCTTGAGGGAAGTCTGCGATGATGAAAGAGATTGTATTGCTGTTTTGGAAAGAATTAGCAGATTGGCCGATGATTCAG |
| hsa_circ_0006853 | GGGCCCGGCGGATGCCTCCTTTGCCGGAGCTTGGAACAGACTCACGGCCAGCGAAGTGAGTTCAATGGCTGAGGTGAG |
| hsa_circ_0006884 | TGAACAGCAGAGAATTTCAAAGGACCTTGCTAATATCTGTAAGACGGCAGCTACAGCAGGCATCATTGGCTGGGTGTATGGGGGAATACCAGCTTTTATTCATGCTAAACAACAATACATTGAGCAGAGCCAGGCAGAAATTTATCATAACCGGTTTGATGCTGTGCAATCTGCACATCGTGCTGCCACACGAGGCTTCATTCGTTATGGCTGGCGCTGGGGTTGGAGAACTGCAGTGTTTGTGACTATATTCAACACAGTGAACACTAGTCTGAATGTATACCGAAATAAAGATGCCTTAAGCCATTTTGTAATTGCAGGAGCTGTCACGGGAAGTCTTTTTAGGATAAACGTAGGCCTGCGTGGCCTGGTGGCTGGTGGCATAATTGGAGCCTTGCTGGG |
| hsa_circ_0006908 | GTGATGTGGAAACAGCTGTAAAATTTGCAACTCAGCTTATTGACCTGGGAGCAGACATTAGTTTGCGGAGTCGCTGGACAAACATGAATGCTTTGCATTATGCTGCTTATTTTGATGTCCCTGAACTTATAAGAGTGATTTTGAAAACATCGAAACCAAAAGATGTGGATGCCACTTGCAGTGATTTTAATTTTGGAACAGCTTTGCATATTGCAGCATACAACTTGTGTGCAGGTGCTGTGAAGTGCCTCTTGGAGCAGGGAGCAAATCCTGCATTTAGGAATGACAAAGGACAGATCCCTGCTGATGTTGTTCCAGACCCAGTAGATATGCCGTTAGAGATGGCTGACGCCGCAGCCACTGCTAAGGAAATCAAGCAGATGCTTCTAGATGCGGTGCCTCTGTCATGTAACATCTCAAAGGCCATGCTCCCAAATTATGATCATGTCACTGGCAAGGCAATGCTTACGTCACTTGGCCTGAAGTTGGGGGATCGTGTTGTTATTGCAGGACAGAAGGTTGGTACATTAAGATTTTGTGGAACAACTGAATTTGCAAGTGGGCAGTGGGCTGGCATTGAACTGGATGAACCAGAAGGAAAAAATAATGGAAGTGTTGGAAAAGTCCAGTACTTTAAATGTGCCCCCAAGTATG |
| hsa_circ_0006962 | AGGATGAAGTTTTTGCTGCTGCAGCAGAAGTACCTAGAATACCTGGAGGATGGCAAGGTCCTGGAGGCACTTCAAGTTCTACGCTGTGAATTGACGCCGCTGAAATACAATACAGAGCGCATTCATGTTCTTAGTGGGTATCTGATGTGTAGCCATGCAGAAGACCTACGTGCAAAAGCAGAATGGGAAGGCAAAGGGACAGCTTCCCGATCTAAACTATTGGATAAACTTCAGACCTATTTACCACCATCAGTGATGCTTCCCCCACGGCGTTTACAGACTCTCCTGCGGCAGGCGGTGGAACTACAAAGGGATCGGTGCCTATATCACAATACCAAACTTGATAATAATCTAGATTCTGTGTCTCTGCTTATAGACCATGTTTGTAGTAGGAGGCAGTTCCCATGTTATACGCAGCAGATACTTACGGAGCATTGTAATGAAGTGTGGTTCTGTAAATTCTCTAATGATGGCACTAAACTAGCAACAGGATCAAAAGATACAACAGTTATCATATGGCAAGTTGATCCGGATACACACCTGCTAAAACTGCTTAAAACATTAGAAGGACATGCTTATGGCGTTTCTTATATTGCATGGAGTCCAGATGACAACTATCTTGTTGCTTGTGGCCCAGATGACTGCTCTGAGCTTTGGCTTTGGAATGTACAA |
| hsa_circ_0007181 | GGCTGGTGAATTACCAGATCTCCGTCAAGTGCAGTAACCAGTTCAAGTTGGAAGTGTGTCTTTTGAATGCAGAAAACAAAGTCGTGGACAACCAGGCTGGGACCCAGGGCCAGCTGAAGGTGCTGGGTGCCAACCTCTGGTGGCCGTACCTGATGCACGAACACCCCGCCTACCTGTACTCGTGGGAGGATGGTGATTGCTCACACCAAAGCCTTGGACCCCTCCCAGCCTGTGACCTTTGTGACCAACTCCACCTACGCAGCAGACAAGGGGGCTCTGTATGTGGATGTGATCCGTGTGAACAGCTACTACTCTTGGTATCGCAACTACGGGCACCTGGAGTTGATTCAGCTGCAGCTGCCCGCCCAGTTTGAGAATTGGTGTAAGACATCACAATCCCATTATTCAGAGCGCGTATGGAGTGGAAACGCTTATAGGGTTTCACCAGGATCCACCTCTGATGTTCAGTGAAGAGTACCAGAAAAGTCTGCTAGAGCAGTACCATCTGGGTCTGGATCAAAAACGCAGAAAATACGTGGTTGGAGAGCTCATCTGGAATTTTGCCGATTTCATGACTAACCAGT |
| hsa_circ_0007209 | GTAACGAGTCTGACCTTGAACTAGAAAAGAAGTGTAAGGAAGATGATCGGGAAAAAGCCTCGAAAAGACCACGGTCACAGAAAACAGAGAAAGTCCAGAAGATCTCAGGAAAGGAGGCCAGACAGCTTTCTGGGGCGAAGAAACCCATCATAAGTGTGGTTTTAACTGCACACGAAGCAATTCCAGGTGCTACCAAGATTGTGCCAGTGGAGGCTGGGCCCCCTGAAACAGGAGCTACAAATTCTGAGACCACTTCAGCAGACCTGGTGCCTCGGAGAGGCTACCAGGAATACGCCATTCAGCAGACACCTTATGAGCAACCAATGAAGTCAAGCAGGCTAGGTCCCACTCAGCTCAAAATCTTCACTTGTGAATACTGCAACAAGGTCTTCAAGTTCAAGCACTCGCTGCAGGCCCACCTGAGGATCCACACCAATGAAAAGCCATACAAGTGCCCCCAGTGCAGCTATGCCAGTGCCATCAAGGCCAACCTCAATGTGCACCTGCGCAAGCACACTGGAGAGAAGTTCGCCTGCGACTATTGCTCGTTCACCTGCCTGAGCAAGGGCCACCTCAAGGTGCACATCGAGCGAGTGCACAAGAAGATCAAGCAGCACTGCCGCTTCTGCAAGAAGAAGTACTCTGACGTCAAGAACCTCATCAAGCACATCCGAGACGCGCATGACCCACAGGACAAGAAGGTCAAAGAGGCCTTGGACGAGCTCTGCCTGATGACGAGGGAGGGCAAGCGGCAGCTGCTCTATGACTGCCACATCTGTGAGCGCAAGTTCAAGAACGAGCTGGACCGTGACCGCCATATGCTGGTCCACGGAGACAAGTGGCCTTTTGCCTGTGAGCTCTGTGGCCATGGGGCCACCAAGTACCAGGCGCTGGAACTGCATGTCAGGAAGCACCCCTTCGTGTACGTCTGTGCCGTCTGCCGCAAGAAGTTCGTCAGCTCCATCAGGCTGCGCACCCACATCAAAGAGGTGCACGGGGCTGCCCAGGAGGCCTTGGTCTTCACCAGTTCCATCAACCAGAGCTTCTGCCTCCTGGAACCTGGTGGGGACATCCAGCAAGAAGCTCTGGGGGACCAGCTACAGCTGGTGGAAGAGGAGTTTGCCCTCCAGGGCGTGAATGCACTCAAGGAAGAGGCCTGTCCTGGGGACACTCAGCTGGAGGAGGGCCGGAAGGAGCCGGAGGCCCCTGGGGAAATGCCTGCCCCAGCTGTGCACCTGGCCTCCCCGCAGGCCGAAAGCACAGCCCTGCCACCCTGTGAGCTGGAAACCACCGTGGTCTCCTCCTCAGACCTGCATTCTCAAGAGGTGGTTTCAGATGATTTTTTGTTGAAAAATGATACCTCCTCCGCAGAGGCTCATGCTGCTCCTGAGAAGCCCCCAGACATGCAGCACAGAAGCTCAGTCCAGACGCAAGGTGAAGTGATCACACTACTGCTGTCCAAGGCCCAGAGTGCTGGGTCAGATCAGGAAAGCCATGGCGCCCAGAGCCCCCTAGGGGAAGGGCAGAACATGGCTGTGCTTTCAGCTGGTGACCCAGATCCCAGCAGGTGTCTCAGGTCAAACCCAGCTGAGGCCTCAGACCTCCTCCCTCCAGTAGCTGGTGGTGGGGACACCATCACACATCAGCCTGACTCTTGCAAAGCTGCCCCTGAGCACCGGTCAGGCATCACCGCTTTCATGAAGGTCCTGAACAGTTTACAGAAGAAGCAAATGAACACCAGCTTGTGTGAGCGGATCCGGAAGGTTTATGGAGACCTGGAGTGTGAATACTGTGGCAAACTTTTTTGGTACCAAGTGCATTTTGATATGCATGTCCGCACCCACACCCGGGAACATCTGTATTATTGCTCTCAGTGTCATTATTCTTCCATCACCAAAAACTGCCTTAAACGCCACGTAATTCAGAAACACAGTAACATCTTGCTGAAGTGTCCCACCGATGGCTGTGACTACTCAACTCCAGATAAATATAAGCTACAGGCACATCTTAAAGTTCACACAGCACTG |
| hsa_circ_0007252 | TCCATGCTCCGAGGGAGAGGAGGTTGTTACAAACACACATTCTATGGAATTGAGAGCCATCGCTGCATGGAAACCACCCCGAGCTTGGCGTGTGCTAATAAATGTGTCTTCTGTTGGCGGCACCACACCAACCCCGTGGGCACTGAGTGGCGGTGGAAGATGGACCAGCCTGAAATGATCTTGAAGGAAGCCATTGAAAACCATCAGAACATGATTAAGCAGTTTAAAG |
| hsa_circ_0007330 | CATGATACCAGTAGTCCTTTGCTAATCAGTGGAACCTCTGCAGCTGAGCTCCCATGGGCTGTAAAACCTGAAGATAAGGCCAAATATGATGCAATATTTGATAGTTTAAGCCCAGTGAATGGATTTCTGTCTGGTGATAAAGTGAAACCAGTGTTGCTCAACTCTAAGTTACCTGTGGATATCCTTGGAAGAGTTTGGGAGTTGAGTGATATTGACCATGATGGAATGCTTGACAGAGATGAGTTTGCAGTT |
| hsa_circ_0007355 | AAATGCACTCGGTGATGGAAAGAGAGCCACTATTCTGAAGAACACTTGGCCAAAGGGTCATTATCGTTATTGTGATGCTCTTTCTATGCTGGGGGAATATGACTGGGCCCTGCAAGCAAACATAAAAGCTCAAAAACTCTGTAAAAATGACCCTGAGGGAATCAAGGATCTAATTCAGCAGCATGTAAAGTTACAAAAACAAATAGAAGACCTACAAGGTCGAACAGCAAATAAGGATCCAATTAAAGCCTTTTATGAAAACAGGGCCTACACACCTAGGAGTTTATCAGCACCTATATTTACTACTTCACTTAACTTTGTGGAGAAGGAAAGAGATTTCAGAAAAATTAATCACGAAATGGCCAACGGTGGTAATCAGAATCTAAAGGTGGCGGATGAGGCGTTGAAGGTAGATGATTGTGACTGTCATCCTGAATTTTCACCACCATCAAGTCAGCCTCCAAAACATAAAGGAAAACAAAAATCTCGAAACAATGAATCAGAAAAGTTCAG |
| hsa_circ_0007376 | GTTCTCCGGGGCTTGGCGTACCTCCGAGAGAAGCACCAGATCATGCACCGAGATGTGAAGCCCTCCAACATCCTCGTGAACTCTAGAGGGGAGATCAAGCTGTGTGACTTCGGGGTGAGCGGCCAGCTCATCGACTCCATGGCCAACTCCTTCGTGGGCACGCGCTCCTACATGGCT |
| hsa_circ_0007439 | GATTGTCACCAGACCTGCAATCTATGGAGCAGATTCGGAGAATTATGAGACCTACTGATGTCCCTGATACAGGTTTGCTCTGTGATTTGCTATGGTCTGATCCAGATAAGGATGTGCAAGGCTGGGGAGAAAATGATCGTGGTGTTTCCTTTACTTTTGGAGCTGATGTAGTCAGTAAATTTCTGAATCGTCATGATTTAGATTTGATTTGTCGAGCTCATCAG |
| hsa_circ_0007503 | GGACCAGTTACTCTCCACAAGAAAATTCACACAACCACAGTGCTCTTCATAGTTCAAATTCACATTCTTCTAATCCAAGCAATAACCCAAGCAAAACTTCAGATGCACCTTATGATTCTGCAGATGACTGGTCTGAGCATATTAGCTCTTCTGGGAAAAAGTACTACTACAATTGTCGAACAGAAGTTTCACAATGGGAAAAACCAAAAGAGTGGCTTGAAAGAGAACAGAGACAAAAAGAAGCAAACAAGATGGCAGTCAACAGCTTCCCAAAAGATAGGGATTACAGAAGAGAGGTGATGCAAGCAACAGCCACTAGTGGGTTTGCCAGTGGAATGGAAGACAAGCATTCCAGTGATGCCAGTAGTTTGCTCCCACAGAATATTTTGTCTCAAACAAGCAGACACAATGACAGAGACTACAGACTGCCAAGAGCAGAGACTCACAGTAGTTCTACGCCAGTACAGCACCCCATCAAACCAGTGGTTCATCCAACTGCTACCCCAAGCACTGTTCCTTCTAGTCCATTTACGCTACAGTCTGATCACCAGCCAAAGAAATCATTTGATGCTAATGGAGCATCTACTTTATCAAAACTGCCTACACCCACATCTTCTGTCCCTGCACAGAAAACAGAAAGAAAAG |
| hsa_circ_0007509 | TTGGTGTGGATGACTACAGCTCAGAGTCTGATGTGATTATTATACCTTCAGCCCTGGACTTTGTCTCACAAGATGAAATGTTGACGCCCCTGGGGAGATTGGACAAGTATGCTGCAAGTGAGAACATATTTAACAGACAAATGGTGGCCCGGAGTTTGCTCGATACCTTGAGGGAAGTCTGCGATGATGAAAGAGATTGTATTGCTGTTTTGGAAAGAATTAGCAGATTGGCCGATGATTCAGAACCAACTGTGAGAGCGGAGCTGATGGAACAGGTGCCTCACATCGCACTGTTTTGTCAAGAAAACCGGCCTTCAATACCATATGCTTTTTCAAAATTCTTACTACCTATTGTGGTTAGATACCTTGCAGATCAGAATAATCAGGTGAGGAAAACAAGTCAGGCAGCTTTGCTGGCTCTGTTGGAGCAGGAGCTCATTGAACGATTTGATGTGGAGACCAAAGTGTGCCCTGTCCTCATAGAGCTGACAGCCCCAGATAGCAATGATGATGTGAAAACAGAAGCTGTGGCTATAATGTGCAAAATGGCTCCCATGGTTGGGAAGGATATTACAGAGCGTCTTATCCTCCCTAGGTTTTGTGAGATGTGCTGCGATTGCAGAATGTTTCACGTTCGAAAGGTCTGTGCTGCCAATTTTGGAGATATTTGCAGTGTAGTTGGCCAGCAAGCTACTGAAGAAATGTTGCTGCCCAGATTTTTCCAGCTTTGTTCTGATAATGTATGGGGAGTCCGAAAGGCTTGTGCTGAATGCTTCATGGCGGTTTCATGTGCAACATGTCAAGAAATCCGACGGACCAAATTATCAGCACTTTTTATTAATTTGATCAGTGATCCTTCACGTTGG |
| hsa_circ_0007545 | CTGTTATGAATAATGCTGCAATAGACATGGGGTTACAGTTATCTCTTCAAGACCTGGCTTTCAGTTCCGGATATATACCCAGAAGTAAGATGGTAAATTTTATCTTATAAAAATTTTTTTAAAACACATAAGGTGAAGTCAATAAAACCTCTGTTTTGATATGGCAAACCATTTTATTTTAAGGCAATGAGACAGAATTCTTTTGATGAATAAATATCACCATTACCACTCTCTCTTATAACCTCACCTAAGCCGCCTCACCTAGGCTTCCAATCTGTCAAATAACACACATAGAGATCCAAAAGTAGGGAGAAAGGAAAGAGGAGATAGGACGTTTACCTGTCAGTTCAATCTCCGTCCCCCCCCCTTTTGTTTTTTGAAGTTTGATTAAGAATTCCGGACTCTCTGATTTGCCACATAAAGTAGGCATCGATTTAGGCTTCCCTCATATTTTTAAAGACTAAAAATAAGTCTTAACATGCTTTCTTTTGACCAAAATGACCTTAAAGCTCGTCCCAACTAACATTGTTACTTAGTTGTCATAGTAGTAACTGTAGTTTTCAACTGTGCTAGGACTATTACAAATGCAATCTAGGTTGGTCAGATAACTGTTATGAATATTATGTTTCTTTTTAGAGGAACAGTAGACTAGAATGTCTCCATAGGCAAGTAAACAAGTTGATGATGAATCTGAAACATATATCCTATGAAAGGAACTGGGGATGTTTTATATTAAAAATAGACAGCCAAGACAGGAGCCATTACCTCTGTCTTCAGATATTTGAGGGACAATTATACGAAAGAGTAATTTATTTGGAACATGAGTGTAAATTACGGAAGGAAAGATTTCAATTTTACGTTTTAAAAATACTTTTTGTAACTTACAGTTGCCCATCAGGGTCCCTTGTGGGGAGGTGTACTCCCCACAAAAAATTCTTAAGAAAGTAGCTTACTGCCTGACCAACATGGAGAAACCCCATCTCTACTAAAAATACAAAATTAGCCGGGCATGGTGGCGCATGCCTGTAATCCCAGCTACTTGGGAGGCTGAGGCAGGAGAATTGCTTGAACTCAGGAGGCGGAGGTTGCAATGAGCTGAGTTCGTGCCATTGCACTCCAGCCTGCACAACAAGAGCGAAACTCCATCTCAAAAAAAAAGAAAAGAAAAAGGAAAGTAGCTTACTTTCTATTTGGAGAATTATATGGGAAATGCCTTCTTGGTGGTCTCTGGGACATTTATTTGGAGCCCTGGGGCTTTAAGGATCCAATTTGAAAACCACTGATCAAGACCAATATAGCCAGATTAAAAATCTAGTTATTCCAAATATTTATTTCCTATTTAGTCATTCAGTATAAAAAATGAAGAGCCATTGAAAGATTCTGAGCAGAATGACATCTTTTGGTTTGTATTTAGAAAGGTCACCACTTTGACAATATGGAAATTAAAATCAAAACTGTGATATTGGAAGCAAGGAGACTAGTTTGGAGGCTGCTACAATGGCCTAAGCAAGAAGTGATTAGAAGCTAAACTAAGACAGTTGCAATGATAAGTAGAGAAAAGGGGTTATAGTTAACTGTACTGAGAAGTAGAATTAAAAGACCTAATAATCAGTTTGCTATGGTGGGAAGGGAGGAGTCTGAATTAATTCTCAACTTTATTATTTTGGGGATTGAGTAAATAAGGTTGACTTTAGTGGAGATAGAATATGTCGGGGGAGCTGTAGGTTTGAAGGGAAAGATCTTGAATTTAGTTTTAGAACTGGTAAATTAATTGGCAACTCACCTCAGATTAAGTTGTCTAATAGCTAGCAAAGTTAGTTATGGAACTTAGGCGCAAAGTCTGGGCTAGAGAAGTAGTGTTGGGAGACATGGATGTAAGTGTGGCAGGCGAAGCCATGGAAGTCCAGGGACAGAGGGAATCAGAAAGACTGAGGAAGCAGTTTGATAAATATAAGAGGAAACTGAGTAAGTTCTTATGATTTTTTTTAATTTTTTTAAAGTTTATGTATTTCATATATATATATTTTTTAATTTGAACAGGGTCTTGCTCTGTTGCCCAGGCTGGAGTGCAGTGGCACAATCATAGCTCACTGCAGCCTTGACCTCCCAGGCTCAAGTGATCCTCCCGCCTCTGTCCCCAAAGTAGCTGGGACTACAGATAGGTGCCACCACACCTGGCTAATTTTTTGTATTTTTTATAGAGACAGAGTTTCACTATGTTGCTTAGGCTGGTCTTGAACTCCTGGGCTCAAGCATCCCTTCCACCTTAGCCTCCCAAAGTTCTGAGGTTACAGGCCACCATGCCTAGCCAAGTCCTCATAATTTCTTACAGCTGAAAATGTACAATAACAATAGCAATGCAGGCTTAAATGCAAAGAAAAAAAGCCCACCAAAAACACCTGAGTTTTTAAAATTTACTTTCTTTGAGAAATTTGAAATAAAGCTGAAATGATTTTTAATAAAATTCTGATAATGATTTTACTAAACTTCTGAAATGTTTCTACAATCTAAACATTCCAGACCTTTGCCTAATCATCAGCTTAATTTTTATTTGGTCTGGGTATTTTTTAATTCTTATTTTATTTCACTTTGTTCTTTATGAAATTAATTATGTTGGCCTTGAATCTGACCAGTGCAAATTTATTATATGGCTTCATAAAAAAATCATACTTCAAAGAAAATACTTCCTTAAATGTAAGAACTTATACCTTCCTTATGTTCTTTGTTTACCTAAGGAAATGCAACCTCACCACAAGTACAAAGACCATCTTTCATGGACTACTTTGATAAGCAAGACTTCAAGAATAAGAGTCACGAAAACTGTAATCAGAACATGCATGAACCCTTCCCTATGTCCAAAAACGTTTTTCCTGATAACTGGAAAGTTCCTCAAGATGGAGACTTTGACTTT |
| hsa_circ_0007616 | ATCACTAAAGTGGTTCTTAGCAAAGGTTGGAGGTGTCTTGAGTGCACTGTTTGTGAGGCCTGTGGGAAGGCAACTGACCCAGGAAGACTCCTGCTGTGTGATGATTGTGACATAAGTTATCACACCTACTGCCTAGACCCTCCATTGCAGACAGTTCCCAAAGGAGGCTGGAAGTGAAGTGCAAATGGTTCTCTAGGGTTTGTTTGCCTTGTTAGTCTTTCAAGTTCAGAGCTTTCTCATACCACTTTAGTTTTTAAAAATTAGCCATACCTATTTAATTGAATAATACACATATTCTATGATACATACCACTAAGCAGAAAAATTTTCACATACACATTAAATCATTTGCCCCATTATGTTCGTATGTAGCTTCCTGAATTACAGTTACTGAATAACTAAGAAAATAAAATGGAGACTTTTCGGGGGGATTTGGATTTCAGGTGTGTTTGGTGCAGACACTGTGGAGCAACATCTGCAGGTCTAAGATGTGAATGGCAGAACAATTACACACAGTGCGCTCCTTGTGCAAGCTTATCTTCCTGTCCAGTCTGCTATTGAAACTATAGAGAAGAAGATCTTATTCTGCAATGTAGACAATGTGATAGGTATTGTGCTGTTTTTTCATCTTTTTAAAGCTTTTCTCTTTGAAATGTAGCAAAAAAAAAAAAAAAAAGGAAAATAGCTTTTCCTTAATCACAAGTTTTAGGTACAGAACTTTTTGCCTTGTAGATTTTTAGTCACCTAGAATCTTACAGAATTGATTTCCTGTTTTGAATTCTCAACTCCAGACTAAAGTTTTGTTTTGTTTTGTTTTGTTTTGTTTTTAAATTTAGAGACAGAGTCTTGTTCTGTCGCCAGGCTGGAGTGCAGCAGCGCTATCTTGGCTCACTGCAAACTCCACCTCCACCTCCTGGGTTCAAGCGATTCTCCTGCCTCAGCCTCCGGAGTAGCTGGGACTACAGGTGCATGCCACCATGCCCAGCTAATTTTTGTATTTTTAGTAGAGACAGGGTTTCACCATGGTGGCCAGGATGGTCTCCATCTTTTGACCTTGTGATCTGCCCGCCTCAGCCTCCCAAAGTTTTATGATTATAGGCGTGAGCCACTGTGTCCACCCAAGACTAAAGATTTTTAATTTAGGCCTTTTTGAGGGTTTAGGAATCCCTTGAAATTAGATGGAGAATTATTGCCTTCATCTATGCCGTTTCTTATGAAGGGTTTCTGAATCTTTTAATTGATTATAAAAATATCTAACACTTTCTGTTCTCCTTAAACCACTTTCTCTTAAAGCTCTAGATACTAGATATCTAGGTATTAGATAGCACCTTCTGCCCTCCCTACGTAATTATGTGGAATTTCAAAATCAAGAATGTTTCCTTGCTTTCATTGGTATATTGTTGTACTCTTTAGAAGTTAAGCAGTGAACATATATTGATAGTATTATTTTATCAGTAGTACAGTATTCTTGGGACTCTGGCTACTAATTATCTGTTCCATTGCAAGACAACTTTTTACTTTATTTCCCAATTACCATTCAACATCGCTTTCCATGAGATATGTCTACTTCAAGTGAGATGCATTGCCTGGAGCCCATATATGCTAGCACTGCCATTTGCAGTTTTCTGAATACCTTTGTGTTTGCCCTAACTAGCTTCCTTGCTGTCTTTGAAATATTTAATATATGATGATAAAATAATTAGCTTCCTTATGTAATGTGCTTTGCTTCCTCTCTAATAGTTGTTCTCATTCCTTTTTATTTCCTCCTTAGCTCTATGAAAGTTTTTCTGTTACTAGGGATAGTTAGGAGAAAAGGGCAAGGTAGGAGGAGCATGTGAGGCTTAGGGCTTTTAAGTTTGAAGACTCAGTGTTACAGGTTTTAAAAGGTAGCAGTTCTCAGTATATTCCATTTTTAAAAAAAAATGTACAAATATGGTCTTTTTAGATGGATGCATGCAGTTCGTCAGAACTTAAATACTGAGGAAGAAGTGGAAAATGTAGCAGACATTGGTTTTGATTGTAACATGTGCAGACCCTATATGCCTGCATCTAATGGTAACAGAATAATTTAAACTGTGAGTCTGCACTCTTGTACCACTCTCTTGCACCTTACTGTCCATAACCAATGAATTAGCTTAGCTCTACTCTATTTTGTCTTTGTGAAACTTACTTTGACAAGTATTTTATGAAAAATATTATTGTTGGTTATACATGACTTATCACAACTTGTTATAAAACAATTTACATGAAACAATAAAAAGCATATACTTTAGATGTAAACTATAATTTTGCTTCCAAGGAACATGATCTTGTAGTTATTGACAATATGTCAAAATCCAATGTGTTGATTCTTTCTTAGCCAGGTTTTCCTCCATGACTTGAGTATTTCCTTCATCACTTCTTGTTTTGTTGTTGCTTTAAAAAGTGCTTTTAACTTTAGTGTTCAAACATTTATTTTAATAAAATGACTATAGAAACAGAAATTTTAATCATATATAAGTATGTAAATACACTTTACCTTTTCTGAAAAGAATTACCTGGATTTTTTTTTTTTTCATTTCAGTGCCTTCCTCAGACTGCTGTGGATCTTCACTTGTAGCACAAATTGTCACAAAAGTAAAAAAGCTAGGTAAAATTTGAAATGCTTTACTTAATTTAATTAATTTACTTTGCTTAATTTTTACATAATTGGCTTACCACTTCTAAAATCTGCTTCAATCATATGGGTGTTCTATCCAAATTCCATAATGTTGGTAATCATTTCCACAATGATATATAAAATGTCATCCAGCTTTACTGGGGCAGTATTCCTATAAATTTCAGCAAGTTGGCAATAAAAATAACAGCTCTTAGAATAACCATTAATGCCATACTTGCTTTGGTTTCATTGATATATTACTGTGCTTAATTATCAGTTAGCAGAAAATACGGCCTAGTTAGCAAGCAGATTTCTTTTAGAATTAATTCAATCTCTTAATTTTTTAAAATAATTAATAAGCCTAGTATGGTGATTAATATGATATTCTTATTAAACAGTCATTCTTTTGAATACTTGTATTTAATAGCACCTGATACAAAAACATTTGGATAGTACAGGAATTGTTCTAAGGAACAACAGTTTTGTATGTTTAAAATTAAATCTGCAGGATTTGTACTTATTTATTACTTCTCCCTGTTAGTAATTATGTTGATACTCTGATTTTTCCAGATGAGCTTCTGGAGTATTCTCTCTCCTCTTGTGTAAATAGATCCCTGCCTTTTGATCTTTTCCAGGAAAAAGCTCATAGTGGATTAGCTGAGCATTGCATTTATTTGCAGTGCTTCTAACTCTTTTTATTGGGACATGAAAAAAGAAATGCCAGGAAGACTTTTTTGAGACGGAGTCTCGCCCTGTCGCCCAGGCTGGAGTGCAGTGGCACGATCTTGGCTCACTGCAACCTCCGCCTCCCGGGTTCAAGCAATTCTCCTGCCTCAGCCTCCCGCATAGCTGGGATTACAGGTGCCCGCCACCACGCCCGGCTAATTTTTTGTATTTTTAGAAGAGACAGAGTTTCACTGTGTTAGCCAGGATGGTCTCGATCTCCTGACCTCGTGATCTGCCTGCCACCTCGGCCTCCCAGAGTGCTGGGATTACAGGCATGAGCCCCCGCACCCAGCCCAGGAAGACCATTTTTTAAAAACATGTAACATTTCTGCCTATAATCCAAGGAAGTTTGACCTATTGTTCCTAGTTTTTATTGGGTATCATGAAGTTAATTATTCATGCATTTCATAGATACATAATTGTTTTCTAGAACTACAGTCACATTCCCTTGATATGGGTATTTTGGAACAGTAATCAGTCAAATTTAAAATGAAAGTTTAAATTTGTATTCTTGGGATTTTGTAATTTTAGACCCACCCAAGACTTATACCCAGGATGATGTGTGTTTGATTGAATCAGGGATGACTCAGTTACAGAGCCTCACAGTTACAGTTCCAAGAAGAAAACTGTCAAAACCAAAACTGAAATTGAAGATTATAAATCAGAATAGCGTGGCCGTCCTTCAGACCCCTCCAGACATCCAATCAGAACATTCAAGGGATGGTGATATGGATGATAGTCGAG |
| hsa_circ_0007643 | GTTGAGACTGAGCTAAAGTTAATCTGTTGTGACATTCTGGATGTACTGGACAAACACCTCATTCCAGCAGCTAACACTGGCGAGTCCAAGGTTTTCTATTATAAAATGAAAGGGGACTACCACAGGTATCTGGCAGAATTTGCCACAGGAAACGACAGGAAGGAGGCTGCGGAGAACAGCCTAGTGGCTTATAAAGCTGCTAGTGATATTGCAATGACAGAACTTCCACCAACGCATCCTATTCGCTTAGGTCTTGCTCTCAATTTTTCCGTATTCTACTACGAAATTCTTAATTCCCCTGACCGTGCCTGCAG |
| hsa_circ_0007683 | ACTCTCCTTTCCAATTCCTCGGAAACAGGCACTGCCAGTGGACTGGTTTTCATCCTTAGACCAGAGCAGAGTACATGCACTTGGTACTTGGGGACTTCAGGCATACAGCCTGTCCAGAATATGGCTATCCTACTCTCCTACTCAGAAAGAGATCCTGTCCCTGGAGGCTGTAATTTGGAGTTCGATTTAGATATTGATCCCAACATTTACTTGGAGTATAATTTCTTTGAAACGACTATCAAGTTTGCCCCAGCAAACCTAGGCTATGCGAG |
| hsa_circ_0007700 | GGATTTGAGAGCACCTCCAGAACAAGGAAAGATTTTTATTGCAAGGCGCTCTCTCTTAGATGAACTGCTTGAAGTGGACCACATCAGAACAATATATCACATGTTTATTGCCCTCCTCATTCTCTTTATCCTCAGCACACTTGTAGTAGATTACATTGATGAAGGAAGGCTGGTGCTTGAGTTCAGCCTCCTGTCTTATGCTTTTGGCAAATTTCCTACCGTTGTTTGGACCTGGTGGATCATGTTCCTGTCTACATTTTCAGTTCCCTATTTTCTGTTTCAACATTGGGCCACTGGCTATAGCAAGAGTTCTCATCCGCTGATCCGTTCTCTCTTCCATGGCTTTCTTTTCATGATCTTCCAGATTGGAGTTCTAGGTTTTGGACCAACATATGTTGTGTTAGCATATACACTGCCACCAGCTTCCCGGTTCATCATTATATTCGAGCAGGATTTTCTGACTTTACTGGCTATCTATAATGCATTTGACTAATGGAAAGGACACTTGCAGGAGACTTTCTTTGAAGAGACATCTTTTTCCTCCTGCTTTTGTAGATTCGTTTTGTAATGAAGGCCCACTCATTTGTCAGAGAGAACGTGCCTCGGGTACTAAATTCAGCTAAGGAGAAATCAAGCACTGTTCCAATACCTACAGTCAACCAGTATTTGTACTTCTTATTTGCTCCTACCCTTATCTACCGTGACAGCTATCCCAG |
| hsa_circ_0008010 | GTCCCGTCAGGAAGCCCAAGTATGTGGAAAGCCCCAGAGTGCCTGGAGATGCAGTTATAATGCCATTCAGAGAAGTAGCCAAGCCAACAGAGCCTGATGAGCATGAAGCAAAGGCCGATAATGAACCGAGCTGTTCGCCGGCAGCTCAAGAACTGTTGACAAGGCTGGGATTTTTACTGGGAGAAGGGATCCCAAGTGCCACACACATAACCATTGAAGACAAAAATGAAACCATGTGCACAGCTCTGAGTCAAGGCATCAGTCCTTGCTCCACACTAACAAGCAGCACCGCATCTCCTAGCACCGATAGCCCCTGCTCAACCTTGAATAGCTGTGTCAGCAAGACGGCAGCCAACAAAAGTCCCTGTGAGACCATTAGCAGCCCTAGTTCCACCCTGGAAAGCAAGGACAGTGGAATTATAG |
| hsa_circ_0008060 | CAATGACTCAGATGCATATAAAGATCAAATATCAGTACTGCCAAATGAACAAGACTTGGTGAGAGAAGAAGCCCAGAAAATGAGTAGTCTTTTACCAACTATGTGGCTTGGAGCTCAAAATGGCTGTTTGTATGTCCATTCATCTGTAGCCCAGTGGAGGAAATGTCTCCATTCCATTAAACTTAAAGATTCGATTCTCAGTATTGTACACGTGAAGGGAATCGTGTTAGTAGCCCTGGCTGACGGCACCCTTGCAATCTTTCACAGAGGAGTGGATGGGCAGTGGGATTTGTCAAACTATCACCTCTTAGACCTTGGACGGCCTCATCATTCCATCCGTTGCATGACTGTGGTACATGACAAAGTCTGGTGTGGCTATAGGAACAAAATCTATGTGGTGCAGCCAAAGGCCATGAAAATAGAGAAATCTTTTGATGCACATCCCAGGAAGGAGAGCCAAGTGCGACAGCTTGCGTGGGTGGGGGATGGCGTGTGGGTCTCCATTCGCTTGGATTCTACGCTCCGTCTCTATCATGCACACACTTATCAACATCTACAGGATGTGGACATTGAGCCTTATGTAAGCAAAATGTTAGGTACTGGAAAACTGGGCTTCTCTTTTGTGAGAATTACAGCTCTTATGGTGTCTTGTAATCGTTTGTGGGTGGGGACAGGAAATGGTGTCATTATCTCCATCCCATTGACAGAAACCGTAATCCTCCACCAGGGACGTTTACTGGGGCTGAGGGCAAATAAAACCTCAGGTGTACCAGGAAATCGTCCTGGAAGTGTAATCCGTGTATATGGTGATGAAAACAGTGATAAAGTGACTCCAGGGACATTTATACCCTATTGTTCAATGGCACATGCACAGCTTTGCTTCCATGGGCACCGGGATGCTGTGAAATTCTTTGTGGCAGTCCCAGGTCAAGTCATCAGCCCACAAAGTAGCAGTAGTGGCACGGATCTGACGGGTGACAAAGCAGGGCCATCTGCACAGGAGCCTGGTAGTCAGACGCCCTTGAAGTCTATGCTTGTCATCAGTGGAGGAGAGGGCTACATCGACTTCCGAATGG |
| hsa_circ_0008106 | AGTGATGACAGACCTAATGCTCTATTAAGTTCACCTGCAACAGAAACAGTTCATCATTCCCCTGCATATTCTTTTCCTGCTGCTATCCAGAGAAATCAGCCTCAGCGCCCTGAAAGCTTCCTTTTCCGAGCAGGTGTCAGGGCAGAAACCAACAAAGGTCATGCTTCACCCCTTCCTCCATCTGCTGCACCTACCACTGATTCTACAGATTCCATAACAGGACAGAATTCAAGACAGAGAGAAGAAGAGCTGGAATTAATAGACCAACTGCGTAAACATATTGAGTACCGGTTGAAAGTGTCTCTACCTTGTGATCTCGGAGCAGCTCTAACTGACGGTGTTGTTCTTTGCCATTTGGCCAATCATGTGCGACCTCGATCTGTCCCAAGCATTCATGTTCCCTCACCAGCTGTA |
| hsa_circ_0008107 | TTGGATTGGATGCTGCTGGCAAGACAACCATTCTGTATAAACTGAAGTTAGGGGAGATAGTCACCACCATTCCTACCATTGGTTTTAATGTGGAAACAGTAGAATATAAGAACATTTGTTTCACAGTATGGGATGTTGGTGGTCAAGATAGAATTAGGCCTCTCTGGAAGCATTACTTCCAGAATACCCAGGGTCTTATTTTTGTGGTAGATAGCAACGATCGTGAAAGAATTCAGGAAGTAGCAGATGAGCTGCAGAAAATGCTTCTGGTAGATGAATTGAGAGATGCAGTGCTGCTACTTTTTGCAAACAAACAGGATTTGCCAAATGCTATGGCCATCAGTGAAATGACAGATAAACTAGGGCTTCAGTCTCTTCGTAACAGAACA |
| hsa_circ_0008143 | AGAACCTATTGATAATTTAACTCCTGAGGAAAGAGATGCAAGGACAGTCTTCTGTATGCAGCTGGCGGCAAGAATTCGACCAAGGGATTTGGAAGAGTTTTTCTCTACAGTAGGAAAGGTTCGAGATGTGAGGATGATTTCTGACAGAAATTCAAGACGTTCCAAAGGAATTGCTTATGTGGAGTTCGTCGATGTTAGCTCAGTGCCTCTAGCAATAGGATTAACTGGCCAACGAGTTTTAGGCGTGCCAATCATAGTACAGGCATCACAGGCAGAAAAAAACAGAGCTGCAGCAATGGCAAACAATTTACAAAAGGGAAGTGCTGGACCTATGAGGCTTTATGTGGGCTCATTACACTTCAACATAACTGAAGATATGCTTCGTGGGATCTTTGAGCCTTTTGGAAGAATTGAAAGTATCCAGCTGATGATGGACAGTGAAACTGGTCGATCCAAGGGATATGGATTTATTACATTTTCTGACTCAGAATGTGCCAAAAAGGCTTTGGAACAACTTAATGGATTTGAACTAGCAGGAAGACCAATGAAAGTTGGTCATGTTACTGAACGTACTGATGCTTCGAGTGCTAGTTCATTTTTGGACAGTGATGAACTGGAAAGGACTGGAATTGATTTGGGAACAACTGGTCGTCTTCAGTTAATGGCAAGACTTGCAGAGGGTACAGGTTTGCAGATTCCGCCAGCAGCACAGCAAGCTCTACAGATGAGTGGCTCTTTGGCATTTGGTGCTGTGGCAG |
| hsa_circ_0008163 | AGATTGCCATCTGCCCCAACAACCATGAGGTGCATATCTATGAAAAGAGCGGTGCCAAATGGACCAAGGTGCACGAGCTCAAGGAGCACAACGGGCAGGTGACAGGCATCGACTGGGCCCCCGAGAGTAACCGTATTGTGACCTGCGGCACAGACCGCAACGCCTACGTGTGGACGCTGAAGGGCCGCACATGGAAGCCCACGCTGGTCATCCTGCGGATCAACCGGGCTGCCCGCTGCGTGCGCTGGGCCCCCAACGAGAACAAGTTTGCTGTGGGCAGCGGCTCTCGTGTGATCTCCATCTGTTATTTCGAGCAGGAGAATGACTG |
| hsa_circ_0008190 | TCTGATCTGAAATGTGTCCAGGATGCCAAGGGAGGTTCTTTCTACAGAGATCACTGCCCTGTGCTAGGTGAGCAGAATGGCAACAGGAATCCTGGTGGATTGCAGATTGGTGACCTGGTAAATATAGATCTCGACCTCGAAATTGTACAGTCTTTGCAGCATGGTCATGGAGGATGGACTGATGGAATGTTTGAGACTTTAACTACAACTGGAACTGTTTGTGGCATTGATGAAGATCATGACATTGTAGTACAGTATCCAAGTGGCAATAG |
| hsa_circ_0008230 | ATGCTGAGATGAATCGTCACCTGTGTGTTTGGCTTTTTAGACATCCATCTCTTAATGGTTACCTCCAGTGTCACATCCAGCTCCATTCTCATCAATTTAGACAGATACATCTTGATACAAGGCTGCAAGTTTTTAGACAAAACAGGAATTGCATTCTTCATCTGTTAAGTAAGAATTGGTCCAGGAGATATTGCCATCAAGACACCAAGATGCTCTGGAAGCATAAAGCACTACAGAAATATATGGAGAACCTGAGTAAGGAGTACCAAACACTTGAGCAATGTCTGCAGCATATCCCTGTGAATGAGGAAAACCGAAGGTCCTTGAACAGAAGGCATGCTGAGTTGGCACCTCTTGCAGCCATTTACCAAGAAATTCAGGAGACTGAACAAGCAATTGAAGAATTAGAATCAATGTGTAAAAGCCTAAATAAACAAGATGAAAAGCAGTTACAAGAACTTGCACTGGAAGAAAGGCAAACCATTGATCAAAAAATCAACATGTTGTACAATGAGCTTTTCCAGAGCCTTGTGCCAAAGGAGAAATATGACAAAAATGATGTTATTTTAGAGGTGACAGCTGGAAGGACTACTGGAGGTGACATCTGCCAACAATTTACCCGAGAAATATTTGACATGTACCAGAATTATTCGTGCTATAAACACTGGCAATTTGAACTTCTGAATTATACACCAGCAGATTATG |
| hsa_circ_0008260 | GGCAGCGGTCTTAATTCTTTTTATGGTCAACTAGAATACATAGGGAGAAGTGTTCATTATTGGAAGAAAGTTTTGCCATTGTTGAAGATAATAAAGAAGAACAGTATTCCTGAACCTATTGATCCTCTGTTTAAACATTTTCATAGTGTAGACATTCAGGCATCAGAAATTGTTGAATATGAAGAAGACGCACACATAACTTTTGCTATGTTGGATGCAGTAAATGGAAATATAGAAGATGCTGTGACTGCTTTTGAATCTATAAAAAGTGTTGTTTCTTATTGGAATCTTGCACTG |
| hsa_circ_0008276 | ACAAATGACCATGGAAACAGTTGAATCCCAGCATGATGGAAGTATAACAGCTTCTTTGACAGAGAGCAAGTCTGCTCATGTGCAGACTCAGACTGGCCAAAATTCAATCCCTGCTTTAGCTCAGGTAGGCAATAGGCAGGCACCGTTGAAAGTCAAAATATATGGAAATGAGAATTAGGTGCAGATTCTGTTTTCTTGCCATTGATCTTAAATTTTCCATGTTCCCTGGTTATCAGCCATTCTTGCTTATTTGATTGTGAAGAAAAGATACGTGTTGTAATTAGCCTTGCATTTTAACAGACATTCAGCAGTGCCCCATCTCCATTGCCACCACCACTATGGAAGGAAAAACAAAAACCCTTCCTGAATATGTAATCCTCAAAGTATGATTTGAGAGGGTTGCTAATTCGAAATTGATACAGTTCTGATGTCCTTAGCTGTTAAGATGACAGAAATCTTACCATTTTCCCCGTATATATTTGTCTTAATCATTACTTTCTTTTCAGTTAGGTCTCGAACTTTGAATTGGTTTCATTCAGAGGTCACCAGTATCAGTTAGTACCTGTTTCTAGATCCTAAAATTTATTTCTGAGATATCTATTAAAAATATAGTAAAAATAAACACATTTGTTAGTTCATCAAATATTTATTGAATGTCTGCTGTGTCTAATTATTCCGGATGCATGTGGATATGTAGTTTCAGGACTTAAATATCCCATGGTTTGGTAGGGTAGACAGATATTTTAGCAATAATTCCAGTAGGGTAAGAAGTGCTGTAAGATACTATGTAGAAAATGAATAAGGCCTACAGAAGAGGGAGCAGCCAGCTGACTAGGAGTGCTTTATATATCTCCTTTGAGCTAGGCACAAAGGATGAGTAGGAGTCTGCCAGGTTGATAAGGTGAGGAAGCATATTCTAGGCAGAGCGAAGAGTGTGCACAGAGGCACAGAGCCAGAGCATGGGATGTTCAGGGAACTATGGGTATTGAGTTTAACTGGAGCCAGGTGTCTGGGCTGGGCAGTCCAGCTGGTAAAGGTAAGTAGAGGATCGTATACATTTCAGGGTTTAGAGTACACTGTACTCTAAATAGTATTGAAATATTTTCAGGTGCCCACCGTATTTTCAGTTTTGACACTAGTAGCCATGTGGAGGTGGAATTGAAAGTCAGTGAGTCGGAACTGAAGACACCATCAGGGTGCCTGGTGGAGTCAAAGTAGCGTGAGATACAGAGCAGTGGTGAGACCTTTGTGGTACATTAAAGGGAGAATAAAAAGGACCTGGTGGTTGACTGGAAGGCCCAGGGGAAACACCAGGCACTTCTTGGGTTATATTAGGCCAGACTGGTGTGAGAGCTCTCCTTTCCTGTGCACCCAGCCTGCTTTTCTTTCCTCTTTAAAGCTGCCAACACCCACTCGTTCCCTGCAGGTTTTCTGCGGTATTCTGTAGAGCTGTATCTCCCTAGCAACACCTTTTGTTACACTGAGAGACAGGCATTTAGGTTAGGCCAGGCCTTTTGCTTAAACACACACACACACACACACACACACACACACACACACACACACACACACCCTTGTTAAAAAAAATGCATGTTCATTGTAGAAAATAAATATTAAAATGAAGAATATAAAAACTATCCATAATCTTAACATCCAGAGATTTAATCATTGTTAACATTTTGGTGTAGCTCCTTTAGTCTTTTTGTCTGTGTGAATAGAATAGAAAGCATATACTTTTTCTCATGATTTTGGGATTTTCCAGAATCTTTAGAGCTATGGTTTCTGGTCAGTAATTCTGCCCTTTGCTGAAGTTGTAGTTGAAAGCACTTTCTTTTTTTTTTGGTAGAGACAAGTTCTCACTATATTGCCCAGGCTGGTCTCAAACTCCTGTGCTGAAGCAGTCCTTGCACCTCAGCTTCCCAAAGTGCTGGAATTACAGGTGTGAGCTTCTTGTACCCAGCCTGAAAGTACATTTTCTTCCTTCTGACTCTACTTCCTTCTAGGTGGGTCCCGGCTCACTAAGTTCTACAGATGCCTTTTGCCCCAGAATTCTGGGGCTGAAGTCCCAGCAATAGATTTCCATTATCTAAATATGGTAATTTGCAATTCTGAGGACCTTCAGGCTGGGCGACAGCCACCTCTGTGAGCCTCACTCAGAAATTGCCAACAGGTGGCCAGGTGCAGTGGCTCATATCTGTAATCCCAGTACTTTGGGAGGCCAAAGCAGGTGGATCGCTTGAGGCCAGGAGTCCGAGACCAGCCTGGCCAACATGAAGAAACCCTGTCTCTACTAAAAATACAAAACTAGGTGGTTATGGTGGCACACGCCTGTAGTCGCAGCTGCTCGGGAGGCTGAGGCACGAGAATCACTTGAACCCAGGAGGCTGAGGTTGCAGTGAGCCAAGTTGGAGCCACTTCACTCCGGCCTGGGTGACAGAGTGAGACCCTGTCTCAAAAAAAAAAAGTGTTGCCAACAGGTTAAGTAATGTGGAGTCTGAAAAGTAGTCTTAGGTTTCTGAGGGCTTGAAAGTGTTGCCAACAGGTTAAGTAATGTGGAGTCTGAAAAGTAGTCTTAGGTTTCTGAGGGCTTGGAATTGAATAGGAGACCAGTGGACAGAAACTAGGTTTTAGTAGGTTATAGAATAAGTAAGAGGAGGGGAAGTGGAAGTTGGAAGTATAGACTCTTTTAAGAAATTTGTCTTGAACTAATAGAGAAAGATGGGTTTGTGAAACAAAGGGGTGAGAACTGGGATGTGAGAAATTTGGGAATGTTTTGAATAAAATGGAACATTATAGAGACTTGTGAGAAGGCAGAATCAAGGAACTAGAAAAAGCCTGTGTTTACGTTTGTAATGCTTCTTTAAAAATCTCAAATAATGTTGTATGCTAATAAATATTGATAGTTATTAAATCTGGATAGTAAATTCTTGGAGTTTTGCTGTTTTTCTATTTTATATTGTTAGAAGTATTTTTTTGTTGTTGTTTTTGTTTTGTTTCGTTTTGTTTTTTTGAGACAGAGTCTCACTATGTCACCCAAGCTGGAATGCAGTGGTGCAATCTCGGCTCACTGCAACCTCTGCTCACTGCATCCTCCACCTCCCGGGTTCCAATGATTATTATGCCTCAGCCTCCCGAGTAGCTGGGATTACAGGCATGTGCCACCACGCCCAACTGATTTTTGTGTTTTTAGTAGAGAGGGTTATGCCATGTTGGCCAGGCTGGTTTTGAACTCCTGACCTCAAGCAGTCCACCTGCTTTGATCTCCTGAAGTGCTGGGATTACTGGTGTGAGCCACTGCACCTGGCCTGTTTGAAGTATTTGATCAAACTGAAACTATGGTTTTTATCTTTCACATGCTAGAAATATAGAGATTAAGAAGTAATCTGTATGCCGACTTATGGATTATTTTAGTTAACTAAGAATTATCCTCCATGCCATGCATGGATCATGATTTTTTGAAGAATTAGAAGACAATTCTTAGCAGTCAGAATTTTCTGAACATAATTACTCATTAAAGTTCTTGTGACACCAGCAGCTGTTCGTTTATGAGTATTGTTACTATCATACAATAGTATATGTCTTACAAGGAGTAGGAAATCAAGCTGAACTCTATTAACCCTATGATGTATGCCAAAAGTGACTGACTCTTCTGAATTTCTGATGGCTTCATATTTACTTGCCACTTGTCTCATTTTTCTTCAGACTTGAGGATTGTAAATTTCCATATGTTTTAGATTTAAACTTAGTTGTGAATAAAGAGGAATTTCTGACACTCACCTTCCTTGTTTATATGATCAGATTATCAGTAAACAGTGTGGGAACAAAAGGCGTAAGCAGAACGTTCAGTGGGTTTGATTCTTTGCATCTAAGGTCATTTGATACTGGTTGTTTTGATTGTCCTGGCCTTTCCAGAGCTTCTGCTAATCAAGAATATACTATGAAAATCATCAGAGACCTAGCAACTGTTGAGCTTGGTTTTGGCTGTCAGGCCCCTGTTCTGCTGATGGATTGAG |
| hsa_circ_0008351 | AGTGATGACAGACCTAATGCTCTATTAAGTTCACCTGCAACAGAAACAGTTCATCATTCCCCTGCATATTCTTTTCCTGCTGCTATCCAGAGAAATCAGCCTCAGCGCCCTGAAAGCTTCCTTTTCCGAGCAGGTGTCAGGGCAGAAACCAACAAAGGTCATGCTTCACCCCTTCCTCCATCTGCTGCACCTACCACTGATTCTACAGATTCCATAACAGGACAGAATTCAAGACAGAGAGAAGAAGAGCTGGAATTAATAGACCAACTGCGTAAACATATTGAGTACCGGTTGAAAGTGTCTCTACCTTGTGATCTCGGAGCAGCTCTAACTGACGGTGTTGTTCTTTGCCATTTGGCCAATCATGTGCGACCTCGATCTGTCCCAAGCATTCATGTTCCCTCACCAGCTGTAGTAAGTTGATAATCCTAAAAAGCCTTGGCTATTCATCAGACATTTGTTAAGCACAGTGGACAGACATTTCTTAAGCATTGTGCTAGATATTAAGGATACAGATATAAAGAGTCCAGTCTCTACCTTCCTGGTCTCACTTCTACTCGAGAAGAAATAGACATGTATACAGCTACTTACAGCAGTGCGATAAATGTAAGAAAGGCATGTACAGTGTGTGTTATAGGGATATAAAAAGGGGCCACTTAACTCCACCTGGAGAGTTCAGGGAAGCCTTCCCAGAACGGGCAAAATTTGAACTAATTCTTGAAGTTTAAGAAGGAATTTTCCAGCAGTACAGAGAGGGGAGGGGGCCTTCCAGGCATAGGATTTCTAGATTCAGAAGCTAAGGAAAGTTTGGCTTTTACTTAAGGAATTGGTAAATTGTTCAGAATGGATCGTGCACAGGGTTGTAATACAAATGGTAAGGGGTAAGGCTAGAGACGTGAGCTGGGGCCGTGGTACAAAGGCCTTATGAACCGTGTGAAGGACGTTTACCCTAGTACAGACAATGAGGATACACTGAATTAAAGAGGGAATACCATGATTCTAACTGCAGTTTAGAATGACGAATCTAGGCTGGGCGCGGTGGCTCATGCTTATAATCTTTGCAATTTGGGAGGCCGAGGCAGACGATTGCTTGAGCCCAGGAGTTTGAGACCAGCCAGGACAATATGGCAAAACCCCGTCTCTACCAAAAATACAAAAATCAGCTGGGTATGGTGGCATGTGCCTATAGTCCCAGCTACTCAGGAGGCTGATGTGGGAGGATCACTTGAGCCCAGGAGTTCGAGGCTGCAGTGAGCCGTGATTGTGCCAGTGCACTCCAGCCTAGGTGAGAGAGCAAGACCCTGTCTCAAAGAAAAAAAAAAAAAAAAAAAAGGAATGATGAATCTAGGTGCTAAGTGAGAAATGGAAAAATTAAAGGTGGTACACTACAAAGATGCCCAAGCCAGGCTCAGTGGCTTGTGCCTGTAGTCCCAGCTACTTGGGAGCCTGAGGCATGAGGATCCTATGAGCCCAGGAGTTTGAGACCAGCTTGGGTGATCTTGTTTCTTAAAAGAAAAAGGAAAAGAGACTGGGTGCACTGGCTTACGCCTATAATCCCAGCGTTTTGGGAGCCCGAGGCTGGTGGATCACCTGAGGTCAGGAGTTCGAGACCAGCCTGGCCAACATGGTGAAACCCCAACTCTACTAAAAATACAAAATTAGCCAGGAGTGGTTCATACATCTGTAATCCCAGCTACTTGGGAGGCTGAGCCAGGAGAATCACTTGAACCCAGGAGGCGGAGCTTGCAGTGAGCTGAGATCGCACCACTGCACTCCAGCCTGGGCAAGACAGAGCGAGACTCTGTCTCAAAAGAAAAAAAGGGAAAAGAAAAGAAAGATGCCCAATTAAAAGGCTGTTGTATTAGTCCAGGCCAGGAAGATAACAAGCCGGGACTAAAGCCTGGACTAACTGGGCAGTGAGAGGGAAGATGGTTCAAAGGTAAAACTGACATGATCTGTGACCGACCTCAGGTGGAAGATGAGGGAGAAAATGCATTCAGGATAACTGGCAGGTTCTCTTTGCACTGCTGGTCAGGTCTGGGCATCAGTTTCAGGATGGCAGTCCACAAAAGAACAGGCTTTGGGTTGACACACCCCTCCTCTGTTTTCCTCACATTTTTATCATGAAAAATTTCAACCTTCAGAAAAGTGGAAAGAATCCTACAACGAACACTATTAAAGTCTGCACCTAGATCTGACGTTTCTTGCATTTTGCCGCACTTGCTTTATGTATCTATATTTGTATGTGTATTTTAACTACACCATCCAAAACTTACAGATGTAATATACTTTTCCCCCAGTGTGTTTCAGGGACCTGTCCTAGGCCACCTTGCTCTTCCCTTAAGCCCATAGCCAGTGACGGACAAGCATGGGGCTGAGCCCTCCTACCTCAAGGAGGGAGTGTCGTGTGGTTCACACTTGTGAGCTCCCTGTGGGATCAAGCTGAAGCTGAGAAGGAGACAAGCAGGAGGATCACCTGTGGCTGGGAGTTTAAGACCAGCCTGGGCAATATAGTGAGATCCCATCTCTACAAAAATACTTAAAAAAATTAGGCGTGCACCTGCTACTCAGGAAGCTGTGGCGGGAGGATCACTTGGGCCCAGGAGTTTGAGGCTGCAGTGAGCCACGATCATGCCACTGCACAACAACCTGGGCAACAGAGCGAGACCCTGACCCTAAAAAAGAAAGAAAGGGCCAGGCACAGTGGCTCTTGCCTGTAATCCCAGCACTTTGGGAGGCCCAGGTGGGCAGATCACGAGGTCAGGAGTTCGAGACCATCCTGGCCAACATGATGAAACCCCATCTCTACTAAAAATACAAAAATTAGCTGTGCATGGTGGTGCACACCTGTAGTCCCAGCTACTTGGGAGGCAGAAGCAGGAGAATCACTTGAGTCCAAGAGACGGAGGTTGCAGTGAGCCAAGATAGCACCACCACACTCCAGCCTGGCGACAGAGTGAGACTCTGTCTCAAAAGAAAAAAAAAAAAAAAAAAGACTGAAAGGAAATGAATCAAGTGTTGTAGTTACATTTAAATGATGAACTATGGGTAATTAATTTTTTTGGCTGTTCCTCCAAGTTTTCTTTAGTGCAGGGGTCCCCAACCCCTGGGCCACAAATGGGTACCAGTCTGTGGCCTGTTAGGAACTGGGCTGCACAGCAGGAGGTGAACAGGCCAGTGAGCAAAGCTTCATCTGTATTTACAGCCACTCCCCATTACTCACATCACCACCCGAGCTCTGCCTCCTGTCAGATCAGCAACAGTATTAGATCCTCAGAGGAGCATGAACGCTGGTGTGAACTGCGCATGCGAGGAAGGGATCTAGGCCACGGGCTCCTTATGAGAATCTAATGCCTGATGATCTGTCAGTGTCTCCCATCATCCCCAGATGGGACTGTCTAGTTGCAGGTAAACAAGCTCAGGGCTCCCACTGACTCTACATTACGGTAGATTTCATTATGTATGATTATTTCATTATATATTACAATGTAATAATAGAAATAAAATACACAATAAATGTAATGTGCCTGAATCAGCCTGAAACCATCCCCCCCAGCTCCAGCCCTGTCCATGGAAAAATTGTCTTCTGTGAAACCAGTCCCTGGTGCCAAAAAGGTTGAGGACTGCTGCCTTAGTGTTACACTGTTTTAAAATGGAAAAAAAAAGTTTGTAACTATTCGTTCAAAGTTTTTCTATTTAGTACTTGCCTTCCAAAATCAAAGCCAATGTCTCAAGTGTGGGGTGACAAAGTACTGCCCACAGGCCAGCACTTCTTCTTGTAAATCAAGTTTCACTGGAACCCAGTCACACTTTCTTGTTTGCATATTGTCTATAGTTGCTTTCAAGCTCCTGGCAGACTCGAGTGGTTGCAACAGTTCTTTTTTTTTTTTTGAAATGGAGCTTTGTTCTTGTTGCCCAGGCAGGAGTGCAATGGTGCGATCTCAGCTCCCCACAACCTCTGCCTCCCGGGTTCCCGGGTTCAAGTGATTCTCGTGCCTCAGCCTCCCGAGTAGCTGGGATTACAGGTGCCCGCCACCACACCTGGCTAATTTTTGTATTATTAGTAGAGACGGGGTTTCTCCATGTTCGTCAGGCTGGTCTCGAACTCCCGACCTGAGGTGATCCGCCCACCTCAGCCTCCCAAAGTGCTGGGATTATGGCGTGAGCCACCGCACCTGGCATTGCAACAGTTCTTATGGCCCTCAGAACTAACATTTATTATCTGATCCTTTAAAAAAAAGTTCGCTGACCCCCTAGTCTACAGCATTGAGAGTTATGAAGCGATTGCCAGGAGTATTTCTAAAGTTAGCAAATATTTTTTAATGTTGCTAGTAGGCTGGGCATTTAGCTGATGCCTCTGATCCCAGCACTTTGGGAGGCTGAGGCAGGCAGATTGCTTGAACTCAGGAATTCAAGACCAGCCTGGGCAACGTGGCGAACCCTGTCTCTACAAAAATTGCCCAGGTGTGATGGCATGCAACTATAGTCCCAGCTACTTGGGAGGCTGAGGTGGGAGGATCACCTGAGCCTGGGAGGTCAATGATACAGCGAGCCAGGCTTGCGCCACTGCACTCCAGCCTGGGTGGCAGTGAGACCCTGTCCCAGAAACAAAACTTCTCTAGTAACACATTTTCTTTTCCTCATTTCAGCCTAAATTAACAATGGCGAAATGCAGGCGAAATGTGGAAAATTTCCTAGAAGCTTGCAGAAAAATTGGTGTACCTCAG |
| hsa_circ_0008521 | ACCTAATCTGGGAGCCTGCAAGTGACAACAGCCTTTGCGGTCCTTAGACAGCTTGGCCTGGAGGAGAACACATGAAAGAAAGAACCTCAAGAGGCTTTGTTTTCTGTGAAACAGTATTTCTATACAGTTGCTCCAATGACAGAGTTACCTGCACCGTTGTCCTACTTCCAGAATGCACAGATGTCTGAGGACAACCACCTGAGCAATACT |
| hsa_circ_0008602 | CCTCAGACGATACAGCAGACTCTCAAGAGGACACTGCAGTATTATGAACATCAAGTTATTGGTTACAGGGATGCAGAAAAGAATTTCCACAATATCTCTAACAGATGCTCCTATGCAGACCACTCCAACAAAGAAGAAATTGAAGATGTCTCAGGAATTCTTCAGTGTACTGCTAATATACTCG |
| hsa_circ_0008774 | CCTTCCTTGAATCCAGAAGCTGGCAAACAGCATCAGCCATGCAGACCTATTGGGACACCTTCTGGAGTATGGGAAAACCCGCCTAGTGCCAAGCAACCCTCCAAGATGCTAGTTATCAAAAAAGTTTCCAAAGAGGATCCTGCTGCTGCCTTCTCTGCTGCATTCACCTCACCAGGATCTCACCATGCAAATGGGAACAAATTGTCATCCGTGGTTCCAAGTGTCTATAAGAACCTGGTTCCTAAGCCTGTACCACCTCCTTCCAAG |
| hsa_circ_0008792 | AGCCTACTTGGATGTTAATGAGCTGAAGAACATTCTTAAATTGGATGGATCAACACATCTCAATATTTTCTTTGCAAAATCCTCAGAGGAGGAGTTGGCAGGAGTAGCAACTTGGCCATGGGACAAGGAGGCCCTGATGCACTTAGGTGGCATTGTCTTGAACCCATCTTTCTATGGCATGCCTGGGCACACCCACACCATGATCCATGAGATTGGTCACAGCCTGGGCCTCTATCACGTCTTCCGAGGCATCTCAGAAATCCAGTCCTGCAGTGACCCCTGCATGGAGACAGAGCCCTCCTTCGAGACTGGAGACCTCTGCAATGATACCAACCCAGCCCCTAAACACAAGTCCTGTGGTGACCCAGGGCCAGGAAATGACACCTGTGGCTTTCATAGCTTCTTCAACACTCCTTACAACAACTTCATGAGCTATGCAGATGACGACTGTACGGACTCCTTCACGCCCAATCAAGTCGCCAGAATGCACTGTTACCTGGACCTGGTCTACCAGGGCTGGCAGCCCTCCAGGAAACCAGCGCCTGTTGCCCTCGCCCCCCAAGTTCTGGGCCACACAACGGACTCTGTGACACTGGAGTGGTTCCCACCTATAGATGGCCATTTCTTTGAAAGAGAATTGGGATCAGCATGTCATCTTTGCCTGGAAGGGAGAATCCTGGTGCAGTATGCTTCCAACGCTTCCTCCCCAATGCCCTGCAGCCCATCAGGACACTGGAGCCCTCGTGAAGCAGAAGGTCATCCTGATGTTGAACAGCCCTGTAAGTCCAGTGTCCGCACCTGGAGCCCAAATTCAGCTGTCAACCCACACACGGTTCCTCCAGCCTGCCCTGAGCCTCAAGGCTGCTACCTCGAGCTGGAGTTCCTCTACCCCTTGGTCCCTGAGTCTCTGACCATTTGGGTGACCTTTGTCTCCACTGACTGGGACTCTAGTGGAGCTGTCAATGACATCAAACTGTTGGCTGTCAGTGGGAAGAACATCTCCCTGGGTCCTCAGAATGTCTTCTGTGATGTCCCACTGACCATCAGACTCTGGGACGTGGGCGAGGAGGTGTATGGCATCCAAATCTACACGCTGGATGAGCACCTGGAGATCGATGCTGCCATGTTGACCTCCACTGCAGACACCCCACTCTGTCTACAGTGTAAGCCCCTGAAGTATAAGGTGGTCCGGGACCCTCCTCTCCAGATGGATGTGGCCTCCATCCTACATCTCAATAGGAAATTCGTAGACATGGATCTAAATCTTGGCAGTGTGTACCAGTATTGGGTCATAACTATTTCAGGAACTGAAGAGAGTGAGCCATCACCTGCTGTCACATACATCCATGGAAGTGGGTACTGTGGCGATGGCATTATACAAAAAGACCAAGGTGAACAATGCGACGACATGAATAAGATCAATGGTGATGGCTGCTCCCTTTTCTGCCGACAAGAAGTCTCCTTCAATTGTATTG |
| hsa_circ_0008802 | AATCATATTCTAAGAACTCAGCCACTCAGGTATCCACCATGGTGCTGGGTCCTGAACAGAAGATGTCAGATGACAGTGTTTCTGGAGATCATGGGGAGTCTGCCAGTCTTGGTAACATCAACCCTGCCTATAGTAATCCCTCTCTTTCACAGTCCCCTGGGGACTCAGAGGAGTACTTCGCCACTTACTTTAATGAGAAGATCTCCATTCCTGAGGAGGAG |
| hsa_circ_0008812 | GTGAAAGCACTGAAAGAGAAGATTGAATCTGAAAAGGGGAAAGATGCCTTTCCAGTAGCAGGTCAAAAATTAATTTATGCAGGCAAAATCCTCAATGATGATACTGCTCTCAAAGAATATAAAATTGATGAGAAAAACTTTGTGGTGGTTATGGTGACCAAACCCAAAGCAGTGTCCACACCAGCACCAGCTACAACTCAGCAGTCAGCTCCTGCCAGCACTACAGCAGTTACTTCCTCCACCACCACAACTGTGGCTCAGGCTCCAACCCCTGTCCCTGCCTTGGCCCCCACTTCCACACCTGCATCCATCACTCCAGCATCAGCGACAGCATCTTCTGAACCTGCACCTGCTAGTGCAGCTAAACAAGAGAAGCCTGCAGAAAAGCCAGCAGAGACACCAGTGGCTACTAGCCCAACAGCAACTGACAGTACATCGGGTGATTCTTCTCGGTCAAACCTTTTTGAAGATGCAACGAGTGCACTTG |
| hsa_circ_0008838 | GGGCCATTGACATTTAGGGATGTGGCCATAGAATTTTCTCTGGAGGAGTGGCAATGCCTGGACACTGCTCAGCAGGATTTGTATAGAAAAGTGATGTTAGAGAACTACAGAAACCTGGTCTTCTTGGCAGGTATTGCTGTTTCTAAGCCAGACCTGATCACCTGTCTAGAGCAAGGAAAAGAGCCCTGGAATATGAAGAGACATGCGATGGTAGATCAACCCCCAG |
| hsa_circ_0008839 | ATACTGTGGATGGTAGGGAAGAAAAGTCTGCTTCTGATTCTTCTGGAAAACAGTCTACTCAGGTTATGGCAGCAAGTATGTCTGCTTTTGATCCTTTAAAAAACCAAGATGAAATCAATAAAAATGTTATGTCAGCGTTTGGCTTAACAGATGATCAGGTTTCAGGGCCACCCAGTGCTCCTGCAGAAGATCGTTCAGGAACACCCGACAGCATTGCTTCCTCCTCCTCAGCAGCTCACCCACCAGGCGTTCAGCCACAGCAGCCACCATATACAGGAGCTCAGACTCAAGCAGGTCAGATTGAAG |
| hsa_circ_0008870 | CTCTGCTTATGATAATGTCAACAAAGTTCGAGTAGCTATCAAGAAAATCAGCCCCTTTGAGCACCAGACCTACTGCCAGAGAACCCTGAGGGAGATAAAAATCTTACTGCGCTTCAGACATGAGAACATCATTGGAATCAATGACATTATTCGAGCACCAACCATCGAGCAAATGAAAGATGTATATATAGTACAGGACCTCATGGAAACAGATCTTTACAAGCTCTTGAAGACACAACACCTCAGCAATGACCATATCTGCTATTTTCTCTACCAGATCCTCAGAGGGTTAAAATATATCCATTCAGCTAACGTTCTGCACCGTGACCTCAAGCCTTCCAACCTGCTGCTCAACACCACCTGTGATCTCAAG |
| hsa_circ_0008922 | GATGACTTGAAAGTAGGGCATCCTTCACCCATCTGAAGGGAGGAAATAGTGGCAGGTGACAGTCTGCATGTGCAGTTTTCAGATGCCTTCACCTGAATGACATCTACCTCCATCAGGACCCCAGATGTCTGACAGCCCTGTGTGACACCAAGATAAGTAAC |
| hsa_circ_0008955 | CACTTCTACCCTAGCCGGGCCCAGCCCCCGAGCAGTGCAGCCTCCCGAGTGCAGAGTGCAGCCCCTGCCCGCCCTGGCCCAGCTGCCCATGTCTACCCTGCTGGATCCCAAGTAATGATGATCCCTTCCCAGATCTCCTACCCAGCCTCCCAGGGGGCCTACTACATCCCTGGACAGGGGCGTTCCACATACGTTGTCCCGACACAGCAGTACCCTGTGCAGCCAGGAGCCCCAGGCTTCTATCCAGGTGCAAGCCCTACAGAATTTGGGACCTACG |
| hsa_circ_0008984 | GCTGAACATATGCAGAAGAAAAGGAGAAAACAAGAAAACAAAGATGAAGCATTGCCGCCACCACCACCTCCTGAATTCTGCCCTGCTTTCAGCAAAGTGATTAACCTTCTCAACTGTGATATCATGATGTACATTCTCAGGACCGTATTTGAGCGGGCAATAGACACAGATTCTAACTTGTGGACCGAAGGGATGCTCCAAATGGCTTTTCATATTCTGGCATTGGGTTTACTAGAAGAGAAGCAACAGCTTCAAAAAGCTCCTGAAGAAGAAGTAACATTTGACTTTTATCATAAGGCTTCAAGATTGGGAAGTTCAGCCATGAATATACAAATGCTTTTGGAAAAACTCAAAGGAATTCCCCAGTTAGAAGGCCAGAAGGACATGATAACGTGGATACTTCAG |
| hsa_circ_0008998 | GTTCATGCTGAATACTCTAGATTTGTGAATCAGATTAATACTGCTGTACCTTTACCAGGCTATACACAACCCTCTGCTATAAGTAGTGTCCCTCCTCAACCACCATATTATCCATCCAATGGCTATCAGTCTGGTTACCCTGTTGTTCCCCCTCCTCAGCAGCCAGTTCAACCTCCCTACGGAGTACCAAGCATAGTGCCACCAGCTGTTTCATTAGCACCTGGAGTCTTGCCGGCATTACCTACTGGAGTCCCACCTGTGCCAACACAATACCCGATAACACAAGTGCAGCCTCCAGCTAGCACTGGACAG |
| hsa_circ_0009043 | AATCAAGTGACGGATACTAATAGAAAACTACAACATGAGGGAAAGGAACTGGTAATAGCAATGGAAGAGCTGAAGCAGTGTCGACTACAACAGAGAAATATTTCTGCCACTGTTGATAAATTAATGCTGTGTCTTCCAGTCCTAGAGATGTACAGCAAACTGAGGGACCAGATGAAAACTAAAAGGCATTATCCTGCACTGAAAACTCTGGAACATCTAGAGCATACCTACCTGCCTCAAGTAAGCCACTATCGATTCTGCAAGGTGATGGTGGACAACATCCCCAAGCTTCGAGAAGAAATAAAAGATGTTTCTATGTCCGATCTCAAAGACTTTCTGGAGAGCATCCGCAAACATTCAGACAAAATTGGAGAGACTGCCATGAAGCAA |
| hsa_circ_0009061 | GTAGAGTACAGAGAGATGGATGAAAGCTTGGCCAACCTCTCAGAAGATGAGTATTATTCAGAAGAAGAGAGAAATGCCAAAGCAGAGAAGGAAAAGAAGCTTCCCCCACCACCCCCTCAAGCCCCACCTGAGGAAGAAAATGAAAGTGAGCCTGAAGAACCATCGGGGCAAGCAGGAGGACTTCAAGACGACAGTTCTGGAGGGTATGGAGACGGCCAAGCATCAGGTGTGGAGGGCGCAGCTTTCCAGAGCCGACTTCCTCATGACCGGATGACTTCTCAAGAAGCAGCCTGTTTTCCAGATATTATCAGTGGACCACAACAGACCCAGAAGGTTTTTCTTTTCATTAGAAACCGCACA |
| hsa_circ_0009076 | TGGTATTCATGGGTAGTTTGAAATATCCAGATGAGAATGGATTTGATGCCTTCCTGAAGAAGCATGGGGGTAGTGATAATGCCTCAACTGATTGTGAACGCACTGTCTTTCAGTTTGATGTCCAGAGGAAGTACTTCAAGGAAGCTCTTGATAGATGGGCGCAGTTCTTCATCCACCCACTAATGATCAGAGATGCAATTGACCGTGAAGTTGAAGCTGTTGATAGTG |
| hsa_circ_0009109 | GTTAATACAATCTGTTGGAATGACACTGGAGAATATATTTTATCTGGCTCAGATGACACCAAATTAGTAATTAGTAATCCTTACAGCAGAAAGGTTTTGACAACAATTCGTTCAGGGCACCGAGCAAACATATTTAGTGCAAAGTTCTTACCTTGTACAAATGATAAACAGATTGTATCCTGCTCTGGAGATGGAGTAATATTTTATACCAACGTTGAGCAAGATGCAGAAACCAACAGACAATGCCAATTTACGTGTCATTATGGAACTACTTATGAG |
| hsa_circ_0009319 | CGTCTGCTTCTACGCTTTGCCTGGGAGAGGCCCTGGTGGCCTCGTTCCTGGCGCCCGGAGTCCCTGCTGCGGCCCCACCCCCGGGCGGTCACGGTGACCCATGCTGCCCAGCCTGGAGGTAAAATCGTTCGTGGCTGTGGCTTCAGCATGTCGTCCTCGGTGAAAACCCCAGCACTGGAAGAGCTGGTTCCTGGCTCCGAAGAGAAGCCGAAAGGCAGGTCGCCTCTCAGCTGGGGCTCTCTGTTTGGTCACCGAAGTGAGAAGATTGTTTTTGCCAAGAGCGACGGCGGCACAGATGAGAACGTACTGACCGTCACCATCACGGAGACCACGGTCATCGAGTCAGACTTGGGTGTGTGGAGCTCGCGGGCGCTGCTCTACCTCACGCTGTGGTTCTTCTTCAGCTTCTGCACGCTCTTCCTCAACAAGTACATCCTGTCCCTGCTGGGAGGCGAGCCCAGCATGCTAGGTGCGGTGCAGATGCTGTCCACCACGGTTATCGGGTGTGTGAAAACCCTCGTTCCTTGCTGTTTATATCAGCACAAGGCCCGGCTTTCCTACCCACCCAACTTCCTTATGACGATGCTGTTTGTGGGTCTGATGAG |
| hsa_circ_0010674 | ATCGTGGGCTCCCGGCTGCGGATCCCTCAGGTGACTCCGGCAGACTCGGGCGAGTACGTGTGTCACGTCAGTAACGGTGCAGGCTCCCGGGAGACCTCGCTCATCGTCACCATCCAGGGCAGCGGTTCCTCCCACGTGCCCAGCGTCTCCCCACCGATCAGGATCGAGTCGTCTTCCCCCACGGTGGTGGAAGGGCAGACCTTGGATCTGAACTGCGTGGTCGCCAGGCAGCCCCAGGCTATCATCACATGGTACAAGCGTGGGGGCAGCCTTCCCTCCCGACACCAGACCCATGGCTCCCACCTGCGGTTGCACCAAATGTCTGTGGCTGACTCGGGCGAGTATGTGTGCCGGGCCAACAACAACATCGATGCCCTGGAGGCCTCCATCGTCATCTCCGTCTCCCCTAGCGCCGGCAGCCCCTCCGCCCCTGGCAGCTCCATGCCCATCAGAATTGAGTCATCCTCCTCACACGTGGCCGAAGGGGAGACCCTGGATCTGAACTGCGTGGTCCCCGGGCAGGCCCATGCCCAGGTCACTTGGCACAAGCGTGGGGGCAGCCTCCCCAGTCACCATCAGACCCGCGGCTCACGGCTGCGGCTGCACCATGTGTCCCCGGCCGACTCGGGTGAATACGTGTGCCGGGTGATGGGCAGCTCTGGCCCCCTGGAGGCCTCAGTCCTGGTCACCATCGAAGCCTCTGGCTCAAGTGCTGTCCACGTCCCCGCCCCAGGTGGAGCCCCACCCATCCGCATCGAGCCCTCCTCCTCCCGAGTGGCAGAAGGGCAGACCCTGGATCTGAAGTGCGTGGTGCCCGGGCAGGCCCACGCCCAGGTCACGTGGCACAAGCGTGGAGGAAACCTCCCTGCCCGGCACCAGGTCCACGGCCCACTGCTGAGGCTGAACCAGGTGTCCCCGGCTGACTCTGGCGAGTACTCGTGCCAAGTGACCGGAAGCTCAGGCACCCTGGAGGCATCTGTCCTGGTCACAATTGAGCCCTCCAGCCCAGGACCCATTCCTGCTCCAGGACTGGCCCAGCCCATCTACATCGAGGCCTCCTCTTCACACGTGACTGAAGGGCAGACTCTGGATCTGAACTGTGTGGTGCCCGGGCAGGCCCATGCCCAGGTCACGTGGTACAAGCGCGGGGGCAGCCTCCCCGCCCGGCACCAG |
| hsa_circ_0010872 | ACAGCCGAGCTCACTCCGGAACTTGTCATCTCCAACGACAAAAGGAGCTTTTGCCACTGACTCGGCCGTGTCCTGACACCTCCAG |
| hsa_circ_0012571 | CTTGTGGCTATAGGAATGAGGAATCACCCATTGGATTTGCGAGTGCAGTTCACAGCCAGTGCTTGCGCTCTCAACCTAACACGCCAGGGCCTGGCCAAGGGGATGCCTGTTCGCCTGTTGTCAGAGGTCACCTGTCTACTTTTCAAGGCTCTGAAAAATTTCCCCCATTACCAGCAGTTACAGAAGAATTGTCTTCTCTCCTTAACCAATTCCAGGATTCTTGTGGATGTTCCATTTGACAGGTTTGATGCTGCCAAGTTTGTCATGAGATGGCTCTGTAAGCATGAAAACCCCAAGATGCAAACAATGGCAGTGAGTGTCACCTCTATTCTGGCTCTGCAGCTCTCACCTGAGCAAACGGCACAGCTTGAAGAGCTTTTCATGGCAGTTAAGGAACTTCTAGCAATAGTAAAACAAAAGACTACTGAGAATTTAGATGATGTCACCTTCTTGTTTACTTTGAAAGCACTTTGGAATCTTACAGATGGGTCTCCAGCTGCCTGCAAGCACTTCATTGAAAATCAAGGATTGCAAATCTTCATCCAAGTCTTGGAGACCTTTTCAGAGTCAGCAATACAAAGCAAAGTACTTGGTCTTTTG |
| hsa_circ_0012989 | GTGTTCCTCCAAGAAAAGCTGGTTATATTGAAGACCTGGTTTTGATGCTGCCTCAGAACATTTGGGATAACCTATATAGCAGGTATGGTGGAGGACCAGCTGTCAACCATCTGTACATTTGTCATACTTGCCAAATTGAGGCGGAGAAAATTGAAAAAAGAAGAAAAACTGAATTGGAAATTTTTATTCGG |
| hsa_circ_0013348 | GTATGTTAGAGCGGAAGTTCTGGCTGGCTTTGTCAATGGCCTATTTTTGATCTTCACTGCTTTTTTTATTTTCTCAGAAGGAGTTGAGAGAGCATTAGCCCCTCCAGATGTCCACCATGAGAGACTGCTTCTTGTTTCCATTCTTGGGTTTGTGGTAAACCTAATAGGAATATTTGTTTTCAAACATGGAGGTCATGGACATTCTCATGGCTCTGGCCACGGACACAGTCATTCCCTCTTTAATGGTGCTCTAGATCAGGCACATGGCCATGTCGATCATTGCCATAGCCATGAAGTGAAACATGGTGCTGCACATAGCCATGATCATGCTCATGGACATGGACACTTTCATTCTCATGATGGCCCGTCCTTAAAAGAAACAACAGGACCCAGCAGACAGATTTTACAAGGTGTATTTTTACATATCCTAGCAGATACACTTGGAAGTATTGGTGTAATTGCTTCTGCCATCATGATGCAAAATTTTGGTCTGATGATAGCAGATCCTATCTGTTCAATTCTTATAGCCATTCTTATAGTTGTAAG |
| hsa_circ_0014088 | GACTGACCACTGAGCAGATGCTGAGAAAAGACCAGAAGACTATCTATAGACAAGGCGTCAAGGTGGCCATTAGTGCAATATATATGGATTTGGAGGCCTTTCTGCAGAGGTCTAACCTCCTTGCAGATCTCCATGCTTTCTGCCAGGCTCACAGCTATGATGTCCTGGTTGCCATGACTATCTTTTTCAACACTCACAATGAGCCAGTGCGGCAGTTGGCTATTTTCTGTCCCCATGTGGCACTCCAAACAACG |
| hsa_circ_0014604 | TTCCAAGATGAGATGGGATTCTCCAACATGGAAGATGATGGCCCAGAAGAGGAGGAGCGTGTGGCTGAGCCTCAAGCTAACTTTAACACCCCTCAAGCTCTACGCCAGCTTCTGTTACTGATGTAAGGATGCAGAGTCCTGTCAGGAGTTAGGTGTCAAGTGAAGTCTCCTTGTTATGGGGCAGTGCAGCTGTAGGCCAAGCTGTATCTGTTTGGGAAGGGAGAAAAAACAGTAGCTGGCATCCATATCCACTTCTCCGGGTTTGAGGAACTACTGGCCAACCTACTAAATGAACAACATCAGATAGCGAAGGAACTATTTGAACAGCTGAAGATGAAGAAACCTTCAGCCAAACAGCAGAAGGAGGTAGAGAAGGTTAAACCCCAGTGTAAGGAAGTTCATCAGACCCTGATTCTGGACCCAGCACAAAGGAAGAGACTCCAGCAGCAGATGCAGCAGCATGTTCAGCTCTTGACACAAATCCACCTTCTTGCCACCTGCAACCCCAATCTCAATCCGGAGGCCAGTAGCACCAGGATATGTCTTAAAGAGCTGGGAACCTTTGCTCAAAGCTCCATCGCCCTTCACCATCAGTACAACCCCAAGTTTCAGACCCTGTTCCAACCCTGTAACTTGATGGGAGCTATGCAGCTGATTGAAGACTTCAGCACACATGTCAGCATTGACTGCAGCCCTCATAAAACTGTCAAGAAGACTG |
| hsa_circ_0014664 | TGCTGCATTAGACGTGGAACTATCTGATGATTCCTTCCCTCCAGAAGACTTTGGCATTGTGTCTGGAATGCTCAATGTCAAATGGGACCGGATTGCCCC |
| hsa_circ_0015094 | ACTCAAGAATGAACAATCCGTCAGAAACCAGTAAACCATCTATGGAGAGTGGAGATGGCAACACAGGCACACAAACCAATGGTCTGGACTTTCAGAAGCAGCCTGTGCCTGTAGGAGGAGCAATCTCAACAGCCCAGGCGCAGGCTTTCCTTGGACATCTCCATCAGGTCCAACTCGCTGGAACAAGTTTACAGGCTGCTGCTCAGTCTTTAAATGTACAGTCTAAATCTAATGAAGAATCGGGGGATTCGCAGCAGCCAAGCCAGCCTTCCCAGCAGCCTTCAGTGCAGGCAGCCATTCCCCAGACCCAGCTTATGCTAGCTGGAGGACAGATAACTGGG |
| hsa_circ_0015415 | GTATAGCCGAGACCATGTGGTGGAAGGGGAACCGTATGCTGGTTATGATAGACACAATGCAGAGGTAGCAGCCTTTCACTTGGACAGGATTCTGGGTTTCCACCGAGCCCCCTTGGTAGTTGGCAGATTTGTTAATCTTCGGACAGAGATCAAACCTGTCGCCACAGAGCAGCTGTTGAGCACCTTCCTAACTGTAG |
| hsa_circ_0015430 | GTGTATGTGACTGCTGAGAGCCGCTTCAGCACCTTGGCAGAGCTTGTACACCATCACTCCACAGTGGCTGATGGGCTGGTGACAACATTACACTACCCAGCACCCAAGTGTAATAAGCCTACAGTCTATGGTGTGTCCCCCATCCACGACAAATGGGAAATGGAGCGAACAGATATTACCATGAAGCACAAACTTGGGGGCGGTCAGTATGGAGAGGTTTACGTTGGCGTCTGGAAGAAATACAGCCTTACAGTTGCTGTGAAAACATTGAAGGAAGATACCATGGAGGTAGAAGAATTCCTGAAAGAAGCTGCAGTAATGAAGGAAATCAAGCATCCTAATCTGGTACAACTTTTAG |
| hsa_circ_0016683 | TGATGACCAGATCCTGGAATGGTGTGGGGAGGACGCCATCCACTACCTGTCCTTCCAGAGGCACATCATCTTCCTGTTGGTGGTGGTCAGCTTTTTGTCCCTGTGTGTCATCCTGCCTGTCAACCTCTCAGGGGACTTGCTGG |
| hsa_circ_0017242 | GCTGAGTCATCACTAGAGAGTGGGAAGGGCAGCAGCAGCAGAGAATCCAAACCCTAAAGCTGATATCACAAAGTACCATTTCTCCAAGTTGGGGGCTCAGAGGGGAGTCATCATGAGCGATGTTACCATTGTGAAAGAAGGTTGGGTTCAGAAGAGGGGAGAATATATAAAAAACTGGAGGCCAAGATACTTCCTTTTGAAGACAGATGGCTCATTCATAGGATATAAAGAGAAACCTCAAGATGTGGATTTACCTTATCCCCTCAACAACTTTTCAGTGGCAAAATGCCAGTTAATGAAAACAGAACGACCAAAGCCAAACACATTTATAATCAGATGTCTCCAGTGGACTACTGTTATAGAGAGAACATTTCATGTAGATACTCCAGAGGAAAGGGAAGAATGGACAGAAGCTATCCAGGCTGTAGCAGACAGACTGCAGAGGCAAGAAGAGGAGAGAATGAATTGTAGTCCAACTTCACAAATTGATAATATAGGAGAGGAAGAGATGGATGCCTCTACAACCCATCATAAAAGAAAGACAATGAATGATTTTGACTATTTGAAACTACTAGGTAAAGGCACTTTTGGGAAAGTTATTTTGGTTCGAGAGAAGGCAAGTGGAAAATACTATGCTATGAAGATTCTGAAGAAAGAAGTCATTATTGCAAAGGATGAAGTGGCACACACTCTAACTGAAAGCAGAGTATTAAAGAACACTAGACATCCCTTTTTAACATCCTTGAAATATTCCTTCCAGACAAAAGACCGTTTGTGTTTTGTGATGGAATATGTTAATGGGGGCGAGCTGTTTTTCCATTTGTCGAGAGAGCGGGTGTTCTCTGAGGACCGCACACGTTTCTATGGTGCAGAAATTGTCTCTGCCTTGGACTATCTACATTCCGGAAAGATTGTGTACCGTGATCTCAAGTTGGAGAATCTAATGCTGGACAAAGATGGCCACATAAAAATTACAGATTTTGGACTTTGCAAAGAAGGGATCACAGATGCAGCCACCATGAAGACATTCTGTGGCACTCCAGAATATCTGGCACCAGAGGTGTTAGAAGATAATGACTATGGCCGAGCAGTAGACTGGTGGGGCCTAGGGGTTGTCATGTATGAAATGATGTGTGGGAGGTTACCTTTCTACAACCAGGACCATGAGAAACTTTTTGAATTAATATTAATGGAAGACATTAAATTTCCTCGAACACTCTCTTCAGATGCAAAATCATTGCTTTCAGGGCTCTTGATAAAGGATCCAAATAAACGCCTTGGTGGAGGACCAGATGATGCAAAAGAAATTATGAGACACAGTTTCTTCTCTGGAGTAAACTGGCAAGATGTATATGATAAAAAG |
| hsa_circ_0017254 | GCTGAGTCATCACTAGAGAGTGGGAAGGGCAGCAGCAGCAGAGAATCCAAACCCTAAAGCTGATATCACAAAGTACCATTTCTCCAAGTTGGGGGCTCAGAGGGGAGTCATCATGAGCGATGTTACCATTGTGAAAGAAGGTTGGGTTCAGAAGAGGGGAGAATATATAAAAAACTGGAGGCCAAGATACTTCCTTTTGAAGACAGATGGCTCATTCATAGGATATAAAGAGAAACCTCAAGATGTGGATTTACCTTATCCCCTCAACAACTTTTCAGTGGCAAAATGCCAGTTAATGAAAACAGAACGACCAAAGCCAAACACATTTATAATCAGATGTCTCCAGTGGACTACTGTTATAGAGAGAACATTTCATGTAGATACTCCAGAGGAAAGGGAAGAATGGACAGAAGCTATCCAGGCTGTAGCAGACAGACTGCAGAGGCAAGAAGAGGAGAGAATGAATTGTAGTCCAACTTCACAAATTGATAATATAGGAGAGGAAGAGATGGATGCCTCTACAACCCATCATAAAAGAAAG |
| hsa_circ_0018477 | AGCAGGTTGTTGCCCTGGATGCCCAAAATATTGTAGCTGTTTCATGTGGAGAAGCTCATACGTTAGCGCTAAATGACAAAGGCCAGGTGTATGCTTGGGGTCTCGATTCTGATGGACAGCTTGGCCTGGTAGGATCAGAGGAATGCATCAGAGTACCCAGAAATATTAAAAGTTTGTCAGATATCCAGATTGTACAGGTTGCTTGTGGTTACTATCATTCACTTGCACTTTCTAAAGCAAGTGAAGTCTTCTGTTGGGGACAGAATAAATATGGCCAATTGGGTTTAGGTACTGACTGTAAAAAGCAAACTTCACCGCAGCTGCTTAAGTCTTTGCTTGGAATCCCTTTCATGCAAGTTGCAGCAGGAGGAGCCCATAGTTTTGTACTCACCCTTTCTGGAGCTATCTTTGGATGGGGACGCAACAAGTTTGGTCAGCTAGGTCTTAATGATGAAAATGATAGGTATGTTCCTAATTTACTAAAGTCACTAAGATCTCAGAAAATAGTTTATATTTGTTGTGGAGAAGATCATACTGCTGCTCTAACCAAGGAAGGTGGAGTGTTTACTTTTGGAGCTGGAGGGTATGGTCAGTTGGGCCATAATTCTACCAGTCATGAAATAAACCCAAGGAAAGTTTTTGAACTTATGGGAAGCATTGTCACTGAGATTGCTTGTGGACGGCAGCACACTTCTGCTTTTGTTCCTTCATCAGGACGAATTTACTCTTTTGGGCTTGGTGGTAATGGGCAGCTGGGAACCGGTTCAACAAGCAACAGGAAAAGCCCCTTTACTGTAAAAGGAAATTGGTACCCCTATAATGGGCAGTGTCTACCAGATATTGATTCTGAAGAATATTTCTGTGTAAAAAGAATTTTCTCAGGGGGAGATCAAAGCTTTTCACATTACTCTAGTCCCCAGAACTGTGGGCCACCAGATGACTTCAGATGTCCCAATCCGACAAAGCAGATCTGGACAGTGAATGAAGCTCTAATTCAGAAATGGCTGAGCTATCCTTCTGGAAGGTTTCCTGTGGAGATAGCCAATGAGATAGATGGAACGTTTTCTTCCTCTGGTTGCCTAAATGGAAGTTTTTTAGCTGTTAGCAATGATGATCACTATAGAACAGGTACCAGATTTTCAGGGGTTGATATGAATGCTGCTAGGCTTTTATTCCACAAACTTATACAACCTGATCATCCGCAGATATCTCAGCAGGTGGCAGCTAGTTTGGAAAAGAATCTTATTCCTAAACTGACTAGCTCCTTACCTGATGTTGAAGCATTGAGGTTTTATCTTACTCTACCAGAATGTCCCCTGATGAGTGATTCCAACAATTTCACAACAATAGCAATTCCCTTTGGTACAGCTCTTGTGAACCTAGAAAAGGCACCACTGAAAGTACTTGAAAACTGGTGGTCAGTACTTGAACCTCCACTATTCCTCAAGATAGTAGAACTTTTTAAGGAAGTTGTGGTACATCTTTTGAAACTCTACAAGATCGGTATTCCCCCTTCTGAAAGAAGAATTTTCAACAGTTTTCTTCATACTGCATTAAAGGTTTTAGAAATACTACATAGGGTAAATGAGAAAATGGGACAGATTATACAGTATGATAAATTTTATATACATGAAGTACAAGAATTGATAGACATAAGAAATGATTATATCAACTGGGTCCAACAGCAGGCCTATGGAATGGATGTCAACCATGGATTAACTGAGTTGGCAGATATCCCTGTTACAATCTGTACATATCCATTTGTATTTGATGCCCAAGCAAAAACTACTCTGTTACAGACCGATGCAGTCTTACAGATGCAGATGGCTATTGATCAGGCCCACAGGCAGAATGTCTCCTCTCTTTTTCTCCCAGTGATTGAATCTGTGAATCCCTGCTTAATTCTAGTGGTGCGTAGAGAAAATATTGTAGGAGATGCAATGGAAGTCCTTAGGAAAACAAAGAACATAGATTACAAGAAGCCACTCAAGGTTATATTTGTTGGAGAAGATGCTGTGGATGCAGGAGGGGTGCGCAAAGAATTTTTCTTGCTCATCATGAGGGAATTATTGGATCCTAAATACGGCATGTTTAGGTATTATGAAGATTCCAGGCTCATTTGGTTTTCTGATAAGACATTTGAAGACAGTGATTTGTTCCATTTGATTGGTGTTATCTGTGGCTTAGCAATTTATAATTGTACCATTGTGGACCTCCATTTTCCTTTGGCTTTATATAAGAAACTACTGAAAAAGAAGCCATCCTTGGATGATTTGAAAGAACTAATGCCTGATGTTGGGAG |
| hsa_circ_0018687 | ACATCACGGCTGCAGCCCTGGCTACGGGTGCCTGCATCGTAGGAATCCTCTGCCTCCCCCTCATCCTGCTCCTGGTCTACAAGCAAAGGCAGGCAGCCTCCAACCGCCGTGCCCAGGAGCTGGTGCGGATGGACAGCAACATTCAAGGGATTGAAAACCCCGGCTTTGAAGCCTCACCACCTGCCCAGGGGATACCCGAGGCCAAAGTCAGGCACCCCCTGTCCTATGTGGCCCAGCGGCAGCCTTCTGAGTCTGGGCGGCATCTGCTTTCGGAGCCCAGCACCCCCCTGTCTCCTCCAGGCCCCGGAGACGTCTTCTTCCCATCCCTGG |
| hsa_circ_0019079 | ATCTAAATGTTAAAGAGAAAATAATTGAAGACATGCGAATGACACTAGAAGAACAGGAACAAACTCAGGTAGAACAGGATCAAGTGCTTGAGGCTAAATTAGAGGAAGTTGAAAGGCTGGCCACAGAATTGGAAAAATGGAAGGAAAAATGCAATGATTTGGAAACCAAAAACAATCAAAGGTCAAATAAAGAACATGAGAACAACACAGATGTGCTTGGAAAGCTCACTAATCTTCAAGATGAGTTACAGGAGTCTGAACAGAAATATAATGCTGATAGAAAGAAATGGTTAGAAGAAAAAATGATGCTTATCACTCAAGCGAAAGAAGCAGAGAATATACGAAATAAAGAGATGAAAAAATATGCTGAGGACAGGGAGCGTTTTTTTAAGCAACAGAATGAAATGGAAATACTGACAGCCCAGCTGACAGAGAAAGATAGTGACCTTCAAAAGTGGCGAGAAGAACGAGATCAACTGGTTGCAGCTTTAGAAATACAGCTAAAAGCACTGATATCCAGTAATGTACAGAAAGATAATGAAATTGAACAACTAAAAAGGATCATATCAGAGACTTCTAAAATAGAAACACAAATCATGGATATCAAGCCCAAACGTATTAGTTCAGCAGATCCTGACAAACTTCAAACTGAACCTCTATCGACAAGTTTTGAAATTTCCAGAAATAAAATAGAGGATGGATCTGTAGTCCTTGACTCTTGTGAAGTGTCAACAGAAAATGATCAAAGCACTCGATTTCCAAAACCTGAGTTAGAGATTCAATTTACACCTTTACAGCCAAACAAAATGGCAGTGAAACACCCTGGTTGTACCACACCAGTGACAGTTAAGATTCCCAAGGCTCGGAAGAGGAAGAGTAATGAAATGGAGGAG |
| hsa_circ_0019607 | AGCAACTTATGACTCTCATCTCTGCTGCACGAGAATATGAGATAGAGTTCATCTATGCGATCTCACCTGGATTGGATATCACTTTTTCTAACCCCAAGGAAGTATCCACATTGAAACGTAAATTGGACCAGGTTTCTCAGTTTGGGTGCAGATCATTTGCTTTGCTTTTTGATGATATAGACCATAATATGTGTGCAGCAGACAAAGAGGTATTCAGTTCTTTTGCTCATGCCCAAGTCTCCATCACAAATGAAATCTATCAGTACCTAGGAGAGCCAGAAACTTTCCTCTTCTGTCCCACAGAATACTGTGGCACTTTCTGTTATCCAAATGTGTCTCAGTCTCCATATTTAAGGACTGTGGGTGAAAAGCTTCTACCTGGAATTGAAGTGCTTTGGACAGGTCCCAAAGTTGTTTCTAAAGAAATTCCAGTAGAGTCCATCGAAGAGGTTTCTAAGATTATTAAGAGAGCTCCAGTAATCTGGGATAACATTCATGCTAATGATTATGATCAGAAGAGACTGTTTCTGGGCCCGTACAAAGGAAGATCCACAGAACTCATCCCACGGTTAAAAGGAGTCCTCACTAATCCAAATTGTGAATTTGAAGCCAACTACGTTGCTATCCACACCCTTGCCACCTGGTACAAATCAAACATGAATGGAGTGAGAAAAGATGTAGTGATGACTGACAGTGAAGATAGTACTGTGTCCATCCAGATAAAATTAGAAAATGAAGGCAGTGATGAAGATATTGAAACTGATGTACTCTATAGTCCACAGATGGCTCTAAAGCTAGCATTAACAGAATGGTTGCAAGAGTTTGGTGTGCCTCATCAATACAGCAGTAGGCAAGTTGCACACAGTGGAGCTAAAGCAAGTGTAGTTGATGGGACTCCTTTAGTTGCAGCACCCTCTTTAAATGCCACAACCGTAGTAACAACAGTTTATCAGGAGCCCATTATGAGCCAGGGAGCAGCCTTGAGTGGTGAGCCTACTACTCTGACCAAGGAAGAAGAAAAGAAACAGCCTGATGAAGAACCCATGGACATGGTGGTGGAAAAACAAGAAGAAACGGACCACAAGAATGACAATCAAATACTGAGTGAAATTGTTGAAGCGAAAATGGCAGAGGAATTGAAACCAATGGACACTGATAAAGAGAGCATAGCTGAATCAAAATCCCCAGAGATGTCCATGCAAGAAGATTGTATTAGTGACATTGCCCCCATGCAAACTGATGAACAGACAAACAAGGAGCAGTTTGTGCCAGGTCCAAATGAAAAGCCTTTGTACACTGCGGAACCAGTGACCCTGGAGGATTTGCAGTTACTTGCTGATCTATTCTACCTTCCTTACGAGCATGGACCCAAAGGAGCACAGATGTTACGGGAATTTCAATGGCTTCGAGCAAATAGTAGTGTTGTCAGTGTCAATTGCAAAGGAAAAGACTCTGAAAAAATTGAAGAATGGCGGTCACGAGCAGCCAAGTTTGAAGAGATGTGTGGACTAGTGATGGGAATGTTCACTCGGCTCTCCAATTGTGCCAACAGGACAATTCTTTATGACATGTACTCCTATGTTTGGGATATCAAGAGTATAATGTCTATGGTGAAGTCTTTTGTACAGTGGTTAGGGTGTCGTAGTCATTCTTCAGCACAATTCTTAATTGGAGACCAAGAACCCTGGGCCTTTAGAGGTGGTCTAGCAGGAGAGTTCCAGCGTTTGCTGCCAATTGATGGGGCAAATGATCTCTTTTTTCAGCCACCTCCACTGACTCCTACCTCCAAAGTTTATACTATCAGACCTTATTTTCCTAAGGATGAG |
| hsa_circ_0019799 | GTTGGACCGACAGTTACAAGACATAGTGTACAAATTAGTGATCAATCTAGAGGAAAGAGAAAAAAAGCAAATGCATGATTTCTATAAAGAAAGAGGTCTAGAAGTACCTAAACCTG |
| hsa_circ_0020151 | CTTTCAAATTCTGCCTCCAATACACGCGTAAGGCTGAATTCCGTAAACTGTGTGACAATTTGAGAATGCACTTATCGCAGATTCAGCGCCACCATAACCAAAGTACGGCAATCAATCTTAATAATCCAGAGAGCCAGTCCATGCATTTGGAAACCAGACTTGTTCAGCTGGACAGTGCTATCAGCATGGAATTGTGGCAGGAAGCATTCAAAGCTGTGGAAGATATTCACGGGCTATTCTCCTTGTCTAAAAAACCACCTAAACCTCAGTTGATGGCAAATTACTATAACAAAGTCTCAACTGTGTTTTGGAAATCTGGAAATGCTCTTTTTCATGCATCTACACTCCATCGTCTTTACCATCTCTCTAGAGAAATGAGAAAGAATCTCACACAAGATGAGATGCAAAG |
| hsa_circ_0022936 | GCTATTCAGCTTGACCCTGAAGAAACTCGTCATGCTAAAAGAAATGGACAAAGATCTTAACTCAGTGGTCATCGCTGTGAAGCTGCAGGGTTCAAAAAGAATTCTTCGCTCCAACGAGATCGTCCTTCCAGCTAGTGGACTGGTGGAAACAGAGCTCCAATTAACCTTCTCCCTTCAGTACCCTCATTTCCTTAAGCGAGATGCCAACAAGCTGCAGATCATGCTGCAAAGGAGAAAACGTTACAAGAATCGGACCATCTTGGGCTATAAGACCTTGGCCGTGGGACTCATCAACATGGCAGAG |
| hsa_circ_0023892 | TGATGTAAATTGGAGTCAACCAGGTGAAAAGAAGTTAACTGGGGGATCTAACTGGCAACCAAAGGTTGCACCAACAACCGCTTGGAATGCTGCAACAATGAATGGCATGCATTTTCCACAATACGCACCCCCTGTAATGGCCTATCCTGCTACTACACCAACAGGCATGATAGGATATGGAATT |
| hsa_circ_0024840 | GGAAAAAGTTGGATTGTTAAAAGAAGTTATGAAGATTTTCGGGTACTTGATAAACATCTTCATCTGTGTATTTATGACCGAAGATTTTCCCAGCTCTCAGAACTTCCCCGTTCTGACACCCTGAAGGACAGTCCAGAGTCGGTCACTCAGATGCTTATGGCTTACCTGTCACGCCTTTCAGCTATCGCTGGCAACAAGATCAACTGTGGGCCCGCCCTTACCTGGATGGAGATTGATAATAAGGGAAATCACCTTTTGGTTCATGAGGAGTCATCCATCAACACTCCTGCTGTCGGTGCTGCCCATGTTATCAAGAGGTACACTGCTCGGGCCCCTGACGAACTGACCTTAGAGGTGGGAGACATTGTTTCTGTTATTGACATGCCCCCGAAAGTGTTAAGCACATGGTGGAGAGGCAAGCACGGATTCCAG |
| hsa_circ_0024872 | TTCCTCCAACTCCTCTGCCAACCAATGATGTTGATGTGTATTTCGAGACCTCTGCAGATGATAATGAGCATGCTCGCTTCCAGAAGGCTAAGGAGCAGCTGGAGATTCGGCACCGCAACCGAATGGACAGGGTAAAGAAGGAATGGGAAGAGGCAGAGCTTCAAGCTAAGAACCTCCCCAAAGCAGAGAGGCAGACTCTGATTCAGCACTTCCAAGCCATGGTTAAAGCTTTAGAGAAGGAAGCAGCCAGTGAGAAGCAGCAGCTGGTGGAGACCCACCTGGCCCGAGTGGAAGCTATGCTGAATGACCGCCGTCGGATGGCTCTGGAGAACTACCTGGCTGCCTTGCAGTCTGACCCGCCACGGCCTCATCGCATTCTCCAGGCCTTACGGCGTTATGTCCGTGCTGAGAACAAAGATCGCTTACATACCATCCGTCATTACCAGCATGTGTTGGCTGTTGACCCAGAAAAGGCGGCCCAGATGAAATCCCAGGTGATGACACATCTCCACGTGATTGAAGAAAGGAGGAACCAAAGCCTCTCTCTGCTCTACAAAGTACCTTATGTAGCCCAAGAAATTCAAGAGGAAATTGATGAGCTCCTTCAGGAGCAGCGTGCAGATATGGACCAGTTCACTGCCTCAATCTCAGAGACCCCTGTGGACGTCCGGGTGAGCTCTGAGGAGAGTGAGGAGATCCCACCGTTCCACCCCTTCCACCCCTTCCCAGCCCTACCTGAGAACGAAG |
| hsa_circ_0024897 | TACGAGACAGCCCTTGACGGTGAAAACAGCAGCGGCCTGCAGCAGCTGGCCTACCACACTGTGAATCGTCGCTATCGGGAGTTCTTGAATCTGCAGACCCGTCTGGAGGAGAAACCAGATCTACGAAAGTTCATCAAAAATGTGAAGGGTCCTAAAAAGCTCTTTCCAGATCTTCCATTGGGAAACATGGACAGTGACAGAGTAGAAGCCCGTAAGAGCCTCCTAGAATCATTCCTAAAGCAACTCTGTGCCATTCCGGAGATCGCTAACAGTGAGGAGGTGCAGGAGTTCCTTGCTCTGAACACAGATGCTCGTATTGCCTTTGTCAAGAAACCATTTATGGTCTCTAGAATAGACAAGATGGTGGTGAGTGCCATTGTGGACACCTTGAAGACAGCGTTTCCTCGCTCTGAACCCCAGAGCCCCACAGAGGAGCTGAGTGAGGCCGAGACCGAAAGCAAGCCCCAGACAGAAGGCAAGAAGGCTAGCAAGTCTAGGCTGAGGTTCTCATCCAGTAAAATTTCTCCAGCACTAAGTGTGACTGAAGCACAAGACAAGATTCTTTATTGTCTCCAGGAAGGCAATGTGGAGTCTGAGACTCTATCCATGTCTGCGATGGAATCTTTTATTGAAAAACAGACAAAGTTACTGGAAATGCAGCCAACAAAAGCCCCAGAAAAAGATCCTGAACAACCTCCCAAAGGACGTGTGGACAGTTGCGTGTCAGATGCAGCCGTGCCAGCCCAAGACCCCAGCAACAGCGATCCAGGAACAGAGACAGAGTTAGCTGACACAGCCCTGGATCTGCTCCTCTTGCTACTAACAGAACAGTGGAAATGGCTATGTACCGAAAACATGCAAAAGTTTCTTCGTCTTATCTTTGGGACCCTAGTTCAAAG |
| hsa_circ_0024961 | GTACCCCAGAGTTCATGGCCCCTGAGATGTATGAGGAGAAATATGATGAATCCGTTGACGTTTATGCTTTTGGGATGTGCATGCTTGAGATGGCTACATCTGAATATCCTTACTCGGAGTGCCAAAATGCTGCACAGATCTACCGTCGCGTGACCAGTGGGGTGAAGCCAGCCAGTTTTGACAAAGTAGCAATTCCTGAAGTGAAGGAAATTATTGAAGGATGCATACGACAAAACAAAGATGAAAGATATTCCATCAAAGACCTTTTGAACCATGCCTTCTTCCAAGAGGAAACAGGAGTACGGGTAGAATTAGCAGAAGAAGATGATGGAGAAAAAATAGCCATAAAATTATGGCTACGTATTGAAGATATTAAGAAATTAAAGGGAAAATACAAAGATAATGAAGCTATTGAGTTTTCTTTTGATTTAGAGAGAGATGTCCCAGAAGATGTTGCACAAGAAATGGTAGAGTCTGGGTATGTCTGTGAAGGTGATCACAAGACCATGGCTAAAGCTATCAAAGACAGAGTATCATTAATTAAGAGGAAACGAGAGCAGCGGCAGTTGGTACGGGAGGAGCAAGAAAAAAAAAAGCAGGAAGAGAGCAGTCTCAAACAGCAGGTAGAACAATCCAGTGCTTCCCAGACAGGAATCAAGCAGCTCCCTTCTGCTAGCACCGGCATACCTACTGCTTCTACCACTTCAGCTTCAGTTTCTACACAAGTAGAACCTGAAGAACCTGAGGCAGATCAACATCAACAACTACAGTACCAGCAACCCAGTATATCTGTGTTATCTGATGGGACGGTTGACAGTGGTCAGGGATCCTCTGTCTTCACAGAATCTCGAGTGAGCAGCCAACAGACAGTTTCATATGGTTCCCAACATGAACAGGCACATTCTACAGGCACAGTCCCAGGGCATATACCTTCTACTGTCCAAGCACAGTCTCAGCCCCATGGGGTATATCCACCCTCAAGTGTG |
| hsa_circ_0025048 | GTCGGAACGAGCTGATTGCCCGCTACATCAAGCTCCGGACAGGGAAGACCCGCACCAGGAAGCAGGTCTCCAGCCACATCCAGGTGCTGGCTCGTCGCAAAGCTCGCGAGATCCAGGCCAAGCTAAAGGACCAGGCAGCTAAGGACAAGGCCCTGCAGAGCATGGCTGCCATGTCGTCTGCACAGATCATCTCCGCCACGGCCTTCCACAGTAGCATGGCCCTCGCCCGGGGCCCCGGCCGCCCAGCAGTCTCAGGGTTTTGGCAAGGAGCTTTGCCAGGCCAAGCCGGAACGTCCCATGATGTGAAGCCTTTCTCTCAGCAAACCTATGCTGTCCAGCCTCCGCTGCCTCTGCCAGGGTTTGAGTCTCCTGCAGGGCCCGCCCCATCGCCCTCTGCGCCCCCGGCACCCCCATGGCAGGGCCGCAGCGTGGCCAGCTCCAAGCTCTGGATGTTGGAGTTCTCTGCCTTCCTGGAGCAGCAGCAGGACCCGGACACGTACAACAAGCACCTGTTCGTGCACATTGGCCAGTCCAGCCCAAGCTACAGCGACCCCTACCTCGAAGCCGTGGACATCCGCCAAATCTATGACAAATTCCCGGAGAAAAAGGGTGGACTCAAGGATCTCTTCGAACGGGGACCCTCCAATGCCTTTTTTCTTGTGAAGTTCTGG |
| hsa_circ_0025359 | AACCCAGAAGTACCATGGCTTCTGACCTAGAGAGTAGCCTCACCTCCATAGACTGGCTCCCCCAGCTGACCCTCCGAGCTACCATTGAGAAGCTTGGAAGTGCCTCCCAGGCTGGGCCTCCCGGGAGCAGCCGCAAGTGTTCACCAGGGTCACCCACAGATCCTAATGCCACCCTGAGCAAAGACGAGGCAGCAGTGCACCAGGACGGCAAGCCACGATACAGCTATGCCACTCTCATCACCTATGCCATCAACTCCTCTCCAGCCAAGAAGATGACCCTCAGCGAGATTTACCGCTGGATCTGTGATAACTTCCCCTATTACAAGAATGCTGGCATTGGTTGGAAG |
| hsa_circ_0025873 | TTGGCACTTGGCAATGTAATAAGTGCCTTGGGAGACAAGAGCAAGAGGGCCACACATGTCCCCTATAGAGATTCCAAGCTAACAAGACTACTACAGGATTCCCTCGGGGGTAATAGCCAAACAATCATGATAGCATGTGTCAGCCCTTCAGACAGAGACTTTATGGAAACGTTAAACACCCTGAAATACGCCAATCGAGCTAGAAATATCAAGAATAAGGTGATGGTCAATCAGGACAGAGCTAGTCAGCAAATCAATGCACTTCGTAGTGAAATCACACGACTTCAGATGGAGCTCATGGAGTACAAAACAGGTAAAAGAATAATTGACGAAGAGGGTGTGGAAAGCATCAATGACATGTTTCATGAGAATGCTATGCTACAGACTGAAAATAATAACCTGCGTGTAAGAATTAAAGCCATGCAAGAGACGGTTGATGCATTGAGGTCCAGAATTACACAGCTTGTTAGTGATCAGGCCAACCATGTTCTTGCCAGAGCAGGTGAAGGAAATGAGGAGATTAGTAATATGATTCATAGTTATATAAAAGAAATCGAAGATCTCAG |
| hsa_circ_0025933 | ACATCGATGAGTGTGCTGAAGGGCGCCATTACTGTCGTGAAAATACAATGTGTGTCAACACCCCGGGTTCTTTTATGTGCATCTGCAAAACTGGATACATCAGAATTGATGATTATTCATGTACAGAACATGATGAGTGTATCACAAATCAGCACAACTGTGATGAAAATGCTTTATGCTTCAACACTGTTGGAGGACACAACTGTGTTTGCAAGCCGGGCTATACAGGGAATGGAACGACATGCAAAG |
| hsa_circ_0026743 | GCTCAGATCTTGCTGGACTGTGGAGAAGACAACATCTGTGTGCCTGACCTGCAGCTGGAAGTGTTTGGGGAGCAGAACCATGTGTACCTGGGTGACAAGAATGCCCTGAACCTCACTTTCCATGCCCAGAATGTGGGTGAGGGTGGCGCCTATGAGGCTGAGCTTCGGGTCACCGCCCCTCCAGAGGCTGAGTACTCAGGACTCGTCAGACACCCAGGGAACTTCTCCAGCCTGAGCTGTGACTACTTTGCCGTGAACCAGAGCCGCCTGCTGGTGTGTGACCTGGGCAACCCCATGAAGGCAGGAGCCAGTCTGTGGGGTGGCCTTCGGTTTACAGTCCCTCATCTCCGGGACACTAAGAAAACCATCCAGTTTGACTTCCAGATCCTCAGCAAGAATCTCAACAACTCGCAAAGCGACGTGGTTTCCTTTCGGCTCTCCGTGGAGGCTCAGGCCCAGGTCACCCTGAACGGTGTCTCCAAGCCTGAGGCAGTGCTATTCCCAGTAAGCGACTGGCATCCCCGAGACCAGCCTCAGAAGGAGGAGGACCTGGGACCTGCTGTCCACCATGTCTATGAGCTCATCAACCAAGGCCCCAGCTCCATTAGCCAGGGTGTGCTGGAACTCAGCTGTCCCCAGGCTCTGGAAGGTCAGCAGCTCCTATATGTGACCAGAGTTACGGGACTCAACTGCACCACCAATCACCCCATTAACCCAAAGGGCCTGGAGTTGGATCCCGAGGGTTCCCTGCACCACCAGCAAAAACGGGAAGCTCCAAGCCGCAGCTCTGCTTCCTCGGGACCTCAGATCCTGAAATGCCCGGAGGCTGAGTGTTTCAGGCTGCGCTGTGAGCTCGGGCCCCTGCACCAACAAGAGAGCCAAAGTCTGCAGTTGCATTTCCGAGTCTGGGCCAAGACTTTCTTGCAGCGGGAGCACCAGCCATTTAGCCTGCAGTGTGAGGCTGTGTACAAAGCCCTGAAGATGCCCTACCGAATCCTGCCTCGGCAGCTGCCCCAAAAAGAGCGTCAGGTGGCCACAGCTGTGCAATGGACCAAGGCAGAAGGCAGCTATGGCGTCCCACTGTGGATCATCATCCTAGCCATCCTGTTTGGCCTCCTGCTCCTAGGTCTACTCATCTACATCCTCTACAAG |
| hsa_circ_0027214 | AGTACCCGACCTGCGGCCCCAGTGAGTTCCGCTGTGCCAATGGGCGCTGTCTGAGCTCCCGCCAGTGGGAGTGTGATGGCGAGAATGACTGCCACGACCAGAGTGACGAGGCTCCCAAGAACCCACACTGCACCAGCCAAGAGCACAAGTGCAATGCCTCGTCACAGTTCCTGTGCAGCAGTGGGCGCTGTGTGGCTGAGGCACTGCTCTGCAACGGCCAGGATGACTGTGGCGACAGCTCGGACGAGCGTGGCTGCCACATCAATGAGTGTCTCAGCCGCAAGCTCAGTGGCTGCAGCCAGGACTGTGAGGACCTCAAGATCGGCTTCAAGTGCCGCTGTCGCCCTGGCTTCCGGCTGAAGGACGACGGCCGGACGTGTGCTGATGTGGACGAGTGCAGCACCACCTTCCCCTGCAGCCAGCGCTGCATCAACACTCATGGCAGCTATAAGTGTCTGTGTGTGGAGGGCTATGCACCCCGCGGCGGCGACCCCCACAGCTGCAAGGCTGTGACTGACGAGGAACCGTTTCTGATCTTCGCCAACCGGTACTACCTGCGCAAGCTCAACCTGGACGGGTCCAACTACACGTTACTTAAGCAGGGCCTGAACAACGCCGTTGCCTTGGATTTTGACTACCGAGAGCAGATGATCTACTGGACAGATGTGACCACCCAGGGCAGCATGATCCGAAGGATGCACCTTAACGGGAGCAATGTGCAGGTCCTACACCGTACAGGCCTCAGCAACCCCGATGGGCTGGCTGTGGACTGGGTGGGTGGCAACCTGTACTGGTGCGACAAAGGCCGGGACACCATCGAGGTGTCCAAGCTCAATGGGGCCTATCGGACGGTGCTGGTCAGCTCTGGCCTCCGTGAGCCCAGGGCTCTGGTGGTGGATGTGCAGAATGGGTACCTGTACTGGACAGACTGGGGTGACCATTCACTGATCGGCCGCATCGGCATGGATGGGTCCAGCCGCAGCGTCATCGTGGACACCAAGATCACATGGCCCAATGGCCTGACGCTGGACTATGTCACTGAGCGCATCTACTGGGCCGACGCCCGCGAGGACTACATTGAATTTGCCAGCCTGGATGGCTCCAATCGCCACGTTGTGCTGAGCCAGGACATCCCGCACATCTTTGCACTGACCCTGTTTGAGGACTACGTCTACTGGACCGACTGGGAAACAAAGTCCATTAACCGAGCCCACAAGACCACGGGCACCAACAAAACGCTCCTCATCAGCACGCTGCACCGGCCCATGGACCTGCATGTCTTCCATGCCCTGCGCCAGCCAGACGTGCCCAATCACCCCTGCAAGGTCAACAATGGTGGCTGCAGCAACCTGTGCCTGCTGTCCCCCGGGGGAGGGCACAAATGTGCCTGCCCCACCAACTTCTACCTGGGCAGCGATGGGCGCACCTGTGTGTCCAACTGCACGGCTAGCCAGTTTGTATGCAAGAACGACAAGTGCATCCCCTTCTGGTGGAAGTGTGACACCGAGGACGACTGCGGGGACCACTCAGACGAGCCCCCGGACTGCCCTGAGTTCAAGTGCCGGCCCGGACAGTTCCAGTGCTCCACAGGTATCTGCACAAACCCTGCCTTCATCTGCGATGGCGACAATGACTGCCAGGACAACAGTGACGAGGCCAACTGTGACATCCACGTCTGCTTGCCCAGTCAGTTCAAATGCACCAACACCAACCGCTGTATTCCCGGCATCTTCCGCTGCAATGGGCAGGACAACTGCGGAGATGGGGAGGATGAGAGGGACTGCCCCGAGGTGACCTGCGCCCCCAACCAGTTCCAGTGCTCCATTACCAAACGGTGCATCCCCCGGGTCTGGGTCTGCGACCGGGACAATGACTGTGTGGATGGCAGTGATGAGCCCGCCAACTGCACCCAGATGACCTGTGGTGTGGACGAGTTCCGCTGCAAGGATTCGGGCCGCTGCATCCCAGCGCGTTGGAAGTGTGACGGAGAGGATGACTGTGGGGATGGCTCGGATGAGCCCAAGGAAGAGTGTGATGAACGCACCTGTGAGCCATACCAGTTCCGCTGCAAGAACAACCGCTGCGTGCCCGGCCGCTGGCAGTGCGACTACGACAACGATTGCGGTGACAACTCCGATGAAGAGAGCTGCACCCCTCGGCCCTGCTCCGAGAGTGAGTTCTCCTGTGCCAACGGCCGCTGCATCGCGGGGCGCTGGAAATGCGATGGAGACCACGACTGCGCGGACGGCTCGGACGAGAAAGACTGCACCCCCCGCTGTGACATGGACCAGTTCCAGTGCAAGAGCGGCCACTGCATCCCCCTGCGCTGGCGCTGTGACGCAGACGCCGACTGCATGGACGGCAGCGACGAGGAGGCCTGCGGCACTGGCGTGCGGACCTGCCCCCTGGACGAGTTCCAGTGCAACAACACCTTGTGCAAGCCGCTGGCCTGGAAGTGCGATGGCGAGGATGACTGTGGGGACAACTCAGATGAGAACCCCGAGGAGTGTG |
| hsa_circ_0027317 | CTGGTGAGCTTGTTGCTCATTGGAGTGGCTGCTTGGGGCAAGGGCCTGGGTCTGGTGTCCAGCATCCACATCATCGGCGGAGTCATTGCTGTGGGAGTCTTCCTTCTCCTTATTGCAGTGGCTGGACTGGTGGGTGCTGTCAACCACCACCAAGTCCTGCTGTTCTTTTACATGATCATCCTTGGTTTGGTCTTCATCTTCCAATTTGTAATCTCTTGCTCATGTCTGGCTATTAACCGAAGCAAACAG |
| hsa_circ_0027337 | ATCCCAGGGACCTGCCTGCTCCCAGAGGTGACAGAGGAAGATCAAGGAAGGATCTGTGTGGTCATTGACCTCGATGAAACCCTTGTGCATAGCTCCTTTAAGCCAATCAACAATGCTGACTTCATAGTGCCTATAGAGATTGAGGGGACCACTCACCAG |
| hsa_circ_0027463 | ACTGTACAATTTGTTCAAGGAATTTTTGTAGAAAAATACGATCCTACGATAGAAGATTCTTATAGAAAGCAAGTTGAAGTAGATGCACAACAGTGTATGCTTGAAATCTTGGATACTGCAGGAACG |
| hsa_circ_0027493 | AATCATCGGACTCAGGTACATCTGTGAGTGAGAACAGGTGTCACCTTGAAGGTGGGAGTGATCAAAAGGACCTTGTACAAGAGCTTCAGGAAGAGAAACCTTCATCTTCACATTTGGTTTCTAGACCATCTACCTCATCTAGAAGGAGAGCAATTAGTGAGACAG |
| hsa_circ_0028299 | GCTCGACAACCTGGTGTTTCATCCAGAAGAGCCAGAGGTGCTTGCTGTCCTTGACTGGGAACTTTCTACCTTGGGCGACCCCCTTGCTGATGTGGCCTACAGCTGCCTGGCTCATTACCTGCCATCCAGTTTTCCCGTGCTGAGAG |
| hsa_circ_0029340 | GTTGACTTCTGGCATTCCGATCAGTGCAACATGATCAATGGAACTTCTGGGCAAATGTGGCCGCCCTTCATGACTCCTGAGTCCTCGCTGGAGTTCTACAGCCCGGAGGCCTGCCGATCCATGAAGCTAATGTACAAGGAGTCAGGGGTGTTTGAAGGCATCCCCACCTATCGCTTCGTGGCTCCCAAAACCCTGTTTGCCAACGGGTCCATCTACCCACCCAACGAAGGCTTCTGCCCGTGCCTGGAGTCTGGAATTCAGAACGTCAGCACCTGCAGGTTCA |
| hsa_circ_0030838 | GGGACCATGGCTTTCCGGGCTCCTCAGGACCCAGGGGAGACCCTGGCTTGAAAGGTGATAAGGGGGATGTCGGTCTCCCTGGCAAGCCTGGCTCCATGGATAAGGTGGACATGGGCAGCATGAAGGGCCAGAAAGGAGACCAAGGAGAGAAAGGACAAATTGGACCAATTGGTGAGAAGGGATCCCGAGGAGACCCTGGGACCCCAGGAGTGCCTGGAAAGGACGGGCAGGCAGGACAGCCTGGGCAGCCAGGACCTAAAGGTGATCCAGGTATAAGTGGAACCCCAGGTGCTCCAGGACTTCCGGGACCAAAAGGATCTGTTGGTGGAATGGGCTTGCCAGGAACACCTGGAGAGAAAGGTGTGCCTGGCATCCCTGGCCCACAAGGTTCACCTGGCTTACCTGGAGACAAAGGTGCAAAAGGAGAGAAAGGGCAGGCAGGCCCACCTGGCATAGGCATCCCAGGGCTGCGAGGTGAAAAGGGAGATCAAGGGATAGCGGGTTTCCCAGGAAGCCCTGGAGAGAAGGGAGAAAAAGGAAGCATTGGGATCCCAGGAATGCCAGGGTCCCCAGGCCTTAAAGGGTCTCCCGGGAGTGTTGGCTATCCAGGAAGTCCTGGGCTACCTGGAGAAAAAGGTGACAAAGGCCTCCCAGGATTGGATGGCATCCCTGGTGTCAAAGGAGAAGCAGGTCTTCCTGGGACTCCTGGCCCCACAGGCCCAGCTGGCCAGAAAGGGGAGCCAGGCAGTGATGGAATCCCGGGGTCAGCAGGAGAGAAGGGTGAACCAGGTCTACCAGGAAGAGGATTCCCAGGGTTTCCAGGGGCCAAAGGAGACAAAGGTTCAAAGGGTGAGGTGGGTTTCCCAGGATTAGCCGGGAGCCCAGGAATTCCTGGATCCAAAGGAGAGCAAGGATTCATGGGTCCTCCGGGGCCCCAGGGACAGCCGGGGTTACCGGGATCCCCAGGCCATGCCACGGAGGGGCCCAAAGGAGACCGCGGACCTCAGGGCCAGCCTGGCCTGCCAGGACTTCCGGGACCCATGGGGCCTCCAGGGCTTCCTGGGATTGATGGAGTTAAAGGTGACAAAGGAAATCCAGGCTGGCCAGGAGCACCCGGTGTCCCAGGGCCCAAGGGAGACCCTGGATTCCAGGGCATGCCTGGTATTGGTGGCTCTCCAGGAATCACAGGCTCTAAGGGTGATATGGGGCCTCCAGGAGTTCCAGGATTTCAAGGTCCAAAAGGTCTTCCTGGCCTCCAGGGAATTAAAGGTGATCAAGGCGATCAAGGCGTCCCGGGAGCTAAAGGTCTCCCGGGTCCTCCTGGCCCCCCAGGTCCTTACGACATCATCAAAGGGGAGCCCGGGCTCCCTGGTCCTGAGGGCCCCCCAGGGCTGAAAGGGCTTCAGGGACTGCCAGGCCCGAAAGGCCAGCAAGGTGTTACAGGATTGGTGGGTATACCTGGACCTCCAGGTATTCCTGGGTTTGACGGTGCCCCTGGCCAGAAAGGAGAGATGGGACCTGCCGGGCCTACTGGTCCAAGAGGATTTCCAGGTCCACCAGGCCCCGATGGGTTGCCAGGATCCATGGGGCCCCCAGGCACCCCATCTGTTGATCACGGCTTCCTTGTGACCAGGCATAGTCAAACAATAGATGACCCACAGTGTCCTTCTGGGACCAAAATTCTTTACCACGGGTACTCTTTGCTCTACGTGCAAGGCAATGAACGGGCCCATGGCCAGGACTTGG |
| hsa_circ_0030849 | GGAATTCCTGGCCAAGACGGCCCGCCAGGCCCCCCAGGTATTCCAGGATGCAATGGCACAAAGGGGGAGAGAGGGCCGCTCGGGCCTCCTGGCTTGCCTGGTTTCGCTGGAAATCCCGGACCACCAGGCTTACCAGGGATGAAGGGTGATCCAGGTGAGATACTTGGCCATGTGCCCGGGATGCTGTTGAAAGGTGAAAGAGGATTTCCCGGAATCCCAGGGACTCCAGGCCCACCAGGACTGCCAGGGCTTCAAGGTCCTGTTGGGCCTCCAGGATTTACCGGACCACCAGGTCCCCCAGGCCCTCCCGGCCCTCCAGGTGAAAAGGGACAAATGGGCTTAAGTTTTCAAGGACCAAAAGGTGACAAGGGTGACCAAGGGGTCAGTGGGCCTCCAGGAGTACCAGGACAAGCTCAAGTTCAAGAAAAAGGAGACTTCGCCACCAAGGGAGAAAAGGGCCAAAAAGGTGAACCTGGATTTCAGGGGATGCCAGGGGTCGGAGAGAAAGGTGAACCCGGAAAACCAGGACCCAGAGGCAAACCCGGAAAAGATGGTGACAAAGGGGAAAAAGGGAGTCCCGGTTTTCCTGGTGAACCCGGGTACCCAGGACTCATAGGCCGCCAGGGCCCGCAGGGAGAAAAGGGTGAAGCAGGTCCTCCTGGCCCACCTGGAATTGTTATAGGCACAGGACCTTTGGGAGAAAAAGGAGAGAGGGGCTACCCTGGAACTCCGGGGCCAAGAGGAGAGCCAGGCCCAAAAGGTTTCCCAGGACTACCAGGCCAACCCGGACCTCCAGGCCTCCCTGTACCTGGGCAGGCTGGTGCCCCTGGCTTCCCTGGTGAAAGAGGAGAAAAAGGTGACCGAGGATTTCCTGGTACATCTCTGCCAGGACCAAGTGGAAGAGATGGGCTCCCGGGTCCTCCTGGTTCCCCTGGGCCCCCTGGGCAGCCTGGCTACACAAATGGAATTGTGGAATGTCAGCCCGGACCTCCAGGTGACCAGGGTCCTCCTGGAATTCCAGGGCAGCCAGGATTTATAGGCGAAATTGGAGAGAAAGGTCAAAAAGGAGAGAGTTGCCTCATCTGTGATATAGACGGATATCGGGGGCCTCCCGGGCCACAGGGACCCCCGGGAGAAATAGGTTTCCCAGGGCAGCCAGGGGCCAAGGGCGACAGAGGTTTGCCTGGCAGAGATGGTGTTGCAGGAGTGCCA |
| hsa_circ_0032155 | ACGTTCCATCCTCAGAGCAGCCTGAACTGTTCCTAAAGAAACTTCAGCAGTGCTGTGTCATTTTTGACTTCATGGACACGCTATCTGATCTTAAAATGAAAGAATACAAGCGCTCCACTCTTAATGAACTGGTGGACTACATTACAATAAGCAGAGGCTGTTTGACAGAGCAGACTTACCCTGAAGTAGTTAGAATG |
| hsa_circ_0032269 | GTGTTACTTTGCCAGTATTTGAACATTACCATGAAGGAACTGACAGTTATGAACTGACTGGTTTAGCCAGAGGTGGGGAACAGTTGGCTAAATTAAAGAGGAATTATGCCAAAGCAGTGGAACTACTGGTGGAACTAGCTTCTCTGCAGACTTCTTTTGTTACTTTGGATGAAGCTATTAAGATAACCAACAGGCGTGTAAATGCCATTGAACATGTCATCATTCCCCGGATTGAACGTACTCTTGCTTATATCATCACAGAGCTGGATGAGAGAGAGCGAGAAGAGTTCTATAG |
| hsa_circ_0032352 | GTGAATCAGCTCCCGGCCGACTTTAGGATTCTTCTGGATTTTAAATTTTTTCTTTTTAAAAAAACTTGGACGGATAAAAGATGTGCCATGGCAGGATAGCACCAAAGAGCACCTCAGTGTTTGCCGTGGCCTCCGTGGGACATGGAGTGTTCCTTCCGCTAGTGATCCTTTGCACCCTGCTTGGAGACGGACTTGCTTCCG |
| hsa_circ_0032940 | CTAATTTGACAGGACATGCTGAGAAGGTGGGAATAGAAAATTTTGAGCTCCTGAAGGTCCTAGGAACTGGAGCTTATGGAAAAGTATTTCTAGTTCGTAAAATAAGTGGCCATGATACTGGAAAGCTGTATGCCATGAAAGTTTTGAAAAAGGCAACAATCGTTCAAAAGGCCAAAACCACAGAGCATACAAGGACAGAACGACAAGTCCTGGAACACATTAGGCAGTCGCCATTTTTGGTAACATTACATTATGCTTTCCAGACAGAAACCAAACTTCATCTCATTTTAGATTATATAAATGGTGGTGAACTTTTTACTCATCTTTCTCAAAGAGAGCGTTTCACAGAGCATGAGGTGCAGATTTATGTTGGAGAGATTGTGCTTGCCCTCGAACATCTCCACAAGTTGGGGATTATATATCGTGATATTAAGCTTGAGAATATTCTACTTGATTCTAATGGCCATGTGGTGCTGACAGATTTTGGTCTGAGTAAGGAGTTTGTGGCTGATGAA |
| hsa_circ_0033126 | GATGAAACAAGTGAAGATGCTAACTGTCTTGCTTTGAGTGGACATGATAAAACAGAAGCAAAGGAACAACTTGATACAGAGACAAGTACAACTCAATCAGAAACTATTCAGACAGCGGCTTCTCTGTTGGCCTCTCAG |
| hsa_circ_0034802 | TTGATGGAGAACCTTGTGACCTGTCTTTGAATATAACCTGGTATCTGAAAAGCGCTGATTGTTACAATGAAATCTATAACTTCAAGGCAGAAGAAGTAGAGTTGTATTTGGAAAAACTTAAGGAAAAAAGAGGCTTGTCTGGGAAATATCAAACATCATCAAAATTGTTCCAGAACTGCAGTGAACTCTTTAAAACACAGACCTTTTCTGGAGATTTTATGCATCGACTGCCTCTTTTAGGAGAAAAACAGGAGGCTAAGGAGAATGGAACAAACCTTACCTTTATTGGAGACAAAACCGCAATGCATGAACCATTGCAAACTTGGCAAGATGCACCATACATTTTTATTGTACATATTGGCATTTCATCCTCAAAGGAATCATCAAAAGAAAATTCACTGAGTAATCTTTTTACCATGACTGTTGAAGTGAAGGGTCCCTATGAATACCTCACACTTGAAGACTATCCCTTGATGATT |
| hsa_circ_0035150 | ATATAAATGAATGTGCACTAGATCCTGATATTTGCCCAAATGGAATCTGTGAAAACCTTCGTGGGACCTATAAATGTATATGCAATTCAGGATATGAAGTGGATTCAACTGGGAAAAACTGCGTTGATATTAATGAATGTGTACTGAACAGTCTCCTTTGTGACAATGGACAATGTAGAAATACTCCTGGAAGTTTTGTCTGTACCTGCCCCAAGGGATTTATCTACAAACCTGATCTAAAAACATGTGAAGACATTGATGAATGCGAATCAAGTCCTTGCATTAATGGAGTCTGCAAGAACAGCCCAGGCTCTTTTATTTGTGAATGTTCTTCTGAAAGTACTTTGGATCCAACAAAAACCATCTGCATAGAAACCATCAAGGGCACTTGCTGGCAGACTGTCATTGATGGGCGATGTGAGATCAACATCAATGGAGCCACCTTAAAGTCCCAGTGCTGCTCCTCCCTCGGTGCTGCGTGGGGAAGCCCGTGCACCCTATGCCAAGTTGATCCCATATGTGGTAAAGGGTACTCAAGAATTAAAGGAACACAATGTGAAGATATAGATGAATGTGAAGTGTTCCCAGGAGTGTGTAAAAATGGCCTGTGTGTTAACACTAGGGGGTCATTCAAGTGTCAGTGTCCCAGTGGAATGACTTTGGATGCCACAGGAAGGATCTGTCTTGATATCCGCCTGGAAACCTGCTTCCTGAGGTACGAGGACGAGGAGTGCACCCTGCCTATTGCTGGCCGCCACCGCATGGACGCCTGCTGCTGCTCCGTCGGGGCAGCCTGGGGTACTGAGGAATGCGAGGAGTGTCCCATGAGAAATACTCCTGAGTACGAGGAGCTGTGTCCGAGAGGACCCGGATTTGCCACAAAAGAAATTACAAATGGAAAGCCTTTCTTCAAAGATATCAATGAGTGCAAGATGATACCCAGCCTCTGCACCCACGGCAAGTGCAGAAACACCATTGGCAGCTTTAAGTGCAGGTGTGACAGCGGCTTTGCTCTTGATTCTGAAGAAAGGAACTGCACAGACATTGACGAATGCCGCATATCTCCTGACCTCTGTGGCAGAGGCCAGTGTGTGAACACCCCTGGGGACTTTGAATGCAAGTGTGACGAAGGCTATGAAAGTGGATTCATGATGATGAAGAACTGCATGGATATTGATGAGTGTCAGAGAGATCCTCTCCTATGCCGAGGTGGTGTTTGCCATAACACAGAGGGAAGTTACCGCTGTGAATGCCCGCCTGGCCATCAGCTGTCCCCCAACATCTCCGCGTGTATCGACATCAATGAATGTGAGCTGAGTGCACACCTGTGCCCCAATGGCCGTTGCGTGAACCTCATAGGGAAGTATCAGTGTGCCTGCAACCCTGGCTACCATTCAACTCCCGATAGGCTATTTTGTGTTGACATTGATGAATGCAGCATAATGAATGGTGGTTGTGAAACCTTCTGCACAAACTCTGAAGGCAGCTATGAATGTAGCTGTCAGCCGGGATTTGCACTAATGCCTGACCAGAGATCATGCACCGACATCGATGAGTGTGAAGATAATCCCAATATCTGTGATGGTGGTCAGTGCACAAATATCCCTGGAGAGTACAGGTGCTTGTGTTATGATGGATTCATGGCATCTGAAGACATGAAGACTTGTGTAGATGTCAATGAGTGTGACCTGAATCCAAATATCTGCCTAAGTGGGACCTGTGAAAACACGAAAGGCTCATTTATCTGCCACTGTGATATGGGCTACTCCGGCAAAAAAGGAAAAACTGGCTGTACAGACATCAATGAATGTGAAATTGGAGCACACAACTGTGGCAAACATGCTGTATGTACCAATACAGCAGGAAGCTTCAAATGTAGCTGCAGTCCCGGGTGGATTGGAGATGGCATTAAGTGCACTGATCTGGACGAATGTTCCAATGGAACCCATATGTGCAGCCAGCATGCAGACTGCAAGAATACCATGGGATCTTACCGCTGTCTGTGCAAGGAAGGATACACAGGTGATGGCTTCACTTGTACAGACCTTGATGAGTGCTCTGAGAACCTGAATCTCTGTGGCAATGGCCAGTGCCTCAATGCACCAGGAGGATACCGCTGTGAATGCGACATGGGCTTCGTGCCCAGTGCTGACGGGAAAGCCTGTGAAG |
| hsa_circ_0036865 | ATTCAGTCCCTGTCAGGACGCCCTCGATCCTGTGATGTTGGAGGTGGCAATGCTTTTCCACATAATGGTCAAAACCTAGGCCTCTCACCCTTCTTGGGGACCTTGAACACTGGAGGGTCATTGCCAGATCTAACCAACCTCCACTACTCGACACCCCTGCCAGCCTCCCTGGACACCACCGACCACCACTTTGGCAGTATGAGTGTGGGGAATAGTGTGAACAACATCCCAGCTGCTATGACCCACCTGGGTATAAGAAGCTCCTCTGGTCTCCAGAGTTCTCGGAGTAACCCCTCCATCCAAGCCACGCTCAATAAGACTGTGCTTTCCTCTTCCTTAAATAACCACCCACAGACATCTGTTCCCAACGCATCTGCTCTTCACCCTTCGCTCCGTCTGTTTTCCCTTAGCAACCCATCTCTTTCCACCACAAACCTGAGCGGCCCGTCTCGGCGTCGGCAGCCTCCCGTCAGCCCTCTCACGCTTTCTCCTGGCCCTGAAGCACATCAAGGTTTCAGCAGACAGCTGTCTTCAACCAGCCCACTGGCCCCATATCCTACCTCCCAG |
| hsa_circ_0037173 | GGTCTTTGTCCCCGATTACACGCGGGAGCTTCTGCTGGAGCTGCGGGACTGCGTGTCCAATGGGAGCCTGGGCTGCCCCGTGCGTCTCACCGTGGGCCCGGTCACCCTGCCTAGCAACTTCCAGAAGGTGCTCACCTGCACCGGTGCCCCCTGGCCCTGCCGCCTGCTGCTGCCCTCACCGCCCTGGGACCGGTGGCTGCAAGTGACAGCTGAGAGCCTGGTGGGGCCCCTCGGGACAGTGGCTTTCAGTGCTGTAGCTGCCCTCACAGCTTGCAGGCCACGGAGCGTGACCATCCAGCCCCTTCTGCAGAGCAGCCAAAACCAGAGCTTCAATGCCTCCTCTGGTCTGCTGTCCCCGAGCCCCGACCACCAGGACCTGGGCAGGAGTGGCAGGGTGGACCGCAGCCCCTTCTGCCTCACAAACTACCCAGTCACGCGGGAGGACATGGACGTGGTGTCGGTGCACTTCCAGCCCCTGGACAGGGTCTCGGTGAGGGTGTGTTCGGACACGCCCTCCGTGATGCGGCTGCGCCTGAACACCGGCATGGACAGCGGGGGTTCCCTCACCATCTCCCTGCGGGCCAACAAGACAGAGATGCGGAACGAGACCGTCGTAGTGGCCTGCGTGAATGCTGCCTCGCCCTTCCTTGGCTTCAATACTTCGCTCAACTGCACCACAGCCTTCTTCCAGGGCTACCCTTTGTCTCTGAGCGCCTGGTCTCGCAGGGCCAACCTCATCATCCCCTACCCAGAGACAGACAACTGGTACCTCTCCCTGCAGCTCATGTGCCCTGAGAATGCTGA |
| hsa_circ_0037895 | CACCTGTGGAATCCTCTCAAGAGGAACAGTCATTGTGTGAAGGTTCAAATTCAGCTGTTAGCATGGAACTTTCAGAACCTATTGTAGAAAATGGAGAGACAGAAATGTCTCCAGAAGAATCATGGGAGCACAAAGAAGAAATAAGTGAAGCAGAGCCAGGGGGTGGTTCCTTGGGAGATGGAAGGCCGCCAGAGGAAAGTGCCCATGAAATGATGGAGGAGGAAGAGGAAATCCCAAAACCTAAGTCTGTGGTTGCACCGCCAGGTGCTCCTAAGAAAGAGCATGTAAATGTAGTATTCATTGGGCACGTAGATGCTGGCAAGTCAACCATTGGAGGACAAATAATGTATTTGACTGGAATGGTTGACAAAAGGACGCTTGAAAAGTATGAAAGAGAAGCTAAAGAGAAAAACAGAGAAACTTGGTACTTGTCTTGGGCCTTAGACACAAATCAGGAAGAACGAGACAAGGGTAAAACAGTAGAAGTGGGTCGTGCCTATTTTGAAACCGAAAAGAAGCATTTCACAATTCTAGATGCCCCTGGCCACAAGAGTTTTGTCCCAAATATGATTGGTGGTGCCTCTCAAGCTGATTTGGCTGTGCTGGTAATCTCAGCCAGGAAAGGAGAGTTTGAAACTGGATTTGAAAAAGGAGGACAGACAAGAGAACATGCAATGTTGGCAAAGACAGCAGGTGTAAAACACCTAATTGTGCTAATTAATAAGATGGATGATCCAACAGTAAATTGGAGCAATGAGAGATATGAAGAATGTAAGGAGAAACTAGTGCCATTTTTGAAAAAAGTTGGCTTCAATCCCAAAAAGGACATTCACTTTATGCCCTGCTCAGGACTTACTGGAGCAAATCTCAAAGAGCAGTCGGATTTCTGTCCTTGGTACATTGGATTACCGTTTATTCCATATCTGGATAATTTGCCGAACTTCAATAGATCAGTTGATGGACCAATCAGGCTGCCAATTGTGGATAAGTACAAGGATATGGGCACTGTGGTCCTGGGAAAGCTGGAATCAGGATCTATTTGTAAAGGCCAGCAGCTTGTGATGATGCCAAACAAGCACAACGTGGAAGTTCTTGGAATACTTTCCGATGATGTAGAGACTGATACCGTAGCCCCAGGTGAAAACCTCAAAATCAGACTGAAAGGAATTGAAGAAGAGGAGATTCTTCCAGGGTTTATACTTTGTGATCCTAATAATCTTTGTCATTCTGGACGCACATTTGATGCCCAGATAGTGATTATAGAGCACAAATCCATCATCTGCCCAGGCTATAATGCGGTGCTGCATATTCATACCTGTATTGAGGAGGTGGAAATAACAGCCTTAATCTGCTTGGTAGACAAAAAATCAGGAGAAAAAAGTAAGACCCGACCCCGTTTTGTCAAACAAGATCAAGTATGCATTGCTCGCTTAAGGACAGCAGGAACCATCTGCCTTGAGACCTTTAAAGACTTCCCTCAGATGGGTCGTTTCACCTTAAGAGATGAGG |
| hsa_circ_0038773 | GCTTTCGGACAGTCCTTTCTCCAGCCCGACATCCACCTTTTTAAACAAAATCTCTTCTACTTGGAGACTCTCAACACCAAGCAGAAGCTGTACCACAAGAAGATCTTCCGGACTGCCATGCTGTTCCAGTTTGTGAACGTGCTGCTCCAGGTCCTGGTCCACAAGTCCCATGATCTTCTGCAGGAGGAGATTGGCATCGCCATCTACAACATGGCCTCAGTCGACTTTGATGGCTTCTTTGCCGCCTTCCTCCCAGAGTTCCTGACCAGCTGTGATGGTGTGGATGCCAACCAGAAAAGTGTGCTGGGGCGGAATTTCAAGATGGATCGG |
| hsa_circ_0038794 | CCTACTCATGAAGGTTACTTCTCTTGTTTGGATATCTGGACGCTGTTTTTGGACTATCTGACAAGTAAAATTAAAAGTCGTCTTGGAGACAAGGAAGCAGTTCTCAACAGGTACGAAGATGCCCTGGTGCTCCTGCTCACAGAGGTGTTGAATCGAATCCAGTTCAGATACAACCAAGCCCAGCTGGAGGAGTTGGATGATGAGACTCTGGATGACGATCAGCAGACGGAGTGGCAGCGGTACTTACGGCAGAGCTTGGAGGTGGTGGCCAAAGTGATGGAGCTCCTGCCCACGCACGCCTTCTCCACACTGTTCCCTGTTCTTCAGGACAATTTAGAAGTTTATTTGGGATTACAACAGTTTATAGTCACTTCAGGGTCAGGACACAGGTTGAACATCACGGCGGAGAACGACTGCCGGCGGCTGCACTGCTCCCTGAGAGACTTGAGCTCCCTGCTGCAGGCCGTGGGCCGCCTGGCCGAGTACTTTATCGGGGATGTGTTTGCTGCACGGTTCAATGATGCCCTCACAGTCGTGGAAAGGTTGGTCAAAGTCACTCTGTACGGATCTCAGATAAAATTGTACAACATTGAAACTGCTGTGCCATCAGTATTGAAACCTGACCTCATTGATGTGCATGCTCAGTCCCTGGCTGCGCTGCAGGCTTACTCTCACTGGTTAGCACAGTATTGCAGTGAAGTTCACCGGCAGAACACGCAGCAGTTCGTGACACTCATCTCTACTACCATGGATGCAATCACACCTCTAATCAGCACCAAGGTCCAAGACAAGCTGCTGCTATCTGCGTGCCACTTACTGGTCTCACTGGCCACCACCGTGCGGCCCGTCTTTCTGATCAGCATCCCTGCAGTGCAGAAAGTATTCAACAGAATCACTGATGCCTCTGCCCTGCGACTTGTCGATAAGGCCCAGGTGTTGGTGTGCCGAGCCCTCTCTAACATCTTGCTGCTTCCGTGGCCAAACCTTCCAGAGAATGAGCAGCAGTGGCCCGTGCGCTCCATCAACCACGCCAGCCTCATCTCTGCACTCTCCCGGGACTATCGCAACCTGAAGCCCAGTGCTGTTGCCCCACAGAGAAAGATGCCACTGGATGACA |
| hsa_circ_0039522 | GAGTCTGCACTATGGAAACAACCTGTCAATCCAGCTCAAGGCACACATAGCCCAGACACCCATGAGACCCTCTCCGTGGGGACCCTAGAGCACCTATCATGAACGAGGAGACCAAGGCTGGCTCCTCATGGACCCCGTTGGCCTCCAGCTCGGCAACAAGAACCTGTGGAGCTGTCTTGTGAGGCTGCTCACCAAAGACCCAGAATGGCTGAACGCCAAGATGAAGTTCTTCCTCCCCAACACGGACCTGGATTCCAGGAACGAGACCTTGGACCCTGAACAGAGAGTCATCCTGCAACTCAACAAGCTGCATGTCCAGGGTTCGGACACCTGGCAGTCTTTCATTCATTGTGTGTGCATGCAGCTGGAGGTGCCTCTGGACCTGGAGGTGCTGCTGCTGAGTACTTTTGGCTATGATGATG |
| hsa_circ_0043025 | AATGATGCCAATGGGTGGAATGATGCCACCTGGACCAGGAATACCACCTCTGATGCCTGGAATGCCACCAGGTATGCCCCCACCTGTTCCACGTCCTGGAATTCCTCCAATGACTCAAGCACAGGCTGTTTCAGCGCCAGGTATTCTTAATAGACCACCTGCACCAACAGCAACTGTACCTGCCCCACAGCCTCCAGTTACTAAGCCTCTTTTCCCCAGTGCTGGACAG |
| hsa_circ_0044269 | AGGATCTCTGGGAAAGTTTAGAAAATGCTAGTGGTAAACCTATAGCAGCTGTGATGAATACCTGGACCAAACAAATGGGATTTCCCCTCATTTATGTGGAAGCTGAACAGGTAGAAGATGACAGATTATTGAGGTTGTCCCAAAAGAAGTTCTGTGCTGGTGGGTCATATGTTG |
| hsa_circ_0044520 | GGTCCCACCGGCCCCGCTGGTCCTCCTGGCTTCCCTGGTGCTGTTGGTGCTAAGGGTGAAGCTGGTCCCCAAGGGCCCCGAGGCTCTGAAGGTCCCCAGGGTGTGCGTGGTGAGCCTGGCCCCCCTGGCCCTGCTGGTGCTGCTGGCCCTGCTGGAAACCCTGGTGCTGATGGACAGCCTGGTGCTAAAGGTGCCAATGGTGCTCCTGGTATTGCTGGTGCTCCTGGCTTCCCTGGTGCCCGAGGCCCCTCTGGACCCCAGGGCCCCGGCGGCCCTCCTGGTCCCAAGGGTAACAGCGGTGAACCTGGTGCTCCTGGCAGCAAAGGAGACACTGGTGCTAAGGGAGAGCCTGGCCCTGTTGGTGTTCAAGGACCCCCTGGCCCTGCTGGAGAGGAAGGAAAGCGAGGAGCTCGAGGTGAACCCGGACCCACTGGCCTGCCCGGACCCCCTGGCGAGCGTGGTGGACCTGGTAGCCGTGGTTTCCCTGGCGCAGATGGTGTTGCTGGTCCCAAGGGTCCCGCTGGTGAACGTGGTTCTCCTGGCCCTGCTGGCCCCAAAGGATCTCCTGGTGAAGCTGGTCGTCCCGGTGAAGCTGGTCTGCCTGGTGCCAAGGGTCTGACTGGAAGCCCTGGCAGCCCTGGTCCTGATGGCAAAACTGGCCCCCCTGGTCCCGCCGGTCAAGATGGTCGCCCCGGACCCCCAGGCCCACCTGGTGCCCGTGGTCAGGCTGGTGTGATGGGATTCCCTGGACCTAAAGGTGCTGCTGGAGAGCCCGGCAAGGCTGGAGAGCGAGGTGTTCCCGGACCCCCTGGCGCTGTCGGTCCTGCTGGCAAAGATGGAGAGGCTGGAGCTCAGGGACCCCCTGGCCCTGCTGGTCCCGCTGGCGAGAGAGGTGAACAAGGCCCTGCTGGCTCCCCCGGATTCCAGGGTCTCCCTGGTCCTGCTGGTCCTCCAGGTGAAGCAGGCAAACCTGGTGAACAGGGTGTTCCTGGAGACCTTGGCGCCCCTGGCCCCTCTGGAGCAAGAGGCGAGAGAGGTTTCCCTGGCGAGCGTGGTGTGCAAGGTCCCCCTGGTCCTGCTGGTCCCCGAGGGGCCAACGGTGCTCCCGGCAACGATGGTGCTAAGGGTGATGCTGGTGCCCCTGGAGCTCCCGGTAGCCAGGGCGCCCCTGGCCTTCAGGGAATGCCTGGTGAACGTGGTGCAGCTGGTCTTCCAGGGCCTAAGGGTGACAGAGGTGATGCTGGTCCCAAAGGTGCTGATGGCTCTCCTGGCAAAGATGGCGTCCGTGGTCTGACTGGCCCCATTGGTCCTCCTGGCCCTGCTGGTGCCCCTGGTGACAAGGGTGAAAGTGGTCCCAGCGGCCCTGCTGGTCCCACTGGAGCTCGTGGTGCCCCCGGAGACCGTGGTGAGCCTGGTCCCCCCGGCCCTGCTGGCTTTGCTGGCCCCCCTGGTGCTGACGGCCAACCTGGTGCTAAAGGCGAACCTGGTGATGCTGGTGCTAAAGGCGATGCTGGTCCCCCTGGCCCTGCCGGACCCGCTGGACCCCCTGGCCCCATTGGTAATGTTGGTGCTCCTGGAGCCAAAGGTGCTCGCGGCAGCGCTGGTCCCCCTGGTGCTACTGGTTTCCCTGGTGCTGCTGGCCGAGTCGGTCCTCCTGGCCCCTCTGGAAATGCTGGACCCCCTGGCCCTCCTGGTCCTGCTGGCAAAGAAGGCGGCAAAGGTCCCCGTGGTGAGACTGGCCCTGCTGGACGTCCTGGTGAAGTTGGTCCCCCTGGTCCCCCTGGCCCTGCTGGCGAGAAAGGATCCCCTGGTGCTGATGGTCCTGCTGGTGCTCCTGGTACTCCCGGGCCTCAAGGTATTGCTGGACAGCGTGGTGTGGTCGGCCTGCCTGGTCAGAGAGGAGAGAGAGGCTTCCCTGGTCTTCCTGGCCCCTCTGGTGAACCTGGCAAACAAGGTCCCTCTGGAGCAAGTGGTGAACGTGGTCCCCCTGGTCCCATGGGCCCCCCTGGATTGGCTGGACCCCCTGGTGAATCTGGACGTGAGGGGGCTCCTGGTGCCGAAGGTTCCCCTGGACGAGACGGTTCTCCTGGCGCCAAGGGTGACCGTGGTGAGACCGGCCCCGCTGGACCCCCTGGTGCTCCTGGTGCTCCTGGTGCCCCTGGCCCCGTTGGCCCTGCTGGCAAGAGTGGTGATCGTGGTGAGACTGGTCCTGCTGGTCCCACCGGTCCTGTCGGCCCTGTTGGCGCCCGTGGCCCCGCCGGACCCCAAGGCCCCCGTGGTGACAAGGGTGAGACAGGCGAACAGGGCGACAGAGGCATAAAGGGTCACCGTGGCTTCTCTGGCCTCCAGGGTCCCCCTGGCCCTCCTGGCTCTCCTGGTGAACAAGGTCCCTCTGGAGCCTCTGGTCCTGCTGGTCCCCGAGGTCCCCCTGGCTCTGCTGGTGCTCCTGGCAAAGATGGACTCAACGGTCTCCCTGGCCCCATTGGGCCCCCTGGTCCTCGCGGTCGCACTGGTGATGCTGGTCCTGTT |
| hsa_circ_0044531 | GGTCCCGCCGGTCAAGATGGTCGCCCCGGACCCCCAGGCCCACCTGGTGCCCGTGGTCAGGCTGGTGTGATGGGATTCCCTGGACCTAAAGGTGCTGCTGGAGAGCCCGGCAAGGCTGGAGAGCGAGGTGTTCCCGGACCCCCTGGCGCTGTCGGTCCTGCTGGCAAAGATGGAGAGGCTGGAGCTCAGGGACCCCCTGGCCCTGCTGGTCCCGCTGGCGAGAGAGGTGAACAAGGCCCTGCTGGCTCCCCCGGATTCCAGGGTCTCCCTGGTCCTGCTGGTCCTCCAGGTGAAGCAGGCAAACCTGGTGAACAGGGTGTTCCTGGAGACCTTGGCGCCCCTGGCCCCTCTGGAGCAAGAGGCGAGAGAGGTTTCCCTGGCGAGCGTGGTGTGCAAGGTCCCCCTGGTCCTGCTGGTCCCCGAGGGGCCAACGGTGCTCCCGGCAACGATGGTGCTAAGGGTGATGCTGGTGCCCCTGGAGCTCCCGGTAGCCAGGGCGCCCCTGGCCTTCAGGGAATGCCTGGTGAACGTGGTGCAGCTGGTCTTCCAGGGCCTAAGGGTGACAGAGGTGATGCTGGTCCCAAAGGTGCTGATGGCTCTCCTGGCAAAGATGGCGTCCGTGGTCTGACTGGCCCCATTGGTCCTCCTGGCCCTGCTGGTGCCCCTGGTGACAAGGGTGAAAGTGGTCCCAGCGGCCCTGCTGGTCCCACTGGAGCTCGTGGTGCCCCCGGAGACCGTGGTGAGCCTGGTCCCCCCGGCCCTGCTGGCTTTGCTGGCCCCCCTGGTGCTGACGGCCAACCTGGTGCTAAAGGCGAACCTGGTGATGCTGGTGCTAAAGGCGATGCTGGTCCCCCTGGCCCTGCCGGACCCGCTGGACCCCCTGGCCCCATTGGTAATGTTGGTGCTCCTGGAGCCAAAGGTGCTCGCGGCAGCGCTGGTCCCCCTGGTGCTACTGGTTTCCCTGGTGCTGCTGGCCGAGTCGGTCCTCCTGGCCCCTCTGGAAATGCTGGACCCCCTGGCCCTCCTGGTCCTGCTGGCAAAGAAGGCGGCAAAGGTCCCCGTGGTGAGACTGGCCCTGCTGGACGTCCTGGTGAAGTTGGTCCCCCTGGTCCCCCTGGCCCTGCTGGCGAGAAAGGATCCCCTGGTGCTGATGGTCCTGCTGGTGCTCCTGGTACTCCCGGGCCTCAAGGTATTGCTGGACAGCGTGGTGTGGTCGGCCTGCCTGGTCAGAGAGGAGAGAGAGGCTTCCCTGGTCTTCCTGGCCCCTCT |
| hsa_circ_0044541 | GGTCCCGCCGGTCAAGATGGTCGCCCCGGACCCCCAGGCCCACCTGGTGCCCGTGGTCAGGCTGGTGTGATGGGATTCCCTGGACCTAAAGGTGCTGCTGGAGAGCCCGGCAAGGCTGGAGAGCGAGGTGTTCCCGGACCCCCTGGCGCTGTCGGTCCTGCTGGCAAAGATGGAGAGGCTGGAGCTCAGGGACCCCCTGGCCCTGCTGGTCCCGCTGGCGAGAGAGGTGAACAAGGCCCTGCTGGCTCCCCCGGATTCCAGGGTCTCCCTGGTCCTGCTGGTCCTCCAGGTGAAGCAGGCAAACCTGGTGAACAGGGTGTTCCTGGAGACCTTGGCGCCCCTGGCCCCTCTGGAGCAAGAGGCGAGAGAGGTTTCCCTGGCGAGCGTGGTGTGCAAGGTCCCCCTGGTCCTGCTGGTCCCCGAGGGGCCAACGGTGCTCCCGGCAACGATGGTGCTAAGGGTGATGCTGGTGCCCCTGGAGCTCCCGGTAGCCAGGGCGCCCCTGGCCTTCAGGGAATGCCTGGTGAACGTGGTGCAGCTGGTCTTCCAGGGCCTAAGGGTGACAGAGGTGATGCTGGTCCCAAAGGTGCTGATGGCTCTCCTGGCAAAGATGGCGTCCGTGGTCTGACTGGCCCCATTGGTCCTCCTGGCCCTGCTGGTGCCCCTGGTGACAAGGGTGAAAGTGGTCCCAGCGGCCCTGCTGGTCCCACTGGAGCTCGTGGTGCCCCCGGAGACCGTGGTGAGCCTGGTCCCCCCGGCCCTGCTGGCTTTGCTGGCCCCCCTGGTGCTGACGGCCAACCTGGTGCTAAAGGCGAACCTGGTGATGCTGGTGCTAAAGGCGATGCTGGTCCCCCTGGCCCTGCCGGACCCGCTGGACCCCCTGGCCCCATTGGTAATGTTGGTGCTCCTGGAGCCAAAGGTGCTCGCGGCAGCGCTGGTCCCCCTGGTGCTACTGGTTTCCCTGGTGCTGCTGGCCGAGTCGGTCCTCCTGGCCCCTCT |
| hsa_circ_0044542 | GGTGCTGACGGCCAACCTGGTGCTAAAGGCGAACCTGGTGATGCTGGTGCTAAAGGCGATGCTGGTCCCCCTGGCCCTGCCGGACCCGCTGGACCCCCTGGCCCCATTGGTAATGTTGGTGCTCCTGGAGCCAAAGGTGCTCGCGGCAGCGCTGGTCCCCCT |
| hsa_circ_0044554 | GGTGAGCCTGGCAGCCCTGGTGAAAATGGAGCTCCTGGTCAGATGGGCCCCCGTGGCCTGCCTGGTGAGAGAGGTCGCCCTGGAGCCCCTGGCCCTGCTGGTGCTCGTGGAAATGATGGTGCTACTGGTGCTGCCGGGCCCCCTGGTCCCACCGGCCCCGCTGGTCCTCCTGGCTTCCCTGGTGCTGTTGGTGCTAAGGGTGAAGCTGGTCCCCAAGGGCCCCGAGGCTCTGAAGGTCCCCAGGGTGTGCGTGGTGAGCCTGGCCCCCCTGGCCCTGCTGGTGCTGCTGGCCCTGCTGGAAACCCTGGTGCTGATGGACAGCCTGGTGCTAAAGGTGCCAATGGTGCTCCTGGTATTGCTGGTGCTCCTGGCTTCCCTGGTGCCCGAGGCCCCTCTGGACCCCAGGGCCCCGGCGGCCCTCCTGGTCCCAAGGGTAACAGCGGTGAACCTGGTGCTCCTGGCAGCAAAGGAGACACTGGTGCTAAGGGAGAGCCTGGCCCTGTTGGTGTTCAAGGACCCCCTGGCCCTGCTGGAGAGGAAGGAAAGCGAGGAGCTCGAGGTGAACCCGGACCCACTGGCCTGCCCGGACCCCCTGGCGAGCGTGGTGGACCTGGTAGCCGTGGTTTCCCTGGCGCAGATGGTGTTGCTGGTCCCAAGGGTCCCGCTGGTGAACGTGGTTCTCCTGGCCCTGCTGGCCCCAAAGGATCTCCTGGTGAAGCTGGTCGTCCCGGTGAAGCTGGTCTGCCTGGTGCCAAGGGTCTGACTGGAAGCCCTGGCAGCCCTGGTCCTGATGGCAAAACTGGCCCCCCTGGTCCCGCCGGTCAAGATGGTCGCCCCGGACCCCCAGGCCCACCTGGTGCCCGTGGTCAGGCTGGTGTGATGGGATTCCCTGGACCTAAAGGTGCTGCT |
| hsa_circ_0044894 | GTTATTGTTCAAAACTACAACTCCATTTTGACACTTTCTCACTTGTACCGATCTTCAGACGCCCTCCTTCTTCATGAGAATGATGCCATCCATAAGATCTGTGCAAAACTGATGAATATCAAGCAGATCTCCTTTAGTGATATCAATCAAGTCCTCGCACATCAGCTGGGAAGTGTGTTCCAGCCTACTTATTCTGCAGAAAGCTCATTTCACTACAGACGAAATCCACTAGGAGACTTAATGGAGCATTTAGTTCCCCATCCTGAATTCAAGATGCTGAGTGTTCGTAACATTCCTCACATGTCTGAGAATTCATTGGCATACACCACATTTACTTGGGCTGGCCTCCTCAAGCATTTGAGACAGATGCTCATTTCTAATGCAAAGATGGAAGAAGGTATTGATAGGCATGTATGGCCTCCTTTATCAGGACTTCCTCCTCTTAGTAAAATGTCTCTCAACAAGGACCTGCATTTTAACACTTCCATTGCTAACTTGGTCATTCTTCGTGGGAAAGATGTGCAAAGTGCAGATGTGG |
| hsa_circ_0046909 | GCCCCTTCACAGATGTAGTCACTACAAATCTTAAATTGCGAAATCCATCGGATAGAAAAGTGTGTTTCAAAGTGAAGACTACAGCACCTCGCCGGTACTGTGTGAGGCCCAACAGTGGAATTATTGACCCAGGGTCAACTGTGACTGTTTCAGTAATGCTACAGCCCTTTGACTATGATCCGAATGAAAAGAGTAAACACAAGTTTATGGTACAGACAATTTTTGCTCCACCAAACACTTCAGATATGGAAGCTGTGTGGAAAGAGGCAAAACCTGATGAATTAATGGATTCCAAATTGAGATGCGTATTTGAAATGCCCAATGAAAATGATAAATTGGGTATAACTCCACCAGGGAATGCTCCGACTGTCACTTCAATGAGCAGCATCAACAACACAGTTGCAACACCTGCCAGTTATCACACGAAGGATGACCCCAGGGGACTCAGTGTGTTGAAACAGGAGAAACAGAAGAATGATATGGAACCTAGCAAAGCTGTTCCACTGAATGCATCTAAGCAAGATGGACCTATGCCAAAACCACACAGTGTTTCACTTAATGATACCGAAACAAGGAAACTAATGGAAGAGTGTAAAAGACTTCAGGGAGAAATGATGAAGCTATCAGAAGAAAATCGGCACCTGAGA |
| hsa_circ_0047961 | GGTTGCTTTTGTAATGAAGAAGCACTTAAATACTCATCTACTAGGCAAGCATGGAGTTGGCACCCCAAAAGAAAGGAAATTTACATGCCACTTATGTGATAGAAGTTTCACAGAGAAGTGGGCCCTGAACAACCACATGAAACTCCACACGGGAGAAAAGCCGTTTAAATGTACCTGGCCCACGTGCCATTACTCATTCCTCACAGCCTCCGCAATGAAAGACCACTACAGGACGCACACAGGCGAGAAGTCGTTTCTGTGTGACCTCTGCGGCTTTGCCGGCGGGACCCGCCACGCCCTCACCAAGCATCGCAGACAGCACACAGGAGAAAAACCTTTCAAGTGCGATGAGTGTAACTTTGCCTCCACAACTCAGTCCCATTTGACTCGGCATAAACGTGTCCACACTGGAGAAAAGCCCTACAGATGCCCCTGGTGTGACTACAG |
| hsa_circ_0048504 | GTTTGGCTCAGAAGAAGGCGGCCCCAACAGAGGTTCTGTCCATGACGGCACAGCCGGGCCCTGGCCATGGGAAGAAGTTGGGCCATCGAGGTGTGGACGCATCCGGCGAAACCACCTACAAGAAGACCACCTCCTCCACCCTGAAGGGTGCCATCCAGCTGGGCATCGGCTACACCGTGGGCCACCTGAGCTCCAAGCCCGAACGCGACGTGCTCATGCAGGACTTCTACGTGGTGGAGAGCATCTTCTTCCCCAGCGAAGGCAGCAACCTCACCCCCGCCCACCACTTCCAGGACTTCCGCTTCAAGACCTATGCACCTGTCGCCTTCCGCTACTTCCGGGAGCTCTTTGGGATCCGGCCAGATGATTACTTGTACTCCCTGTGCAATGAGCCGCTGATCGAGCTGTCCAACCCGGGCGCCAGTGGCTCCCTCTTCTACGTCACCAGCGACGACGAGTTCATCATCAAGACCGTCATGCACAAGGAGGCCGAGTTCCTGCAGAAGCTGCTCCCTGGCTACTACATGAACCTCAACCAGAACCCGCGGACGCTGCTGCCCAAGTTCTATGGGCTGTACTGCGTGCAGTCGGGGGGCAAGAACATCCGCGTCGTGGTCATGAACAACATCCTGCCCCGCGTGGTCAAGATGCACCTCAAGTTCGACCTCAAGGGCTCCACCTACAAGCGGCGCGCCAGCAAGAAGGAGAAGGAGAAGAGCTTCCCCACCTACAAGGACCTGGACTTCATGCAGGACATGCCCGAGGGGCTCCTGCTGGACGCCGACACCTTCAGCGCCCTGGTCAAGACGCTGCAGCGGGACTGCCTGGTCCTGGAAAGTTTCAAGATCATGGACTACAGCCTGCTGCTGGGCGTGCACAACATCGACCAGCACGAGCGCGAGCGGCAGGCGCAGGGCGCCCAGAGCACCTCAGATGAGAAGCGGCCTGTGGGCCAGAAGGCGCTCTACTCCACGGCCATGGAGTCCATCCAGGGTGGCGCCGCGCGCGGGGAGGCCATCGAATCGGATGACAC |
| hsa_circ_0049024 | AGATGCCTCGGCAGTGGAATCCAATTTCTTTCCAGCCACAACCTACCCCATGGGTCGACCTATCAGATGCGCCGGCCGGGCGGAGAGCTGCCACTGTCCAAATCATATTCTTCTGGAAACAGAAAAGGCTTTCTGTCCGGCTTGCTAGATAATGTCAAACAAGAATTAGCCAAAAACAAAGAAATGAAAGAAAGTATAAAAAAATTCCGTGACGAGGCCAGAAGGCTAGAAGAATCAGACGTGCTCCAGGAGGCCAGAAGGAAATAC |
| hsa_circ_0049164 | CACGGATGATACCATCGGGGACCTTAAGAAGCTGATTGCAGCCCAAACTGGTACCCGTTGGAACAAGATTGTCCTGAAGAAGTGGTACACGATTTTTAAGGACCACGTGTCTCTGGGGGACT |
| hsa_circ_0049767 | GTGGAGATTCTGGACGCAAAGACAAGGGAGAAGCTGTGTTTCTTGGACAAG |
| hsa_circ_0049865 | TATTCTGAAAAGGAGGACAAATATGAAGAAGAAATTAAACTTCTGTCTGACAAACTGAAAGAGGCTGAGACCCGTGCTGAATTTGCAGAGAGAACGGTTGCAAAACTGGAAAAGACAATTGATGACCTGGAAG |
| hsa_circ_0050463 | GTCGCTCAAGCCCTCAGTTAGACCCTTTGAGAAAAAGCCCAACCATGGAACAAGCAGTGCAGACCGCCTCAGCCCACTTACCTGCTCCAGCAGCTGTTGGGAGAAGGAGTCCTGTATCAACCAGGCCTTTGCCATCTGCCAGCCAAAAGGCAGGAGAGAATCAGGAGCACAGGCGAGCTGAAGTACACAAAGTTTCAAGGCCAGAAAATGAGCAACTCAGAAATGATAACAAGAGACAAGTAG |
| hsa_circ_0050545 | GGGAACCAACGGAAGCCGAAGCCAGAGCTAGAGCATCTAATGAAGATGGTGACATTAAACGTATTTCTACTAAGGAATGGGCTAAATCAACTGGATATGATCCAGTTAAACTTTTTACCAAGCTTTTTAAAGATGACATCAGGTATCTGTTGACAATGGACAAACTATGGCGGAAAAGGAAACCTCCAGTTCCGTTGGACTGGGCTGAAGTACAAAGTCAAGGAGAAGAAACGAATGCATCAGATCAACAGAATGAACCCCAGTTAGGCCTGAAAGACCAGCAGGTTCTAGATGTAAAGAGCTATGCACGTCTTTTTTCAAAGAGCATCGAGACTTTGAGAGTTCATTTAGCAGAAAAGGGGGATGGAGCTGAGCTCATATGGGATAAGGATGACCCATCTGCAATGGATTTTGTCACCTCTGCTGCAAACCTCAGGATGCATATTTTCAGTATGAATATGAAGAGTAGATTTGATATCAAATCAATGGCAGGGAACATTATTCCTGCTATTGCTACTACTAATGCAGTAATTGCTGGGTTGATAGTATTGGAAGGATTGAAGATTTTATCAGGAAAAATAGACCAGTGCAGAACAATTTTTTTGAATAAACAACCAAACCCAAGAAAGAAGCTTCTTGTGCCTTGTGCACTGGATCCTCCCAACCCCAATTGTTATGTATGTGCCAGCAAGCCAGAGGTGACTGTGCGGCTGAATGTCCATAAAGTGACTGTTCTCACCTTACAAGACAAGATAGTGAAAGAAAAATTTGCTATGGTAGCACCAGATGTCCAAATTGAAGATGGGAAAGGAACAATCCTAATATCTTCCGAAGAGGGAGAGACGGAAGCTAATAATCACAAGAAGTTGTCAGAATTTGGAATTAGAAATGGCAGCCGGCTTCAAGCAGATGACTTCCTCCAGGACTATACTTTATTGATCAACATCCTTCATAGTGAAGACCTAGGAAAGGACGTTGAATTTGAAGTTGTTGGTGATGCCCCGGAAAAAGTGGGGCCCAAACAAGCTGAAGATGCTGCCAAAAGCATAACCAATGGCAGTGATGATGGAGCTCAGCCCTCCACCTCCACAG |
| hsa_circ_0052817 | TCCGTGACAAGTGTGCCCTAGGCTCTCATGGTTGCCAGCACATTTGTGTGAGTGATGGGGCCGCATCCTACCACTGTGATTGCTATCCTGGCTACACCTTAAATGAGGACAAGAAAACATGTTCAG |
| hsa_circ_0052941 | GCTACTGTGGAGCCGATATCAAGGCCCTGTGCACTGAAGCCGCCCTGATTGCACTGCGGAGGCGTTATCCCCAGATCTATGCTAGCAGTCATAAACTGCAGCTGGATGTTTCCTCAATAGTGCTTAGTGCCCAAGATTTTTACCATGCAATGCAGAATATCGTGCCTGCTTCCCAACGTGCTGTGATGTCTTCAGGGCATGCACTATCCCCCATCATAAGACCACTGCTGGAAAGAAGCTTCAACAACATCCTAGCAGTCTTGCAAAAAGTGTTTCCTCATGCTGAAATTAGCCAGAGTGACAAAAAAGAAGATATAGAAACTTTAATTTTAGAGGATAGTGAAGATGAAAATGCTTTATCAATTTTTGAGACCAATTGTCACTCAGGATCACCAAAGAAACAGTCATCATCTGCTGCTATACATAAACCCTACCTTCATTTTACAATGTCACCATATCATCAGCCAACCTCTTACAGGCCACGCTTATTGCTCTCTGGAGAACGGGGCTCAGGTCAAACTTCTCACCTTGCTCCAGCACTTTTGCACACTCTAGAAAGATTCTCTGTGCATAGACTAGATCTCCCAGCACTTTATTCAGTTAGTGCCAAAACACCTGAGGAATCATGTGCACAGATTTTTCGTGAAGCTCGAAGAACAGTACCTAGTATTGTTTACATGCCTCACATTGGGGATTGGTGGGAAGCTGTCAGTGAAACTGTGAGAGCAACTTTTCTGACATTGCTACAAGATATACCATCATTTTCACCTATATTTTTATTGTCTACCTCTGAAACCATGTACAGTGAACTGCCTGAAGAG |
| hsa_circ_0053926 | ATATTGATGAGTGTACTCAGGTCCAACACCTCTGCTCCCAGGGCCGCTGTGAAAACACCGAGGGAAGTTTCTTGTGCATTTGCCCAGCAGGATTTATGGCCAGTGAGGAGGGTACTAACTGCATAGATGTTGACGAATGCCTGAGGCCGGACGTCTGTGGGGAGGGGCACTGTGTCAATACTGTGGGGGCCTTCCGGTGTGAATACTGTGACAGCGGGTACCGCATGACTCAGAGAGGCCGTTGTGAGGATATTGATGAATGTTTGAATCCAAGCACTTGTCCAGATGAGCAGTGTGTGAATTCTCCTGGATCTTACCAGTGCGTTCCCTGCACAGAAGGATTCCGAGGCTGGAATGGACAGTGCCTTGATGTGGACGAGTGCCTGGAACCAAACGTCTGCGCAAATGGTGATTGTTCCAACCTTGAAGGCTCCTACATGTGTTCATGCCACAAAGGCTATACCCGGACTCCGGACCACAAGCACTGTAGAG |
| hsa_circ_0054677 | ATTATTATGTAGTTGGCAGCTGAGAACAATACTTAGTGGATACCATCGAATAGTACAACAGAGAATGCAGCACTCTCCTGATCTAATGAGCTTTATGATGGAGTTGAAGATGCTTTTGGAAGTTGCCTTAAAGAATAGACAAGAGCTGTATGCACTACCTCCTCCTCCCCAGTTCTACTCAAGCCTTATTGAAGAGATAGGAACTCTTGGTTGGGATAA |
| hsa_circ_0054982 | GATCTTATGGTTGGTGATGAGGCAAGTGAATTACGATCAATGTTAGAAGTTAACTACCCTATGGAAAATGGCATAGTACGAAATTGGGATGACATGAAACACCTGTGGGACTACACATTTGGACCAGAGAAACTTAATATAGATACCAGAAATTGTAAAATCTTACTCACAGAACCTCCTATGAACCCAACCAAAAACAGAGAGAAGATTGTAGAGGTAATGTTTGAAACTTACCAGTTTTCCGGTGTATATGTAGCCATCCAGGCAGTTCTGACTTTGTACGCTCAAG |
| hsa_circ_0055779 | ACCGAGACAACACTTGGACTCAGTTCATATCAGCAGAAAAGTATATCTCTCTACCGGGGGAATTGCAGGCCCATACGATTTGAGCCACCAATGCTGGATTTCCATGAACA |
| hsa_circ_0055924 | GACTTGTCTTACAAGGACCGGCACTGGCATGAAGCCTGTTTCCACTGCTCGCAGTGCAGAAACTCACTGGTGGACAAGCCCTTTGCTGCCAAGGAGGACCAGCTGCTCTGTACAGACTGCTATTCCAACGAGTACTCATCCAAGTGCCAGGAATGCAAGAAGACCATCATGCCAGGTACCCGCAAGATGGAGTACAAGGGCAGCAGCTGGCATGAGACCTGCTTCATCTGCCACCGCTGCCAGCAGCCAATTGGAACCAAGAGTTTCATCCCCAAAGACAATCAGAATTTCTGTGTGCCCTGCTATGAGAAACAACATGCCATGCAGTGCGTTCAGTGCAAAAAGCCCATCACCACGGGAGGGGTCACTTACCGGGAGCAGCCCTGGCACAAGGAGTGCTTCGTGTGCACCGCCTGCAGGAAGCAGCTGTCTGGGCAGCGCTTCACAGCTCGCGATGACTTTGCCTACTGCCTGAACTGCTTCTGTGACTTGTATGCCAAGAAGTGTGCTGGGTGCACCAACCCCATCAGCG |
| hsa_circ_0056356 | CTGGTCCCACCTATAGGAATGTTGAATAATCCTATGAATGCAGTAACAACAAAATTTGTTCGGACATCAACAAATAAAGTAAAGTGTCCTGTATTTGTTGTTAGGTGGACTCCAGAAGGAAGACGCTTGGTCACTGGAGCTTCTAGTGGGGAGTTTACCCTGTGGAATGGACTCACTTTCAATTTTGAAACAATATTACAGGCTCACGACAGCCCAGTGAGGGCCATGACGTGGTCACATAATGACATGTGGATGTTGACAGCAGACCACGGAGGATATGTGAAATATTGGCAGTCGAACATGAACAACGTCAAGATGTTCCAGGCACATAAGGAGGCGATTAGAGAGGCCAGTTTCTCACCCACGGATAATAAATTTGCTACATGCTCTGATGACGGCACTGTTAGAATCTGGGACTTTCTTCGTTGCCATGAGGAAAGAATTCTCCGAGGGCATGGTGCTGATGTGAAATGTGTAGACTGGCATCCAACCAAAGGGTTAGTTGTTTCAGGAAGTAAAGATAGTCAACAGCCAATCAAGTTCTGGGATCCCAAGACTGGGCAGAGTCTTGCAACACTTCATGCCCATAAAAACACAGTAATGGAAGTGAAATTAAACCTCAATGGCAATTGGCTACTCACAGCATCACGTGATCATCTCTGTAAACTTTTTGATATCAGAAACCTAAAAGAAGAGCTTCAAGTCTTCCGAGGTCATAAGAAAGAAGCCACAGCTGTGGCCTGGCATCCTGTTCATGAAGGACTTTTTGCCAGTGGAGGGTCTGATGGTTCTTTGTTATTCTGGCATGTTGG |
| hsa_circ_0056651 | ATCAGCAGTTGTGTGATCCTGGTGAATTTCTTTGCCACGATCACGTGACTTGTGTCTCCCAGAGCTGGCTGTGTGATGGGGACCCTGACTGCCCTGATGATTCAGACGAGTCTTTAGATACCT |
| hsa_circ_0057261 | CTGTTGACAGTGTCTTGAAGAGGATGACAATAATTGGTGTAATTTTATCCTTCCGATCATTGGCACAAGAAGCACTTAGAGATGTCTTATCCTACCACATTCCTTTTCTTGTAAGTTCAATTGAAGATTTTAAGGATCACATTCCAAGGGAAACTGATATGAAG |
| hsa_circ_0057384 | GGTGAAGTTGGACCTGCAGGGTCTCCTGGTTCAAATGGTGCCCCTGGACAAAGAGGAGAACCTGGACCTCAGGGACACGCTGGTGCTCAAGGTCCTCCTGGCCCTCCTGGGATTAATGGTAGTCCTGGTGGTAAAGGCGAAATGGGTCCCGCTGGCATTCCTGGAGCTCCTGGACTGATGGGAGCCCGGGGTCCTCCAGGACCAGCCGGTGCTAATGGTGCTCCTGGACTGCGAGGTGGTGCAGGTGAGCCTGGTAAGAATGGTGCCAAAGGAGAGCCCGGACCACGTGGTGAACGCGGTGAGGCTGGTATTCCAGGTGTTCCAGGAGCTAAAGGCGAAGATGGCAAGGATGGATCACCTGGAGAACCTGGTGCAAATGGGCTTCCAGGAGCTGCAGGAGAAAGGGGTGCCCCTGGGTTCCGAGGACCTGCTGGACCAAATGGCATCCCAGGAGAAAAGGGTCCTGCTGGAGAGCGTGGTGCTCCAGGCCCTGCAGGGCCCAGAGGAGCTGCTGGAGAACCTGGCAGAGATGGCGTCCCTGGAGGTCCAGGAATGAGGGGCATGCCCGGAAGTCCAGGAGGACCAGGAAGTGATGGGAAACCAGGGCCTCCCGGAAGTCAAGGAGAAAGTGGTCGACCAGGTCCTCCTGGGCCATCTGGTCCCCGAGGTCAGCCTGGTGTCATGGGCTTCCCCGGTCCTAAAGGAAATGATGGTGCTCCTGGTAAGAATGGAGAACGAGGTGGCCCTGGAGGACCTGGCCCTCAGGGTCCTCCTGGAAAGAATGGTGAAACTGGACCTCAGGGACCCCCAGGGCCTACTGGGCCTGGTGGTGACAAAGGAGACACAGGACCCCCTGGTCCACAAGGATTACAAGGCTTGCCTGGTACAGGTGGTCCTCCAGGAGAAAATGGAAAACCTGGGGAACCAGGTCCAAAGGGTGATGCCGGTGCACCTGGAGCTCCAGGAGGCAAGGGTGATGCTGGTGCCCCTGGTGAACGTGGACCTCCTGGATTGGCAGGGGCCCCAGGACTTAGAGGTGGAGCTGGTCCCCCTGGTCCCGAAGGAGGAAAGGGTGCTGCTGGTCCTCCTGGGCCACCTGGTGCTGCTGGTACTCCTGGTCTGCAAGGAATGCCTGGAGAAAGAGGAGGTCTTGGAAGTCCTGGTCCAAAGGGTGACAAGGGTGAACCAGGCGGTCCAGGTGCTGATGGTGTCCCAGGGAAAGATGGCCCAAGGGGTCCTACTGGTCCTATTGGTCCTCCTGGCCCAGCTGGCCAGCCTGGAGATAAGGGTGAAGGTGGTGCCCCCGGACTTCCAGGTATAGCTGGACCTCGTGGTAGCCCT |
| hsa_circ_0057386 | GGTGAGCCTGGTAAGAATGGTGCCAAAGGAGAGCCCGGACCACGTGGTGAACGC |
| hsa_circ_0057402 | GGTGATGCTGGTGCCCCTGGTGAACGTGGACCTCCTGGATTGGCAGGGGCCCCAGGACTTAGAGGTGGAGCTGGTCCCCCTGGTCCCGAAGGAGGAAAGGGTGCTGCTGGTCCTCCTGGGCCACCTGGTGCTGCTGGTACTCCTGGTCTGCAAGGAATGCCTGGAGAAAGAGGAGGTCTTGGAAGTCCTGGTCCAAAGGGTGACAAGGGTGAACCAGGCGGTCCAGGTGCTGATGGTGTCCCAGGGAAAGATGGCCCAAGGGGTCCTACTGGTCCTATTGGTCCTCCTGGCCCAGCTGGCCAGCCTGGAGATAAGGGTGAAGGTGGTGCCCCCGGACTTCCAGGTATAGCTGGACCTCGTGGTAGCCCT |
| hsa_circ_0057408 | GGTGAACCAGGCGGTCCAGGTGCTGATGGTGTCCCAGGGAAAGATGGCCCAAGGGGTCCTACTGGTCCTATTGGTCCTCCTGGCCCAGCTGGCCAGCCTGGAGATAAGGGTGAAGGTGGTGCCCCCGGACTTCCAGGTATAGCTGGACCTCGTGGTAGCCCTGGTGAGAGAGGTGAAACTGGCCCTCCAGGACCTGCTGGTTTCCCTGGTGCTCCTGGACAGAATGGTGAACCTGGTGGTAAAGGAGAAAGAGGGGCTCCGGGTGAGAAAGGTGAAGGAGGCCCTCCTGGAGTTGCAGGACCCCCTGGAGGTTCTGGACCTGCTGGTCCTCCTGGTCCCCAAGGTGTCAAAGGTGAACGTGGCAGTCCTGGTGGACCTGGTGCTGCTGGCTTCCCTGGTGCTCGTGGTCTTCCTGGTCCTCCTGGTAGTAATGGTAACCCAGGACCCCCAGGTCCCAGCGGTTCTCCAGGCAAGGATGGGCCCCCAGGTCCTGCGGGTAACACTGGTGCTCCTGGCAGCCCTGGAGTGTCTGGACCAAAAGGTGATGCTGGCCAACCAGGAGAGAAGGGATCGCCTGGTGCCCAGGGCCCACCAGGAGCTCCAGGCCCACTTGGGATTGCTGGGATCACTGGAGCACGGGGTCTTGCAGGACCACCAGGCATGCCAGGTCCTAGGGGAAGCCCTGGCCCTCAGGGTGTCAAGGGTGAAAGTGGGAAACCAGGAGCTAACGGTCTCAGTGGAGAACGTGGTCCCCCTGGACCCCAGGGTCTTCCTGGTCTGGCTGGTACAGCTGGTGAACCTGGAAGAGATGGAAACCCTGGATCAGATGGTCTTCCAGGCCGAGATGGATCTCCTGGTGGCAAG |
| hsa_circ_0057602 | GAGAAACATCAGATGGAGTGAGGAAGTCAGTTCACAAGGTCTTTGCTTCCATGCTTGGAGAGAATGAAGATGATGAGGAGGAAGAGGAAGAAGAGGAGGAGGAGGAGGAGGAGGAAGAAACACCTGAGCAACCCACTGCGGGCGATGTATTTGTATTGGAGATGGTTCTCAATCGTGAAACCAAGAAAATGATGAAAGAGAAAAGGCCTCGGAGTAAACTTCCCAGAGCTCTGAGAGGTCTCATGGGTGAAGCCAACATTCGTTTTGCTCGAGGAGAACGTGAAGAGGCGATATTGATGTGCATGGAAATCATAAGACAAGCTCCTCTGGCTTATGAGCCATTCTCTACTCTAGCCATGATATATGAGGACCAAGGTGACATGGAAAAATCATTGCAGTTTGAGTTGATTGCTGCGCATTTAAATCCCAGTGACACAGAAGAATGGGTTAGACTGGCAGAAATGTCTCTGGAACAAGACAATATTAAGCAGGCTATTTTTTGCTATACAAAAGCTCTTAAATATGAACCTACTAATGTCCGTTATCTGTGGGAGCGATCAAGCCTTTATGAACAGATGGGTGATCATAAAATGGCCATGGATGGTTATAGGCGTATTTTAAACCTTTTGTCTCCATCTGATGGCGAACGTTTTATGCAGCTGGCTAGAGATATGGCAAA |
| hsa_circ_0057829 | ATGATCGTTGCCAGCCCAACAGAAAATGGACAGGTACTTCGTGTAATTCCACCTACCCAGACAGGAATGGCACAAGTGATTATACCTCAGGGGCAACTTGTGGATGTGAATAGTCCTCGGGATGTCCCTGAAGAGAAACCCAGTAACAGAAACTTACCAACTGTAAGAGTGGATACTCTAGCAGACAATACCAGCAATTACATTCTTCATCCTCAAACATCCTTCCCATTGCCCAAAAAGTCAGTGACCGG |
| hsa_circ_0059664 | GTTGGGGTGACTCAGTGGGAACGCCATCTGAGCGGGGCATGACCTATGACGCACTCCACGTTTTTGACTGGATCAAAGCAAGAAGTGGTGACAACCCCGTGTACATCTGGGGCCACTCTCTGGGCACTGGCGTGGCGACAAATCTGGTGCGGCGCCTCTGTGAGCGAGAGACGCCTCCAGATGCCCTTATATTGGAATCTCCATTCACTAATATCCGCGAAGAAGCTAAGAGCCATCCATTTTCAGTG |
| hsa_circ_0059665 | GCACACCGTCCCTGCAGTCTGGTGGAAGAACGCCCAAGGCAAAGACCAGATGTGGTATGAGGATGCCTTGGCTTCCAGCCACCCTATCATTCTGTACCTGCATGGGAACGCAGGTACCAGAGGAGGCGACCACCGCGTGGAGCTTTACAAGGTGCTGAGTTCCCTTGGTTACCATGTGGTCACCTTTGACTACAGAGGTTGGGGTGACTCAGTGGGAACGCCATCTGAGCGGGGCATGACCTATGACGCACTCCACGTTTTTGACTGGATCAAAGCAAGAAGTGGTGACAACCCCGTGTACATCTGGGGCCACTCTCTGGGCACTGGCGTGGCGACAAATCTGGTGCGGCGCCTCTGTGAGCGAGAGACGCCTCCAGATGCCCTTATATTGGAATCTCCATTCACTAATATCCGCGAAGAAGCTAAGAGCCATCCATTTTCAGTG |
| hsa_circ_0060295 | ATGAGGACGCTGAGGCCCAGAGAGGGAAAGCCACTTGCCTAGGGACACACAGCGGGGAGAGGTGGAGCAGGGCCTCTATTTCGAGACCCCTGACTCCACACCTGGTGTTTGTGCCAAGACCCCAGGCTGCCTCCCAGGTCCTCTGGGACAGCCCCTGCCTTCTACCAG |
| hsa_circ_0060558 | AGCCCAGCAGTGATTGACTCCCCATTGAAGCTGGAGCTGCGGGTCCTGGCCCCACCGCGCTGCACCATCAAGCCCTCTGGCACCACCATCTCTGTCACTGCTAGCGTCACCATTGCCCTGGTCCCACCAGACCAGCCTGAGGTCCAGCTGTCCAGCATGACTATGGACGCCCGTCTCAGCGCCAAGATGGCTCTCCGGGGGAAGGCCCTGCGCACGCAGCTGGACCTGCGCAG |
| hsa_circ_0060990 | ATGGTCTGCGAGCAGCCGGAGGTCTTTGCTTCCGCCTGTGCCCTGGCCCGGGCCTTCCCGCTGTTCACCCACCGCTCAGGTGCCTCTCGGCGCTTGGAGAAGAAGACGGTCACCGTGGAGTTTTTCCTGGTGGGACAAGACAACGGGCCGGTGGAGGTGTCCACATTGCAGTGCTTAGCGAATGCCACAGACGGCGTGCGGCTAGCAGCCCGCATCGTGGACACACCCTGCAATGAGATGAACACCGACACCTTCCTCGAGGAGATTAACAAAGTTGGAAAGGAGCTGGGGATCATCCCAACCATCATCCGGGATGAGGAACTGAAGACGAGAGGATTTGGAGGAATCTATGGGGTTGGCAAAGCCGCCCTGCATCCCCCAGCCCTGGCCGTCCTCAGCCACACCCCAGATGGAGCCACGCAGACCATCGCCTGGGTGGGCAAAGGCATCGTCTATGACACTGGAGGCCTCAGCATCAAAGGGAAG |
| hsa_circ_0061276 | GAAGTGTTTGGATTGTGAGCTATTTCAGAACTGTTCTCAGGACTCATTATTTTAACATTTGGGAGAAACACAGCCAGAAG |
| hsa_circ_0063324 | TATGAGGTTGATGAGCTACGCCGAAAGAAGGAGATTACAGTGAGGGGGGGAGATGTTTGTCCTAAACCCGTGTTTGCCTTCCATCATGCTAACTTCCCACAATATGTAATGGATGTGTTGATGGATCAGCACTTTACAGAACCAACTCCAATTCAGTGCCAGGGATTTCCGTTGGCTCTTAGTGGCCGGGATATGGTGGGCATTGCTCAGACTGGCTCTGGGAAGACGTTGGCGTATCTCCTGCCTGCAATTGTTCATATTAACCACCAGCCATACTTGGAAAGGGGAGATGGCCCAATCTGTCTAGTTCTGGCTCCTACCAGAGAGCTTGCCCAGCAAGTACAGCAGGTGGCCGATGACTATGGCAAATGTTCTAGATTGAAGAGTACTTGTATTTATGGAGGTGCTCCTAAAGGTCCCCAGATTCGAGACTTGGAAAGAGGTGTTGAGATCTGCATAGCCACTCCTGGACGTCTGATAGATTTCCTGGAGTCAGGAAAGACAAATCTTCGCCGATGTACTTACCTTGTATTGGACGAAGCTGACAGAATGCTTGATATGGGGTTTGAACCCCAGATCCGTAAAATTGTTGACCAAATCAGGCCTGATAGGCAGACACTGATGTGGAGTGCAACCTGGCCAAAAGAAGTAAGACAGCTTGCAGAGGATTTCCTTCGTGATTACACCCAGATCAACGTAGGCAATCTGGAGTTGAGTGCCAACCACAACATCCTCCAGATAGTGGATGTCTGCATGGAAAGTGAAAAAGACCACAAGTTGATCCAACTAATGGAAGAAATAATGGCTGAAAAGGAAAACAAAACAATAATATTTGTGGAGACAAAGAGACGCTGTGATGATCTGACTCGAAGGATGCGCAGAGATGGTTGGCCAGCTATGTGTATCCATGGAGACAAGAGTCAACCAGAAAGAGATTGGGTACTTAATG |
| hsa_circ_0064031 | GCTGAGAAGTTTGGCGACTCCTTTGTCTTTGAAGGCATGTTGAGTGAGCAAGTGAAGACCAATATTCAACAGGCAGTTGCAGCTGCTCCCTGGTGGTTACCTGTGAAAGGCGCTAACTGGAGACACCCAGAAGGGCCTGACTCTACTATTCTGCACAGGCCGGATCATCCAGTTCTCCATGTGTCCTGGAATGATGCGGTTGCCTACTGCACTTGGGCAGGGAAGCGGCTGCCCACGGAAGCTGAGTGGGAATACAGCTGTCGAGGAGGCCTGCATAATAGACTTTTCCCCTGGGGCAACAAACTGCAGCCCAAAGGCCAGCATTATGCCAACATTTGGCAGGGCGAGTTTCCGGTGACCAACACTGGTGAGGATGGCTTCCAAGGAACTGCGCCTGTTGATGCCTTCCCTCCCAATGGTTATGGCTTATACAACATAGTGGGGAACGCATGGGAATGGACTTCAGACTGGTGGACTGTTCATCATTCTGTTGAAGAAACGCTTAACCCAAAAGGTCCCCCTTCTGGGAAAGACCGAGTGAAGAAAGGTGGATCCTACATGTGCCATAGG |
| hsa_circ_0064338 | AAATGACCATGGTTGACACAGAGATGCCATTCTGGCCCACCAACTTTGGGATCAGCTCCGTGGATCTCTCCGTAATGGAAGACCACTCCCACTCCTTTGATATCAAGCCCTTCACTACTGTTGACTTCTCCAGCATTTCTACTCCACATTACGAAGACATTCCATTCACAAGAACAGATCCAGTGGTTGCAGATTACAAGTATGACCTGAAACTTCAAGAGTACCAAAGTGCAATCAAAGTGGAGCCTGCATCTCCACCTTATTATTCTGAGAAGACTCAGCTCTACAATAAGCCTCATGAAGAGCCTTCCAACTCCCTCATGGCAATTGAATGTCGTGTCTGTGGAGATAAAGCTTCTGGATTTCACTATGGAGTTCATGCTTGTGAAGGATGCAAG |
| hsa_circ_0066783 | GAGAGACAGCGTCTGGAGACCATCCTCAGTCTCTGTGCTGAATACACAAAGCCTGACAGTCGCTTATCTACTGGGACCACCGTGGAAGATGTGCAGAAAATCAACAAGGAGCTTGAGAAGCTGCAGCTCTCTGATGAGGAGTCTGTGTTTGAGGAAGCCCTCATGAGCCCTGACACAAGATACAGGTGCCACCGGAAAGACTCCCTCCCTGATGCAGACTTGGCAAGCTGTGGGAGTCTCAGTCAGAGCAGTGCCAGCTTCTTTACCCCCAGGAGCACCAGGAATGATGAACTACTCAGTGACCTCACCCGGACTCCTCCACCACCATCCTCCACCTTTCCGAAAGCTTCCAGCGAGTCCTCTTATCTAAGTATCCTACCAAAG |
| hsa_circ_0066916 | GACCCATCCATTTTCACAGTATTGACTGCTAAGTCTGTCCGCCCTGGAGTGGCCATTGCTGATTTTGTCATCTTCCCACCTCGATGGGGGGTTGCTGATAAGACCTTCAGGCCTCCTTATTACCATA |
| hsa_circ_0067384 | ATCCTCTGCCTTGTCAATGTTAGTCACAATGGTGTGAGTGAATCAGAACTGATGGAACTCTATCCTGAGATGTCCTGGACTTTCTTGACCTCCCTTATTCACAGTTTATACAAAATGTGTTTGTTGACTTATGGATGTGGCTTGCTTAGGTTTCAACATCTGCAGGCTTGGGAAACAGTGAGATTGGAGTACCTGGAAGGCCCCACTGTTACTTCTTCATACAGGCAAAAGCTAATCAACTATTTCACCTTGCAGCTAAGTCAGGACAGAGTGACTTGGAGAAGTGCAGATGAACTCCCGTGGCTTTTTCAGCAGCAGGGAAGTAAACAGAAGCTGCATGATTGCCTTCTTAATCTCTTTGTGTCTCAAAACCTTTATAAAAGGGGACACTTTGCTGAGTTGCTGAGTTATTGGCAGTTTGTTGGCAAAGACAAAAGTGCAATGGCAACAGAATACTTCGATTCATTGAAGCAGTATGAGAAAAACTGCGAAGGCGAGGACAACATGAGTTGCTTAGCTGATCTTTATGAAACCTTGGGGCGATTTCTCAAGGATCTAGGCCTTCTCAGTCAGGCCATAGTACCTTTGCAGAGGTCTTTAGAGATTCGAGAAACAGCTTTAGATCCCGATCACCCAAGAGTAGCCCAGTCCCTCCACCAACTAGCAAGTGTATACGTGCAGTGGAAGAAGTTTGGCAATGCAGAACAACTGTATAAACAGGCGTTGGAAATCTCAGAAAATGCTTATGGTGCGGACCATCCATATACTGCTCGTGAACTTGAAGCACTTGCAACTTTGTACCAGAAACAAAATAAATATGAACAAGCTGAACATTTTAGGAAAAAATCCTTTAAAATTCATCAGAAAGCTATAAAGAAAAAAGGCAACTTGTACGGATTTGCCCTTTTACGTAGACGGGCTTTACAGTTAGAAGAGCTTACATTAGGTAAGGACACACCTGATAATGCTCGGACCCTCAATGAACTGGGTGTTCTCTACTATCTTCAAAATAACCTGGAAACAGCTGACCAGTTTCTGAAGCGTTCCTTAGAAATGAGGGAGCGAGTTCTAGGACCAGATCACCCTGACTGTGCTCAGTCTTTGAATAATCTGGCAGCTCTATGCAATGAAAAGAAACAGTATGATAAAGCAGAAGAACTTTATGAAAGAGCTTTAGATATTCGGAGACGTGCATTAGCTCCTGATCACCCTTCTTTGGCATATACGGTGAAGCATCTTGCCATCTTGTATAAGAAAATGGGGAAACTTGACAAAGCTGTACCTTTGTATGAATTGGCTGTTGAAATTCGACAGAAATCTTTTGGCCCAAAGCACCCTAGTGTAGCTACTGCCTTGGTGAACTTAGCTGTTCTTTATAGCCAAATG |
| hsa_circ_0068032 | ATGAAAGTTTTAGACAAAGCCGATCAAACCTCACCTCTCTATTAGTGCAGCCCATCTCTGCACCCCTCGTTGCAAAACTGATCTCTTCGCCAAAAGCTAGAACCAAAAATGAAGCGTGTAGCTCTCTAGAGCTTCCAAATAATGAAATAAGAGTCGTCAGCATGCAAGTTCAGACAGTCACAAAATTGAAAACAGTTACTAATGTTGTTGGATTTGTAATGGGCTTGACATCTCCAGACCGGTATATCATAGTTGGCAGCCATCATCACACTGCACACAGTTATAATGGACAAGAATGGGCCAGTAGTACTGCAATAATCACAGCGTTTATCCGTGCCTTGATGTCAAAAGTTAAGAGAGGGTGGAGACCAGACCGAACTATTGTTTTCTGTTCTTGGGGAGGAACAGCTTTTGGCAATATTGGCTCATATGAATGGGGAGAGGATTTCAAGAAGGTTCTTCAGAAAAATGTTGTGGCTTATATTAGCCTCCACAGTCCCATAAGGGGGAACTCTAGTCTGTATCCTGTAGCATCACCATCTCTTCAGCAACTGGTAGTAGAG |
| hsa_circ_0069570 | GTTGGAGTTGCTGTGGGTGAGCTGCTGTGGTCTGTAGCCAAGCATGCTGTGGTCGGATCTGCCCAGCCGTGGAACAGAAACATTTGCTGGATGGAAAATCCATAAAAGAAAGCTCCTGTGAAAAGCTGAGGCTGACAATAATTTAAGCAAAATCAG |
| hsa_circ_0069718 | ATTTTCAGCTGAACTCTCATCTCTCAACACTGGCAAATATTCATAAGATCTACCACACCCTTAATAAGCTGAACCTAACAGAAGACATTGGCCAAGACGATCACCAAACAGGAAGTCTGCGGTCTTGCAGTTCTTCAGACTGCTTTAATAAAGTGATGCCACCAAGGAAAAAGAGAAGACCTGCCTCTGGAGATGATTTATCTGCCAAGAAAAGTAGACATGATAGCATGTATAGAAAATATGATTCGACTAGAATAAAGACTGAAGAAGAAGCCTTTTCAAGTAAAAGGTGCTTGGAATGGTTCTATGAATATGCAGGAACTGATGATGTTGTAGGCCCTGAAGGCATGGAGAAATTTTGTGAAGACATTGGTGTTGAACCAGAAAACGTAGTTATGCTTGTCCTAGCTTGGAAATTGGATGCACAAAACATGGGTTATTTTACTCTACAGGAGTGGTTAAAAGGAATGACTTCTCTCCAATGTGATACAACAGAAAAACTCAGAAATACTTTGGATTACTTAAGATCATTCTTAAATGATTCTACAAACTTTAAACTTATTTACAGATATGCGTTTGACTTTGCACGG |
| hsa_circ_0070039 | GTGGGTTTGGAGGATTTGGGACAACATCTACAACTGCAGGTTCTGCATTCAGCTTTTCTGCCCCAACTAACACAGGCACTACTGGACTCTTTGGTGGTACTCAGAACAAAGGTTTTGGATTTGGTACTGGTTTTGGCACAACAACGGGAACTAGTACTGGTTTAGGTACTGGTTTGGGAACTGGACTGGGATTTGGAGGATTTAATACACAGCAGCAGCAGCAAACTACATTAGGTGGTCTCTTCAGTCAGCCTACACAAGCTCCTACCCAGTCCAACCAGCTGATAAATACTGCGAGTGCTCTTTCTGCTCCAACGCTGTTGGGAGATGAGAGAGATGCTATTTTGGCAAAATGGAATCAACTGCAGGCCTTTTGGGGAACAGGAAAAGGGTATTTCAACAATAATATTCCGCCAGTGGAATTCACACAAGAAAATCCCTTTTGCCGATTTAAGGCAGTAGGTTATAGTTGCATGCCCAGTAATAAAGATGAAGATGGGCTAGTGGTTTTAGTTTTCAACAAAAAAGAAACAGAGATTCGAAGCCAACAACAACAGTTGGTAGAATCATTGCATAAAGTTTTGGGAGGAAACCAGACCCTTACTGTAAATGTAGAGGGCACTAAAACATTGCCAGATGATCA |
| hsa_circ_0070467 | AGCATCTGCTGCACCCAAGCCTGAGCCGGTTCCTGTTCAAAAGGGAGAACCTAAAGAAGTAGTTAAACCTGTGCCCATTACATCTCCTGCTGTGTCCAAAGTCACTTCCACAAACAACATGGCCTACAATAAGGCACCACGGCCTTTTGGTTCTGTGTCTTCACCAAAAGTCACATCCATCCCATCACCATCGTCTGCCTTCACCCCAGCCCATGCGACCACCTCATCACATGCTTCCCCTTCACCCGTGGCTGCCGTCACTCCTCCCCTGTTCGCTGCATCTGGACTGCATGCTAATGCCAATCTTAGTGCTGACCAGTCTCCATCTGCACTGAGCGCTGGTAAAACTGCAGTTAATGTCCCACGGCAGCCCACAGTCACCAGCGTGTGTTCCGAGACTTCTCAGGAGCTAGCAGAGGGACAGAGAAGAGGATCCCAGGGTGACAGTAAACAGCAAAATGGCCCACCAAGAAAACACATTGTGGAGCGCTATACAGAGTTTTATCATGTACCCACTCACAGTGATGCCAGCAAGAAGAGACTGATTGAGGATACTGAAGACTGGCGTCCAAGGACTGGAACAACTCAGTCTCGCTCTTTCCGAATCCTTGCCCAGATCACTGGGACTGAACATTTGAAAGAATCTGAAGCCGATAATACAAAGAAGGCAAA |
| hsa_circ_0071099 | GTCTGTCAGTGGCCCAGCGGAAGTTTGCTCATTCACTCAGAGACTTTAAGTTTGAGTTTATCGGTGATGCTGTGACAGATGATGAACGATGCATAGATGCTTCCTTACGTGAATTTTCAAATTTTTTGAAGAATCTGGAGGAACAGAGAGAAATTATG |
| hsa_circ_0072022 | GGCTGGTGAATTACCAGATCTCCGTCAAGTGCAGTAACCAGTTCAAGTTGGAAGTGTGTCTTTTGAAATCAGAAAACAAAGTCGTGGACAACCAGGCTGGGACCCAGGGCCAGCTGAAGGTGCTGGGTGCCAACCTCTGGTGGCCGTACCTGATGCACGAACACCCCGCCTACCTGTACTCGTGGGAG |
| hsa_circ_0072023 | GGCTGGTGAATTACCAGATCTCCGTCAAGTGCAGTAACCAGTTCAAGTTGGAAGTGTGTCTTTTGAAATCAGAAAACAAAGTCGTGGACAACCAGGCTGGGACCCAGGGCCAGCTGAAGGTGCTGGGTGCCAACCTCTGGTGGCCGTACCTGATGCACGAACACCCCGCCTACCTGTACTCGTGGGAGGATGGTGATTGCTCACACCAAAGCCTTGGACCCCTCCCAGCCTGTGACCTTTGTGACCAACTCCACCTACGCAGCAGACAAGGGG |
| hsa_circ_0072309 | GACTGACTGCATTGCACAGATGATGGATATTTACGTATGTTTGAAACGACCATCCTGGATGGTGGACAATAAAAGAATGAGGACTGCTTCAAATTTCCAGTGGCTGTTATCAACATTTATTCTTCTATATCTAATGAATCAAGTAAATAGCCAGAAAAAGGGGGCTCCTCATGATTTGAAGTGTGTAACTAACAATTTGCAAGTGTGGAACTGTTCTTGGAAAGCACCCTCTGGAACAGGCCGTGGTACTGATTATGAAGTTTGCATTGAAAACAGGTCCCGTTCTTGTTATCAGTTGGAGAAAACCAGTATTAAAATTCCAGCTCTTTCACATGGTGATTATGAAATAACAATAAATTCTCTACATGATTTTGGAAGTTCTACAAGTAAATTCACACTAAATGAACAAAACGTTTCCTTAATTCCAGATACTCCAGAGATCTTGAATTTGTCTGCTGATTTCTCAACCTCTACATTATACCTAAAGTGGAACGACAGGGGTTCAGTTTTTCCACACCGCTCAAATGTTATCTGGGAAATTAAAGTTCTACGTAAAGAGAGTATGGAGCTCGTAAAATTA |
| hsa_circ_0072799 | GGCTGGTGAATTACCAGATCTCCGTCAAGTGCAGTAACCAGTTCAAGTTGGAAGTGTGTCTTTTGAATGCAGAAAACAAAGTCGTGGACAACCAGGCTGGGACCCAGGGCCAGCTGAAGGTGCTGGGTGCCAACCTCTGGTGGCCGTACCTGATGCACGAACACCCCGCCTACCTGTACTCGTGGGAGGATGGTGATTGCTCACACCAAAGCCTTGGACCCCTCCCAGCCTGTGACCTTTGTGACCAACTCCACCTACGCAGCAGACAAGGGG |
| hsa_circ_0072838 | GGCTGGTGAATTACCAGATCTCCGTCAAGTGCAGTAACCAGTTCAAGTTGGAAGTGTGTCTTTTGAATGCAGAAAACAAAGTCGTGGACAACCAGGCTGGGACCCAGGGCCAGCTGAAGGTGCTGGTGCCAACCTCTGGTGGCCGTACCTGATGCACGAACACCCCGCCTACCTGTACTCGTGGGAGGATGGTGATTGCTCACACCAAAGCCTTGGACCCCTCCCAGCCTGTGACCTTTGGGACCAACTCCACCTACGCAGCAGACAAGGCG |
| hsa_circ_0072841 | GGCTGGTGAATTACCAGATCTCCGTCAAGTGCAGTAACCAGTTCAAGTTGGAAGTGTGTCTTTTGAATGCAGAAAACAAAGTCGTGGACAACCAGGCTGGGACCCAGGGCCAGCTGAAGGTGCTGGTGCCAACCTCTGGTGGCCGTACCTGATGCACGAACACCCCGCCTACCTGTACTCGTGGGAG |
| hsa_circ_0072847 | GGCTGGTGAATTACCAGATCTCCGTCAAGTGCAGTAACCAGTTCAAGTTGGAAGTGTGTCTTTTGAATGCAGAAAACAAAGTCGTGGACAACCAGGCTGGGACCCAGGGCCAGCTGAAGGTGCTGGGTGCCAACCTCTGGTGGCCGTACCTGATGCACGAACACCCCGCCTCCCTGTACTCGTGGGAG |
| hsa_circ_0072877 | GGCTGGTGAATTACCAGATCTCCGTCAAGTGCAGTAACCAGTTCAAGTTGGAAGTGTGTCTTTTGAATGCAGAAAACAAAGTCGTGGACAACCAGGCTGGGACCCAGGGCCAGCTGAAGGTGCTGGGTGCCAACCTCTGGTGGCCGTACCTGATGCACGAACACCCCGCCTACCTGTACTCGTGGGAGGATGGTGATTGCTCACACCAAAGCCTTGGACCCCTCCCAGCCTGTGACCTTTGGGACCAACTCCACCTACGCAGCAGACAAGGGGGCTCTGTATGTGGATGTGATCCGTGTGAACAGCTACTACTCTTGGTATCGCAACTACGGGCACCTGGAGTTGATTCGGCTGCAGCTGGCCGCCCAGTTTGAGAATTGGTGTAAGACATCACAATCCCATTATTCAGAGCGCGTATGGAGTGGAAACGCTTGTAGGGCTTCACCAGGATCCACCTCTGATGTTCAGTGAAGAGGACCAGAAAAGTCTGCTAGAGCAGTACCATCTGGGTCTGGATCAAAAACGCAGAAAATACGTGGTTGGAGAGCTCATCTGGAATTTTGCCGATTTCATGACTAACCAGT |
| hsa_circ_0072880 | GGCTGGTGAATTACCAGATCTCCGTCAAGTGCAGTAACCAGTTCAAGTTGGAAGTGTGTCTTTTGAATGCAGAAAACAAAGTCGTGGACAACCAGGCTGGGACCCAGGGCCAGCTGAAGGTGCTGGGTGCCAACCTCTGGTGGCCGTACCTGATGCACGAACACCCCGCCTACCTGTACTCGTGGGAGGATGGTGATTGCTCACACCAAAGCCTTGGACCCCTCCCAGCCTGTGACCTTTGGGACCAACTCCACCTACGCAGCAGACAAGGGG |
| hsa_circ_0072881 | GGCTGGTGAATTACCAGATCTCCGTCAAGTGCAGTAACCAGTTCAAGTTGGAAGTGTGTCTTTTGAATGCAGAAAACAAAGTCGTGGACAACCAGGCTGGGACCCAGGGCCAGCTGAAGGTGCTGGGTGCCAACCTCTGGTGGCCGTACCTGATGCACGAACACCCCGCCTACCTGTACTCGTGGGAG |
| hsa_circ_0073788 | ATATTGATGAGTGCATCCAGAATGGGGTTCTTTGTAAAAACGGTCGATGCGTGAACACAGATGGAAGTTTCCAGTGCATTTGCAATGCCGGCTTTGAATTAACTACAGATGGAAAAAACTGTGTTGATCATGATGAATGTACAACTACCAACATGTGTTTGAATGGAATGTGCATCAATGAAGATGGCAGCTTCAAGTGCATCTGCAAACCAGGATTTGTCTTGGCTCCAAATGGGCGTTACTGTACTGATGTTGATGAATGCCAGACCCCAGGAATCTGCATGAATGGGCACTGCATCAACAGTGAAGGGTCCTTCCGCTGTGACTGTCCCCCAGGCCTGGCTGTGGGCATGGATGGACGTGTGTGTGTTGATACTCACATGCGCAGTACCTGCTATGGAGGAATCAAGAAAGGAGTGTGTGTGCGTCCTTTCCCCGGTGCAGTGACCAAGTCCGAATGCTGCTGTGCCAATCCAGACTATGGTTTTGGAGAACCCTGCCAGCCATGCCCTGCAAAAAATTCAGCTGAATTCCACGGCCTTTGTAGTAGTGGAGTAGGTATCACTGTGGATGGAAGAGATATCAATGAATGTGCTTTGGATCCTGATATATGTGCCAATGGGATTTGTGAAAACTTACGTGGTAGTTACCGTTGTAATTGCAACAGTGGCTATGAACCAGATGCCTCTGGAAGAAACTGTATTGACATTGATGAATGTTTAGTAAACAGACTGCTTTGTGATAACGGATTGTGCCGAAACACGCCAGGAAGTTACAGCTGTACGTGCCCACCAGGGTATGTGTTCAGGACTGAGACAGAGACCTGTGAAGATATAAATGAATGTGAAAGCAACCCATGTGTCAATGGGGCCTGCAGAAACAACCTTGGATCTTTCAATTGTGAATGTTCGCCCGGCAGCAAACTCAGCTCCACAGGATTGATCTGTATTGACAGCCTGAAGGGGACCTGTTGGCTCAACATCCAGGACAGCCGCTGTGAGGTGAATATTAATGGAGCCACTCTGAAATCTGAATGCTGTGCCACCCTCGGAGCCGCCTGGGGGAGCCCCTGTGAGCGGTGTGAACTAGATACAGCTTGCCCAAGAGGGCTTGCCAGGATTAAAGGTGTTACGTGTGAAGATGTTAATGAGTGTGAGGTGTTCCCTGGCGTTTGTCCAAATGGACGCTGTGTCAACAGTAAGGGATCTTTTCATTGCGAGTGCCCTGAAGGCCTTACGTTGGATGGGACTGGCCGTGTATGTTTGGATATTCGCATGGAGCAGTGTTACTTGAAGTGGGATGAAGATGAATGCATCCACCCCGTTCCTGGAAAGTTCCGCATGGATGCCTGCTGCTGTGCTGTCGGGGCGGCTTGGGGCACCGAGTGTGAGGAGTGCCCCAAACCTGGCACCAAGGAATACGAGACGCTGTGCCCCCGCGGGGCTGGCTTTGCTAACCGAGGGGATGTTCTTACTGGGCGGCCATTTTACAAAGACATCAATGAATGCAAAGCATTTCCTGGGATGTGCACTTATGGGAAGTGCAGAAATACAATCGGAAGCTTCAAATGCCGTTGCAATAGTGGCTTTGCTCTAGACATGGAGGAAAGAAACTGCACGGACATCGACGAGTGCAGGATTTCTCCTGACCTCTGTGGCAGTGGAATCTGCGTCAATACACCGGGCAGCTTTGAGTGCGAGTGCTTCGAAGGCTATGAAAGTGGCTTCATGATGATGAAGAACTGCATGGACATTGACGAATGTGAACGTAACCCTCTCCTTTGTAGGGGTGGCACCTGTGTGAACACTGAGGGCAGCTTTCAGTGTGACTGCCCACTGGGACACGAGCTGTCACCATCCCGTGAGGACTGTGTGGATATTAATGAATGCTCCCTGAGTGACAATCTCTGCAGAAATGGAAAATGTGTGAACATGATTGGAACCTATCAGTGCTCTTGCAATCCTGGATATCAGGCTACGCCAGACCGCCAGGGCTGTACAGATATTGATGAATGTATGATAATGAACGGAGGCTGTGACACCCAGTGCACAAATTCAGAGGGAAGCTACGAATGCAGCTGCAGTGAGGGTTATGCCCTGATGCCAGATGGGAGATCGTGTGCAGACATTGATGAATGTGAAAACAATCCTGATATCTGTGATGGCGGCCAGTGTACCAACATTCCTGGAGAGTATCGCTGCCTCTGCTATGATGGCTTCATGGCTTCCATGGACATGAAAACATGCATTGATGTCAATGAATGTGACCTAAATTCAAATATCTGCATGTTTGGGGAATGTGAGAACACAAAGGGATCCTTCATTTGCCACTGTCAGCTGGGTTACTCAGTGAAGAAGGGGACCACAGGATGTACAGATGTGGATGAGTGTGAAATTGGTGCTCATAACTGCGACATGCATGCCTCATGTCTGAATATCCCAGGAAGCTTCAAGTGTAGCTGCAGAGAAGGCTGGATTGGAAACGGCATCAAGTGTATTGATCTGGACGAATGTTCTAATGGAACCCACCAGTGTAGCATCAATGCTCAGTGTGTAAATACCCCGGGCTCATACCGCTGTGCCTGCTCCGAAGGTTTCACTGGTGATGGCTTTACCTGCTCAGATGTTGATGAGTGTGCAGAAAACATAAACCTCTGTGAGAACGGACAGTGCCTTAATGTCCCGGGTGCATATCGCTGCGAGTGTGAGATGGGCTTCACTCCAGCCTCAGACAGCAGATCCTGCCAAGATATTGATGAATGCTCCTTCCAAAACATTTGTGTCTTTGGAACATGTAATAACCTGCCTGGAATGTTTCATTGCATCTGCGATGATGGTTATGAATTGGACAGAACAGGAGGGAACTGTACAGATATTGATGAGTGTGCAGATCCTATAAACTGTGTCAATGGCCTATGTGTCAACACGCCTGGTCGCTATGAGTGTAACTGCCCACCCGATTTTCAGTTGAACCCAACTGGTGTGGGTTGTGTTGACAACCGTGTGGGCAACTGCTACCTGAAGTTTGGACCTCGAGGAGATGGGAGTCTGTCTTGCAACACCGAGATCGGGGTGGGCGTCAGTCGCTCTTCATGCTGCTGCTCTCTGGGAAAGGCCTGGGGAAACCCCTGTGAGACATGCCCCCCTGTCAATAGCACTGAATATTACACCCTGTGTCCCGGAGGTGAAGGCTTCAGACCTAACCCCATCACAATCATTTTAGAAGACATTGACGAATGCCAGGAGTTACCAGGTCTCTGCCAGGGTGGAAACTGCATCAACACTTTTGGGAGCTTCCAGTGTGAGTGCCCACAAGGCTACTACCTCAGCGAGGATACCCGCATCTGTGAAGATATTGATGAGTGTTTTGCACATCCTGGTGTGTGTGGGCCTGGGACCTGCTATAACACCCTGGGAAATTACACCTGCATTTGCCCACCTGAGTACATGCAGGTCAATGGAGGCCACAACTGCATGGACATGAGAAAAAGCTTTTGCTACCGAAGCTATAATGGAACCACTTGTGAGAATGAGTTGCCTTTCAATGTGACAAAAAGGATGTGCTGCTGCACATATAATGTGGGCAAAGCCTGGAACAAACCTTGTGAACCATGCCCAACTCCAGGAACAGCTGACTTTAAAACCATATGTGGAAATATTCCTGGATTCACCTTTGACATTCACACAGGAAAAGCTGTTGACATTGATGAATGTAAAGAGATTCCAGGCATTTGTGCAAATGGTGTGTGCATTAACCAGATTGGCAGTTTCCGCTGTGAATGCCCTACAGGATTCAGTTACAATGACCTGCTGTTGGTTTGTGAAG |
| hsa_circ_0073816 | CTCTCCGAGATGCTGGCAAACAGTCTATTAATAGTGACTGGAAGATTGAACACTCTGGAGCCTTCAATTTGGCTGGAACTACCGTTCATTATGTAAGACGAGGCCTCTGGGAGAAGATCTCTGCCAAAGGTCCTACTACAGCACCTTTACATCTTCTGGTGCTCCTGTTTCAGGATCAGAATTATGGTCTTCACTATGAATACACTATCCCATCAGACCCTCTTCCAGAAAACCAGAGCTCTAAAGCACCTGAGCCCCTCTTCATGTGGACACACACAAGCTGGGAAGATTGCGATGCCACTTGTGGAGGAGGAGAAAGGAAGACAACAGTGTCCTGCACAAAAATCATGAGCAAAAATATCAGCATTGTGGACAATGAGAAATGCAAATACTTAACCAAGCCAGAGCCACAGATTCGAAAGTGCAATGAGCAACCATGTCAAACAAGGTGGATGATGACAGAATGGACCCCTTGTTCACGAACTTGTGGAAAAGGAATGCAGAGCAGACAAGTGGCCTGTACCCAACAACTGAGCAATGGAACACTGATTAGAGCCCGAGAGAGGGACTGCATTGGGCCCAAGCCCGCCTCTGCCCAGCGCTGTGAGGGCCAGGACTGCATGACCGTGTGGGAGGCGGGAGTGTGGTCTGAGTGTTCAGTCAAGTGTGGCAAAGGCATACGTCATCGGACCGTTAGATGTACCAACCCAAGAAAGAAGTGTGTCCTCTCTACCAGACCCAGGGAGGCTGAAGACTGTGAGGATTATTCAAAATGCTATGTGTGGCGAATGGGTGACTGGTCTAAG |
| hsa_circ_0073867 | AAACTCGGCAGTGATGCTCAAGTTAAAGTCTTTGGGAAATGCTGCCAACTGAAACCTGGAGGAGACAGTTCTTCCTCTTTAGATAGTTCTGTGACTTCATCTTCTGATATAAAAGACCAGTGTCTTAAGTACCAG |
| hsa_circ_0074598 | GACCTATACGAGGCAGGGGAACTGAAATGGGGAACAGATGAAGCCCAGTTCATTTACATCTTGGGAAATCGCAGCAAGCAGCATCTTCGGTTGGTGTTCGATGAGTATCTGAAGACCACAGGGAAGCCGATTGAAGCCAGCATCCGAGGGGAGCTGTCTGGGGACTTTGAGAAGCTAATGCTGGCCGTAGTGAAGTGTATCCGGAGCACCCCGGAATATTTTGCTGAAAGGCTCTTCAAGGCTATGAAGGGCCTGGGGACTCGGGACAACACCCTGATCCGCATCATGGTCTCCCGTAGTGAGTTGGACATGCTCGACATTCGGGAGATCTTCCGGACCAAGTATGAGAAGTCCCTCTACAGCATGATCAAGAATGACACCTCTGGCGAGTACAAGAAGACTCTGCTGAAGCTGTCTGGGGGAGATGATGATGCTGCTGGCCAGTTCTTCCCGGAGGCAGCGCAGGTGGCCTATCAGATGTGGGAACTTAGTGCAGTGGCCCGAGTAGAGCTGAAGGGAACTGTGCGCCCAGCCAATGACTTCAACCCTGACGCAGATGCCAAAGCGCTGCGGAAAGCCATGAAGGGACTCGGGACTGACGAAGACACAATCATCGATATCATCACGCACCGCAGCAATGTCCAGCGGCAGCAGATCCGGCAGACCTTCAAGTCTCACTTTGGCCGGGACTTAATGACTGACCTGAAGTCTGAGATCTCTGGAGACCTGGCAAGGCTGATTCTGGGGCTCATGATGCCACCGGCCCATTACGATGCCAAGCAGTTGAAGAAGGCCATGGAGGGAGCCGGCACAGATGAAAAGGCTCTTATTGAAATCCTGGCCACTCGGACCAATGCTGAAATCCGGGCCATCAATGAGGCCTATAAGGAGGACTATCACAAGTCCCTGGAGGATGCTCTGAGCTCAGACACATCTGGCCACTTCAGGAGGATCCTCATTTCTCTGGCCACGGGGCATCGTGAGGAGGGAGGAGAAAACCTGGACCAGGCACGGGAAGATGCCCAG |
| hsa_circ_0075026 | AGCAAGGCTTAGATCACATAGCAGAAAACATTCTTTCGTACCTGGATGCCAGGTCTCTGTGTGCAGCAGAGCTGGTATGTAAAGAATGGCAGCGAGTGATCTCAGAAGGAATGCTTTGGAAGAAGCTGATTGAACGAATGGTACGCACTGATCCCCTATGGAAAGGACTTTCAGAAAGAAGAGGGTGGGATCAGTACCTGTTTAAAAACAGACCCACAGATGGCCCTCCAAATTCATTTTATAGGTCATTATACCCAAAGATTATCCAGGATATAGAGACTATAGAATCTAACTGGCGGTGTGGACGACACAACTTGCAGAGGATTCAGTGCCGCTCTGAAAATAGTAAAGGTGTCTACTGTTTACAGTACGATGATGAAAAAATTATCAGTGGCCTACGAGATAATTCTATTAAGATATGGGATAAAACCAGCCTGGAATGTTTGAAAGTGTTAACAGGACACACAGGCTCTGTCCTCTGTCTGCAGTATGATGAGCGTGTCATTGTAACTGGCTCTTCAGATTCTACGGTGAGAGTGTGGGATGTGAACACGGGTGAAGTTCTTAACACATTGATCCACCACAATGAGGCTGTATTGCACTTACGCTTCAGCAATGGACTGATGGTGACCTGTTCCAAGGACCGCTCCATTGCTGTGTGGGACATGGCTTCTGCGACCGACATCACTTTACGCCGTGTCCTGGTTGGCCACCGGGCTGCCGTCAATGTAGTAGACTTTGACGACAAGTACATCGTGTCTGCCTCTGGTGACAGGACCATCAAAGTCTGGAGCACGAGCACCTGTGAATTTGTTCGTACTCTCAATGGGCACAAGCGGGGCATTGCCTGTCTCCAGTACAGGGATCGCCTGGTTGTTAGTGGATCATCAGATAATACCATTAGGCTCTGGGATATTGAATGTGGTGCCTGTTTAAGAGTCCTAGAGGGACATGAAGAATTGGTCCGATGCATCCGGTTTGATAACAAGAGGATTGTCAGTGGGGCCTATGATGGGAAAATTAAAGTTTGGGACTTGCAAGCTGCTCTTGACCCTCGAGCCCCAGCAAGCACATTGTGTTTGCGCACATTGGTGGAACATTCTGGACGTGTGTTTCGGCTCCAGTTTGATGAGTTTCAGATCATCAGCAGCTCCCATGATGACACTATTTTGATTTGGGATTTCTTAAATGTGCCTCCCAGTGCCCAGAATGAGACCCGTTCTCCCTCCAGAACATACACTTACATCTCTAGATAACAGTCTGCACTTTCACCCGTTTCAG |
| hsa_circ_0075116 | GTGTGTGGTCTGCTTCAGTGACTTCGAGGCGCGGCAGCTGCTCCGAGTCCTCCCCTGCAACCATGAGTTCCACACCAAGTGTGTTGACAAGTGGTTGAAG |
| hsa_circ_0075303 | GGATGAAGATGCCCCTGCTAAGATTCCAGATGAAGAGGCCACAAAACCCGAAGGCTGGTTAGATGATGAGCCTGAGTACGTACCTGATCCAGACGCAGAGAAACCTGAGGATTG |
| hsa_circ_0075322 | GGCTTCTCGCACAGCCGCTGGCTCCGGAAGGTGAAACACGGACACTTCGGGTGGCCAGGATGGGAAATGGGTCCACCAGGAAACTGGAGCCCACGTCCTCCTCGTGCAGGGGAGGCCCGCCCTGGCCCCACGGCAGAATCAGCTTCTGGTCCATCGGAGGATCCGAGTGTGAATTTCCTGAAGAACGTTGGGGAGAGTGTGGCAGCTGCCCTTAGCCCTCTGGGCATTGAAGTTGATATCGATGTGGAGCACGGAGGGAAAAGAAGCCGCCTGACCCCCGTCTCTCCAGAGAGTTCCAGCACAGAGGAGAAGAGCAGCTCACAGCCAAGCAGCTGCTGCTCTGACCCCAGCAAGCCGGGTGGGAATGTTGAGGGCGCCACGCAGTCTCTGGCGGAGCAGATGAGGAAGATCGCCTTGGAGTCCGAGGGGCGCCCTGAG |
| hsa_circ_0075346 | GGATCTGAAACTTGCCCACCCTTCGGGATATTGCAGGACGCTGCATCATGAGCGACAGTAAATGTGACAGTCAGTTTTATAGTGTGCAAGTGGCAGACTCAACCTTCACTGTCCTAAAACGTTACCAGCAGCTGAAACCAATTGGCTCTGGGGCCCAAGGGATTGTTTGTGCTGCATTTGATACAGTTCTTGGGATAAATGTTGCAGTCAAGAAACTAAGCCGTCCTTTTCAGAACCAAACTCATGCAAAGAGAGCTTATCGTGAACTTGTCCTCTTAAAATGTGTCAATCATAAAAATATAATTAGTTTGTTAAATGTGTTTACACCACAAAAAACTCTAGAAGAATTTCAAGATGTGTATTTGGTTATGGAATTAATGGATGCTAACTTATGTCAGGTTATTCACATGGAGCTGGATCATGAAAGAATGTCCTACCTTCTTTACCAGATGCTTTGTGGTATTAAACATCTGCATTCAGCTGGTATAATTCATAGAGATTTGAAGCCTAGCAACATTGTTGTGAAATCAGACTGCACCCTGAAGATCCTTGACTTTGGCCTGGCCCGGACAGCGTGCACTAACTTCATGATGACCCCTTACGTGGTGACACGGTACTACCGGGCGCCCGAAGTCATCCTGGGTATGGGCTACAAAGAGAACG |
| hsa_circ_0075407 | GTGGGAACATCCAGTTGCGGGAAAACAAGCTTAACACGCCCACTGATTCTACATTATGGTGAGTTCTATAATTATTTTATTATATATCACAGTGTAATAATGGAAATAAAGTGCCTAATAAATGCAAATGTGCTTACATCTTTTGGCCCAGCTCCTACCTCCCGGCAGCCTCTCCAGGCCCAGAACTTTCTCCAGTCAGCCTCTACAGACCAAGCTCATGACTCACAATGGCCTATTTAGGCCCATACCCTACGTCACGGCAGCCTCCGCAGATGAGCCTACTGCCTCACAACAGCCTCCACAGGCACAGCTCCATCGTTACAATGGCCTCTTTAGACCCAGCTCCTGCCTCCCAGCCTTCTCTCCAGGCTCTGAACTTTCTCAGTAAGTTCAGGTAGCTGGGACTGTAGGTATACATGATGATACTTGGCTAATTTTTAAATTGTTTTGTAGACACGGGGTCTCACTTTGTTGGCCAGGCTGGTGTCAAACTAATGGCCTCAAGTGACCCTTCCACCCCTGCCTCCCATCCTCGAGGCATGTGCCACCACAAGGAGCACTTGTTCAATTTTCTAAAAAAAAAATGTCTAAAGTAAGGCTGTGGGATGATGGCAGGAAGATAAAAGAAAAACAGAAGAATAAGTTAAAATGACTTATTCACACATATTCTTTTGACAGCAAGAAGAACTTTTAGTATGTACATTCCTTACAAACAAACAAAAGGCAGATAAACAATGTTGTATAGGAACTTCAACACACACTGTACAATATTCCCACTTTGCTGACATAAGTTATGGAAATTTCATGGTTTACTTGAGTGTCGCTACCAGTATTTTGCTTCTCTGATGATTTTTATCAACTTCCTCATCTGTTAACTTCTCTCCAAGGTATGTCATGTCACGACATACTGCCGCTGCACGAACATGGCCAGTGTCTTCCTATTCAACATGTAGAATGCTTTCCTAATTTCTCTTTTTACTCTCTGTCTTTGTGTTCTGCATTTTCCTTACTTTTATTGTCAGAAACTCCAGAAAGTCAATCGTACTAATTTATCACGATTTGCTTTATTAATTTATACTTTGCTTATATGGAATTTTGCCCAGCAGACCTCATTACAATTTCTAACCTGTTTTATTTTGTTTTTTTTTCTGAGACAGGGTCTCCCTCTGTTGTCCAAGGCTGGAGTGTAGTAGTGCTATCGCAGCTGACTGCAGCCTCAACCTTCCAGGCTGAAGCGATCCTCCCACCTCAACCTCCCACGTGGCTGAGACTACAGGTGCTTGCCACTATGCCCAACTAACATTTGGAATTTTCGTATACGTGGATTCCAGAGGGGTGACAGCGAAAC |
| hsa_circ_0076091 | ATGTGGATGAGTGTGTGGAGGGGACTGACAACTGCCACATCGATGCTATCTGCCAGAACACCCCGAGGTCATACAAGTGCATCTGCAAGTCTGGCTACACAGGGGACGGCAAACACTGCAAAG |
| hsa_circ_0076424 | AGGTACTTGGAAGGCTCCATCGCCCCTGAAGACAAAGGAAGCTGCTCATTGCTGCCTGGGCCTCCATTTGCCAGAGAGTTACACATCGAAATTGTGTCTTCTCCCCACTACAGCACTAATGGAAATTATGACGGTGTTCTTTACCGGCACTTTCAGATACCCAGGGTAGTCCAGGAAGGGGATGTTCTATGTGTGCCAACAATTGGGCAAGTAGAGATCCTGGAAGGAAGTCCAGAGAAACTGCCCAGGTGGCGGGAAATGTTTTTTAAAGTGAAGAAAACAGTTGGGGAAGCTCCAGATGGACCAGCCAGTGCCTACTTGGCCGACACCACCCATACCTCCTTGTACATGGTGGGTTCTACCCTGAGCCCTGTTCCATGGCTCCCTTCAGAGGAATCCACTCTCTGGAGCAGTTTGTCTCCTCCAGGCCTGGAGGCCTTGGTGTCTGAACTCTGTGCTGTCCTGAAGCCTCGCCTCCAGCCAGGGGGTGCCCTGCTGACAGGAACTAGCAGTGTCCTTCTACGGGGCCCCCCAGGCTGTGGGAAGACCACAGTAGTTGCTGCTGCCTGTAGTCACCTTGGGCTCCACTTACTGAAGGTGCCCTGCTCCAGCCTCTGTGCAGAAAGTAGTGGGGCTGTGGAGACAAAACTGCAGGCCATCTTCTCCCGGGCCCGCCGTTGCCGGCCTGCAGTCCTGTTGCTCACAGCTGTGGACCTTCTGGGCCGGGACCGTGATGGGCTGGGTGAGGATGCCCGTGTGATGGCTGTGCTGCGTCACCTCCTCCTCAATGAGGACCCCCTCAACAGCTGCCCTCCCCTCATGGTTGTGGCCACCACAAGCCGGGCCCAGGACCTGCCTGCTGATGTGCAGACAGCATTTCCTCATGAGCTCGAGGTGCCTGCTCTGTCAGAGGGGCAGCGGCTCAGCATCCTGCGGGCCCTCACTGCCCACCTTCCCCTGGGCCAGGAGGTGAACTTGGCACAGCTAGCACGGCGGTGTGCAGGCTTTGTGGTAGGGGATCTCTATGCCCTTCTGACCCACAGCAGCCGGGCAGCCTGCACCAGGATCAAGAACTCAGGTTTGGCAGGTGGCTTGACTGAGGAGGATGAGGGGGAGCTGTGTGCTGCCGGCTTTCCTCTCCTGGCTGAGGACTTTGGGCAGGCACTGGAGCAACTGCAGACAGCTCACTCCCAGGCCGTTGGAGCCCCCAAGATCCCCTCAGTGTCCTGGCATGATGTGGGTGGGCTGCAGGAGGTGAAGAAGGAGATCCTGGAGACCATTCAGCTCCCCCTGGAGCACCCTGAGCTACTGAGCCTGGGCCTGAGACGCTCAGGCCTTCTGCTCCATGGGCCCCCTGGCACCGGCAAGACCCTTCTGGCCAAGGCAGTAGCCACTGAGTGCAGCCTTACCTTCCTCAG |
| hsa_circ_0076432 | AGGTACTTGGAAGGCTCCATCGCCCCTGAAGACAAAGGAAGCTGCTCATTGCTGCCTGGGCCTCCATTTGCCAGAGAGTTACACATCGAAATTGTGTCTTCTCCCCACTACAGCACTAATGGAAATTATGACGGTGTTCTTTACCGGCACTTTCAGATACCCAG |
| hsa_circ_0076916 | GGCTGGTGAATTACCAGATCTCCATCAAGTGCAGTAACCAGTTCAAGTTGGAAGTGTGTCTTTTGAATGCAGAAAACAAAGTCGTGGACAACCAGGCTGGGACCCAGGGCCAGCTGAAGGTGCTGGGTGCCAACCTCTGGTGGCCGTACCTGATGCACGAACACCCCGCCTACCTGTACTCGTGGGAGGATGGTGATTGCTCACACCAAAGCCTTGGACCCCTCCCAGCCTGTGACCTTTGTGACCAACGTCACCTATGCAGCAGACAAGGGG |
| hsa_circ_0076917 | GGCTGGTGAATTACCAGATCTCCATCAAGTGCAGTAACCAGTTCAAGTTGGAAGTGTGTCTTTTGAATGCAGAAAACAAAGTCGTGGACAACCAGGCTGGGACCCAGGGCCAGCTGAAGGTGCTGGGTGCCAACCTCTGGTGGCCGTACCTGATGCACGAACACCCCGCCTACCTGTACTCGTGGGAG |
| hsa_circ_0077495 | CTATTTGAACTGCTGGGACCTGAAGGACTTGAACTTATTGAGAAACTCCTCCAGAACAGAATTACAATTGTGGATAGATTTCTTAATTCTTCAAATGATCATAGGTTTCAGGCTCTTCAAGACAATTGTAAAAAAATTTTAGGAGAAAATGCTAAACCCAATTATGGTTGTCAAGTCACTATTCAGTCTGAACAAGAAAAGCAGTTAATGAAACAATATCGACGTGAAGAAAAAAGAATTGCCAGACGAGAAAAAAAGGCTGGAGAAGATTTAGAAGTTTCAGAAGGACTTATGTGCTTTGATCCTAAGGAATTGCGGATACAAAGAGAACAGGCACTTCTGAATGCTAGAAGTGTTCCAATTCTGAGCAGGCAGAGAGATGCAGACGTTGAAAAAATACATTATCCCCATGTGTATGATTCCCAGGCTGAAGCCATGAAAACATCAGCATTTATTGCTGGTGCAAAGATGATTTTGCCAGAAGGAATCCAAAGAGAGAATAACAAGCTTTATGAAGAAGTAAGGATTCCCTACAGCGAACCAATGCCACTCAGCTTTGAGGAAAAGCCAGTTTATATCCAAGACTTAGATGAG |
| hsa_circ_0077522 | TTGTGGATCGGTGGAATGCTTGGTTTTGCTGTTAAAGAAAGGAGCAAATCCTAACTATCAAGATATTTCAGGCTGTACACCCCTTCATTTGGCAGCAAGAAATGGGCAGAAGAAATGTATGAGTAAATTATTAGAATATAGCGCTGATGTCAACATTTGTAATAATGAAGGCCTTACAGCA |
| hsa_circ_0078776 | AGATCTCGGCTCACCAAATTCCACAACCTGAAGGCAGTCGTCTGCAAGGCCTGCATGAAGGAGAACAGACGCATCACTGGCCGAGCCCACTGGGGCTCACACCACGCAG |
| hsa_circ_0079788 | TGGCAGCCGATTAGGAGGCCTGGTTACAGTGTTGTGCTTCTTTTTCAGTCATGGAGAGTGTACCTGTGTAATGTGGACACCACCTCTCCGCGAAAGCTTCTCATATCCATTTCTTGTTCTTCAGATGTTGCTTGTGACTCATATTCTCAG |
| hsa_circ_0079909 | GAAGAAGAGAGCGAAGAGGAACCCAAGCTGAAGTATGAAAGGCTTTCCAATGGGGTAACTGAAATACTTCAGAAGGATGCAGCTAGCTGCATGACAGTCCATGACAAGTTTTTGGCATTGGGCACACATTATGGCAAGGTTTATTTACTTGATGTCCAGGGGAACATCACTCAGAAGTTTGATGTA |
| hsa_circ_0080320 | GGCTGGTGAATTACCAGATCTCTGTCAAGGGCAGTAACCTGTTCAAGTTGGAAGTGCGTCTTTTGGATGCAGAAAACAAAGTCGTGGCGAATGGGACTGGGACCCAGGGCCAACTTAAGGTGCCAGGTGTCAGCCTCTGGTGGCCGTACCTGATGCACGAACGCCCTGCCTATCTGTATTCATTGGAGGTGCAGCTGACTGCACAGACGTCACTGGGGCCTGTGTCTGACTTCTACACACTCCCTGTGGGGATCCGCACTGTGGCTGTCACCAAGAGCCAGTTCCTCATCAATGGGAAACCTTTCTATTTCCACGGTGTCAACAAGCATGAGGATGCGGACATCCGAGGGAAGGGCTTCGACTGGCCGCTGCTGGTGAAGGACTTCAACCTGCTTCGCTGGCTTGGTGCCAACGCTTTCCGTACCAGCCACTACCCCTATGCAGAGGAAGTGATGCAGATGTGTGACCGCTATGGGATTGTGGTCATCGATGAGTGTCCCGGCGTGGGCCTGGCGCTGCCGCAGTTCTTCAACAACGTTTCTCTGCATCACCACATGCAGGTGATGGAAGAAGTGGTGCGTAGGGACAAGAACCACCCCGCGGTCGTGATGTGGTCTGTGGCCAACGAGCCTGCGTCCCACCTAGAATCTGCTGGCTACTACTTGAAGATGGTGATCGCTCACACCAAATCCTTGGACCCCTCCCGGCCTGTGACCTTTGTGAGCAACTCTAACTATGCAGCAGACAAGGGG |
| hsa_circ_0082360 | AGCTTGCCCTTGCATCTCTCTTCAGCCTTAATGCCTTATTTGATTTTTGGAGATATTTCAAATATACTGTGGCACCAACAAGTCTGGTTGTTAGTCCTGGACAGCAAACACTTTTAGGGTTGAAAACAGCTGTTGTACAGACTACGCCTCCACATGATCTGGCAGCAACCCAAATCCCTCCCGCTCCACCTTCCCCTTCAATTCAGGGTCAGAGTGTGTTGAGTTATAGCCCTTCTCGTTCGCCCAGTACCAGTCCCAAGTTCACCACCAGCTGTATGACTGGTTACAGCCCTCAGCTGCAAGGTCTGTCCTCAGGTGGCAGTGGTTCTTATAGCCCTGGAGTGACCTACTCGCCCGTCAGTGGTTATAATAAGTTGGCGAGCTTTAGCCCCTCTCCTCCTTCTCCGTACCCTACCACTGTTGGACCAGTGGAGAGCAGTGGATTGAGATCTCGCTACCGTTCTTCACCTACCGTCTACAACTCACCTACTGACAAAGAAGACTACATGACCGACCTACGAACTTTGGATACTTTTCTCAGAAGTGAAGAGGAGAAACAGCATAGGGTTAAGCTGGGGAGCCCAGATTCTACCTCTCCTTCCAGCAGTCCTACTTTCTGGAACTATAGTCGTTCTATGGGGGATTATGCACAAACTTTAAAGAAGTTTCAGTATCAGCTTGCCTGTAGGTCTCAGGCCCCATGTGCTAACAAAGATGAAGCCGATCTCAGCTCTAAACAAGCCGCAGAAGAG |
| hsa_circ_0084013 | GAGTGGCTGTAAGGTGAAGAAGCATGAAACTCAGTCTCTCGCCCTGGATGCATGTTCTCGGGATGAAGGGGCAGTGATCTCCCAGATTTCAGACATTTCTAATAGGGATGGCCATGCTACTGATGAGGAGAAACTGGCATCCACGTCATGTGGTCAGAAATCAGCTGGTGCCGAGGTGAAAGGTGAGCCAGAGGAAGACCTGGAGTACTTTGAATGTTCCAATGTTCCTGTGTCTACCATAAATCATGCGTTTTCATCCTCAGAAGCAGGCATAGAGAAGGAGACGTGCCAGAAGATGGAAGAAGACGGGTCCACTGTGCTT |
| hsa_circ_0084309 | AATGGAGATGAGGCGCCTGGTGTTTGGATGATCCCAACTTCAGATCCAGCGGCTAACTTGCATCCAGCTAAACCTAAAGATTTTTCGGCTTTCATTAACCTGGTGGAATTTTGCAGAGAGATTCTCCCTGAGAAACAAGCAGAATTTTTTGAACCATGGGTGTACTCATTTTCATATGAATTAATTTTGCAATCTACAAGGTTGCCCCTCATCAGTGGTTTCTACAAATTGCTTTCTATTACAGTAAGAAATGCCAAGAAAATAAAATATTTCGAGGGAGTTAGTCCAAAGAGTCTGAAACACTCTCCTGAAGACCCAGAAAAGTATTCTTGCTTTGCTTTATTTGTGAAATTTGGCAAAGAGGTGGCAGTTAAAATGAAGCAGTACAAAGATGAACTTTTGGCCTCTTGTTTGACCTTTCTTCTGTCCTTGCCACACAACATCATTGAACTCGATGTTAGAGCCTACGTTCCTGCACTGCAGATGGCTTTCAAACTGGGCCTGAGCTATACCCCCTTGGCAGAAGTAGGCCTGAATGCTCTAGAAGAATGGTCAATTTATATTGACAGACATGTAATGCAGCCTTATTACAAAGACATTCTCCCCTGCCTGGATGGATACCTGAAGACTTCAGCCTTGTCAGATGAGACCAAGAATAACTGGGAAGTGTCAGCTCTTTCTCGGGCTGCCCAGAAAGGATTTAATAAAGTGGTGTTAAAGCATCTGAAGAAGACAAAGAACCTTTCATCAAACGAAGCAATATCCTTAGAAGAAATAAGAATTAGAGTAGTACAAATGCTTGGATCTCTAGGAGGACAAATAAACAAAAATCTTCTGACAGTCACGTCCTCAGATGAGATGATGAAGAGCTATGTGGCCTGGGACAGAGAGAAGCGGCTGAGCTTTGCAGTGCCCTTTAGAGAGATGAAACCTGTCATTTTCCTGGATGTGTTCCTGCCTCGAGTCACAGAATTAGCGCTCACAGCCAGTGACAGACAAACTAAAGTTGCAGCCTGTGAACTTTTACATAGCATGGTTATGTTTATGTTGGGCAAAGCCACGCAGATGCCAGAAGGGGGACAGGGAGCCCCACCCATGTACCAGCTCTATAAGCGGACGTTTCCTGTGCTGCTTCGACTTGCGTGTGATGTTGATCAGGTGACAAGGCAACTGTATGAGCCACTAGTTATGCAGCTGATTCACTGGTTCACTAACAACAAGAAATTTGAAAGTCAGGATACTGTTGCCTTACTAGAAGCTATATTGGATGGAATTGTGGACCCTGTTGACAGTACTTTAAGAGATTTTTGTGGTCGGTGTATTCGAGAATTCCTTAAATGGTCCATTAAGCAAATAACACCACAGCAGCAGGAGAAGAGTCCAGTAAACACCAAATCGCTTTTCAAGCGACTTTATAGCCTTGCGCTTCACCCCAATGCTTTCAAGAGGCTGGGAGCATCACTTGCCTTTAATAATATCTACAGGGAATTCAGGGAAGAAGAGTCTCTGGTGGAACAGTTTGTGTTTGAAGCCTTGGTGATATACATGGAGAGTCTGGCCTTAGCACATGCAGATGAGAAGTCCTTAGGTACAATTCAACAGTGTTGTGATGCCATTGATCACCTATGCCGCATCATTGAAAAGAAGCATGTTTCTTTAAATAAAGCAAAGAAACGACGTTTGCCGCGAGGATTTCCACCTTCCGCATCATTGTGTTTATTGGATCTGGTCAAGTGGCTTTTAGCTCATTGTGGGAGGCCCCAGACAGAATGTCGACACAAATCCATTGAACTCTTTTATAAATTCGTTCCTTTATTGCCAGGCAACAGATCCCCTAATTTGTGGCTGAAAGATGTTCTCAAGGAAGAAGGTGTCTCTTTTCTCATCAACACCTTTGAGGGGGGTGGCTGTGGCCAGCCCTCGGGCATCCTGGCCCAGCCCACCCTCTTGTACTTCGGGGGCCATTCAGCCTGCAGGCCACGCTATGCTGGCTGGACCTGCTCCTGGCCGCGTTGGAGTGCTACAACACGTTCATTGGCGAGAGAACTGTAGGAGCGCTCCAGGTCCTAGGTACTGAAGCCCAGTCTTCACTTTTGAAAGCAGTGGCTTTCTTCTTAGAAAGCATTGCCATGCATGACATTATAGCAGCAGAAAAGTGCTTTGGCACTGGGGCAGCAGGTAACAGAACAAGCCCACAAGAGGGAGAAAGGTACAACTACAGCAAATGCACCGTTGTGGTCCGGATTATGGAGTTTACCACGACTCTGCTAAACACCTCCCCGGAAGGATGGAAGCTCCTGAAGAAGGACTTGTGTAATACACACCTGATGAGAGTCCTGGTGCAGACGCTGTGTGAGCCCGCAAGCATAGGTTTCAACATCGGAGACGTCCAGGTTATGGCTCATCTTCCTGATGTTTGTGTGAATCTGATGAAAGCTCTAAAGATGTCCCCATACAAAGATATCCTAGAGACCCATCTGAGAGAGAAAATAACAGCACAGAGCATTGAGGAGCTTTGTGCCGTCAACTTGTATGGCCCTGACGCGCAAGTGGACAGGAGCAGGCTGGCTGCTGTTGTGTCTGCCTGTAAACAGCTTCACAGAGCTGGGCTTCTGCATAATATATTACCGTCTCAGTCCACAGATTTGCATCATTCTGTTGGCACAGAACTTCTTTCCCTGGTTTATAAAGGCATTGCCCCTGGAGATGAGAGACAGTGTCTGCCTTCTCTAGACCTCAGTTGTAAGCAGCTGGCCAGCGGACTTCTGGAGTTAGCCTTTGCTTTTGGAGGACTGTGTGAGCGCCTTGTGAGTCTTCTCCTGAACCCAGCGGTGCTGTCCACGGCGTCCTTGGGCAGCTCACAGGGCAGCGTCATCCACTTCTCCCATGGGGAGTATTTCTATAGCTTGTTCTCAGAAACGATCAACACGGAATTATTGAAAAATCTGGATCTTGCTGTATTGGAGCTCATGCAGTCTTCAGTGGATAATACCAAAATGGTGAGTGCCGTTTTGAACGGCATGTTAGACCAGAGCTTCAGGGAGCGAGCAAACCAGAAACACCAAGGACTGAAACTTGCGACTACAATTCTGCAACACTGGAAGAAGTGTGATTCATGGTGGGCCAAAGATTCCCCTCTCGAAACTAAAATGGCAGTGCTGGCCTTACTGGCAAAAATTTTACAGATTGATTCATCTGTATCTTTTAATACAAGTCATGGTTCATTCCCTGAAGTCTTTACAACATATATTAGTCTACTTGCTGACACAAAGCTGGATCTACATTTAAAGGGCCAAGCTGTCACTCTTCTTCCATTCTTCACCAGCCTCACTGGAGGCAGTCTGGAGGAACTTAGACGTGTTCTGGAGCAGCTCATCGTTGCTCACTTCCCCATGCAGTCCAGGGAATTTCCTCCAGGAACTCCGCGGTTCAATAATTATGTGGACTGCATGAAAAAGTTTCTAGATGCATTGGAATTATCTCAAAGCCCTATGTTGTTGGAATTGATGACAGAAGTTCTTTGTCGGGAACAGCAGCATGTCATGGAAGAATTATTTCAATCCAGTTTCAGGAGGATTGCCAGAAGGGGTTCATGTGTCACACAAGTAGGCCTTCTGGAAAGCGTGTATGAAATGTTCAGGAAGGATGACCCCCGCCTAAGTTTCACACGCCAGTCCTTTGTGGACCGCTCCCTCCTCACTCTGCTGTGGCACTGTAGCCTGGATGCTTTGAGAGAATTCTTCAGCACAATTGTGGTGGATGCCATTGATGTGTTGAAGTCCAGGTTTACAAAGCTAAATGAATCTACCTTTGATACTCAAATCACCAAGAAGATGGGCTACTATAAGATTCTAGACGTGATGTATTCTCGCCTTCCCAAAGATGATGTTCATGCTAAGGAATCAAAAATTAATCAAGTTTTCCATGGCTCGTGTATTACAGAAGGAAATGAACTTACAAAGACATTGATTAAATTGTGCTACGATGCATTTACAGAGAACATGGCAGGAGAGAATCAGCTGCTGGAGAGGAGAAGACTTTACCATTGTGCAGCATACAACTGCGCCATATCTGTCATCTGCTGTGTCTTCAATGAGTTAAAATTTTACCAAGGTTTTCTGTTTAGTGAAAAACCAGAAAAGAACTTGCTTATTTTTGAAAATCTGATCGACCTGAAGCGCCGCTATAATTTTCCTGTAGAAGTTGAGGTTCCTATGGAAAGAAAGAAAAAGTACATTGAAATTAGGAAAGAAGCCAGAGAAGCAGCAAATGGGGATTCAGATGGTCCTTCCTATATGTCTTCCCTGTCATATTTGGCAGACAGTACCCTGAGTGAGGAAATGAGTCAATTTGATTTCTCAACCGGAGTTCAGAGCTATTCATACAGCTCCCAAGACCCTAGACCTGCCACTGGTCGTTTTCGGAGACGGGAGCAGCGGGACCCCACGGTGCATGATGATGTGCTGGAGCTGGAGATGGACGAGCTCAATCGGCATGAGTGCATGGCGCCCCTGACGGCCCTGGTCAAGCACATGCACAGAAGCCTGGGCCCGCCTCAAGGAGAAGAGGATTCAGTGCCAAGAGATCTTCCTTCTTGGATGAAATTCCTCCATGGCAAACTGGGAAATCCAATAGTACCATTAAATATCCGTCTCTTCTTAGCCAAGCTTGTTATTAATACAGAAGAGGTCTTTCGCCCTTACGCGAAGCACTGGCTTAGCCCCTTGCTGCAGCTGGCTGCTTCTGAAAACAATGGAGGAGAAGGAATTCACTACATGGTGGTTGAGATAGTGGCCACTATTCTTTCATGGACAGGCTTGGCCACTCCAACAGGGGTCCCTAAAGATGAAGTGTTAGCAAATCGATTGCTTAATTTCCTAATGAAACATGTCTTTCATCCAAAAAGAGCTGTGTTTAGACACAACCTTGAAATTATAAAGACCCTTGTCGAGTGCTGGAAGGATTGTTTATCCATCCCTTATAGGTTAATATTTGAAAAGTTTTCCGGTAAAGATCCTAATTCTAAAGACAACTCAGTAGGGATTCAATTGCTAGGCATCGTGATGGCCAATGACCTGCCTCCCTATGACCCACAGTGTGGCATCCAGAGTAGCGAATACTTCCAGGCTTTGGTGAATAATATGTCCTTTGTAAGATATAAAGAAGTGTATGCCGCTGCAGCAGAAGTTCTAGGACTTATACTTCGATATGTTATGGAGAGAAAAAACATACTGGAGGAGTCTCTGTGTGAACTGGTTGCGAAACAATTGAAGCAACATCAGAATACTATGGAGGACAAGTTTATTGTGTGCTTGAACAAAGTGACCAAGAGCTTCCCTCCTCTTGCAGACAGGTTCATGAATGCTGTGTTCTTTCTGCTGCCAAAATTTCATGGAGTGTTGAAAACACTCTGTCTGGAGGTGGTACTTTGTCGTGTGGAGGGAATGACAGAGCTGTACTTCCAGTTAAAGAGCAAGGACTTCGTTCAAGTCATGAGACATAGAGATGATGAAAGACAAAAAGTATGTTTGGACATAATTTATAAGATGATGCCAAAGTTAAAACCAGTAGAACTCCGAGAACTTCTGAACCCCGTTGTGGAATTCGTTTCCCATCCTTCTACAACATGTAGGGAACAAATGTATAATATTCTCATGTGGATTCATGATAATTACAGAGATCCAGAAAGTGAGACAGATAATGACTCCCAGGAAATATTTAAGTTGGCAAAAGATGTGCTGATTCAAGGATTGATCGATGAGAACCCTGGACTTCAATTAATTATTCGAAATTTCTGGAGCCATGAAACTAGGTTACCTTCAAATACCTTGGACCGGTTGCTGGCACTAAATTCCTTATATTCTCCTAAGATAGAAGTGCACTTTTTAAGTTTAGCAACAAATTTTCTGCTCGAAATGACCAGCATGAGCCCAGATTATCCAAACCCCATGTTCGAGCATCCTCTGTCAGAATGCGAATTTCAGGAATATACCATTGATTCTGATTGGCGTTTCCGAAGTACTGTTCTCACTCCGATGTTTGTGGAGACCCAGGCCTCCCAGGGCACTCTCCAGACCCGTACCCAGGAAGGGTCCCTCTCAGCTCGCTGGCCAGTGGCAGGGCAGATAAGGGCCACCCAGCAGCAGCATGACTTCACACTGACACAGACTGCAGATGGAAGAAGCTCATTTGATTGGCTGACCGGGAGCAGCACTGACCCGCTGGTCGACCACACCAGTCCCTCATCTGACTCCTTGCTGTTTGCCCACAAGAGGAGTGAAAGGTTACAGAGAGCACCCTTGAAGTCAGTGGGGCCTGATTTTGGGAAAAAAAGGCTGGGCCTTCCAGGGGACGAGGTGGATAACAAAGTGAAAGGTGCGGCCGGCCGGACGGACCTACTACGACTGCGCAGACGGTTTATGAGGGACCAGGAGAAGCTCAGTTTGATGTATGCCAGAAAAGGCGTTGCTGAGCAAAAACGAGAGAAGGAAATCAAGAGTGAGTTAAAAATGAAGCAGGATGCCCAGGTCGTTCTGTACAGAAGCTACCGGCACGGAGACCTTCCTGACATTCAGATCAAGCACAGCAGCCTCATCACCCCGTTACAGGCCGTGGCCCAGAGGGACCCAATAATTGCAAAACAGCTCTTTAGCAGCTTGTTTTCTGGAATTTTGAAAGAGATGGATAAATTTAAGACACTGTCTGAAAAAAACAACATCACTCAAAAGTTGCTTCAAGACTTCAATCGTTTTCTTAATACCACCTTCTCTTTCTTTCCACCCTTTGTCTCTTGTATTCAGGACATTAGCTGTCAGCACGCAGCCCTGCTGAGCCTCGACCCAGCGGCTGTTAGCGCTGGTTGCCTGGCCAGCCTACAGCAGCCCGTGGGCATCCGCCTGCTAGAGGAGGCTCTGCTCCGCCTGCTGCCTGCTGAGCTGCCTGCCAAGCGAGTCCGTGGGAAGGCCCGCCTCCCTCCTGATGTCCTCAGATGGGTGGAGCTTGCTAAGCTGTATAGATCAATTGGAGAATACGACGTCCTCCGTGGGATTTTTACCAGTGAGATAGGAACAAAGCAAATCACTCAGAGTGCATTATTAGCAGAAGCCAGAAGTGATTATTCTGAAGCTGCTAAGCAGTATGATGAG |
| hsa_circ_0085616 | ATATCCAGGAATGATTTTCAGAGTATTTGAGAAAATGAGAGGTGCAGATGGTTTTCACTGAGGCATGGTTTCAAATTTAAAGAGGATTATGGCCCAGAAATGGATGCCGGACCAGATCTCTGTCTCGGAGTTCATCGCCGAGACCACCGAGGACTACAACTCGCCCACCACGTCCAGCTTCACCACGCGGCTGCACAACTGCAGGAACACCGTCACGCTGCTGGAGGAG |
| hsa_circ_0085767 | GCAGAACTGGGGCTCCCTTGCATCTTCCAGTTACAAATTCAGTGCCTTCTGCAGTTTCCCCAGAGCTCCTCAAGAATAACGGAAGGGAGAATATGACAGATACCTAGCATCTAGCAAAATAATGGCAGCTGCTTACCTTGACCCCAACTTGAATCACACACCAAATTCGAGTACTAAGACTCACCTGGGTACTGGTATGGAACGTTCTCCTGGTGCAATGGAGCGAGTATTAAAGGTCTTTCATTATTTTGAAAGCAATAGTGAGCCAACCACCTGGGCCAGTATTATCAGGCATGGAGATGCTACTGATGTCAGGGGCATCATTCAGAAGATAGTGGACAGTCACAAAGTAAAGCATGTGGCCTGCTATGGATTCCGCCTCAGTCACCTGCGGTCAGAGGAGGTTCACTGGCTTCACGTGGATATGGGCGTCTCCAGTGTGAGGGAGAAGTATGAGCTTGCTCACCCACCAGAGGAGTGGAAATATGAATTGAGAATTCGTTATTTGCCAAAAGGATTTCTAAACCAGTTTACTGAAGATAAGCCAACTTTGAATTTCTTCTATCAACAGGTGAAGAGCGATTATATGTTAGAGATAGCTGATCAAGTGGACCAGGAAATTGCTTTGAAGTTGGGTTGTCTAGAAATACGGCGATCATACTGGGAGATGCGGGGCAATGCACTAGAAAAGAAGTCTAACTATGAAGTATTAGAAAAAGATGTTGGTTTAAAGCGATTTTTTCCTAAGAGTTTACTGGATTCTGTCAAGGCCAAAACACTAAGAAAACTGATCCAACAAACATTTAGACAATTTGCCAACCTTAATAGAGAAGAAAGTATTCTGAAATTCTTTGAGATCCTGTCTCCAGTCTACAGATTTGATAAGGAATGCTTCAAGTGTGCTCTTGGT |
| hsa_circ_0086709 | CATTGATCTGGTAGCCTTGCTCCAGAAGCCTGTTCCTCACAGTCAAGCCTCAGAAGCCAACTCCTTTGAAACTTCCCAACAGCAGGGCTTTGGCCAAGCCCTTGTCTTCACAAATTCGCAACACAACAATCAGATGGCACCAGGGACTGGCAGCTCCACTGCCGTCAACTCCTGTTCTCCTCAGAGCCTGTCATCCGTCCTTGGCTCAGGATTTGGAGAGCTTGCACCACCAAAAATGGCAAACATCACCAGCTCCCAGATTTTGGACCAGTTGAAAGCTCCGAGTTTGGGCCAGTTTACCACCACCCCAAGTACACAGCAGAATAGTACAAGTCACCCTACAACTACTACTTCTTGGGACCTCAAGCCCCCAACATCCCAGTCCTCAGTCCTCAGTCATCTTGACTTCAAATCTCAACCTGAGCCATCCCCAGTTCTTAGCCAGTTGAGCCAGCGACAACAGCACCAGAGCCAGGCAGTCACTGTTCCTCCTCCTGGTTTGGAGTCCTTTCCTTCCCAGGCAAAACTTCGAGAATCAACACCTGGAGACAGTCCCTCCACTGTGAACAAGCTTTTGCAGCTTCCCAGCACGACCATTGAAAATATCTCTGTGTCTGTCCACCAGCCACAGCCCAAACACATCAAACTTGCTAAGCGGCGGATACCCCCAGCTTCTAAGATCCCAGCTTCTGCAGTGGAAATGCCTGGTTCAGCAGATGTCACAGGATTAAATGTGCAGTTTGGGGCTCTGGAATTTGGGTCAGAACCTTCTCTCTCTGAATTTGGATCAGCTCCAAGCAGTGAAAATAGTAATCAGATTCCCATCAGCTTGTATTCGAAGTCTTTAAGTGAGCCTTTGAATACATCTTTATCAATGACCAGTGCAGTACAGAACTCCACATATACAACTTCCGTCATTACCTCCTGCAGTCTGACAAGCTCATCACTGAATTCTGCTAGTCCAGTAGCAATGTCTTCCTCTTATGACCAGAGTTCTGTGCATAACAGGATCCCATACCAAAGCCCTGTGAGTTCATCAGAGTCAGCTCCAGGAACCATCATG |
| hsa_circ_0087938 | ATCTGGACGTTGGTGGTTGGCTATGTGTTGACTGTTTCATTCAAGTGGAATGCAAGCACTGAACGCTACTTGAGAGCAGTTTCAATTCCTGTCTGGATTATATTGCTTTTTCATTTAGCATCCCTGGCTGGCTCATGGAGAATTCCAGTATTCCTGGTTATTGTTTTCCTGATGTCTGTGGGTACCCTCTATGAAAAACAGAATGGAAAAGAGTCTTCTG |
| hsa_circ_0087960 | GTGGACGTCTGATTTATGAAGCTCCCCATCCACCTATCTGAGTACCTGACTTCTCAGGACTGACACCTACAGCATCAGGTACACAGCTTCTCCTAGCATGACTTCGATCTGATCAGCAAACAAGAAAATTTGTCTCCCGTAGTTCTGGGGCGTGTTCACCACCTACAACCACAGAGCTGTCATGGCTGCCATCTCTACTTCCATCCCTGTAATTTCACAGCCCCAG |
| hsa_circ_0087963 | GTGGACGTCTGATTTATGAAGCTCCCCATCCACCTATCTGAGTACCTGACTTCTCAGGACTGACACCTACAGCATCAG |
| hsa_circ_0088213 | AGCCTACTTGGATGTTAATGAGCTGAAGAACATTCTTAAATTGGATGGATCAACACATCTCAATATTTTCTTTGCAAAATCCTCAGAGGAGGAGTTGGCAGGAGTAGCAACTTGGCCATGGGACAAGGAGGCCCTGATGCACTTAGGTGGCATTGTCTTGAACCCATCTTTCTATGGCATGCCTGGGCACACCCACACCATGATCCATGAGATTGGTCACAGCCTGGGCCTCTATCACGTCTTCCGAGGCATCTCAGAAATCCAGTCCTGCAGTGACCCCTGCATGGAGACAGAGCCCTCCTTCGAGACTGGAGACCTCTGCAATGATACCAACCCAGCCCCTAAACACAAGTCCTGTGGTGACCCAGGGCCAGGAAATGACACCTGTGGCTTTCATAGCTTCTTCAACACTCCTTACAACAACTTCATGAGCTATGCAGATGACGACTGTACGGACTCCTTCACGCCCAATCAAGTCGCCAGAATGCACTGTTACCTGGACCTGGTCTACCAGGGCTGGCAGCCCTCCAGGAAACCAGCGCCTGTTGCCCTCGCCCCCCAAGTTCTGGGCCACACAACGGACTCTGTGACACTGGAGTGGTTCCCACCTATAGATGGCCATTTCTTTGAAAG |
| hsa_circ_0088240 | GTGTGTCGAACCAAGGTGATAGATCTCAGTGAAGGCATTTCCCAGCATGCCTGGTACCCTTGCACCATCAGCTACCCATATTCCCAGCTGGCTCAGACCACTTTTTGGCTCCGGGCGTATTTTTCTCAACCAATGGTTGCCGCAGCTGTCATTGTCCACCTGGTGACGGATGGGACATATTATGGGGACCAAAAGCAGGAGACCATCAGCGTGCAGCTGCTTGATACCAAAGATCAGAGCCACGATCTAGGCCTCCATGTCCTGAGCTGCAGGAACAATCCCCTGATTATCCCTGTGGTCCATGACCTCAGCCAGCCCTTCTACCACAGCCAGGCGGTACGTGTGAGCTTCAGTTCGCCCCTGGTCGCCATCTCGGGGGTGGCCCTCCGTTCCTTCGACAACTTTGACCCCGTCACCCTGAGCAGCTGCCAGAGAGGGGAGACCTACAGCCCTGCCGAGCAGAGCTGCGTGCACTTCGCATGTGAGAAAACTGACTGTCCAGAGCTGGCTGTGGAGAATGCTTCTCTCAATTGCTCCAGCAGCGACCGCTACCACGGTGCCCAGTGTACTGTGAGCTGCCGGACAGGCTACGTGCTCCAGATACGGCGGGATGATGAGCTGATCAAGAGCCAG |
| hsa_circ_0088249 | CTGCGTGCACTTCGCATGTGAGAAAACTGACTGTCCAGAGCTGGCTGTGGAGAATGCTTCTCTCAATTGCTCCAGCAGCGACCGCTACCACGGTGCCCAGTGTACTGTGAGCTGCCGGACAGGCTACGTGCTCCAGATACGGCGGGATGATGAGCTGATCAAGAGCCAGACGGGACCCAGCGTCACAGTGACCTGTACAGAGGGCAAGTGGAATAAGCAGGTGGCCTGTGAGCCAGTCGACTGCAGCATCCCAGATCACCATCAAGTCTATGCTGCCTCCTTCTCCTGCCCTGAGGGCACCACCTTTGGCAGTCAATGTTCCTTCCAGTGCCGTCACCCTGCACAATTGAAAGGCAACAACAGCCTCCTGACCTGCATGGAGGATGGGCTGTGGTCCTTCCCAGAGGCCCTGTGTGAGCTCATGTGCCTCGCTCCACCCCCTGTGCCCAATGCAGACCTCCAGACCGCCCGGTGCCGAGAGAATAAGCACAAGGTGGGCTCCTTCTGCAAATACAAATGCAAGCCTGGATACCATGTGCCTGGATCCTCTCGGAAGTCAAAGAAACGGGCCTTCAAGACTCAGTGTACCCAGGATGGCAGCTGGCAGGAGGGAGCTTGTGTTCCTGTGACCTGTGACCCACCTCCACCAAAATTCCATGGGCTCTACCAGTGTACTAATGGCTTCCAGTTCAACAGTGAGTGTAGGATCAAGTGTGAAGACAGTGATGCCTCCCAGGGACTTGGGAGCAATGTCATTCATTGCCGGAAAGATGGCACCTGGAACGGCTCCTTCCATGTCTGCCAGGAGATGCAAGGCCAGTGCTCGGTTCCAAACGAGCTCAACAGCAACCTCAAACTGCAGTGCCCTGATGGCTATGCCATAGGGTCGGAGTGTGCCACCTCGTGCCTGGACCACAACAGCGAGTCCATCATCCTGCCAATGAACGTGACCGTGCGTGACATCCCCCACTGGCTGAACCCCACACGGGTAGAG |
| hsa_circ_0088469 | GCAGAATCCAGAGACCACATTTGAAGTATATGTTGAAGTGGCCTATCCCAGGACAGGTGGCACTCTTTCAGATCCTGAGGTGCAGAGGCAATTCCCGGAGGACTACAGTGACCAGGAAGTTCTACAGACTTTGACCAAGTTTTGTTTCCCCTTCTATGTGGACAGCCTCACAGTTAGCCAAGTTGGCCAGAACTTCACATTCGTGCTCACTGACATTGACAGCAAACAGAGATTCGGGTTCTGCCGCTTATCTTCAGGAGCGAAGAGCTGCTTCTGTATCTTAAGCTATCTCCCCTGGTTCGAGGTATTTTATAAGCTGCTTAACATCCTGGCAGATTACACGACAAAAAGACAGGAAAATCAGTGGAATGAGCTTCTTGAAACTCTGCACAAACTTCCCATCCCTGACCCAGGAGTGTCTGTCCATCTCAGCGTGCATTCTTATTTTACTGTGCCTGATACCAGAGAACTTCCCAGCATACCTGAGAATAGAAATCTGACAGAATATTTTGTGGCTGTGGATGTTAACAACATGTTGCATCTGTACGCCAGTATGCTGTACGAACGCCGGATACTCATCATTTGCAGCAAACTCAGCACT |
| hsa_circ_0089842 | GCTGCCTTGGAGTTTTCATAAGAAATTGTCCCTGGAGGTGTTGGATGATCACAGCTTCCTTGGAGCATTGCAGTTGCTGGAATCCAGTTTCAGGATTAAGGGAGGGCTGCCTCCTTGCAATGGGCTGCCAAGAAAACGGCTGTGCTTGTTCTTAACCTCAGGCTCTGTCTGTGATCAGTCTGAGAGTCTCTCCCAGGTCTACTGCTCCCTGGAAAGCCCTATCTCTCTGCAGGCTCGCCTCTGGGCTTTGTCTCCTTGGAGCCACATCACTGGGACAGCTGTGGATGTGGATGCAGATTTGAACCATGTCACGGCCCCAGGGACTGCTATGGCTTCCTTTGTTGTTCACCCCGGTCTGCGTCATGTTAAACTCCAATGTCCTCCTGTGGTTAACTGCTCTTGCCATCAAGTTCACCCTCATTGACAGCCAAGCACAGTATCCAGTTGTCAACACAAATTATGGCAAAATCCGGGGCCTAAGAACACCGTTACCCAATGAGATCTTGGGTCCAGTGGAGCAGTACTTAGGGGTCCCCTATGCCTCACCCCCCACTGGAGAGAGGCGGTTTCAGCCCCCAGAACCCCCGTCCTCCTGGACTGGCATCCGAAATACTACTCAGTTTGCTGCTGTGTGCCCCCAGCACCTGGATGAGAGATCCTTACTGCATGACATGCTGCCCATCTGGTTTACCGCCAATTTGGATACTTTGATGACCTATGTTCAAGATCAAAATGAAGACTGCCTTTACTTAAACATCTACGTGCCCACGGAAGATG |
| hsa_circ_0089972 | CTACCAAGTCAGGATTGTTACCCCCACCACCTGCGCTCCCTCCAAGACCTTGTCCATCACAGTCTGAACAAGTGTCGGAGGCCGAGTTACTCCCACAGCTGAGCAGAGCCCCATCCCAGGCTGCAGAAAGTAGTCCAGCAAAGAAGGATGTACTGTATTCTCAGCCACCATCAAAGCCCATTCGTAGGAAATTCAGACCAGAAAACCAAGCTACAGAAAACCAAGAGCCTTCCACTGCTGCAAGTGGGCCAGCTTCTGCGGCAACCATGAAACCGCATCCAACAGTCCAAAAGCAGTCTTCCAAACAGAAGAAGGCCATTCAAACTGCTATCCGCAAAAATAAAGAGGCAAACGCAGTGCTGGCTCGGCTGAACAGTGAGCTCCAGCAGCAGCTCAAG |
| hsa_circ_0090801 | ATCGTAAAGCTGAAAATTGAAATCATGAAAGTAGACAGGACTAAACTGAAGAAGACACCTACTGAGGCTCCTGCAGACTGCAGAGCCTTAATAGACAAACTCAAAGTTTGTAATGATGAGCAACTTCTCTTGGAACTGCAGCAGATCAAAACATGGAACATTGGAAAGTGCGAGTTATATCACTGGGTGGACCTGTTGGACCGCTTCGATGGAATACTGGCAGATGCTGGACAGACAGTGGAGAATATGTCATGGATGCTCGTATGTGATAGGCCAGAAAGAGAGCAACTGAAAATGCTTCTCTTGGCTGTGTTGAACTTCACAGCCTTGCTCATTGAGTACAGCTTTTCCCGGCATCTGTACAGTTCCATAGAG |
| hsa_circ_0090856 | GTGCTGTTGATGGGTAAAAGTGGGTCTGGTAAGACCAGCATGAGGTCTATTATCTTTGCAAATTATATTGCCAGAGACACACGTCGCCTTGGCGCAACAATACTAGACCGTATACATAGTCTTCAAATTAATAGCAGTTTGAGCACCTACTCTCTCGTAGACTCTGTTGGAAATACAAAGACATTTGATGTAGAACATTCTCATGTTCGATTTCTGGGAAACCTGGTATTGAACCTGTGGGATTGTGGTGGGCAAGACACCTTCATGGAAAATTATTTCACTAGCCAACGGGACAACATCTTCCGAAATGTGGAGGTTCTGATTTATGTCTTTGATGTGGAGAGCCGCGAACTGGAAAAGGACATGCACTATTACCAATCATGCCTGGAGGCCATTCTGCAGAATTCTCCAGATGCCAAAATATTTTGCTTGGTACACAAAATGGATCTGGTACAGGAGGATCAACGGGACCTG |
| hsa_circ_0091038 | GTTGCTGAAGCAGAAGATTGTTATAATACAGCTCTCCGTCTGTGTCCCACCCATGCAGACTCTCTGAATAACCTAGCCAATATCAAACGAGAACAGGGAAACATTGAAGAGGCAGTTCGCTTGTATCGTAAAGCATTAGAAGTCTTCCCAGAGTTTGCTGCTGCCCATTCAAATTTAGCAAGTGTACTGCAGCAGCAGGGAAAACTGCAGGAAGCTCTGATGCATTATAAGGAGGCTATTCGAATCAGTCCTACCTTTGCTGATGCCTACTCTAATATGGGAAACACTCTAAAGGAGATGCAGGATGTTCAGGGAGCCTTGCAGTGTTATACGCGTGCCATCCAAATTAATCCTGCATTTGCAGATGCACATAGCAATCTGGCTTCCATTCATAAGGATTCAGGGAATATTCCAGAAGCCATAGCTTCTTACCGCACGGCTCTGAAACTTAAGCCTGATTTTCCTGATGCTTATTGTAACTTGGCTCATTGCCTGCAGATTGTCTGTGATTGGACAGACTATGATGAGCGAATGAAGAAGTTGGTCAGTATTGTGGCTGACCAGTTAGAGAAGAATAGGTTGCCTTCTGTGCATCCTCATCATAGTATGCTATATCCTCTTTCTCATGGCTTCAGGAAGGCTATTGCTGAGAGGCACGGCAACCTGTGCTTAGATAAG |
| hsa_circ_0094094 | GTGAAAGAGATGCCCATGCAGACTCTGGTCCCCGCCAAAGTGGCCTCCAAGAATGTCATCCCTGCCCTGGAACTGGTGGAGCCCATTAAGAAGCACGAGGTCCCAGCAAAGTCTGATGTTTACTGTGAGGTGTGTGAATTCCTG |
| hsa_circ_0094298 | CAATGAATGAGAACTTCTGTTGCTGCTCCATATCCTCATCAGCATTTGGTACTATGCTTTGGACATTAACTATTAGGTATGTAGTGGTATTTCAATGTTGTTTTAATTTGCAGTTCCCTAATGATAAATGATGTTGAACATTTTCTCACATGCTTATTTGCCATCTGTATATCTTCTTTGGTGATGTGTCTGTTCAGATCTTTGCTTATTTTTATTTTATTTTTTATAATTTCAACTTTTACTTTAGATTCAGTGGGTACATACGCAGGTTTGTATCACATATACATGGATATATTATGTGATACTGAGGTTTGGGATACAGATGATCCCATCACCCAGGTAGTGAGCATAGTACCCAACAATTTTTCAGCCCTTGCTCCTCTTTCCCTCCCCTGTCTAGTAGTCCCCAGCGTCTGTGTTACCATTGTTATGTCTGTGAGTACAATGTTTAGCTCCCACTTATAAGTGAGAACATGTGGTATTTGGTTTTCTCTTCCAGCGTTAATTCGCTTAGAATAATGGCCTCTAGCTATTTGCCCATTTTAAAATTGGGTTGTTTATTCTCTTGTTGAGTTTTAAGAGTTCTTTGTATATTTTAGATACCAATCCTTTATCAGATATATGTTTTGCAAATAATTTTTTCCAGTCGGTGGCTTATTCTCTTGATAGTACTTTTTGCAGGACAGATTTTCAAAAATTTTTAACATTTTCAACATCCATTTTTTCTTTCTTGGGTTGCACTTTTCATGTTATATCCAAAAAACTCATTGCCAAATCCAAGGTCACCTGGATTTTCTCCCTTTTCATATTATAGGAATTTTATAGTTTCGCATTTTGCATTTAGGCCTTTGATCCATTTTGACTTAATTTTTGTGAAAGATTTAAAGTCATTCTTTTTGTTTGTAGATGTCCATTTCTAGTCTCATTTGTTGAAAAGACTATCTTTTATACTCTGTTTTGAATTGCCTTTGCTCCTTTGTCGAAGATCAATTGACTGTGTATATGTGGGTCTATTTCTGAGCTCTCTATTGTATTCAGTTGATATGTCGGTTCTTTTGCCAATACCACACATGCCATTTTGATGACTGTAGCTTTGTATAGTAAGTCTTGAAGTTGAGTAGTGTTAGGCCTTCGACTTTCTTCTTCTTCAATATTGTTTCGGATATTTTGGGTCTTTTGCCTGTTCTAATAAAGAATTTAGAATCACCTTGCCGATATCCTCAAAATAACTTGCTGAAATTTTGATTGGGATTGTCTTAAAATGTTACCACAAACATAAAAATACGTACATTTAACTTTTAGTATAAAGTTTTTATTAATGGTGATGAAACACTAATGATTTTATGAAATCAAAACTGTGACTCAGGTTACATAAGAAATGAAGAAGAAAAATCTCCCCACCCCACACACATACACACACACACACACATACACAAGTATACACACACAGATATACAAACATCTGCACTTGCCAGTCTAACCTATAAATCACAAACTTTTAGATTAGCATGAAATCACACATCTTAGTAATCAAGAAGTTCTGTTGGGAGTGGGAAGAATAAAGTTATTTGTAAGTATATGGGTGTAGCCATAGAATTTGGAGAAATTCCTTTAAAACTGCATTAAATAATTATGCAACAATGATTTGTATGCTTGTGGGTTTAATATCAGGTTCTGCTACTGTATGTGAATTCAGTTTAAGTGGGTAAAGACGGTTATCTCTTCAAGATTCTTTTCTCTAGTTTGCATCTTTTTTTTTATCTTGTTGATTTTATTGGCAGCACTTAAAGAGCAAGGTTAGATAATATTGGAGACAGTGGGCTTTCATTCTCATGCCTTTGAAACCACTGAACTCATTTTTGCCTTCACCCATATCTCTAATGGTTCATTTCTTCATATTTACCTGCCTGCCTGGGCTATGATCGGCCACATAAGATCTCACTTCACTAACACATGGCTACTAGCATTCTAGTGTGTTCATGCCACAGCAGCTTTGCAGTCTTCATGTTGCTTTCACTACCGGAGTTTCTGTTAGAAATTCATGAATGTTAAAATTTATACAGAGGTACAAATGTATCAACTTAATGTGGGGGATTATGGCCAGTAATTCCCCCTTCATCTCTTATTTTTTATTACCCTTCTTACACATTTGCCCCTGGATTTGTGTCTTTAGTCTTAGAAAGTGATTTTACCTCCCATTCTGAGATTTATTTTTTAATGTGGAAACTGCATCATCTTCTCAGAATCTGAAATCTCAAATTCTGAGATTAAAATCTTCATTATCTTTCTGTCCCGTGTTCCCGCTCCCTCCTATCTTCATAGTCTCCTTTTATTTTCTGAAAATAATCCCTGTTATATATTTGCTTTCTGTTCCATCTTTCCCTCTTTCTGTCTCTTAGATTTCTCTGTTATATTGATTTTTTTCCCCAGAAAACTTCATGTGTTTCTCCCAGTGGGATTTTATGTCACTTTTAATTTATTGTCAGATGGTTTAGCAACAAATACATAGATGCAAAGCAAATGTGTCAATATTTTAACAGTTGGTAAATATAGGTGAAGGGTATATTGTTGTTCATTGTAGTATTCTTTTGATACTTCTATTTCAAGAAATGTTTCAAGATAAAGAGGTTGGAAACTTAATAATATTATAGTAAGTCATTTTTATTTAATATTTACTATATATTATGTATTAGTCCAAGCACTTTAACATATATTACCTCATTTAAGTCTTATACTAACCTAAGGAGACAAGTATTGTTATTTTGCCCATTTTATAGATTAGGAAATGAAGCATAGAGGGATTTGGTAACTTGCCCTGTGTGTTGTGTGGCTAGTAAGTGGCAGCTGAATTTGGACTCAGATCATCTATGCAGATAATATTAAAATTAATAAGATATGGCTCTGTTTTCAAAGGGCTTACAATCCAATTAGAGTTGAAAAGTATATAGGTTTCTTTATGCTGTGAAACAAATTAAGGATCATAAAATGATAGTTTTATCTAGTTGAGGGGATCAGGAAGGGCTTCTGAAGAAAGTAGCATTTCAGTACTAAGAGAAAATACAGGGTTAATTTTGAATCTCATGGTGGAGGTATTTGAAATGCAACCTAAGGCATATAAACTTTGTTCAATGAGCAGTAGAGAACATTGAGAGTTAAAGAAACTATGGCCCAAAGATAATAAATATTTTCAACAGCTAGTTACTGTCAGATAGGATTTAAACCTAAATCTGAGGTGAGAGGTGGCTATTGTGGCTTTTCTAGATGTCAGTTCTTTGTTTCAGTTAGTGAACAAGACTTGTTGACTCTTCCTTAAAACATTTTAAATTTATTAGTTCCATGCATTTGGATCACATTTATGTCAATATATGTCCATATTCCATACCTGATTCTCATGGCGTAGCCTGCAGACCAGTCTCCTTCAGTGTATTCCCTTTCTAATGCTCTCTCACTTGACAAAATAAGAAGTACCTTTTAATTTGATTATTAGCTATTCAGATTATAGTTCTAAAGCAGTGCTTTTGTCATGTGACAGCTACAGAATTTTAGAATCTTGGAGATATAAGCTTTTAAAAAAAATTCTAGTGGGTAAAATACATGTTCCTTACCCATTCAGTGCAAAAAAAAAAAAAAAAAACAAGAACAACAAAAAAATAAATAACCAAAAAAACCAAAAACTCAGATGCAAAGGAAAAGAGTACACAGTTCATTCCTTCATAGAGTTTGTAATTTGGCCTGAATTGCAAACAAAGTATACTGTAATAAATACTATAATGGGACACAGTACAGGTCGCTATAAAAACACCTGTGGAAGTGCCAAGTCCTGCCTTAGGGGATTTAAGGAAGTGTTCTCTGAAGTAGTGTCAGCTGATTTAAGATGAAGAGGAATTATCTAGATCAAGTGTGCACAGTGGAGGGCAGAATGGAGAGACTGACAAAGACTACTCAGAGGTTGTGGGTTTGGAGATGAGATTGTAGGATGCTTTGGGAATCTCAGGTAATTAGGTATGAATTAAGAAGTCACTCTGGGCCCGGCGCCGTGGCCGACGCCTGTAATCCCACCACTTTGGGAGGGCGAGGCGGGCGAATCACGAGGTCAGGAGATCGAGACCACCCTGTGAATGGTGAAACCCCCGTCTCTACTAAAAATACAAAAAATTAGCCGAGTGTGGTGGCGTTACTTGTAGTCCCAGCTACTCGGGAGGCTGAGGCAGGAGAATGGCGTGAACCCGGGAGGCGGAGCTTGCAGTGAGCCGAGATCACGCCACTGCACTCCAGCCTGGGCGACAGAGCGAGACTCCGTCTCAAAAAAAAAAAAAAAAAAAAAAAAAAAATGCCGGGCGCGGTGTCTCAAGCCTGTCATCCCAGCACTTTGGGAGGCCGAGGCGGGCGGATCACGAGGTCAGGAAATCGAGACCATCCTGGCTAACACGGTAAAACCCCGTCTGTACTAAAAATACAAAAAATTAGCCCGGCGTGCTGGCGGGCACCTGTAGTCCCAGCTACTTGGGAGACTGAGGCAGAGGAATGGCATGAATCCCCGAGGCGGAGCTTGCAGTGAGCCGAGATCGCGCCACTGCACTCTAGCCTGGGGGACAGAGCGAGACTCCGTCTCAAAAAAAAAAAAAAAAAAGGAAGTCACTAGTCACTGCGGGAGGCAGGTGTGTTGAGGGAAGAAGATGAAGAATAAATAGGATTCAGGCTGTCTGTGGCATCATAAAATCAACAGTTTGTAAATTTCTAAAAATCTTTTTCTTGGGTTAAAAGAAAGTTGGGCCTGAAGTACTTAATGAGAAGTAGTTTCAGAGAAAAATAATCTTGTAAAAATCGACCTCATACTGAATATTTGAGAATTCAGAGATGTGGGAATATTTGTTAAAAGCTAGAAAATTTGGTAGAAACCATATTTCTACGTTTTCAATATGGTTGAAATTTAATGCGTTGGAATTTTTGCACATTTAATGCATTATGAATTTAATTTTAATCTTGATTTTAAAATAGCCATTATATTGGAGATAATATTTTTTCAATGTCTTATTTCTTGAAATTTTGCTAGAAGTTGAAGACCAGCCTGAGCAACCCGTCTCTACTTACTAACTAGCCAACCAAATACACACACACACACACACACAGAGAGAGAGAGAGAGAGAGAGAGACAGAGAGACAGAGAGAGACAGACAGACAGCGAGGGCGATTTCACATATATTAGAAGTTCTCGGCCGGGCATGGTGGCTCACACCTGTAATCCCAGAACTTTGGCAGGCCAAGGCGGGTGGATCACCTGAGGTCAGAAGTTCAAGACCAGCCTGGCCAACATGGTGAAACCCCCTCTCTACAAAAATACAAAAATTAGGCATGATGGTGGGTGCCTGTAATCCCAGCTACTCGGGAGGTTGAGGCAGGAGAATCGCTTGAACTCCGGAGGCAGAGGTTACAATGAGCCAAGATCGTACCATTGCGCTCCAGCCTGGATGACTGAGCGAGACTCTGTCTCAAAAAAAAAAAGTTCTCCTCACAGTTTAAGAACCAACTGGAATTAAGGTAGCAATTTTTAAATTGCTATAACTATGACTTCTGGTTAAAATATTTTGCTTTTTACTCCCATGAAACACATTACTTTTATTTTTTTCCTTATTCAGCATGTATGTTATTGTATGAAACTGAAGGGAAATGTAAAAAATTTTAGAGTAGTTTTTTTTTTTTTTTTTTTTGAGACGGAGTCTCACTCTGTTGCCCAGCTTGGAGTGTAGTGGTGCGATCTTGGCTCACTGCAACCTCCGCCTCCCAGGTTCAGGGGATTCTCCTGCATTAGCCTCCTAAGTAACTAGGATTACAGGCACGTGCCACCATGCTGGGGTAATTTTTTGTATTTTTAGTAGGGATGAGGTTTCACCGAGAGTAGATTTTTTTAATGCACTGTAAGATTAGGAAATATGCGGTATATATAAAACTTTTATAAAAGCTTAAGTAACCTTTATTGGAAAATATGCTTTATTAGTAATATAAAAACAAAAAATTCATCTTAACTGCCTGTTGAGACTTTCAAGAAATGGGAGTCCAAGGATGTATACAAACTGAGAGTTAATGTTGGTAGCATCGTTATGGGGCTAAAAAAGGTGTGCAATTTAAGAAGTTAAACAAAGTTAAAAACTAATGAACAACAGTCAGTGCAGAAAAGGAAGAATAAGTTTAACTTTTTCACATAAAGTCTTTCCAGAGTATTGTTTCTATTCCGGTGCCTAAAGAAGGTTTATTCTTCCAGTATGTAAAATACACAAAAGATGGGGGATACAGAAGATAATTGTTTTAATAAACTACAGCTGATTGCGCCTTTTGTGGAAACACATCAGTGACTGAGGTGAGTGGAAGTCAGAGCCATGGTGATAATGATAAGTGATTTGTAATAATCACTGCAATAATTTCATTATCTTTATTTCTTTCTGAAATTGTATTGTATATCTTTTCCCTCCACCTTCTTCCCTGCCCTTCCAGAGGCTCTCCCTATAGAGAATCTCCTTTGGGTCATTTTGAAAGCTATGGAGGGATGCCCTTTTTCCAGGCTCAGAAGATGTTTGTTGATGTACCAGAAAATACAGTGATACTGGATGAGATGACCCTTCGGCACATGGTTCAGGATTGCACTGCTGTAAAAACTCAGTTACTCAAACTGAAACGTCTCCTGCATCAGCATGATGGAAGTGGTTCATTGCATGATATTCAACTGTCATTGCCATCCAGTCCAGAACCAGAAGATGGTGATAAAGTATATAAGAATGAAGATTTATTAAATGAAATAAAACAACTTAAAGACGAAATAAAGAAAAAAGATGAAAAGATCCAACTATTAGAACTTCAGCTTGCAACTCAGCATATCTGCCACCAAAAATGTAAAGAGGAAAAATGCACTTATGCTGATAAATATACCCAAACACCCTGGAGACGAATTCCT |
| hsa_circ_0094584 | GTGTATCTGTTGCTGATCGAGAGGCCAGTCTGGAATTAATTAAGTTGGACATATCCCGTACATTTCCATCTCTCTACATCTTTCAGAAGGGTGGTCCATATCATGATGTCTTGCATAGTATTTTAGGGGCATACACATGCTACAGGCCTGATGTTGGTTATGTCCAAGGGATGTCCTTCATTGCAGCAGTACTCATTCTCAATTTGGAAGAGGCAGATGCCTTTATCGCATTTGCCAATCTCCTGAATAAGCCATGCCAGTTGGCCTTTTTTCGTGTGGATCACAGCATGATGTTGAAATATTTTGCAACATTTGAAGTATTCTTTGAAGAAAATCTTTCCAAATTATTTCTTCACTTCAAATCTTACAGTCTTACACCAGATATATACTTGATAGACTG |
| hsa_circ_0094934 | GTGAAACACCATTCAAAGTCGTAGTCAAATCTCTTTCACCTAAAGAGTTGGTCCGGATACATGTCCCTAAACCTTTGGACAGGAATGATGGAACATTTTTGATGAGATATAGGATGTATGAAACTGTCGATGAAGGCCTGAAGATAGAGGTCCTTTATGGTGATGAACATGTGGCTCAGTCTCCCTATATTTTGAAAGGACCAGTGTACCATGAGTACTGTGAGTGTCCGGAAGATCCTCAGGCCTGGCAGAAGACTCTTTCTTGTCCAACCAAGGAACCACAGATTGCAAAAGATTTTGCTTCCTTTCCCAGCATCAATCTCCAGCAAATGCTAAAAGAAGTCCCCAAAAGGTTTGGGGATGAGAGAGGTGCCATTGTTCATTACACGATTCTCAATAACCATGTTTACCGGAGATCTTTAGGGAAATACACAGACTTCAAGATGTTCTCTGATGAGATTTTGTTATCATTGACAAGAAAGGTCCTTCTCCCAGATTTAGAATTTTATGTTAATCTTGGAGATTGGCCCTTGGAGCATCGAAAAGTCAATGGAACCCCTAGCCCCATACCTATCATTTCATGGTGTGGCTCTCTGGATTCAAGAGATGTTGTCCTTCCAACGTATGACATCACCCACTCCATGCTTGAAGCCATGCGGGGTGTTACAAATGATCTCCTCTCTATTCAGGGAAATACAGGGCCTTCCTGGATCAATAAAACAGAGAGAGCTTTCTTCAGAGGTAGAGACAGCCGAGAGGAGAGGCTCCAGTTGGTACAGCTGTCCAAAGAAAATCCTCAGCTACTAGATGCAGGAATTACAGGATATTTCTTTTTCCAAGAGAAAGAAAAGGAGCTTGGAAAAGCCAAGTTGATGGGTTTCTTTGATTTCTTTAAGTACAAGTATCAAGTAAATGTGGATGGGACCGTGGCTGCTTACAGATATCCATATCTCATGCTGGGCGACAGTCTGGTTTTAAAGCAGGACTCGCCATATTATGAACATTTCTACATGGCACTAGAACCTTGGAAGCATTATGTTCCAATTAAAAGAAATCTGAGTGATTTATTAGAGAAAGTTAAATGGGCTAAG |
| hsa_circ_0095937 | TCGGAATTGAGATGGGCGATTGGCAGGAAGTCTGGGATGAGAACACGGGATGTTATTATTATTGGAATACACAAACAAATGAAGTGACTTGGGAGTTACCCCAATATCTTGCCACACAGGTACAGGGATTACAGCATTACCAGCCCAGTTCTGTGCCAGGTGCTGAAACTAGTTTTGTGGTAAATACAGACATATATTCTAAGGAGAAAACGATTTCTGTTTCCAGTAGTAAAAGTGGACCAGTCATAGCCAAGCGAGAAGTTAAAAAGGAAGTAAATGAAGGAATTCAGGCTCTCTCAAATAGTGAGGAGGAGAAGAAAGGGGTGGCAGCATCGCTGCTTGCTCCTTTATTGCCTGAGGGAATAAAAGAAGAAGAAGAGAGATGGAGAAGAAAAGTAATTTGTAAAGAGGAGCCAGTTTCAGAAGTAAAAGAAACAAGTACAACAGTAGAAGAAGCAACAACAATAGTAAAGCCACAGGAAATTATGTTGGACAATATAGAAGACCCTTCTCAGGAGGATCTTTGCAGTGTTGTCCAATCTGGAGAAAGTGAGGAGGAAGAGGAACAAGATACCCTTGAACTGGAGCTAGTTTTGGAAAGGAAAAAAGCAGAGTTGCGAGCCTTGGAGGAAGGAGATGGTAGTGTGTCAGGGTCTAGTCCACGTTCTGATATCAGCCAGCCAGCATCTCAAGATGGAATGCGTAGGCTTATGTCTAAAAGAGGAAAATGGAAGATGTTTGTTCGAGCTACCAGTCCAGAATCTACCAGTAGGAGTTCTAGTAAAACTGGACGAGATACTCCAGAAAATGGAGAAACTGCAATTGGTGCTGAAAATTCAGAAAAAATAGATGAGAATTCAGATAAAGAGATGGAAGTAGAAGAATCTCCAGAGAAAATAAAAGTACAGACAACACCAAAAGTAGAAGAAGAACAGGATTTGAAATTTCAGATTGGAGAACTGGCAAATACCCTGACAAGTAAATTCGAGTTTCTAGGCATTAATAGACAATCCATCTCCAACTTTCATGTGCTGCTCTTACAGACTGAG |
| hsa_circ_0096042 | GTTCCTGGAGAGCCACTGGTTTGTGTGGGTCACACAGATGAATCACATCGTCATGGAGATTGACCAGGAGGCCTACCGTGACTGGTTCAGTAGCCAGCTGACAGCCACCTGCAACGTGGAGCAGTCCTTCTTCAACGACTGGTTCAGTGGACACCTTAACTTCCAGATTGAGCACCACCTCTTCCCCACCATGCCCCGGCACAACTTACACAAGATCGCCCCGCTGGTGAAGTCTCTATGTGCCAAGCATGGCATTGAATACCAGGAGAAGCCGCTACTGAGGGCCCTGCTGGACATCATCAG |
| hsa_circ_0096050 | TTTAAGATGCCTGAGATGCACTTCAAGACCCCCAAGATCTCCATGCCTGATGTGAACTTAAACTTGAAAGGCCCCAAAGTCAAAGGGGATATGGATGTGTCTGTTCCCAAG |
| hsa_circ_0096360 | TGACTGCATTATTGAGGAGAAGACGGTGGTCCTGCAGAAAAAAGACAATGAGGGCTTTGGATTCGTGCTTCGAGGGGCCAAAGCTGACACACCCATTGAAGAATTCACACCAACACCGGCTTTCCCAGCCCTACAGTACCTGGAGTCCGTGGATGAAGGTGGGGTGGCGTGGCAAGCCGGACTAAGGACCGGGGACTTCTTGATTGAG |
| hsa_circ_0097905 | CGACCGTGGCAGCCATGTATTACAGCTACTACATGCTACCGGACGGCACTTACTGCCTGGCGCCGCCCCCTCCCGGAATCGACGTGACTACTTACTACAGCACCCTTCCTGCTGGCGTGACCGTGTCTAACTCCCCTGGAGTGACGACCACCGCCCCACCACCTCCTGGGACCACACCACTACCGCCCCCAACCACAGCAGAGACTAGCAGCGGGGCCACCTCCACAACCACCACCACAAGTGCACTTGCCCCCGTGGCCGCCATCATCCCCCCGCCCCCCGACGTCCAGCCCGTGATTGACAAGCTGGCCGAGTATGTCGCCAGGAACGGCCTGAAGTTCGAGACCAGTGTTCGTGCCAAGAATGATCAAAGATTTGAGTTCCTGCAGCCGTGGCACCAGTATAATGCTTATTATGAGTTTAAGAAGCAGTTCTTCCTCCAGAAAGAAGGGGGCGATAGCATGCAGGCTGTGTCTGCACCAGAAGAGGCTCCCACAGACTCTGCTCCCGAGAAGCCAAGTGATGCTGGGGAGGATGGCGCGCCTGAAGACGCAGCCGAGGTGGGAGCACGGGCAGGCTCAGGCGGGAAGAAGGAGGCATCGTCCAGTAAGACCGTCCCGGACGGGAAGCTGGTGAAAGCTTCCTTTGCTCCAATAAGCTTTGCAATCAAGGCCAAAGAAAATGATCTGCTTCCCCTGGAAAAAAATCGTGTTAAGCTAGATGATGACAGTGATGATGATGAAGAAAGCAAAGAAGGCCAAGAAAGTTCTAGTAGTGCTGCAAACACTAACCCAGCAGTTGCCCCACCCTGTGTAGTTGTTGAGGAGAAGAAGCCTCAACTTACCCAGGAGGAGCTAGAAGCAAAGCAAGCAAAGCAAAAGCTGGAAGATCGCCTCGCAGCTGCTGCCCGGGAAAAGCTGGCCCAGGCGTCTAAGGAGTCAAAAGAGAAACAGCTTCAAGCAGAACGTAAAAGGAAAGCGGCGTTATTTTTACAGACCCTCAAAAATCCTCTGCCGGAAGCAGAAGCTGGGAAAATTGAGGAGAGTCCTTTCAGTGTCGAG |
| hsa_circ_0098286 | GTGCTATACGTGGATGACCTGCTTAGTTTTAATTTTTGGAGAAATACATTTTATAAAACATTTTCATCAGATTGCTAAAATTGTGCCACTTGAAGAATGGATGATGATGATTTTGGTGGTTTTGAGGCTGCGGAGACTTTTGATGGTGGAAGTGGTGAAACCCAAACAACATCTCCTGCTATTCCTTGGGCTGCCTTTCCTGCAGTATCTGGAGTCCATCTTTCACCATCTTCTCCTGAGATTGTACTGGACCGTGACCACTCTTCTTCCATTGGCTGCCTCTCTTCTGATGCCATTATTTCATCACCAGAGAATACACATGCAGCAAATAGCATTGTGAGTCAAACTATTCCAAAAGCACAGATTCAGCAATCAACACACACTCATCTGGATATCTCACTTTTTCCATTGGGTTTAACTGATGAAAAAAGTAATGGAACAATTGCCCTTGTGGATGATTCTGAGGATCCTGGAGCCAATGTATCTAACATACAGCTTCAGCAAAAAATTTCAAGTCTGGAGATTAAACTCAAAGTATCTGAAGAAGAAAAACAGAGAATTAAACAGGATGTGGAATCATTGATGGAAAAGCATAATGTCTTAGAAAAAGGCTTTCTAAAAGAAAAAGAGCAAGAGGCCATTTCTTTTCAAGATAGATACAAAGAACTTCAG |
| hsa_circ_0099053 | ATGAGAGTCCCTACACTAAATCCGCCAGCCAGACAAAGCCGCCTGATGGAGCGTTGGCTGTGAGGAGACAGAGCATCCCAGAGGAATTCAAGGGCTCCACAGTCGTCGAGCTGATGAAGAAGGAAGGCACTACCCTGGGTCTGACGGTATCGGGAGGAATTGATAAGGATGGCAAGCCAAGAGTATCTAATCTGCGGCAAGGAGGAATTGCTGCTAGAAGTGACCAGCTGGATGTGGGTGACTACATCAAAGCAGTGAATGGAATCAACCTGGCCAAATTCCGCCATGACGAGATCATCAGCTTGCTGAAGAATGTGGGAGAAAGAGTGGTTCTTGAAGTAGAGTACGAGCTTCCACCGGTCTCTGTGCAAGGATCAAGTGTTATTTTCCGAACAGTGGAGGTCACATTACATAAAGAAGGCAATACCTTTGGTTTTGTAATTCGAG |
| hsa_circ_0099702 | TTTACGTTTAATAAGAAGGAGACCTGTAAGCTTGTTTGTACAAAAACATACCATACAGAGAAAGCTGAAGACAAACAAAAGTTAGAATTCTTGAAAAAAAGCATGTTATTGAATTATCAACATCACTGGATTGTGGATAATATGCCTGTAACGTGGTGTTACGATGTTGAAGATGGTCAGAGGTTCTGTAATCCTGGATTTCCTATTGGCTGTTACATTACAGATAAAGGCCATGCAAAAGATGCCTGTGTTATTAGTTCAGATTTCCATGAAAGAGATACATTTTACATCTTCAACCATGTTGACATCAAAATATACTATCATGTTGTTGAAACTGGGTCCATGGGAGCAAGATTAGTGGCTGCTAAACTTGAACCGAAAAGCTTCAAACATACCCATATAGATAAACCAGACTGCTCAGGGCCCCCCATGGACATAAGTAACAAGGCTTCTGGGGAGATAAAAATTGCCTATACTTACTCTGTTAGCTTCGAGGAAGATGATAAGATCAGATGGGCGTCTAGATGGGACTATATTCTGGAGTCTATGCCTCATACCCACATTCAGTGGTTTAGCATTATGAATTCCCTGGTCATTGTTCTCTTCTTATCTGGAATGGTAGCTATGATTATGTTACGGACACTGCACAAAGATATTGCTAGATATAATCAGATGGACTCTACGGAAGATGCCCAGGAAGAATTTGGCTGGAAACTTGTTCATGGTGATATATTCCGTCCTCCAAGAAAAGGGATGCTGCTATCAGTCTTTCTAGGATCCGGGACACAGATTTTAATTATGACCTTTGTGACTCTAT |
| hsa_circ_0100594 | ACTCAGATTGATGGACAACTTTTCTTAATTAAGCACCTTTTGATACTTCGTGAACAAATTGCTCCATTTCACACTGAATTCACCATTAAGGAAATTTCCCTGGACCTCAAGAAAACTAGAGATGCAGCATTTAAAATCCTGAACCCTATGACTGTCCCAAGATTTTTTAGGCTGAATAGCAACAATGCCTTGATAGAGTTCTTGTTGGAGGGTACTCCTGAGATAAGAGAACATTATCTTGACTCTAAAAAAGACGTAGACCGTCATCTGAAATCGGCCTGTGAGCAGTTTATTCAGCAGCAGACCAAGCTGTTTGTAGAACAGCTGGAGGAGTTCATGACAAAGGTTTCAGCGTTAAAAACAATGGCCAGTCAGGGAGGCCCCAAGTATACTCTCTCACAGCAGCCTTGGGCACAACCAGCAAAGGTCAATGACCTTGCGGCAACTGCATATAAGACAATAAAAACAAAGCTGCCTGTGACATTGAGAAGTATGTCCTTGTACCTATCCAATAAAGATACCGAGTTCATCTTGTTTAAACCTGTGAGGAATAATATTCAGCAAGTCTTCCAGAAGTTCCACGCTCTGTTAAAGGAAGAGTTCAGCCCTGAAGACATCCAGATCATTGCCTGTCCATCTATGGAACAGCTGAGCCTTCTGCTGTTGGTTTCTAAATAAGCAGGCCAGCCGGGCTGTGCACCTAAATGTCTGTCTGGGAGGAGCAGGCTGAGAAGTCTTGCAGTCTGCAGGACACCGAGGAATC |
| hsa_circ_0101085 | CTGTGACTAATACTACTGTAGAGGATGAGACTGAAACAAATGAAGTTCAAGGATTTCTCTTTGGGAAACTAAAAGAAATATATTCAGATCTTAGAGATAATCTGACAGCATTCCAAAAATACCTGATTGAGAGTAACAAACAAATGATGCCTTTGAAAGTCTGGGAACTACAAGATCCTGTTAAGACTATTGCATGTTTCATTGATAAATGTTTAATGGAAACATCAAATGGAGGACATGCAGTTTTACAAAATCTTAGTTTTCAAGCAGCTTCTCAAATAATGTCCGCTCCAGTTTATGATTCCATTAAATTAATGAAAGACATTTCACAGAACTTCCCCATAAAAGCCAG |
| hsa_circ_0101220 | AATGAAAACCATGTAGCTGGTCTTATTATTCTCTAATCATGAAGACCATTATTGTTATTTGTGGACTAAGGAGCAGAAAGACTGATGTAAGTAAACATCAAATTGATGTTTTGGGCTTGGACTGTATTCATTCATGGGAACCCACTACATTATTTTGATAAAATAAATGGTTAAATAACACATGAAATTAAGGAATGCCAGTCATGGACCTATGATTGTAATGTGTCATCAACAAAATTTTATGATTAATTCAACCCAACCCAACTGAAGACCATTGTTAAATAAAGGAAGAGCATTTCATATATTGTCATAGTAATTTAAATTCCCATTTAATGTTTTTGCTATAGTTGCTTAACATATTCCTTCACGGACAGACATTGAAGTTGTTGAGTTTTTGAGGTAGAGGTACCTTGGGAGATATTATATATTAATTTGCTTAATTGTGGGCTCACATGGTAGATCCATGGTTTGCAATCACATTAATGAGTAGACAAATCAAGTGGAAGGGAAGCTGGTAGACACCAATCATAAGTATGAGATTTAAATGCAGCAGAGTCGCTGATGAATCTCTATGTGTAAACTTGAGGTTGATCACAAAATATGATCTAGTCTATTTGTTCTGCATGAAAGAGAGTAATGAACAGATCTTGGTTCTATTAAAGACTTCTGTTCTCAGTTTTGTGTCATTAAGGGATATTGATCATATAATTATTATTCCTGAAACCATGAATGACTGAGTCAGTTCAACAAGGTATAAATGAATGAAGAAATAGAATGCCTAGCCTGGATCAATGATGAGTATTGGTGGAGGTGTCTGAATCAACACTTTTGATTAAGCCCTCTGTGTAACTCTGAGATCTGTTATTGAATGAGAACCAGCCTATATTCTCTGTAGAAATTACCTAATAGAAGTTAGGAAAATTCAATAAAACTTGTTCCTGAACTTTTGTAGTTCAAAGATATGGAATGTATTATTTTAAGTGAATTCAATGCGTAAATAGAGAAATGTGTATACATGAGAGAAGATTAACAGTCCTACTTATGGAACTATTCTTTGAGTACAATGAAAAAGTATTATAAATATTAACACTGGCAATAAAACAAGTATAATTATTAATCTGAGGTAAAATCCTATTATACAGAACATATTGTATCTGCAGTTGAGACAATTTTTTTGGTTTTATAGTTTGCTTTTTTTTTAATTAAGAAAAGGGAAAGTGTTTGCATCAGCCAGAAATCCATTAAGTAATATCTACTTAACAGATTTTTATATAGACAAATATTCATAATCATTACAGATCTTATTCTTGGGCTAGGTGACGCATTAGTGTCTATTATACTATTTAAAACTAAAAGTTCAATACTGGCCTGAAAAAATTGCATTTTTGAGCCAAGGTTTATTGTTATCTAGTTCTGCTTGACTAACATCCATTGAGAAAACCGTATATCTATTCTAAATCTGCCTTTTTCCCTCCAGAGTGGACATAGGCCAGCTGATTTAATAATTTAAGAAAGAACTGTTATCCTGAGTAAGGAGCATAGAGACAAATATAAGTAAGTTCCTTATTTCAAGTTCTTTCTTTGAATGGTTTGTTTTTGGGTGTTAGCTAAAGGATTTCAAAGGAATCAATGTCTACATACGTGAAATAAATAACACACCCCAAATGCACCAATGATGGGAATGTGTAATGAACCATGAATTATGTTTAATCTAAATCGTCACAACTGAGTTGATTATAAAAGAAGACAAAAGTTAACTTATTCTCAGAGCAGCTCAACATTATTCTTTATTTCATGCCATGGTTATTTTACACGTTCCCTTATGGGTAGAGATCCTGGCTGACATCCGTTTTGGGGCAGCGCATGAGAATATGGAAATAGGTGATTTCGTTGTTTTTTATCAGGCCATCGTTGACATATGTATGAATTAAATAGAAGTGGGCTATATCAGAACGAATGCTGAATGTGAACTGAGAGCATGCAGGGGCATTGACCAATTCACATGTGCACCTTTACGTGTCATGGCCTAAGAAATGTAGTCTAGCTCTATGGGCCAAGAGCCTAAAATTTAATGATCTTGTTTTGGGGATTTTTGATTGTATCGTTATTTGTGAGTCAATAGGTCAATCAATAGATGAAAAAAATGCACATCATGGACCAGTGATGAATATCATGGGGTTTCTGAAACAACATTTTTGATTAAACCCATCTGCAACTCTGAGGTCCATTACAAAGAGACACCTCTTTATTCTTCATGGAAAATGAGTCTAGGACCTTGTTCATGGGCCTTTTGTGGTTTGAGGGTATGAATTAATTATTATAATAAACTTAGTCCATGAATATAGAATTTTATCAATGAATGAAGAATAAGAAAGGTATGCATGCACTTACCTACTAAGGCATGTAATGCAACATGGATTATTATTGAAACAATTTTGAGGTTCTTAGTCCCAATAAATTAGTGGCTAAGGCTCTATTCATTTTGGTAAGGAGCATATGGTCATCTGTGCACAAGACTGAATGTTTTTGTTCTGTGTTGAGGCAGGTTACTGGTTGATCATTTTATTAAGGAAAAAGAAATACATTTACATAAATAAATAGTTGACTGAGTAACTGTAATTGAGAGAGGGAGAGCATGGAATGATGATGAGATCTATATACAACGACACTCATGATTAATTCAGTCTTTTATGGAATTTTACTTAAGCAAACAGACAAGTTTGGTACTAGATTCACAGCGTTTATTATATTATCTAAAACAAAAAATTTCATGGTGAGAGATATTTCATCTTGACAAAAACAATTTATGTAAGTTAATAAATAGAAGTGTTCTCTCTTCTCAAGTGTTCAGAATAGGGTACAAAACAAGGTTTATGATTGTTTACTACAGAGCTATGATTCATTTTAAAGAGCTAAACATATAGTATTTGATTCTTTTTAAAATTTGCGTTTTATGCTGCAGAATGAACGCACTCTACCTGATTTAATTATAGTCTTCTGGTGAAGATCTTGCTGTTATCTCTGTGAATCAGGAGAGAAGAGATTGATGTAAGTAAAAATCAAACTTTGTGTTCTCGCTTGGGAAACTTGATCAAGTTTATTCATTGAATTATTTCAAAGGAATAAAAGGCTACATACATAAATGAGTCAAAGGTGGTTATGCATGGAACAATGGTAGAAATGTGTTATGAATTAAGAATTATGATTAATCCATTTCAGCACAACGAAATTCATTAGGACAAAAAGTTGAAAGGTATTTCTGTCATTTTCAGAATGCCTTAATATGCTGTTTTTGTACCATAGTGGATTTAAATACTTCCCCATTGTGATTGTTATTGACAGTTAACTAATTGAGGGAAGAACATAAGAAAATGGAAAACCTTGCCTATAGCTGTGGTATAACTTGGTGGTTATACCATGATATAACCATAGTAACGTCTTAAAAATACGGATGTTAAATGAAAAAAGCCCAACCATGGACTAATGGAATATATGGAATTGACGTGGTGCAATTTGTGGAATGAATTAATTCACCTTTGCAATACTGAGGCTCATTACAGAGAGAGCCAGTCCTCTAGTTCTGTGGAATATGAATCTTACAGACATAGTTACTTCTTCAAGGATCTTTTTGCCCTCTAATTCATGGATAGTGATTTTACTATTAGTGAATCAATGCCTAAATCAAGAACAGTATAATTGAATTAAAGCACAGAATACCAAGTCTGGATCAATGATGAGTATGCGTGGGGCATCTGAATCAAATATTCTGATTATACCCTGTCTGTATCTCTGAGGTCCATTGCATCTATGGGAAATGTGCTTGGAGAGAAAGTTAGACATATTCAAGGAATATGTTTTTGGTCCTTTTTGGTTAATGGGTATAGATTGTATTATAAGTAAATCAATGAATGAATAAAGAAAAATACAAATAAAGACAAGGAAGGCTTCATTTCATTCAATTATGAAAGGAGCTAATGATGTTTTGAGTTTATATAAGTCTGCTCCTCCTTCGAGAGCATATAGATAGATATGCACTCAGTGTTCCGAGTTAGTGGCAAGTTTCTGGTTGCGTGTCTTGTTTGATTTAGAAAGGGATAAAACATATACTTAAATGATGTATTTAGTTTAATAATACTTAATATAATAAATATTAGCCAATAAAATTAACATTCATATTCAATAATAACAATATTGCAAATCATGCTAAACACATTAACATAATTTTTATAATAAACAACAATTTAATACAATTAATAATAAACACTAAAATACTAATGATTAGGTTGTGAAATGTGGAGAAAGTCTAAGGATGAACGTTGAAAATTTGAATATAATAACCAAAAATCCTCTTATGTTTGCTCATTTTTATTTAAAAAGAAAAATGCAAATGCATTTTTATGACTTTCATGAGTAATAAGTGAATAAATAGATGCTAGTGTCATGAAGGGTTTTTGTCCAGTGTCTTCTGAATTCATGAATAGTGGCACTATTATTATGAGTAAAAGAATCATTTTATATCTCCATCCCTTATTTAAAAGAGGGCAAATCATGGACTAGTGAGAAGCATATGTAATGTATGAAAGGGTGTGATTAATCTTGTCTATCCTCTTGAGGTTTACTTAAACAAAAAGGTCCAGGACCCTCCTTCCTTTGGAAGGGTTGTGAAGACCCACTCTCTAGACATATTTTGATATTAACATTTAAATGGAAATTAATTAAAAATAAAAATTCAGTTCCTCAGTCGCACTAGCCACACCCCAAGCCACATGTCGCAAGTGACCACCATTTCAGAATTGCCAATAGAGAACACTCGTATCACCGAAGAACGTTCCCACAGTGCATACAGGGCTAATATGAACAGTTGCAGGTAATAGTATTTAATTTTAGGTAGCAGGTGTCTTCACCATTTTGAGTTTTCTTTAGAAAGGAGAGTAGATAATTAAATTATTTTTTGAGTTATTAAATAGATGAGTAAATAAACAAATCAGTAAATAGTTTGAATTAAAATAACTGTGGCCCACTGGTCAGATTGTGTTATAAAACAACAATTTAAATTCGTCTTATAGTGCAAAAGTTTTCCTCATTAAAAATGTGTCTTCCCTTTATGCTAGGAATACCTAGGCAGATATTGAAAACTGCTCTTCTAGTGTTTGGTGGTAGATTCAAAAATGTTCATGATATTATTTCATGCTAAGAATTATGTCAATACATGAAAAAGAACTACTGTTGGGTTGATGATTAAAATTTGACACAAACCAATGTTTATGATCCATCCAGTAATGGAGAACTAACTTCTACTTAAAAATGGATTTTAAAATGTACACTTTTTATTCTTATAAAAATCTGCCTTTTCCTCCAGAAGGAATAGACGAGATTGGATTTGGTCATTTCCCCAAGGTGAAGAGCATGGCGATTTCTTCTGTGGTAAGGAGCAAT |
| hsa_circ_0101420 | GAGAGTAGTCTGAATTGGGTTATGAGGTCCCCTGCGGGGTACCTCACCTCAGCCATTGAACTCACTTCGCTGGCCGTGAGTCTGTTCCAAGCTCCGGCAAAGGAGGCATCCGCCGGGCCCCTCCCCGAAGGGCGGG |
| hsa_circ_0101423 | GCGGCCCTAACAGGGCTCTCCCTGAGCTTCGGGGAGGTGAGTTCCCAGAGAACGGGGCTCCGCGCGAGGTCAGACTGGGCAGGAGATGCCGTGGACCCCGCCCTTCGGGGAGGGGCCCGGCGGATGCCTCCTTTGCCGGAGCTTGGAACAGACTCACGGCCAGCGAAGTGAGTTCAATGGCTGAGGTGAG |
| hsa_circ_01017 | GTGAACGGTGTGGACATGAAGCTGCCCGTGGTGCTGGCCAACGGCCAGATCCGTGCCTCCCAGCATGGTTCAGATGTTGTGATTGAGACCGACTTCGGCCTGCGTGTGGCCTACGACCTTGTGTACTATGTGCGGGTCACCGTCCCCGGAAACTACTACCAGCAGATGTGTGGCCTGTGTGGGAACTACAACGGCGACCCCAAGGATGACTTCCAGAAGCCCAATGGCTCACAGGCAGGCAACGCCAATGAGTTCGGCAACTCCTGGGAGGAGGTGGTGCCCGACTCTCCCTGCCTGCCGCCCACCCCTTGCCCGCCGGGGAGCGAGGACTGTATCCCCAGCCACAAGTGTCCTCCCGAGCTGGAGAAGAAGTATCAGAAGGAGGAGTTCTGTGGGCTCCTCTCCAGCCCCACAGGGCCACTGTCCTCCTGCCACAAGCTGGTGGATCCCCAGGGTCCCTTGAAAGATTGCATCTTTGATCTCTGCCTGGGTGGTGGGAACCTGAGCATTCTCTGCAGCAACATCCATGCCTACGTGAGTGCTTGCCAGGCGGCTGGAGGCCACGTGGAGCCCTGGAGGACTGAAACTTTCTGTCCCATGGAGTGCCCTCCGAACAGTCACTACGAGCTCTGTGCGGACACCTGCTCCCTGGGCTGCTCAGCTCTCAGTGCCCCTCCACAGTGCCAGGATGGGTGTGCTGAGGGCTGCCAGTGTGACTCCGGCTTCCTCTACAATGGCCAAGCCTGCGTGCCCATCCAGCAATGCGGCTGCTACCACAATGGTGTCTACTATGAGCCGGAGCAGACAGTCCTCATTGACAACTGTCGGCAGCAGTGCACGTGCCATGCGGGTAAAGGCATGGTGTGCCAGGAACACAGCTGCAAGCCGGGGCAGGTGTGCCAGCCCTCCGGAGGCATCCTGAGCTGCGTCACCAAAGACCCGTGCCACGGCGTGACATGCCGGCCACAGGAGACATGCAAGGAGCAGGGTGGCCAGGGCGTGTGCCTGCCCAACTATGAGGCCACGTGCTGGCTGTGGGGCGACCCACACTACCACTCCTTCGATGGCCGGAAGTTTGACTTCCAGGGCACCTGTAACTATGTGCTGGCAACAACTGGCTGCCCGGGGGTCAGCACCCAGGGCCTGACACCCTTCACCGTCACCACCAAGAACCAGAACCGGGGCAACCCTGCTGTGTCCTACGTGAGAGTCGTCACCGTGGCTGCCCTCGGCACCAACATCTCCATCCACAAGGACGAGATCGGCAAAGTCCGGGTGAACGGTGTGCTCACAGCCTTGCCTGTCTCTGTGGCCGACGGGCGGATTTCAGTGACCCAGGGTGCATCGAAGGCACTGCTGGTGGCTGACTTTGGACTGCAAGTCAGCTATGACTGGAACTGGCGGGTAGACGTGACGCTGCCCAGCAGCTATCATGGCGCAGTGTGCGGGCTCTGCGGTAACATGGACCGCAACCCCAACAATGACCAGGTCTTCCCTAATGGCACACTGGCTCCCTCCATACCCATCTGGGGCGGCAGCTGGCGAGCCCCAGGCTGGGACCCACTGTGTTGGGACGAATGTCGGGGGTCCTGCCCAACGTGCCCTGAGGACCGGTTGGAGCAGTACGAGGGCCCTGGCTTCTGCGGACCCCTGGCCCCCGGCACAGGGGGCCCTTTCACCACCTGCCATGCTCATGTGCCACCTGAGAGCTTCTTCAAGGGCTGTGTTCTGGACGTCTGCATGGGTGGTGGGGACCGTGACATTCTTTGCAAGGCTCTGGCTTCCTATGTGGCCGCCTGCCAGGCTGCTGGGGTTGTCATCGAAGACTGGCGGGCACAGGTTGGCTGTGAGATCACCTGCCCAGAAAACAGCCACTATGAGGTCTGTGGCTCACCCTGCCCGGCCAGCTGTCCGTCCCCTGCACCCCTTACGACGCCAGCCGTATGTGAGGGCCCCTGTGTGGAGGGCTGCCAGTGCGACGCGGGTTTCGTGTTAAGTGCTGACCGCTGTGTTCCCCTCAACAACGGCTGCGGCTGCTGGGCCAATGGCACCTACCACGAGGCGGGCAGTGAGTTTTGGGCTGATGGCACCTGCTCCCAGTGGTGTCGCTGCGGGCCTGGGGGTGGCTCGCTGGTCTGCACACCTGCCAGCTGTGGGCTGGGTGAAGTGTGTGGCCTCCTGCCATCCGGCCAGCACGGCTGCCAGCCCGTCAGCACAGCTGAGTGCCAGGCGTGGGGTGACCCCCATTACGTCACTCTGGATGGGCACCGATTCAATTTCCAAGGCACCTGCGAGTACCTGCTGAGTGCACCCTGCCACGGACCACCCTTGGGGGCTGAGAACTTCACTGTCACTGTAGCCAATGAGCACCGGGGCAGCCAGGCTGTCAGCTACACCCGCAGTGTCACCCTGCAAATCTACAACCACAGCCTGACACTGAGTGCCCGCTGGCCCCGGAAGCTACAGGTGGACGGCGTGTTCGTCACTCTGCCCTTCCAGCTGGACTCGCTCCTGCACGCACACCTGAGCGGCGCCGACGTGGTGGTGACCACAACCTCAGGGCTCTCGCTGGCTTTCGACGGGGACAGCTTCGTGCGCCTGCGCGTGCCGGCGGCGTACGCGGGCTCTCTCTGTGGCTTATGCGGGAACTACAACCAGGACCCCGCAGACGACCTGAAGGCGGTGGGCGGGAAGCCCGCCGGATGGCAGGTGGGCGGCGCCCAGGGCTGCGGGGAATGTGTGTCCAAGCCATGCCCGTCGCCGTGCACCCCAGAGCAGCAAGAGTCCTTCGGCGGCCCGGACGCCTGCGGCGTGATCTCCGCCACCGACGGCCCGCTGGCGCCCTGCCACGGCCTTGTGCCGCCCGCGCAGTACTTCCAGGGCTGCTTGCTGGACGCCTGCCAAGTTCAGGGCCATCCTGGAGGCCTCTGTCCTGCAGTGGCCACCTACGTGGCAGCCTGTCAGGCCGCTGGGGCCCAGCTCCGCGAGTGGAGGCGGCCGGACTTCTGTCCCTTCCAGTGCCCTGCCCACAGCCACTACGAGCTCTGCGGTGACTCCTGTCCTGGGAGCTGCCCGAGCCTGTCGGCACCCGAGGGCTGTGAGTCGGCCTGCCGTGAAGGCTGTGTCTGCGATGCTGGCTTCGTGCTCAGTGGTGACACGTGTGTACCTGTGGGCCAGTGTGGCTGCCTCCACGATGACCGCTACTACCCACTGGGCCAGACCTTCTACCCTGGCCCTGGGTGTGATTCCCTTTGCCGCTGCCGGGAGGGCGGTGAGGTGTCCTGTGAGCCCTCCAGCTGCGGCCCGCATGAGACCTGCCGGCCATCCGGTGGCAGCTTGGGCTGCGTGGCCGTGGGCTCTACCACCTGCCAGGCGTCGGGAGATCCCCACTACACCACCTTCGATGGCCGCCGCTTCGACTTCATGGGCACCTGCGTGTATGTGCTGGCTCAGACCTGCGGCACCCGGCCTGGCCTACATCGGTTTGCCGTCCTGCAGGAGAACGTGGCCTGGGGTAATGGGCGAGTCAGTGTGACCAGGGTGATCACGGTCCAGGTGGCAAACTTCACCCTGCGGCTGGAGCAGAGACAGTGGAAGGTCACGGTGAACGGTGTGGACATGAAGCTGCCCGTGGTGCTGGCCAACGGCCAGATCCGTGCCTCCCAGCATGGTTCAGATGTTGTGATTGAGACCGACTTCGGCCTGCGTGTGGCCTACGACCTTGTGTACTATGTGCGGGTCACCGTCCCTGGAAACTACTACCAGCTGATGTGTGGCCTGTGTGGGAACTACAACGGCGACCCCAAGGATGACTTCCAGAAGCCCAATGGCTCGCAGGCAGGCAACGCCAATGAGTTCGGCAACTCCTGGGAGGAGGTGGTGCCCGACTCTCCCTGCCTGCCGCCGCCCACCTGCCCGCCGGGGAGCGAGGGCTGTATCCCCAGCGAGGAGTGTCCTCCCGAGCTGGAGAAGAAGTATCAGAAGGAGGAGTTCTGTGGGCTCCTCTCCAGCCCCACAGGGCCACTGTCCTCTTGCCACAAGCTGGTGGATCCCCAGGGTCCCTTGAAAGATTGCATCTTTGATCTCTGCCTGGGTGGTGGGAACCTGAGCATTCTCTGCAGCAACATCCATGCCTACGTGAGTGCTTGCCAGGCAGCTGGAGGCCAGGTGGAGCCCTGGAGGAATGAAACTTTCTGTCCCATGGAATGCCCTCAGAACAGTCACTACGAGCTCTGTGCGGACACCTGCTCCCTGGGCTGCTCGGCTCTCAGTGCCCCTCTGCAGTGCCCAGATGGGTGTGCTGAGGGCTGCCAGTGTGACTCCGGCTTCCTCTACAACGGCCAAGCCTGCGTGCCCATCCAGCAATGTGGCTGCTACCACAATGGTGCCTACTATGAGCCGGAGCAGACAGTCCTCATTGACAACTGTCGGCAGCAGTGCACGTGCCATGTGGGTAAAGTCGTGGTGTGCCAGGAACACAGCTGCAAGCCGGGGCAGGTGTGCCAGCCCTCCGGAGGCATCCTGAGCTGCGTCAACAAAGACCCGTGCCACGGCGTGACATGCCGGCCACAGGAGACATGCAAGGAGCAGGGTGGCCAGGGCGTGTGCCTGCCCAACTATGAGGCCACGTGCTGGCTGTGGGGCGACCCACACTACCACTCCTTCGATGGCCGGAAGTTTGACTTCCAGGGCACCTGTAACTATGTGCTGGCAACAACTGGCTGCCCGGGGGTCAGCACCCAGGGCCTGACACCCTTCACCGTCACCACCAAGAACCAGAACCGGGGCAACCCTGCTGTGTCCTACGTGAGAGTCGTCACCGTGGCTGCCCTCGGCACCAACATCTCCATCCACAAGGACGAGATCGGCAAAGTCCGG |
| hsa_circ_0102236 | GACTTGGAAAACAGTGTGGGTGAACTTAGTGAAGGACAAAGACCCCAGCTAACAGCGGCAGCAGAGAACATCTTAATGGGACATTCTCTCTATATGCAGCCACCTGTCACTAATACACAGTCTTTGGATCAACAATGTGATCCTAAACCATTATCTCGGCAATTTGACACAGTTTCAGGTAGTATTTATGAAGATTCATGTGCTAGTCATGGTCCAATGAGTTTGGGAGAATTGGAGTTGGAGCCAAATTCTAAGCTGGTTCTTCCCACAACACTTCTGACAGCACAAGAAAATGATGTTAATTTACCAGTAGCCGCTGAAGATTTTTCCCAGTACCAACTAAAGCAAAATCAGGATGTTAAGCAAGTTGAACACAAACCATCACAAAGTTACCTACGTGTTAGAAATAAATCTGATATTGCACCTTCACAGCAACAAG |
| hsa_circ_0102537 | GTTTTGAGAGATTACCTGAAGTTGCACTCAAAAGCAAGATATACTACTAACTTTGAAATGGAATAAGGTGAGTTGGTGGATTAGTTCCAAAGTCACATTGCAGGGAGGAAATGTCACTCAAACTGTCATTCTTAGAAGACCTGCTATTTGGCCAGGCGCTGTGGCTCACGCCTGTAATCCCAGCACTTCGGGAGGCTGAGGTGGATGGATCACCTGAGGTCAGGAGTTTGAGAACATTCTGACCAACATGGTGAAACCCTGTCTCTACTAAAAATACAAAAATTAGCTGGGTGTGGTGGCACACACCTGTAATCTCAGCTACTTGGGAGGCTGAGGCAGGAGAATTGCTAGAACCCAGGAGGTGGAGGTTGCAGTGAGCCGAGATTGTGCCATTGCACTCCAGCCTGGGGGACAGGGCGAGACTCCATCTCAGGGAAAAAAAACAAAAACAAAAACAAAAACAAAAAACCCTGCTCTTTATAGCAGAATTTTGATTTATCAAATGTCCGATATTTTCTGCCCACTGGCTTTTCCCTTTTTGAAGTGGAAAAGGAAAATATGGGAAATGCTTGTGCAGTTTTAGTCATTGATTTCTGAATTCATTGTTGTCATCTTTGCAGATGAAATAGTTTGGATAATTTTCTGGCTACTTAAGAAGGTTTTCTGTTACATGTTTATGTTTTCCCTCCATAGTGAATGCATTTAAAATCTTCTCTATGTTTCTTGAAATTAGGTGAGAGCTTATACTTCAACCCATGTTCTAAGAATCTTGTTAACTCTAATGCATTGGTGTATAGTTTGGGGTGCCAGGCTTTTCCTGAATAATTGAGAAATGATCAGATGATCTACCCTTTGACTGACGTATACTGGTATATGCATTACTATTGCTCTTCGAGTGACTGTACTGGCTTACTCATTTCTTAAACATTTTAAACAATTACAAAAGGATATTCTTTTATTGAGAAAAACAACAGCTTAATGCAGATTAAGGGAGACGTATTTTATACAGGCCAAAAATACTGTCATATGTTAAATTCTGGGGTTGTATAGGTTTCATTATACTTAACCTTGATCTCAGCCAGATGCTGAAACAAAGACCTTGGAGTTTTTCTCATTTGTTGCTTTGTCAGAAGAAAATTTCCTAAGGAGTCAGCTCTTCTGGAGTGACTGCATCCTATAACGGATGGTCAAGTTATCTGACCATCTCCCCTCCATAAACCTTTGTGCAGAGCTTAGGGGATGGGTAATTGGAGCACTGCTTTTAAAGTGGCTTGCTATTTGTGACCTTCCTTGAATTTCAGTAAGGGAAATTTGGGAATCATTATCTCTGAAAATGTTTTTAAAGTACATTAGGGCACTTTTCTGGGTTACATTTATTTGACCGTTCCCAGCATCATTCTTCTTTAATAGAGGTGGAATCTAAGATTTTTAGATATCTGTAATTTTTAGCCATTTGAAAAATCCAGGCTTATAATAGCAACACAACTGAAAAACTGTCTTTGAAAACATAACATCTTTGGAACTGCCAAGAATTGCTTAATGGTGAAGCAAGCAGCCTGCAGGAATGCTCTGTGAATGTAGCTCTGCTCTGGTACTTCGAGAGGGAAGAAAAGTCAGTACATATTATTGAGTGCCCTGTGATTTTTACCTCAATCTTGGAAGCTGTTTTTCTAGATATACTTTTTTTTTCCCTCCAGAATAGCTATATGTTATTGAAATGTGCACCTTCAGTGCAGTTCTCAAATGTCTTAATTATTTTTTTTTTTTTGAGATGGGATCTCACTCTGTCTCCCAGACTGGAGTGCAATGGCATGATCTCGGCTCACTGCAACCTCTAACTCCCAGGTTCAAGCGATTCTCCCGCCTCAGCCTCCCAAGTAGCTGGGATTACAGGCGTGTGCCTCCACACCCAGCTAATTTTTGTATTTTTAGTAGAGATGAGGTTTCGCCATGTTGGCTAGGCTTGTCTTGAGCTCTTGACCTCAGATGATCGGCCTGCCTCGGCTTCCCAAAGTGTTAGAATTACAGGCGTGAGCCACCATGCCCGGATGTATTAATGGTATTTTAATAATAACTCTTGGTTGATAAACTTAAACATGACAGTTTTGAGCAGGCACCTTAAGTTCTGTATACTACCGTGAGGCCAGGCACTTGTTTAGCTTCTGTGTCATACAGCTACCCCTTGTATTTTGTTCTTTTTTTTTTTAGTATAATAAATCTATAGTTTTATTAACACAGAAACTGATAGTGTGGTATGAGGTTTACGTTTAAACAAAGTTTTCACAGAAATCTAACACATGCCTAAAAAGATTTTACAATCTAGCTCTAGATGCAAGTCCAGACAATATCAAGAACTGATGGATCTCATGACTCAAGACAGAGCATTTTGGGTTTCAGTTACTTGTTAGGATTTCTTAAAAATGGTTTTTGTTTTTTTTTTATGGGTTTTAATACACCTTGTCTTTTGAATTGAGTTGCTTCGTTTGATAACTTGAACCTCTGGGCACTGAAATATCTTCTTTTAATAGAAGTGATATCTGGTAATGCTGGTACAGTATCTCTAATAGGTCAGGTGGGAGATTCTTCTGTTATACATCCATTCTTACTCAGGTCTCCTCAGGCAACACAGGAGGAGGCCATGACTCAACAGTTCTCCCAACCTGTTGGTGACATGACTCTTCGCCTTCTAAGTGTATACTTCCTTCCTAAGTGCTATTTCTCTACTTAAGGGATATCTAAAACAATTTCTATACTCTAAAAGAAAGAAAAATCTACACACACACACACACACACACACACACACACGCATGCACACACACACAACCTTGTATATATATAGTTTTATGTGTACCTTATTAAATCCATCATTCTTTTTTAAATTATTTTTTGAGCAGCATTATTCTAGAATATAAATACTGAATTGAATAAGCATACTTTTTTCTTTTCCTTTAAACACACTTTTAAAATACAGTTTTTAAAATTAAATAGAAACATGGTCTCACTGTGTTGCCCAGGCTGGTCTGGAACTCCTGACCTCAAGTGATCCTTCTCCGTTGGCCTCCTGAAGTGCTTGGAATAAAGGCATGAACCACCGTGCCTGGCCTGCATGTAACTTTCGACTCCTCCAGAACTTAACTACTAGTAGCCTACAGTTGACCAGAAGCCTTACCAATAACATGAACAGTTGAATAACATGCATTTTGTATGTTATATGTATTATATACTGTGTTGTTACAATAACATAAGCTAGAGAGAAGAAAATGTTATTAAGAAAATCCTATTGGGAGGCCGAGGCGGGTGGATCACGAGGTCAGGAGATCGAGACCATCCTGGCTAACACGGTGAAACCCCGTCTCTACTAAAAATACAAAAAATTAGCCGGGCGAGGTGGCGGGTGCCTGTAGTCCCAGCTACTCGGGAGGCTGAGGCAGGAGAATGGCGTGAACCCCAGGGGGCGGAGCCTGCAGTGAGCCGAGATTGCGCCACTGCACTCCAGCCTGGGCGACAGCGAGACTCCGTCTCAAAAAAAAAAAAAAAAAAAAAAAAAAAAAAAAAAGAAAATCCTAAAGAAGAGAAAATATATTTGCTATTCATTAATGGAAGTGGATCATAAAGGTCTTTATCCTCATTGTCTTTGCATTGGGTAAGCTGATGAGGAGGAGGGAAAGAAGAGATTGGTCTTGCTGTCTCCAGTGGCAGAGGTGGAGGAGGAGGAAGGGGAGGCAGGAGAGGCAGGCACACTTGGTGTAACTGTTATTGAAAAAATCCACTTTTAAGTGGACATGCACAGTTCAAACTCGTATTGTTGAAGGGTCAACAGTGCTAGACAGTTTTATTTTCTAAATGCTAACTATAATACAGTATAAAAATACTAAAAATGTATAGTAGCTAGGGGATGATTAGAGAATCTTCCTTTCATCTTTGGCTGTGACTAGTTTGAATTGAGTTTGACATGGCCTGAGGTCACTTCAGAATTATTTTGTTACACGTTTTTATTATCCAAAGCCCATTTCTTTGTTCTTAAAGAAGAAAATGATAGGAAATCCTTTAGCCTAACAAACATTTTTATCAGCCAGTAGAATGGTGATCTGACTCTTTCGTATTTACTAATGCTGTTGTACCCTTTTTCTCTTCAGTAGGAACAACTTATTCTCACTCAGCTTTTATTTTTATGTTCTCCAGAACATAAGAGAGCAGCTGCCCATGCCAAATAGTTACCTCTTTTAAACTCATTCTACTGGAGCAAATG |
| hsa_circ_0103033 | GATGAAACAAGTGAAGATGCTAACTGTCTTGCTTTGAGTGGACATGATAAAACAGAAGCAAAGGAACAACTTGATACAGAGACAAGTACAACTCAATCAGAAACTATTCAGACAGCGGCTTCTCTGTTGGCCTCTCAGAAAACATCCAGTACAGACCTTTCTGATATCCCTGCTCTCCCTGCAAATCCTATTCCTGTTATCAAGAATTCAATAAAACTGAGATTGAATCGGTAAAAACAACCTCAGGGGTCCATAAACAATATCTGCCAACTCAACCTGTTGTCTTCAAATGCTAAAAAAGGAGAATGGAGGGTACAAGACTAGACATGACTGAAATGGATTTGGGTTTTTTGGTGACCTCCCTTACTGGGCTAATCAGCACTTGATCGGAAGTCCAG |
| hsa_circ_0103455 | ATTGAGATTCTAATGTATTGCCAACTGACCAGCCGACAGAAGCTGCTATATCAGGCACTAAAGAACAAAATTTCCATTGAGGATTTATTGCAGTCTTCTATGGGCTCTACCCAACAAGCACAGAACACCACCAGCAGCCTCATGAATCTGGTCATGCAGTTTAGGAAGGTGTGTAATCACCCGGAGTTATTTGAACGGCAAGAAACTTGGTCTCCATTTCATATTTCCCTAAAGCCATACCACATTTCAAAGTTTATCTACCGTCATGGACAGATCAGGGTCTTCAATCATTCACGAGACAGGTGGTTAAGGGTTCTTTCTCCATTTGCACCAGACTATATCCAACGGTCTCTCTTTCACAGAAAAGGTATTAATGAAGAAAGCTGTTTCTCTTTCCTTCGCTTTATTGATATATCTCCAGCAGAAATGGCAAACCTTATGCTTCAGGGACTTTTGGCCAG |
| hsa_circ_0104297 | ATTTGGAAATAGTGGGATTTATTGATATAGCTGATATTTCAAGTCCCCCAGTTCTGTCCAGACATCTGGTCTTACCTATAGCACTTAACAAAGAAGGTGATGAGGTGGGTACTGGCATCACTGATGACAATGAAGATGAAAATTCAGCCAATCAGATTGCAGGCAAAATACCCAACTTTTGTGTCCTGCTCCATGGTAGCCTAAAAGTGGAAGGAATGGTAGCGATTGTTCAATTAGG |
| hsa_circ_0105876 | AACGGTGTGCTGCTGAGGACTGTCTTGGACCCTGTCACTGGGGATTTGTCTGATACTCGCACTCGGTACCTGGGGTCCCGTCCTGTGAAGCTCTTCCGAGTCCGAATGCAAGGCCAGGAGGCAGTATTGGCCATGTCAAGCCGCTCATGGTTGAGCTATTCTTACCAATCTCGCTTCCATCTCACCCCACTGTCTTACGAGACACTGGAATTTGCATCGGGTTTTGCCTCGGAACAGTGTCCCGAGGGCATTGTGGCCATCTCCACCAACACCCTACGGATTTTGGCATTAGAGAAGCTCGGTGCTGTCTTCAATCAAGTAGCCTTCCCACTGCAGTACACACCCAGGAAATTTGTCATCCACCCTGAGAGTAACAACCTTATTATCATTGAAACGGACCACAATGCCTACACTGAGGCCACGAAAGCTCAGAGAAAGCAGCAGATGGCAGAG |
| hsa_circ_0106002 | GATCAGAAGTGGTTGATCTTGATGGAAAAAGTTCCCTTCTCTACAGATTTGATCAAAAATCCCTGAGCCCAATAAAAGACATTATTTCTTTGAAATTCAAAACCATGCAGAGTGATGGGATTCTACTCCACAGGGAAGGGCCAAATGGAGATCACATCACACTGCAATTAAGAAGAGCAAGACTCTTTTTACTTATTAATTCAGGTGAAGCTAAACTGCCTTCCACTTCCACCCTGGTCAATCTCACCCTGGGCAGCCTGCTAGATGATCAGCATTGGCATTCAGTGCTCATCCAGCGTTTGGGCAAACAAGTCAACTTCACAGTGGACGAACACAGGCATCATTTCCATGCACGGGGAGAATTCAATCTCATGAATCTTGATTATGAGATCAGCTTTGGAGGGATTCCAGCACCTGGAAAATCAGTGTCATTCCCACATAGAAATTTTCATGGATGTTTAGAAAATCTCTATTATAATGGAGTGGATATCATTGATTTGGCCAAGCAGCAAAAACCACAGATCATTGCTATGGGAAATGTGTCATTTTCTTGTTCACAACCACAATCTATGCCCGTGACTTTTCTGAGCTCCAGGAGTTATTTAGCACTGCCAGACTTCTCTGGAGAGGAGGAGGTTTCTGCCACTTTTCAATTTCGAACTTGGAATAAGGCAGGGCTTCTGCTGTTCAGTGAACTTCAGCTGATTTCAGGGGGTATCCTCCTCTTTCTGAGTGATGGAAAACTTAAGTCGAATCTCTACCAGCCAGGAAAATTACCCAGTGACATCACAGCAGGTGTCGAATTAAATGATGGGCAGTGGCATTCTGTCTCTTTATCTGCTAAAAAGAATCACTTGAGTGTGGCGGTGGACGGCCAGATGGCTTCTGCTGCTCCTCTGCTGGGGCCTGAGCAGATTTATTCGGGTGGCACCTATTATTTTGGAGGTTGTCCTGACAAAAGCTTTGGATCCAAATGTAAAAGTCCACTTGGTGGATTTCAGGGATGTATGAGGCTCATTTCTATCAGCGGCAAAGTGGTAGATCTGATTTCAGTTCAGCAGGGGTCCCTTGGGAACTTCAGTGACCTTCAGATAGACTCATGTGGCATCTCAGACAG |
| hsa_circ_0106854 | TGATTACAGTGGCGAGATCTTGAACAACTGCTGCGTCATGGAGTACCACCAAGCCACAGGCACCCTTAGTGCCCACTTCAGGAATATGTCCCTGAAACGAATTAAGAGGTCAGACCGTCGTGGGGCAGAGTCGGTGACAGAAGAAAAATTTACAATCCTGTTTGAATCCCAGTTCAGTGTTGGTGGAAATGAGCTGGTTTTTCAAGTCAAGACCCTGTCCCTGCCAGTGGTGGTGATCGTTCATGGCAGCCAGGACAACAATGCGACGGCCACTGTTCTCTGGGACAATGCTTTTGCAGAGCCTGGCAGGGTGCCATTTGCCGTGCCTGACAAAGTGCTGTGGCCACAGCTGTGTGAGGCGCTCAACATGAAATTCAAGGCCGAAGTGCAGAGCAACCGGGGCCTGACCAAGGAGAACCTCGTGTTCCTGGCGCAGAAACTGTTCAACAACAGCAGCAGCCACCTGGAGGACTACAGTGGCCTGTCTGTGTCCTGGTCCCAGTTCAACAGGGAGAATTTACCAGGACGGAATTACACTTTCTGGCAATGGTTTGACGGTGTGATGGAAGTGTTAAAAAAACATCTCAAGCCTCATTGGAATGATGGGGCCATTTTGGGGTTTGTAAACAAGCAACAGGCCCATGACCTACTCATTAACAAGCCAGATGGGACCTTCCTCCTGAGATTCAGTGACTCAGAAATTGGCGGCATCACCATTGCTTGGAAGTTTGATTCTC |
| hsa_circ_0107273 | GCATCAGTTGGATGATTCATCATGGTAATTATGGCATTATCATATTCTTCATACTTGTCATACAAAAACACCAGTTCTGCCCAAAGATGAGCTTGTTCTGCAGCTCTTAGCACCTGCATAAGAAAGACTTAATTGCTTTAGATATGAGTCTAGCTAACAGCCTTAAAAAGAATAAGATATTTTGAAGCATTAGTTTTAACTACACAGTTGCTTCACTGTGTTACAATGACTGGTTACCTTGGGAATATTCACTCTAGACCAGAACAGCTCCAG |
| hsa_circ_0107299 | GTTTGTTTAATGCTTTTGATGAAAATCGTGACAATCACATAGATTTTAAGGAGATATCCTGTGGGTTATCAGCCTGTTGCAGGGGACCCCTGGCTGAAAGACAAAAATTTTGCTTCAAGGTATTTGATGTTGACCGTGATGGAGTTCTCTCCAGGGTTGAACTGAGAGACATGGTGGTTGCACTTTTAGAAGTCTGGAAGGACAACCGCACTGATGATATTCCT |
| hsa_circ_0107852 | CTCTGCTCTACAAGCCCATTGACCGGGTCACTCGGAGCACCCTAGTCCTACACGACCTGCTGAAGCACACACCTGTGGACCACCCCGACTACCCGCTGCTGCAGGATGCCCTCCGCATCTCCCAGAACTTCCTGTCCAGCATCAACGAGGACATCGACCCCCGCCGGACTGCAGTGACAACGCCCAAGGGGGAG |
| hsa_circ_0108704 | TGATCCGTATGTGAAACTTTCATTGTACGTAGCGGATGAGAATAGAGAACTTGCTTTGGTCCAGACAAAAACAATTAAAAAGACACTGAACCCAAAATGGAATGAAGAATTTTATTTCAGGGTAAACCCATCTAATCACAGACTCCTATTTGAAGTATTTGACGAAAATAGACTG |
| hsa_circ_0108824 | ATTTTCAGACGATGCTGGTTGGTTTTCAAGAAGGCTTCTAGCAAAGGACCCAGAAGGTTAGAAAAATTTCCAGATGAAAAGGCAGCTTATTTCAGAAACTTTCATAAGGTAACTGAACTGCACAATATCAAAAATATAACCAGACTGCCCCGAGAGACAAAGAAGCATGCGGTGGCAATCATCTTTCACGATGAAACATCGAAGACATTTGCCTGTGAGTCAG |
| hsa_circ_0109291 | GAGACGTTGACATTTAGGGATGTGGCCATAGAATTCTCTCTGGAGGAGTGGGAATGCCTGAACCCTGCTCAGCAGAATTTATATATGAATGTGATGTTAGAGAACTACAAAAACCTGGTCTTCTTGGCAGGTATTGCTGTCTCTAAGCAAGACCCGATCACCAGTCTAGAGCAAGAAAAAGAGCCCTGGAATATGAAGATATGTGAGATGGTGGATGAATCCCCAG |
| hsa_circ_0109324 | GGACTGTTGACATTTAGGGATGTGGCCATAGAATTCTCTCCGGAGGAGTGGCAATGTCTGGACACTGCACAGCAGAATTTATATAGGAATGTGATGTTAGAGAACTACAGAAACCTGGCCTTCCTGGGTATTGCTCTCTCTAAGCCAGACCTGATTACTTATCTGGAGCAAGGAAAAGAGCCCTGGAATATGAAGCAACATGAGATGGTGGATGAACCCACAG |
| hsa_circ_0109326 | GGACTGTTGACATTTAGGGATGTGGCCATAGAATTCTCTCTGGAAGAATGGCAATGCCTGGACACTGCACAGCGGAATTTATATAAAAATGTGATTTTAGAGAACTACAGAAACCTGGTCTTCCTGGGTATTGCTGTCTCTAAGCAAGACCTGATCACCTGTCTGGAGCAAGAAAAAGAGCCTTTGACTGTGAAGAGACATGAGATGGTGAATGAACCCCCAG |
| hsa_circ_0109331 | GGACTGTTGACATTTAGGGATGTGGCCATAGAATTCTCTCTGGAGGAGTGGCAACACCTGGACATTGCACAGCAGAATTTATATAGAAATGTGATGTTAGAGAACTACAGAAACCTGGCCTTCCTGGGTATTGCTGTCTCTAAGCCAGACCTGATCACCTGTCTGGAACAAGGGAAAGAGCCCTGGAATATGAAGCGACATGAGATGGTGGATGAACCCCCAG |
| hsa_circ_0109590 | ATTGTCCTGCCGCCCCATCTGGAGCGCATTCGGGAGAAGCTGGCGGAGAACATCCACGAGCTCTGGGCGCTAACCCGCATCGAGCAGGGCTGGACCTACGGCCCGGTTCGGGATGACAACAAGAGGCTGCACCCGTGTCTTGTGGACTTCCACAGCCTTCCAGAGCCTGAGAGGAACTACAACCTGCAGATGTCTGGGGAGACGCTCAA |
| hsa_circ_0109617 | TGCTGCAACCTGAGCGTGAGCGAGAGTCCCCGAGACCCTCTAGGGTTCAAGGTGTCTGATCTGACCATTCCCAAGCACAGACACCTGCTCCAGGCCAAGAACCAAGAAGAGAAGAGGCTGTGGATTCACTGTCTCCAGCGCCTCTTCTTTGAGAACCACCCTGCCTCCATCCCTGCCAAGGCAAAGCAAGTTCTCCTTGAAAACAGCCTGCATTGTGCCCCCAAAAGTAAGCCTGTCCTAGAGCCCCTGACACCCCCACTTGGGTCTCCTCGACCTCGAGATGCTAGAAGTTTTACCCCTGGGCGAAGGAACACAGCTCCATCTCCTGGGCCCTCTGTGATTCGCCGAGGCCGCAGGCAGTCTGAGCCGGTGAAGGACCCTTATGTCATGTTCCCACAGAACGCTAAGCCTGGATTCAAGCACGCTGGCAGCGAAGGGGAACTCTACCCTCCAGAATCTCAGCCACCAGTTTCAGGCTCTGCACCCCCTGAGGACCTGGAGGATGCTGGACCCCCAACACTGGACCCCTCTGGGACCTCAATCACTGAAGAAATCCTGGAACTGCTGAATCAGCGAGGCCTTCGAGATCCAGGG |
| hsa_circ_0109690 | CCCACTGCTCCTTGGGGACCTGTGTGCAGGCTTTTTCTCCCAGAGAAATGTAGACCCTGTTTCCCTCTGAGGCTCTGAAGCCCAAGAGTTCCAGCTCCCTGGGGCGCTCAGGTCTCCCTCCCCTATACTGGAGTTCAGGTCCCAGTCATGGACACCTCCCCACCAATTAGGAAACAGGACTTCTACCAGCCTCCCTGGAGGACTAAACATCCAGTAACTCCAGTTTCTACTCCCTAGGAGACAAAGACATAGTCCCTGCTAGAGTCTGGGCCACCAACTCCCTTCAGGACAGAAGGGTCTAGATGTCATTCACCATGGTTCCCAAAG |
| hsa_circ_0110097 | GAATGTGGGAAATGCTTCACTGTTTCTTCACACCTAGTTGAACATGTAAGAATTCATACTGGAGAGAAACCTTATCAATGTAAGGAATGTGGAAGAGCCTTCGCTGGGCGCTCAGGCCTTACTAAACATGTACGAATACACACTGGAGAGAAGCCCTATGAATGTAACGAATGTGGGAAAGCCTACAATAGGTTTTATCTACTAACTGAACATTTTAAAACTCACACAGAGGAGAAGCCCTTTGAATGTAAG |
| hsa_circ_0110791 | ATGTCTTTGGTGGACTTGGGAAAGAGGTTGCTAGAAGCAGCAAGAAAAGGCCAAGATGATGAAGTGAGAACGTTGATGGCAAATGGCGCCCCATTCACCACAGACTGGCTTGGAACATCACCCCTCCACCTTGCAGCTCAATATGGTCATTATTCCACAGCAGAAGTACTCCTTCGAGCAGGTGTTAGCAGGGATGCCCGGACTAAAGTAGACAGGACCCCCTTGCACATGGCTGCAGCCGATGGACATGCGCACATCGTGGAACTGCTTGTTCGGAATGGTGCAGATGTGAATGCCAAGGACATGCTGAAGATGACAGCTTTGCATTGGGCCACAGAGCGCCACCATCGAGATGTCGTAGAGTTACTTATCAAATATGGAGCTGATGTCCATGCTTTCAGCAAATTTGATAAATCAGCCTTTGACATAGCTCTGGAGAAAAACAATGCTGAGATTTTGGTCATCCTCCAGGAAGCAATGCAGAATCAGGTGAATGTTAATCCAGAGAGAGCCAACCCTGTGACTGACCCTGTGAGTATGGCTGCTCCATTCATCTTCACGTCGGGTGAGGTTGTTAACCTCGCAAGCCTTATTTCTTCAACCAACACCAAAACAACCTCAGGTGACCCCCATGCCTCAACAGTACAGTTTTCAAATTCTACCACCTCAGTGCTGGCTACCCTTGCAGCTCTTGCTGAGGCATCAGTCCCCCTCTCCAACTCACACAGAGCCACAGCCAATACAGAGGAAATTATAGAAGGAAATTCCGTTGACTCATCAATCCAGCAAGTAATGGGGAGTGGAGGCCAGAGGGTCATCACCATAGTGACTGATGGAGTCCCTCTGGGTAATATCCAAACTTCAATCCCTACTGGAGGCATTGGCCAGCCATTTATTGTAACTGTGCAAGATGGACAGCAAGTTCTAACTGTACCTGCTGGTAAGGTTGCAGAGGAGACTGTAATTAAAGAGGAAGAAGAAGAGAAGTTGCCACTAACAAAGAAACCAAGGATAGGAGAGAAGACAAACAGTGTGGAGGAAAGCAAGGAAGGCAATGAAAGAGAGCTACTACAGCAACAACTCCAGGAGGCCAATCGAAGAGCCCAGGAATACCGACACCAGCTCCTAAAGAAAGAGCAGGAAGCAGAACAGTACCGTCTTAAGCTGGAGGCCATAGCCCGACAGCAGCCCAATGGAGTTGATTTCACCATGGTTGAAGAGGTGGCTGAG |
| hsa_circ_0111274 | GAACCCACAAGACTCCCAGAAGGTGAAGTTAAGAGCTCCCAGACTCATAAGGTTATTAGAACAGCAAACTGGCACCCCAAAGAACTTTACGGAGACTTGCAACCTATCAACAAGTTGGATGAGGGATTAAAAGCCTTCAACAACCAACAACCCCAAGCATCAAACTGAAGGAAACATTCTAACCTTCACAGACAGACTGGAGGCTGGATGGGGACCTGGCTGAAGACATCTGGAGAATGAAAGTTAAGTACCAGCTTGCATTTTTGTGCCCCTAGATTATTTTTGCATTTTAAAATAAGAAGCATCAAATTGCGTGTCTCTGTGTAAAAGTTCTAGCAATTTGTTTTAAGGTGAACTTATTTTGGCTTAGGGACTACAAAAAGAGAAGGTAATTCCTAGGGAAGGAAGAAGAGAAAGAAATGAAAATTAGAGAATAAGATTATTTTGAATGACTTCAGGTAGCGAGGAGTGTGTGTTTGTGAGTGTGTATTTGAGAGACTTGGCTCATGCCTGTGGGTCTTCTCTTCTAGTATCAGTGAGGGGAGGGATTACTGAAGAAGAAGGGGGGAAAAAAAAAGAAAGAAATCTGAGCTTTCTGGGAGGAAATTCAAAGGAACCAAGAGAAATTAACTTCGTTCTGCAAGGACTAAAGTACAGCAAGAGGAGAGAGGTCAAGCGAGAAGCGTGCGGGAAGCACATGCCCTGGGGAGGCATAGAAGCCACACTGGCAGAGCGGCCAGCACAGGTAGCCAGCAGAGGCATTCTTGGGGCTATTTGAAAAAGTTTGGTCTGTGAACAAAACAGTTTCCCTGGTGACTGCAAATCCATTGCTAGCTGCCTCTTTCTCGTCTGCCCATCACTCTGGTGTGGTACCCAGAAGTTGACTTCTGGTTCTGTAGAAAGAGCTAGGGGAGGTATGATGTGCTTAAAGATCCTAAGAATAAGCCTGGCGATTTTGGCTGGGTGGGCACTCTGTTCTGCCAACTCTGAGCTGGGCTGGACACGCAAGAAATCCTTGGTTGAGAGGGAACACCTGAATCAGGTGCTGTTGGAAGGAGAACGTTGTTGGCTGGGGGCCAAGGTTCGAAGACCCAGAGCTTCTCCACAGCATCACCTCTTTGGAGTCTACCCCAGCAGGGCTGGGAACTACCTAAGGCCCTACCCCGTGGGGGAGCAAGAAATCCATCATACAGGACGCAGCAAACCAGACACTGAAGGAAATGCTGTGAGCCTTGTTCCCCCAGACCTGACTGAAAATCCAGCAGGACTGAGGGGTGCAGTTGAAGAGCCGGCTGCCCCATGGGTAGGGGATAGTCCTATTGGGCAATCTGAGCTGCTGGGAGATGATGACGCTTATCTCGGCAATCAAAGATCCAAGGAGTCTCTAGGTGAGGCCGGGATTCAGAAAGGCTCAGCCATGGCTGCCACTACTACCACCGCCATTTTCACAACCCTGAACGAACCCAAACCAGAGACCCAAAGGAGGGGCTGGGCCAAGTCCAGGCAGCGTCGCCAAGTGTGGAAGAGGCGGGCGGAAGATGGGCAGGGAGACTCCGGTATCTCTTCACATTTCCAACCTTGGCCCAAGCATTCCCTTAAACACAGGGTCAAAAAGAGTCCACCGGAGGAAAGCAACCAAAATGGTGGAGAGGGCTCCTACCGAGAAGCAGAGACCTTTAACTCCCAAGTAGGACTGCCCATCTTATACTTCTCTGGGAGGCGGGAGCGGCTGCTGCTGCGTCCAGAAGTGCTGGCTGAGATTCCCCGGGAGGCGTTCACAGTGGAAGCCTGGGTTAAACCGGAGGGAGGACAGAACAACCCAGCCATCATCGCAG |
| hsa_circ_0111829 | GAGAGCTGATAGTATTCATTCCACTCTTTTCATAATTGGTCAGAACAGTTAGAAATTGCTTTTAACTATAGTCATGCATTGCTTAACAATGGGTATACATTCTGAGAGATACATCTTTAGGCGATTTCATCATCGTGCCAACATCATAAAGTGTACTTACACAAACCTACGTGGTACAGCCTACTACATACCTAGGCTTTATGGGATAGTCTGTTGCTTCTAGGCTACAAACCTGTACAGCATGTTACTGTACTAAATACTGTAGGCAGTTATAACACAACG |
| hsa_circ_0112437 | AGTACAGAGAGATGGATGAAAGCTTGGCCAACCTCTCAGAAGATGAGTATTATTCAGAAGAAGAGAGAAATGCCAAAGCAGAGAAGGAAAAGAAGCTTCCCCCACCACCCCCTCAAGCCCCACCTGAGGAAGAAAATGAAAGTGAGCCTGAAGAACCATCGGGGCAAGCAGGAGGACTTCAAGACGACAGTTCTGGAGGGTATGGAGACGGCCAAGCATCAGGTGTGGAGGGCGCAGCTTTCCAGAGCCGACTTCCTCATGACCGGATGACTTCTCAAGAAGCAGCCTGTTTTCCAGATATTATCAGTGGACCACAACAGACCCAGAAGGTTTTTCTTTTCATTAGAAACCGCACAGTAA |
| hsa_circ_0113381 | GATGACATCTGAACTGGAGAGCAGCCTAACGTCTATGGACTGGTTACCACAGCTCACCATGAGAGCAGCCATCCAAAAATCTGATGCTACACAAAATGCACATGGAACAGGAATTTCTAAGAAGAATGCACTCCTTGACCCAAATACAACTCTGGACCAGGAAGAAGTCCAACAGCACAAAGATGGGAAACCTCCATACAGTTATGCCAGCCTCATTACATTTGCAATTAATAGCTCACCCAAAAAGAAAATGACTTTAAGTGAAATTTATCAGTGGATTTGTGATAACTTCCCATATTATAGAGAGGCTGGCAGTGGTTGGAAG |
| hsa_circ_0115374 | CTGCTGATGAAGAAGCATTTGAAGATAATTCTGAGGAGTACATAAGGAGAGATTTGGAAGGATCTGATATTGATACTAGACGCAGGGCTGCTTGTGATCTGGTACGAGGATTATGCAAGTTTTTTGAGGGACCTGTGACAGGAATCTTCTCTGGTTATGTTAATTCCATGCTGCAGGAATACGCAAAAAATCCATCTGTCAACTGGAAACACAAAGATGCAGCCATCTACCTAGTGACATCTTTGGCATCAAAAGCCCAAACACAGAAGCATGGAATTACACAAGCAAATGAACTTGTAAACCTAACTGAGTTCTTTGTGAATCACATCCTCCCTGATTTAAAATCAGCTAATGTGAATGAATTTCCTGTCCTTAAAGCTGACGGTATCAAATATATTATGATTTTTAGAAATCAAGTGCCAAAAGAACATCTTTTAGTCTCGATTCCTCTCTTGATTAATCATCTTCAAGCTGAAAGTATTGTTGTTCATACTTACGCAGCTCATGCTCTTGAACGGCTCTTTACTATGCGAGGGCCTAACAATGCCACTCTCTTTACAGCTGCAGAAATCGCACCGTTTGTTGAGATTCTGCTAACAAACCTTTTCAAAGCTCTCACACTTCCTGGCTCTTCAGAAAATGAATATATTATGAAAGCTATCATGAGAAGTTTTTCTCTCCTACAAGAAGCCATAATCCCCTACATCCCTACTCTCATCACTCAGCTTACACAGAAGCTATTAGCTGTTAGTAAGAACCCAAGCAAACCTCACTTTAATCACTACATGTTTGAAGCAATATGTTTATCCATAAGAATAACTTGCAAAGCTAACCCTGCTGCTGTTGTAAATTTTGAGGAGGCTTTGTTTTTGGTGTTTACTGAAATCTTACAAAATGATGTGCAAGAATTTATTCCATACGTCTTTCAAGTGATGTCTTTGCTTCTGGAAACACACAAAAATGACATCCCGTCTTCCTATATGGCCTTATTTCCTCATCTCCTTCAGCCAGTGCTTTGGGAAAGAACAGGAAATATTCCTGCTCTAGTGAGGCTTCTTCAAGCATTCTTAGAACGCGGTTCAAACACAATAGCAAGTGCTGCAGCTGACAAAATTCCTGGGTTACTAGGTGTCTTTCAGAAGCTGATTGCATCCAAAGCAAATGACCACCAAGGTTTTTATCTTCTAAACAGTATAATAGAGCACATGCCTCCTGAATCAGTTGACCAATATAGGAAACAAATCTTCATTCTGCTATTCCAGAGACTTCAGAATTCCAAAACAACCAAGTTTATCAAGAGTTTTTTAGTCTTTATTAATTTGTATTGCATAAAATATGGGGCACTAGCACTACAAGAAATATTTGATGGTATACAACCAAA |
| hsa_circ_0116250 | ATCTGTGACTTTGGCCTGGCCCGTGTTGCAGATCCAGACCATGATCACACAGGGTTCCTGACAGAATATGTGGCCACACGTTGGTACAGGGCTCCAGAAATTATGTTGAATTCCAAGGGCTACACCAAGTCCATTGATATTTGGTCTGTAGGCTGCATTCTGGCAGAAATGCTTTCTAACAGGCCCATCTTTCCAGGGAAGCATTATCTTGACCAGCTGAACCACATTTTGGGTATTCTTGGATCCCCATCACAAGAAGACCTGAATTGTATAATAAATTTAAAAGCTAGGAACTATTTGCTTTCTCTTCCACACAAAAATAAGGTGCCATGGAACAGGCTGTTCCCAAATGCTGACTCCAAAG |
| hsa_circ_0116773 | ACATCAATGAGTGCAGCAGCAGCCCCTGTAGCCAGGAGTGTGCCAACGTCTACGGCTCCTACCAGTGTTACTGCCGGCGAGGCTACCAGCTCAGCGATGTGGATGGAGTCACCTGTGAAG |
| hsa_circ_0116782 | TGACAACATCAGTCCAGAGTGTAGTAATTTAAAAATTGTCTTCACGAAGCCTGGCAGTAATTGTTCTGTGCTTTTCTGCTGTCCATCATCCAAATACCAGCTGACGGAAACATCTACTGCATTTCAATAAGGGTAACAATGGTAGCAGAGTCCACATCTTGAGGTCTTATTCCATTTAATAAATCTCTCACTGCTCCTTCTGCTATTCTGAATTCACGATTCTCTTTAACAAATAATTCTGTCAAGCTCTTCAATCTTTCTGTGACAAATGACTAGAATTCTGGAGCCTGCACCTTCATTTTAGGCAGCTATTGCTTCAGAAGTCACAGTCTGTTCCACCAGCCTTTGCCAAGGATGCAGGTGCCACAGCATCTCAAGAGAAGTCACCAATCACACAGCTCCATGCTGAGGCCCTCTTGCCTGCTACTGAGGAGCTCTTGCCACCCCCGATTCCTGCCTTTCCCCAGTGATGCAGGACAGGTAAATGAGCTGGATGGTATCCCGTTGATCCTGGACAACTGCAACATCAGTGACAGTAACCCCT |
| hsa_circ_0118071 | GTTGACTTTGGCAGAATATCATGAACAGGAAGAAATTTTCAAACTTAGACTAGGACATCTCAAAAAGGTATGTGAAACATATGCTGCATTTTTAGTTCAATTTGCTACCAGAATTTGTGGGAAGTTGTGTTATAAGTCACTTATTTTAAAAGCCAAAATTTAAGAGAGTAAGATTTCAAATTAAAAATTTCAGATTAAAAAGAGATGCATTAAAGTTACTTTATTTTATTTATTAGTATTTTTATTTTTTGAAACAGTCTCATCACTCAGGCTGGAGTGCAGCGGCACAATCTAAGCTCAGTGCAACCTCCACCTCCCTGGTTCAAGCAATTTTCGTGCTTCAGCCTCCTGAGTAGCTGGGATTACAGGCATGCGCCACCACATCTGGCTAATTTTTGTATTTTTAGTAGAGTGAGGGTTTCGCCATGTTCACCAGGCTGGTCTCAAACTCCTGGCTTCGTGTGATCCACCCACCTTGGCCTCCCAAGGTGCTGGGATTACAGGCATGAGCTGTCGCACCCGGCCAAAATTACATTTTTATGTTGAGTATATTAAAACAACATTCCTTTATCTTGTGTCAACTTAAAAATCTTGAGATGGTGACACACCATATCCCTTTAGTGGAGGAATGATTCTTGGACAGACTTCCAAGGTCTTTAGATTATTCTATTTAAGTACTACCTTTTGGTAAAAAATACTTCATTTTAAAGATGAAATCTAAAATTCCATATTGTTTATGTTCATTTCATAATAAAATAGCAATTGTAAATAAATAGCAAACTTATTTCTTTGGCTTATCTTTTAATTTGTTGCATGTGTGATGTTTCGATTAGAAGATGTACGTAGGAAAAAGCACTTAGTGATAGTATAAAGGGATAAAACTGAGAAAAAATGTTGCTATGTGTTATTGTCGTAAACTTTTAAATAATGTGTATCTTTCTACTTGTTTGTAAACTCTGAAATTGTAATCAGTTTATTGTTTTTACAGGTCTTAATTTTAGTTCAAATGATTTCAGAGAAGTAGTAATAATAGAGTAGGTTTTTTTCCCTCAAAATTAAGTTACTGACAGACATAAAGTTCTTTTTCTTTGACCCAATTTTTTGTGCTTTCTGCTTGTCAGGTTCTTTCAATTTTTTAAATAGGCTGAATTATAGGTAAAATTGAAGGAGTGAACTAATTGGAGATAGTATCTGTGAAGTTGTCTCCATGGGATAGGGTGAGGTAGGATGGAGAAACCCATTCTGGTAGCAGCTACTATTACACTGCATACTAATTTGTTTTGGAAATGTTATTAATATGCTGGGGATTCCTACCATTTATAGGGAATAGAAAATTATTATATATAATTTGCATTAGTATCCTTTAATATGCTATATCTGTCTATGCCAACTGCCCAGAAAGCTTCAACGTAAACTACTTGAACAGTGAATTAAAAAGTGATTTATTTTGGTTTTGTTATTTAAGTAGTTCTATTCTGTTCTATTGTAATTATTGACTACTTTTCCTGCGCTTTGAGACTTAGTTGACAACTTAAGTCTATTCTCTATAATAATAGATGGAGCTATATAACTATCATAATAGTTCTATAATAGTGATAGAACCATATAAAGGAACTATGGAAGCGAAAAGGGAATTTTTAGTACAGGATGAGTCGTAAGTATCTGTGTGCCCACTTAAACTGTTTTGATAATGAGTTCGTAAGGGCCTATGCTTTATGTCTCATATAAAGATTTACCAGTTATTTTCTAACCAGCCTGTGGTGATAAAGGAATACTTATTTACTTAGTTATAGTGATTCCAAGGTGTACTTGAGAGTTTTACTGAAGGGTTATTATCTTCACGTGTAATACCAAATTTCAGATTACTCTTTCTAAATGATGACATTTTACATATGCACCTTATTTTATGAAAATCTGTTATAAAATCTGAAATTGTAGAGCAGTTAAATTAGGAATTGGCTATTTGTATTACAAAAAGTACACTTTGAAAAGGCTTCAAAGGAGGGTTTGTTTTTGTTTTTGGTTGGTTGGTTTTAAAGCTTTCCTGGTACTTTGAAAATACTGGTTTCTGTGAAAAACACTTTTATAAAGTAAGGTATACTTAGTGTCCAAATATTATATAGACATTTGTAAAATGTTGTAGAACACTAAACCTTCTAACCTTCGTATTTTCCTCTTTCCCAGTGTTTGACTTCTATATTTTTCGTATTTACTTACTCTTTCCAATAATTATCCCTTCCATAATCTTTTTCCTGCTTTTTTTCCTAGCTTTTATCAAATTTACTGAGCAGTCTTAGATTTCTAACTTTATCTTCTTTTTAACTAATTTACAACATAACTTTTCTTTTCTTTGACCTTCATCATGGAATATTTTGGGGAAATGGATTAACAGTAATGCTGAAACATACTCTCATGTCATGAATACCCATGTTTTCTTCTGAGAGACTATTTTGGGTGAGAAGAATGAGTGTATTATAAAGAAAGTGGCCAGTAGTACATACAGGGAACAAAATTAAGCAAGTAATGTCACCTTAGAGCATAAATAGCAGTTGCCTGCTTAGAACCGAGTATTAATTTTTGAGTTATACAGTTAAAAAATAATAAGCAATAAGTTACATGTATAAAAATAAGTTATTTGGCATTCTGAAGCACAGTTGGAAAATAGGTCATTCTGTTTTCTAAAAAAGATTAAACAGATTTTTAAAACACAGGACAGGCTTGAGTCAAAAGAAAAGATGTTATCACTTCTGTCTTATCAGCTCCCTATGCTCCTGGGTTTATTAAGCAGACATGAACCCCGACTTGTAAATATACAGCTTCTACCATAGGCTGGATCTGAATATTTGACGCCCCTTGTATCCTGCACAGGAAATATAACCAGTCAGTTTACGGTTTAGATTTGGAGCCTGGCATACTCTTGCATTAGCATGGGTGACTCTCTTGCCCTTTCTCCCTTCAATAAATTCTTAACATTAAGCAGAACTAATTATTAGACTAACTTTCTTAGCACTGAAGGACCAAAGTTCATTGAGGAAGCCCATGGCATATGGACATAGGAGAATAGAAAGACCTGTTTACTTTTTCTTGAAGTGGATAATTTATGACATGCTTTGTGTTTCATAATGATAATCACTATAATTGAAAAGCTAAACTAAAATATGAAAAAGTATATTTACCTTTGAATAAATCATGAAGTTTAGTTCATATTTTATGCTGTTCTTCAGGGTTAGAAAAATAATAAATCCAAAGATAGCCTTACTCTATTATCCAATGTGTGATAGAGTAAGGAATGAGAGTCAGGCAGAAACCTCCAAAAGTAAATACATGGCTAAAATAAATAGGTGTTTCTAGTTACTACCAAAAAGTAAATGTAAACTTACTTATGGACTTATTAGTTTTCTCTTCTGAGATTAGGAAAATCACAGCAGAGCGTCACTTTTTTTCCTCTCAACTGTATCAAGAATTCTTTTATCTAATATTTATTGTTATAGTTGTTCCCAAGTTTAAGCAGTCGTTGCTCACCCATTTATTAAGTGACTTATTGCCATACTCTGCCTGACACAGGCATAATTCAAATTCCCATTTTAACGGAATTACATATTACTTTTTATAGACTTTGAAGCTTTCACCATTTTTGAGTGAACATTAATTACACCTTTTTGAAAGCCCAGATCTATATATACTTCCTCTGGAGTAATACCACCAGTAAGTGACAAAGCCAAAACTTGAACCATTCCCACTCCTATTTCAATGACTGTACCCATGTAGTTAATTCCTAGAATTATTTTGTTGTAGATTAATAGTAAGTTGATTCTTTGTATTCAGAAAAATTAATTCACTTGTGGCTTTCCTTGCATCTCTATTAAGTACTGGACTGTTTGCTTTTCCTTAAGTTTTCTTTTGGTGTGTGGCGGGATGAACGTGCTATTTCATTTTCATTACAGATAAAGGAGAACAGTTTTAAGTTAATTATTTTTCATACACATTCTTTTTCATTCTCTTTCACATACTTTCTTCCTTGGGCATTATGTTAGTGACTGTTGAATCAACTACAATATTTCTTTATTATTAACTAATATATTGATTTTCACTCGATTTATTTTTAAAGGTCAACAAAGACCTTTTTAAAAAGCACATTTTTAAATAAAAACTCAAATAATTTTAAAATATATCTTAAGGAATTCTTAGAATAGAACTATATAGTAAAATGTATGGAGACTATACAAATTATTCCTGAAACTATTTCAAAAGGAACAAGTAACTTTTTTGGTTATCATTAATTATATACAAACTTCCCCAGTTATTACTTAATAAAAGCTTGTAGTTGTAAAGAGATTAATTTCCTATCTTTAAAAAGTTGTTAATTTGAGGCAAAGCTTCTAAATGATCCTATTTTATCTACCTGTGCCCATGTGGAATCGTGGGTTCAAGGAAACCAACCAAAAACCAGTTTGCTCTTATAGTTGATCAGGTTTTGATACCTGTCTCTGATAAGTTCTAATTATGGGAAAACAATTTATCTTTGTGATTCAGAAGCTCTGCTAGGTTGATTATATAGTCAGATCTTTATTCTGGAGTTGAAGAAGATGGTGTGGATTTGCTTTCAAGGATTGTCACTAAATTAACACATAAAACTAAAAATACAATCCCTGCAAAATCAACTTCCTTGAAATGTCTATATTTTTAAAAAGATACAAGTTATTAGTCATAATACCCCAAACTGATGAGTAAATGAGAATTTTGAAAGTTATTAGGTCCAGTAACTATGTCTTGAGACTGCTGTTACTCAAATTTGTGATATGAACAGCATGTGGGTATGTTACTCTTTATTTTCTTGTCTTCAGATTTTTTTGTCTTCTTGATTTTAAATATTTGATTAGTTGGAGAGAATGTGTTTAGAAAGTGTGACATCTCAGTATGGAGTATTAAATAGTGTAGTAGGGATTGGCTTTAATGTGTGTGTTTTGGTGTGTGTGTGTGTGTGTGTGTGTATGTGTGAGTGCATTTGACTTATGAGCCCTGAATATAAGTGCCAGAAGTTTTATTTGAAAAACATAACCAATTTGTAATTGAAACAGGATTAGTCAAGAACCATCAGCACCAACACAAATGTATCTTTGCAGACCGAAGGAATCAGCTAAACAATTTACAGTCATCTCAATCTCTACTAAAACAAAAATCACATCCAACATGCCACCTGACACCATTTCTTTCTCTCTCTCTCCTTTGCTCCTTGCGATGTGGCATTCATCTCTCCTTGTGCCTCCGTTCTGAAGAGATAACAGTATAGCAACAACTCTGCCACTGAAATCCTGTTCTCTGACCGATATTGGCACCTGCAAAGAGAAACAACCAGTAACAGGCAGCAGCAGCATCAGTATTAATCTTCCATGATGAAATCTTTACAGGTCAAGAACAAGTACACAGCTCTTTTCTCACTCCTTCACAGTGGACCATGCAACTAGTTGAAATGGAAGACAATGGATTGTCTACAAGCCTTTTGAACAGTGGAGAATGCAGGGCGTTGGCTTTAG |
| hsa_circ_0118328 | GGTTGTGGAGTTGCTCAGTGCTTGAAGATTGTCTGCCAAGTTGGGAGATTAGACAGAGGAAAGAGTGCAATCTTGTACGTAAAGTCATTACTGTGGACTGAGACTTTTATGAATAAAGAAAATCAGAATCATTCCTATTCTCTGAAGTCGTCTGCTTCATTTAATGTCATAGAGTTTCCTTATAAGAATCTTCCAATTGAGGATATCACCAACTCCACATTG |
| hsa_circ_0119150 | ATGTGGAGAGATTATCCCCAAAAGAGAACAGTTTAATGACCTCTCTATTGACCTTCCTCGTAGGAAAAAACCACTCCCTCCTCGTTCAATTCAAGATTCTCTTGATCTTTTCTTTAGGGCCGAAGAACTGGAGTATTCTTGTGAGAAGTGTGGTGGGAAGTGTGCTCTTGTCAGGCACAAATTTAACAGGCTTCCTAGGGTCCTCATTCTCCATTTGAAACGATATAGCTTCAATGTGGCTCTCTCGCTTAACAATAAGATTGGGCAGCAAGTCATCATTCCAAGATACCTGACCCTGTCATCTCATTGCACTGAAAATACAAAACCACCTTTTACCCTTGGTTGGAGTGCACATATGGCAATTTCTAGACCATTGAAAGCCTCTCAAATGGTGAATTCCTGCATCACCAGCCCTTCTACACCTTCAAAGAAATTCACCTTCAAATCCAAGAGCTCCTTGGCTTTATGCCTTGATTCAGACAGTGAGGATGAGCTAAAACGTTCTGTGGCCCTCAGCCAGAGACTTTGTGAAATGTTAGGCAACGAACAGCAGCAGGAAGACCTGGAAAAAGATTCAAAATTATGCCCAATAGAGCCTGACAAGTCTGAATTGGAAAACTCAGGATTTGACAGAATGAGCGAAGAAGAGCTTCTAGCAGCTGTCTTGGAGATAAGTAAGAGAGATGCTTCACCATCTCTGAGTCATGAAGATGATGATAAGCCAACTAGCAGCCCAGATACCGGATTTGCAGAAGATGATATTCAAGAAATGCCAGAAAATCCAGACACTATGGAAACTGAGAAGCCCAAAACAATCACAGAGCTGG |
| hsa_circ_0119249 | GTGGAGAAGGCAGACAAAAAAAGAAACAAGTAAATATAGAGTAAGTGCAAATGTAACAAATTCTGTGAAAAAAAAATACTAAACTAGAGAATTGAGGGTCAGGGGTATGGGGAGAGTGCAATTTAAAATAAGCTCAGGGAAAGACTCACTGAGGATGTGACATTTGAGTAAAGACCTGAAGTAATAAGTGAGGAGAGAAGCCATGCAGATCTCAGGAAAAACAGATTTCTAAGGAAGAAGAAATGTGGAGAGAAAAGGTCGTAATGGGGAGCATTCACAGCACATAGAAGGTCCCTGAAGGACCTCACTGTGGCTCGAACAGGGCAGAGCAGGTGAGGCGGAGGTTAGAAGATGAGGCCAGAGGCAATGGAGTCCAATCTTGTGAGGACATGGAGGTTACTGTCAGGAAGCTGGCTTAGCTCAGGGTGAAATTGGGATGAGAGAGGTCTCAGTAAGGGCAGGGCAGGGAGACCAGTCAGGAGGCTGTAAAAGCAATAGAGATGAGGGAAGATGTTGGCTGGGACCAGAGCAGCAGTCCTGGAGGAGGTGAGATGTGCTGGAATCGTAGTTACACTCTGAAGGTAGAATTGACAGAATTTGGTGACAGATTGGATGGGAG |
| hsa_circ_0119637 | ACTGATGGTGCATCTGCAATGTTAATCATGGCGGAGGAAAAGGCTCTGGCCATGGGTTATAAGCCGAAGGCATATTTGAGGGATTTTATGTATGTGTCTCAGGATCCAAAAGATCAACTATTACTTGG |
| hsa_circ_0119776 | ACAAGACTACTTTTGCTTAAAAATCTGTTATGTAAACAAGGAAGTCCATTGACCCAAAAAGAACTAGCACAACTTGCTAGAATGACTGATGGATACTCAGGAAGTGACCTAACAGCTTTGGCAAAAGATGCAGCACTGGGTCCTATCCGAG |
| hsa_circ_0121735 | CCTTTGGAGAACATCAGCTTGCCAGCAGCTCTGAGGTGCAAAATGGAAGTCCCATGTCTCAGACTGAGACTGTGTCTAGGTCAGTCGCACCCATGAGAGGTGGAGAGATCACTGCACACTGGCTCTTGACCAACAGCACAACATCTGCAGATGTGACAGGAAGCTCTGCTTCATATCCTGAAGGTGTGAATGCTTCAGTGTTGACCCAGTTCTCAGACTCTACTGTACAGTCTGGAGGAAGTCACACAGCATTGGGAGATAGGAGTTATTCAGAGTCTTCATCTACATCTTCCTCGGAAAGCTTGAATTCATCAGCACCACGTGGAGAACGTTCGA |
| hsa_circ_0125543 | ATTACTATTTTGGATGCAAAACGGAGCATGAACATTGGGATATTTCTTAAGCAATTTAAGAAGTCTCCTCGGTCCATTGTAGAAGATATTCATCAAGGAAAAAGTGAGCATTATGGATCAGAGACCTTGCGAGAATTTCTTAAGTTTTTGCCAGAGTCAGAAGAG |
| hsa_circ_0126686 | GATGGGACTGAGGAAGACCCACAGGCAGAACTGGCCATCATTCATGGTCAGATGGCTTATATTCTGCAGCTTCAGGGTCGAACAGAGGAGGCTTTGCAACTTTACAATCAAATAATAAAACTAAAACCAACAGATGTGGGATTACTAGCTGTAATTGCAAATAACATCATTACCATTAACAAGGACCAAAATGTCTTTGACTCCAAGAAGAAAGTGAAATTAACCAATGCGGAAGGAGTAGAGTTTAAGCTTTCCAAGAAACAACTACAAGCTATAGAATTTAACAAAGCTTTACTTGCTATGTACACAAACCAG |
| hsa_circ_0129046 | GACTGACTGCATTGCACAGATGATGGATATTTACGTATGTTTGAAACGACCATCCTGGATGGTGGACAATAAAAGAATGAGGACTGCTTCAAATTTCCAGTGGCTGTTATCAACATTTATTCTTCTATATCTAATGAATCAAGTAAATAGCCAGAAAAAGGGGGCTCCTCATGATTTGAAGTGTGTAACTAACAATTTGCAAGTGTGGAACTGTTCTTGGAAAGCACCCTCTGGAACAGGCCGTGGTACTGATTATGAAGTTTGCATTGAAAACAGGTCCCGTTCTTGTTATCAGTTGGAGAAAACCAGTATTAAAATTCCAGCTCTTTCACATGGTGATTATGAAATAACAATAAATTCTCTACATGATTTTGGAAGTTCTACAAGTAAATTCACACTAAATGAACAAAACGTTTCCTTAATTCCAGATACTCCAGAGATCTTGAATTTGTCTGCTGATTTCTCAACCTCTACATTATACCTAAAGTGGAACGACAGGGGTTCAGTTTTTCCACACCGCTCAAATGTTATCTGGGAAATTAAAGTTCTACGTAAAGAGAGTATGGAGCTCGTAAAATTAGTGACCCACAACACAACTCTGAATGGCAAAGATACACTTCATCACTGGAGTTGGGCCTCAGATATGCCCTTGGAATGTGCCATTCATTTTGTGGAAATTAGATGCTACATTGACAATCTTCATTTTTCTGGTCTCGAAGAGTGGAGTGACTGGAGCCCTGTGAAGAACATTTCTTGGATACCTGATTCTCAGACTAAGGTTTTTCCTCAAGATAAAGTGATACTTGTAGGCTCAGACATAACATTTTGTTGTGTGAGTCAAGAAAAAGTGTTATCAGCACTGATTGGCCATACAAACTGCCCCTTGATCCATCTTGATGGGGAAAATGTTGCAATCAAGATTCGTAATATTTCTGTTTCTGCAAGTAGTGGAACAAATGTAGTTTTTACAACCGAAGATAACATATTTGGAACCGTTATTTTTGCTGGAT |
| hsa_circ_0129881 | GATTGGATGAAAGGTCTGCAGGCATTTTGCAATTTACGGAAAAGTAGTCCAGGGACATCCAATAAACGCCTTCGTCAGGTCAGCAGCCTTGTTTTACATATTGAAGAAGCCCATAAACTCCCAGTAAAACATTTTACTAATCCATATTGTAACATCTACCTGAATAGTGTCCAAGTAGCAAAAACTCATGCAAGGGAAGGGCAAAACCCAGTATGGTCAGAAGAGTTTGTCTTTGA |
| hsa_circ_0131420 | CCTGAGACCTCAGATCATTGTTCCCTCCCAGAGGATCTAAGAGTATTGGAAGTTTCCAACCATTGGTGGTACTCTATGCTCATCCTACCTCCTTTGCTGAAAGACAGTGTGGCAGCGCCCCTGCTGTCTGCCTACTACCCTGACTGTGTTGGCATGAGCCCCTCCTGCACCAGCACAAACCGCGCCGCTGCCACTGGCAATGCCAGCCCTGGGAAGCTGGAGCACTCCAAGGCTGCCCTCTCCGTGCACG |
| hsa_circ_0132013 | GGCTGGTGAATTACCAGATCTCCATCAAGTGCAGTAACCAGTTCAAGTTGGAAGTGTGTCTTTTGAATGCAGAAAACAAAGTCGTGGACAACCAGGCTGGGACCCAGGGCCAGCTGAAGGTGCTGGGTGCCAACCTCTGGTGGCCGTACCTGATGCACGAACACCCCGCCTACCTGTACTCGTGGGAGGATGGTGATTGCTCACACCAAAGCCTTGGACCCCTCCCAGCCTGTGACCTTTGTGACCAACGTCACCTATGCAGCAGACAAGGGGGCTCTGTATGTGGATGTGATCCGTGTGAACAGCTACTACTCTTGGTATCGCAACTACGGGCACCTGGAGTTGATTCAGCTGCAGCTGGCCACCCAGTTTGAGAATTGGTGTAAGACATCACAATCCCATTATTCAGAGCGCGTATGGAGTGGAAACGCTTGTAGGGTTTCACCAGGATCCACCTCTGATGTTCAGTGAAGAGTACCAGAAAAGTCTGCTAGAGCAGTACCATCTGGGTCTGGATCAAAAACGCAGAAAATACGTGGTAGGAAAGCTCATCTGGAATTTTGCCGATTTCATGACTAACCAGT |
| hsa_circ_0132430 | GGTTCTGAATGAGTTTATTATGAAAAACCCTAGTTTGGAAAATAAAAAAGACCAAAGAGACCTTCAGGATGTAACTCACAAAATAGTGGATGCAATTGGTGCAATTGCTGGTTCTTCTCTGGAACAGACAACATGGCTGCGACGAAATCTTGAAGTTAAGCCTTCTCCCAAAATAATGGTAGATGGAACCAATTTGGAATCTGATGTTGAAGATATGTTATCACCTGCAATGGAAACCGCAAACATAACTCCTTCTGTATATAGTGTCCATGCATTGACATTACTCTCTGAGGTTTTGGCTCATCTTTTGGATATGGTTTTCTATAGTGATGAAAAGGAGCGGGTTATTCCTTTACTTGTAAATATTATGCATTATGTTGTGCCCTACCTCAGAAATCACAGTGCACATAATGCCCCTAGTTATCGAGCTTGTGTCCAGCTGCTCAGCAGTCTTAGTGGGTATCAGTACACACGGAGAGCTTGGAAAAAAGAAGCTTTTGACCTCTTTATGGATCCCAGTTTCTTTCAGATGGATGCCTCTTGTGTTAATCATTGGAGAGCAATTATGGACAATCTGATGACACATGATAAAACAACATTTAGAGATTTGATGA |
| hsa_circ_0133954 | ACTCTATCAAGAAGAATATCTAATCCGTACCTTGAACATACGCCTTCCCAGATTTATGGAGAGAATTCTTCTTGTGCAGGAAGAGCATTGAGGAATATTATTATCGTTCAAGCAGCTGACCTGATAAAGGACAGAGTGAACCTCAAGGGGTTTTACAGGAGGAGCTGCGTTGGGTCAGAGCTGGTAGACTGGCTTCTAGAACACTGTCCTTTCGTCCAGTGCAGATCTATGGCCATAGGAGTCTGGCAACTCCTACTGGACATGGGAATTATGTTATCAGTGGACCAGCATCTATACTTTCAAGATACTTATGTTTTCTACCAGTTTTCCTCTGATGAATGTAGCTACTTGTACTGTGAATTTGAAAGAGAAGAAGAATGGCAAAATGGTGTCAAGCTTTTACTGCAACTTGTGCCTCTCATTCCTGCCAGAGGTGGCATCTGTGAACT |
| hsa_circ_0134731 | ATAAACCTCTACTTGAAAATGGAGAAAAAACCTAACAAAAAGGAGGAACTGACACTAGTGAATAATGTTTTAAAACTGGCTACTAAACTGCTAAAGGAGTTGGACAGTCCTTTTAGATTATATGGGCTTACAATGAATCCGCTGCTTTATAACATCACCCAGGTTGTTATCCTGTCAGCTGTTTCTGGTGTTATCAGTGACTTGCTTGGATTTAATTTAAAG |
| hsa_circ_0136538 | TGGGCTACCTTGAGAATGGAGCGAGGAGCCAAGGAGAAGAACCACCAGCTTTACAAGCCCTACACCAACGGAATCATTGCAAAGGATCCCACTTCACTAGAAGAAGAGATCAAAGAGATTCGTCGAAGTGGTAGTAGTAAGGCTCTGGACAACACTCCAGAGTTCGAGCTCTCTGACATTTTCTACTTTTGCCGGAAAGGAATGGAGACCATTATGGATGATGAGGTGACAAAGAGATTCTCAGCAGAAGAACTGGAGTCCTGGAACCTGCTGAGCAGAACCAATTATAACTTCCAGTACATCAGCCTTCGGCTCACGGTCCTGTGGGGGTTAGGAGTGCTGATTCGGTACTGCTTTCTGCTGCCGCTCAGGATAGCACTGGCTTTCACAGGGATTAGCCTTCTGGTGGTGGGCACAACTGTGGTGGGATACTTGCCAAATGGGAGGTTTAAGGAGTTCATGAGTAAACATGTTCACTTAATGTGTTACCGGATCTGCGTGCGAGCGCTGACAGCCATCATCACCTACCATGACAGGGAAAACAGACCAAGAAATGGTGGCATCTGTGTGGCCAATCATACCTCACCGATCGATGTGATCATCTTGGCCAGCGATGGCTATTATGCCATG |
| hsa_circ_0136839 | ATCAGGATCCTCATGACCAGCCAAAGAGAAGAAGAATTAGGAAGCATAAATCAAAGAAAAAATTTAAAAATCCCAATAATGTTCTTATAGAACAAGCAGAATTAGAGAAACAGCAGAGTCTGTTACAGGAGAAATCTCAGCGACAGCACACAGATGGCACCACAATAAGCAAAAATAAAAAAAGGAAACTGAAAAAGAAACAGCAAATTAAAAGGAAGAAAGCAGCCGGCTTGGCAGCAAAGGCTGCTGGTGTCAGTTTCATGTACCAGCCCGAGGACAGCAGCAATGAAGGGGAAGGCGTGGGAGAGGCTTGTGAGGAGGATGGTGTGGACACCAGCGAGGAAGACCCGACACTGGCCGGGGAGGAAGACGTTAAAGATACCAGGGAGGAAGATGGTGCGGACGCTAGCGAGGAAGACCTGACACGGGCCAGGCAGGAAGAGGGTGCGGACGCCAGTGAGGAAGATCCGACACCGGCCGGGGAGGAAGACGTTAAAGACGCCAGGGAGGAGGACGGTGTGGACACCATTGAGGAAGACCTGACACGGGCCGGGGAGGAAGACGGTAAAGACACCAGGGAGGAGGACGGTGCGGACGCCAGCGAGGAAGACCCGACATGGGCTGGGGAGGAAGAGGGTGCAGACTCCGGGGAGGAGGACGGTGCAGACGCCAGCGAGGAAGATGATACAATTACCAATGAAAAGGCACACAGTATTCTAAATTTTTTGAAGTCAACACAGGAAATGTATTTTTATGACGGTGTCTCCAGAGATGCAGCTTCAGCTGCCCTCGCAGATGCCGCTGAGGAGCTGCTGGACCGCCTTGCGTCACACAGCATGCTGCCCTCAGACGTGTCCATCCTGTACCACATGAAAACGCTGCTGCTCCTGCAAGATACTGAGAGATTGAAGCATGCTCTGGAAATGTTCCCAGAACATTGCACGATGCCTCCTG |
| hsa_circ_0137651 | ACTCATCATATTCAGAGCCCCCAGATGTTCAGCAGCAGTTGAACCACTATCAGTCAGCTGCCCTGGCAAGGAACAACAGCCGTGTTAGCCCTGTGCCTCTTTCTGGGGCTGCTGCTGGCACTGAGCAGAAAACTGAAGCCGTGCTTCACTGCGAATTCTGTGAATTCTCCTCCGGCTACATCCAGAGCATCAGGCGTCATTACCGGGACAAGCATGGTGGGAAGAAGCTTTTCAAGTGCAAAGACTGCTCCTTTTACACAGGCTTTAAATCTGCTTTTACTATGCACGTGGAAGCTGGGCACTCAGCAGTTCCCGAGGAGGGCCCCAAAGATCTTCGCTGTCCTCTCTGCCTCTATCACACCAAATACAAGCGCAACATGATTGACCACATCGTGCTGCACCGAG |
| hsa_circ_0137730 | ACATAGATGAGTGTGAAGTTTCTGGCCTGTGCAGGCATGGAGGGCGATGCGTGAACACTCATGGGAGCTTTGAATGCTACTGTATGGATGGATACTTGCCAAGGAATGGACCTGAACCTTTCCACCCGACCACCGATGCCACATCATGCACAGAAATAGACTGTGGTACCCCTCCTGAGGTTCCAGATGGCTATATCATAGGAAATTATACGTCTAGTCTGGGCAGCCAGGTTCGTTATGCTTGCAGAGAAGGATTCTTCAGTGTTCCAGAAGATACAGTTTCAAGCTGCACAGGCCTGGGCACATGGGAGTCCCCAAAATTACATTGCCAAG |
| hsa_circ_0138708 | CCCCGTGTGGTTGGTGCGCGGACACGCACTGCCTGCGTAACTAGAGGGAGCTGACGGATGACGCCCCCGCGCCACGCCGCTCAGCGGGATACGCTTCTTGGCGGACTTTGGAGTGGGAAGCGGGGAATGTCTACGTGCGTATGCACGTGGCACTCTCTGCCCGAG |
| hsa_circ_0138870 | GCACTTGAGAGAAAAGACAACGATAGCAGTTACATCTAGAGGCTATTATGGATTGGAGGATGAGAAGGGAACTGCATGTACCTCAACAAGGCGTCGGTCAACACCGCGAAGTTTGGCAGGCTTGACAAGTGGAGTTTTTGAATCTATAATGGTTCAAGTTTTGAGACAGGAAGAACAGCTGAGAGCAAAAGAAGAAAAAAGGCTTCGGGAGCAGGAAAGAAAAGAAGCAGAAGAAGCTAGTCAAAAGGAAATAGAAGAATGGGAAAGAAAACTTCTAGCTCAAGCAGCTCCAACTTGTATGGAGACCATGTGGGAAATTCCAGCTATTGGGCATTTCCTTTGTTTAGCTCAGCAAATTCTAAATTTGCCAGAAATAGTCTTTTACGAATTGGAACGTTGTCTTCTGATGCCTCAGTGTAATGCTTTTCTATCGAAAATAATGACTTCTCTATTAAGTCCTCCCCATCGCAGACCTACCTTACATCGAAGACCTACTTTGCCTTATAGGACCTGGGAAGCAGCGCTGAGGCAGAAAGTACAACAGTGGTACACTGCTGTAGGGCAAACTGAAAATCCTGATAACTGTGCTGAAAAACTGGGGTTGTGTCCTCAGTTTTTTAAAGTTCTTGGAGAAGTTAATCCATTGGAAGAAAAACCTTTTCACGAACTACCTTTTTACCAAAAAGTGTGGCTACTTAAGGGTCTTTGTGACTTTGTGTATGAAACACAAAAGGAAGTTCAAGATGCTGTACTTGGACAGCCTATCCATGAATGCAGGGAAGTTATTCTTGGTTATGATTATTTGGAGAATGCTTATGTACATTTTCCACAGTTCTGTGGTGCAGATGTACGGATTTATAAACAGAGACCCTTTCAGGCCCCAGAATTTCCAATTCCACCCATTAAAATACAAAGAGTACCTCGGATTAAATTGGAGAAATTGAAGTGTGACTATGTTAGTACAAGTAATGGAGAACATAGATGTAGCAGAGATAGCCTGCCCTCTTCCTTCAAGAAAGAGCAGGAAAATAATTTTGATCCAGCTTGCTGTCCTGCTAAAATGATCTTGGATAATCATGACATCTCTGTTGAAATGGGAGTAAAATCCAACTATGAAATTAGAATTCGCAGGCCTTGTGAAATTAAAAAAACTGATTGTTGTAAAGAAAATTTAGAGAAACCAAGGAGTCCAGGGGAAGTTACTGGCTTTGGAGAGCCTCTTAGTCCAGGTGAAATAAGGTTTATAGAAAATCAGGAAAAATACGGTGAAGCTTCCAGAATAAAGATTGAACCCAGTCCATTAAAAGAAAATACTCTAAAATCTTGCCAAATACATGTAAATGGAAGTCACAGTGATCATCCAGAAATTAACTGCCACAAAGTTGTAAGGGATATTCTATTAGAGCAGTCACTACAGAGCCACAAGAAACTCAAACTAACTAAAATGAGGGCAAAAAAGAAGAAAAAGAAAAAAAAGAAATTGAAAGATGTTTTGAATGAAAACTTACAGAGAAAGCGTGAAGGTCTTCATTCTCTTGCATTCAAGTCTTACAAACCTGAGATCCAGAATAAGTTATTGATCATCAAAAAAAAAGCAAAACACAAGAAGCACAAATCTGGAAAAAAATCCGTCTCTAAAAAAGCAATCACAAAGAAGAGGAAAACTGTCATAAAGTCACCTACTGTACCAGAGTTTCAG |
| hsa_circ_0139204 | TGGATGACGTAGTAGATGAAAGTGATGACAACGATGATATTGATGTAGAAGCTGAAAACGAAACTGAGAATGAAGATGACCTAGATCAAAATTTTAAGAATGATGATATTGAAACAGATATTAACAAACTAAAACCCCAGCAAGAACCGGGACGAACAATAGAAGATCTAAAAATGTATGAACACCTTTTCCCTGAGCTTGTTGATGATTTTCAG |
| hsa_circ_0139869 | AGTTCAAGGCAGCTTGCCTCGAGATTTCATGAACAGTTTATCGTAAGAGAAGATCTGATGGGTCTAGCTATTGGTACTCATGGTGCTAATATTCAGCAAGCTAGAAAAGTACCTGGGGTCACTGCTATTGATCTAGATGAAGATACCTGCACATTTCATATTTATGGAGAGGATCAGGATGCAGTGAAAAAAGCTAGAAGCTTTCTCGAATTTGCTGAAGATGTAATACAAGTTCCAAGGAACTTAGTAG |
| hsa_circ_02158 | GGTCCCCAAGGCTTCCAAGGTCCCCCTGGTGAGCCTGGCGAGCCTGGAGCTTCAGGTCCCATGGGTCCCCGAGGTCCCCCAGGTCCCCCTGGAAAGAATGGAGATGATGGGGAAGCTGGAAAACCTGGTCGTCCTGGTGAGCGTGGGCCTCCTGGGCCTCAGGGTGCTCGAGGATTGCCCGGAACAGCTGGCCTCCCTGGAATGAAGGGACACAGAGGTTTCAGTGGTTTGGATGGTGCCAAGGGAGATGCTGGTCCTGCTGGTCCTAAGGGTGAGCCTGGCAGCCCTGGTGAAAATGGAGCTCCTGGTCAGATGGGCCCCCGTGGCCTGCCTGGTGAGAGAGGTCGCCCTGGAGCCCCTGGCCCTGCTGGTGCTCGTGGAAATGATGGTGCTACTGGTGCTGCCGGGCCCCCTGGTCCCACCGGCCCCGCTGGTCCTCCTGGCTTCCCTGGTGCTGTTGGTGCTAAGGGTGAAGCTGGTCCCCAAGGGCCCCGAGGCTCTGAAGGTCCCCAGGGTGTGCGTGGTGAGCCTGGCCCCCCTGGCCCTGCTGGTGCTGCTGGCCCTGCTGGAAACCCTGGTGCTGATGGACAGCCTGGTGCTAAAGGTGCCAATGGTGCTCCTGGTATTGCTGGTGCTCCTGGCTTCCCTGGTGCCCGAGGCCCCTCTGGACCCCAGGGCCCCGGCGGCCCTCCTGGTCCCAAGGGTAACAGCGGTGAACCTGGTGCTCCTGGCAGCAAAGGAGACACTGGTGCTAAGGGAGAGCCTGGCCCTGTTGGTGTTCAAGGACCCCCTGGCCCTGCTGGAGAGGAAGGAAAGCGAGGAGCTCGAGGTGAACCCGGACCCACTGGCCTGCCCGGACCCCCTGGCGAGCGTGGTGGACCTGGTAGCCGTGGTTTCCCTGGCGCAGATGGTGTTGCTGGTCCCAAGGGTCCCGCTGGTGAACGTGGTTCTCCTGGCCCTGCTGGCCCCAAAGGATCTCCTGGTGAAGCTGGTCGTCCCGGTGAAGCTGGTCTGCCTGGTGCCAAGGGTCTGACTGGAAGCCCTGGCAGCCCTGGTCCTGATGGCAAAACTGGCCCCCCTGGTCCCGCCGGTCAAGATGGTCGCCCCGGACCCCCAGGCCCACCTGGTGCCCGTGGTCAGGCTGGTGTGATGGGATTCCCTGGACCTAAAGGTGCTGCTGGAGAGCCCGGCAAGGCTGGAGAGCGAGGTGTTCCCGGACCCCCTGGCGCTGTCGGTCCTGCTGGCAAAGATGGAGAGGCTGGAGCTCAGGGACCCCCTGGCCCTGCTGGTCCCGCTGGCGAGAGAGGTGAACAAGGCCCTGCTGGCTCCCCCGGATTCCAGGGTCTCCCTGGTCCTGCTGGTCCTCCAGGTGAAGCAGGCAAACCTGGTGAACAGGGTGTTCCTGGAGACCTTGGCGCCCCTGGCCCCTCTGGAGCAAGAGGCGAGAGAGGTTTCCCTGGCGAGCGTGGTGTGCAAGGTCCCCCTGGTCCTGCTGGTCCCCGAGGGGCCAACGGTGCTCCCGGCAACGATGGTGCTAAGGGTGATGCTGGTGCCCCTGGAGCTCCCGGTAGCCAGGGCGCCCCTGGCCTTCAGGGAATGCCTGGTGAACGTGGTGCAGCTGGTCTTCCAGGGCCTAAGGGTGACAGAGGTGATGCTGGTCCCAAAGGTGCTGATGGCTCTCCTGGCAAAGATGGCGTCCGTGGTCTGACTGGCCCCATTGGTCCTCCTGGCCCTGCTGGTGCCCCTGGTGACAAGGGTGAAAGTGGTCCCAGCGGCCCTGCTGGTCCCACTGGAGCTCGTGGTGCCCCCGGAGACCGTGGTGAGCCTGGTCCCCCCGGCCCTGCTGGCTTTGCTGGCCCCCCTGGTGCTGACGGCCAACCTGGTGCTAAAGGCGAACCTGGTGATGCTGGTGCTAAAGGCGATGCTGGTCCCCCTGGCCCTGCCGGACCCGCTGGACCCCCTGGCCCCATTGGTAATGTTGGTGCTCCTGGAGCCAAAGGTGCTCGCGGCAGCGCTGGTCCCCCTGGTGCTACTGGTTTCCCTGGTGCTGCTGGCCGAGTCGGTCCTCCTGGCCCCTCTGGAAATGCTGGACCCCCTGGCCCTCCTGGTCCTGCTGGCAAAGAAGGCGGCAAAGGTCCCCGTGGTGAGACTGGCCCTGCTGGACGTCCTGGTGAAGTTGGTCCCCCTGGTCCCCCTGGCCCTGCTGGCGAGAAAGGATCCCCTGGTGCTGATGGTCCTGCTGGTGCTCCTGGTACTCCCGGGCCTCAAGGTATTGCTGGACAGCGTGGTGTGGTCGGCCTGCCTGGTCAGAGAGGAGAGAGAGGCTTCCCTGGTCTTCCTGGCCCCTCTGGTGAACCTGGCAAACAAGGTCCCTCTGGAGCAAGTGGTGAACGTGGTCCCCCTGGTCCCATGGGCCCCCCTGGATTGGCTGGACCCCCTGGTGAATCTGGACGTGAGGGGGCTCCTGGTGCCGAAGGTTCCCCTGGACGAGACGGTTCTCCTGGCGCCAAGGGTGACCGTGGTGAGACCGGCCCCGCTGGACCCCCTGGTGCTCCTGGTGCTCCTGGTGCCCCTGGCCCCGTTGGCCCTGCTGGCAAGAGTGGTGATCGTGGTGAGACTGGTCCTGCTGGTCCCACCGGTCCTGTCGGCCCTGTTGGCGCCCGTGGCCCCGCCGGACCCCAAGGCCCCCGTGGTGACAAGGGTGAGACAGGCGAACAGGGCGACAGAGGCATAAAGGGTCACCGTGGCTTCTCTGGCCTCCAGGGTCCCCCTGGCCCTCCTGGCTCTCCTGGTGAACAAGGTCCCTCTGGAGCCTCTGGTCCTGCTGGTCCCCGA |
| hsa_circ_04440 | TTCCAAGATGAGATGGGATTCTCCAACATGGAAGATGATGGCCCAGAAGAGGAGGAGTGTGTAGCTGAGCCTCGTCCTAACTTTAACACCCCTCAAGCTCTACGGTTTGAGGAACCACTGGCCAACCTGTTAAATGAACAACATCGGACAGTGAAGGAGCTATTTGAACAGCTGAAGATGAAGAAATCTTCAGCCAAACAGCTGCAGGAAGTAGAGAAGGTTAAACCCCAGAGTGAGAAAGTTCATCAGACTCTGATTCTGGACCCAGCACAGAGGAAGAGACTCCAGCAGCAGATGCAGCAGCACGTTCAGCTCTTGACCCAAATCCACCTTCTTGCCACCTGCAACCCCAACCTCAATCCGGAGGCCACTACCACCAGGATATTTCTTAAAGAGCTGGGAACCTTTGCTCAAAGCTCCATCGCCCTTCACCATCAGTACAACCCCAAGTTTCAGACCCTGTTCCAACCCTGTAACTTGATGGGAGCTATGCAGCTGATTGAAGACTTCAGCACACATGTCAGCATTGACTGCAGCCCTCATAAAACTGTCAAGAAGACTG |
| hsa_circ_06511 | ACATTGATGAATGTTTAGTAAACAGACTGCTTTGTGATAACGGATTGTGCCGAAACACGCCAGGAAGTTACAGCTGTACGTGCCCACCAGGGTATGTGTTCAGGACTGAGACAGAGACCTGTGAAGATATAAATGAATGTGAAAGCAACCCATGTGTCAATGGGGCCTGCAGAAACAACCTTGGATCTTTCAATTGTGAATGTTCGCCCGGCAGCAAACTCAGCTCCACAGGATTGATCTGTATTGACAGCCTGAAGGGGACCTGTTGGCTCAACATCCAGGACAGCCGCTGTGAGGTGAATATTAATGGAGCCACTCTGAAATCTGAATGCTGTGCCACCCTCGGAGCCGCCTGGGGGAGCCCCTGTGAGCGGTGTGAACTAGATACAGCTTGCCCAAGAGGGCTTGCCAGGATTAAAGGTGTTACGTGTGAAGATGTTAATGAGTGTGAGGTGTTCCCTGGCGTTTGTCCAAATGGACGCTGTGTCAACAGTAAGGGATCTTTTCATTGCGAGTGCCCTGAAGGCCTTACGTTGGATGGGACTGGCCGTGTATGTTTGGATATTCGCATGGAGCAGTGTTACTTGAAGTGGGATGAAGATGAATGCATCCACCCCGTTCCTGGAAAGTTCCGCATGGATGCCTGCTGCTGTGCTGTCGGGGCGGCTTGGGGCACCGAGTGTGAGGAGTGCCCCAAACCTGGCACCAAGGAATACGAGACGCTGTGCCCCCGCGGGGCTGGCTTTGCTAACCGAGGGGATGTTCTTACTGGGCGGCCATTTTACAAAGACATCAATGAATGCAAAGCATTTCCTGGGATGTGCACTTATGGGAAGTGCAGAAATACAATCGGAAGCTTCAAATGCCGTTGCAATAGTGGCTTTGCTCTAGACATGGAGGAAAGAAACTGCACGGACATCGACGAGTGCAGGATTTCTCCTGACCTCTGTGGCAGTGGAATCTGCGTCAATACACCGGGCAGCTTTGAGTGCGAGTGCTTCGAAGGCTATGAAAGTGGCTTCATGATGATGAAGAACTGCATGGACATTGACGAATGTGAACGTAACCCTCTCCTTTGTAGGGGTGGCACCTGTGTGAACACTGAGGGCAGCTTTCAGTGTGACTGCCCACTGGGACACGAGCTGTCACCATCCCGTGAGGACTGTGTGGATATTAATGAATGCTCCCTGAGTGACAATCTCTGCAGAAATGGAAAATGTGTGAACATGATTGGAACCTATCAGTGCTCTTGCAATCCTGGATATCAGGCTACGCCAGACCGCCAGGGCTGTACAGATATTGATGAATGTATGATAATGAACGGAGGCTGTGACACCCAGTGCACAAATTCAGAGGGAAGCTACGAATGCAGCTGCAGTGAGGGTTATGCCCTGATGCCAGATGGGAGATCGTGTGCAG |
| hsa_circ_06790 | CAAGCTCCTGAAGATACAATGCCTGGAAGGTGGAATCTGGGAGCAAGGCAGCTGCATTCCTGTGGTGTGTGAGCCACCCCCTCCTGTGTTTGAAGGCATGTATGAATGTACCAATGGCTTCAGCCTGGACAGCCAGTGTGTGCTCAACTGTAACCAGGAACGTGAAAAGCTTCCCATCCTCTGCACTAAAGAGGGCCTGTGGACCCAGGAGTTTAAGTTGTGTGAGAATCTGCAAGGAGAATGCCCACCACCCCCCTCAGAGCTGAATTCTGTGGAGTACAAATGTGAACAAGGATATGGGATTGGTGCAGTGTGTTCCCCATTGTGTGTAATCCCCCCCAGTGACCCCGTGATGCTACCTGAGAATATCACTGCTGACACTCTGGAGCACTGGATGGAACCTGTCAAAGTCCAGAGCATTGTGTGCACTGGCCGGCGTCAATGGCACCCAGACCCCGTCTTAGTCCACTGCATCCAGTCATGTGAG |
| hsa_circ_07579 | CCTTCCAGTGCCCTGCCCACAGCCACTACGAGCTCTGCGGTGACTCCTGTCCTGGGAGCTGCCCGAGCCTGTCGGCACCCGAGGGCTGTGAGTCGGCCTGCCGTGAAGGCTGTGTCTGCGATGCTGGCTTCGTGCTCAGTGGTGACACGTGTGTACCTGTGGGCCAGTGTGGCTGCCTCCACGATGACCGCTACTACCCACTGGGCCAGACCTTCTACCCTGGCCCTGGGTGTGATTCCCTTTGCCGCTGCCGGGAGGGCGGTGAGGTGTCCTGTGAGCCCTCCAGCTGCGGCCCGCATGAGACCTGCCGGCCATCCGGTGGCAGCTTGGGCTGCGTGGCCGTGGGCTCTACCACCTGCCAGGCGTCGGGAGATCCCCACTACACCACCTTCGATGGCCGCCGCTTCGACTTCATGGGCACCTGCGTGTATGTGCTGGCTCAGACCTGCGGCACCCGGCCTGGCCTACATCGGTTTGCCGTCCTGCAGGAGAACGTGGCCTGGGGTAATGGGCGAGTCAGTGTGACCAGGGTGATCACGGTCCAGGTGGCAAACTTCACCCTGCGGCTGGAGCAGAGACAGTGGAAGGTCACGGTGAACGGTGTGGACATGAAGCTGCCCGTGGTGCTGGCCAACGGCCAGATCCGTGCCTCCCAGCATGGTTCAGATGTTGTGATTGAGACCGACTTCGGCCTGCGTGTGGCCTACGACCTTGTGTACTATGTGCGGGTCACCGTCCCTGGAAACTACTACCAGCTGATGTGTGGCCTGTGTGGGAACTACAACGGCGACCCCAAGGATGACTTCCAGAAGCCCAATGGCTCGCAGGCAGGCAACGCCAATGAGTTCGGCAACTCCTGGGAGGAGGTGGTGCCCGACTCTCCCTGCCTGCCGCCGCCCACCTGCCCGCCGGGGAGCGAGGGCTGTATCCCCAGCGAGGAGTGTCCTCCCGAGCTGGAGAAGAAGTATCAGAAGGAGGAGTTCTGTGGGCTCCTCTCCAGCCCCACAGGGCCACTGTCCTCTTGCCACAAGCTGGTGGATCCCCAGGGTCCCTTGAAAGATTGCATCTTTGATCTCTGCCTGGGTGGTGGGAACCTGAGCATTCTCTGCAGCAACATCCATGCCTACGTGAGTGCTTGCCAGGCAGCTGGAGGCCAGGTGGAGCCCTGGAGGAATGAAACTTTCTGTCCCATGGAATGCCCTCAGAACAGTCACTACGAGCTCTGTGCGGACACCTGCTCCCTGGGCTGCTCGGCTCTCAGTGCCCCTCTGCAGTGCCCAGATGGGTGTGCTGAGGGCTGCCAGTGTGACTCCGGCTTCCTCTACAACGGCCAAGCCTGCGTGCCCATCCAGCAATGTGGCTGCTACCACAATGGTGCCTACTATGAGCCGGAGCAGACAGTCCTCATTGACAACTGTCGGCAGCAGTGCACGTGCCATGTGGGTAAAGTCGTGGTGTGCCAGGAACACAGCTGCAAGCCGGGGCAGGTGTGCCAGCCCTCCGGAGGCATCCTGAGCTGCGTCAACAAAGACCCGTGCCACGGCGTGACATGCCGGCCACAGGAGACATGCAAGGAGCAGGGTGGCCAGGGCGTGTGCCTGCCCAACTATGAGGCCACGTGCTGGCTGTGGGGCGACCCACACTACCACTCCTTCGATGGCCGGAAGTTTGACTTCCAGGGCACCTGTAACTATGTGCTGGCAACAACTGGCTGCCCGGGGGTCAGCACCCAGGGCCTGACACCCTTCACCGTCACCACCAAGAACCAGAACCGGGGCAACCCTGCTGTGTCCTACGTGAGAGTCGTCACCGTGGCTGCCCTCGGCACCAACATCTCCATCCACAAGGACGAGATCGGCAAAGTCCGGGTGAACGGTGTGCTCACAGCCTTGCCTGTCTCTGTGGCCGACGGGCGGATTTCAGTGACCCAGGGTGCATCGAAGGCACTGCTGGTGGCTGACTTTGGACTGCAAGTCAGCTATGACTGGAACTGGCGGGTAGACGTGACGCTGCCCAGCAGCTATCATGGCGCAGTGTGCGGGCTCTGCGGTAACATGGACCGCAACCCCAACAATGACCAGGTCTTCCCTAATGGCACACTGGCTCCCTCCATACCCATCTGGGGCGGCAGCTGGCGAGCCCCAGGCTGGGACCCACTGTGTTGGGACGAATGTCGGGGGTCCTGCCCAACGTGCCCTGAGGACCGGTTGGAGCAGTACGAGGGCCCTGGCTTCTGCGGACCCCTGGCCCCCGGCACAGGGGGCCCTTTCACCACCTGCCATGCTCATGTGCCACCTGAGAGCTTCTTCAAGGGCTGTGTTCTGGACGTCTGCATGGGTGGTGGGGACCGTGACATTCTTTGCAAGGCTCTGGCTTCCTATGTGGCCGCCTGCCAGGCTGCTGGGGTTGTCATCGAAGACTGGCGGGCACAGGTTGGCTGTGAGATCACCTGCCCAGAAAACAGCCACTATGAGGTCTGTGGCCCACCCTGCCCGGCCAGCTGTCCGTCCCCTGCACCCCTTACGACGCCAGCCGTATGTGAGGGCCCCTGTGTGGAGGGCTGCCAGTGCGACGCGGGTTTCGTGTTAAGTGCTGACCGCTGTGTTCCCCTCAACAACGGCTGCGGCTGCTGGGCCAATGGCACCTACCACGAGGCGGGCAGTGAGTTTTGGGCTGATGGCACCTGCTCCCAGTGGTGTCGCTGCGGGCCTGGGGGTGGCTCGCTGGTCTGCACACCTGCCAGCTGTGGGCTGGGTGAAGTGTGTGGCCTCCTGCCATCCGGCCAGCACGGCTGCCAGCCCGTCAGCACAGCTGAGTGCCAGGCGTGGGGTGACCCCCATTACGTCACTCTGGATGGGCACCGATTCGATTTCCAAGGCACCTGCGAGTACCTGCTGAGTGCACCCTGCCACGGACCACCCTTGGGGGCTGAGAACTTCACTGTCACTGTAGCCAATGAGCACCGGGGCAGCCAGGCTGTCAGCTACACCCGCAGTGTCACCCTGCAAATCTACAACCACAGCCTGACACTGAGTGCCCGCTGGCCCCGGAAGCTACAGGTGGACGGCGTGTTCGTCACTCTGCCCTTCCAGCTGGACTCGCTCCTGCACGCACACCTGAGCGGCGCCGACGTGGTGGTGACCACAACCTCAGGGCTCTCGCTGGCTTTCGATGGGGACAGCTTCGTGCGCCTGCGCGTGCCGGCGGCGTACGCGGGCTCTCTCTGTGGCTTATGCGGGAACTACAACCAGGACCCCGCAGACGACCTGAAGGCGGTGGGCGGGAAGCCCGCCGGATGGCAGGTGGGCGGCGCCCAGGGCTGCGGGGAATGTGTGTCCAAGCCATGCCCGTCGCCGTGCACCCCAGAGCAGCAAGAGTCCTTCGGCGGCCCGGACGCCTGCGGCGTGATCTCCGCCACCGACGGCCCGCTGGCGCCCTGCCACGGCCTTGTGCCGCCCGCGCAGTACTTCCAGGGCTGCTTGCTGGACGCCTGCCAAGTTCAGGGCCATCCTGGAGGCCTCTGTCCTGCAGTGGCCACCTACGTGGCAGCCTGTCAGGCCGCTGGGGCCCAGCTCCGCGAGTGGAGGCGGCCGGACTTCTGTCCCTTCCAGTGCCCTGCCCACAGCCACTACGAGCTCTGCGGTGACTCCTGTCCTGGGAGCTGCCCGAGCCTGTCGGCACCCGAGGGCTGTGAGTCGGCCTGCCGTGAAGGCTGTGTCTGCGATGCTGGCTTCGTGCTCAGTGGTGACACGTGTGTACCTGTGGGCCAGTGTGGCTGCCTCCACGATGACCGCTACTACCCACTGGGCCAGACCTTCTACCCTGGCCCTGGGTGTGATTCCCTTTGCCGCTGCCGGGAGGGCGGTGAGGTGTCCTGTGAGCCCTCCAGCTGCGGCCCGCATGAGACCTGCCGGCCATCCGGTGGCAGCTTGGGCTGCGTGGCCGTGGGCTCTACCACCTGCCAGGCGTCGGGAGATCCCCACTACACCACCTTCGATGGCCACCGCTTCGACTTCATGGGCACCTGCGTGTATGTGCTGGCTCAGACCTGCGGCACCCGGCCTGGCCTGCATCGGTTTGCCGTCCTGCAGGAGAACGTGGCCTGGGGTAATGGGCGAGTCAGTGTGACCAGGGTGATCACGGTCCAGGTGGCAAACTTCACCCTGCGGCTGGAGCAGAGACAGTGGAAGGTCACGGTGAACGGTGTGGACATGAAGCTGCCCGTGGTGCTGGCCAACGGCCAGATCCGTGCCTCCCAGCATGGTTCAGATGTTGTGATTGAGACCGACTTCGGCCTGCGTGTGGCCTACGACCTTGTGTACTATGTGCGGGTCACCGTCCCTGGAAACTACTACCAGCTGATGTGTGGCCTGTGTGGGAACTACAACGGCGACCCCAAGGATGACTTCCAGAAGCCCAATGGCTCGCAGGCAGGCAACGCCAATGAGTTCGGCAACTCCTGGGAGGAGGTGGTGCCCGACTCTCCCTGCCTGCCGCCGCCCACCTGCCCGCCGGGGAGCGCGGGCTGTATCCCCAGCGACAAGTGTCCTCCCGAGCTGGAGAAGAAGTATCAGAAGGAGGAGTTCTGTGGGCTCCTCTCCAGCCCCACAGGGCCACTGTCCTCCTGCCACAAGCTGGTGGATCCCCAGGGTCCCTTGAAAGATTGCATCTTTGATCTCTGCCTGGGTGGTGGGAACCTGAGCATTCTCTGCAGCAACATCCATGCCTACGTGAGTGCTTGCCAGGCGGCTGGAGGCCACGTGGAGCCCTGGAGGAATGAAACTTTCTGTCCCATGGAATGCCCTCAGAACAGTCACTACGAGCTCTGTGCGGACACCTGCTCCCTGGGCTGCTCGGCTCTCAGTGCCCCTCTGCAGTGCCCAGATGGGTGTGCTGAGGGCTGCCAGTGTGACTCCGGCTTCCTCTACAACGGCCAAGCCTGCGTGCCCATCCAGCAATGTGGCTGCTACCACAATGGTGTCTACTATGAGCCGGAGCAGACAGTCCTCATTGACAACTGTCGGCAGCAGTGCACGTGCCATGTGGGTAAAGTCGTGGTGTGCCAGGAACACAGCTGCAAGCCGGGGCAGGTGTGCCAGCCCTCCGGAGGCATCCTGAGCTGCGTCACCAAAGACCCGTGCCACGGCGTGACATGCCGGCCACAGGAGACATGCAAGGAGCAGGGTGGCCAGGGCGTGTGCCTGCCCAACTATGAGGCCACGTGCTGGCTGTGGGGCGACCCACACTACCACTCCTTCGATGGCCGGAAGTTTGACTTCCAGGGCACCTGTAACTATGTGCTGGCAACAACTGGCTGCCCGGGGGTCAGCACCCAGGGCCTGACACCCTTCACCGTCACCACCAAGAACCAGAACCGGGGCAACCCTGCTGTGTCCTACGTGAGAGTCGTCACCGTGGCTGCCCTCGGCACCAACATCTCCATCCACAAGGACGAGATCGGCAAAGTCCGGGTGAACGGTGTGCTCACAGCCTTGCCTGTCTCCGTGGCCGACGGGCGGATTTCAGTGGCCCAGGGTGCATCGAAGGCACTGCTGGTGGCTGACTTTGGACTGCAAGTCAGCTATGACTGGAACTGGCGGGTAGACGTGACGCTCCCCAGCAGCTATCATGGCGCAGTGTGCGGGCTCTGCGGTAACATGGACCGCAACCCCAACAATGACCAGGTCTTCCCTAATGGCACACTGGCTCCCTCCATACCCATCTGGGGCGGCAGCTGGCGAGCCCCAGGCTGGGACCCACTGTGTTGGGACGAATGTCGGGGGTCCTGCCCAACGTGCCCTGAGGACCGGTTGGAGCAGTACGAGGGCCCTGGCTTCTGCGGACCCCTTTCATCTGGCACAGGGGGCCCCTTCACCACCTGCCATGCTCATGTGCCACCTGAGAGCTTCTTCAAGGGCTGTGTTCTGGACGTCTGCATGGGTGGTGGGGACCGTGACATTCTTTGCAAGGCTCTGGCTTCCTACGTGGCCGCCTGCCAGGCCGCTGGGGTTGTCATCGAAGACTGGCGGGCACAGGTTGGCTGTGAGATCACCTGCCCAGAAAACAGCCACTATGAGGTCTGTGGCCCACCCTGCCCAGCCAGCTGTCCGTCCCCTGCACCCCTTACGACGCCAGCCGTATGTGAGGGCCCCTGTGTGGAGGGCTGCCAGTGCGACGCGGGTTTCGTGTTAAGTGCTGACCGCTGTGTTCCCCTCAACAACGGCTGCGGCTGCTGGGCCAATGGCACCTACCACGAGGCGGGCAGTGAGTTTTGGGCTGATGGCACCTGCTCCCAGTGGTGTCGCTGCGGGCCTGGGGGTGGCTCGCTGGTCTGCACACCTGCCAGCTGTGGGCTGGGTGAAGTGTGTGGCCTCCTGCCATCCGGCCAGCACGGCTGCCAGCCCGTCAGCACAGCTGAGTGCCAGGCGTGGGGTGACCCCCATTACGTCACTCTGGATGGGCACCGATTCGATTTCCAAGGCACCTGCGAGTACCTGCTGAGTGCACCCTGCCACGGACCACCCTTGGGGGCTGAGAACTTCACTGTCACTGTAGCCAATGAGCACCGGGGCAGCCAGGCTGTCAGCTACACCCGCAGTGTCACCCTGCAAATCTACAACCACAGCCTGACACTGAGTGCCCGCTGGCCCCGGAAGCTACAGGTCGACGGCGTGTTCGTGGCTCTGCCTTTCCAGCTGGACTCGCTCCTGCACGCACACCTGAGCGGCGCCGACGTGGTGGTGACCACAACCTCAGGGCTCTCGCTGGCTTTCGATGGGGACAGCTTCGTGCGCCTGCGCGTGCCGGCGGCGTACGCGGCCTCTCTCTGTGGCTTATGCGGGAACTACAACCAGGACCCCGCAGACGACCTCAAGGCTGTGGGCGGGAAGCCCGCTGGATGGCAGGTGGGCGGGGCCCAGGGCTGCGGGGAATGTGTGTCCAAGCCATGCCCGTCGCCGTGCACCCCAGAGCAGCAGGAGTCCTTCGGCGGCCCGGACGCCTGCGGCGTGATCTCCGCCACCGACGGCCCGCTGGCACCCTGCCACGGCCTTGTGCCGCCCGCGCAGTACTTCCAGGGCTGCTTGCTGGACGCCTGCCAAGTTCAGGGCCATCCTGGAGGCCTCTGTCCTGCAGTGGCTACCTACGTGGCAGCCTGTCAGGCCGCTGGGGCCCAGCTCGGCGAGTGGAGGCGGCCGGACTTCTGTC |
| hsa_circ_07870 | GTGAACGGTGTGGACATGAAGCTGCCCGTGGTGCTGGCCAACGGCCAGATCCGTGCCTCCCAGCATGGTTCAGATGTTGTGATTGAGACCGACTTCGGCCTGCGTGTGGCCTACGACCTTGTGTACTATGTGCGGGTCACCGTCCCTGGAAACTACTACCAGCTGATGTGTGGCCTGTGTGGGAACTACAACGGCGACCCCAAGGATGACTTCCAGAAGCCCAATGGCTCGCAGGCAGGCAACGCCAATGAGTTCGGCAACTCCTGGGAGGAGGTGGTGCCCGACTCTCCCTGCCTGCCGCCGCCCACCTGCCCGCCGGGGAGCGAGGGCTGTATCCCCAGCGAGGAGTGTCCTCCCGAGCTGGAGAAGAAGTATCAGAAGGAGGAGTTCTGTGGGCTCCTCTCCAGCCCCACAGGGCCACTGTCCTCTTGCCACAAGCTGGTGGATCCCCAGGGTCCCTTGAAAGATTGCATCTTTGATCTCTGCCTGGGTGGTGGGAACCTGAGCATTCTCTGCAGCAACATCCATGCCTACGTGAGTGCTTGCCAGGCAGCTGGAGGCCAGGTGGAGCCCTGGAGGAATGAAACTTTCTGTCCCATGGAATGCCCTCAGAACAGTCACTACGAGCTCTGTGCGGACACCTGCTCCCTGGGCTGCTCGGCTCTCAGTGCCCCTCTGCAGTGCCCAGATGGGTGTGCTGAGGGCTGCCAGTGTGACTCCGGCTTCCTCTACAACGGCCAAGCCTGCGTGCCCATCCAGCAATGTGGCTGCTACCACAATGGTGCCTACTATGAGCCGGAGCAGACAGTCCTCATTGACAACTGTCGGCAGCAGTGCACGTGCCATGTGGGTAAAGTCGTGGTGTGCCAGGAACACAGCTGCAAGCCGGGGCAGGTGTGCCAGCCCTCCGGAGGCATCCTGAGCTGCGTCAACAAAGACCCGTGCCACGGCGTGACATGCCGGCCACAGGAGACATGCAAGGAGCAGGGTGGCCAGGGCGTGTGCCTGCCCAACTATGAGGCCACGTGCTGGCTGTGGGGCGACCCACACTACCACTCCTTCGATGGCCGGAAGTTTGACTTCCAGGGCACCTGTAACTATGTGCTGGCAACAACTGGCTGCCCGGGGGTCAGCACCCAGGGCCTGACACCCTTCACCGTCACCACCAAGAACCAGAACCGGGGCAACCCTGCTGTGTCCTACGTGAGAGTCGTCACCGTGGCTGCCCTCGGCACCAACATCTCCATCCACAAGGACGAGATCGGCAAAGTCCGGGTGAACGGTGTGCTCACAGCCTTGCCTGTCTCTGTGGCCGACGGGCGGATTTCAGTGACCCAGGGTGCATCGAAGGCACTGCTGGTGGCTGACTTTGGACTGCAAGTCAGCTATGACTGGAACTGGCGGGTAGACGTGACGCTGCCCAGCAGCTATCATGGCGCAGTGTGCGGGCTCTGCGGTAACATGGACCGCAACCCCAACAATGACCAGGTCTTCCCTAATGGCACACTGGCTCCCTCCATACCCATCTGGGGCGGCAGCTGGCGAGCCCCAGGCTGGGACCCACTGTGTTGGGACGAATGTCGGGGGTCCTGCCCAACGTGCCCTGAGGACCGGTTGGAGCAGTACGAGGGCCCTGGCTTCTGCGGACCCCTGGCCCCCGGCACAGGGGGCCCTTTCACCACCTGCCATGCTCATGTGCCACCTGAGAGCTTCTTCAAGGGCTGTGTTCTGGACGTCTGCATGGGTGGTGGGGACCGTGACATTCTTTGCAAGGCTCTGGCTTCCTATGTGGCCGCCTGCCAGGCTGCTGGGGTTGTCATCGAAGACTGGCGGGCACAGGTTGGCTGTGAGATCACCTGCCCAGAAAACAGCCACTATGAGGTCTGTGGCCCACCCTGCCCGGCCAGCTGTCCGTCCCCTGCACCCCTTACGACGCCAGCCGTATGTGAGGGCCCCTGTGTGGAGGGCTGCCAGTGCGACGCGGGTTTCGTGTTAAGTGCTGACCGCTGTGTTCCCCTCAACAACGGCTGCGGCTGCTGGGCCAATGGCACCTACCACGAGGCGGGCAGTGAGTTTTGGGCTGATGGCACCTGCTCCCAGTGGTGTCGCTGCGGGCCTGGGGGTGGCTCGCTGGTCTGCACACCTGCCAGCTGTGGGCTGGGTGAAGTGTGTGGCCTCCTGCCATCCGGCCAGCACGGCTGCCAGCCCGTCAGCACAGCTGAGTGCCAGGCGTGGGGTGACCCCCATTACGTCACTCTGGATGGGCACCGATTCGATTTCCAAGGCACCTGCGAGTACCTGCTGAGTGCACCCTGCCACGGACCACCCTTGGGGGCTGAGAACTTCACTGTCACTGTAGCCAATGAGCACCGGGGCAGCCAGGCTGTCAGCTACACCCGCAGTGTCACCCTGCAAATCTACAACCACAGCCTGACACTGAGTGCCCGCTGGCCCCGGAAGCTACAGGTGGACGGCGTGTTCGTCACTCTGCCCTTCCAGCTGGACTCGCTCCTGCACGCACACCTGAGCGGCGCCGACGTGGTGGTGACCACAACCTCAGGGCTCTCGCTGGCTTTCGATGGGGACAGCTTCGTGCGCCTGCGCGTGCCGGCGGCGTACGCGGGCTCTCTCTGTGGCTTATGCGGGAACTACAACCAGGACCCCGCAGACGACCTGAAGGCGGTGGGCGGGAAGCCCGCCGGATGGCAGGTGGGCGGCGCCCAGGGCTGCGGGGAATGTGTGTCCAAGCCATGCCCGTCGCCGTGCACCCCAGAGCAGCAAGAGTCCTTCGGCGGCCCGGACGCCTGCGGCGTGATCTCCGCCACCGACGGCCCGCTGGCGCCCTGCCACGGCCTTGTGCCGCCCGCGCAGTACTTCCAGGGCTGCTTGCTGGACGCCTGCCAAGTTCAGGGCCATCCTGGAGGCCTCTGTCCTGCAGTGGCCACCTACGTGGCAGCCTGTCAGGCCGCTGGGGCCCAGCTCCGCGAGTGGAGGCGGCCGGACTTCTGTCCCTTCCAGTGCCCTGCCCACAGCCACTACGAGCTCTGCGGTGACTCCTGTCCTGGGAGCTGCCCGAGCCTGTCGGCACCCGAGGGCTGTGAGTCGGCCTGCCGTGAAGGCTGTGTCTGCGATGCTGGCTTCGTGCTCAGTGGTGACACGTGTGTACCTGTGGGCCAGTGTGGCTGCCTCCACGATGACCGCTACTACCCACTGGGCCAGACCTTCTACCCTGGCCCTGGGTGTGATTCCCTTTGCCGCTGCCGGGAGGGCGGTGAGGTGTCCTGTGAGCCCTCCAGCTGCGGCCCGCATGAGACCTGCCGGCCATCCGGTGGCAGCTTGGGCTGCGTGGCCGTGGGCTCTACCACCTGCCAGGCGTCGGGAGATCCCCACTACACCACCTTCGATGGCCACCGCTTCGACTTCATGGGCACCTGCGTGTATGTGCTGGCTCAGACCTGCGGCACCCGGCCTGGCCTGCATCGGTTTGCCGTCCTGCAGGAGAACGTGGCCTGGGGTAATGGGCGAGTCAGTGTGACCAGGGTGATCACGGTCCAGGTGGCAAACTTCACCCTGCGGCTGGAGCAGAGACAGTGGAAGGTCACGGTGAACGGTGTGGACATGAAGCTGCCCGTGGTGCTGGCCAACGGCCAGATCCGTGCCTCCCAGCATGGTTCAGATGTTGTGATTGAGACCGACTTCGGCCTGCGTGTGGCCTACGACCTTGTGTACTATGTGCGGGTCACCGTCCCTGGAAACTACTACCAGCTGATGTGTGGCCTGTGTGGGAACTACAACGGCGACCCCAAGGATGACTTCCAGAAGCCCAATGGCTCGCAGGCAGGCAACGCCAATGAGTTCGGCAACTCCTGGGAGGAGGTGGTGCCCGACTCTCCCTGCCTGCCGCCGCCCACCTGCCCGCCGGGGAGCGCGGGCTGTATCCCCAGCGACAAGTGTCCTCCCGAGCTGGAGAAGAAGTATCAGAAGGAGGAGTTCTGTGGGCTCCTCTCCAGCCCCACAGGGCCACTGTCCTCCTGCCACAAGCTGGTGGATCCCCAGGGTCCCTTGAAAGATTGCATCTTTGATCTCTGCCTGGGTGGTGGGAACCTGAGCATTCTCTGCAGCAACATCCATGCCTACGTGAGTGCTTGCCAGGCGGCTGGAGGCCACGTGGAGCCCTGGAGGAATGAAACTTTCTGTCCCATGGAATGCCCTCAGAACAGTCACTACGAGCTCTGTGCGGACACCTGCTCCCTGGGCTGCTCGGCTCTCAGTGCCCCTCTGCAGTGCCCAGATGGGTGTGCTGAGGGCTGCCAGTGTGACTCCGGCTTCCTCTACAACGGCCAAGCCTGCGTGCCCATCCAGCAATGTGGCTGCTACCACAATGGTGTCTACTATGAGCCGGAGCAGACAGTCCTCATTGACAACTGTCGGCAGCAGTGCACGTGCCATGTGGGTAAAGTCGTGGTGTGCCAGGAACACAGCTGCAAGCCGGGGCAGGTGTGCCAGCCCTCCGGAGGCATCCTGAGCTGCGTCACCAAAGACCCGTGCCACGGCGTGACATGCCGGCCACAGGAGACATGCAAGGAGCAGGGTGGCCAGGGCGTGTGCCTGCCCAACTATGAGGCCACGTGCTGGCTGTGGGGCGACCCACACTACCACTCCTTCGATGGCCGGAAGTTTGACTTCCAGGGCACCTGTAACTATGTGCTGGCAACAACTGGCTGCCCGGGGGTCAGCACCCAGGGCCTGACACCCTTCACCGTCACCACCAAGAACCAGAACCGGGGCAACCCTGCTGTGTCCTACGTGAGAGTCGTCACCGTGGCTGCCCTCGGCACCAACATCTCCATCCACAAGGACGAGATCGGCAAAGTCCGG |
| hsa_circ_08292 | ATGATTCTGGGCCTCCCCCATCTACTGTCATTAACCAAAATGAAACATTTGCCAACATAATTTTTAAACCTACTGTAGTACAACAAGCCAGGATTGCCCAGAATGGAATTTTGGGAGACTTTATCATTAGATATGACGTCAATAGAGAACAGAGCATTGGGGACATCCAGGTTCTAAATGGCTATTTTGTGCACTACTTTGCTCCTAAAGACCTTCCTCCTTTACCCAAGAATGTGGTATTCGTGCTTGACAGCAGTGCTTCTATGGTGGGAACCAAACTCCGGCAG |
| hsa_circ_08490 | TCGATGCTCCGAGGGAGAGGAGCTTGTTACAAACACACATTCTATGGAATGAGAGCCATCGCTGCATGGAAACCACCCCGAGCTTGGCGTGTGCTAATAAATGTGTCTTCTGTTGGTGGCACCACAACAACCCTGTGGGCACTGAATGGCGGTAGAAGATGGACCAGCCTGAAATGATCTTGAAGGAAGCCATTGAAAACCATCAGAACATGATTAAGCAGTTTAAAG |
| hsa_circ_09183 | GGCAGCGGTCTTAATTCTTTTTATGATCAACGAGAATACATAGGGAGAAGTGTTCATTATTGGAAGAAAGTTTTGCCATTGTTGAAGATAATAAAAAAGAAGAACAGTATTCCTGAACCTATTGATCCTCTGTTTAAACATTTTCATAGTGTAGACATTCAGGCATCAGAAATTGTTGAATATGAAGAAGACGCACACGTAACTTTTGCTATATTGGATGCAGTAAATGGAAATATAGAAGATGCTATGACTGCTTTTGAATCTATAAAAAGTGTTGTTTCTTATTGGAATCTTGCACTG |
| hsa_circ_09631 | AGTGATGACAGACCTAATGCTCTATTAAGTTCACCTGCAACAGAAACAGTTCATCATTCCCCTGCATATTCTTTTCCTGCTGCTATCCAGAGAAATCAGCCTCAGCGCCCTGAAAGCTTCCTTTTCCGAGCAGGTGTCAGGGCAGAAACCAACAAAGGTCATGCTTCACCCCTTCCTCCATCTGCTGCACCTACCACTGATTCTACAGATTCCATAACAGGACAGAATTCAAGACAGAGAGAAGAAGAGCTGGAATTAATAGACCAACTGCGTAAACATATTGAGTACCGGTTGAAAGTGTCTCTACCTTGTGATCTCGGAGCAGCTCTAACTGACGGTGTTGTTCTTTGCCATTTGGCCAATCATGTGCGACCTCGATCTGTCCCAAGCATTCATGTTCCCTCACCAGCTGTACCTAAATTAACAATGGCGAAATGCAGGCGAAATGTGGAAAATTTCCTAGAAGCTTGCAGAAAAATTGGTGTACCTCAG |
| hsa_circ_09985 | AATGCTGGGACCCAGACCCCCACGGGCGGCCAGATTTCGGTAGCATCTTGAAGCGGCTTGAAGTCATCGAACAGTCAGCCCTGTTCCAGATGCCACTGGAGTCCTTCCACTCGCTGCAGGAAGACTGGAAGCTGGAGATTCAGCACATGTTTGATGACCTTCGGACCAAGGAGAAGGAGCTTCGGAGCCGTGAGGAGGAGCTGCTGCGGGCGGCACAGGAGCAGCGCTTCCAGGAGGAGCAGCTGCGGCGGCGGGAGCAGGAGCTGGCAGAACGTGAGATGGACATCGTGGAACGGGAGCTGCACCTGCTCATGTGCCAGCTGAGCCAGGAGAAGCCCCGGGTCCGCAAGCGCAAGGGCAACTTCAAGCGCAGCCGCCTGCTCAAGCTGCGGGAAGGCGGCAGCCACATCAGCCTGCCCTCTGGCTTTGAGCATAAGATCACAGTCCAGGCCTCTCCAACTCTGGATAAGCGGAAAGGATCCGATGGGGCCAGCCCCCCTGCAAGCCCCAGCATCATCCCCCGGCTGAGGGCCATTCGCC |
| hsa_circ_10837 | GGTCTCTCCTCAGGGATGACATCATCCGTCCACCTCCTTGTCTTCAAGGACCACCTCCTCTCCATGCTGAGCTGCTGCCAAGGGGCCTGCTGCCCATCTACACCTCACGAGGGCACTAGGAGCACGGTTTCCTGGATCCCACCAACATACAAAGCAGCCACTCACTGACCCCCAGGACCAGGATGGCAAAGGATGAAGAGGACCGGAACTGACCAGCCAGCTGTCCCTCTTACCTAAAGACTTAAACCAATGCCCTAGTGAGGGGGCATTGGGCATTAAGCCCTGACCTTTGCTATGCTCATACTTTGACTCTATGAGTACTTTCCTATAAGTCTTTGCTTGTGTTCACCTGCTAGCAAACTGGAGTGTTTCCCTCCCCAAGGGGGTGTCAGTCTTTGTCGACTGACTCTGTCATCACCCTTATGATGTCCTGAATGGAAGGATCCCTTTGGGAAATTCTCAGGAGGGGGACCTGGGCCAAGGGCTTGGCCAGCATCCTGCTGGCAACTCCAAGGCCCTGGGTGGGCTTCTGGAATGAGCATGCTACTGAATCACCAAAGGCACGCCCGACCTCTCTGAAGATCTTCCTATCCTTTTCTGGGGGAATGGGGTCGATGAGAGCAACCTCCTAGGGTTGTTGTGAGAATTAAATGAGATAAAAGAGGCCTCAGGCAGGATCTGGCATAGAGGAGGTGATCAGCAAATGTTTGTTGAAAAGGTTTGACAGGTCAGTCCCTTCCCACCCCTCTTGCTTGTCTTACTTGTCTTATTTATTCTCCAACAGCACTCCAGGCAGCCCTTGTCCACGGGCTCTCCTTGCATCAGCCAAGCTTCTTGAAAGGCCTGTCTACACTTGCTGTCTTCCTTCCTCACCTCCAATTTCCTCTTCAACCCACTGCTTCCTGACTCGCTCTACTCCGTGGAAGCACGCTCACAAAGGGCTAATCTCGGGCCTTGTCGAAGGAAGAGGCTGCAGACGTTAATGAGGTTAGCTGCTGGATTCCAGTATTCGTCGCATAAGGATCCTTCTTTGTCTGCGAAGGAAAAACACACTGATTATCATAATGAGTTCCTGACCTGGCCATCCCGGGGTGCCCTTGACCAGCCCCGTGTCTCCTCAGGGTGTCCCAGCACCAGCCTGGCACAGAGTGGGGCTCAGTTAGAGTATGTGGGATGTTGGTTTCGCCAGGCACGTGGGCCGTGGCCCGGCTGGGTCGGCTGAAGAACTGCGGATGGAAGCTGCGGAAGAGGCCCTGATGGGGCCCACCATCCCGGACCCAAGTCTTCTTCCTGGCGGGCCTCTCGTCTCCTTCCTGGTTTGGGCGGAAGCCATCACCTGGATGCCTACGTGGGAAGGGACCTCGAATGTGGGACCCCAGCCCCTCTCCAGCTCGAAATC |
| hsa_circ_12240 | AATTCTGCTTGGTCAGAATCGTGATGGCATTGTGTTCAGCACTGATGACTATTTTCACCATCAAGATGGGTACAGGTATAATGTTAATCAACTTGGTGATGCCCATGACTGGAACCAGAACAGAGCAAAACAAGCTATCGATCAGGGAAGATCTCCAGTTATAATAGATAACACTAATATACAAGCTTGGGAAATGAAGCCATATGTGGAAGTGGCCATAGGAAAAGGATACAGAGTAGAGTTTCATGAACCTGAAACTTGGTGGAAATTTGATCCTGAAGAATTAGAAAAGAGGAATAAACATGGTGTGTCTCGAAAGAAGATTGCTCAGATGTTGGATCGTTATGAATATCAAATGTCCATTTCTATTGTAATGAATTCAGTGGAACCATCACACAAAAGCACACAAAGACCTCCTCCTCCACAGGGGAGACAGAG |
| hsa_circ_13113 | ATATTGATGAGTGCATCCAGAATGGGGTTCTTTGTAAAAACGGTCGATGCGTGAACACAGATGGAAGTTTCCAGTGCATTTGCAATGCCGGCTTTGAATTAACTACAGATGGAAAAAACTGTGTTGATCATGATGAATGTACAACTACCAACATGTGTTTGAATGGAATGTGCATCAATGAAGATGGCAGCTTCAAGTGCATCTGCAAACCAGGATTTGTCTTGGCTCCAAATGGGCGTTACTGTACTGATGTTGATGAATGCCAGACCCCAGGAATCTGCATGAATGGGCACTGCATCAACAGTGAAGGGTCCTTCCGCTGTGACTGTCCCCCAGGCCTGGCTGTGGGCATGGATGGACGTGTGTGTGTTGATACTCACATGCGCAGTACCTGCTATGGAGGAATCAAGAAAGGAGTGTGTGTGCGTCCTTTCCCCGGTGCAGTGACCAAGTCCGAATGCTGCTGTGCCAATCCAGACTATGGTTTTGGAGAACCCTGCCAGCCATGCCCTGCAAAAAATTCAGCTGAATTCCACGGCCTTTGTAGTAGTGGAGTAGGTATCACTGTGGATGGAAGAGATATCAATGAATGTGCTTTGGATCCTGATATATGTGCCAATGGGATTTGTGAAAACTTACGTGGTAGTTACCGTTGTAATTGCAACAGTGGCTATGAACCAGATGCCTCTGGAAGAAACTGTATTGACATTGATGAATGTTTAGTAAACAGACTGCTTTGTGATAACGGATTGTGCCGAAACACGCCAGGAAGTTACAGCTGTACGTGCCCACCAGGGTATGTGTTCAGGACTGAGACAGAGACCTGTGAAGATATAAATGAATGTGAAAGCAACCCATGTGTCAATGGGGCCTGCAGAAACAACCTTGGATCTTTCAATTGTGAATGTTCGCCCGGCAGCAAACTCAGCTCCACAGGATTGATCTGTATTGACAGCCTGAAGGGGACCTGTTGGCTCAACATCCAGGACAGCCGCTGTGAGGTGAATATTAATGGAGCCACTCTGAAATCTGAATGCTGTGCCACCCTCGGAGCCGCCTGGGGGAGCCCCTGTGAGCGGTGTGAACTAGATACAGCTTGCCCAAGAGGGCTTGCCAGGATTAAAGGTGTTACGTGTGAAGATGTTAATGAGTGTGAGGTGTTCCCTGGCGTTTGTCCAAATGGACGCTGTGTCAACAGTAAGGGATCTTTTCATTGCGAGTGCCCTGAAGGCCTTACGTTGGATGGGACTGGCCGTGTATGTTTGGATATTCGCATGGAGCAGTGTTACTTGAAGTGGGATGAAGATGAATGCATCCACCCCGTTCCTGGAAAGTTCCGCATGGATGCCTGCTGCTGTGCTGTCGGGGCGGCTTGGGGCACCGAGTGTGAGGAGTGCCCCAAACCTGGCACCAAGGAATACGAGACGCTGTGCCCCCGCGGGGCTGGCTTTGCTAACCGAGGGGATGTTCTTACTGGGCGGCCATTTTACAAAGACATCAATGAATGCAAAGCATTTCCTGGGATGTGCACTTATGGGAAGTGCAGAAATACAATCGGAAGCTTCAAATGCCGTTGCAATAGTGGCTTTGCTCTAGACATGGAGGAAAGAAACTGCACGGACATCGACGAGTGCAGGATTTCTCCTGACCTCTGTGGCAGTGGAATCTGCGTCAATACACCGGGCAGCTTTGAGTGCGAGTGCTTCGAAGGCTATGAAAGTGGCTTCATGATGATGAAGAACTGCATGGACATTGACGAATGTGAACGTAACCCTCTCCTTTGTAGGGGTGGCACCTGTGTGAACACTGAGGGCAGCTTTCAGTGTGACTGCCCACTGGGACACGAGCTGTCACCATCCCGTGAGGACTGTGTGGATATTAATGAATGCTCCCTGAGTGACAATCTCTGCAGAAATGGAAAATGTGTGAACATGATTGGAACCTATCAGTGCTCTTGCAATCCTGGATATCAGGCTACGCCAGACCGCCAGGGCTGTACAGATATTGATGAATGTATGATAATGAACGGAGGCTGTGACACCCAGTGCACAAATTCAGAGGGAAGCTACGAATGCAGCTGCAGTGAGGGTTATGCCCTGATGCCAGATGGGAGATCGTGTGCAGACATTGATGAATGTGAAAACAATCCTGATATCTGTGATGGCGGCCAGTGTACCAACATTCCTGGAGAGTATCGCTGCCTCTGCTATGATGGCTTCATGGCTTCCATGGACATGAAAACATGCATTGATGTCAATGAATGTGACCTAAATTCAAATATCTGCATGTTTGGGGAATGTGAGAACACAAAGGGATCCTTCATTTGCCACTGTCAGCTGGGTTACTCAGTGAAGAAGGGGACCACAGGATGTACAGATGTGGATGAGTGTGAAATTGGTGCTCATAACTGCGACATGCATGCCTCATGTCTGAATATCCCAGGAAGCTTCAAGTGTAGCTGCAGAGAAGGCTGGATTGGAAACGGCATCAAGTGTATTGATCTGGACGAATGTTCTAATGGAACCCACCAGTGTAGCATCAATGCTCAGTGTGTAAATACCCCGGGCTCATACCGCTGTGCCTGCTCCGAAGGTTTCACTGGTGATGGCTTTACCTGCTCAGATGTTGATGAGTGTGCAGAAAACATAAACCTCTGTGAGAACGGACAGTGCCTTAATGTCCCGGGTGCATATCGCTGCGAGTGTGAGATGGGCTTCACTCCAGCCTCAGACAGCAGATCCTGCCAAGATATTGATGAATGCTCCTTCCAAAACATTTGTGTCTTTGGAACATGTAATAACCTGCCTGGAATGTTTCATTGCATCTGCGATGATGGTTATGAATTGGACAGAACAGGAGGGAACTGTACAGATATTGATGAGTGTGCAGATCCTATAAACTGTGTCAATGGCCTATGTGTCAACACGCCTGGTCGCTATGAGTGTAACTGCCCACCCGATTTTCAGTTGAACCCAACTGGTGTGGGTTGTGTTGACAACCGTGTGGGCAACTGCTACCTGAAGTTTGGACCTCGAGGAGATGGGAGTCTGTCTTGCAACACCGAGATCGGGGTGGGCGTCAGTCGCTCTTCATGCTGCTGCTCTCTGGGAAAGGCCTGGGGAAACCCCTGTGAGACATGCCCCCCTGTCAATAGCACTGAATATTACACCCTGTGTCCCGGAGGTGAAGGCTTCAGACCTAACCCCATCACAATCATTTTAGAAGACATTGACGAATGCCAGGAGTTACCAGGTCTCTGCCAGGGTGGAAACTGCATCAACACTTTTGGGAGCTTCCAGTGTGAGTGCCCACAAGGCTACTACCTCAGCGAGGATACCCGCATCTGTGAAG |
| hsa_circ_13568 | TGGCAGCCGATTAGGAGGCCTGGTTACAGTGTTGTGCTTCTTTTTCAATCATGGAGAGTGTACCCGTGTAATGTGGACACCACCTCTCCGTGAAAGCTTCTCATATCCATTTCTTGTTCTTCAGATGTTGCTAGTGACTCATATTCTCAG |
| hsa_circ_14833 | GGACTCTTGACGTTTAGGTATGTGGCCATAGAATTCTCTCTGGAGGAGTAGCAGTGCCTGGACACTGCACAGCAGAATTTATAGAGGAATGTTATGAAAACCTGGCCTTCCTGGGTATTACTGTCTCTAAGCCAGACCTGATCACCTATCTGGAGCGAGAAAAAGAGCCCTGGAATATGAAGTGACATGAGATGGTGGATGAACCCCCAG |
| hsa_circ_15550 | GGCTGGTGAATTACCAGATCTCCGTCAAGTGCAGTAACCAGTTCAAGTTGGAAGTGTGTCTTTTGAATGCAGAAAACAAAGTCGTGGACAACCAGGCTGGGACCCAGGGCCAGCTGAAGGTGCTGGGTGCCAACCTCTGGTGGCCGTACCTGATGCACGAACACCCCGCCTCCCTGTACTCGTGGGAGGATGGTGATTGCTCACACCAAAGCCTTGGACCCCTCCCAGCCTGTGACCTTTGTGACCAACTCCACCTACGCAGCAGACAAGGGGGCTCTGTATGTGGATGTGATCCGTGTGAACAGCTACTACTCTTGGTATCGCAACTACGGGCACCTGGAGTTGATTCAGCTGCAGCTGGCCGCCCAGTTTGAGAATTGGTGTAAGACATCACAATCCCATTATTCAGAGCGCGTATGGAGTGGAAACGCTTGTAAGGCTTCACCAGGATCCACCTCTGATGTTCAGTGAAGAGGACCGGAAAAGTCTGCTAGAGCAGTACCATCTGGGTCTGGATCAAAAACGCAGAAAATACGTGGTTGGAGAGCTCATCTGGAATTTTGCCGATTTCATGACTAACCAGT |
| hsa_circ_16621 | GAATTCCTTGTGGGCTACCCCCCACCATCACCAATGGAGATTTCATTAGCACCAACAGAGAGAATTTTCACTATGGATCAGTGGTGACCTACCGCTGCAATCCTGGAAGCGGAGGGAGAAAGGTGTTTGAGCTTGTGGGTGAGCCCTCCATATACTGCACCAGCAATGACGATCAAGTGGGCATCTGGAGCGGCCCCGCCCCTCAGTGCATTATACCTAACAAATGCACGCCTCCAAATGTGGAAAATGGAATATTGGTATCTGACAACAGAAGCTTATTTTCCTTAAATGAAGTTGTGGAGTTTAGGTGTCAGCCTGGCTTTGTCATGAAAGGACCCCGCCGTGTGAAGTGCCAGGCCCTGAACAAATGGGAGCCGGAGCTACCAAGCTGCTCCAGGGTATGTCAGCCACCTCCAGATGTCCTGCATGCTGAGCGTACCCAAAGGGACAAGGACAACTTTTCACCTGGGCAGGAAGTGTTCTACAGCTGTGAGCCCGGCTACGACCTCAGAGGGGCTGCGTCTATGCGCTGCACACCCCAGGGAGACTGGAGCCCTGCAGCCCCCACATGTGAAGTGAAATCCTGTGATGACTTCATGGGCCAACTTCTTAATGGCCGTGTGCTATTTCCAGTAAATCTCCAGCTTGGAGCAAAAGTGGATTTTGTTTGTGATGAAGGATTTCAATTAAAAGGCAGCTCTGCTAGTTACTGTGTCTTGGCTGGAATGGAAAGCCTTTGGAATAGCAGTGTTCCAGTGTGTGAACAAATCTTTTGTCCAAGTCCTCCAGTTATTCCTAATGGGAGACACACAGGAAAACCTCTGGAAGTCTTTCCCTTTGGGAAAACAGTAAATTACACATGCGACCCCCACCCAGACAGAGGGACGAGCTTCGACCTCATTGGAGAGAGCACCATCCGCTGCACAAGTGACCCTCAAGGGAATGGGGTTTGGAGCAGCCCTGCCCCTCGCTGTGGAATTCTGGGTCACTGTCAAGCCCCAGATCATTTTCTGTTTGCCAAGTTGAAAACCCAAACCAATGCATCTGACTTTCCCATTGGGACATCTTTAAAGTACGAATGCCGTCCTGAGTACTACGGGAGGCCATTCTCTATCACATGTCTAGATAACCTGGTCTGGTCAAGTCCCAAAGATGTCTGTAAACGTAAATCATGTAAAACTCCTCCAGATCCAGTGAATGGCATGGTGCATGTGATCACAGACATCCAGGTTGGATCCAGAATCAACTATTCTTGTACTACAGGGCACCGACTCATTGGTCACTCATCTGCTGAATGTATCCTCTCGGGCAATGCTGCCCATTGGAGCACGAAGCCGCCAATTTGTCAACGAATTCCTTGTGGGCTACCCCCCACCATCGCCAATGGAGATTTCATTAGCACCAACAGAGAGAATTTTCACTATGGATCAGTGGTGACCTACCGCTGCAATCCTGGAAGCGGAGGGAGAAAGGTGTTTGAGCTTGTGGGTGAGCCCTCCATATACTGCACCAGCAATGACGATCAAGTGGGCATCTGGAGCGGCCCGGCCCCTCAGTGCATTATACCTAACAAATGCACGCCTCCAAATGTGGAAAATGGAATATTGGTATCTGACAACAGAAGCTTATTTTCCTTAAATGAAGTTGTGGAGTTTAGGTGTCAGCCTGGCTTTGTCATGAAAGGACCCCGCCGTGTGAAGTGCCAGGCCCTGAACAAATGGGAGCCGGAGCTACCAAGCTGCTCCAGGGTATGTCAGCCACCTCCAGATGTCCTGCATGCTGAGCGTACCCAAAGGGACAAGGACAACTTTTCACCCGGGCAGGAAGTGTTCTACAGCTGTGAGCCCGGCTACGACCTCAGAGGGGCTGCGTCTATGCGCTGCACACCCCAGGGAGACTGGAGCCCTGCAGCCCCCACATGTGAAGTGAAATCCTGTGATGACTTCATGGGCCAACTTCTTAATGGCCGTGTGCTATTTCCAGTAAATCTCCAGCTTGGAGCAAAAGTGGATTTTGTTTGTGATGAAGGATTTCAATTAAAAGGCAGCTCTGCTAGTTACTGTGTCTTGGCTGGAATGGAAAGCCTTTGGAATAGCAGTGTTCCAGTGTGTGAACAAATCTTTTGTCCAAGTCCTCCAGTTATTCCTAATGGGAGACACACAGGAAAACCTCTGGAAGTCTTTCCCTTTGGGAAAACAGTAAATTACACATGCGACCCCCACCCAGACAGAGGGACGAGCTTCGACCTCATTGGAGAGAGCACCATCCGCTGCACAAGTGACCCTCAAGGGAATGGGGTTTGGAGCAGCCCTGCCCCTCGCTGTGGAATTCTGGGTCACTGTCAAGCCCCAGATCATTTTCTGTTTGCCAAGTTGAAAACCCAAACCAATGCATCTGACTTTCCCATTGGGACATCTTTAAAGTACGAATGCCGTCCTGAGTACTACGGGAGGCCATTCTCTATCACATGTCTAGATAACCTGGTCTGGTCAAGTCCCAAAGATGTCTGTAAACGTAAATCATGTAAAACTCCTCCAGATCCAGTGAATGGCATGGTGCATGTGATCACAGACATCCAGGTTGGATCCAGAATCAACTATTCTTGTACTACAGGGCACCGACTCATTGGTCACTCATCTGCTGAATGTATCCTCTCGGGCAATGCTGCCCATTGGAGCACGAAGCCGCCAATTTGTCAACGAATTCCTTGTGGGCTACCCCCCACCATCGCCAATGGAGATTTCATTAGCACCAACAGAGAGAATTTTCACTATGGATCAGTGGTGACCTACCGCTGCAATCCTGGAAGCGGAGGGAGAAAGGTGTTTGAGCTTGTGGGTGAGCCCTCCATATACTGCACCAGCAATGACGATCAAGTGGGCATCTGGAGCGGCCCGGCCCCTCAGTGCATTATACCTAACAAATGCACGCCTCCAAATGTGGAAAATGGAATATTGGTATCTGACAACAGAAGCTTATTTTCCTTAAATGAAGTTGTGGAGTTTAGGTGTCAGCCTGGCTTTGTCATGAAAGGACCCCGCCGTGTGAAGTGCCAGGCCCTGAACAAATGGGAGCCGGAGCTACCAAGCTGCTCCAGGGTATGTCAGCCACCTCCAGATGTCCTGCATGCTGAGCGTACCCAAAGGGACAAGGACAACTTTTCACCCGGGCAGGAAGTGTTCTACAGCTGTGAGCCCGGCTATGACCTCAGAGGGGCTGCGTCTATGCGCTGCACACCCCAGGGAGACTGGAGCCCTGCAGCCCCCACATGTGAAGTGAAATCCTGTGATGACTTCATGGGCCAACTTCTTAATGGCCGTGTGCTATTTCCAGTAAATCTCCAGCTTGGAGCAAAAGTGGATTTTGTTTGTGATGAAGGATTTCAATTAAAAGGCAGCTCTGCTAGTTATTGTGTCTTGGCTGGAATGGAAAGCCTTTGGAATAGCAGTGTTCCAGTGTGTGAACAAATCTTTTGTCCAAGTCCTCCAGTTATTCCTAATGGGAGACACACAGGAAAACCTCTGGAAGTCTTTCCCTTTGGAAAAGCAGTAAATTACACATGCGACCCCCACCCAGACAGAGGGACGAGCTTCGACCTCATTGGAGAGAGCACCATCCGCTGCACAAGTGACCCTCAAGGGAATGGGGTTTGGAGCAGCCCTGCCCCTCGCTGTGGAATTCTGGGTCACTGTCAAGCCCCAGATCATTTTCTGTTTGCCAAGTTGAAAACCCAAACCAATGCATCTGACTTTCCCATTGGGACATCTTTAAAGTACGAATGCCGTCCTGAGTACTACGGGAGGCCATTCTCTATCACATGTCTAGATAACCTGGTCTGGTCAAGTCCCAAAGATGTCTGTAAACGTAAATCATGTAAAACTCCTCCAGATCCAGTGAATGGCATGGTGCATGTGATCACAGACATCCAGGTTGGATCCAGAATCAACTATTCTTGTACTACAGGGCACCGACTCATTGGTCACTCATCTGCTGAATGTATCCTCTCAGGCAATACTGCCCATTGGAGCACGAAGCCGCCAATTTGTCAAC |
| hsa_circ_16871 | ATATTGATGAGTGTCAGAGAGATCCTCTCCTATGCCGAGGTGGTGTTTGCCATAACACAGAGGGAAGTTACCGCTGTGAATGCCCGCCTGGCCATCAGCTGTCCCCCAACATCTCCGCGTGTATCGACATCAATGAATGTGAGCTGAGTGCACACCTGTGCCCCAATGGCCGTTGCGTGAACCTCATAGGGAAGTATCAGTGTGCCTGCAACCCTGGCTACCATTCAACTCCCGATAGGCTATTTTGTGTTGACATTGATGAATGCAGCATAATGAATGGTGGTTGTGAAACCTTCTGCACAAACTCTGAAGGCAGCTATGAATGTAGCTGTCAGCCGGGATTTGCACTAATGCCTGACCAGAGATCATGCACCGACATCGATGAGTGTGAAGATAATCCCAATATCTGTGATGGTGGTCAGTGCACAAATATCCCTGGAGAGTACAGGTGCTTGTGTTATGATGGATTCATGGCATCTGAAGACATGAAGACTTGTGTAGATGTCAATGAGTGTGACCTGAATCCAAATATCTGCCTAAGTGGGACCTGTGAAAACACGAAAGGCTCATTTATCTGCCACTGTGATATGGGCTACTCCGGCAAAAAAGGAAAAACTGGCTGTACAGACATCAATGAATGTGAAATTGGAGCACACAACTGTGGCAAACATGCTGTATGTACCAATACAGCAGGAAGCTTCAAATGTAGCTGCAGTCCCGGGTGGATTGGAGATGGCATTAAGTGCACTGATCTGGACGAATGTTCCAATGGAACCCATATGTGCAGCCAGCATGCAGACTGCAAGAATACCATGGGATCTTACCGCTGTCTGTGCAAGGAAGGATACACAGGTGATGGCTTCACTTGTACAGACCTTGATGAGTGCTCTGAGAACCTGAATCTCTGTGGCAATGGCCAGTGCCTCAATGCACCAGGAGGATACCGCTGTGAATGCGACATGGGCTTCGTGCCCAGTGCTGACGGGAAAGCCTGTGAAG |
| hsa_circ_19485 | GAATTCCTTGTGGGCTACCCCCCACCATCACCAATGGAGATTTCATTAGCACCAACAGAGAGAATTTTCACTATGGATCAGTGGTGACCTACCGCTGCAATCCTGGAAGCGGAGGGAGAAAGGTGTTTGAGCTTGTGGGTGAGCCCTCCATATACTGCACCAGCAATGACGATCAAGTGGGCATCTGGAGCGGCCCCGCCCCTCAGTGCATTATACCTAACAAATGCACGCCTCCAAATGTGGAAAATGGAATATTGGTATCTGACAACAGAAGCTTATTTTCCTTAAATGAAGTTGTGGAGTTTAGGTGTCAGCCTGGCTTTGTCATGAAAGGACCCCGCCGTGTGAAGTGCCAGGCCCTGAACAAATGGGAGCCGGAGCTACCAAGCTGCTCCAGGGTATGTCAGCCACCTCCAGATGTCCTGCATGCTGAGCGTACCCAAAGGGACAAGGACAACTTTTCACCTGGGCAGGAAGTGTTCTACAGCTGTGAGCCCGGCTACGACCTCAGAGGGGCTGCGTCTATGCGCTGCACACCCCAGGGAGACTGGAGCCCTGCAGCCCCCACATGTGAAGTGAAATCCTGTGATGACTTCATGGGCCAACTTCTTAATGGCCGTGTGCTATTTCCAGTAAATCTCCAGCTTGGAGCAAAAGTGGATTTTGTTTGTGATGAAGGATTTCAATTAAAAGGCAGCTCTGCTAGTTACTGTGTCTTGGCTGGAATGGAAAGCCTTTGGAATAGCAGTGTTCCAGTGTGTGAACAAATCTTTTGTCCAAGTCCTCCAGTTATTCCTAATGGGAGACACACAGGAAAACCTCTGGAAGTCTTTCCCTTTGGGAAAACAGTAAATTACACATGCGACCCCCACCCAGACAGAGGGACGAGCTTCGACCTCATTGGAGAGAGCACCATCCGCTGCACAAGTGACCCTCAAGGGAATGGGGTTTGGAGCAGCCCTGCCCCTCGCTGTGGAATTCTGGGTCACTGTCAAGCCCCAGATCATTTTCTGTTTGCCAAGTTGAAAACCCAAACCAATGCATCTGACTTTCCCATTGGGACATCTTTAAAGTACGAATGCCGTCCTGAGTACTACGGGAGGCCATTCTCTATCACATGTCTAGATAACCTGGTCTGGTCAAGTCCCAAAGATGTCTGTAAACGTAAATCATGTAAAACTCCTCCAGATCCAGTGAATGGCATGGTGCATGTGATCACAGACATCCAGGTTGGATCCAGAATCAACTATTCTTGTACTACAGGGCACCGACTCATTGGTCACTCATCTGCTGAATGTATCCTCTCGGGCAATGCTGCCCATTGGAGCACGAAGCCGCCAATTTGTCAACGAATTCCTTGTGGGCTACCCCCCACCATCGCCAATGGAGATTTCATTAGCACCAACAGAGAGAATTTTCACTATGGATCAGTGGTGACCTACCGCTGCAATCCTGGAAGCGGAGGGAGAAAGGTGTTTGAGCTTGTGGGTGAGCCCTCCATATACTGCACCAGCAATGACGATCAAGTGGGCATCTGGAGCGGCCCGGCCCCTCAGTGCATTATACCTAACAAATGCACGCCTCCAAATGTGGAAAATGGAATATTGGTATCTGACAACAGAAGCTTATTTTCCTTAAATGAAGTTGTGGAGTTTAGGTGTCAGCCTGGCTTTGTCATGAAAGGACCCCGCCGTGTGAAGTGCCAGGCCCTGAACAAATGGGAGCCGGAGCTACCAAGCTGCTCCAGGGTATGTCAGCCACCTCCAGATGTCCTGCATGCTGAGCGTACCCAAAGGGACAAGGACAACTTTTCACCCGGGCAGGAAGTGTTCTACAGCTGTGAGCCCGGCTACGACCTCAGAGGGGCTGCGTCTATGCGCTGCACACCCCAGGGAGACTGGAGCCCTGCAGCCCCCACATGTGAAGTGAAATCCTGTGATGACTTCATGGGCCAACTTCTTAATGGCCGTGTGCTATTTCCAGTAAATCTCCAGCTTGGAGCAAAAGTGGATTTTGTTTGTGATGAAGGATTTCAATTAAAAGGCAGCTCTGCTAGTTACTGTGTCTTGGCTGGAATGGAAAGCCTTTGGAATAGCAGTGTTCCAGTGTGTGAACAAATCTTTTGTCCAAGTCCTCCAGTTATTCCTAATGGGAGACACACAGGAAAACCTCTGGAAGTCTTTCCCTTTGGGAAAACAGTAAATTACACATGCGACCCCCACCCAGACAGAGGGACGAGCTTCGACCTCATTGGAGAGAGCACCATCCGCTGCACAAGTGACCCTCAAGGGAATGGGGTTTGGAGCAGCCCTGCCCCTCGCTGTGGAATTCTGGGTCACTGTCAAGCCCCAGATCATTTTCTGTTTGCCAAGTTGAAAACCCAAACCAATGCATCTGACTTTCCCATTGGGACATCTTTAAAGTACGAATGCCGTCCTGAGTACTACGGGAGGCCATTCTCTATCACATGTCTAGATAACCTGGTCTGGTCAAGTCCCAAAGATGTCTGTAAACGTAAATCATGTAAAACTCCTCCAGATCCAGTGAATGGCATGGTGCATGTGATCACAGACATCCAGGTTGGATCCAGAATCAACTATTCTTGTACTACAGGGCACCGACTCATTGGTCACTCATCTGCTGAATGTATCCTCTCGGGCAATGCTGCCCATTGGAGCACGAAGCCGCCAATTTGTCAAC |
| hsa_circ_20137 | GTGAACGGTGTGGACATGAAGCTGCCCGTGGTGCTGGCCAACGGCCAGATCCGTGCCTCCCAGCATGGTTCAGATGTTGTGATTGAGACCGACTTCGGCCTGCGTGTGGCCTACGACCTTGTGTACTATGTGCGGGTCACCGTCCCCGGAAACTACTACCAGCAGATGTGTGGCCTGTGTGGGAACTACAACGGCGACCCCAAGGATGACTTCCAGAAGCCCAATGGCTCACAGGCAGGCAACGCCAATGAGTTCGGCAACTCCTGGGAGGAGGTGGTGCCCGACTCTCCCTGCCTGCCGCCCACCCCTTGCCCGCCGGGGAGCGAGGACTGTATCCCCAGCCACAAGTGTCCTCCCGAGCTGGAGAAGAAGTATCAGAAGGAGGAGTTCTGTGGGCTCCTCTCCAGCCCCACAGGGCCACTGTCCTCCTGCCACAAGCTGGTGGATCCCCAGGGTCCCTTGAAAGATTGCATCTTTGATCTCTGCCTGGGTGGTGGGAACCTGAGCATTCTCTGCAGCAACATCCATGCCTACGTGAGTGCTTGCCAGGCGGCTGGAGGCCACGTGGAGCCCTGGAGGACTGAAACTTTCTGTCCCATGGAGTGCCCTCCGAACAGTCACTACGAGCTCTGTGCGGACACCTGCTCCCTGGGCTGCTCAGCTCTCAGTGCCCCTCCACAGTGCCAGGATGGGTGTGCTGAGGGCTGCCAGTGTGACTCCGGCTTCCTCTACAATGGCCAAGCCTGCGTGCCCATCCAGCAATGCGGCTGCTACCACAATGGTGTCTACTATGAGCCGGAGCAGACAGTCCTCATTGACAACTGTCGGCAGCAGTGCACGTGCCATGCGGGTAAAGGCATGGTGTGCCAGGAACACAGCTGCAAGCCGGGGCAGGTGTGCCAGCCCTCCGGAGGCATCCTGAGCTGCGTCACCAAAGACCCGTGCCACGGCGTGACATGCCGGCCACAGGAGACATGCAAGGAGCAGGGTGGCCAGGGCGTGTGCCTGCCCAACTATGAGGCCACGTGCTGGCTGTGGGGCGACCCACACTACCACTCCTTCGATGGCCGGAAGTTTGACTTCCAGGGCACCTGTAACTATGTGCTGGCAACAACTGGCTGCCCGGGGGTCAGCACCCAGGGCCTGACACCCTTCACCGTCACCACCAAGAACCAGAACCGGGGCAACCCTGCTGTGTCCTACGTGAGAGTCGTCACCGTGGCTGCCCTCGGCACCAACATCTCCATCCACAAGGACGAGATCGGCAAAGTCCGG |
| hsa_circ_21613 | GGTGCTGACGGCCAACCTGGTGCTAAAGGCGAACCTGGTGATGCTGGTGCTAAAGGCGATGCTGGTCCCCCTGGCCCTGCCGGACCCGCTGGACCCCCTGGCCCCATTGGTAATGTTGGTGCTCCTGGAGCCAAAGGTGCTCGCGGCAGCGCTGGTCCCCCTGGTGCTACTGGTTTCCCTGGTGCTGCTGGCCGAGTCGGTCCTCCTGGCCCCTCTGGAAATGCTGGACCCCCTGGCCCTCCTGGTCCTGCTGGCAAAGAAGGCGGCAAAGGTCCCCGTGGTGAGACTGGCCCTGCTGGACGTCCTGGTGAAGTTGGTCCCCCTGGTCCCCCTGGCCCTGCTGGCGAGAAAGGATCCCCTGGTGCTGATGGTCCTGCT |
| hsa_circ_22296 | GTGAACGGTGTGGACATGAAGCTGCCCGTGGTGCTGGCCAACGGCCAGATCCGTGCCTCCCAGCATGGTTCAGATGTTGTGATTGAGACCGACTTCGGCCTGCGTGTGGCCTACGACCTTGTGTACTATGTGCGGGTCACCGTCCCTGGAAACTACTACCAGCTGATGTGTGGCCTGTGTGGGAACTACAACGGCGACCCCAAGGATGACTTCCAGAAGCCCAATGGCTCGCAGGCAGGCAACGCCAATGAGTTCGGCAACTCCTGGGAGGAGGTGGTGCCCGACTCTCCCTGCCTGCCGCCGCCCACCTGCCCGCCGGGGAGCGCGGGCTGTATCCCCAGCGACAAGTGTCCTCCCGAGCTGGAGAAGAAGTATCAGAAGGAGGAGTTCTGTGGGCTCCTCTCCAGCCCCACAGGGCCACTGTCCTCCTGCCACAAGCTGGTGGATCCCCAGGGTCCCTTGAAAGATTGCATCTTTGATCTCTGCCTGGGTGGTGGGAACCTGAGCATTCTCTGCAGCAACATCCATGCCTACGTGAGTGCTTGCCAGGCGGCTGGAGGCCACGTGGAGCCCTGGAGGAATGAAACTTTCTGTCCCATGGAATGCCCTCAGAACAGTCACTACGAGCTCTGTGCGGACACCTGCTCCCTGGGCTGCTCGGCTCTCAGTGCCCCTCTGCAGTGCCCAGATGGGTGTGCTGAGGGCTGCCAGTGTGACTCCGGCTTCCTCTACAACGGCCAAGCCTGCGTGCCCATCCAGCAATGTGGCTGCTACCACAATGGTGTCTACTATGAGCCGGAGCAGACAGTCCTCATTGACAACTGTCGGCAGCAGTGCACGTGCCATGTGGGTAAAGTCGTGGTGTGCCAGGAACACAGCTGCAAGCCGGGGCAGGTGTGCCAGCCCTCCGGAGGCATCCTGAGCTGCGTCACCAAAGACCCGTGCCACGGCGTGACATGCCGGCCACAGGAGACATGCAAGGAGCAGGGTGGCCAGGGCGTGTGCCTGCCCAACTATGAGGCCACGTGCTGGCTGTGGGGCGACCCACACTACCACTCCTTCGATGGCCGGAAGTTTGACTTCCAGGGCACCTGTAACTATGTGCTGGCAACAACTGGCTGCCCGGGGGTCAGCACCCAGGGCCTGACACCCTTCACCGTCACCACCAAGAACCAGAACCGGGGCAACCCTGCTGTGTCCTACGTGAGAGTCGTCACCGTGGCTGCCCTCGGCACCAACATCTCCATCCACAAGGACGAGATCGGCAAAGTCCGG |
| hsa_circ_22913 | GGTGAAGGTGGTGCCCCCGGACTTCCAGGTATAGCTGGACCTCGTGGTAGCCCT |
| hsa_circ_24068 | ACTGTCACTCTCTGTTGCCCAAGTGCCTTGGTGTGGAATTATTCCACAAATGAAAATAAGAGCGCAAACCCAGGCTCACCCCTGGATCTGCTGCAGGTGGCCCCGTCCAGCCTCCCCATGCCGGGTGGGAACACGGCTTTCAATCAGCAGGTTCGGGCAAGGATTTATGAAGTAGAACAACAGATAAAACAAAGAGGCCGTGCAGTGGAAGTTCGGTGGTCATTTGACAAGTGCCAAGAATCCACAGCAGGGGTGACTATTAGTCGGGTTTTGCACACGTTGGAAGTTTTGGATCGTCACTGTTTTGACCGAACTGATTCCAGCAATTCCATGGAGACACTTTATCATAAGATTTTCTGGGCAAACCAAAACAAAGATAACCAAGAG |
| hsa_circ_24869 | GGTGCTCCTGGTATTGCTGGTGCTCCTGGCTTCCCTGGTGCCCGAGGCCCCTCTGGACCCCAGGGCCCCGGCGGCCCTCCTGGTCCCAAGGGTAACAGCGGTGAACCTGGTGCTCCTGGCAGCAAAGGAGACACTGGTGCTAAGGGAGAGCCTGGCCCTGTTGGTGTTCAAGGACCCCCTGGCCCTGCTGGAGAGGAAGGAAAGCGAGGAGCTCGAGGTGAACCCGGACCCACTGGCCTGCCCGGACCCCCTGGCGAGCGTGGTGGACCTGGTAGCCGTGGTTTCCCTGGCGCAGATGGTGTTGCTGGTCCCAAGGGTCCCGCTGGTGAACGTGGTTCTCCTGGCCCTGCTGGCCCCAAAGGATCTCCTGGTGAAGCTGGTCGTCCCGGTGAAGCTGGTCTGCCTGGTGCCAAGGGTCTGACTGGAAGCCCTGGCAGCCCTGGTCCTGATGGCAAAACTGGCCCCCCTGGTCCCGCCGGTCAAGATGGTCGCCCCGGACCCCCAGGCCCACCTGGTGCCCGTGGTCAGGCTGGTGTGATGGGATTCCCTGGACCTAAAGGTGCTGCTGGAGAGCCCGGCAAGGCTGGAGAGCGAGGTGTTCCCGGACCCCCTGGCGCTGTCGGTCCTGCTGGCAAAGATGGAGAGGCTGGAGCTCAGGGACCCCCTGGCCCTGCTGGTCCCGCTGGCGAGAGAGGTGAACAAGGCCCTGCTGGCTCCCCCGGATTCCAGGGTCTCCCTGGTCCTGCTGGTCCTCCAGGTGAAGCAGGCAAACCTGGTGAACAGGGTGTTCCTGGAGACCTTGGCGCCCCTGGCCCCTCTGGAGCAAGAGGCGAGAGAGGTTTCCCTGGCGAGCGTGGTGTGCAAGGTCCCCCTGGTCCTGCTGGTCCCCGAGGGGCCAACGGTGCTCCCGGCAACGATGGTGCTAAGGGTGATGCTGGTGCCCCTGGAGCTCCCGGTAGCCAGGGCGCCCCTGGCCTTCAGGGAATGCCTGGTGAACGTGGTGCAGCTGGTCTTCCAGGGCCTAAGGGTGACAGAGGTGATGCTGGTCCCAAAGGTGCTGATGGCTCTCCTGGCAAAGATGGCGTCCGTGGTCTGACTGGCCCCATTGGTCCTCCTGGCCCTGCTGGTGCCCCTGGTGACAAGGGTGAAAGTGGTCCCAGCGGCCCTGCTGGTCCCACTGGAGCTCGTGGTGCCCCCGGAGACCGTGGTGAGCCTGGTCCCCCCGGCCCTGCTGGCTTTGCTGGCCCCCCTGGTGCTGACGGCCAACCTGGTGCTAAAGGCGAACCTGGTGATGCTGGTGCTAAAGGCGATGCTGGTCCCCCTGGCCCTGCCGGACCCGCTGGACCCCCTGGCCCCATTGGTAATGTTGGTGCTCCTGGAGCCAAAGGTGCTCGCGGCAGCGCTGGTCCCCCTGGTGCTACTGGTTTCCCTGGTGCTGCTGGCCGAGTCGGTCCTCCTGGCCCCTCTGGAAATGCTGGACCCCCTGGCCCTCCTGGTCCTGCTGGCAAAGAAGGCGGCAAAGGTCCCCGTGGTGAGACTGGCCCTGCTGGACGTCCTGGTGAAGTTGGTCCCCCTGGTCCCCCTGGCCCTGCTGGCGAGAAAGGATCCCCTGGTGCTGATGGTCCTGCT |
| hsa_circ_25045 | GGACCATTGGAATTTAGAGATGTGGCCATAGAATTCTCTCTGGAGGAGTGGCATTGCCTGGACACTGCACAGCAGAATTTATATAGGGATGTGATGTTAGAGAACTACAGACACCTGGTCTTCCTTGGTATTGTTGTCACTAAGCCAGACCTGATCACCTGTCTGGAGCAAGGAAAAAAACCCTTCACTGTGAAGAGACATGAGATGATTGCCAAATCCCCAG |
| hsa_circ_27127 | GAATTCCTTGTGGGCTACCCCCCACCATCACCAATGGAGATTTCATTAGCACCAACAGAGAGAATTTTCACTATGGATCAGTGGTGACCTACCGCTGCAATCCTGGAAGCGGAGGGAGAAAGGTGTTTGAGCTTGTGGGTGAGCCCTCCATATACTGCACCAGCAATGACGATCAAGTGGGCATCTGGAGCGGCCCCGCCCCTCAGTGCATTATACCTAACAAATGCACGCCTCCAAATGTGGAAAATGGAATATTGGTATCTGACAACAGAAGCTTATTTTCCTTAAATGAAGTTGTGGAGTTTAGGTGTCAGCCTGGCTTTGTCATGAAAGGACCCCGCCGTGTGAAGTGCCAGGCCCTGAACAAATGGGAGCCGGAGCTACCAAGCTGCTCCAGGGTATGTCAGCCACCTCCAGATGTCCTGCATGCTGAGCGTACCCAAAGGGACAAGGACAACTTTTCACCTGGGCAGGAAGTGTTCTACAGCTGTGAGCCCGGCTACGACCTCAGAGGGGCTGCGTCTATGCGCTGCACACCCCAGGGAGACTGGAGCCCTGCAGCCCCCACATGTGAAGTGAAATCCTGTGATGACTTCATGGGCCAACTTCTTAATGGCCGTGTGCTATTTCCAGTAAATCTCCAGCTTGGAGCAAAAGTGGATTTTGTTTGTGATGAAGGATTTCAATTAAAAGGCAGCTCTGCTAGTTACTGTGTCTTGGCTGGAATGGAAAGCCTTTGGAATAGCAGTGTTCCAGTGTGTGAACAAATCTTTTGTCCAAGTCCTCCAGTTATTCCTAATGGGAGACACACAGGAAAACCTCTGGAAGTCTTTCCCTTTGGGAAAACAGTAAATTACACATGCGACCCCCACCCAGACAGAGGGACGAGCTTCGACCTCATTGGAGAGAGCACCATCCGCTGCACAAGTGACCCTCAAGGGAATGGGGTTTGGAGCAGCCCTGCCCCTCGCTGTGGAATTCTGGGTCACTGTCAAGCCCCAGATCATTTTCTGTTTGCCAAGTTGAAAACCCAAACCAATGCATCTGACTTTCCCATTGGGACATCTTTAAAGTACGAATGCCGTCCTGAGTACTACGGGAGGCCATTCTCTATCACATGTCTAGATAACCTGGTCTGGTCAAGTCCCAAAGATGTCTGTAAACGTAAATCATGTAAAACTCCTCCAGATCCAGTGAATGGCATGGTGCATGTGATCACAGACATCCAGGTTGGATCCAGAATCAACTATTCTTGTACTACAGGGCACCGACTCATTGGTCACTCATCTGCTGAATGTATCCTCTCGGGCAATGCTGCCCATTGGAGCACGAAGCCGCCAATTTGTCAAC |
| hsa_circ_28956 | CTGTTTGGGGCAACTTCGTTAATATGAGCTTTCTACTCAACAGGTCTATCCAGGAAAATGGTGAACTAAAAATTGAAAGCAAGATTGAAGAGATGGTTGAACCACTAAGAGAGAAAATCAGAGATTTAGAAAAAAGCTTTACCCAGAAATACCCACCAGTAAAGTTTTTATCAGAAAAGGATCGGAAAAGAATTTTGATAACAGGAGGCGCAGGGTTCGTGGGCTCCCATCTAACTGACAAACTCATGATGGACGGCCACGAGGTGACCGTGGTGGACAATTTCTTCACGGGCAGGAAGAGAAACGTGGAGCACTGGATCGGACATGAGAACTTCGAGTTGATTAACCACGACGTGGTGGAGCCCCTCTACATCGAGG |
| hsa_circ_30924 | GCTGTGTGCTGTGAGGACCACATACACTGCTGTCCCGCGGGGTTTACGTGTGACACGCAGAAGGGTACCTGTGAACAGGGGCCCCACCAGGTGCCCTGGATGGAGAAGGCCCCAGCTCACCTCAGCCTGCCAGACCCACAAGCCTTGAAGAGAGATGTCCCCTGTGATAATGTCAGCAGCTGTCCCTCCTCCGATACCTGCTGCCAACTCACGTCTGGGGAGTGGGGCTGCTGTCCAATCCCAGAG |
| hsa_circ_32319 | AGGATCTTCGAAAGTACGTCACTACTATGGTGTGTGTGGCTGTAAATGGTAAACCCATGCTAGGAGTTATACATAAGCCATTTTCCGAATATACAG |
|  |  |

| **Supplementary Table 6. The putative peptides encoded by the ribosome associated circRNAs** | | |
| --- | --- | --- |
| **circRNA** | **Protein** | **Sequence** |
| circRNA010064 | circRNA010064_22aa | MPDRARKDGLSLFLVAETSLTS |
| circRNA010064 | circRNA010064_26aa | MDVITKDCLIGPERMGFPFSSWQKLP |
| circRNA011723 | circRNA011723_56aa | MEFDVNWSQPGEKKLTGGSNWQPKVAPTTAWNAATMAPPVMAYPATTPTGMIGYGI |
| circRNA011723 | circRNA011723_38aa | MATKGCTNNRLECCNNGTPCNGLSCYYTNRHDRIWNLM |
| circRNA011723 | circRNA011723_21aa | MAPPVMAYPATTPTGMIGYGI |
| circRNA011936 | circRNA011936_158aa | MQFLDSKHKNHYKIYNLCAERHYDTAKFNCRVAQYPFEDHNPPQLELIKPFCEDLDQWLSEDDNHVAAIHCKAGKGRTGVMICAYLLHRGKFLKAQEALDFYGEVRTRDKKGVTIPSQRRYVYYYSYLLKNHLDYRPVALLFHKMMFETIPMFSGGTC |
| circRNA011936 | circRNA011936_18aa | MKLFQCSVAELAVFGFKA |
| circRNA011936 | circRNA011936_198aa | MDSKHKNHYKIYNLCAERHYDTAKFNCRVAQYPFEDHNPPQLELIKPFCEDLDQWLSEDDNHVAAIHCKAGKGRTGVMICAYLLHRGKFLKAQEALDFYGEVRTRDKKGVTIPSQRRYVYYYSYLLKNHLDYRPVALLFHKMMFETIPMFSGGTCSFWIQSIKTITRYTIFVLKDIMTPPNLIAELHNILLKTITHHS |
| circRNA012383 | circRNA012383_167aa | MKFVLILEDLEGGILEQDAFHRSLLACCLEVVTFSYKPPGNFPFITEIFDVPLYHFYKVIEVFIRAEDGLCREVVKHLNQIEEQILDHLAWKPESPLWEKIRDNENRVPTCEEVMPPQNLERADEICIAGSPLTPRRVTEVRADTGGLGRRYSGTRCVPQISLGLLP |
| circRNA012383 | circRNA012383_153aa | MEQDAFHRSLLACCLEVVTFSYKPPGNFPFITEIFDVPLYHFYKVIEVFIRAEDGLCREVVKHLNQIEEQILDHLAWKPESPLWEKIRDNENRVPTCEEVMPPQNLERADEICIAGSPLTPRRVTEVRADTGGLGRRYSGTRCVPQISLGLLP |
| circRNA012651 | circRNA012651_17aa | MSEIAVESLQFSVEVYF |
| circRNA012651 | circRNA012651_691aa | MRSGFLGLHAAMDLDKPSVWGSLKQRTRPLLINLSKKKVKKNPSKPPDLRARHHLDRRLSLSVPDLLEAEALAPEGRPYSGPQSSYTSVPSSLSTAGIFPKSSSSSLKQSEEELDWSQEEASHLHVVETDSEEAYASPAERRRVSSNGIFDLQKTSLGGDAPEEPEKLCGSSDLNASMTSQHFEEQSVPGEASDGLSNLPSPFAYLLTIHLKEGRNLVVRDRCGVIAVFSRGVLLRSGFLGLHAAMDLDKPSVWGSLKQRTRPLLINLSKKKVKKNPSKPPDLRARHHLDRRLSLSVPDLLEAEALAPEGRPYSGPQSSYTSVPSSLSTAGIFPKSSSSSLKQSEEELDWSQEEASHLHVVETDSEEAYASPAERRRVSSNGIFDLQKTSLGGDAPEEPEKLCGSSDLNASMTSQHFEEQSVPGEASDGLSNLPSPFAYLLTIHLKEGRNLVVRDRCGVIAVFSRGVLLRSGFLGLHAAMDLDKPSVWGSLKQRTRPLLINLSKKKVKKNPSKPPDLRARHHLDRRLSLSVPDLLEAEALAPEGRPYSGPQSSYTSVPSSLSTAGIFPKSSSSSLKQSEEELDWSQEEASHLHVVETDSEEAYASPAERRRVSSNGIFDLQKTSLGGDAPEEPEKLCGSSDLNASMTSQHFEEQSVPGEASDGLSNLPSPFAYLLTIHLKEGRNLVVRDRC |
| circRNA012781 | circRNA012781_264aa | MQRLKVHMRTHNGEKPFMCHESGCGKQFTTAGNLKNHRRIHTGENVHLGSGDGQSKDSGPLPQVEKKLKCTVEGCDRTFVWPAHFKYHLKTHRNDRSFICPAEGCGKSFYVLQRLKVHMRTHNGEKPFMCHESGCGKQFTTAGNLKNHRRIHTGENVHLGSGDGQSKDSGPLPQVEKKLKCTVEGCDRTFVWPAHFKYHLKTHRNDRSFICPAEGCGKSFYVLQRLKVHMRTHNGEKPFMCHESGCGKQFTTAGNLKNHRRIHT |
| circRNA012959 | circRNA012959_532aa | MPKPYITINNSKPRENKDVLAFTCEPKSENYTYIWWLNGQSLPVSPRVKRPIENRILILPSVTRNETGPYECEIRDRDGGMRSDPVTLNVLYGPDLPSIYPSFTYYRSGENLYLSCFAESNPPAEYFWTINGKFQQSGQKLSIPQITTKHRGLYTCSVRNSATGKESSKSMTVEVSVKLPKPYITINNSKPRENKDVLAFTCEPKSENYTYIWWLNGQSLPVSPRVKRPIENRILILPSVTRNETGPYECEIRDRDGGMRSDPVTLNVLYGPDLPSIYPSFTYYRSGENLYLSCFAESNPPAEYFWTINGKFQQSGQKLSIPQITTKHRGLYTCSVRNSATGKESSKSMTVEVSVKLPKPYITINNSKPRENKDVLAFTCEPKSENYTYIWWLNGQSLPVSPRVKRPIENRILILPSVTRNETGPYECEIRDRDGGMRSDPVTLNVLYGPDLPSIYPSFTYYRSGENLYLSCFAESNPPAEYFWTINGKFQQSGQKLSIPQITTKHRGLYTCSVRNSATGKESSKSMTVEVS |
| circRNA014889 | circRNA014889_27aa | MQLVEMEDNGLSTSLLNSGECRALALG |
| circRNA014889 | circRNA014889_30aa | MQGVGFRLTLAEYHEQEEIFKLRLGHLKKR |
| circRNA015581 | circRNA015581_300aa | MVMASHPLNPLWLLLLRGSDELLSSGIINGPFTMNSSTPSTANGNDSKKFKRDRPPCSPSRVLHLRKIPCDVTEAEIISLGLPFGKVTNLLMLKGKSQAFLEMASEEAAVTMVNYYTPITPHLRSQPVYIQYSNHRELKTDNLPNQARAQAALQAVSAVQSGSLALSGGPSNEGTVLPGQSPVLRIIIENLFYPVTLEVLHQIFSKFGTVLKIITFTKNNQFQALLQYADPVNAHYAKMALDGQNIYNACCTLRIDFSKLTSLNVKYNNDKSRDFTRLDLPTGDGQPSLEPPMAAAFAGI |
| circRNA015581 | circRNA015581_267aa | MNSSTPSTANGNDSKKFKRDRPPCSPSRVLHLRKIPCDVTEAEIISLGLPFGKVTNLLMLKGKSQAFLEMASEEAAVTMVNYYTPITPHLRSQPVYIQYSNHRELKTDNLPNQARAQAALQAVSAVQSGSLALSGGPSNEGTVLPGQSPVLRIIIENLFYPVTLEVLHQIFSKFGTVLKIITFTKNNQFQALLQYADPVNAHYAKMALDGQNIYNACCTLRIDFSKLTSLNVKYNNDKSRDFTRLDLPTGDGQPSLEPPMAAAFAGI |
| circRNA015584 | circRNA015584_37aa | MQGCMTNVLISHVTEDGSWAFTPSVTKTTKTHATFSP |
| circRNA015584 | circRNA015584_1001aa | LYDKCSYISRDRGWVVGIHTISDQDNKDPRYFFSLKTDRARQVTTINAHRSYLPGQWVYLAATYDGQFMKLYVNGAQVATSGEQVGGIFSPLTQKCKVLMLGGSALNHNYRGYIEHFSLWKVARTQREILSDMETHGAHTALPQLLLQENWDNVKHAWSPMKDGSSPKVEFSNAHGFLLDTSLEPPLCGQTLCDNTEVIASYNQLSSFRQPKVVRYRVVNLYEDDHKNPTVTREQVDFQHHQLAEAFKQYNISWELDVLEVSNSSLRRRLILANCDISKIGDENCDPECNHTLTGHDGGDCRHLRHPAFVKKQHNGVCDMDCNYERFNFDGGECCDPEITNVTQTCFDPDSPHRAYLDVNELKNILKLDGSTHLNIFFAKSSEEELAGVATWPWDKEALMHLGGIVLNPSFYGMPGHTHTMIHEIGHSLGLYHVFRGISEIQSCSDPCMETEPSFETGDLCNDTNPAPKHKSCGDPGPGNDTCGFHSFFNTPYNNFMSYAGLYDKCSYISRDRGWVVGIHTISDQDNKDPRYFFSLKTDRARQVTTINAHRSYLPGQWVYLAATYDGQFMKLYVNGAQVATSGEQVGGIFSPLTQKCKVLMLGGSALNHNYRGYIEHFSLWKVARTQREILSDMETHGAHTALPQLLLQENWDNVKHAWSPMKDGSSPKVEFSNAHGFLLDTSLEPPLCGQTLCDNTEVIASYNQLSSFRQPKVVRYRVVNLYEDDHKNPTVTREQVDFQHHQLAEAFKQYNISWELDVLEVSNSSLRRRLILANCDISKIGDENCDPECNHTLTGHDGGDCRHLRHPAFVKKQHNGVCDMDCNYERFNFDGGECCDPEITNVTQTCFDPDSPHRAYLDVNELKNILKLDGSTHLNIFFAKSSEEELAGVATWPWDKEALMHLGGIVLNPSFYGMPGHTHTMIHEIGHSLGLYHVFRGISEIQSCSDPCMETEPSFETGDLCNDTNPAPKHKSCGDPGPGNDTCGFHSFFNTPYNNFMSYA |
| circRNA01630 | circRNA01630_556aa | MTLISAAREYEIEFIYAISPGLDITFSNPKEVSTLKRKLDQVSQFGCRSFALLFDDIDHNMCAADKEVFSSFAHAQVSITNEIYQYLGEPETFLFCPTEYCGTFCYPNVSQSPYLRTVGEKLLPGIEVLWTGPKVVSKEIPVESIEEVSKIIKRAPVIWDNIHANDYDQKRLFLGPYKGRSTELIPRLKGVLTNPNCEFEANYVAIHTLATWYKSNMNGVRKDVVMSRQVAHSGAKASVVDGTPLVAAPSLNATTVVTTVYQEPIMSQGAALSGEPTTLTKEEEKKQPDEEPMDMVVEKQEETDHKNDNQILSEIVEAKMAEELKPMDTDKESIAESKSPEMSMQEDCISDIAPMQTDEQTNKEQFVPGPNEKPLYTAEPVTLEDLQLLADLFYLPYEHGPKGAQMLREFQWLRANSSVVSVNCKGKDSEKIEEWRSRAAKFEEMCGLVMGMFTRLSNCANRTILYDMYSYVWDIKSIMSMVKSFVQWLGCRSHSSAQFLIGDQEPWAFRGGLAGEFQRLLPIDGANDLFFQPPPLTPTSKVYTIRPYFPKDESNL |
| circRNA01630 | circRNA01630_14aa | MRATYDSHLCCTRI |
| circRNA016607 | circRNA016607_37aa | MYFLCPSIRKKIAVWFELEFLAMSHWRFSLEVMLNQH |
| circRNA016607 | circRNA016607_60aa | MRCHEFLFPYCQDCPEPSQALDFYYLSCISSAHPLGRRLLSGLNWNFSLCPTGGSLWKLC |
| circRNA016607 | circRNA016607_23aa | MEVLSGSYVESTLKGQRGSRETN |
| circRNA01724 | circRNA01724_714aa | MKSATNRYKLFPAKKLKSVRGSCGSHHNTPNLAAKNVFPPPSQTWARRHKRETLKATKYVELVIVADNREFQRQGKDLEKVKQRLIEIANHVDKFYRPLNIRIVLVGVEVWNDMDKCSVSQDPFTSLHEFLDWRKMKLLPRKSHDNAQLVSGVYFQGTTIGMAPIMSMCTADQSGGIVMDHSDNPLGAAVTLAHELGHNFGMNHDTLDRGCSCQMAVEKGGCIMNASTGGLIVFENESYVLEPMKSATNRYKLFPAKKLKSVRGSCGSHHNTPNLAAKNVFPPPSQTWARRHKRETLKATKYVELVIVADNREFQRQGKDLEKVKQRLIEIANHVDKFYRPLNIRIVLVGVEVWNDMDKCSVSQDPFTSLHEFLDWRKMKLLPRKSHDNAQLVSGVYFQGTTIGMAPIMSMCTADQSGGIVMDHSDNPLGAAVTLAHELGHNFGMNHDTLDRGCSCQMAVEKGGCIMNASTGGLIVFENESYVLEPMKSATNRYKLFPAKKLKSVRGSCGSHHNTPNLAAKNVFPPPSQTWARRHKRETLKATKYVELVIVADNREFQRQGKDLEKVKQRLIEIANHVDKFYRPLNIRIVLVGVEVWNDMDKCSVSQDPFTSLHEFLDWRKMKLLPRKSHDNAQLVSGVYFQGTTIGMAPIMSMCTADQSGGIVMDHSDNPLGAAVTLAHELGHNFGMNHDTLDRGCSCQMAVEKGGCIMNAST |
| circRNA01724 | circRNA01724_13aa | MHHERFHRGTYCV |
| circRNA017254 | circRNA017254_59aa | MFKRDGVSLCCPGWSAVAIHRRDPTTDQHGSFDLLRFRPGPVHPSLGNLVVPRSRECHW |
| circRNA018689 | circRNA018689_222aa | MLEIYEKDEKIQKHLQDSLADLKSLYNEWGCTNYINLGSFLIKPVQRVMRYPLLLMELLNSTPESHPDKVPLTNAVLAVKEINVNINEYKRRKDLVLKYRKGDEDSLMEKISKLNIHSIIKKSNRVSSHLKHLTGFAPQIKDEVFEETEKNFRMQERLIKSFIRDLSLYLQHIRDLCFLVTGMSLREHTRFTARIMMRPLRCLKSTRRMRRSRSIFRTPWQI |
| circRNA018769 | circRNA018769_19aa | MTMRSLSGDLLCLKMKAMS |
| circRNA018769 | circRNA018769_480aa | MKSATNRYKLFPAKKLKSVRGSCGSHHNTPNLAAKNVFPPPSQTWARRHKRETLKATKYVELVIVADNREFQRQGKDLEKVKQRLIEIANHVDKFYRPLNIRIVLVGVEVWNDMDKCSVSQDPFTSLHEFLDWRKMKLLPRKSHDNAQLVRGLIVFENESYVLEPMKSATNRYKLFPAKKLKSVRGSCGSHHNTPNLAAKNVFPPPSQTWARRHKRETLKATKYVELVIVADNREFQRQGKDLEKVKQRLIEIANHVDKFYRPLNIRIVLVGVEVWNDMDKCSVSQDPFTSLHEFLDWRKMKLLPRKSHDNAQLVRGLIVFENESYVLEPMKSATNRYKLFPAKKLKSVRGSCGSHHNTPNLAAKNVFPPPSQTWARRHKRETLKATKYVELVIVADNREFQRQGKDLEKVKQRLIEIANHVDKFYRPLNIRIVLVGVEVWNDMDKCSVSQDPFTSLHEFLDWRKMKLLPRKSHDNAQLV |
| circRNA018853 | circRNA018853_216aa | MVGLRWWNHIDEDGKSHWVFESRKESSQENKTVSEAESRIFWLGLIACSVLWVIFAFSALFSFTVKWLNVTGRLMVGLRWWNHIDEDGKSHWVFESRKESSQENKTVSEAESRIFWLGLIACSVLWVIFAFSALFSFTVKWLNVTGRLMVGLRWWNHIDEDGKSHWVFESRKESSQENKTVSEAESRIFWLGLIACSVLWVIFAFSALFSFTVKWL |
| circRNA020003 | circRNA020003_142aa | MRLCLPANPKNCLPHRRGISILEKLIKTCPVWLQLSLGQAEVARILHRVVAGMFLVRRDSSSKQLVLCVHFPSLNESSAEVLEYTIKEEKSIRFQLLAKERKRKRKMACGFVCQPTRKTAFLTAGASASWRSSSKHARCGCS |
| circRNA020175 | circRNA020175_261aa | MEAVSLTCDPETPDASYLWWMNGQSLPMSHRLQLSETNRTLFLLGVTKYTAGPYECEIRNPVSASRSDPFTLNLLLETPKPSISSSKLNPREAMEAVSLTCDPETPDASYLWWMNGQSLPMSHRLQLSETNRTLFLLGVTKYTAGPYECEIRNPVSASRSDPFTLNLLLETPKPSISSSKLNPREAMEAVSLTCDPETPDASYLWWMNGQSLPMSHRLQLSETNRTLFLLGVTKYTAGPYECEIRNPVSASRSDPFTLNLL |
| circRNA020827 | circRNA020827_70aa | MGQQPQPRCLTLLSMMQQEAPGECFSPSLYYCHVLITVWWNLNDLLSRWINASFPVLEYRKWMDKDCAAF |
| circRNA020827 | circRNA020827_24aa | MDKRKFPSAGIQKMDGQGLCCILT |
| circRNA021462 | circRNA021462_255aa | MVIAHTKALDPSQPVTFVTNSTYAADKGALYVDVIRVNSYYSWYRNYGHLELIQLQLAAQFENWCKTSQSHYSERVWSGNACRVSPGSTLMFSEEYQKSLLEQYHLGLNQKRRKYVVGELIWNFADFMTNQWLVNYQISVKCSNQFKLEVCLLKSENKVVDNQAGTQGQLKVLGANLWWPYLMHEHPAYLYSWEDGDCSHQSLGPLPACDLCDQLHLRSRQGGSVCGCDPCEQLLLLVSQLRAPGVDSAAAGRPV |
| circRNA021573 | circRNA021573_195aa | MRIGVRHHNPIIQSAYGVETLVGLHQDPPLMFSEEDRKSLLEQYHLGLDQKRRKYVVGELIWNFADFMTNQWLVNYQISVKCSNQFKLEVCLLNAENKVVDNQAGTQGQLKVLGANLWWPYLMHEHPAYLYSWEDGDCSHQSLGPLPACDLCDQLHLRSRQGGSVCGCDPCEQLLLLVSQLRAPGVDSAAAGRPV |
| circRNA021696 | circRNA021696_58aa | MWMEEISMNVLWILIYVPMGFVKTYVVVTVVIATVAMNQMPLEETVLMLMNARPQESA |
| circRNA021696 | circRNA021696_445aa | MNGHCINSEGSFRCDCPPGLAVGMDGRVCVDTHMRSTCYGGIKKGVCVRPFPGAVTKSECCCANPDYGFGEPCQPCPAKNSAEFHGLCSSGVGITVDGRDINECALDPDICANGICENLRGSYRCNCNSGYEPDASGRNCIDVDECQTPGICMNGHCINSEGSFRCDCPPGLAVGMDGRVCVDTHMRSTCYGGIKKGVCVRPFPGAVTKSECCCANPDYGFGEPCQPCPAKNSAEFHGLCSSGVGITVDGRDINECALDPDICANGICENLRGSYRCNCNSGYEPDASGRNCIDVDECQTPGICMNGHCINSEGSFRCDCPPGLAVGMDGRVCVDTHMRSTCYGGIKKGVCVRPFPGAVTKSECCCANPDYGFGEPCQPCPAKNSAEFHGLCSSGVGITVDGRDINECALDPDICANGICENLRGSYRCNCNSGYEPDASGRNCI |
| circRNA024119 | circRNA024119_2076aa | MMCVPLDCGKPPPIQNGFMKGENFEVGSKVQFFCNEGYELVGDSSWTCQKSGKWNKKSNPKCMPAKCPEPPLLENQLVLKELTTEVGVVTFSCKEGHVLQGPSVLKCLPSQQWNDSFPVCKIVLCTPPPLISFGVPIPSSALHFGSTVKYSCVGGFFLRGNSTTLCQPDGTWSSPLPECVPVECPQPEEIPNGIIDVQGLAYLSTALYTCKPGFELVGNTTTLCGENGHWLGGKPTCKAIECLKPKEILNGKFSYTDLHYGQTVTYSCNRGFRLEGPSALTCLETGDWDVDAPSCNAIHCDSPQPIENGFVEGADYSYGAIIIYSCFPGFQVAGHAMQTCEESGWSSSIPTCMPIDCGLPPHIDFGDCTKLKDDQGYFEQEDDMMEVPYVTPHPPYHLGAVAKTWENTKESPATHSSNFLYGTMVSYTCNPGYELLGNPVLICQEDGTWNGSAPSCISIECDLPTAPENGFLRFTETSMGSAVQYSCKPGHILAGSDLRLCLENRKWSGASPRCEAISCKKPNPVMNGSIKGSNYTYLSTLYYECDPGYVLNGTERRTCQDDKNWDEDEPICIPVDCSSPPVSANGQVRGDEYTFQKEIEYTCNEGFLLEGARSRVCLANGSWSGATPDCVPVRCATPPQLANGVTEGLDYGFMKEVTFHCHEGYILHGAPKLTCQSDGNWDAEIPLCKPVNCGPPEDLAHGFPNGFSFIHGGHIQYQCFPGYKLHGNSSRRCLSNGSWSGSSPSCLPCRCSTPVIEYGTVNGTDFDCGKAARIQCFKGFKLLGLSEITCEADGQWSSGFPHCEHTSCGSLPMIPNAFISETSSWKENVITYSCRSGYVIQGSSDLICTEKGVWSQPYPVCEPLSCGSPPSVANAVATGEAHTYESEVKLRCLEGYTMDTDTDTFTCQKDGRWFPERISCSPKKCPLPENITHILVHGDDFSVNRQVSVSCAEGYTFEGVNISVCQLDGTWEPPFSDESCSPVSCGKPESPEHGFVVGSKYTFESTIIYQCEPGYELEHTTGRIFESEVRYQCNPGYKSVGSPVFVCQANRHWHSESPLMCVPLDCGKPPPIQNGFMKGENFEVGSKVQFFCNEGYELVGDSSWTCQKSGKWNKKSNPKCMPAKCPEPPLLENQLVLKELTTEVGVVTFSCKEGHVLQGPSVLKCLPSQQWNDSFPVCKIVLCTPPPLISFGVPIPSSALHFGSTVKYSCVGGFFLRGNSTTLCQPDGTWSSPLPECVPVECPQPEEIPNGIIDVQGLAYLSTALYTCKPGFELVGNTTTLCGENGHWLGGKPTCKAIECLKPKEILNGKFSYTDLHYGQTVTYSCNRGFRLEGPSALTCLETGDWDVDAPSCNAIHCDSPQPIENGFVEGADYSYGAIIIYSCFPGFQVAGHAMQTCEESGWSSSIPTCMPIDCGLPPHIDFGDCTKLKDDQGYFEQEDDMMEVPYVTPHPPYHLGAVAKTWENTKESPATHSSNFLYGTMVSYTCNPGYELLGNPVLICQEDGTWNGSAPSCISIECDLPTAPENGFLRFTETSMGSAVQYSCKPGHILAGSDLRLCLENRKWSGASPRCEAISCKKPNPVMNGSIKGSNYTYLSTLYYECDPGYVLNGTERRTCQDDKNWDEDEPICIPVDCSSPPVSANGQVRGDEYTFQKEIEYTCNEGFLLEGARSRVCLANGSWSGATPDCVPVRCATPPQLANGVTEGLDYGFMKEVTFHCHEGYILHGAPKLTCQSDGNWDAEIPLCKPVNCGPPEDLAHGFPNGFSFIHGGHIQYQCFPGYKLHGNSSRRCLSNGSWSGSSPSCLPCRCSTPVIEYGTVNGTDFDCGKAARIQCFKGFKLLGLSEITCEADGQWSSGFPHCEHTSCGSLPMIPNAFISETSSWKENVITYSCRSGYVIQGSSDLICTEKGVWSQPYPVCEPLSCGSPPSVANAVATGEAHTYESEVKLRCLEGYTMDTDTDTFTCQKDGRWFPERISCSPKKCPLPENITHILVHGDDFSVNRQVSVSCAEGYTFEGVNISVCQLDGTWEPPFSDESCSPVSCGKPESPEHGFVVGSKYTFESTIIYQCEPGYELE |
| circRNA025171 | circRNA025171_62aa | MSPAIFICRLMEVLRNLGSSGAPQTSRMPVPMSAKNRPGTLDKPGKQSKLQDPRQYRQVVLP |
| circRNA025171 | circRNA025171_66aa | MQANGSAKKSGLQRGPTDEQDACPHECQEQTRNPGQTRQAVQTAGSPPISSGSFTLNPLLDGRYFS |
| circRNA026414 | circRNA026414_15aa | MSSVEERATSTSEWQ |
| circRNA026414 | circRNA026414_353aa | MPNEQDLVREEAQKMSSLLPTMWLGAQNGCLYVHSSVAQWRKCLHSIKLKDSILSIVHVKGIVLVALADGTLAIFHRGVDGQWDLSNYHLLDLGRPHHSIRCMTVVHDKVWCGYRNKIYVVQPKAMKIEKSFDAHPRKESQVRQLAWVGDGVWVSIRLDSTLRLYHAHTYQHLQDVDIEPYVSKMLGTGKLGFSFVRITALMVSCNRLWVGTGNGVIISIPLTETNKTSGVPGNRPGSVIRVYGDENSDKVTPGTFIPYCSMAHAQLCFHGHRDAVKFFVAVPGQVISPQSSSSGTDLTGDKAGPSAQEPGSQTPLKSMLVISGGEGYIDFRMAMTQMHIKIKYQYCQMNKTW |
| circRNA026955 | circRNA026955_688aa | MCFSGLVNGRCAQELPGRMTKMQCCCEPGRCWGIGTIPEACPVRGSEEYRRLCMDGLPMGGIPGSAGSRPGGTGGNGFAPSGNGNGYGPGGTGFIPIPGGNGFSPGVGGAGVGAGGQGPIITGLTILNQTIDICKHHANLCLNGRCIPTVSSYRCECNMGYKQDANGDCIDVDECQAIPGICQGGNCINTVGSFECRCPAGHKQSETTQKCEDIDECSIIPGICETGECSNTVGSYFCVCPRGYVTSTDGSRCIDQRTGMCFSGLVNGRCAQELPGRMTKMQCCCEPGRCWGIGTIPEACPVRGSEEYRRLCMDGLPMGGIPGSAGSRPGGTGGNGFAPSGNGNGYGPGGTGFIPIPGGNGFSPGVGGAGVGAGGQGPIITGLTILNQTIDICKHHANLCLNGRCIPTVSSYRCECNMGYKQDANGDCIDVDECQAIPGICQGGNCINTVGSFECRCPAGHKQSETTQKCEDIDECSIIPGICETGECSNTVGSYFCVCPRGYVTSTDGSRCIDQRTGMCFSGLVNGRCAQELPGRMTKMQCCCEPGRCWGIGTIPEACPVRGSEEYRRLCMDGLPMGGIPGSAGSRPGGTGGNGFAPSGNGNGYGPGGTGFIPIPGGNGFSPGVGGAGVGAGGQGPIITGLTILNQTIDICKHHANLCLNGRCIPTVSSYRCECNMGYKQDANGDCI |
| circRNA028717 | circRNA028717_52aa | MEPHCRENRPSPISLSQASLAACGGSISRRSLAWSCGARASGRGNVLRTPWI |
| circRNA028717 | circRNA028717_280aa | MAGHGASGLQSQYFGRPSAWIKVEQLKPYHAHKEEMIKINKGKRFQQAVDAVEEFLRRAKGKDQTSSHNSSDDKNRRNSSEERSRPNSGDEKRKLSLSEGKVKKNMGEGKKRVSSGSSERGSKSPLKRAQEQSPRKRGRPPKDEKDLTIPESSTVKGMMAGPMAAFKWQPTASEPVKDADPHFHHFLLSQTEKPAVCYQAITKKLKICEEETGSTSIQAADSTAVNGSITPTDKKIGFLGLGLMGSGIVSNLLKMGHTVTVWNRTAEKIDPAPFLYHKPA |
| circRNA03251 | circRNA03251_27aa | MIRNRRDWIWSFPQGEEHGDFFCGKEQ |
| circRNA03251 | circRNA03251_21aa | MAISSVVRSNNENHVAGLIIL |
| circRNA032706 | circRNA032706_203aa | MEESLYLQLDPAIPGVYFGNPYPFGIDPIWNLASNKLTFLNSYKMKMSVILGIVQMVFGVILSLFNHIYFRRTLNIILQFIPEMIFILCLFGYLVFMIIFKWCCFDVHVSQHAPSILIHFINMFLFNYSDSSNAPLYKHQQEVQSFFVVMALISVPWMLLIKPFILRASHRKSQYSCNGGKSISAAGPSHTRSVFWKSIPVWD |
| circRNA032774 | circRNA032774_74aa | MGCCFSRAYPTPLRSKKTMLVQKNVTSESTCCVAKSYNRIAQNARYRKTHSSPSRVPQYFSAWAAASLEHIPLH |
| circRNA033514 | circRNA033514_72aa | MRWPDMKESRLPPPGAAQWLPCPSSAGAGSNVRNGSHCDHPNLAKQATWGCSKCSKKNWDQPTLEAVCPGVL |
| circRNA033514 | circRNA033514_60aa | MPSGCLVLQVQELVQMSGMEATVTIPIWQNKPHGAARSVVRRIGTNLPLKPCARASFEMA |
| circRNA03380 | circRNA03380_209aa | LDRFEKTNEMLLNFNNLSSARLQQMSERFLHHTRTLVEMKRDLDSIFRRIRLHCALKCLGQFQPWDKILTLDRFEKTNEMLLNFNNLSSARLQQMSERFLHHTRTLVEMKRDLDSIFRRIRLHCALKCLGQFQPWDKILTLDRFEKTNEMLLNFNNLSSARLQQMSERFLHHTRTLVEMKRDLDSIFRRIRLHCALKCLGQFQPWDKIL |
| circRNA034136 | circRNA034136_134aa | MLQSTIVCHNRVDPNGSRYLLGDMEGRLFMLLLEKEEQMDGTVTLKDLRVELLGEDPQGRHVKTYEVSLREKEFNKGPWKQENVEAEASMVIAVPEPFGGAIIIGQESITYHNGDKYLAIAPPIIKNDDISVNI |
| circRNA03459 | circRNA03459_233aa | MSCSADSNPPAQYSWTINEKFQLPGQKLFIRHITTKHSGLYVCSVRNSATGKESSKSMTVEVSDGPDLPRIYPSFTYYRSGEVLYLSCSADSNPPAQYSWTINEKFQLPGQKLFIRHITTKHSGLYVCSVRNSATGKESSKSMTVEVSDGPDLPRIYPSFTYYRSGEVLYLSCSADSNPPAQYSWTINEKFQLPGQKLFIRHITTKHSGLYVCSVRNSATGKESSKSMTVEVS |
| circRNA03459 | circRNA03459_80aa | MMVQTSPEFTLHSPITVQEKSSTCPVLRTLTHRHSILGQLMKSFSYQDKSSLSAILLQSIAGSMFALFVTQPLARKAPNP |
| circRNA03460 | circRNA03460_233aa | MSCFADSNPPAEYSWTINGKFQLSGQKLFIPQITTNHSGLYACSVRNSATGKEISKSMIVKVSDGPDLPRIYPSFTYYRSGENLDLSCFADSNPPAEYSWTINGKFQLSGQKLFIPQITTNHSGLYACSVRNSATGKEISKSMIVKVSDGPDLPRIYPSFTYYRSGENLDLSCFADSNPPAEYSWTINGKFQLSGQKLFIPQITTNHSGLYACSVRNSATGKEISKSMIVKVS |
| circRNA03460 | circRNA03460_80aa | MMVQTSPEFTLHSPITVQEKTSTCPALRTLTHRQSILGQLMGSFSYQDKSSLSPKLLQIIAGSMLALFVTQPLARKSPNP |
| circRNA03462 | circRNA03462_209aa | MPTLNEGCLWRNQRCHTGQSSVIHTAKLPMPYITINNLNPREKKDVLAFTCEPKSRNYTYIWWLNGQSLPVSPRVKRPIENRILILPSVTRNETGPYQCEIRDRYGGIRSNPVTLNVLYGPDLPRIYPSFTYYRSGENLDLSCFADSNPPAEYSWTINGKFQLSGQKLFIPQITTNHSGLYACSVRNSATGKEISKSMIVKVSGGRCQL |
| circRNA03465 | circRNA03465_261aa | METVILTCNPETPDASYLWWMNGQSLPMTHRMQLSETNRTLFLFGVTKYTAGPYECEIWNSGSASRSDPVTLNLLLETPKPSISSSNLNPREAMETVILTCNPETPDASYLWWMNGQSLPMTHRMQLSETNRTLFLFGVTKYTAGPYECEIWNSGSASRSDPVTLNLLLETPKPSISSSNLNPREAMETVILTCNPETPDASYLWWMNGQSLPMTHRMQLSETNRTLFLFGVTKYTAGPYECEIWNSGSASRSDPVTLNLL |
| circRNA03467 | circRNA03467_261aa | MEAVRLICDPETLDASYLWWMNGQSLPVTHRLQLSKTNRTLYLFGVTKYIAGPYECEIRNPVSASRSDPVTLNLLLETPKPYISSSNLNPREAMEAVRLICDPETLDASYLWWMNGQSLPVTHRLQLSKTNRTLYLFGVTKYIAGPYECEIRNPVSASRSDPVTLNLLLETPKPYISSSNLNPREAMEAVRLICDPETLDASYLWWMNGQSLPVTHRLQLSKTNRTLYLFGVTKYIAGPYECEIRNPVSASRSDPVTLNLL |
| circRNA034835 | circRNA034835_59aa | MGHLTRIANCIVHSTDKGPNSALVQQLIKDLPDEVRERWETFCTSSLGETNKRNTVDLG |
| circRNA03512 | circRNA03512_13aa | MVLGYLRQWQLLD |
| circRNA03512 | circRNA03512_99aa | MTLDTQAASPTQSQQQTLLQTFPGSQSHTHHHGPGIPQTMAAFGLKYLLNDTGYTSSKSNTITATDSPADLPRKPEPHTPSWSWDTSDNGSFWIEIFAE |
| circRNA03512 | circRNA03512_60aa | MAAFGLKYLLNDTGYTSSKSNTITATDSPADLPRKPEPHTPSWSWDTSDNGSFWIEIFAE |
| circRNA03556 | circRNA03556_164aa | MRNPSDRKVCFKVKTTAPRRYCVRPNSGIIDPGSTVTVSVMLQPFDYDPNEKSKHKFMVQTIFAPPNTSDMEAVWKEAKPDELMDSKLRCVFEMPNENDKLNDMEPSKAVPLNASKQDGPMPKPHSVSLNDTETRKLMEECKRLQGEMMKLSEENRHLRAPSQM |
| circRNA036250 | circRNA036250_165aa | MRIGVRHHNPIIQSAYGVETLVGFHQDPPLMFSEEYQKSLLEQYHLGLDQKRRKYVVGKLIWNFADFMTNQWLVNYQISIKCSNQFKLEVCLLNAENKVVDNQAGTQGQLKVLGANLWWPYLMHEHPAYLYSWEVRLTAQKSLGPLTSTHSLWGSALCPSPRARW |
| circRNA036366 | circRNA036366_82aa | MVAVPDARTPRLPVLVGGLVNYHISVKCSNQFKLEVCLLKSDNKVVDNQAGTQGQLKVLGANLWWPYLMHEHPAYLYSWEGW |
| circRNA036366 | circRNA036366_64aa | LVNYHISVKCSNQFKLEVCLLKSDNKVVDNQAGTQGQLKVLGANLWWPYLMHEHPAYLYSWEGW |
| circRNA03794 | circRNA03794_134aa | MQRFPSGSLLLPGTMTVRGDVLAPDPASPTTAAASPSVSVIPEGSPTAMEQPVFLMTTAAQAISGFFVWTALLITCHQIYMHLRCYSCPNEQRYIVRILFIVPIYAFDSWLSLLFFTNDQYYVYFGTVRDCYEG |
[truncated: 254,646 more chars]
